# Photochemical Production of Singlet Oxygen in Adirondack Long-Term Monitoring Lakes of Varying Browning Status

**DOI:** 10.1021/acs.est.5c04001

**Published:** 2025-07-02

**Authors:** Birdem Öz, Philip K. Snyder, Xiaoyu Jiao, Charles T. Driscoll, Teng Zeng

**Affiliations:** † Department of Civil and Environmental Engineering, 2029 Syracuse University, 151 Link Hall, Syracuse, New York 13244, United States; ‡ Ausable Freshwater Center, 5698 NY-86, Wilmington, New York 12997, United States

**Keywords:** browning, DOM, photochemistry, singlet oxygen, temperate lakes

## Abstract

Widespread browning of surface waters in boreal and temperate regions of the Northern Hemisphere has been documented through long-term monitoring of color or dissolved organic matter (DOM) over recent decades. While the ecological implications of browning have received considerable attention, its impacts on the photochemical production of reactive intermediates from DOM remain understudied despite their importance for biogeochemical processes and contaminant fate in sunlit surface waters. To address this gap, we investigated singlet oxygen (^1^O_2_) production in 37 lakes within the Adirondack Long-Term Monitoring (ALTM) program. Wavelet coherence tests confirmed the synchrony between dissolved organic carbon (DOC) and color as well as the time-scale-dependent influence of regional atmospheric and hydroclimatic factors on DOC dynamics in these lakes. Hydrogeological conditions of lake watersheds (e.g., hydrologic connectivity and surficial geology) and seasonal variations in DOM quality jointly shaped the spatiotemporal patterns of the apparent quantum yields of ^1^O_2_. Within the euphotic zone, depth-averaged steady-state concentrations of ^1^O_2_ were higher in lakes experiencing more intense browning, as operationally defined through trend analyses of long-term water chemistry data; however, the relevance of ^1^O_2_-mediated reactions depends on the time scales of photochemical transformation relative to lake flushing. Overall, our study provides an initial assessment of ^1^O_2_ production in relation to lake browning and highlights the need for long-term photochemical measurements for an improved assessment.

## Graphical abstract

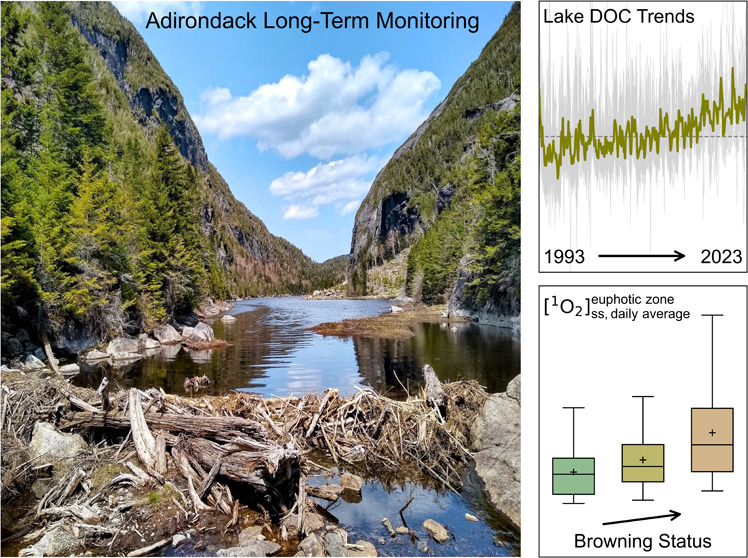

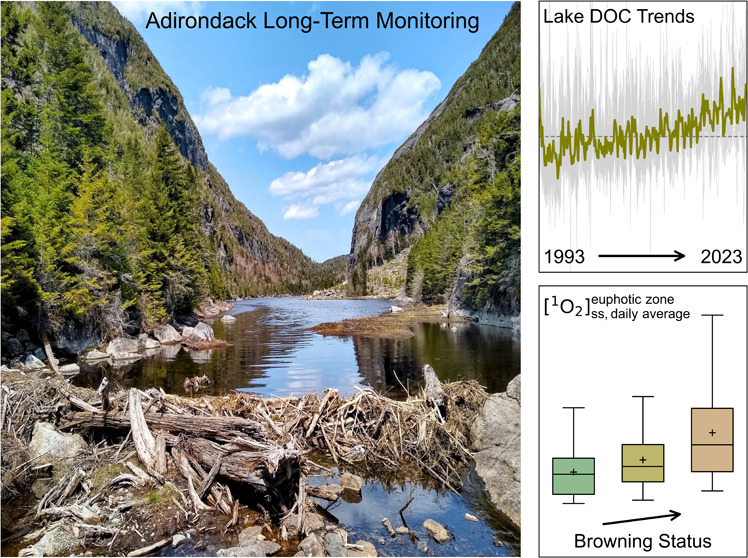

## Introduction

Many surface water systems in the temperate and boreal regions of North America and Europe have experienced varying degrees of browning in recent decades—a shift in optical properties that gives the water a brownish hue and is often attributed to increasing concentrations of dissolved organic matter (DOM), as indicated by long-term records of color or dissolved organic carbon (DOC).^1–3^ Multiple anthropogenic and hydroclimatic factors, including reduced atmospheric acid deposition,^4–6^ changing precipitation regimes,^7,8^ rising atmospheric temperatures and CO_2_,^9,10^ and evolving land use patterns (e.g., afforestation^11^ and ditching^12^), have been put forward as potential drivers of browning, though their relative importance and interactions remain difficult to disentangle and may be further complicated by landscape heterogeneity.^13^ Collectively, previous studies have established that browning has profound impacts on lake ecosystem functioning (e.g., carbon budgets,^14^ primary productivity,^15^ oxythermal habitat conditions^16^)^17,18^ while also posing operational challenges for water treatment by increasing coagulant demand, energy consumption, and byproduct formation.^19–23^

Considering the effects of browning on light attenuation and thermal stratification, prior research has explored its implications for the photochemical production of reactive intermediates from DOM in sunlit lakes.^24^ For example, modeling of boreal lakes in Sweden projected that browning would lead to increases in steady-state concentrations of excited triplet states of DOM (^3^DOM*) and singlet oxygen (^1^O_2_) down to 5 cm and the mean depth of each lake, thereby accelerating the indirect photodegradation of compounds that primarily react with these reactive intermediates.^25^ Modeling of a smaller set of Nordic lakes also predicted that browning would enhance the formation of reactive oxygen species in the upper centimeters of the water column while inhibiting their production in deeper layers.^26^ Together, these and related efforts^27,28^ provided a theoretical assessment of reactive intermediate formation in the context of lake browning. Nonetheless, their modeling framework did not incorporate long-term monitoring records to define the extent of browning and relied on literature data or reference materials to approximate the apparent quantum yields of reactive intermediates instead of lake-specific measurements.

To address this gap, we investigated the production of ^1^O_2_ in the Adirondack Long-Term Monitoring (ALTM) lakes by integrating time series and trend analyses of long-term data sets with photochemical characterization of field-collected samples. Historically, the Adirondack region of New York was a hotspot for atmospheric acid deposition from industrial emissions.^5^ To assess the recovery of Adirondack lakes from acidification, the ALTM program was launched in 1992 to monitor surface water chemistry in 52 lakes selected from a large-scale survey of 1469 lakes conducted between 1984 and 1987.^29,30^ Lakes within the ALTM program thus represent an ideal set of acid-impacted systems draining remote forested watersheds for studying photochemistry in relation to browning. Leveraging long-term data sets, we applied wavelet coherence tests to examine synchronous dynamics between the time series of DOC and regional drivers previously hypothesized to influence browning across 37 ALTM lakes at different time scales, followed by seasonal Mann–Kendall trend analyses of DOC, color, and specific UV absorbance at 254 nm (SUVA_254_)^31^ to classify the browning status of these lakes. Concurrently, we analyzed the spatiotemporal patterns of apparent quantum yields of singlet oxygen (Φ_app,^1^O_2__) for whole water samples collected from these lakes with respect to watershed hydrologic connectivity, surficial geology, and season. ^1^O_2_ was selected as the focus of our work for its critical role in biogeochemical cycling,^32–36^ transformations of organic micropollutants and bioactive secondary metabolites,^37–44^ and pathogen inactivation,^45–47^ among other processes, as well as its better-understood formation and deactivation mechanisms that facilitate the estimation of environmentally relevant concentrations for fate modeling.^48,49^ To this end, we compared depth-averaged steady-state concentrations of ^1^O_2_ in the euphotic zone of ALTM lakes based on their browning status and evaluated the half-lives of ^1^O_2_-mediated reactions for selected contaminants relative to lake hydraulic residence times.

## Materials and Methods

Chemical sources and reagent preparation are described in the Supporting Information.

### Field Sampling

Whole water samples were collected from 37 lakes (Figure S1) monitored through the ALTM program for water chemistry parameters (e.g., pH, DOC, dissolved inorganic carbon, specific conductance, acid neutralizing capacity, acid anions, base cations, and speciated aluminum).^29^ SUVA_254_ has also been monitored in these lakes since 2013.^50^ Of the lakes sampled, 35 are headwater or chain drainage lakes situated in watersheds with predominantly thin, medium, or thick deposits of glacial till, while the remaining two are mounded seepage lakes.^51^ Most lakes are surrounded by a mix of deciduous forest, coniferous forest, and deciduous-coniferous forest, with some also bordered by shrub-sapling habitats and wetlands, whereas open grasslands, agricultural fields, and developed areas are minimal.^29^ Overall, these lakes exhibit a range of morphometric characteristics and watershed attributes (Table S1). Whole water samples (*n* = 129) were collected over three seasons (Table S2): fall–winter (October–November 2022), spring–summer (May–June 2023), and summer–fall (September 2023) under dry weather conditions. Samples were taken (e.g., using a Kemmerer sampler) at a depth of 0.5 m from the deepest part of each lake (37 samples per season), and in six cases, paired samples were also grabbed from outlet streams of a subset of drainage lakes (6 samples per season) for comparison. Samples were shipped in coolers to Syracuse University as soon as practically possible, filtered through 0.2-μm poly­(ether sulfone) membranes, and stored under 4 °C until analysis. Field blanks (*n* = 9) were collected and processed with each batch of samples.

### Sample Analysis

Water chemistry parameters were analyzed by the U.S. Geological Survey Soil and Low-Ionic-Strength Water Quality Laboratory (Troy, NY). Optical properties, such as Napierian absorption coefficient at 440 nm (*a*_440_),^52^ SUVA_254_,^31^ *E2:E3* (the ratio of Napierian absorption coefficients at 250 and 365 nm),^53^ spectral slope coefficients (e.g., *S*_290–400_ and *S*_300–600_),^54,55^ fluorescence index (FI),^56^ humification index (HIX),^57^ freshness index (*β*:*α*),^58^ as well as total dissolved iron ([Fe]), were measured at Syracuse University. Water chemistry and optical data are summarized in Tables S3 and S4.

### Photochemistry Experiments

Photolysis experiments were performed at least in duplicate using an Atlas Suntest XLS+(II) solar simulator equipped with a 1700 W xenon arc lamp and a daylight glass 300 nm UV filter. The lamp irradiance was controlled at 320 W/m^2^ between 300 and 800 nm, and the solar simulator chamber temperature was maintained at 25 ± 1 °C with an Atlas SunCool chiller. Prior to irradiation, filtered samples were standardized to a DOC of 4.0 mg C/L when applicable (samples with DOC below this threshold were analyzed at their ambient DOC)^59^ and spiked with furfuryl alcohol (FFA) as a probe to measure ^1^O_2_ production,^60,61^ along with 0.1 mM of methanol to quench hydroxyl radicals (^•^OH).^62^ Samples were then transferred to quartz test tubes (100 mm × 11 mm i.d.; held at ∼30° from the horizontal) and irradiated inside the solar simulator with bimolecular *p*-nitroanisole/pyridine actinometer solutions (to monitor the incident light intensity^63,64^ ). Solutions of Suwannee River natural organic matter (SRNOM; 2R101N), Suwannee River fulvic acid (SRFA; 3S101F), and Suwannee River humic acid (SRHA; 3S101H) were also irradiated as reference samples.

Φ_app,^1^O_2__ were calculated over the wavelength range of 290–550 nm and combined with daily average solar irradiance to estimate the depth-averaged steady-state concentrations of ^1^O_2_ in the euphotic zone ([^1^O_2_]_ss,daily average_^euphotic zone^) for each lake on the corresponding sampling dates using eq 1:^48,65,66^

1 [O12]ss,daily averageeuphotic zone=Φapp,O12kdΔ×CF×∑λ=290nm550nmZλ,daily averagezeuphotic zone×(1−e−Kd,λzeuphotic zone)×(1−fbackscatter)×fabs,CDOM

where Φ_app,^1^O_2__ (mol mol-photons^–1^) is the apparent quantum yield of ^1^O_2_, *k*_d_^Δ^ (s^–1^) is the pseudo-first-order deactivation rate constant of ^1^O_2_ by water,^61^ CF is the nonclear-sky correction factor for solar irradiance in the Adirondack region (i.e., 0.60 ± 0.06),^66^ *Z*_λ,daily average_ (10^–3^ mol-photons cm^–2^ s^–1^ nm^–1^) is the site-specific daily average solar irradiance at a given wavelength λ modeled using the *Simple Model of the Atmospheric Radiative Transfer of Sunshine (SMARTS) v2.9.9* (Table S5)^67–69^ with adjustments made for reflection off the water surface and increased path length within the water column,^63,65,70^ *z*_euphotic zone_ (cm) is the euphotic zone depth estimated using an empirical relationship established for Adirondack lakes (i.e., *z*_euphotic zone_ = 4.6/[0.15­[DOC]^1.08^] × 100),^71^ *K*_d,λ_ (cm^–1^) is the diffuse attenuation coefficient modeled following the relationship developed using UV wavelengths (i.e., *K*_d,λ_ = exp­(−0.01347λ + 5.36­[DOC]^0.157^)),^72^ *f*_backscatter_ is the fraction of sunlight backscattered out of the water column,^48^ and *f*_abs,CDOM_ is the fraction of lake water absorbance attributable to colored DOM (CDOM).^48^ Complete details of the photochemistry experiments and related calculations are provided in Sections S6–S8.

### Data Analysis

Long-term water chemistry data (June 1992 to September 2023) for the 37 ALTM lakes were retrieved from the U.S. Geological Survey (USGS) National Water Information System^50^ and imported into *Colab Pro* (Google) for analysis using *Python* or *R*. To investigate synchrony between the time series of DOC and four external drivers (preselected from a larger set of variables based on a variance inflation factor threshold of 2.0 to minimize collinearity^73^), including summed wet deposition of sulfate and nitrate, precipitation, soil wetness, and solar irradiance, across all lakes, wavelet coherence tests were performed using the *wsyn* package^74^ over short (3–6 months), intermediate (12–24 months), and long (36–72 months) time scale bands (Table S6). Monthly wet deposition data for sulfate and nitrate were averaged from four Adirondack monitoring sites within the National Atmospheric Deposition Program’s National Trends Network.^75^ Monthly total precipitation data were retrieved from the Parameter-elevation Regressions on Independent Slopes Model (PRISM) Climate Group^76^ using the *prism* package.^77^ Monthly all-sky surface shortwave downward irradiance and root zone soil wetness data were retrieved from the National Aeronautics and Space Administration’s Langley Research Center Prediction of Worldwide Energy Resource (POWER) Project^78^ using the *nasapower* package.^79,80^ Each time series was detrended, Box-Cox transformed, and standardized before analysis.^81^ For every time scale band, the strength of association between two time series was quantified using a magnitude metric (ranging from 0 to 1), with a phase metric (ranging from −π to π) further derived to characterize the relationship as positive in-phase (−π/4 to π/4), lagged positive (−3π/4 to −π/4), lagged negative (π/4 to 3π/4), or negative antiphase (3π/4 to π or −π to −3π/4).^81–83^ Wavelet linear modeling was then applied to quantify the proportion of synchrony explained by time scale-specific relationships between DOC and statistically significant drivers and to assess their independent contributions and potential interactions (Table S7).^81^

To evaluate directional trends and rates of change in DOC and other water chemistry parameters (e.g., color and SUVA_254_), the nonparametric seasonal Mann–Kendall test^84^ was performed using the *pyMannKendall* package^85^ to calculate Sen’s slopes for the time series of each lake (based on the full extent of data available).^86^ Sen’s slopes with *p* < 0.05 were interpreted as statistically significant indicators of either increasing or decreasing trends, whereas those with *p* ≥ 0.05 were considered insignificant and replaced with zeros (Table S8).^87^ Sen’s slopes were further standardized for k-means clustering to classify the browning status of ALTM lakes.

Statistical analyses (e.g., the Kruskal–Wallis test, Mann–Whitney *U* test, Spearman’s correlation analysis, and generalized additive modeling) were performed using the *stats* ^88^ and *mgcv* ^89^ packages.

## Results and Discussion

### Mechanisms Regulating DOC Dynamics

To test the hypothesis that browning in ALTM lakes responds to temporal changes in DOM, wavelet coherence tests were first performed to evaluate the synchrony between the time series of color and DOC as a function of time scale. Coherences between color and DOC were significant (band-aggregated *p* < 0.0001–0.006) and positive in-phase (mean phase 0.013–0.160) with comparable magnitudes (0.56 ± 0.02–0.64 ± 0.07) across three time scale bands (Figure 1a), confirming the temporally persistent nature of their synchrony. On average, a 0.095 ± 0.038 mg C/L change in DOC corresponded to a one-unit shift in color on the platinum–cobalt scale for ALTM lakes, which compared well with the value of 0.098 ± 0.043 mg C/L per platinum–cobalt unit derived from a large-scale survey of Adirondack lakes that preceded the ALTM program.^30^

**Figure 1:**
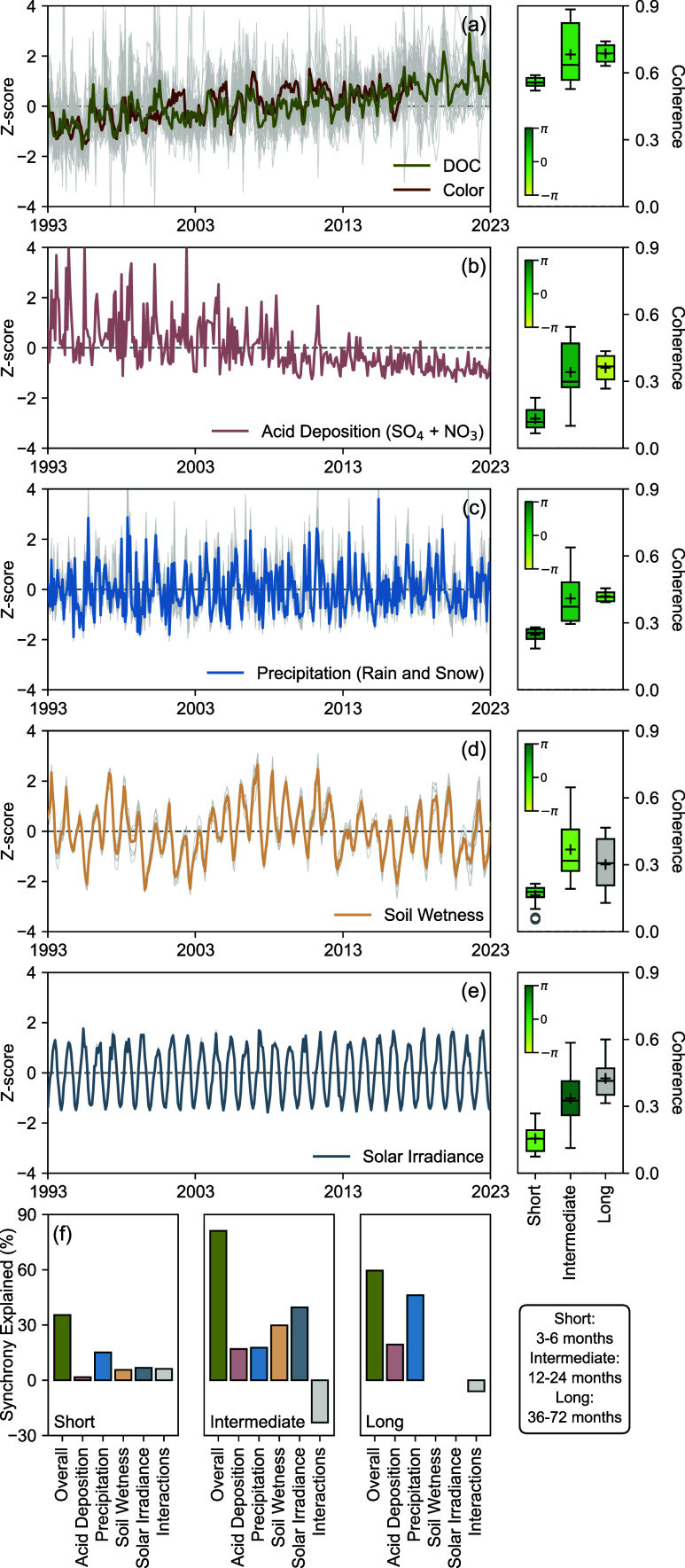
Wavelet coherences between the time series of DOC and regional drivers across 37 ALTM lakes over short (3–6 months), intermediate (12–24 months), and long (36–72 months) time scale bands: (a) Z-score-standardized time series of DOC and color for ALTM lakes along with coherence magnitudes and phase relationships for three time scales (Figure S5). Note that color data collection discontinued after 2017. (b) Z-score-standardized time series of the summed sulfate (SO_4_) and nitrate (NO_3_) wet deposition for ALTM lakes along with coherence magnitudes and phase relationships for three time scales (Figure S6). (c) Z-score-standardized time series of precipitation for ALTM lakes along with coherence magnitudes and phase relationships for three time scales (Figure S7). (d) Z-score-standardized time series of root zone soil wetness for ALTM lakes along with coherence magnitudes and phase relationships for three time scales (Figure S8). (e) Z-score-standardized time series of solar irradiance for ALTM lakes along with coherence magnitudes and phase relationships for three time scales (Figure S9). On panels (a)–(e), the solid line represents the site-average Z-score time series, while gray lines represent site-specific time series when available. Coherence magnitudes are normalized between 0 and 1, and phase relationships are classified as follows: positive in-phase (−π/4 to π/4), lagged positive (−3π/4 to −π/4), lagged negative (π/4 to 3π/4), and negative antiphase (3π/4 to π or −π to −3π/4).^83^ On panels (d) and (e), the gray box in the boxplot indicates statistically insignificant coherence. (f) Synchrony explained by statistically significant drivers across the three time scales based on wavelet linear modeling (Table S7). Gray bars represent interacting effects among drivers.

To probe mechanisms influencing DOC trends, wavelet coherence tests were further performed to characterize time scale-specific synchrony between DOC and four external drivers representing regional atmospheric and hydroclimatic conditions, including acid deposition (i.e., the summed wet deposition of sulfate and nitrate), precipitation, soil wetness, and solar irradiance (Figure 1b–e). Wavelet linear modeling was then applied with DOC as the response variable to quantify the proportion of synchrony attributable to its relationships with these drivers (Figure 1f).^81^ Over short time scales, all four drivers were statistically significant contributors, but only precipitation accounted for >10% of synchrony. Consistent with earlier findings,^81,90^ precipitation showed a lagged negative relationship with DOC (mean phase 1.91), likely reflecting the joint influence of dilution (e.g., event-driven inputs of low-DOM upland runoff that bypass organic-rich soil horizons) and flushing (e.g., reduced hydraulic residence times that limit the retention and accumulation of DOM) within lakes.^91–93^ Over intermediate time scales, the four drivers collectively explained 81% of synchrony, with solar irradiance contributing the largest fraction (39%), followed by soil wetness (30%), precipitation (18%), and acid deposition (17%). Solar irradiance exhibited a negative antiphase relationship with DOC (mean phase 3.06), underscoring the importance of in-lake photodegradation as a sink for DOM (e.g., through mineralization to carbon dioxide).^94,95^ Meanwhile, soil wetness showed a lagged positive relationship with DOC (mean phase −1.31), as expected for increased DOM production and leaching driven by microbial activity under redox oscillations associated with drying–rewetting cycles.^96^ Precipitation and acid deposition both exhibited a lagged negative relationship with DOC (mean phase 1.35), supporting the notion that declines in atmospheric acid deposition alter the acidity of soils and/or the ionic strength of soil solutions,^1^ thereby enhancing DOM solubility and mobility. For instance, deprotonation of more acidic functional groups increases the net negative surface charge of DOM as soils become less acidic, while lower ionic strength concurrently reduces cation bridging and aggregation of DOM.^97,98^ Over long time scales, precipitation and acid deposition were the only statistically significant drivers that explained 60% of synchrony. Contrary to the phase relationships observed at shorter time scales, precipitation showed a lagged positive relationship with DOC (mean phase −0.87) as previously noted,^6,81^ suggesting that under extended precipitation regimes, enhanced hydrologic connectivity across the terrestrial–aquatic interface may promote DOM export to lakes by activating subsurface flow paths (e.g., those intersecting organic-rich soil horizons^99^) and expanding source areas (e.g., those otherwise hydrologically disconnected during prolonged dry periods^100^).^101^ Together, these results provide insights into the time-scale-specific and time-lagged processes driving DOC dynamics in ALTM lakes that cannot be readily resolved by standard correlation metrics.

### Classification of Lake Browning Status

To classify the browning status of ALTM lakes, Sen’s slopes for the time series of DOC, color, and SUVA_254_ were calculated to determine the direction and magnitude of temporal changes. Most lakes (i.e., 33 out of 37) showed statistically significant positive slopes for DOC (0.005–0.105 mg C/L-year; *p* < 0.0001–0.0042; Figure S11), with a median value of 0.057 mg C/L-year similar to those recorded for lakes in eastern Canada (e.g., 0.05 mg C/L-year)^14,102^ and over 400 headwater lakes and streams in Europe and North America (e.g., 0.04 mg C/L-year).^2^ Sen’s slopes for DOC were positively correlated with those for pH (Spearman’s ρ = 0.481; *p* < 0.0001) but negatively correlated with those for the summed sulfate and nitrate concentrations (ρ = −0.607; *p* < 0.0001), reinforcing the link between recovery from acidification in ALTM lakes and their long-term DOC trends. Color also increased in 90% of the lakes with a median slope of 0.027 m^–1^/year (converted to Napierian absorption coefficient at 440 nm^52^), which was equivalent to a 2.2% annual increase that exceeded rates (e.g., 1.1–1.6%/year) estimated for browning lakes in southern Sweden^11^ and eastern Canada.^14^ Cross-regional comparisons were intended to contextualize the rates of temporal change in DOC and color for ALTM lakes within the broader spectrum of values reported in the literature rather than to infer shared underlying mechanisms given the environmental heterogeneity among systems. Somewhat contrary to the trends of DOC and color, SUVA_254_ either declined or remained mostly stable over the past decade. Multiple studies, such as those on acid-impacted streams in upland catchments in Scotland,^103^ temperate lakes at the North Temperate Lakes Long-Term Ecological Research site in northern Wisconsin,^104^ and boreal lakes on the Boreal Plains of western Canada,^105^ have also observed divergent trends between DOC and SUVA_254_, which may stem from their differential responses to shifting source contributions (e.g., disproportionate mobilization of low-aromatic DOM from mineral soil horizons^106,107^ ) and/or changing processing mechanisms (e.g., preferential loss of aromatic DOM moieties via in-lake decomposition and flocculation^108^). Varying degrees of temporal (de)­coupling among Sen’s slopes for DOC, color, and SUVA_254_ suggest that, while biogeochemically linked, these metrics do not necessarily serve as proxies for one another.

To provide a framework for interpreting ^1^O_2_ formation in the context of browning, k-means clustering of Sen’s slopes was performed to classify ALTM lakes into three clusters (Figure 2a). Overall, the slopes for DOC and color were more positive in clusters B and C than in cluster A lakes, whereas the slopes for SUVA_254_ were less negative in clusters A and B than in cluster C lakes (Figure 2b–d). Color for ALTM samples analyzed in this work showed a stronger positive correlation with its Sen’s slopes than DOC did with its corresponding slopes, whereas SUVA_254_ was negatively correlated with its slopes (Figure 2e–g). Since lakes with faster increases in color and DOC but slower declines in SUVA_254_ were more likely to feature darker water color, higher DOC, and more aromatic DOM, lakes in clusters A, B, and C were operationally designated as experiencing mild, moderate, and intense browning, respectively.

**Figure 2:**
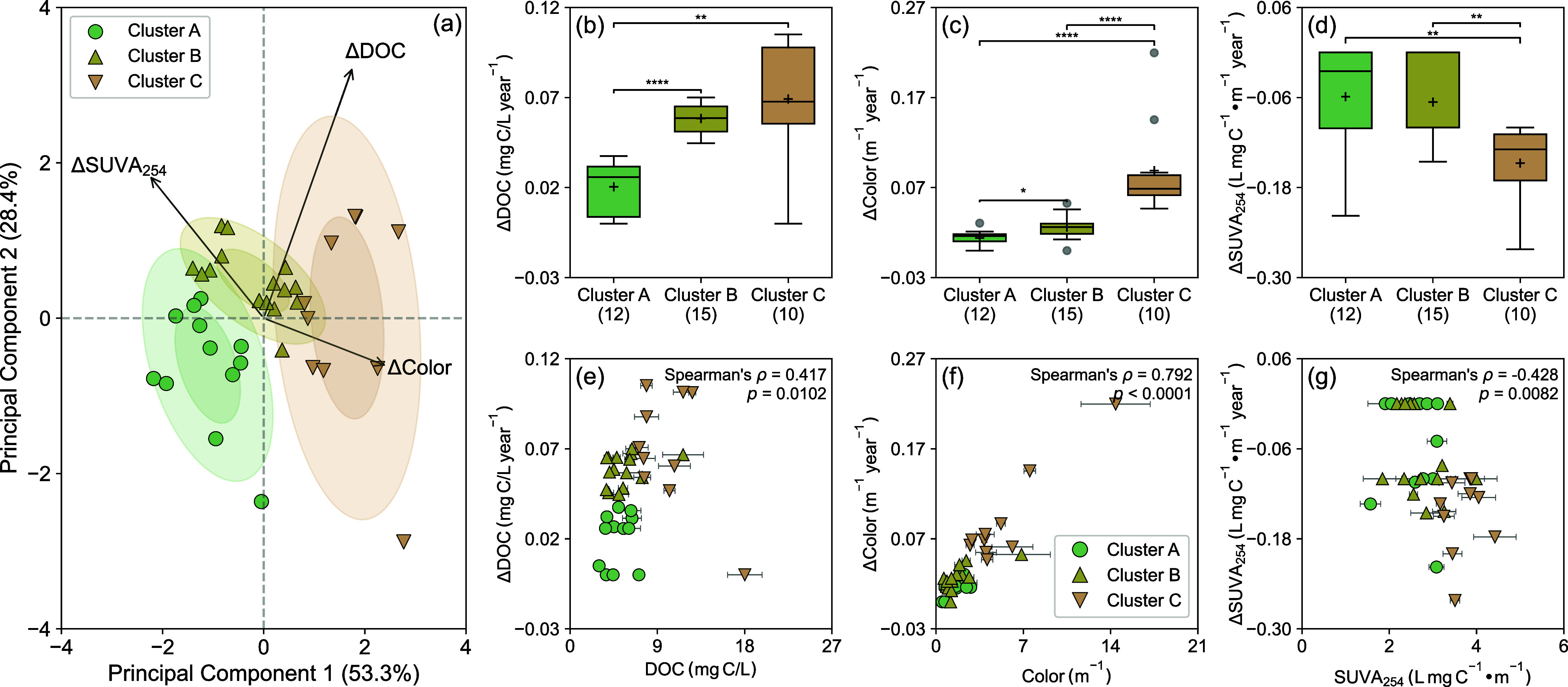
Classification of ALTM lakes by Sen’s slopes of the time series of DOC, color, and SUVA_254_ for multiple comparisons and Spearman’s correlation analysis: (a) K-means clustering of lakes based on their Sen’s slopes for DOC, color, and SUVA_254_ (Table S8). Ellipses represent bivariate confidence intervals derived from the covariance structure of the data. The inner ellipse corresponds to the 68% confidence interval (one standard deviation), while the outer ellipse represents the 95% confidence interval (two standard deviations). (b) Multiple comparisons of Sen’s slopes for DOC (data available from 1992 to 2023) across lake clusters. (c) Multiple comparisons of Sen’s slopes for color (data available through 2017) across lake clusters. (d) Multiple comparisons of Sen’s slopes for SUVA_254_ (data available from 2013 onward) across lake clusters. (e) Spearman’s correlation between Sen’s slopes for DOC and DOC measured for ALTM samples. (f) Spearman’s correlation between Sen’s slopes for color and color (based on *a*_440_) measured for ALTM samples. (g) Spearman’s correlation between Sen’s slopes for SUVA_254_ and SUVA_254_ measured for ALTM samples. On panels (b)–(d), each box spans the 25th to 75th percentiles, with whiskers extending to 1.5 times the interquartile range below the 25th and above the 75th percentiles. The centerline and “+” sign mark the median and mean, respectively. Gray circles represent outliers. Numbers in parentheses represent the number of lakes in each cluster. For multiple comparisons, a Kruskal–Wallis test was first performed to determine whether statistically significant differences existed among groups. If significant, pairwise Mann–Whitney *U* tests were performed, with significant differences marked by asterisks as “*” (*p* < 0.05), “**” (*p* < 0.01), “***” (*p* < 0.001), or “****” (*p* < 0.0001). On panels (e)–(g), error bars represent the standard deviations of measurements for samples from three sampling seasons; where absent, bars fall within symbols.

### Spatiotemporal Patterns of Φ_app,^1^O_2__

Φ_app,^1^O_2__ for ALTM samples (*n* = 129) ranged from 0.6 to 3.7 × 10^–2^ mol mol-photons^–1^ (median 2.2 × 10^–2^; Table S9) and fell within the range (0.5–10.9 × 10^–2^)^55,59,109–112^ reported for whole water samples from temperate lacustrine systems in North America (Figure S12). To better contextualize the photoreactivity of ALTM lake waters, Φ_app,^1^O_2__ were normalized against Φ_app,^1^O_2__^SRNOM^ measured in parallel as recommended by Ossola et al.^49^ and aggregated for comparison with literature data on native or treated water samples across the freshwater-marine continuum, as well as DOM isolates, extracts, or fractions from diverse aquatic and terrestrial sources, compiled from 99 references (Table S18). Thirty-one of these references comeasured polychromatic and/or wavelength-specific Φ_app,^1^O_2__^SRNOM^ to allow for the harmonization of variations in Φ_app,^1^O_2__. SRNOM was selected for normalization because it is more commonly analyzed than other reference materials in aquatic photochemistry studies despite differences in composition and photoreactivity relative to DOM in ALTM lakes. Overall, Φ_app,^1^O_2__/Φ_app,^1^O_2__^SRNOM^ derived from the literature spanned over two orders of magnitude (0.06–33; median 1.05; *n* = 1068) due to heterogeneity in DOM origins, variability in sample treatment conditions, and uncertainties in the wavelength dependence of Φ_app,^1^O_2__.^49^ Φ_app,^1^O_2__/Φ_app,^1^O_2__^SRNOM^ for ALTM samples ranged from 0.29 to 1.82 (median 1.12; Figure S13), which were not significantly different from ratios for whole water samples from other lacustrine environments (0.33–3.10; median 1.12; e.g., reservoirs in Japan^113^) and Nordic Reservoir NOM (0.47–1.93; median 1.12) but were lower than those for Pony Lake Fulvic Acid (0.22–5.51; median 1.57) and several other lacustrine DOM isolates or fractions from diverse geographic areas (0.13–3.74; median 1.55; e.g., U.S.,^114,115^ Europe,^116^ and polar regions^114,115^ ). Compared to other aquatic matrices, Φ_app,^1^O_2__/Φ_app,^1^O_2__^SRNOM^ for ALTM samples were in the same range as ratios observed for riverine samples (0.06–3.96; median 1.13) but were lower than those for estuarine (0.89–3.92; median 2.10; e.g., the Florida Everglades^115,117^ ) and marine samples (0.47–3.71; median 1.37; e.g., coastal seawaters in South China^118^). Higher Φ_app,^1^O_2__/Φ_app,^1^O_2__^SRNOM^ in these matrices may partly reflect the enhancing effects of halides on FFA reactivity^61^ and/or ^1^O_2_ production^119^ relative to ALTM samples with low specific conductance (14.2 ± 4.6 μS/cm; Table S3). Compared to DOM of other origins, Φ_app,^1^O_2__/Φ_app,^1^O_2__^SRNOM^ for ALTM samples were significantly lower than ratios for wastewater samples (0.62–4.97; median 2.26; e.g., effluent organic matter^120^) but showed no statistical difference from those for algal (0.25–3.10; median 1.13), soil (0.48–6.09; median 1.13), or pyrogenic organic matter (0.15–3.04; median 1.27). With normalization to Φ_app,^1^O_2__^SRNOM^, these semiqualitative comparisons position Φ_app,^1^O_2__ for ALTM samples within the broader literature, though our Φ_app,^1^O_2__ only represent solar-integrated values^48^ and bulk aqueous-phase measurements (i.e., that do not account for the microheterogeneous distribution of ^1^O_2_ ^121,122^ ).

Spatial heterogeneity in Φ_app,^1^O_2__ was evident among lakes with different watershed hydrologic connectivity (Figure 3a) and surficial geology (Figure 3b). Φ_app,^1^O_2__ for headwater and chain drainage lakes (1.5–3.6 × 10^–2^; median 2.0 × 10^–2^ and 1.2–3.7 × 10^–2^; median 2.5 × 10^–2^) were higher than those for mounded seepage lakes (0.6–0.9 × 10^–2^; median 0.7 × 10^–2^), which is attributable to (1) enhanced ^1^O_2_ formation efficiency resulting from DOM with a greater proportion of autochthonous components and smaller molecular sizes in drainage lakes and (2) reduced ^1^O_2_ formation efficiency associated with elevated [Fe] in seepage lakes. For example, *E2:E3* (4.14–8.50; median 5.53; Figure S15) and FI (1.36–1.61; median 1.46; Figure S16) of samples from both types of drainage lakes, which receive most of their water from upland runoff and tributary streams, were higher than those of samples from seepage lakes (4.14–5.14; median 4.59 and 1.28–1.38; median 1.34, respectively) that are sustained mainly by precipitation with minimal upland or groundwater inputs.^51^ On the other hand, [Fe] in samples from seepage lakes (13.5–16.7 μM; median 15.6 μM; Figure S17) far exceeded those in samples from drainage lakes (0.3–13.1 μM; median 1.9 μM) and likely exerted a stronger quenching effect on excited states of DOM (e.g., through static and dynamic quenching by bound paramagnetic Fe^3+^) as demonstrated in titration experiments with humic and fulvic acids or effluent organic matter.^123,124^ Φ_app,^1^O_2__ for samples taken from midlake and outlet stream locations at six drainage lakes were not statistically different, possibly due to the relatively short hydraulic residence times of these systems (0.21 ± 0.15 years; Table S1). When further categorized by glacial till depth, Φ_app,^1^O_2__ for thin till drainage lakes (1.6–3.7 × 10^–2^; median 2.5 × 10^–2^) were higher than for both medium till (1.2–3.1 × 10^–2^; median 2.1 × 10^–2^) and thick till (1.8–2.2 × 10^–2^; median 2.0 × 10^–2^) drainage lakes. Such differences in Φ_app,^1^O_2__ may reflect variation in ^1^O_2_ formation efficiency from DOM shaped by a dynamic balance between hydrologic inputs from upland runoff with shorter transit times (e.g., delivering DOM that has undergone photochemical and microbial alteration along the terrestrial–aquatic continuum^125,126^ ) and groundwater inflow with longer residence times (e.g., supplying DOM that is more photodegradable and aerobically biolabile^127^) into lakes underlain by glacial tills of varying depth.^128^

**Figure 3:**
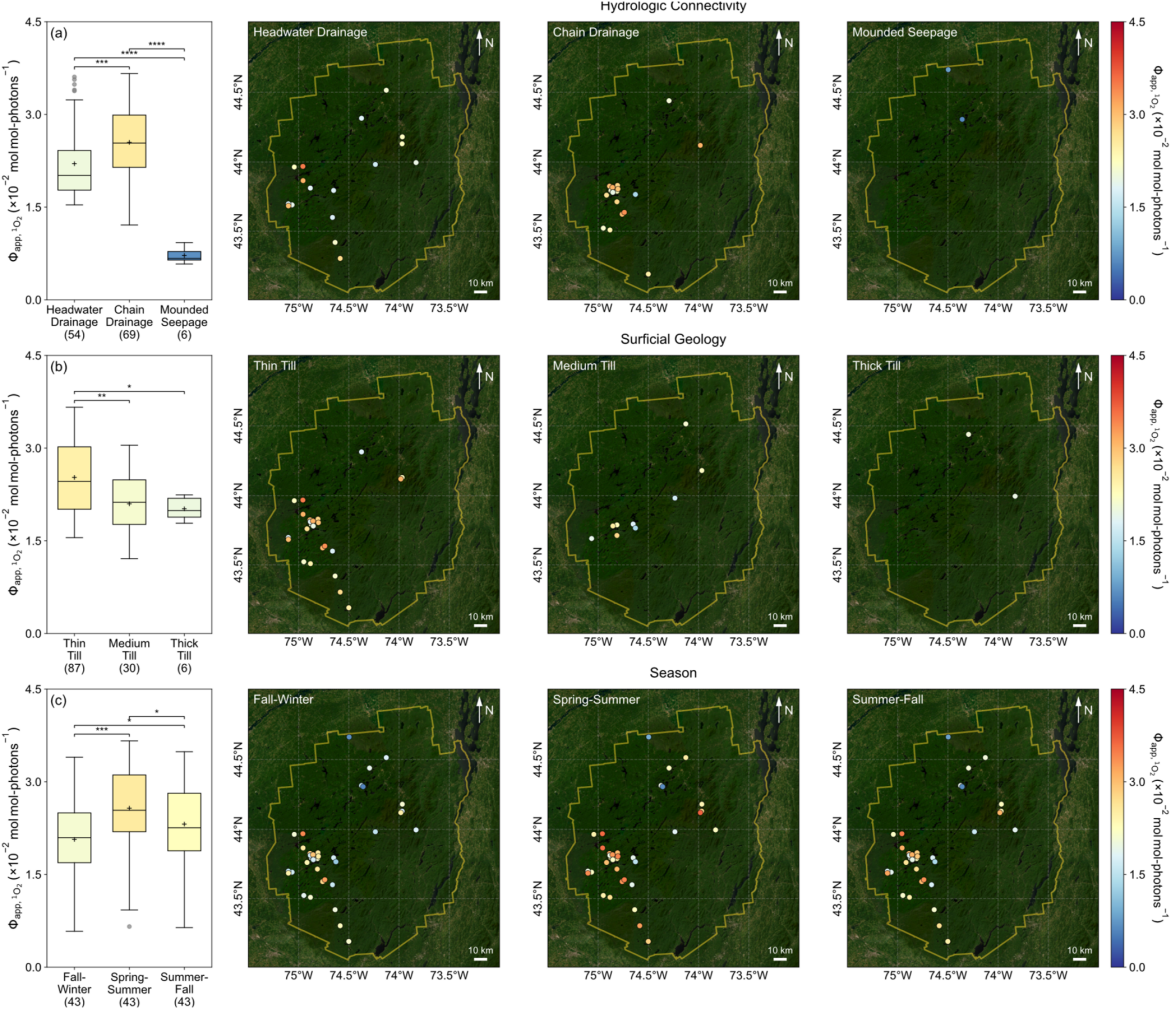
Spatiotemporal patterns of Φ_app,^1^O_2__ for ALTM lakes grouped by (a) hydrologic connectivity (headwater drainage, chain drainage, or mounded seepage), (b) surficial geology (thin till, medium till, or thick till), and (c) season (fall–winter, spring–summer, or summer–fall). On panels (a)–(c), each box spans the 25th to 75th percentiles, with whiskers extending to 1.5 times the interquartile range below the 25th and above the 75th percentiles. The centerline and “+” sign mark the median and mean, respectively. Gray circles represent outliers. Numbers in parentheses represent the number of samples in each group. Box colors correspond to their respective median values referenced against the color bar. For multiple comparisons, a Kruskal–Wallis test was first performed to determine whether statistically significant differences existed among groups. If significant, pairwise Mann–Whitney *U* tests were performed, with significant differences marked by asterisks as “*” (*p* < 0.05), “**” (*p* < 0.01), “***” (*p* < 0.001), or “****” (*p* < 0.0001). On each map, the solid yellow line delineates the boundary of Adirondack Park.

Seasonal variations in Φ_app,^1^O_2__ for ALTM samples followed the pattern of spring–summer > summer–fall > fall–winter (Figure 3c), presumably driven by shifts in the relative contributions of DOM from allochthonous and autochthonous sources (e.g., increased terrestrial inputs during wet periods and elevated phytoplankton production in warmer months), as well as changes in the rates and extent of watershed-scale and in-lake processing of DOM (e.g., enhanced photooxidation under stronger solar irradiance and accelerated microbial reworking at higher temperatures).^108,125^ However, previous studies have documented inconsistent seasonal patterns of Φ_app,^1^O_2__ in other surface water systems (e.g., Scandinavian lakes and streams,^116^ Lake Superior and its tributaries,^55^ and Prairie Pothole wetlands;^109^ Figure S20). Throughout the sampling period, the slope of the linear regression between Φ_app,^1^O_2__ and *E2:E3* increased from 0.24 ± 0.08 for fall–winter to 0.57 ± 0.19 for spring–summer and then decreased to 0.28 ± 0.11 for summer–fall (Figure S21). Seasonal slopes for ALTM samples overlapped with those measured for lakes and wetlands in Wisconsin (0.38 ± 0.15; Table S10),^59,110,112^ Minnesota (0.22 ± 0.03),^111^ and the Prairie Pothole Region (0.62 ± 0.04),^109^ as well as with that for Lake Superior (0.19 ± 0.03).^55^ Consolidating samples from three seasons into one single data set yielded a slope of 0.21 ± 0.07 and an intercept of 1.1 ± 0.4, which closely matched the global slope (0.26 ± 0.02) and intercept (1.3 ± 0.1) derived from a broader set (*n* = 1547) of Φ_app,^1^O_2__ (0.05–18 × 10^–2^; median 2.3 × 10^–2^) and *E2:E3* (0.4–29.9; median 5.7). Such convergence should be interpreted with caution, as it does not necessarily imply a generalizable relationship given the substantial variability in slopes and intercepts reported across studies (Table S10).^49,113,129^ Though *E2:E3* has long been proposed as a predictor of Φ_app,^1^O_2__,^114^ generalized additive modeling (which uses penalized regression splines to model both linear and nonlinear relationships among multiple factors^130^) identified *S*_290–400_ and FI as linear predictors and [Fe] as a nonlinear predictor of Φ_app,^1^O_2__ for ALTM samples across seasons (Figures S24–S26). Slopes from the linear regression between Φ_app,^1^O_2__ and *S*_290–400_ followed a similar pattern to those of *E2:E3*, with the steepest slope observed during spring–summer (Figure S22). Spectral slope coefficients such as *S*_290–400_ ^131,132^ and *S*_300–600_ ^55,113,117^ are indicators of DOM processing and have therefore been explored as alternative single predictors of Φ_app,^1^O_2__ to *E2:E3*. Slopes from the linear regression between Φ_app,^1^O_2__ and FI, in contrast, remained relatively stable (6.6 ± 1.4 for fall–winter, 6.7 ± 1.5 for spring–summer, and 6.8 ± 1.3 for summer–fall; Figure S23). Complementary to *S*_290–400_ and FI, [Fe] exerted a nonlinear negative influence on Φ_app,^1^O_2__, which qualitatively aligned with the bivariate patterns identified by Spearman’s correlation analysis (Figure S27). Taken together, these analyses highlight the interplay between hydrogeological conditions and DOM quality in shaping the spatiotemporal patterns of Φ_app,^1^O_2__ for ALTM lakes.

### ^1^O_2_ Production in Relation to Lake Browning

Overall, Φ_app,^1^O_2__ for samples from ALTM lakes experiencing mild (i.e., cluster A) or intense browning (i.e., cluster C) were lower than those for samples from lakes undergoing moderate browning (i.e., cluster B; Figure 4a). Cluster A samples were characterized by the highest pH (which favors deactivation of excited states of DOM based on the charge-transfer model^133^) and the lowest FI (suggesting limited contribution from autochthonous DOM^56^), along with comparatively high E2:E3 (which reduces the probability of charge-transfer interactions^114^) and low [Fe] (indicating weak quenching of excited states of DOM by Fe^3+^;^124^ Figures S28 and S29). Cluster C samples exhibited lower pH and *E2:E3* but higher FI and [Fe] than cluster A samples; however, Φ_app,^1^O_2__ for samples from these two clusters of lakes showed no significant difference. Cluster B samples struck a balance among these factors, with moderate pH (lower than cluster A but higher than cluster C), high *E2:E3* (similar to cluster A but higher than cluster C), high FI (similar to cluster C but higher than cluster A), and low [Fe] (similar to cluster A but lower than cluster C), again illustrating the multifaceted and sometimes contrasting effects of the water chemistry and DOM composition on Φ_app,^1^O_2__.

**Figure 4:**
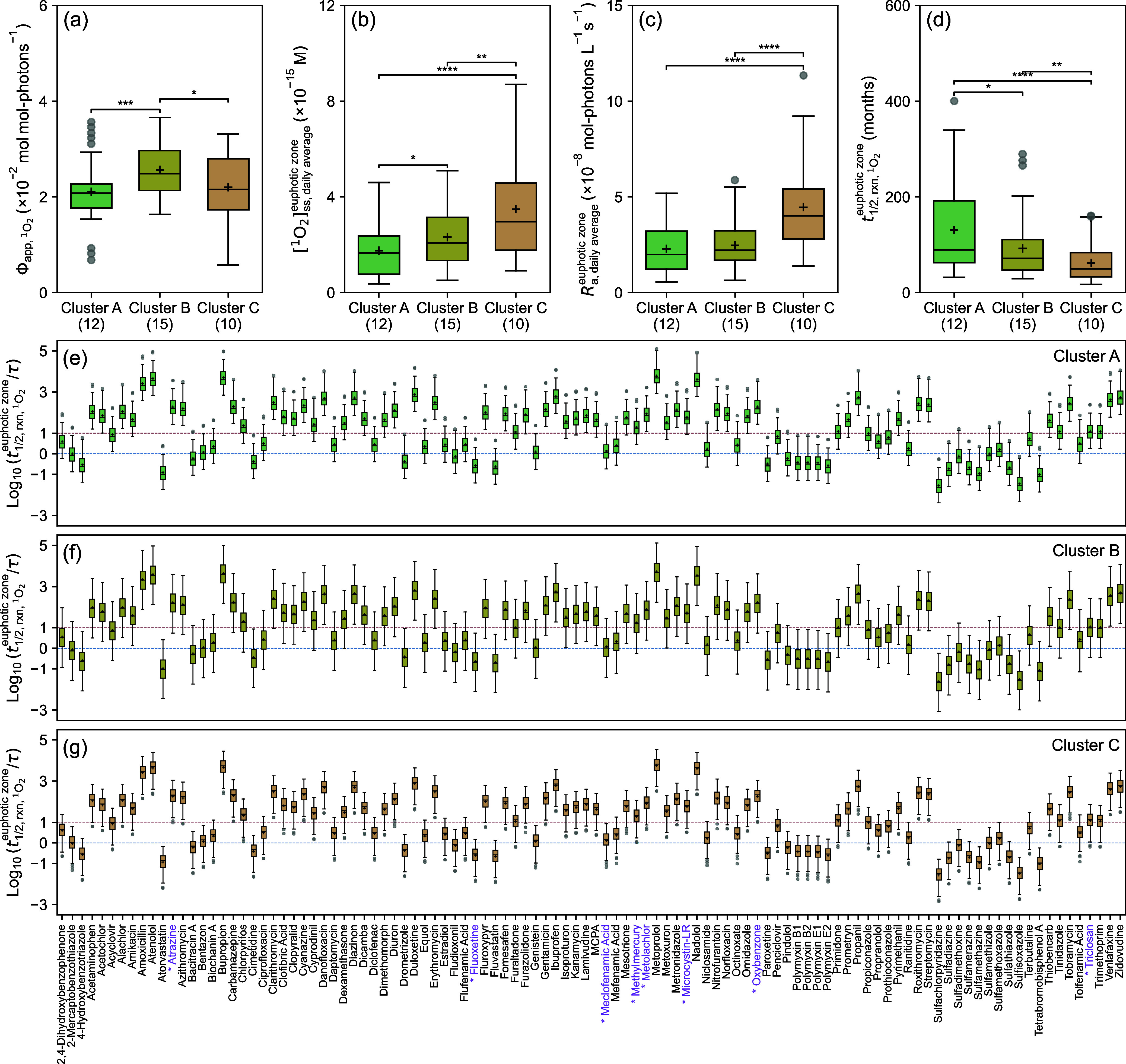
Comparisons of [^1^O_2_]_ss, daily average_^euphotic zone^ and the log_10_-transformed ratios of contaminant half-lives due to reaction with ^1^O_2_ in the euphotic zone (*t*_1/2, rxn, ^1^O_2__^euphotic zone^) to the hydraulic residence times (τ) of ALTM lakes with varying browning status: (a) Multiple comparisons of Φ_app,^1^O_2__ across lake clusters. (b) Multiple comparisons of [^1^O_2_]_ss, daily average_^euphotic zone^ across lake clusters. (c) Multiple comparisons of *R*_a, daily average_^euphotic zone^ across lake clusters. (d) Multiple comparisons of *t*_1/2, rxn, ^1^O_2__^euphotic zone^ (estimated based on a median *k*_rxn, ^1^O_2__ of 1.8 × 10^6^ M^–1^ s^–1^ for 106 compounds; Table S16) across lake clusters. (e) Cluster-specific distribution of log_10_-transformed *t*_1/2, rxn, ^1^O_2__^euphotic zone^/τ ratios for 106 compounds in cluster A lakes. (f) Cluster-specific distribution of log_10_-transformed *t*_1/2, rxn, ^1^O_2__^euphotic zone^/τ ratios for 106 compounds in cluster B lakes. (g) Cluster-specific distribution of log_10_-transformed *t*_1/2, rxn, ^1^O_2__^euphotic zone^/τ ratios for 106 compounds in cluster C lakes. Lakes in clusters A, B, and C were operationally designated as experiencing mild, moderate, and intense browning, respectively. On panels (a)–(d), each box spans the 25th to 75th percentiles, with whiskers extending to 1.5 times the interquartile range below the 25th and above the 75th percentiles. The centerline and “+” sign mark the median and mean, respectively. Gray circles represent outliers. Numbers in parentheses represent the number of lakes in each cluster. For multiple comparisons, a Kruskal–Wallis test was first performed to determine whether statistically significant differences existed among groups. If significant, pairwise Mann–Whitney *U* tests were performed, with significant differences marked by asterisks as “*” (*p* < 0.05), “**” (*p* < 0.01), “***” (*p* < 0.001), or “****” (*p* < 0.0001). On panels (e) to (g), each box spans the 25th to 75th percentiles, with whiskers extending to 1.5 times the interquartile range below the 25th and above the 75th percentiles. The centerline and “+” sign mark the median and mean, respectively. Gray circles represent outliers. The blue dashed line marks a *t*_1/2, rxn, ^1^O_2__^euphotic zone^/τ ratio of 1, whereas the red dashed line marks a *t*_1/2, rxn, ^1^O_2__^euphotic zone^/τ ratio of 10. Compounds (*n* = 8) highlighted in purple and marked with an asterisk denote those previously detected in Adirondack lake waters.^134–136^

To estimate steady-state concentrations of ^1^O_2_ in ALTM lakes, depth-averaged values such as [^1^O_2_]_ss, daily average_^euphotic zone^ and [^1^O_2_]_ss, daily average_^epilimnion^ were calculated in light of their relevance for photochemical modeling.^48^ [^1^O_2_]_ss, daily average_^euphotic zone^ ranged from 3.6 × 10^–16^ to 9.3 × 10^–15^ M (Table S13), whereas [^1^O_2_]_ss, daily average_^epilimnion^ ranged from 5.7 × 10^–16^ to 1.7 × 10^–14^ M (Table S14), which fell on the upper end of the depth-averaged concentrations predicted for lake epilimnia globally (e.g., 6 × 10^–17^–5 × 10^–15^ M).^48^ On average, the ratio of [^1^O_2_]_ss, daily average_^epilimnion^ to [^1^O_2_]_ss, daily average_^euphotic zone^ was 1.8 ± 0.7, but [^1^O_2_]_ss, daily average_^epilimnion^ were excluded from further analysis because epilimnion depths were derived from static lake surface areas^137^ rather than predicted as a function of DOC (as was done for euphotic zone depths^72^). [^1^O_2_]_ss, daily average_^euphotic zone^ were 7 ± 2% of [^1^O_2_]_ss, daily average_^near‑surface^ (4.8 × 10^–15^ to 1.3 × 10^–13^ M; Table S15), which concurred with earlier findings that depth-averaged [^1^O_2_]_ss_ are 1–2 orders of magnitude lower than near-surface [^1^O_2_]_ss_ because of light attenuation within the water column (Figures S30–S32).^48^ Compared to Φ_app,^1^O_2__, [^1^O_2_]_ss, daily average_^euphotic zone^ were less influenced by watershed hydrologic connectivity and surficial geology but followed a similar seasonal pattern of spring–summer > summer–fall > fall–winter (Figure S33). Consistently across seasons, [^1^O_2_]_ss, daily average_^euphotic zone^ were highest in cluster C lakes and lowest in cluster A lakes (Figure S34), although Φ_app,^1^O_2__ were not statistically different across lake clusters within the same season (Figure S35). Considering the similarity in water temperature, dissolved oxygen, and specific conductance among lake clusters (Figure S28), the elevated [^1^O_2_]_ss, daily average_^euphotic zone^ in cluster C lakes (Figure 4b) were largely driven by higher volumetric light absorption rates (Figure 4c), which outweighed the influence of Φ_app,^1^O_2__ when compared with clusters A and B lakes characterized by deeper euphotic zones. [^1^O_2_]_ss, daily average_^euphotic zone^ in cluster B lakes exceeded those in cluster A lakes, as expected from their comparable volumetric light absorption rates and the higher Φ_app,^1^O_2__ for cluster B samples.

Continued browning has been projected to enhance ^1^O_2_ formation and accelerate ^1^O_2_-mediated reactions in sunlit lakes.^24^ Of the ALTM lakes surveyed in this work, 12 were also sampled once in 2018 for photochemical characterization.^131^ [^1^O_2_]_ss, daily average_^euphotic zone^ in this subset of lakes were higher in 2023 than in 2018 across clusters (Figure S34) despite no significant difference in Φ_app,^1^O_2__ for samples collected in these two years (Figure S35). Generalizing ^1^O_2_ production in response to browning was not possible with such limited temporal data. To partially address this constraint, a space-for-time substitution approach^138^ was applied to [^1^O_2_]_ss, daily average_^euphotic zone^ estimated for 1469 Adirondack lakes with historical water chemistry data.^16,30^ [^1^O_2_]_ss, daily average_^euphotic zone^ (1.1 × 10^–15^–2.3 × 10^–14^ M; median 4.4 × 10^–15^ M) in this broader set of lakes showed strong positive correlations with both DOC and color (Figure S36), consistent with the relationships simulated for boreal lakes across Sweden.^25^ Should DOC and color continue to rise, [^1^O_2_]_ss, daily average_^euphotic zone^ are expected to increase; however, the degree to which spatial patterns can substitute for temporal trends warrants validation,^139^ as our estimates relied on a small photochemical data set relative to the spatiotemporal scale of interest.

To assess the significance of ^1^O_2_ in regulating contaminant dynamics in ALTM lakes, biomolecular and/or total quenching rate constants for its reactions with a variety of compounds (e.g., amino acids, peptides, pesticides, pharmaceuticals, phytoestrogens) were compiled from 66 references (Table S17). For these compounds, biomolecular reaction rate constants (*k*_rxn, ^1^O_2__) measured under environmentally relevant conditions (i.e., in aqueous solutions between 5 and 9 using competition kinetics or kinetic solvent isotope effect methods) spanned over five orders of magnitude, ranging from 6.3 × 10^3^ to 3.7 × 10^9^ M^–1^ s^–1^ with a median of 1.3 × 10^7^ M^–1^ s^–1^ (*n* = 192). Given the mildly acidic to neutral pH of ALTM samples (i.e., 6.02 ± 0.64; Table S3), a subset of 106 anthropogenic contaminants and natural toxins with *k*_rxn, ^1^O_2__ measured under similar pH conditions (Table S16) was prioritized to further evaluate their ^1^O_2_-mediated half-lives in the euphotic zone (*t*_1/2, rxn, ^1^O_2__^euphotic zone^; Figure S37). Eight of these 106 compounds, including six organic micropollutants (i.e., atrazine, fluoxetine, meclofenamic acid, metolachlor, oxybenzone, triclosan),^134^ methylmercury,^135^ and microcystin-LR,^136^ have been previously detected in Adirondack lakes. With a median *k*_rxn, ^1^O_2__ of 1.8 × 10^6^ M^–1^ s^–1^ (based on data for 106 compounds), half-lives due to reaction with ^1^O_2_ were estimated to range from 32 to 400 months in the euphotic zone of cluster A lakes, 29 to 290 months in cluster B lakes, and 17 to 160 months in cluster C lakes (Figure 4d). Comparing the log_10_-transformed ratios of *t*_1/2, rxn, ^1^O_2__^euphotic zone^ to the hydraulic residence times (τ) of ALTM lakes (Figure 4e–g) revealed that 47 of the 106 compounds yielded mean ratios below 0 (e.g., sulfonamide antibiotics,^41^ antimicrobial peptides,^44^ benzotriazole derivatives^38,42^ ) or between 0 and 1 (e.g., fenamates,^40^ antiherpesvirus agents,^39^ isoflavone phytoestrogens^43^), suggesting that ^1^O_2_-mediated reactions occur on time scales shorter than or comparable to lake flushing. For the remaining compounds with mean ratios exceeding 1, ^1^O_2_-mediated reactions are unlikely to govern their fate, but natural attenuation in ALTM lakes may still proceed via pathways involving other reactive intermediates and/or nonphotolytic transformation or partitioning processes.

## Environmental Implications

Our work integrated time series and trend analyses of long-term monitoring data sets with photochemical characterization of field samples to investigate ^1^O_2_ production in ALTM lakes, which complements prior modeling studies that predicted the effects of browning on the photochemical production of reactive intermediates in sunlit surface waters.^25–28^ Through wavelet coherence analysis, we identified recovery from acid deposition and changes in precipitation as potential drivers of DOC dynamics in ALTM lakes over longer time scales, while solar irradiance and soil wetness emerged as additional contributors at shorter time scales. Classifying browning status based on Sen’s slopes of temporal trends enabled us to perform a comparative analysis of ^1^O_2_ formation among ALTM lakes, although the broader applicability of this framework warrants further evaluation, as the extent and drivers of lake browning may vary across geographic regions^13^ or shift with the temporal window of analysis.^140^ Our study was somewhat limited in terms of photochemical data, as it focused only on ^1^O_2_. Even so, the depth-averaged steady-state concentrations of other reactive intermediates, such as ^3^DOM* (e.g., 10^–16^–10^–15^ M) and ^•^OH (e.g., 10^–18^–10^–17^ M), in the euphotic zone of ALTM lakes can be approximated through their known orders of magnitude relative to [^1^O_2_]_ss_.^48,141^ Lakes experiencing more intense browning exhibit higher [^1^O_2_]_ss_; however, whether the concentration patterns of ^3^DOM* and ^•^OH track with or diverge from that of ^1^O_2_ across the browning gradient requires additional photochemical measurements and careful consideration of uncertainties (e.g., the bimolecular reaction rate constants of probes with ^3^DOM*^141^ and the specificity of probes in distinguishing ^•^OH from lower-energy hydroxylating species^142^). Concurrent with browning, warming of lake surface waters^16^ also alters nutrient availability, light regimes, and thermal conditions,^16,128,139^ which may further complicate predictions of ^1^O_2_ production due to cascading effects on DOM properties^17^ and pathways involved in ^1^O_2_ formation and quenching.^49^ Overall, this study serves as a springboard for further research into the impact of browning on photochemistry in Adirondack lakes and similar aquatic systems and underscores the need for long-term monitoring of DOM quality and photochemical parameters to better understand their implications for ecosystem function and water quality management.

## Supplementary Material



## Notes

Map, morphometric characteristics, and watershed attributes of Adirondack Long-Term Monitoring (ALTM) lakes. Water chemistry parameters and optical properties of ALTM samples. Photochemistry experimental details and summary of photochemical parameters. Wavelet coherence tests between the time series of DOC and external drivers. Sen’s slopes for the time series of DOC, color, and SUVA_254_. Comparisons of Φ_app,^1^O_2__ for ALTM samples with literature data. Linear regression between Φ_app,^1^O_2__ and selected optical properties. Generalized additive modeling of seasonal variations in Φ_app,^1^O_2__. Steady-state concentrations and depth profiles of ^1^O_2_ in the euphotic zone of ALTM lakes. Half-lives of selected contaminants in the euphotic zone due to reaction with ^1^O_2_. Summary of literature data on *k*_rxn,^1^O_2__ and Φ_app,^1^O_2__.(PDF)

## §

JKMuir, LLC, 2275 Silas Deane Highway, Rocky Hill, Connecticut 06067, United States
