# Supplementary material for: Photochemical Production of Singlet Oxygen in Adirondack Long-Term Monitoring Lakes of Varying Browning Status
Source: Environ Sci Technol. 2025 Jul 2;59(27):13992–4005. doi: 10.1021/acs.est.5c04001 (PMC12269092; doi:10.1021/acs.est.5c04001)
Supplement: Supplementary file 1 [file es5c04001_si_001.pdf]

Supporting Information for

Photochemical Production of Singlet Oxygen in Adirondack Long-Term Monitoring Lakes of Varying Browning Status

Birdem Öz[1], Philip K. Snyder[2], Xiaoyu Jiao[1], Charles T. Driscoll[1], Teng Zeng[,1]*

[1]Department of Civil and Environmental Engineering, Syracuse University, 151 Link Hall, Syracuse, New York 13244, United States

[2]Ausable Freshwater Center, 5698 NY-86, Wilmington, New York 12997, United States

*Corresponding Author: Teng Zeng: Email: tezeng@syr.edu; Phone: +1-315-443-1099

(Total 158 pages, 6 texts, 18 tables, 37 figures)

Table of Contents

1. Chemical sources and reagents preparation S3

2. Map of Adirondack Long-Term Monitoring (ALTM) lakes S4

3. Morphometric characteristics and watershed attributes of ALTM lakes S5

4. Field sampling on ALTM lakes S10

5. Water chemistry parameters and optical properties of ALTM samples S11

6. *p*-Nitroanisole/pyridine actinometry S32

7. Measurements of the apparent quantum yields of $^1O_2$ S34

8. Calculations of steady-state concentrations of $^1O_2$ S36

9. Wavelet coherences between the time series of DOC in ALTM lakes and external drivers S39

10. Sen's slopes for the time series of DOC, color, and $SUVA_{254}$ in ALTM lakes S46

11. $\Phi_{app,\ ^1O_2}$ for ALTM samples and comparisons with literature data S54

12. Spatiotemporal patterns of optical properties of ALTM samples S57

13. Optical properties as predictors of $\Phi_{app,\ ^1O_2}$ for ATLM samples S62

14. Comparisons of water chemistry and optical properties of ALTM samples by browning status S77

15. Steady-state concentrations and depth profiles of $^1O_2$ in ALTM lakes S79

16. Comparisons of steady-state concentrations of $^1O_2$ and $\Phi_{app,\ ^1O_2}$ by lake browning status S92

17. Calculations of contaminant half-lives mediated by $^1O_2$ reactions S95

18. Literature survey of total quenching and bimolecular reaction rate constants with $^1O_2$ S100

19. Literature survey of $\Phi_{app,\ ^1O_2}$ and optical data S106

References S147

1. Chemical sources and reagents preparation

Methanol (MeOH; HPLC grade), water (HPLC grade), sodium hydroxide solution (NaOH; 0.1 N certified), hydrochloric acid solution (HCl; 0.1 N certified), and *o*-phosphoric acid (H_3PO_4; certified ACS grade) were purchased from Fisher Scientific. Sodium dihydrogen phosphate monohydrate ($NaH_2PO_4 \bullet H_2O$; 99+%), sodium phosphate dibasic heptahydrate ($Na_2HPO_4 \bullet 7H_2O$; 99+%), furfuryl alcohol (FFA; 98%), *p*-nitroanisole (PNA; 99+%), and pyridine (pyr; 99+%) were purchased from Acros Organics. Potassium hydrogen phthalate solution (1000 ppm C) and potassium nitrate solution (1000 ppm N) were purchased from RICCA Chemical. pH buffer solutions (pH 4.01, 7.00, and 10.01) and conductivity calibration solution (1413 μS/cm) were purchased from Mettler Toledo. Suwannee River natural organic matter (SRNOM; 2R101N), Suwannee River fulvic acid (SRFA; 3S101F), and Suwannee River humic acid (SRHA; 3S101H) were purchased from the International Humic Substance Society (IHSS).

Stock solutions were prepared by dissolving or diluting a gravimetrically weighted amount of solid or liquid standards into HPLC grade water. Bimolecular PNA/pyr actinometer solutions (10 μM PNA/5 mM pyr) were freshly prepared by mixing 10 mM of PNA and 12.36 M of pyridine stock solutions at a predetermined volumetric ratio.[1, 2] Working solutions and calibration standards were prepared by diluting predetermined volumes of stock solutions into ultrapure water (resistivity 18.2 MΩ•cm) produced by a Thermo Scientific Barnstead MicroPure UV/UF water purification system. Stock and working solutions were stored at 4 °C. Mobile phases for HPLC analysis were prepared using HPLC grade water and solvents.

Non-volumetric glassware was rinsed with HPLC grade methanol, followed by ultrapure water, and combusted at 450 °C in a Thermo Scientific Lindberg/Blue M Moldatherm box furnace for a minimum of 5 h. Volumetric glassware, quartz tubes, and microsyringes were rinsed with HPLC grade methanol and ultrapure water and dried at 70 °C in a Fisherbrand Isotemp general purpose heating and drying oven.

2. Map of Adirondack Long-Term Monitoring (ALTM) lakes

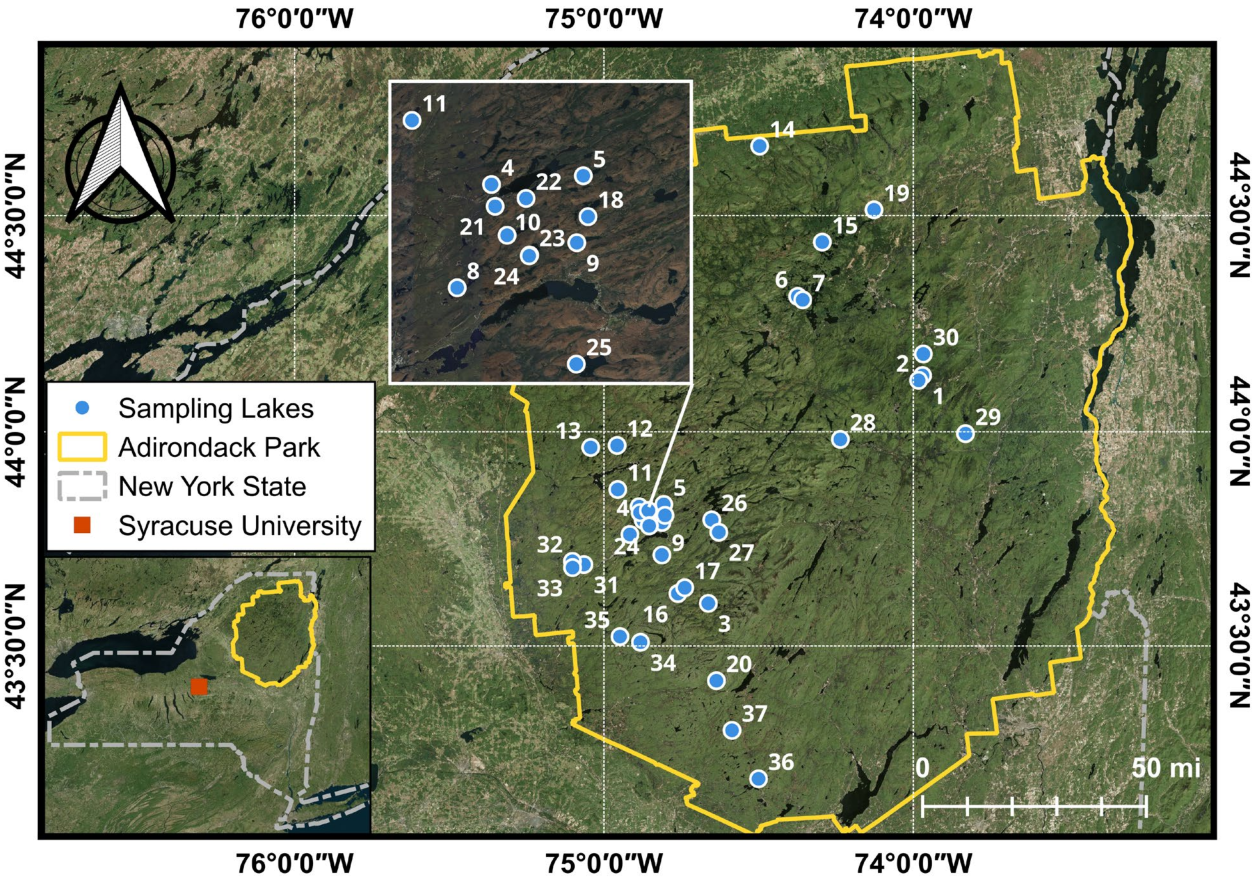

Figure S1. Map of Adirondack Long-Term Monitoring (ALTM) lakes (with site IDs) sampled in the current study: 1 - Avalanche Lake (050707-1); 2 - Lake Colden (050706-1); 3 - Brook Trout Lake (040874-1); 4 - Squash Pond (040754-2); 5 - Constable Pond (040777-2); 6 - East Copperas Pond (020138-1); 7 - Little Echo Pond (020126-1); 8 - Lake Rondaxe (040739-1); 9 - Cascade Lake (040747-1); 10 - Dart Lake (040750-1); 11 - Woods Lake (040576-1); 12 - Willys Lake (040210-1); 13 - Loon Hollow Pond (040186-1); 14 - Little Clear Pond (030172-1); 15 - Black Pond (030256-1); 16 - Indian Lake (040852-1); 17 - Squaw Lake (040850-1); 18 - Queer Lake (060329-1); 19 - Big Hope Pond (020059-1); 20 - G Lake (070859-1); 21 - West Pond (040753-2); 22 - Big Moose Lake (040752-1); 23 - Moss Lake (040746-1); 24 - Bubb Lake (040748-2); 25 - Limekiln Lake (040826-1); 26 - Raquette Lake Reservoir (060315A-1); 27 - Sagamore Lake (060313-1); 28 - Arbutus Pond (050684-1); 29 - Clear Pond (050458-1); 30 - Heart Lake (020264-1); 31 - Grass Pond (040706-1); 32 - Middle Branch Lake (040707-1); 33 - Middle Settlement Lake (040704-1); 34 - South Lake (041004-1); 35 - North Lake (041007-1); 36 - Otter Lake (070729-1); 37 - Jockeybush Lake (050259-1). Morphometric characteristics and watershed attributes of these lakes are summarized in Table S1. Satellite Image Source: Esri, Maxar, GeoEye, Earthstar Geographics, CNES/Airbus DS, USDA, USGS, AeroGRID, IGN, and the GIS User Community.

3. Morphometric characteristics and watershed attributes of ALTM lakes

Table S1. Morphometric characteristics and watershed attributes of ALTM lakes

Lake Name	Site ID	Latitude	Longitude	Hydrologic Connectivity	Surficial Geology	Mean Depth (m)	Maximum Depth (m)	Hydraulic Residence Time (year)
Avalanche Lake	050707-1	44.13056	-73.96983	Headwater Drainage	Thin Till	7.0	3.3	0.20
Lake Colden	050706-1	44.11938	-73.98261	Chain Drainage	Thin Till	7.3	2.3	0.09
Brook Trout Lake	040874-1	43.59990	-74.66236	Headwater Drainage	Thin Till	23.2	8.4	1.43
Squash Pond	040754-2	43.82557	-74.88614	Chain Drainage	Thin Till	5.8	1.4	0.14
Constable Pond	040777-2	43.83101	-74.80642	Chain Drainage	Thin Till	4.0	2.1	0.06
East Copperas Pond	020138-1	44.31384	-74.37335	Headwater Drainage	Thin Till	6.4	4.1	1.67
Little Echo Pond	020126-1	44.30576	-74.35721	Mounded Seepage	Seepage	4.6	2.9	1.24
Lake Rondaxe	040739-1	43.76088	-74.91592	Chain Drainage	Thin Till	10.1	3.0	0.03
Cascade Lake	040747-1	43.78910	-74.81204	Chain Drainage	Medium Till	6.1	4.2	0.48
Dart Lake	040750-1	43.79376	-74.87257	Chain Drainage	Thin Till	17.7	7.3	0.05
Woods Lake	040576-1	43.86566	-74.95512	Headwater Drainage	Thin Till	10.1	3.5	0.51
Willys Lake	040210-1	43.96821	-74.95708	Headwater Drainage	Thin Till	13.7	4.9	1.00
Loon Hollow Pond	040186-1	43.96360	-75.04253	Headwater Drainage	Thin Till	11.6	3.4	0.42
Little Clear Pond	030172-1	44.66022	-74.49632	Mounded Seepage	Seepage	14.0	5.5	0.42
Black Pond	030256-1	44.43910	-74.2939	Chain Drainage	Thick Till	13.7	6.2	0.67
Indian Lake	040852-1	43.62286	-74.76075	Chain Drainage	Thin Till	10.7	3.0	0.10
Squaw Lake	040850-1	43.63645	-74.73844	Chain Drainage	Thin Till	6.7	3.4	0.77
Queer Lake	060329-1	43.80543	-74.80230	Chain Drainage	Thin Till	21.3	10.9	5.00
Big Hope Pond	020059-1	44.51360	-74.12673	Headwater Drainage	Medium Till	11.5	5.8	0.42
G Lake	070859-1	43.41862	-74.63616	Headwater Drainage	Thin Till	9.8	4.5	0.39
West Pond	040753-2	43.81189	-74.88296	Headwater Drainage	Thin Till	5.2	1.5	0.19
Big Moose Lake	040752-1	43.81687	-74.85611	Chain Drainage	Thin Till	21.3	6.8	0.48
Moss Lake	040746-1	43.78140	-74.85299	Chain Drainage	Medium Till	15.2	5.7	0.63
Bubb Lake	040748-2	43.78088	-74.85339	Chain Drainage	Thin Till	4.3	2.1	0.27
Limekiln Lake	040826-1	43.71301	-74.81246	Chain Drainage	Medium Till	21.9	6.1	1.43
Raquette Lake Reservoir	060315A-1	43.79492	-74.65130	Headwater Drainage	Medium Till	3.0	1.6	0.02
Sagamore Lake	060313-1	43.76605	-74.62837	Chain Drainage	Medium Till	22.9	10.5	0.20
Arbutus Pond	050684-1	43.98230	-74.23556	Headwater Drainage	Medium Till	7.9	2.8	0.50
Clear Pond	050458-1	43.99568	-73.83082	Headwater Drainage	Thick Till	24.4	9.2	1.67
Heart Lake	020264-1	44.18050	-73.96726	Headwater Drainage	Medium Till	16.8	5.1	1.11
Grass Pond	040706-1	43.69066	-75.06462	Headwater Drainage	Medium Till	5.2	1.5	0.04
Middle Branch Lake	040707-1	43.69815	-75.10160	Headwater Drainage	Thin Till	5.2	2.1	0.13
Middle Settlement Lake	040704-1	43.68348	-75.10116	Headwater Drainage	Thin Till	11.0	3.4	0.71
South Lake	041004-1	43.50877	-74.88168	Chain Drainage	Thin Till	18.3	8.3	1.30
North Lake	041007-1	43.52218	-74.94789	Chain Drainage	Thin Till	17.7	5.7	0.14
Otter Lake	070729-1	43.18805	-74.50000	Chain Drainage	Thin Till	4.0	2.3	0.14
Jockeybush Lake	050259-1	43.30203	-74.58581	Headwater Drainage	Thin Till	11.3	4.5	0.59

Table S1. Morphometric characteristics and watershed attributes of ALTM lakes (continued)								
Site Name	Site ID	Volume (×10^4 m^3)	Watershed Area : Surface Area	Surface Area (ha)	Watershed Area (ha)	Site Elevation (m)	Watershed Elevation (m)	Watershed Slope (m)
Avalanche Lake	050707-1	14.6	26.18	4.4	115.2	873	1094.2	64.44
Lake Colden	050706-1	35.5	42.62	15.4	656.3	843	1300.7	41.57
Brook Trout Lake	040874-1	242.0	5.77	28.7	165.7	724	774.4	22.21
Squash Pond	040754-2	4.5	37.91	3.3	125.1	653	677.8	13.90
Constable Pond	040777-2	43.5	45.50	20.6	937.4	580	641.0	13.29
East Copperas Pond	020138-1	14.8	3.61	3.6	13.0	480	483.7	3.26
Little Echo Pond	020126-1	2.3	1.25	0.8	1.0	482	483.1	1.39
Lake Rondaxe	040739-1	273.3	156.42	90.5	14155.6	524	639.2	14.10
Cascade Lake	040747-1	171.9	12.35	40.4	498.8	557	610.1	18.98
Dart Lake	040750-1	380.7	208.58	51.8	10804.5	537	647.2	13.59
Woods Lake	040576-1	80.9	8.45	24.7	208.7	605	637.0	15.13
Willys Lake	040210-1	118.8	6.51	24.3	158.2	632	654.1	13.43
Loon Hollow Pond	040186-1	19.1	9.68	5.7	55.2	605	624.6	10.90
Little Clear Pond	030172-1	10.2	9.47	1.9	17.99	381	408.4	8.81
Black Pond	030256-1	180.5	8.20	29.0	237.9	495	522.2	13.20
Indian Lake	040852-1	98.1	33.78	33.2	1121.4	654	728.8	13.61
Squaw Lake	040850-1	124.9	5.02	36.4	182.7	646	667.9	10.11
Queer Lake	060329-1	596.0	6.89	54.5	375.4	597	632.4	13.68
Big Hope Pond	020059-1	51.6	13.39	8.9	119.2	517	536.6	7.09
G Lake	070859-1	143.7	12.72	32.2	409.6	620	717.5	16.57
West Pond	040753-2	15.2	9.58	10.4	99.6	581	595.0	9.94
Big Moose Lake	040752-1	3488.2	18.82	512.5	9643.8	558	649.2	13.47
Moss Lake	040746-1	259.8	27.02	45.7	1234.6	536	605.4	16.42
Bubb Lake	040748-2	38.5	10.94	18.2	199.1	554	617.4	7.58
Limekiln Lake	040826-1	1147.6	7.54	186.9	1409.7	575	642.6	15.28
Raquette Lake Reservoir	060315A-1	2.4	203.67	1.5	305.5	564	624.6	15.50
Sagamore Lake	060313-1	713.1	69.46	68.0	4723.0	580	721.3	13.33
Arbutus Pond	050684-1	134.5	7.24	48.9	354.0	516	664.2	21.19
Clear Pond	050458-1	651.1	8.03	70.4	565.0	584	681.3	17.89
Heart Lake	020264-1	54.5	6.48	10.7	69.3	661	694.9	19.46
Grass Pond	040706-1	7.8	51.32	5.3	272.0	549	603.3	13.58
Middle Branch Lake	040707-1	36.3	7.62	17.0	129.6	496	522.7	9.65
Middle Settlement Lake	040704-1	54.5	7.23	15.8	114.3	526	551.8	13.21
South Lake	041004-1	1630.2	8.42	197.4	1662.2	615	678.1	13.42
North Lake	041007-1	1010.7	43.56	176.8	7700.8	555	671.5	12.76
Otter Lake	070729-1	34.1	23.03	14.8	340.8	505	573.0	15.85
Jockeybush Lake	050259-1	78.6	9.25	17.3	160.0	599	643.2	19.20

**Table S1.** Morphometric characteristics and watershed attributes of ALTM lakes (continued)									
Site Name	Site ID	Deciduous Forest (%)	Conifer Forest (%)	Decid-Conif Mix (%)	Shrub-Sapling Area (%)	Wetland (%)	Open Grass (%)	Agricultural (%)	Developed (%)
		Watershed Level							
Avalanche Lake	050707-1	0	60	20	20	0	0	0	0
Lake Colden	050706-1	0	80	10	7	3	0	0	0
Brook Trout Lake	040874-1	80	0	20	0	0	0	0	0
Squash Pond	040754-2	40	0	50	10	0	0	0	0
Constable Pond	040777-2	60	10	20	0	0	10	0	0
East Copperas Pond	020138-1	0	80	20	0	0	0	0	0
Little Echo Pond	020126-1	0	0	0	0	0	0	0	0
Lake Rondaxe	040739-1	5	20	70	5	0	0	0	0
Cascade Lake	040747-1	60	40	0	0	0	0	0	0
Dart Lake	040750-1	50	0	43	5	0	1	0	1
Woods Lake	040576-1	0	0	0	0	0	0	0	0
Willys Lake	040210-1	0	10	90	0	0	0	0	0
Loon Hollow Pond	040186-1	0	0	98	0	2	0	0	0
Little Clear Pond	030172-1	95	0	5	0	0	0	0	0
Black Pond	030256-1	10	0	75	15	0	0	0	0
Indian Lake	040852-1	89	10	0	0	1	0	0	0
Squaw Lake	040850-1	80	20	0	0	0	0	0	0
Queer Lake	060329-1	5	5	90	0	0	0	0	0
Big Hope Pond	020059-1	0	80	20	0	0	0	0	0
G Lake	070859-1	0	0	0	0	0	0	0	0
West Pond	040753-2	0	20	80	0	0	0	0	0
Big Moose Lake	040752-1	0	0	0	0	0	0	0	0
Moss Lake	040746-1	17	0	80	2	1	0	0	0
Bubb Lake	040748-2	10	5	85	0	0	0	0	0
Limekiln Lake	040826-1	90	3	7	0	0	0	0	0
Raquette Lake Reservoir	060315A-1	45	50	0	2	3	0	0	0
Sagamore Lake	060313-1	10	5	85	0	0	0	0	0
Arbutus Pond	050684-1	0	0	0	0	0	0	0	0
Clear Pond	050458-1	80	5	15	0	0	0	0	0
Heart Lake	020264-1	75	15	0	0	0	0	0	10
Grass Pond	040706-1	0	0	85	5	10	0	0	0
Middle Branch Lake	040707-1	90	0	10	0	0	0	0	0
Middle Settlement Lake	040704-1	0	0	95	2	3	0	0	0
South Lake	041004-1	0	0	0	0	0	0	0	0
North Lake	041007-1	90	0	8	1	0	0	0	1
Otter Lake	070729-1	0	0	0	0	0	0	0	0
Jockeybush Lake	050259-1	75	0	20	5	0	0	0	0

Table S1. Morphometric characteristics and watershed attributes of ALTM lakes (continued)											
Site Name	Site ID	Deciduous Forest (%)	Conifer Forest (%)	Decid-Conif Mix (%)	Shrub-Sapling Area (%)	Wetland (%)	Open Grass (%)	Agricultural (%)	Developed (%)	Sand-Gravel Beach (%)	Boulder Rock Ledge (%)
		Shoreline Level									
Avalanche Lake	050707-1	0	5	0	5	0	0	0	0	0	90
Lake Colden	050706-1	0	70	0	12	9	1	0	1	0	7
Brook Trout Lake	040874-1	0	95	5	0	0	0	0	0	0	0
Squash Pond	040754-2	0	0	70	0	28	0	0	0	0	2
Constable Pond	040777-2	10	10	0	20	0	30	0	0	10	0
East Copperas Pond	020138-1	0	99	0	0	0	0	0	0	0	0
Little Echo Pond	020126-1	0	0	0	0	0	0	0	0	0	0
Lake Rondaxe	040739-1	0	18	60	0	2	0	0	15	5	0
Cascade Lake	040747-1	0	0	89	10	0	1	0	0	0	0
Dart Lake	040750-1	0	30	54	5	1	1	0	5	2	2
Woods Lake	040576-1	0	0	0	0	0	0	0	0	0	0
Willys Lake	040210-1	0	70	10	0	10	0	0	0	0	10
Loon Hollow Pond	040186-1	0	0	95	0	5	0	0	0	0	0
Little Clear Pond	030172-1	20	10	70	0	0	0	0	0	0	0
Black Pond	030256-1	25	10	50	15	0	0	0	0	0	0
Indian Lake	040852-1	30	50	0	10	10	0	0	0	0	0
Squaw Lake	040850-1	35	55	0	5	0	0	0	0	0	5
Queer Lake	060329-1	0	20	78	0	0	0	0	0	0	2
Big Hope Pond	020059-1	0	10	0	10	80	0	0	0	0	0
G Lake	070859-1	0	0	0	0	0	0	0	0	0	0
West Pond	040753-2	0	50	10	0	40	0	0	0	0	0
Big Moose Lake	040752-1	0	0	0	0	0	0	0	0	0	0
Moss Lake	040746-1	0	0	78	10	1	0	0	0	10	1
Bubb Lake	040748-2	0	20	78	0	2	0	0	0	0	0
Limekiln Lake	040826-1	0	70	9	0	0	0	0	10	10	1
Raquette Lake Reservoir	060315A-1	20	70	0	5	4	1	0	0	0	0
Sagamore Lake	060313-1	0	69	20	5	0	0	0	1	0	5
Arbutus Pond	050684-1	0	0	0	0	0	0	0	0	0	0
Clear Pond	050458-1	5	5	85	5	0	0	0	0	0	0
Heart Lake	020264-1	0	15	80	0	0	0	0	5	0	0
Grass Pond	040706-1	0	0	80	10	10	0	0	0	0	0
Middle Branch Lake	040707-1	90	0	10	0	0	0	0	0	0	0
Middle Settlement Lake	040704-1	0	0	70	20	10	0	0	0	0	0
South Lake	041004-1	0	0	0	0	0	0	0	0	0	0
North Lake	041007-1	70	0	10	5	0	1	0	10	1	3
Otter Lake	070729-1	0	0	0	0	0	0	0	0	0	0
Jockeybush Lake	050259-1	0	40	55	0	3	0	0	0	0	2

**Table S1.** Morphometric characteristics and watershed attributes of ALTM lakes (continued)										
Site Name	Site ID	Bedrock (%)	Boulder (%)	Rubble (%)	Gravel (%)	Sand (%)	MuckSilt (%)	Clay (%)	Organic (%)	Other (%)
		Watershed Substrate								
Avalanche Lake	050707-1	20	40	5	0	5	15	0	15	0
Lake Colden	050706-1	0	5	15	10	20	25	0	25	0
Brook Trout Lake	040874-1	10	30	0	0	0	20	0	40	0
Squash Pond	040754-2	0	2	0	0	0	50	0	48	0
Constable Pond	040777-2	0	0	70	0	0	30	0	0	0
East Copperas Pond	020138-1	0	0	0	0	0	80	0	20	0
Little Echo Pond	020126-1	0	0	0	0	0	0	0	0	0
Lake Rondaxe	040739-1	0	0	0	5	30	45	0	20	0
Cascade Lake	040747-1	0	2	70	10	15	3	0	0	0
Dart Lake	040750-1	1	5	22	22	50	0	0	0	0
Woods Lake	040576-1	0	0	0	0	0	0	0	0	0
Willys Lake	040210-1	0	10	0	0	60	20	0	10	0
Loon Hollow Pond	040186-1	1	5	0	0	0	93	0	1	0
Little Clear Pond	030172-1	0	0	0	0	0	80	0	20	0
Black Pond	030256-1	0	5	30	40	15	5	0	5	0
Indian Lake	040852-1	1	1	0	0	0	39	0	59	0
Squaw Lake	040850-1	5	10	35	10	0	35	0	5	0
Queer Lake	060329-1	10	30	15	5	30	10	0	0	0
Big Hope Pond	020059-1	0	10	0	40	0	20	0	30	0
G Lake	070859-1	0	0	0	0	0	0	0	0	0
West Pond	040753-2	5	5	0	0	5	10	0	75	0
Big Moose Lake	040752-1	0	0	0	0	0	0	0	0	0
Moss Lake	040746-1	2	3	20	20	54	1	0	0	0
Bubb Lake	040748-2	10	25	15	0	10	40	0	0	0
Limekiln Lake	040826-1	3	5	5	5	80	2	0	0	0
Raquette Lake Reservoir	060315A-1	10	20	10	10	40	10	0	0	0
Sagamore Lake	060313-1	0	5	30	10	50	0	0	5	0
Arbutus Pond	050684-1	0	0	0	0	0	0	0	0	0
Clear Pond	050458-1	0	10	5	30	50	0	0	5	0
Heart Lake	020264-1	0	10	5	0	70	0	0	15	0
Grass Pond	040706-1	0	30	0	0	0	60	0	10	0
Middle Branch Lake	040707-1	0	10	10	50	30	0	0	0	0
Middle Settlement Lake	040704-1	5	15	10	10	15	45	0	0	0
South Lake	041004-1	0	0	0	0	0	0	0	0	0
North Lake	041007-1	0	10	15	25	45	5	0	0	0
Otter Lake	070729-1	0	0	0	0	0	0	0	0	0
Jockeybush Lake	050259-1	0	5	0	10	65	0	0	20	0

4. Field sampling on ALTM lakes

Table S2. Sampling dates and times on ALTM lakes

Site Name	Site ID	Sampling Date	Sampling Time	Sampling Date	Sampling Time	Sampling Date	Sampling Time	Sampling Site
Avalanche Lake	050707-1	10/17/2022	10:05	6/2/2023	12:25	9/25/2023	12:05	Mid-Lake
Lake Colden	050706-1	10/17/2022	11:00	6/2/2023	11:00	9/25/2023	10:05	Mid-Lake
Brook Trout Lake	040874-1	10/26/2022	11:30	5/22/2023	13:30	9/5/2023	13:30	Mid-Lake
Squash Pond Outlet	040754-1	10/31/2022	10:13	5/15/2023	10:34	9/18/2023	15:00	Outlet
Squash Pond	040754-2	10/31/2022	10:52	5/15/2023	10:58	9/18/2023	14:25	Mid-Lake
Constable Pond Outlet	040777-1	10/31/2022	11:35	5/15/2023	11:32	9/18/2023	13:30	Outlet
Constable Pond	040777-2	10/31/2022	12:26	5/15/2023	12:25	9/18/2023	12:30	Mid-Lake
East Copperas Pond	020138-1	11/1/2022	10:25	6/27/2023	12:10	9/15/2023	12:35	Mid-Lake
Little Echo Pond	020126-1	11/1/2022	11:14	6/29/2023	17:15	9/21/2023	14:30	Mid-Lake
Lake Rondaxe	040739-1	11/2/2022	11:11	6/19/2023	16:50	9/23/2023	14:00	Mid-Lake
Cascade Lake	040747-1	11/2/2022	12:12	6/16/2023	11:45	9/18/2023	10:40	Mid-Lake
Cascade Lake Outlet	040747A-1	11/2/2022	13:00	6/16/2023	12:35	9/18/2023	11:20	Outlet
Dart Lake	040750-1	11/2/2022	13:35	5/23/2023	16:30	9/22/2023	16:30	Mid-Lake
Woods Lake	040576-1	11/2/2022	14:48	6/16/2023	14:30	9/18/2023	16:30	Mid-Lake
Willys Lake	040210-1	11/3/2022	11:15	5/23/2023	14:40	9/19/2023	10:35	Mid-Lake
Loon Hollow Pond	040186-1	11/3/2022	14:40	5/23/2023	18:45	9/19/2023	14:55	Mid-Lake
Little Clear Pond	030172-1	11/7/2022	11:20	6/22/2023	11:15	9/12/2023	12:30	Mid-Lake
Black Pond	030255-1	11/7/2022	12:55	6/29/2023	18:10	9/21/2023	10:43	Outlet
Indian Lake	040852-1	11/8/2022	12:00	5/22/2023	12:30	9/5/2023	12:00	Mid-Lake
Squaw Lake	040850-1	11/8/2022	14:10	5/22/2023	10:55	9/5/2023	13:55	Mid-Lake
Queer Lake	060329-1	11/9/2022	12:20	6/6/2023	12:30	9/13/2023	12:20	Mid-Lake
Big Hope Pond	020059-1	11/10/2022	8:20	6/23/2023	18:40	9/21/2023	12:30	Mid-Lake
G Lake	070859-1	11/10/2022	10:57	6/29/2023	13:05	9/12/2023	9:45	Mid-Lake
West Pond Outlet	040753-1	11/14/2022	11:05	6/16/2023	13:05	9/4/2023	13:25	Outlet
West Pond	040753-2	11/14/2022	11:12	6/16/2023	13:20	9/4/2023	12:52	Mid-Lake
Big Moose Lake	040752-1	11/14/2022	11:45	6/16/2023	15:30	9/4/2023	14:03	Mid-Lake
Moss Lake	040746-1	11/14/2022	12:15	5/25/2023	11:50	9/26/2023	12:10	Mid-Lake
Bubb Lake	040748-2	11/14/2022	12:30	5/25/2023	10:55	9/4/2023	16:50	Mid-Lake
Bubb Lake Outlet	040748-1	11/14/2022	12:45	5/25/2023	11:10	9/4/2023	15:56	Outlet
Limekiln Lake	040826-1	11/15/2022	10:12	6/6/2023	14:50	9/4/2023	10:21	Mid-Lake
Raquette Lake Reservoir	060315A-1	11/15/2022	11:03	5/23/2023	10:10	9/26/2023	13:33	Mid-Lake
Sagamore Lake	060313-1	11/15/2022	11:30	6/20/2023	13:15	9/29/2023	14:45	Mid-Lake
Arbutus Pond	050684-1	11/15/2022	12:55	6/23/2023	13:20	9/26/2023	11:20	Mid-Lake
Clear Pond	050458-1	11/15/2022	13:45	6/23/2023	11:15	9/26/2023	10:15	Mid-Lake
Heart Lake	020264-1	11/15/2022	15:00	6/13/2023	13:55	9/5/2023	15:50	Mid-Lake
Grass Pond	040706-1	11/17/2022	11:40	5/16/2023	11:05	9/4/2023	10:17	Mid-Lake
Middle Branch Lake	040707-1	11/17/2022	12:55	5/16/2023	12:15	9/4/2023	12:25	Mid-Lake
Middle Settlement Lake	040704-1	11/17/2022	13:30	5/16/2023	12:36	9/4/2023	14:09	Mid-Lake
South Lake	041004-1	11/28/2022	11:55	6/26/2023	13:00	9/6/2023	10:50	Mid-Lake
North Lake	041007-1	11/28/2022	13:00	6/26/2023	14:00	9/6/2023	12:10	Mid-Lake
Otter Lake Outlet	070728-1	11/29/2022	11:25	6/5/2023	13:00	9/14/2023	10:20	Outlet
Otter Lake	070729-1	11/29/2022	11:30	6/5/2023	13:10	9/14/2023	10:40	Mid-Lake
Jockeybush Lake	050259-1	11/29/2022	13:05	6/5/2023	11:50	9/12/2023	12:58	Mid-Lake

5. Water chemistry parameters and optical properties of ALTM samples

For each sample, water chemistry parameters, including pH, DOC, dissolved inorganic carbon (DIC), specific conductance, ammonium, acid neutralizing capacity (ANC), acid anions, base cations, and speciated aluminum (total dissolved Al, total monomeric Al, organic monomeric Al, inorganic monomeric Al), were analyzed by the U.S. Geological Survey (USGS) New York Water Science Center Soil and Low-Ionic-Strength Water Quality Laboratory (Troy, NY) using standard methods.[3-8] Concentrations of metals (e.g., iron, manganese, copper, and zinc) were measured at Syracuse University using a PerkinElmer NexION 2000 inductively coupled plasma mass spectrometer. Optical properties were also measured at Syracuse University using a Horiba Scientific Aqualog spectrofluorometer. Excitation-emission matrices (EEMs) were recorded across an excitation wavelength range of 240 to 650 nm in 1-nm increments and an emission wavelength range of 247.68 to 830.02 nm in 2.33-nm increments with an integration time of 1 s and a medium CCD gain. EEMs were corrected for instrument-specific correction factors[9] and inner filter effects,[10, 11] blank subtracted, and normalized against the Raman peak area of a Starna Cells RM-H_2O Raman water fluorescence reference standard.[12, 13] Concurrently, UV-visible absorbance spectra were recorded from 240 to 650 nm in 1-nm increments and corrected for blank and long-wavelength baseline. Optical indices, such as Napierian absorption coefficients (a) at 254 nm, 280 nm, and 440 nm,[14] $SUVA_{254}$ (the specific UV absorbance at 254 nm),[15] $E2{:}E3$ (the ratio of Napierian absorption coefficients at 250 and 365 nm),[16] spectral slope coefficients (e.g., $S_{275\text{-}295}$, $S_{350\text{-}400}$, $S_{290\text{-}400}$, and $S_{300\text{-}600}$),[17-19] spectral slope ratio (S_R; the ratio of $S_{275\text{-}295}$ to $S_{350\text{-}400}$),[20] fluorescence index (FI),[21] humification index (HIX),[22] and freshness index ($\beta{:}\alpha$),[23] were extracted from the absorbance and fluorescence data using *MATLAB R2022a*.

Parallel factor analysis (PARAFAC) was also performed to deconvolute EEMs (n = 266) using the *drEEM* toolbox (v. 0.6.5)[13] in *MATLAB*. Upon examination of the core consistencies, sum of squared errors, and $S_4C_6T_3$ split-half validations (**Figure S2**),[13] a 4-component model was identified as the most appropriate model that explained 99.8% of the measured spectral variation across reverse-normalized EEMs. Following the model validation, the true scores were converted to the maximum fluorescence intensity in water Raman unit (R.U.),[13] and the excitation and emission wavelengths of each component were queried through the *OpenFluor* database.[24]

Four PARAFAC components were defined as component 1 (C1), component 2 (C2), component 3 (C3), and component 4 (C4), respectively (**Figures S3-S4**). C1 is a terrestrial humic-like component (high aromaticity, high molecular weight),[25] C2 is another terrestrial humic-like component (aromatic, high molecular weight),[26] C3 resembles a reduced, humic-like component,[26] and C4 is a tryptophan-like component (aliphatic, low molecular weight).[25, 26]

Water chemistry parameters and optical properties of ALTM samples are summarized in **Tables S3-S4**.

Table S3. Water chemistry parameters of whole water samples from ALTM lakes

Site Name	Sampling Date	pH	DOC (mg C/L)	DIC (mg C/L)	$[NH_4^+]$ (μM)	$[Cl^-]$ (μM)	$[NO_3^-]$ (μM)	$[SO_4^{2-}]$ (μM)	ANC (μeq/L)	Specific Conductance (μS/cm)
Avalanche Lake	10/17/2022	5.55(±0.11)	7.83(±0.12)	0.97(±0.02)	3.57(±0.07)	5.12(±0.10)	12.26(±0.25)	10.15(±0.20)	14.6(±0.3)	10.2(±0.2)
Lake Colden	10/17/2022	5.54(±0.11)	7.30(±0.12)	0.69(±0.01)	0.78(±0.02)	4.97(±0.10)	11.65(±0.23)	12.41(±0.25)	16.5(±0.3)	10.3(±0.2)
Brook Trout Lake	10/26/2022	6.24(±0.12)	3.53(±0.01)	0.68(±0.01)	0.87(±0.02)	5.36(±0.11)	1.03(±0.02)	15.29(±0.31)	17.6(±0.4)	7.5(±0.2)
Squash Pond Outlet	10/31/2022	5.02(±0.10)	7.65(±0.12)	<0.5	0.63(±0.01)	9.25(±0.19)	1.17(±0.02)	15.75(±0.32)	-6.0(±0.1)	12.3(±0.2)
Squash Pond	10/31/2022	4.86(±0.10)	11.03(±0.09)	1.10(±0.02)	1.28(±0.03)	9.46(±0.19)	<0.1	12.44(±0.25)	19.6(±0.4)	13.5(±0.3)
Constable Pond Outlet	10/31/2022	6.06(±0.12)	6.96(±0.13)	<0.5	0.59(±0.01)	7.44(±0.15)	1.70(±0.03)	19.23(±0.38)	35.2(±0.7)	12.6(±0.3)
Constable Pond	10/31/2022	5.58(±0.11)	7.52(±0.11)	0.80(±0.02)	0.65(±0.01)	7.36(±0.15)	<0.1	18.44(±0.37)	29.4(±0.6)	11.9(±0.2)
East Copperas Pond	11/1/2022	4.72(±0.09)	12.67(±0.03)	2.34(±0.05)	2.35(±0.05)	4.92(±0.10)	<0.1	4.89(±0.10)	15.6(±0.3)	14.0(±0.3)
Little Echo Pond	11/1/2022	4.53(±0.09)	20.22(±0.41)	2.70(±0.05)	5.88(±0.12)	11.45(±0.23)	0.71(±0.01)	3.70(±0.07)	-15.7(±0.3)	22.0(±0.4)
Lake Rondaxe	11/2/2022	6.58(±0.13)	4.83(±0.10)	1.65(±0.03)	0.98(±0.02)	12.23(±0.24)	3.34(±0.07)	21.54(±0.43)	88.8(±1.8)	18.6(±0.4)
Cascade Lake	11/2/2022	6.82(±0.14)	7.72(±0.20)	1.37(±0.03)	1.19(±0.02)	5.74(±0.11)	<0.1	19.35(±0.39)	127.4(±2.5)	17.9(±0.4)
Cascade Lake Outlet	11/2/2022	6.66(±0.13)	6.92(±0.09)	<0.5	0.95(±0.02)	8.07(±0.16)	3.07(±0.06)	23.08(±0.46)	145.8(±2.9)	22.4(±0.4)
Dart Lake	11/2/2022	6.34(±0.13)	5.53(±0.06)	1.08(±0.02)	0.44(±0.01)	11.69(±0.23)	4.55(±0.09)	18.55(±0.37)	74.2(±1.5)	14.8(±0.3)
Woods Lake	11/2/2022	6.28(±0.13)	5.69(±0.19)	1.27(±0.03)	2.09(±0.04)	6.24(±0.12)	2.38(±0.05)	14.94(±0.30)	20.0(±0.4)	12.4(±0.2)
Willys Lake	11/3/2022	5.48(±0.11)	3.68(±0.04)	0.57(±0.01)	1.60(±0.03)	5.41(±0.11)	7.47(±0.15)	15.63(±0.31)	0.2(±0.1)	9.4(±0.2)
Loon Hollow Pond	11/3/2022	5.12(±0.10)	7.37(±0.16)	0.74(±0.01)	1.94(±0.04)	7.00(±0.14)	3.17(±0.06)	9.95(±0.20)	9.9(±0.2)	9.9(±0.2)
Little Clear Pond	11/7/2022	6.11(±0.12)	6.96(±0.17)	1.30(±0.03)	0.85(±0.02)	4.92(±0.10)	<0.1	1.29(±0.03)	60.3(±1.2)	9.0(±0.2)
Black Pond	11/7/2022	6.95(±0.14)	3.73(±0.06)	<0.5	1.30(±0.03)	7.00(±0.14)	<0.1	25.96(±0.52)	272.9(±5.5)	34.6(±0.7)
Indian Lake	11/8/2022	5.83(±0.12)	7.49(±0.13)	0.81(±0.02)	2.56(±0.05)	7.00(±0.14)	1.27(±0.03)	12.43(±0.25)	46.6(±0.9)	10.8(±0.2)
Squaw Lake	11/8/2022	6.32(±0.13)	3.51(±0.04)	0.76(±0.02)	2.40(±0.05)	5.31(±0.11)	0.96(±0.02)	17.30(±0.35)	38.7(±0.8)	11.2(±0.2)
Queer Lake	11/9/2022	6.21(±0.12)	4.38(±0.06)	0.95(±0.02)	1.61(±0.03)	6.35(±0.13)	3.17(±0.06)	19.67(±0.39)	57.9(±1.2)	13.0(±0.3)
Big Hope Pond	11/10/2022	6.23(±0.12)	10.31(±0.20)	1.47(±0.03)	2.59(±0.05)	13.69(±0.27)	0.80(±0.02)	12.64(±0.25)	94.3(±1.9)	16.5(±0.3)
G Lake	11/10/2022	6.42(±0.13)	3.44(±0.03)	1.24(±0.02)	0.83(±0.02)	6.95(±0.14)	0.72(±0.01)	16.39(±0.33)	64.0(±1.3)	12.3(±0.2)
West Pond Outlet	11/14/2022	5.00(±0.10)	12.75(±0.20)	<0.5	2.03(±0.04)	19.77(±0.40)	0.51(±0.01)	8.81(±0.18)	11.2(±0.2)	15.3(±0.3)
West Pond	11/14/2022	5.55(±0.11)	7.45(±0.16)	0.95(±0.02)	1.12(±0.02)	4.97(±0.10)	0.59(±0.01)	10.41(±0.21)	15.7(±0.3)	9.4(±0.2)
Big Moose Lake	11/14/2022	6.38(±0.13)	5.58(±0.08)	0.97(±0.02)	2.25(±0.05)	10.82(±0.22)	4.61(±0.09)	17.79(±0.36)	53.2(±1.1)	14.7(±0.3)
Moss Lake	11/14/2022	6.62(±0.13)	5.10(±0.08)	2.08(±0.04)	1.62(±0.03)	16.30(±0.33)	2.04(±0.04)	24.89(±0.50)	138.1(±2.8)	23.5(±0.5)
Bubb Lake	11/14/2022	6.60(±0.13)	3.90(±0.08)	1.39(±0.03)	1.15(±0.02)	6.38(±0.13)	1.32(±0.03)	29.14(±0.58)	78.0(±1.6)	18.4(±0.4)
Bubb Lake Outlet	11/14/2022	6.45(±0.13)	4.23(±0.08)	<0.5	0.85(±0.02)	6.75(±0.14)	2.31(±0.05)	29.92(±0.60)	82.1(±1.6)	17.9(±0.4)
Limekiln Lake	11/15/2022	6.54(±0.13)	4.05(±0.01)	1.26(±0.03)	1.02(±0.02)	39.31(±0.79)	2.33(±0.05)	17.64(±0.35)	71.6(±1.4)	20.1(±0.4)
Raquette Lake Reservoir	11/15/2022	5.59(±0.11)	13.03(±0.19)	1.68(±0.03)	1.18(±0.02)	13.14(±0.26)	2.30(±0.05)	21.98(±0.44)	61.6(±1.2)	18.2(±0.4)
Sagamore Lake	11/15/2022	6.26(±0.13)	10.06(±0.14)	1.55(±0.03)	1.63(±0.03)	7.95(±0.16)	7.06(±0.14)	24.06(±0.48)	78.9(±1.6)	20.1(±0.4)
Arbutus Pond	11/15/2022	6.53(±0.13)	5.37(±0.08)	1.34(±0.03)	1.36(±0.03)	8.13(±0.16)	0.65(±0.01)	28.98(±0.58)	13.8(±0.3)	19.7(±0.4)
Clear Pond	11/15/2022	6.82(±0.14)	3.86(±0.05)	1.90(±0.04)	0.96(±0.02)	6.26(±0.13)	1.13(±0.02)	19.88(±0.40)	144.9(±2.9)	19.7(±0.4)
Heart Lake	11/15/2022	6.55(±0.13)	3.05(±0.06)	1.11(±0.02)	0.71(±0.01)	8.01(±0.16)	0.50(±0.01)	16.24(±0.32)	71.8(±1.4)	12.4(±0.2)
Grass Pond	11/17/2022	6.19(±0.12)	6.49(±0.10)	1.21(±0.02)	1.95(±0.04)	9.91(±0.20)	10.85(±0.22)	21.60(±0.43)	56.1(±1.1)	16.4(±0.3)
Middle Branch Lake	11/17/2022	6.63(±0.13)	5.56(±0.11)	1.60(±0.03)	2.90(±0.06)	7.00(±0.14)	0.95(±0.02)	16.86(±0.34)	102.5(±2.1)	16.1(±0.3)
Middle Settlement Lake	11/17/2022	6.12(±0.12)	5.19(±0.10)	1.17(±0.02)	2.62(±0.05)	7.06(±0.14)	1.51(±0.03)	13.99(±0.28)	45.5(±0.9)	10.3(±0.2)

Table S3. Water chemistry parameters of whole water samples from ALTM lakes (continued)

Site Name	Sampling Date	pH	DOC (mg C/L)	DIC (mg C/L)	[NH_4^+] (μM)	[Cl^-] (μM)	[NO_3^-] (μM)	[SO_4^{2-}] (μM)	ANC (μeq/L)	Specific Conductance (μS/cm)
South Lake	11/28/2022	6.30(±0.13)	3.98(±0.06)	0.96(±0.02)	2.06(±0.04)	7.01(±0.14)	6.30(±0.13)	16.10(±0.32)	21.9(±0.4)	12.2(±0.2)
North Lake	11/28/2022	6.01(±0.12)	8.13(±0.13)	0.94(±0.02)	2.30(±0.05)	7.94(±0.16)	6.36(±0.13)	17.05(±0.34)	44.8(±0.9)	14.2(±0.3)
Otter Lake Outlet	11/29/2022	5.98(±0.12)	4.01(±0.09)	<0.5	1.14(±0.02)	9.13(±0.18)	4.42(±0.09)	23.44(±0.47)	11.5(±0.2)	13.0(±0.3)
Otter Lake	11/29/2022	6.06(±0.12)	3.57(±0.02)	0.90(±0.02)	0.90(±0.02)	9.16(±0.18)	2.56(±0.05)	22.83(±0.46)	29.7(±0.6)	12.7(±0.3)
Jockeybush Lake	11/29/2022	6.02(±0.12)	4.04(±0.03)	1.00(±0.02)	0.50(±0.01)	7.99(±0.16)	2.92(±0.06)	15.22(±0.30)	49.8(±1.0)	10.4(±0.2)
Avalanche Lake	6/2/2023	5.46(±0.11)	5.79(±0.13)	0.62(±0.01)	1.52(±0.03)	4.26(±0.09)	15.36(±0.31)	7.93(±0.16)	23.2(±0.5)	9.2(±0.2)
Lake Colden	6/2/2023	5.46(±0.11)	5.22(±0.10)	0.52(±0.01)	0.90(±0.02)	3.59(±0.07)	13.21(±0.26)	10.32(±0.21)	17.9(±0.4)	9.6(±0.2)
Brook Trout Lake	5/22/2023	6.03(±0.12)	3.72(±0.05)	0.34(±0.01)	1.19(±0.02)	5.90(±0.12)	3.16(±0.06)	14.82(±0.30)	9.2(±0.2)	8.6(±0.2)
Squash Pond Outlet	5/15/2023	4.64(±0.09)	8.91(±0.18)	<0.5	0.85(±0.02)	6.01(±0.12)	8.41(±0.17)	12.38(±0.25)	-13.9(±0.3)	17.6(±0.4)
Squash Pond	5/15/2023	4.83(±0.10)	10.85(±0.26)	1.06(±0.02)	0.84(±0.02)	5.38(±0.11)	2.91(±0.06)	10.77(±0.22)	-11.1(±0.2)	13.6(±0.3)
Constable Pond Outlet	5/15/2023	5.45(±0.11)	7.02(±0.16)	<0.5	1.40(±0.03)	5.09(±0.10)	4.42(±0.09)	15.53(±0.31)	10.5(±0.2)	12.1(±0.2)
Constable Pond	5/15/2023	5.20(±0.10)	7.38(±0.08)	0.66(±0.01)	0.76(±0.02)	4.90(±0.10)	5.18(±0.10)	17.73(±0.35)	20.3(±0.4)	12.4(±0.2)
East Copperas Pond	6/27/2023	4.66(±0.09)	13.00(±0.32)	0.65(±0.01)	1.48(±0.03)	3.16(±0.06)	<0.1	3.80(±0.08)	-2.8(±0.1)	14.2(±0.3)
Little Echo Pond	6/29/2023	4.53(±0.09)	15.86(±0.21)	1.07(±0.02)	1.48(±0.03)	10.86(±0.22)	<0.1	3.39(±0.07)	-19.4(±0.4)	17.6(±0.4)
Lake Rondaxe	6/19/2023	6.61(±0.13)	5.98(±0.11)	1.13(±0.02)	1.17(±0.02)	12.38(±0.25)	5.00(±0.10)	20.66(±0.41)	87.2(±1.7)	17.1(±0.3)
Cascade Lake	6/16/2023	6.67(±0.13)	4.90(±0.03)	1.27(±0.03)	0.50(±0.01)	6.40(±0.13)	7.63(±0.15)	21.00(±0.42)	65.8(±1.3)	16.3(±0.3)
Cascade Lake Outlet	6/16/2023	6.44(±0.13)	7.76(±0.16)	<0.5	0.99(±0.02)	6.49(±0.13)	8.36(±0.17)	19.04(±0.38)	91.7(±1.8)	16.5(±0.3)
Dart Lake	5/23/2023	6.19(±0.12)	6.79(±0.05)	0.76(±0.02)	1.56(±0.03)	13.05(±0.26)	8.81(±0.18)	18.00(±0.36)	48.1(±1.0)	13.9(±0.3)
Woods Lake	6/16/2023	6.23(±0.12)	5.42(±0.05)	1.03(±0.02)	1.31(±0.03)	5.80(±0.12)	11.38(±0.23)	13.24(±0.26)	53.9(±1.1)	12.3(±0.2)
Willys Lake	5/23/2023	5.38(±0.11)	3.57(±0.06)	0.30(±0.01)	1.08(±0.02)	6.12(±0.12)	18.05(±0.36)	14.26(±0.29)	12.4(±0.2)	9.9(±0.2)
Loon Hollow Pond	5/23/2023	5.18(±0.10)	6.39(±0.08)	0.39(±0.01)	0.90(±0.02)	9.34(±0.19)	4.53(±0.09)	9.15(±0.18)	1.2(±0.1)	8.9(±0.2)
Little Clear Pond	6/22/2023	6.11(±0.12)	7.64(±0.06)	0.67(±0.01)	0.59(±0.01)	4.32(±0.09)	<0.1	0.84(±0.02)	46.7(±0.9)	5.8(±0.1)
Black Pond	6/29/2023	6.94(±0.14)	4.63(±0.07)	<0.5	1.03(±0.02)	6.54(±0.13)	1.79(±0.04)	24.76(±0.50)	208.1(±4.2)	28.4(±0.6)
Indian Lake	5/22/2023	5.34(±0.11)	6.60(±0.16)	0.52(±0.01)	0.94(±0.02)	4.08(±0.08)	<0.1	11.89(±0.24)	31.1(±0.6)	10.6(±0.2)
Squaw Lake	5/22/2023	6.34(±0.13)	3.75(±0.08)	0.55(±0.01)	0.42(±0.01)	5.00(±0.10)	2.38(±0.05)	15.81(±0.32)	44.9(±0.9)	10.6(±0.2)
Queer Lake	6/6/2023	6.15(±0.12)	4.79(±0.10)	0.68(±0.01)	1.25(±0.03)	6.01(±0.12)	6.10(±0.12)	19.25(±0.38)	41.3(±0.8)	11.6(±0.2)
Big Hope Pond	6/23/2023	6.42(±0.13)	10.91(±0.17)	0.99(±0.02)	2.79(±0.06)	12.57(±0.25)	1.10(±0.02)	12.03(±0.24)	73.9(±1.5)	15.5(±0.3)
G Lake	6/29/2023	6.35(±0.13)	3.79(±0.02)	0.68(±0.01)	0.92(±0.02)	5.98(±0.12)	3.97(±0.08)	14.96(±0.30)	41.6(±0.8)	10.4(±0.2)
West Pond Outlet	6/16/2023	4.99(±0.10)	15.78(±0.38)	<0.5	3.81(±0.08)	10.44(±0.21)	4.08(±0.08)	5.60(±0.11)	16.3(±0.3)	14.1(±0.3)
West Pond	6/16/2023	5.65(±0.11)	7.47(±0.09)	0.86(±0.02)	0.58(±0.01)	6.32(±0.13)	1.41(±0.03)	9.59(±0.19)	13.1(±0.3)	7.5(±0.1)
Big Moose Lake	6/16/2023	6.15(±0.12)	6.40(±0.10)	0.78(±0.02)	1.25(±0.03)	10.50(±0.21)	7.72(±0.15)	17.09(±0.34)	57.6(±1.2)	13.3(±0.3)
Moss Lake	5/25/2023	6.77(±0.14)	5.13(±0.09)	1.46(±0.03)	0.76(±0.02)	16.11(±0.32)	7.66(±0.15)	23.75(±0.48)	103.1(±2.1)	20.1(±0.4)
Bubb Lake	5/25/2023	6.60(±0.13)	3.35(±0.04)	1.19(±0.02)	1.21(±0.02)	6.38(±0.13)	5.86(±0.12)	24.91(±0.50)	62.4(±1.2)	15.9(±0.3)
Bubb Lake Outlet	5/25/2023	6.49(±0.13)	3.28(±0.06)	<0.5	1.45(±0.03)	6.35(±0.13)	7.92(±0.16)	24.26(±0.49)	93.6(±1.9)	16.3(±0.3)
Limekiln Lake	6/6/2023	6.55(±0.13)	6.38(±0.16)	1.32(±0.03)	0.75(±0.01)	38.19(±0.76)	1.08(±0.02)	16.67(±0.33)	93.7(±1.9)	18.8(±0.4)
Raquette Lake Reservoir	5/23/2023	5.43(±0.11)	8.67(±0.20)	1.45(±0.03)	0.68(±0.01)	4.22(±0.08)	0.97(±0.02)	18.29(±0.37)	36.7(±0.7)	14.2(±0.3)
Sagamore Lake	6/20/2023	5.53(±0.11)	9.19(±0.22)	1.42(±0.03)	1.43(±0.03)	4.87(±0.10)	4.80(±0.10)	17.35(±0.35)	35.3(±0.7)	15.7(±0.3)
Arbutus Pond	6/23/2023	6.44(±0.13)	6.07(±0.12)	1.23(±0.02)	1.44(±0.03)	6.52(±0.13)	3.32(±0.07)	21.20(±0.42)	75.0(±1.5)	16.4(±0.3)

Table S3. Water chemistry parameters of whole water samples from ALTM lakes (continued)

Site Name	Sampling Date	pH	DOC (mg C/L)	DIC (mg C/L)	$[NH_4^+]$ (μM)	$[Cl^-]$ (μM)	$[NO_3^-]$ (μM)	$[SO_4^{2-}]$ (μM)	ANC (μeq/L)	Specific Conductance (μS/cm)
Clear Pond	6/23/2023	6.85(±0.14)	4.50(±0.05)	1.57(±0.03)	1.25(±0.02)	5.72(±0.11)	0.98(±0.02)	17.91(±0.36)	116.6(±2.3)	17.3(±0.3)
Heart Lake	6/13/2023	6.59(±0.13)	3.04(±0.04)	0.80(±0.02)	0.84(±0.02)	7.34(±0.15)	1.58(±0.03)	15.39(±0.31)	54.8(±1.1)	11.0(±0.2)
Grass Pond	5/16/2023	6.17(±0.12)	4.40(±0.11)	1.28(±0.03)	0.83(±0.02)	5.90(±0.12)	8.13(±0.16)	21.92(±0.44)	62.3(±1.2)	14.7(±0.3)
Middle Branch Lake	5/16/2023	6.64(±0.13)	4.50(±0.05)	1.33(±0.03)	1.32(±0.03)	5.85(±0.12)	3.34(±0.07)	20.13(±0.40)	89.9(±1.8)	16.0(±0.3)
Middle Settlement Lake	5/16/2023	6.30(±0.13)	4.32(±0.11)	0.97(±0.02)	4.82(±0.10)	6.17(±0.12)	4.82(±0.10)	21.52(±0.43)	51.8(±1.0)	13.8(±0.3)
South Lake	6/26/2023	6.22(±0.12)	3.96(±0.04)	0.74(±0.01)	1.47(±0.03)	5.86(±0.12)	10.59(±0.21)	16.07(±0.32)	39.1(±0.8)	11.3(±0.2)
North Lake	6/26/2023	5.73(±0.11)	5.90(±0.12)	1.03(±0.02)	0.55(±0.01)	4.96(±0.10)	6.95(±0.14)	15.57(±0.31)	40.5(±0.8)	11.9(±0.2)
Otter Lake Outlet	6/5/2023	5.92(±0.12)	3.35(±0.08)	<0.5	0.77(±0.02)	7.10(±0.14)	5.38(±0.11)	21.37(±0.43)	37.9(±0.8)	11.3(±0.2)
Otter Lake	6/5/2023	5.87(±0.12)	3.80(±0.04)	0.56(±0.01)	0.45(±0.01)	6.90(±0.14)	5.47(±0.11)	19.54(±0.39)	24.8(±0.5)	10.5(±0.2)
Jockeybush Lake	6/5/2023	5.92(±0.12)	3.32(±0.06)	0.75(±0.01)	0.69(±0.01)	6.61(±0.13)	3.72(±0.07)	13.76(±0.28)	39.8(±0.8)	7.9(±0.2)
Avalanche Lake	9/25/2023	5.86(±0.12)	7.73(±0.20)	0.78(±0.02)	4.69(±0.09)	4.72(±0.09)	7.84(±0.16)	8.91(±0.18)	19.6(±0.4)	9.7(±0.2)
Lake Colden	9/25/2023	5.99(±0.12)	6.34(±0.17)	0.58(±0.01)	2.62(±0.05)	4.40(±0.09)	3.94(±0.08)	11.89(±0.24)	48.4(±1.0)	7.9(±0.2)
Brook Trout Lake	9/5/2023	6.05(±0.12)	4.59(±0.11)	0.37(±0.01)	0.56(±0.01)	6.56(±0.13)	<0.1	14.53(±0.29)	42.7(±0.9)	7.6(±0.2)
Squash Pond Outlet	9/18/2023	4.83(±0.10)	9.39(±0.23)	<0.5	1.63(±0.03)	6.27(±0.13)	7.94(±0.16)	10.97(±0.22)	11.6(±0.2)	13.5(±0.3)
Squash Pond	9/18/2023	4.86(±0.10)	13.08(±0.39)	0.97(±0.02)	0.82(±0.02)	8.93(±0.18)	0.23(±0.01)	8.58(±0.17)	0.7(±0.1)	12.8(±0.3)
Constable Pond Outlet	9/18/2023	6.04(±0.12)	8.85(±0.30)	<0.5	1.18(±0.02)	6.53(±0.13)	0.91(±0.02)	13.36(±0.27)	45.4(±0.9)	11.4(±0.2)
Constable Pond	9/18/2023	5.44(±0.11)	8.71(±0.29)	0.63(±0.01)	1.17(±0.02)	5.66(±0.11)	<0.1	15.47(±0.31)	11.7(±0.2)	11.1(±0.2)
East Copperas Pond	9/15/2023	4.60(±0.09)	12.13(±0.54)	1.33(±0.03)	1.34(±0.03)	3.53(±0.07)	<0.1	3.58(±0.07)	-9.4(±0.2)	15.4(±0.3)
Little Echo Pond	9/21/2023	4.37(±0.09)	17.96(±0.83)	2.14(±0.04)	1.72(±0.03)	12.07(±0.24)	<0.1	2.46(±0.05)	-44.9(±0.9)	23.9(±0.5)
Lake Rondaxe	9/23/2023	6.56(±0.13)	5.57(±0.11)	1.14(±0.02)	1.17(±0.02)	11.73(±0.23)	1.51(±0.03)	17.72(±0.35)	85.1(±1.7)	16.1(±0.3)
Cascade Lake	9/18/2023	6.69(±0.13)	4.85(±0.05)	1.70(±0.03)	1.34(±0.03)	6.32(±0.13)	<0.1	18.33(±0.37)	129.5(±2.6)	18.3(±0.4)
Cascade Lake Outlet	9/18/2023	6.82(±0.14)	5.37(±0.11)	<0.5	1.06(±0.02)	10.56(±0.21)	3.10(±0.06)	19.60(±0.39)	184(±3.7)	24.8(±0.5)
Dart Lake	9/22/2023	6.33(±0.13)	6.12(±0.17)	0.60(±0.01)	0.50(±0.01)	11.40(±0.23)	1.58(±0.03)	15.88(±0.32)	44.0(±0.9)	13.2(±0.3)
Woods Lake	9/18/2023	6.08(±0.12)	7.72(±0.23)	1.14(±0.02)	1.24(±0.02)	5.12(±0.10)	0.43(±0.01)	10.53(±0.21)	43.7(±0.9)	11.0(±0.2)
Willys Lake	9/19/2023	5.35(±0.11)	4.07(±0.10)	0.41(±0.01)	1.07(±0.02)	5.21(±0.10)	10.99(±0.22)	13.48(±0.27)	29.5(±0.6)	9.5(±0.2)
Loon Hollow Pond	9/19/2023	4.93(±0.10)	8.53(±0.23)	0.65(±0.01)	0.31(±0.01)	6.67(±0.13)	<0.1	7.81(±0.16)	-5.2(±-0.1)	10.5(±0.2)
Little Clear Pond	9/12/2023	5.87(±0.12)	6.68(±0.06)	1.37(±0.03)	1.00(±0.02)	4.04(±0.08)	<0.1	0.36(±0.01)	35.8(±0.7)	6.2(±0.1)
Black Pond	9/21/2023	6.89(±0.14)	4.94(±0.07)	<0.5	1.33(±0.03)	6.29(±0.13)	<0.1	24.38(±0.49)	251.3(±5.0)	29.0(±0.6)
Indian Lake	9/5/2023	5.39(±0.11)	9.56(±0.26)	0.60(±0.01)	1.92(±0.04)	4.62(±0.09)	<0.1	9.33(±0.19)	30.8(±0.6)	10.0(±0.2)
Squaw Lake	9/5/2023	6.43(±0.13)	4.12(±0.06)	0.53(±0.01)	1.39(±0.03)	5.73(±0.11)	0.43(±0.01)	14.90(±0.30)	29.6(±0.6)	10.6(±0.2)
Queer Lake	9/13/2023	6.30(±0.13)	4.35(±0.07)	0.43(±0.01)	0.80(±0.02)	5.88(±0.12)	1.08(±0.02)	17.91(±0.36)	35.1(±0.7)	11.3(±0.2)
Big Hope Pond	9/21/2023	6.37(±0.13)	9.52(±0.22)	1.32(±0.03)	1.05(±0.02)	12.48(±0.25)	<0.1	10.56(±0.21)	67.5(±1.4)	15.8(±0.3)
G Lake	9/12/2023	6.45(±0.13)	3.72(±0.07)	0.69(±0.01)	1.00(±0.02)	5.83(±0.12)	0.32(±0.01)	14.25(±0.28)	67.2(±1.3)	10.8(±0.2)
West Pond Outlet	9/4/2023	4.70(±0.09)	20.73(±0.85)	<0.5	1.23(±0.02)	12.21(±0.24)	<0.1	1.81(±0.04)	18.8(±0.4)	17.6(±0.4)
West Pond	9/4/2023	5.51(±0.11)	8.02(±0.19)	0.78(±0.02)	0.45(±0.01)	2.84(±0.06)	<0.1	6.95(±0.14)	25.9(±0.5)	7.0(±0.1)
Big Moose Lake	9/4/2023	6.20(±0.12)	7.16(±0.14)	0.60(±0.01)	1.09(±0.02)	10.25(±0.20)	1.30(±0.03)	15.51(±0.31)	40.3(±0.8)	12.7(±0.3)
Moss Lake	9/26/2023	6.92(±0.14)	4.75(±0.07)	1.52(±0.03)	0.82(±0.02)	16.50(±0.33)	<0.1	22.31(±0.45)	122.8(±2.5)	22.1(±0.4)
Bubb Lake	9/4/2023	6.72(±0.13)	3.95(±0.08)	1.07(±0.02)	1.00(±0.02)	7.42(±0.15)	0.18(±0.01)	26.52(±0.53)	85.9(±1.7)	16.3(±0.3)

**Table S3.** Water chemistry parameters of whole water samples from ALTM lakes (continued)										
Site Name	Sampling Date	pH	DOC (mg C/L)	DIC (mg C/L)	$[NH_4^+]$ (µM)	$[Cl^-]$ (µM)	$[NO_3^-]$ (µM)	$[SO_4^{2-}]$ (µM)	ANC (µeq/L)	Specific Conductance (µS/cm)
Bubb Lake Outlet	9/4/2023	6.68(±0.13)	3.54(±0.06)	<0.5	1.21(±0.02)	8.30(±0.17)	3.56(±0.07)	25.58(±0.51)	106.9(±2.1)	18.1(±0.4)
Limekiln Lake	9/4/2023	6.59(±0.13)	4.67(±0.10)	1.39(±0.03)	1.59(±0.03)	40.29(±0.81)	0.51(±0.01)	15.84(±0.32)	82.3(±1.6)	18.6(±0.4)
Raquette Lake Reservoir	9/26/2023	6.47(±0.13)	13.24(±0.33)	3.15(±0.06)	1.62(±0.03)	12.80(±0.26)	0.45(±0.01)	15.51(±0.31)	139.6(±2.8)	23.0(±0.5)
Sagamore Lake	9/29/2023	6.18(±0.12)	13.03(±0.43)	1.11(±0.02)	1.24(±0.02)	6.52(±0.13)	1.45(±0.03)	19.05(±0.38)	74.9(±1.5)	17.8(±0.4)
Arbutus Pond	9/26/2023	6.47(±0.13)	7.70(±0.18)	1.37(±0.03)	1.83(±0.04)	6.48(±0.13)	0.46(±0.01)	20.62(±0.41)	110.6(±2.2)	17.3(±0.3)
Clear Pond	9/26/2023	6.80(±0.14)	5.18(±0.09)	1.35(±0.03)	1.67(±0.03)	5.26(±0.11)	<0.1	15.64(±0.31)	128.7(±2.6)	17.4(±0.3)
Heart Lake	9/5/2023	6.50(±0.13)	2.89(±0.03)	0.98(±0.02)	1.23(±0.02)	7.37(±0.15)	<0.1	15.33(±0.31)	61.6(±1.2)	11.3(±0.2)
Grass Pond	9/4/2023	6.04(±0.12)	7.26(±0.12)	1.10(±0.02)	0.61(±0.01)	5.67(±0.11)	1.04(±0.02)	18.73(±0.37)	35.9(±0.7)	12.4(±0.2)
Middle Branch Lake	9/4/2023	6.47(±0.13)	6.46(±0.05)	1.54(±0.03)	0.92(±0.02)	6.09(±0.12)	<0.1	17.73(±0.35)	80.4(±1.6)	16.4(±0.3)
Middle Settlement Lake	9/4/2023	6.42(±0.13)	4.93(±0.10)	1.21(±0.02)	9.94(±0.20)	6.82(±0.14)	5.09(±0.10)	44.38(±0.89)	88.3(±1.8)	21.4(±0.4)
South Lake	9/6/2023	6.28(±0.13)	4.25(±0.02)	0.64(±0.01)	0.77(±0.02)	6.16(±0.12)	4.26(±0.09)	14.97(±0.30)	55.8(±1.1)	11.7(±0.2)
North Lake	9/6/2023	6.01(±0.12)	8.68(±0.22)	0.64(±0.01)	0.68(±0.01)	5.77(±0.12)	<0.1	13.66(±0.27)	22.6(±0.5)	11.9(±0.2)
Otter Lake Outlet	9/14/2023	6.22(±0.12)	3.86(±0.04)	<0.5	0.94(±0.02)	6.15(±0.12)	0.78(±0.02)	20.78(±0.42)	51.9(±1.0)	13.0(±0.3)
Otter Lake	9/14/2023	6.05(±0.12)	3.86(±0.14)	0.85(±0.02)	0.75(±0.02)	6.22(±0.12)	0.20(±0.01)	18.41(±0.37)	49.2(±1.0)	10.7(±0.2)
Jockeybush Lake	9/12/2023	6.01(±0.12)	4.57(±0.06)	0.70(±0.01)	0.74(±0.01)	5.11(±0.10)	<0.1	11.11(±0.22)	18.2(±0.4)	7.1(±0.1)

**Table S3.** Water chemistry parameters of whole water samples from ALTM lakes (continued)											
Site Name	Sampling Date	$[Na^+]$ (μM)	$[Mg^{2+}]$ (μM)	$[K^+]$ (μM)	$[Ca^{2+}]$ (μM)	[Al] (μM)	$[Al]_{Total Monomeric}$ (μM)	[Mn] (μM)	[Fe] (μM)	[Cu] (μM)	[Zn] (μM)
Avalanche Lake	10/17/2022	20.50(±0.41)	5.81(±0.12)	1.48(±0.03)	25.98(±0.52)	15.50(±0.31)	5.47(±0.11)	0.12(±0.01)	1.49(±0.20)	0.08(±0.01)	0.11(±0.02)
Lake Colden	10/17/2022	22.82(±0.46)	5.86(±0.12)	1.42(±0.03)	25.76(±0.52)	16.87(±0.34)	5.59(±0.11)	0.10(±0.01)	1.30(±0.19)	0.10(±0.01)	0.14(±0.01)
Brook Trout Lake	10/26/2022	20.92(±0.42)	8.74(±0.17)	3.66(±0.07)	21.40(±0.43)	3.17(±0.06)	0.60(±0.01)	0.36(±0.01)	9.04(±1.00)	0.13(±0.01)	0.23(±0.02)
Squash Pond Outlet	10/31/2022	25.93(±0.52)	5.40(±0.11)	3.53(±0.07)	15.09(±0.30)	11.59(±0.23)	5.47(±0.11)	0.23(±0.02)	3.76(±0.02)	0.09(±0.01)	0.20(±0.01)
Squash Pond	10/31/2022	23.63(±0.47)	4.45(±0.09)	2.75(±0.06)	13.45(±0.27)	13.96(±0.28)	8.26(±0.17)	0.27(±0.02)	3.60(±0.32)	0.12(±0.01)	0.22(±0.03)
Constable Pond Outlet	10/31/2022	31.87(±0.64)	9.58(±0.19)	5.97(±0.12)	31.91(±0.64)	5.28(±0.11)	1.88(±0.04)	0.32(±0.01)	5.37(±0.34)	0.07(±0.01)	0.09(±0.01)
Constable Pond	10/31/2022	27.75(±0.55)	7.26(±0.15)	5.12(±0.10)	25.32(±0.51)	7.61(±0.15)	2.76(±0.06)	0.21(±0.01)	5.73(±0.19)	0.08(±0.01)	0.12(±0.01)
East Copperas Pond	11/1/2022	6.51(±0.13)	5.05(±0.10)	5.76(±0.12)	17.66(±0.35)	2.63(±0.05)	1.85(±0.04)	0.43(±0.03)	5.21(±0.48)	0.15(±0.01)	0.14(±0.01)
Little Echo Pond	11/1/2022	14.28(±0.29)	12.49(±0.25)	6.17(±0.12)	21.07(±0.42)	3.14(±0.06)	1.97(±0.04)	0.63(±0.02)	15.4(±0.72)	0.18(±0.01)	0.16(±0.01)
Lake Rondaxe	11/2/2022	44.30(±0.89)	17.19(±0.34)	7.81(±0.16)	53.64(±1.07)	2.30(±0.05)	0.41(±0.01)	0.09(±0.01)	2.99(±0.09)	0.10(±0.01)	0.10(±0.01)
Cascade Lake	11/2/2022	39.64(±0.79)	17.17(±0.34)	7.68(±0.15)	57.33(±1.15)	0.25(±0.01)	0.17(±0.01)	0.24(±0.01)	3.03(±0.15)	0.14(±0.01)	0.17(±0.02)
Cascade Lake Outlet	11/2/2022	52.67(±1.05)	21.49(±0.43)	10.44(±0.21)	70.86(±1.42)	2.51(±0.05)	0.55(±0.01)	0.26(±0.01)	2.72(±0.17)	0.07(±0.01)	0.07(±0.01)
Dart Lake	11/2/2022	34.50(±0.69)	10.59(±0.21)	5.95(±0.12)	42.31(±0.85)	2.88(±0.06)	0.65(±0.01)	0.15(±0.01)	1.03(±0.15)	0.08(±0.01)	0.22(±0.01)
Woods Lake	11/2/2022	20.29(±0.41)	7.14(±0.14)	4.20(±0.08)	40.00(±0.80)	1.60(±0.03)	0.44(±0.01)	0.10(±0.01)	1.95(±0.21)	0.08(±0.01)	0.20(±0.01)
Willys Lake	11/3/2022	18.83(±0.38)	4.37(±0.09)	3.72(±0.07)	15.11(±0.30)	3.15(±0.06)	1.60(±0.03)	0.10(±0.01)	0.58(±0.09)	0.06(±0.01)	0.21(±0.02)
Loon Hollow Pond	11/3/2022	18.21(±0.36)	3.38(±0.07)	5.11(±0.10)	11.51(±0.23)	11.25(±0.22)	5.12(±0.10)	0.22(±0.01)	1.58(±0.21)	0.10(±0.01)	0.15(±0.02)
Little Clear Pond	11/7/2022	4.99(±0.10)	10.01(±0.20)	8.85(±0.18)	25.87(±0.52)	0.62(±0.01)	0.27(±0.01)	0.57(±0.02)	13.51(±0.99)	0.15(±0.01)	0.13(±0.01)
Black Pond	11/7/2022	49.98(±1.00)	18.21(±0.36)	8.94(±0.18)	102.46(±2.05)	0.44(±0.01)	0.13(±0.01)	0.04(±0.01)	1.89(±0.16)	0.06(±0.01)	0.05(±0.01)
Indian Lake	11/8/2022	22.08(±0.44)	9.87(±0.20)	2.69(±0.05)	24.18(±0.48)	7.97(±0.16)	2.35(±0.05)	0.14(±0.01)	2.36(±0.06)	0.09(±0.01)	0.14(±0.01)
Squaw Lake	11/8/2022	19.55(±0.39)	12.20(±0.24)	3.09(±0.06)	28.68(±0.57)	0.42(±0.01)	0.03(±0.01)	0.05(±0.01)	0.46(±0.07)	0.07(±0.01)	0.06(±0.01)
Queer Lake	11/9/2022	20.94(±0.42)	9.37(±0.19)	4.97(±0.10)	33.71(±0.67)	1.84(±0.04)	0.40(±0.01)	0.08(±0.01)	0.94(±0.25)	0.06(±0.01)	0.09(±0.01)
Big Hope Pond	11/10/2022	32.54(±0.65)	20.14(±0.40)	7.69(±0.15)	46.60(±0.93)	3.59(±0.07)	1.04(±0.02)	0.18(±0.01)	1.61(±0.25)	0.14(±0.02)	0.22(±0.01)
G Lake	11/10/2022	29.66(±0.59)	13.57(±0.27)	2.75(±0.06)	28.72(±0.57)	1.77(±0.04)	0.13(±0.01)	0.34(±0.01)	0.84(±0.10)	0.07(±0.01)	0.07(±0.01)
West Pond Outlet	11/14/2022	29.43(±0.59)	7.67(±0.15)	6.61(±0.13)	23.31(±0.47)	7.74(±0.15)	3.87(±0.08)	0.48(±0.03)	5.72(±0.28)	0.17(±0.01)	0.31(±0.03)
West Pond	11/14/2022	23.76(±0.48)	6.27(±0.13)	2.59(±0.05)	17.87(±0.36)	4.77(±0.10)	1.79(±0.04)	0.44(±0.01)	5.36(±0.33)	0.14(±0.01)	0.10(±0.01)
Big Moose Lake	11/14/2022	31.92(±0.64)	9.77(±0.20)	5.87(±0.12)	40.93(±0.82)	4.41(±0.09)	0.80(±0.02)	0.24(±0.01)	1.90(±0.10)	0.09(±0.01)	0.17(±0.02)
Moss Lake	11/14/2022	50.35(±1.01)	20.68(±0.41)	9.26(±0.19)	65.81(±1.32)	1.20(±0.02)	0.29(±0.01)	0.06(±0.01)	1.41(±0.07)	0.07(±0.01)	0.12(±0.02)
Bubb Lake	11/14/2022	39.15(±0.78)	17.25(±0.34)	6.94(±0.14)	46.18(±0.92)	0.66(±0.01)	0.08(±0.01)	0.04(±0.01)	0.66(±0.19)	0.07(±0.01)	0.08(±0.01)
Bubb Lake Outlet	11/14/2022	39.62(±0.79)	16.97(±0.34)	7.48(±0.15)	44.49(±0.89)	1.41(±0.03)	0.29(±0.01)	0.08(±0.01)	0.86(±0.05)	0.11(±0.01)	0.17(±0.01)
Limekiln Lake	11/15/2022	55.75(±1.11)	13.32(±0.27)	5.55(±0.11)	51.09(±1.02)	0.53(±0.01)	0.07(±0.01)	0.06(±0.01)	0.63(±0.09)	0.06(±0.01)	0.07(±0.01)
Raquette Lake Reservoir	11/15/2022	35.62(±0.71)	16.21(±0.32)	13.1(±0.26)	43.03(±0.86)	13.14(±0.26)	5.25(±0.11)	0.39(±0.01)	7.19(±0.54)	0.16(±0.01)	0.27(±0.02)
Sagamore Lake	11/15/2022	42.62(±0.85)	22.58(±0.45)	6.41(±0.13)	57.03(±1.14)	8.73(±0.17)	2.36(±0.05)	0.53(±0.01)	9.78(±0.41)	0.13(±0.01)	0.27(±0.03)
Arbutus Pond	11/15/2022	34.97(±0.70)	16.95(±0.34)	6.17(±0.12)	62.35(±1.25)	1.21(±0.02)	0.27(±0.01)	0.27(±0.02)	2.93(±0.08)	0.09(±0.01)	0.10(±0.01)
Clear Pond	11/15/2022	43.81(±0.88)	13.49(±0.27)	2.29(±0.05)	72.01(±1.44)	1.77(±0.04)	0.29(±0.01)	0.07(±0.01)	0.36(±0.05)	0.09(±0.01)	0.11(±0.01)
Heart Lake	11/15/2022	27.05(±0.54)	9.45(±0.19)	2.23(±0.04)	37.76(±0.76)	1.30(±0.03)	0.01(±0.01)	0.04(±0.01)	0.57(±0.05)	0.06(±0.01)	0.17(±0.02)
Grass Pond	11/17/2022	43.86(±0.88)	13.20(±0.26)	10.64(±0.21)	38.54(±0.77)	9.88(±0.20)	2.29(±0.05)	0.16(±0.01)	1.65(±0.14)	0.10(±0.01)	0.19(±0.02)
Middle Branch Lake	11/17/2022	44.24(±0.88)	15.33(±0.31)	9.14(±0.18)	45.06(±0.90)	2.98(±0.06)	0.53(±0.01)	0.30(±0.01)	1.05(±0.07)	0.10(±0.01)	0.15(±0.01)
Middle Settlement Lake	11/17/2022	31.05(±0.62)	7.69(±0.15)	7.29(±0.15)	22.75(±0.46)	3.31(±0.07)	0.75(±0.01)	0.08(±0.01)	0.90(±0.03)	0.11(±0.01)	0.25(±0.03)

**Table S3.** Water chemistry parameters of whole water samples from ALTM lakes (continued)											
Site Name	Sampling Date	$[Na^+]$ (μM)	$[Mg^{2+}]$ (μM)	$[K^+]$ (μM)	$[Ca^{2+}]$ (μM)	[Al] (μM)	$[Al]_{Total\ Monomeric}$ (μM)	[Mn] (μM)	[Fe] (μM)	[Cu] (μM)	[Zn] (μM)
South Lake	11/28/2022	25.89(±0.52)	10.02(±0.20)	5.02(±0.10)	29.44(±0.59)	3.63(±0.07)	0.45(±0.01)	0.09(±0.01)	1.31(±0.10)	0.09(±0.01)	0.09(±0.01)
North Lake	11/28/2022	30.63(±0.61)	13.15(±0.26)	5.86(±0.12)	35.63(±0.71)	11.53(±0.23)	2.55(±0.05)	0.21(±0.01)	5.39(±0.27)	0.13(±0.01)	0.17(±0.01)
Otter Lake Outlet	11/29/2022	30.50(±0.61)	10.90(±0.22)	5.74(±0.11)	24.28(±0.49)	3.99(±0.08)	0.97(±0.02)	0.09(±0.01)	0.42(±0.01)	0.05(±0.01)	0.08(±0.01)
Otter Lake	11/29/2022	30.19(±0.60)	10.83(±0.22)	5.37(±0.11)	24.12(±0.48)	4.37(±0.09)	0.63(±0.01)	0.04(±0.01)	0.65(±0.07)	0.09(±0.01)	0.10(±0.01)
Jockeybush Lake	11/29/2022	22.36(±0.45)	9.19(±0.18)	4.10(±0.08)	22.85(±0.46)	4.55(±0.09)	1.09(±0.02)	0.09(±0.01)	0.78(±0.04)	0.06(±0.01)	0.09(±0.01)
Avalanche Lake	6/2/2023	17.54(±0.35)	5.85(±0.12)	4.94(±0.10)	27.27(±0.55)	15.78(±0.32)	4.79(±0.10)	0.12(±0.01)	2.49(±0.19)	0.02(±0.01)	0.15(±0.01)
Lake Colden	6/2/2023	18.64(±0.37)	5.88(±0.12)	4.89(±0.10)	23.01(±0.46)	15.58(±0.31)	4.42(±0.09)	0.08(±0.01)	1.98(±0.11)	0.03(±0.01)	0.09(±0.01)
Brook Trout Lake	5/22/2023	20.13(±0.40)	8.02(±0.16)	3.20(±0.06)	19.58(±0.39)	4.16(±0.08)	0.84(±0.02)	0.16(±0.01)	11.30(±1.05)	0.04(±0.01)	0.11(±0.02)
Squash Pond Outlet	5/15/2023	19.88(±0.40)	5.20(±0.10)	4.89(±0.10)	17.46(±0.35)	15.69(±0.31)	7.92(±0.16)	0.16(±0.01)	4.39(±0.12)	0.05(±0.01)	0.11(±0.01)
Squash Pond	5/15/2023	19.98(±0.40)	5.07(±0.10)	4.92(±0.10)	16.29(±0.33)	14.25(±0.28)	7.39(±0.15)	0.09(±0.01)	4.11(±0.14)	0.06(±0.01)	0.33(±0.01)
Constable Pond Outlet	5/15/2023	21.65(±0.43)	6.98(±0.14)	5.09(±0.10)	24.93(±0.50)	9.33(±0.19)	4.16(±0.08)	0.14(±0.01)	6.42(±0.14)	0.06(±0.01)	0.20(±0.01)
Constable Pond	5/15/2023	20.19(±0.40)	6.10(±0.12)	5.04(±0.10)	21.85(±0.44)	9.76(±0.20)	4.30(±0.09)	0.10(±0.01)	6.65(±0.10)	0.06(±0.01)	0.17(±0.01)
East Copperas Pond	6/27/2023	15.92(±0.32)	5.37(±0.11)	5.00(±0.10)	17.74(±0.35)	2.30(±0.05)	1.38(±0.03)	0.34(±0.01)	7.03(±0.15)	0.09(±0.01)	0.15(±0.01)
Little Echo Pond	6/29/2023	15.80(±0.32)	6.68(±0.13)	5.09(±0.10)	14.17(±0.28)	1.54(±0.03)	1.06(±0.02)	0.59(±0.01)	16.72(±0.43)	0.16(±0.01)	0.14(±0.01)
Lake Rondaxe	6/19/2023	27.20(±0.54)	12.28(±0.25)	5.35(±0.11)	42.01(±0.84)	2.86(±0.06)	0.45(±0.01)	0.24(±0.01)	3.67(±0.02)	0.04(±0.01)	0.24(±0.01)
Cascade Lake	6/16/2023	22.29(±0.45)	10.61(±0.21)	5.13(±0.10)	36.81(±0.74)	3.19(±0.06)	0.52(±0.01)	0.19(±0.01)	3.76(±0.30)	0.04(±0.01)	0.13(±0.02)
Cascade Lake Outlet	6/16/2023	31.51(±0.63)	14.78(±0.30)	5.30(±0.11)	51.12(±1.02)	7.45(±0.15)	1.76(±0.04)	0.25(±0.01)	3.07(±0.12)	0.04(±0.01)	0.14(±0.01)
Dart Lake	5/23/2023	22.75(±0.45)	7.80(±0.16)	5.19(±0.10)	29.90(±0.60)	5.55(±0.11)	1.37(±0.03)	0.33(±0.01)	1.35(±0.03)	0.05(±0.01)	0.16(±0.03)
Woods Lake	6/16/2023	19.72(±0.39)	6.44(±0.13)	5.05(±0.10)	29.89(±0.60)	2.93(±0.06)	0.60(±0.01)	0.05(±0.01)	2.68(±0.02)	0.03(±0.01)	0.15(±0.02)
Willys Lake	5/23/2023	18.61(±0.37)	4.46(±0.09)	4.25(±0.08)	16.22(±0.32)	4.40(±0.09)	2.13(±0.04)	0.09(±0.01)	0.50(±0.08)	0.02(±0.01)	0.24(±0.01)
Loon Hollow Pond	5/23/2023	18.71(±0.37)	3.49(±0.07)	5.43(±0.11)	9.33(±0.19)	8.55(±0.17)	3.93(±0.08)	0.28(±0.01)	1.93(±0.13)	0.05(±0.01)	0.23(±0.01)
Little Clear Pond	6/22/2023	18.06(±0.36)	7.03(±0.14)	5.25(±0.10)	20.26(±0.41)	0.64(±0.01)	0.20(±0.01)	0.53(±0.01)	15.93(±0.18)	0.15(±0.01)	0.13(±0.02)
Black Pond	6/29/2023	25.83(±0.52)	22.89(±0.46)	5.27(±0.11)	57.95(±1.16)	0.64(±0.01)	0.40(±0.01)	0.06(±0.01)	2.06(±0.03)	0.03(±0.01)	0.22(±0.01)
Indian Lake	5/22/2023	18.03(±0.36)	7.98(±0.16)	2.11(±0.04)	20.26(±0.41)	10.23(±0.20)	4.06(±0.08)	0.15(±0.01)	2.75(±0.15)	0.03(±0.01)	0.12(±0.01)
Squaw Lake	5/22/2023	18.78(±0.38)	11.50(±0.23)	2.92(±0.06)	27.76(±0.56)	2.20(±0.04)	0.38(±0.01)	0.14(±0.01)	0.51(±0.06)	0.03(±0.01)	0.22(±0.01)
Queer Lake	6/6/2023	17.74(±0.35)	7.24(±0.14)	5.06(±0.10)	27.40(±0.55)	3.64(±0.07)	0.85(±0.02)	0.10(±0.01)	1.16(±0.04)	0.04(±0.01)	0.23(±0.02)
Big Hope Pond	6/23/2023	21.47(±0.43)	12.55(±0.25)	5.32(±0.11)	32.82(±0.66)	3.84(±0.08)	1.13(±0.02)	0.24(±0.01)	2.21(±0.07)	0.07(±0.01)	0.18(±0.03)
G Lake	6/29/2023	19.06(±0.38)	8.45(±0.17)	4.90(±0.10)	21.11(±0.42)	3.66(±0.07)	0.74(±0.01)	0.18(±0.01)	0.95(±0.03)	0.02(±0.01)	0.24(±0.01)
West Pond Outlet	6/16/2023	21.27(±0.43)	5.81(±0.12)	5.39(±0.11)	18.00(±0.36)	9.22(±0.18)	4.70(±0.09)	0.31(±0.01)	7.09(±0.23)	0.08(±0.01)	0.25(±0.02)
West Pond	6/16/2023	19.46(±0.39)	5.91(±0.12)	5.04(±0.10)	16.65(±0.33)	4.42(±0.09)	1.75(±0.04)	0.38(±0.01)	7.52(±0.14)	0.09(±0.01)	0.24(±0.07)
Big Moose Lake	6/16/2023	21.35(±0.43)	7.26(±0.15)	5.12(±0.10)	25.74(±0.51)	6.07(±0.12)	1.61(±0.03)	0.11(±0.01)	2.21(±0.07)	0.06(±0.01)	0.19(±0.03)
Moss Lake	5/25/2023	26.18(±0.52)	11.36(±0.23)	5.26(±0.11)	37.97(±0.76)	4.37(±0.09)	0.67(±0.01)	0.09(±0.01)	1.95(±0.23)	0.07(±0.01)	0.20(±0.02)
Bubb Lake	5/25/2023	22.87(±0.46)	10.13(±0.20)	5.18(±0.10)	31.12(±0.62)	2.24(±0.04)	0.47(±0.01)	0.04(±0.01)	0.93(±0.13)	0.02(±0.01)	0.19(±0.01)
Bubb Lake Outlet	5/25/2023	24.07(±0.48)	10.61(±0.21)	5.21(±0.10)	31.92(±0.64)	5.21(±0.10)	0.97(±0.02)	0.15(±0.01)	1.15(±0.14)	0.02(±0.01)	0.20(±0.01)
Limekiln Lake	6/6/2023	28.15(±0.56)	10.14(±0.20)	5.11(±0.10)	36.09(±0.72)	2.27(±0.05)	0.41(±0.01)	0.22(±0.01)	0.72(±0.04)	0.05(±0.01)	0.25(±0.05)
Raquette Lake Reservoir	5/23/2023	24.25(±0.49)	9.74(±0.19)	5.07(±0.10)	28.07(±0.56)	13.99(±0.28)	5.47(±0.11)	0.24(±0.01)	8.76(±0.18)	0.11(±0.01)	0.18(±0.02)
Sagamore Lake	6/20/2023	22.53(±0.45)	11.86(±0.24)	5.01(±0.10)	30.74(±0.61)	13.70(±0.27)	5.36(±0.11)	0.41(±0.01)	11.38(±0.32)	0.12(±0.01)	0.19(±0.02)
Arbutus Pond	6/23/2023	21.01(±0.42)	9.99(±0.20)	5.07(±0.10)	36.45(±0.73)	4.46(±0.09)	1.06(±0.02)	0.29(±0.01)	2.79(±0.10)	0.07(±0.01)	0.14(±0.02)

Table S3. Water chemistry parameters of whole water samples from ALTM lakes (continued)											
Site Name	Sampling Date	$[Na^+]$ (μM)	$[Mg^{2+}]$ (μM)	$[K^+]$ (μM)	$[Ca^{2+}]$ (μM)	[Al] (μM)	$[Al]_{Total\ Monomeric}$ (μM)	[Mn] (μM)	[Fe] (μM)	[Cu] (μM)	[Zn] (μM)
Clear Pond	6/23/2023	23.12(±0.46)	8.46(±0.17)	4.85(±0.10)	39.41(±0.79)	2.61(±0.05)	0.43(±0.01)	0.08(±0.01)	0.41(±0.07)	0.02(±0.01)	0.11(±0.01)
Heart Lake	6/13/2023	19.15(±0.38)	6.90(±0.14)	4.86(±0.10)	24.02(±0.48)	1.24(±0.02)	0.10(±0.01)	0.09(±0.01)	0.56(±0.19)	0.02(±0.01)	0.17(±0.02)
Grass Pond	5/16/2023	24.30(±0.49)	7.99(±0.16)	5.14(±0.10)	22.14(±0.44)	6.43(±0.13)	1.53(±0.03)	0.12(±0.01)	2.24(±0.02)	0.09(±0.01)	0.19(±0.01)
Middle Branch Lake	5/16/2023	25.74(±0.51)	9.85(±0.20)	5.23(±0.10)	28.50(±0.57)	2.48(±0.05)	0.57(±0.01)	0.09(±0.01)	1.36(±0.09)	0.06(±0.01)	0.13(±0.01)
Middle Settlement Lake	5/16/2023	22.48(±0.45)	6.17(±0.12)	5.06(±0.10)	16.61(±0.33)	2.70(±0.05)	0.79(±0.02)	0.12(±0.01)	1.24(±0.05)	0.03(±0.01)	0.24(±0.02)
South Lake	6/26/2023	18.15(±0.36)	7.44(±0.15)	5.00(±0.10)	20.54(±0.41)	3.47(±0.07)	0.75(±0.02)	0.30(±0.01)	1.64(±0.07)	0.03(±0.01)	0.13(±0.01)
North Lake	6/26/2023	20.55(±0.41)	7.59(±0.15)	5.07(±0.10)	20.95(±0.42)	9.74(±0.19)	3.19(±0.06)	0.21(±0.01)	7.29(±0.29)	0.03(±0.01)	0.26(±0.02)
Otter Lake Outlet	6/5/2023	20.84(±0.42)	8.42(±0.17)	5.06(±0.10)	19.76(±0.40)	5.12(±0.10)	1.38(±0.03)	0.26(±0.01)	0.60(±0.07)	0.04(±0.01)	0.18(±0.03)
Otter Lake	6/5/2023	19.18(±0.38)	6.92(±0.14)	4.95(±0.10)	17.42(±0.35)	4.14(±0.08)	1.27(±0.03)	0.24(±0.01)	0.75(±0.13)	0.02(±0.01)	0.19(±0.02)
Jockeybush Lake	6/5/2023	17.64(±0.35)	6.99(±0.14)	4.97(±0.10)	17.12(±0.34)	4.25(±0.09)	1.19(±0.02)	0.16(±0.01)	1.03(±0.21)	0.04(±0.01)	0.11(±0.01)
Avalanche Lake	9/25/2023	22.82(±0.46)	7.01(±0.14)	1.30(±0.03)	23.68(±0.47)	9.62(±0.19)	2.64(±0.05)	0.08(±0.01)	1.93(±0.08)	0.04(±0.01)	0.27(±0.01)
Lake Colden	9/25/2023	19.89(±0.40)	7.94(±0.16)	3.31(±0.07)	18.58(±0.37)	3.90(±0.08)	0.90(±0.02)	0.09(±0.01)	13.07(±0.73)	0.02(±0.01)	0.35(±0.01)
Brook Trout Lake	9/5/2023	19.53(±0.39)	4.56(±0.09)	3.10(±0.06)	12.14(±0.24)	12.51(±0.25)	7.12(±0.14)	0.20(±0.01)	4.42(±0.25)	0.02(±0.01)	0.43(±0.01)
Squash Pond Outlet	9/18/2023	18.37(±0.37)	4.67(±0.09)	1.43(±0.03)	13.55(±0.27)	15.57(±0.31)	8.15(±0.16)	0.13(±0.01)	4.09(±0.23)	0.03(±0.01)	0.37(±0.01)
Squash Pond	9/18/2023	28.91(±0.58)	8.50(±0.17)	5.54(±0.11)	27.24(±0.54)	4.78(±0.10)	2.18(±0.04)	0.20(±0.01)	6.29(±0.29)	0.08(±0.01)	0.30(±0.02)
Constable Pond Outlet	9/18/2023	24.66(±0.49)	6.58(±0.13)	3.78(±0.08)	22.21(±0.44)	5.69(±0.11)	3.28(±0.07)	0.13(±0.01)	6.42(±0.47)	0.03(±0.01)	0.27(±0.01)
Constable Pond	9/18/2023	16.37(±0.33)	6.43(±0.13)	5.05(±0.10)	24.46(±0.49)	2.37(±0.05)	1.70(±0.03)	0.21(±0.01)	4.98(±0.02)	0.06(±0.01)	0.31(±0.01)
East Copperas Pond	9/15/2023	14.45(±0.29)	10.35(±0.21)	5.24(±0.10)	7.26(±0.15)	2.50(±0.05)	1.65(±0.03)	0.57(±0.03)	15.84(±0.64)	0.21(±0.01)	0.15(±0.01)
Little Echo Pond	9/21/2023	37.75(±0.75)	13.55(±0.27)	6.08(±0.12)	48.21(±0.96)	2.07(±0.04)	0.64(±0.01)	0.06(±0.01)	2.54(±0.15)	0.02(±0.01)	0.44(±0.01)
Lake Rondaxe	9/23/2023	37.22(±0.74)	16.71(±0.33)	7.42(±0.15)	53.29(±1.07)	0.19(±0.01)	0.17(±0.01)	0.09(±0.01)	3.03(±0.06)	0.03(±0.01)	0.22(±0.01)
Cascade Lake	9/18/2023	53.73(±1.07)	23.31(±0.47)	10.23(±0.2)	74.86(±1.50)	1.67(±0.03)	0.49(±0.01)	0.05(±0.01)	2.17(±0.04)	0.03(±0.01)	0.48(±0.01)
Cascade Lake Outlet	9/18/2023	34.59(±0.69)	10.68(±0.21)	5.19(±0.10)	40.17(±0.80)	4.87(±0.10)	1.07(±0.02)	0.08(±0.01)	1.26(±0.09)	0.05(±0.01)	0.39(±0.01)
Dart Lake	9/22/2023	16.52(±0.33)	6.46(±0.13)	4.94(±0.10)	36.83(±0.74)	3.94(±0.08)	1.25(±0.03)	0.04(±0.01)	3.35(±0.16)	0.04(±0.01)	0.41(±0.02)
Woods Lake	9/18/2023	18.15(±0.36)	4.41(±0.09)	3.98(±0.08)	14.82(±0.30)	3.16(±0.06)	2.33(±0.05)	0.05(±0.01)	0.59(±0.04)	0.03(±0.01)	0.35(±0.01)
Willys Lake	9/19/2023	16.14(±0.32)	3.46(±0.07)	4.68(±0.09)	8.95(±0.18)	10.39(±0.21)	6.56(±0.13)	0.31(±0.02)	1.62(±0.11)	0.09(±0.01)	0.47(±0.01)
Loon Hollow Pond	9/19/2023	14.57(±0.29)	9.57(±0.19)	5.33(±0.11)	28.40(±0.57)	0.50(±0.01)	0.24(±0.01)	0.54(±0.03)	14.83(±1.29)	0.19(±0.01)	0.22(±0.01)
Little Clear Pond	9/12/2023	46.87(±0.94)	12.55(±0.25)	8.56(±0.17)	88.61(±1.77)	0.22(±0.01)	0.34(±0.01)	0.04(±0.01)	1.75(±0.07)	0.04(±0.01)	0.20(±0.02)
Black Pond	9/21/2023	17.96(±0.36)	8.27(±0.17)	2.02(±0.04)	20.42(±0.41)	10.5(±0.21)	4.22(±0.08)	0.17(±0.01)	2.15(±0.08)	0.05(±0.01)	0.51(±0.01)
Indian Lake	9/5/2023	18.73(±0.37)	12.00(±0.24)	2.96(±0.06)	27.81(±0.56)	0.42(±0.01)	0.10(±0.01)	0.05(±0.01)	0.56(±0.04)	0.04(±0.01)	0.30(±0.01)
Squaw Lake	9/5/2023	20.88(±0.42)	8.78(±0.18)	5.10(±0.10)	29.19(±0.58)	1.49(±0.03)	0.33(±0.01)	0.06(±0.01)	1.08(±0.04)	0.05(±0.01)	0.22(±0.01)
Queer Lake	9/13/2023	31.48(±0.63)	19.92(±0.40)	6.91(±0.14)	43.54(±0.87)	3.32(±0.07)	0.96(±0.02)	0.16(±0.01)	1.77(±0.02)	0.09(±0.01)	0.25(±0.01)
Big Hope Pond	9/21/2023	27.28(±0.55)	12.22(±0.24)	3.02(±0.06)	26.12(±0.52)	0.91(±0.02)	0.16(±0.01)	0.05(±0.01)	0.88(±0.01)	0.07(±0.01)	0.27(±0.01)
G Lake	9/12/2023	27.20(±0.54)	6.02(±0.12)	13.04(±0.26)	21.75(±0.43)	10.19(±0.20)	6.18(±0.12)	0.30(±0.01)	5.67(±0.21)	0.10(±0.01)	0.29(±0.01)
West Pond Outlet	9/4/2023	18.80(±0.38)	5.86(±0.12)	0.82(±0.02)	17.83(±0.36)	4.21(±0.08)	1.98(±0.04)	0.39(±0.01)	5.12(±0.22)	0.11(±0.01)	0.21(±0.01)
West Pond	9/4/2023	29.56(±0.59)	8.79(±0.18)	4.65(±0.09)	34.62(±0.69)	5.33(±0.11)	1.34(±0.03)	0.04(±0.01)	2.34(±0.11)	0.03(±0.01)	0.24(±0.01)
Big Moose Lake	9/4/2023	39.43(±0.79)	18.93(±0.38)	5.42(±0.11)	56.22(±1.12)	1.26(±0.03)	0.37(±0.01)	0.06(±0.01)	2.30(±0.26)	0.03(±0.01)	0.19(±0.01)
Moss Lake	9/26/2023	38.02(±0.76)	16.00(±0.32)	6.99(±0.14)	42.52(±0.85)	0.35(±0.01)	0.05(±0.01)	0.04(±0.01)	0.92(±0.09)	0.02(±0.01)	0.16(±0.01)
Bubb Lake	9/4/2023	40.16(±0.80)	16.53(±0.33)	8.05(±0.16)	47.02(±0.94)	0.65(±0.01)	0.10(±0.01)	0.07(±0.01)	1.05(±0.14)	0.03(±0.01)	0.16(±0.01)

Table S3. Water chemistry parameters of whole water samples from ALTM lakes (continued)											
Site Name	Sampling Date	$[Na^+]$ (μM)	$[Mg^{2+}]$ (μM)	$[K^+]$ (μM)	$[Ca^{2+}]$ (μM)	[Al] (μM)	$[Al]_{Total\ Monomeric}$ (μM)	[Mn] (μM)	[Fe] (μM)	[Cu] (μM)	[Zn] (μM)
Bubb Lake Outlet	9/4/2023	54.92(±1.10)	12.75(±0.26)	4.67(±0.09)	50.58(±1.01)	1.10(±0.02)	0.29(±0.01)	0.05(±0.01)	0.61(±0.04)	0.03(±0.01)	0.19(±0.02)
Limekiln Lake	9/4/2023	54.63(±1.09)	26.21(±0.52)	14.98(±0.30)	66.70(±1.33)	7.66(±0.15)	2.72(±0.05)	0.34(±0.01)	8.09(±0.52)	0.14(±0.01)	0.37(±0.02)
Raquette Lake Reservoir	9/26/2023	31.58(±0.63)	18.18(±0.36)	5.26(±0.11)	46.81(±0.94)	12.93(±0.26)	3.49(±0.07)	0.47(±0.01)	11.32(±0.46)	0.15(±0.01)	0.31(±0.02)
Sagamore Lake	9/29/2023	29.06(±0.58)	15.23(±0.30)	5.86(±0.12)	54.85(±1.10)	2.92(±0.06)	0.71(±0.01)	0.18(±0.01)	2.39(±0.13)	0.09(±0.01)	0.25(±0.01)
Arbutus Pond	9/26/2023	39.00(±0.78)	11.86(±0.24)	1.95(±0.04)	62.13(±1.24)	3.51(±0.07)	0.59(±0.01)	0.04(±0.01)	0.31(±0.02)	0.02(±0.01)	0.20(±0.01)
Clear Pond	9/26/2023	24.57(±0.49)	8.95(±0.18)	4.86(±0.10)	34.93(±0.70)	0.32(±0.01)	0.04(±0.01)	0.04(±0.01)	0.55(±0.11)	0.02(±0.01)	0.27(±0.01)
Heart Lake	9/5/2023	33.22(±0.66)	9.38(±0.19)	3.80(±0.08)	30.98(±0.62)	7.70(±0.15)	2.54(±0.05)	0.09(±0.01)	2.71(±0.11)	0.03(±0.01)	0.29(±0.01)
Grass Pond	9/4/2023	42.18(±0.84)	16.71(±0.33)	8.08(±0.16)	46.32(±0.93)	2.11(±0.04)	0.58(±0.01)	0.05(±0.01)	1.44(±0.05)	0.08(±0.01)	0.39(±0.02)
Middle Branch Lake	9/4/2023	45.72(±0.91)	17.18(±0.34)	7.91(±0.16)	49.91(±1.00)	2.14(±0.04)	0.64(±0.01)	0.12(±0.01)	0.92(±0.01)	0.04(±0.01)	0.28(±0.01)
Middle Settlement Lake	9/4/2023	24.37(±0.49)	9.63(±0.19)	4.28(±0.09)	27.33(±0.55)	2.48(±0.05)	0.51(±0.01)	0.14(±0.01)	1.55(±0.11)	0.04(±0.01)	0.25(±0.01)
South Lake	9/6/2023	25.00(±0.50)	10.72(±0.21)	3.75(±0.08)	31.59(±0.63)	8.97(±0.18)	2.83(±0.06)	0.10(±0.01)	6.34(±0.39)	0.04(±0.01)	0.26(±0.02)
North Lake	9/6/2023	31.77(±0.64)	12.82(±0.26)	4.74(±0.09)	28.23(±0.56)	1.94(±0.04)	0.62(±0.01)	0.14(±0.01)	0.67(±0.03)	0.09(±0.01)	0.30(±0.01)
Otter Lake Outlet	9/14/2023	26.77(±0.54)	9.48(±0.19)	4.62(±0.09)	21.27(±0.43)	1.05(±0.02)	0.28(±0.01)	0.10(±0.01)	0.72(±0.03)	0.07(±0.01)	0.46(±0.01)
Otter Lake	9/14/2023	19.58(±0.39)	8.10(±0.16)	3.12(±0.06)	17.64(±0.35)	2.95(±0.06)	0.77(±0.02)	0.08(±0.01)	0.87(±0.09)	0.07(±0.01)	0.32(±0.01)
Jockeybush Lake	9/12/2023	20.50(±0.41)	5.81(±0.12)	1.48(±0.03)	25.98(±0.52)	15.50(±0.31)	5.47(±0.11)	0.12(±0.01)	1.49(±0.20)	0.08(±0.01)	0.11(±0.02)

**Table S4.** Optical properties of whole water samples from ALTM lakes										
Site Name	Sampling Date	a_{440} (m^{-1})	*E2:E3*	S_R	$SUVA_{254}$ (L mg C^{-1}•m^{-1})	$S_{290–400}$ (μm^{-1})	$S_{300–600}$ (μm^{-1})	FI	HIX	$\beta:\alpha$
Avalanche Lake	10/17/2022	2.80(±0.01)	5.44(±0.01)	0.82(±0.01)	3.48(±0.01)	16.9(±0.1)	16.9(±0.1)	1.51(±0.02)	0.96(±0.01)	0.42(±0.01)
Lake Colden	10/17/2022	2.45(±0.02)	5.62(±0.04)	0.82(±0.01)	3.51(±0.01)	17.2(±0.1)	18.0(±0.1)	1.52(±0.02)	0.96(±0.01)	0.44(±0.01)
Brook Trout Lake	10/26/2022	0.67(±0.02)	6.70(±0.03)	0.99(±0.01)	2.16(±0.01)	18.2(±0.1)	19.7(±2.7)	1.48(±0.01)	0.88(±0.01)	0.50(±0.01)
Squash Pond Outlet	10/31/2022	2.83(±0.07)	5.39(±0.19)	0.82(±0.03)	3.47(±0.09)	16.5(±0.1)	16.9(±0.6)	1.55(±0.01)	0.95(±0.02)	0.46(±0.01)
Squash Pond	10/31/2022	5.64(±0.01)	5.06(±0.04)	0.87(±0.02)	3.75(±0.02)	15.5(±0.1)	13.5(±0.2)	1.60(±0.01)	0.95(±0.01)	0.49(±0.01)
Constable Pond Outlet	10/31/2022	2.95(±0.03)	5.06(±0.01)	0.80(±0.01)	3.45(±0.01)	16.2(±0.1)	15.7(±0.4)	1.46(±0.01)	0.94(±0.01)	0.41(±0.01)
Constable Pond	10/31/2022	2.66(±0.04)	5.46(±0.02)	0.82(±0.01)	3.23(±0.01)	16.8(±0.1)	16.2(±0.2)	1.51(±0.01)	0.94(±0.01)	0.42(±0.01)
East Copperas Pond	11/1/2022	8.05(±0.01)	4.65(±0.01)	0.85(±0.01)	4.00(±0.01)	15.0(±0.1)	12.5(±0.1)	1.40(±0.01)	0.94(±0.01)	0.39(±0.01)
Little Echo Pond	11/1/2022	17.15(±0.11)	4.14(±0.01)	0.81(±0.01)	4.68(±0.01)	14.3(±0.1)	12.5(±0.1)	1.31(±0.01)	0.95(±0.01)	0.33(±0.01)
Lake Rondaxe	11/2/2022	1.42(±0.05)	5.74(±0.02)	0.85(±0.01)	3.12(±0.01)	17.4(±0.1)	20.0(±2.7)	1.44(±0.02)	0.92(±0.01)	0.45(±0.01)
Cascade Lake	11/2/2022	0.55(±0.04)	7.48(±0.29)	1.00(±0.06)	1.22(±0.03)	19.6(±0.8)	14.5(±3.0)	1.47(±0.01)	0.88(±0.02)	0.57(±0.01)
Cascade Lake Outlet	11/2/2022	2.20(±0.04)	5.04(±0.08)	0.81(±0.01)	2.57(±0.03)	16.2(±0.3)	18.0(±3.0)	1.43(±0.01)	0.92(±0.01)	0.43(±0.01)
Dart Lake	11/2/2022	1.35(±0.01)	6.18(±0.02)	0.86(±0.01)	2.91(±0.01)	18.0(±0.1)	20.7(±2.4)	1.46(±0.01)	0.94(±0.01)	0.44(±0.01)
Woods Lake	11/2/2022	1.20(±0.02)	6.16(±0.03)	0.86(±0.01)	2.58(±0.01)	18.1(±0.1)	17.7(±1.3)	1.47(±0.01)	0.92(±0.01)	0.45(±0.01)
Willys Lake	11/3/2022	0.41(±0.01)	7.10(±0.24)	0.84(±0.03)	2.10(±0.01)	19.4(±0.7)	13.4(±2.5)	1.50(±0.02)	0.91(±0.01)	0.50(±0.01)
Loon Hollow Pond	11/3/2022	2.41(±0.01)	5.62(±0.01)	0.81(±0.01)	3.29(±0.01)	16.9(±0.1)	18.5(±0.1)	1.60(±0.01)	0.95(±0.01)	0.49(±0.01)
Little Clear Pond	11/7/2022	2.89(±0.01)	5.05(±0.04)	0.97(±0.01)	2.98(±0.02)	15.5(±0.1)	14.3(±0.8)	1.38(±0.01)	0.89(±0.01)	0.45(±0.01)
Black Pond	11/7/2022	1.09(±0.02)	5.70(±0.01)	0.92(±0.01)	3.05(±0.02)	17.2(±0.1)	15.7(±0.2)	1.42(±0.01)	0.89(±0.01)	0.48(±0.01)
Indian Lake	11/8/2022	3.61(±0.04)	5.00(±0.01)	0.79(±0.01)	3.89(±0.01)	16.1(±0.1)	15.5(±0.1)	1.48(±0.02)	0.95(±0.01)	0.41(±0.01)
Squaw Lake	11/8/2022	0.21(±0.01)	8.50(±0.05)	0.95(±0.01)	1.97(±0.02)	21.5(±0.1)	10.6(±0.1)	1.47(±0.03)	0.85(±0.01)	0.54(±0.03)
Queer Lake	11/9/2022	0.64(±0.01)	6.99(±0.03)	0.88(±0.01)	2.48(±0.02)	19.4(±0.1)	13.4(±0.4)	1.45(±0.01)	0.91(±0.01)	0.48(±0.01)
Big Hope Pond	11/10/2022	3.56(±0.05)	5.54(±0.02)	0.89(±0.01)	3.09(±0.01)	16.8(±0.1)	15.9(±0.8)	1.41(±0.02)	0.92(±0.01)	0.43(±0.01)
G Lake	11/10/2022	0.29(±0.02)	7.51(±0.13)	0.93(±0.01)	1.95(±0.02)	20.2(±0.2)	11.5(±0.1)	1.49(±0.01)	0.87(±0.01)	0.50(±0.01)
West Pond Outlet	11/14/2022	7.92(±0.07)	4.57(±0.01)	0.81(±0.01)	3.98(±0.01)	15.1(±0.1)	12.6(±0.1)	1.46(±0.01)	0.95(±0.01)	0.38(±0.01)
West Pond	11/14/2022	3.54(±0.07)	4.97(±0.02)	0.85(±0.01)	3.32(±0.01)	15.6(±0.1)	12.8(±0.2)	1.43(±0.01)	0.92(±0.01)	0.41(±0.01)
Big Moose Lake	11/14/2022	1.95(±0.01)	5.70(±0.01)	0.86(±0.01)	3.19(±0.02)	17.0(±0.1)	14.6(±0.1)	1.47(±0.02)	0.94(±0.01)	0.43(±0.01)
Moss Lake	11/14/2022	1.43(±0.01)	5.94(±0.01)	0.94(±0.01)	2.61(±0.01)	17.3(±0.1)	14.2(±0.1)	1.45(±0.01)	0.92(±0.01)	0.46(±0.01)
Bubb Lake	11/14/2022	0.72(±0.01)	6.41(±0.03)	1.13(±0.01)	1.66(±0.01)	17.0(±0.1)	13.6(±0.2)	1.44(±0.01)	0.85(±0.01)	0.50(±0.01)
Bubb Lake Outlet	11/14/2022	1.23(±0.06)	5.45(±0.04)	0.96(±0.02)	2.25(±0.02)	16.1(±0.2)	13.8(±0.8)	1.42(±0.02)	0.88(±0.01)	0.45(±0.01)
Limekiln Lake	11/15/2022	0.64(±0.02)	7.75(±0.01)	1.13(±0.01)	1.95(±0.02)	19.0(±0.1)	15.0(±0.1)	1.47(±0.03)	0.88(±0.01)	0.52(±0.02)
Raquette Lake Reservoir	11/15/2022	6.76(±0.08)	4.95(±0.01)	0.78(±0.01)	3.88(±0.01)	15.9(±0.1)	13.7(±0.2)	1.51(±0.01)	0.96(±0.01)	0.40(±0.01)
Sagamore Lake	11/15/2022	5.42(±0.01)	4.96(±0.01)	0.79(±0.01)	3.99(±0.01)	15.9(±0.1)	13.5(±0.1)	1.46(±0.01)	0.96(±0.01)	0.38(±0.01)
Arbutus Pond	11/15/2022	1.54(±0.01)	6.08(±0.01)	0.99(±0.01)	2.64(±0.01)	17.2(±0.1)	14.4(±0.2)	1.42(±0.01)	0.91(±0.01)	0.45(±0.01)
Clear Pond	11/15/2022	0.78(±0.01)	7.15(±0.05)	1.08(±0.01)	2.36(±0.01)	18.5(±0.1)	17.2(±0.7)	1.43(±0.02)	0.88(±0.01)	0.50(±0.01)
Heart Lake	11/15/2022	0.26(±0.01)	8.35(±0.17)	1.16(±0.01)	1.24(±0.01)	19.4(±0.2)	20.3(±5.1)	1.50(±0.05)	0.83(±0.01)	0.54(±0.01)
Grass Pond	11/17/2022	2.94(±0.01)	5.04(±0.01)	0.85(±0.01)	3.41(±0.01)	15.8(±0.1)	14.0(±0.3)	1.48(±0.01)	0.95(±0.01)	0.41(±0.01)
Middle Branch Lake	11/17/2022	2.19(±0.01)	5.27(±0.01)	0.94(±0.01)	3.06(±0.01)	16.0(±0.1)	13.8(±0.1)	1.42(±0.02)	0.92(±0.01)	0.43(±0.01)
Middle Settlement Lake	11/17/2022	1.23(±0.05)	5.90(±0.03)	0.96(±0.02)	2.14(±0.03)	16.9(±0.1)	14.3(±0.4)	1.49(±0.01)	0.90(±0.01)	0.45(±0.01)

Table S4. Optical properties of whole water samples from ALTM lakes (continued)

Site Name	Sampling Date	a_{440} (m^{-1})	*E2:E3*	S_R	SUVA$_{254}$ (L mg C^{-1}•m^{-1})	$S_{290–400}$ (μm^{-1})	$S_{300–600}$ (μm^{-1})	FI	HIX	$\beta:\alpha$
South Lake	11/28/2022	1.16(±0.02)	6.02(±0.03)	0.93(±0.01)	2.74(±0.01)	17.2(±0.1)	14.5(±0.2)	1.48(±0.05)	0.91(±0.01)	0.47(±0.01)
North Lake	11/28/2022	4.42(±0.02)	4.83(±0.01)	0.79(±0.01)	3.94(±0.01)	15.7(±0.1)	13.8(±0.1)	1.45(±0.02)	0.96(±0.01)	0.39(±0.01)
Otter Lake Outlet	11/29/2022	1.03(±0.03)	5.81(±0.06)	0.89(±0.01)	2.37(±0.01)	17.0(±0.1)	14.7(±1.0)	1.51(±0.01)	0.92(±0.01)	0.46(±0.01)
Otter Lake	11/29/2022	0.82(±0.01)	6.19(±0.05)	0.94(±0.01)	2.35(±0.01)	17.5(±0.1)	16.2(±0.8)	1.46(±0.01)	0.90(±0.01)	0.46(±0.01)
Jockeybush Lake	11/29/2022	1.33(±0.07)	5.75(±0.39)	0.95(±0.08)	2.66(±0.05)	16.5(±1.2)	12.7(±4.0)	1.50(±0.01)	0.91(±0.01)	0.46(±0.01)
Avalanche Lake	6/2/2023	2.30(±0.02)	5.53(±0.01)	0.93(±0.01)	2.80(±0.01)	16.4(±0.1)	13.0(±0.1)	1.45(±0.01)	0.95(±0.01)	0.42(±0.01)
Lake Colden	6/2/2023	1.74(±0.05)	5.75(±0.03)	0.99(±0.01)	2.26(±0.01)	16.6(±0.1)	12.5(±0.5)	1.49(±0.01)	0.93(±0.01)	0.45(±0.01)
Brook Trout Lake	5/22/2023	1.35(±0.07)	5.58(±0.07)	0.98(±0.02)	2.58(±0.01)	16.3(±0.2)	12.3(±0.5)	1.48(±0.02)	0.92(±0.01)	0.45(±0.01)
Squash Pond Outlet	5/15/2023	4.33(±0.04)	4.82(±0.01)	0.82(±0.01)	3.18(±0.02)	15.5(±0.1)	13.4(±0.1)	1.55(±0.02)	0.96(±0.01)	0.46(±0.01)
Squash Pond	5/15/2023	5.18(±0.02)	4.89(±0.01)	0.84(±0.01)	3.57(±0.01)	15.5(±0.1)	13.5(±0.1)	1.55(±0.01)	0.96(±0.01)	0.48(±0.01)
Constable Pond Outlet	5/15/2023	3.08(±0.21)	5.00(±0.08)	0.86(±0.01)	2.73(±0.02)	15.8(±0.2)	13.4(±0.4)	1.48(±0.01)	0.95(±0.01)	0.42(±0.01)
Constable Pond	5/15/2023	2.88(±0.05)	5.24(±0.02)	0.86(±0.01)	3.17(±0.01)	16.2(±0.1)	14.1(±0.3)	1.53(±0.01)	0.95(±0.01)	0.45(±0.01)
East Copperas Pond	6/27/2023	6.91(±0.01)	4.74(±0.01)	0.87(±0.01)	3.90(±0.01)	15.2(±0.1)	12.1(±0.1)	1.36(±0.01)	0.93(±0.01)	0.40(±0.01)
Little Echo Pond	6/29/2023	10.60(±0.13)	4.21(±0.01)	0.82(±0.01)	4.89(±0.01)	14.4(±0.1)	12.4(±0.1)	1.28(±0.01)	0.94(±0.01)	0.35(±0.01)
Lake Rondaxe	6/19/2023	1.74(±0.05)	5.36(±0.06)	0.94(±0.01)	2.75(±0.02)	16.3(±0.1)	13.6(±0.3)	1.41(±0.01)	0.90(±0.01)	0.45(±0.01)
Cascade Lake	6/16/2023	1.16(±0.02)	5.86(±0.01)	1.04(±0.01)	2.13(±0.02)	16.9(±0.1)	13.6(±0.4)	1.46(±0.01)	0.90(±0.01)	0.50(±0.01)
Cascade Lake Outlet	6/16/2023	3.80(±0.01)	4.64(±0.01)	0.81(±0.01)	3.55(±0.01)	15.4(±0.1)	13.5(±0.1)	1.44(±0.01)	0.94(±0.01)	0.39(±0.01)
Dart Lake	5/23/2023	2.27(±0.03)	5.40(±0.01)	0.86(±0.01)	3.52(±0.01)	16.7(±0.1)	15.4(±0.3)	1.45(±0.01)	0.95(±0.01)	0.41(±0.01)
Woods Lake	6/16/2023	1.45(±0.05)	5.70(±0.04)	0.95(±0.01)	2.63(±0.03)	16.9(±0.2)	15.4(±1.3)	1.45(±0.02)	0.90(±0.01)	0.46(±0.01)
Willys Lake	5/23/2023	0.81(±0.07)	6.06(±0.08)	0.89(±0.02)	2.60(±0.01)	17.4(±0.3)	17.8(±1.1)	1.59(±0.02)	0.94(±0.01)	0.58(±0.02)
Loon Hollow Pond	5/23/2023	2.37(±0.01)	5.44(±0.01)	0.85(±0.01)	3.55(±0.01)	16.4(±0.1)	16.0(±0.4)	1.58(±0.01)	0.96(±0.01)	0.53(±0.01)
Little Clear Pond	6/22/2023	2.86(±0.03)	4.97(±0.01)	0.97(±0.01)	3.08(±0.02)	15.4(±0.1)	13.2(±0.4)	1.36(±0.01)	0.89(±0.01)	0.45(±0.01)
Black Pond	6/29/2023	1.77(±0.09)	5.27(±0.05)	1.00(±0.02)	3.33(±0.02)	16.1(±0.2)	13.8(±1.4)	1.39(±0.05)	0.89(±0.01)	0.47(±0.01)
Indian Lake	5/22/2023	3.28(±0.09)	5.03(±0.03)	0.84(±0.01)	2.89(±0.02)	15.9(±0.1)	14.3(±0.9)	1.48(±0.01)	0.95(±0.01)	0.43(±0.01)
Squaw Lake	5/22/2023	1.33(±0.01)	5.40(±0.08)	0.95(±0.03)	2.77(±0.01)	16.4(±0.4)	16.1(±3.4)	1.41(±0.04)	0.92(±0.01)	0.45(±0.01)
Queer Lake	6/6/2023	1.34(±0.05)	5.77(±0.03)	0.94(±0.01)	2.94(±0.01)	17.0(±0.2)	17.1(±2.5)	1.42(±0.02)	0.91(±0.01)	0.47(±0.01)
Big Hope Pond	6/23/2023	4.07(±0.02)	5.38(±0.01)	0.91(±0.01)	3.45(±0.01)	16.4(±0.1)	14.4(±0.3)	1.38(±0.02)	0.92(±0.01)	0.43(±0.01)
G Lake	6/29/2023	1.09(±0.02)	6.10(±0.21)	1.20(±0.04)	2.28(±0.02)	16.1(±0.5)	10.6(±0.9)	1.46(±0.01)	0.88(±0.01)	0.48(±0.01)
West Pond Outlet	6/16/2023	12.93(±0.18)	4.14(±0.01)	0.83(±0.01)	4.55(±0.01)	13.9(±0.1)	11.0(±0.1)	1.44(±0.01)	0.95(±0.01)	0.38(±0.01)
West Pond	6/16/2023	4.08(±0.07)	4.64(±0.02)	0.88(±0.01)	3.60(±0.01)	14.7(±0.1)	11.5(±0.3)	1.45(±0.01)	0.94(±0.01)	0.41(±0.01)
Big Moose Lake	6/16/2023	2.63(±0.03)	5.30(±0.02)	0.90(±0.01)	3.31(±0.01)	16.1(±0.1)	12.6(±0.2)	1.48(±0.02)	0.94(±0.01)	0.43(±0.01)
Moss Lake	5/25/2023	2.07(±0.08)	5.26(±0.06)	0.97(±0.01)	3.27(±0.01)	15.8(±0.2)	11.8(±0.5)	1.42(±0.03)	0.93(±0.01)	0.43(±0.01)
Bubb Lake	5/25/2023	1.26(±0.07)	5.44(±0.11)	1.13(±0.02)	2.12(±0.02)	15.4(±0.3)	11.0(±0.5)	1.42(±0.03)	0.88(±0.01)	0.46(±0.02)
Bubb Lake Outlet	5/25/2023	1.46(±0.03)	4.96(±0.03)	0.97(±0.01)	1.87(±0.01)	15.1(±0.1)	11.3(±0.3)	1.45(±0.01)	0.92(±0.01)	0.44(±0.01)
Limekiln Lake	6/6/2023	2.12(±0.01)	5.31(±0.08)	1.02(±0.01)	2.79(±0.02)	15.7(±0.2)	11.6(±0.4)	1.48(±0.01)	0.89(±0.01)	0.46(±0.01)
Raquette Lake Reservoir	5/23/2023	4.06(±0.07)	5.01(±0.02)	0.84(±0.01)	2.55(±0.01)	15.8(±0.1)	13.2(±0.3)	1.44(±0.01)	0.96(±0.01)	0.39(±0.01)
Sagamore Lake	6/20/2023	4.55(±0.06)	5.04(±0.02)	0.87(±0.01)	2.44(±0.01)	15.7(±0.1)	12.9(±0.2)	1.42(±0.01)	0.94(±0.01)	0.38(±0.01)
Arbutus Pond	6/23/2023	2.21(±0.02)	5.59(±0.02)	1.01(±0.01)	2.40(±0.01)	16.3(±0.1)	12.5(±0.2)	1.37(±0.01)	0.91(±0.01)	0.44(±0.01)

Table S4. Optical properties of whole water samples from ALTM lakes (continued)										
Site Name	Sampling Date	a_{440} (m^{-1})	*E2:E3*	S_R	$SUVA_{254}$ (L mg C^{-1}•m^{-1})	$S_{290–400}$ (μm^{-1})	$S_{300–600}$ (μm^{-1})	FI	HIX	$\beta:\alpha$
Clear Pond	6/23/2023	1.40(±0.01)	6.12(±0.04)	1.20(±0.01)	2.71(±0.02)	16.4(±0.1)	11.4(±0.1)	1.42(±0.06)	0.88(±0.01)	0.49(±0.01)
Heart Lake	6/13/2023	0.76(±0.04)	6.10(±0.11)	1.35(±0.01)	1.83(±0.02)	15.6(±0.2)	10.5(±0.4)	1.49(±0.02)	0.85(±0.01)	0.51(±0.01)
Grass Pond	5/16/2023	1.80(±0.06)	5.24(±0.04)	0.98(±0.01)	2.58(±0.01)	15.8(±0.1)	12.4(±0.6)	1.46(±0.01)	0.93(±0.01)	0.43(±0.01)
Middle Branch Lake	5/16/2023	2.20(±0.01)	4.88(±0.06)	0.95(±0.01)	3.30(±0.02)	15.3(±0.2)	12.1(±0.6)	1.41(±0.01)	0.92(±0.01)	0.41(±0.01)
Middle Settlement Lake	5/16/2023	1.37(±0.04)	5.56(±0.05)	1.02(±0.01)	2.49(±0.02)	16.1(±0.1)	12.4(±0.5)	1.44(±0.01)	0.92(±0.01)	0.44(±0.01)
South Lake	6/26/2023	1.31(±0.01)	5.78(±0.14)	1.04(±0.02)	2.66(±0.02)	16.2(±0.4)	11.9(±1.1)	1.47(±0.02)	0.90(±0.01)	0.46(±0.01)
North Lake	6/26/2023	2.85(±0.10)	5.08(±0.05)	0.90(±0.01)	2.76(±0.01)	15.6(±0.1)	12.6(±0.6)	1.44(±0.02)	0.94(±0.01)	0.41(±0.01)
Otter Lake Outlet	6/5/2023	1.03(±0.08)	5.78(±0.13)	1.04(±0.02)	2.23(±0.02)	16.3(±0.3)	12.2(±1.2)	1.50(±0.03)	0.92(±0.01)	0.47(±0.01)
Otter Lake	6/5/2023	1.08(±0.10)	6.07(±0.14)	1.06(±0.03)	2.42(±0.02)	16.7(±0.4)	12.6(±1.2)	1.49(±0.02)	0.91(±0.01)	0.47(±0.01)
Jockeybush Lake	6/5/2023	1.17(±0.07)	5.95(±0.10)	1.08(±0.02)	2.22(±0.02)	16.4(±0.2)	11.8(±0.7)	1.44(±0.02)	0.89(±0.01)	0.47(±0.01)
Avalanche Lake	6/2/2023	3.09(±0.07)	5.53(±0.01)	0.93(±0.01)	3.65(±0.01)	16.4(±0.1)	13.0(±0.1)	1.45(±0.01)	0.95(±0.01)	0.42(±0.01)
Avalanche Lake	9/25/2023	2.13(±0.09)	5.44(±0.07)	0.84(±0.01)	3.38(±0.01)	16.7(±0.2)	15.3(±1.5)	1.53(±0.01)	0.96(±0.01)	0.43(±0.01)
Lake Colden	9/25/2023	0.90(±0.03)	5.63(±0.05)	0.85(±0.01)	2.36(±0.01)	17.1(±0.2)	17.3(±1.5)	1.52(±0.01)	0.95(±0.01)	0.45(±0.01)
Brook Trout Lake	9/5/2023	4.42(±0.09)	6.41(±0.03)	0.95(±0.01)	3.57(±0.01)	18.0(±0.1)	19.6(±1.8)	1.47(±0.01)	0.91(±0.01)	0.50(±0.01)
Squash Pond Outlet	9/18/2023	4.80(±0.04)	4.96(±0.04)	0.82(±0.01)	3.04(±0.01)	15.6(±0.2)	13.9(±0.9)	1.57(±0.01)	0.96(±0.01)	0.48(±0.01)
Squash Pond	9/18/2023	3.92(±0.01)	5.32(±0.02)	0.85(±0.01)	3.03(±0.01)	16.1(±0.1)	14.1(±0.2)	1.61(±0.01)	0.95(±0.01)	0.50(±0.01)
Constable Pond Outlet	9/18/2023	3.08(±0.02)	4.84(±0.01)	0.78(±0.01)	3.03(±0.01)	15.8(±0.1)	14.0(±0.1)	1.51(±0.01)	0.94(±0.01)	0.42(±0.01)
Constable Pond	9/18/2023	7.59(±0.05)	5.24(±0.01)	0.82(±0.01)	4.23(±0.01)	16.4(±0.1)	15.1(±0.4)	1.54(±0.02)	0.95(±0.01)	0.46(±0.01)
East Copperas Pond	9/15/2023	15.47(±0.16)	4.73(±0.02)	0.83(±0.01)	4.84(±0.01)	15.4(±0.1)	12.7(±0.1)	1.40(±0.01)	0.94(±0.01)	0.39(±0.01)
Little Echo Pond	9/21/2023	1.78(±0.08)	4.15(±0.01)	0.79(±0.01)	2.54(±0.02)	14.5(±0.1)	12.6(±0.1)	1.29(±0.01)	0.95(±0.01)	0.34(±0.01)
Lake Rondaxe	9/23/2023	0.82(±0.04)	5.60(±0.13)	0.87(±0.02)	2.20(±0.02)	17.1(±0.4)	15.8(±0.5)	1.46(±0.01)	0.93(±0.01)	0.44(±0.01)
Cascade Lake	9/18/2023	2.07(±0.03)	6.21(±0.09)	0.93(±0.01)	3.29(±0.01)	18.2(±0.2)	15.0(±0.8)	1.52(±0.01)	0.90(±0.01)	0.53(±0.01)
Cascade Lake Outlet	9/18/2023	2.01(±0.01)	5.11(±0.01)	0.79(±0.01)	3.07(±0.02)	16.5(±0.1)	18.1(±1.4)	1.47(±0.01)	0.94(±0.01)	0.42(±0.01)
Dart Lake	9/22/2023	2.55(±0.07)	5.70(±0.08)	0.85(±0.01)	3.02(±0.01)	17.4(±0.2)	19.8(±1.0)	1.46(±0.01)	0.93(±0.01)	0.45(±0.01)
Woods Lake	9/18/2023	0.65(±0.03)	5.34(±0.04)	0.83(±0.01)	2.36(±0.01)	16.8(±0.1)	18.7(±2.1)	1.48(±0.01)	0.93(±0.01)	0.44(±0.01)
Willys Lake	9/19/2023	3.19(±0.08)	6.32(±0.03)	0.82(±0.01)	3.43(±0.01)	18.3(±0.2)	14.4(±1.3)	1.56(±0.02)	0.93(±0.01)	0.54(±0.01)
Loon Hollow Pond	9/19/2023	2.59(±0.04)	5.34(±0.04)	0.81(±0.01)	3.07(±0.01)	16.5(±0.1)	17.1(±1.0)	1.58(±0.01)	0.95(±0.01)	0.50(±0.01)
Little Clear Pond	9/12/2023	1.89(±0.10)	5.14(±0.03)	0.90(±0.01)	3.30(±0.02)	16.0(±0.1)	16.8(±0.3)	1.38(±0.02)	0.91(±0.01)	0.46(±0.01)
Black Pond	9/21/2023	4.94(±0.10)	5.35(±0.08)	0.92(±0.01)	3.87(±0.01)	16.5(±0.2)	19.7(±1.8)	1.41(±0.01)	0.92(±0.01)	0.45(±0.01)
Indian Lake	9/5/2023	0.61(±0.06)	4.83(±0.01)	0.79(±0.01)	2.39(±0.03)	15.7(±0.1)	14.1(±0.6)	1.53(±0.01)	0.96(±0.01)	0.42(±0.01)
Squaw Lake	9/5/2023	0.65(±0.05)	6.89(±0.18)	0.93(±0.02)	2.50(±0.02)	19.3(±0.4)	13.8(±0.8)	1.44(±0.01)	0.90(±0.01)	0.50(±0.02)
Queer Lake	9/13/2023	3.96(±0.04)	7.03(±0.17)	0.91(±0.01)	3.59(±0.01)	19.4(±0.3)	13.7(±0.8)	1.45(±0.03)	0.90(±0.01)	0.49(±0.01)
Big Hope Pond	9/21/2023	3.09(±0.07)	5.43(±0.03)	0.89(±0.01)	3.65(±0.01)	16.5(±0.1)	15.4(±0.2)	1.42(±0.01)	0.93(±0.01)	0.44(±0.01)
G Lake	9/12/2023	0.73(±0.03)	7.44(±0.92)	1.04(±0.22)	2.06(±0.01)	19.4(±3.1)	11.6(±1.2)	1.43(±0.01)	0.88(±0.01)	0.52(±0.01)
West Pond Outlet	9/4/2023	17.45(±0.11)	4.19(±0.01)	0.80(±0.01)	4.57(±0.01)	14.3(±0.1)	11.5(±0.1)	1.45(±0.01)	0.96(±0.01)	0.36(±0.01)
West Pond	9/4/2023	4.67(±0.03)	4.69(±0.01)	0.83(±0.01)	3.75(±0.01)	15.2(±0.1)	12.3(±0.1)	1.44(±0.01)	0.94(±0.01)	0.42(±0.01)
Big Moose Lake	9/4/2023	2.68(±0.01)	5.46(±0.01)	0.84(±0.01)	3.28(±0.01)	16.7(±0.1)	14.9(±0.2)	1.45(±0.02)	0.94(±0.01)	0.44(±0.01)
Moss Lake	9/26/2023	1.39(±0.03)	6.16(±0.02)	0.97(±0.01)	2.58(±0.01)	17.5(±0.1)	14.9(±0.1)	1.44(±0.01)	0.91(±0.01)	0.47(±0.01)

**Table S4.** Optical properties of whole water samples from ALTM lakes (continued)										
Site Name	Sampling Date	a_{440} (m^{-1})	*E2:E3*	S_R	$SUVA_{254}$ (L mg C^{-1}•m^{-1})	$S_{290–400}$ (μm^{-1})	$S_{300–600}$ (μm^{-1})	FI	HIX	$\beta:\alpha$
Bubb Lake	9/4/2023	0.68(±0.01)	6.88(±0.23)	1.21(±0.06)	1.60(±0.01)	17.4(±0.7)	13.5(±2.8)	1.44(±0.03)	0.86(±0.01)	0.56(±0.01)
Bubb Lake Outlet	9/4/2023	0.76(±0.02)	6.05(±0.06)	1.00(±0.01)	1.92(±0.01)	17.0(±0.1)	15.4(±1.1)	1.48(±0.03)	0.90(±0.01)	0.52(±0.01)
Limekiln Lake	9/4/2023	0.95(±0.03)	6.75(±0.05)	1.02(±0.01)	2.27(±0.01)	18.2(±0.1)	16.4(±1.1)	1.49(±0.01)	0.90(±0.01)	0.50(±0.01)
Raquette Lake Reservoir	9/26/2023	9.73(±0.01)	4.39(±0.01)	0.75(±0.01)	4.62(±0.01)	15.0(±0.1)	12.9(±0.1)	1.47(±0.01)	0.95(±0.01)	0.37(±0.01)
Sagamore Lake	9/29/2023	8.39(±0.05)	4.76(±0.01)	0.79(±0.01)	4.37(±0.01)	15.6(±0.1)	13.5(±0.1)	1.44(±0.01)	0.96(±0.01)	0.39(±0.01)
Arbutus Pond	9/26/2023	3.57(±0.01)	5.27(±0.01)	0.88(±0.01)	3.57(±0.01)	16.2(±0.1)	13.5(±0.1)	1.42(±0.01)	0.94(±0.01)	0.43(±0.01)
Clear Pond	9/26/2023	1.63(±0.01)	5.98(±0.01)	1.00(±0.01)	2.98(±0.01)	17.2(±0.1)	15.0(±0.2)	1.42(±0.02)	0.91(±0.01)	0.47(±0.01)
Heart Lake	9/5/2023	0.44(±0.01)	7.30(±0.01)	1.20(±0.01)	1.67(±0.01)	18.0(±0.1)	18.6(±3.1)	1.42(±0.03)	0.86(±0.01)	0.52(±0.03)
Grass Pond	9/4/2023	2.59(±0.01)	5.26(±0.01)	0.87(±0.01)	2.91(±0.01)	16.2(±0.1)	14.3(±0.2)	1.52(±0.01)	0.95(±0.01)	0.44(±0.01)
Middle Branch Lake	9/4/2023	2.37(±0.01)	5.27(±0.01)	0.87(±0.01)	3.05(±0.01)	16.4(±0.1)	15.0(±0.1)	1.40(±0.02)	0.93(±0.01)	0.42(±0.01)
Middle Settlement Lake	9/4/2023	1.26(±0.01)	6.00(±0.01)	0.93(±0.01)	2.47(±0.01)	17.2(±0.1)	16.5(±0.1)	1.47(±0.03)	0.93(±0.01)	0.46(±0.01)
South Lake	9/6/2023	1.24(±0.03)	6.03(±0.03)	0.94(±0.01)	2.85(±0.01)	17.3(±0.1)	15.5(±0.8)	1.46(±0.01)	0.92(±0.01)	0.46(±0.01)
North Lake	9/6/2023	4.76(±0.01)	4.92(±0.01)	0.81(±0.01)	4.15(±0.01)	15.8(±0.1)	14.3(±0.1)	1.46(±0.01)	0.95(±0.01)	0.41(±0.01)
Otter Lake Outlet	9/14/2023	1.09(±0.01)	5.71(±0.01)	0.88(±0.01)	2.58(±0.01)	17.0(±0.1)	15.6(±0.1)	1.52(±0.03)	0.92(±0.01)	0.47(±0.01)
Otter Lake	9/14/2023	0.63(±0.02)	6.89(±0.04)	0.98(±0.01)	2.07(±0.01)	18.5(±0.1)	21.9(±4.9)	1.51(±0.01)	0.89(±0.01)	0.51(±0.01)
Jockeybush Lake	9/12/2023	1.00(±0.01)	6.24(±0.01)	0.96(±0.01)	2.26(±0.01)	17.5(±0.1)	17.4(±0.8)	1.46(±0.01)	0.91(±0.01)	0.47(±0.01)

**Table S4.** Optical properties of whole water samples from ALTM lakes (continued)									
Site Name	Sampling Date	Peak A : Peak T	Peak C : Peak A	Peak C : Peak M	Peak C : Peak T	PARAFAC C1 (R.U.)	PARAFAC C2 (R.U.)	PARAFAC C3 (R.U.)	PARAFAC C4 (R.U.)
Avalanche Lake	10/17/2022	11.34(±0.18)	0.95(±0.01)	1.58(±0.01)	10.72(±0.13)	4.06(±0.01)	3.11(±0.01)	3.36(±0.02)	0.38(±0.01)
Lake Colden	10/17/2022	10.55(±1.50)	0.98(±0.01)	1.58(±0.01)	10.37(±1.55)	3.43(±0.07)	2.53(±0.01)	2.89(±0.02)	0.30(±0.05)
Brook Trout Lake	10/26/2022	3.25(±0.01)	0.91(±0.01)	1.21(±0.01)	2.96(±0.01)	1.25(±0.01)	0.58(±0.01)	0.63(±0.01)	0.42(±0.01)
Squash Pond Outlet	10/31/2022	10.57(±1.31)	1.05(±0.15)	1.81(±0.21)	10.95(±0.17)	3.54(±0.09)	2.76(±0.04)	3.16(±0.10)	0.38(±0.06)
Squash Pond	10/31/2022	9.02(±0.47)	0.93(±0.01)	1.63(±0.01)	8.36(±0.44)	5.41(±0.10)	3.70(±0.04)	4.73(±0.07)	0.80(±0.08)
Constable Pond Outlet	10/31/2022	6.68(±0.27)	0.95(±0.01)	1.40(±0.01)	6.38(±0.31)	3.09(±0.07)	1.83(±0.03)	1.83(±0.02)	0.40(±0.04)
Constable Pond	10/31/2022	7.46(±1.20)	0.94(±0.01)	1.36(±0.01)	7.00(±1.13)	3.63(±0.06)	2.00(±0.03)	2.20(±0.03)	0.50(±0.08)
East Copperas Pond	11/1/2022	7.33(±0.32)	0.93(±0.01)	1.45(±0.01)	6.83(±0.38)	4.94(±0.08)	3.30(±0.05)	2.68(±0.06)	0.71(±0.05)
Little Echo Pond	11/1/2022	8.15(±0.37)	1.03(±0.01)	1.66(±0.02)	8.43(±0.50)	5.89(±0.10)	4.98(±0.09)	3.64(±0.07)	0.36(±0.07)
Lake Rondaxe	11/2/2022	5.38(±0.41)	0.97(±0.01)	1.28(±0.01)	5.20(±0.39)	2.13(±0.04)	1.02(±0.02)	1.01(±0.01)	0.42(±0.04)
Cascade Lake	11/2/2022	3.53(±0.73)	0.90(±0.01)	1.12(±0.01)	3.17(±0.68)	1.38(±0.02)	0.53(±0.01)	0.56(±0.01)	0.51(±0.08)
Cascade Lake Outlet	11/2/2022	5.32(±0.67)	0.95(±0.01)	1.36(±0.01)	5.04(±0.64)	2.12(±0.05)	1.37(±0.02)	1.22(±0.03)	0.41(±0.05)
Dart Lake	11/2/2022	7.27(±0.06)	0.96(±0.01)	1.29(±0.01)	6.97(±0.03)	2.56(±0.05)	1.25(±0.02)	1.24(±0.02)	0.38(±0.03)
Woods Lake	11/2/2022	5.46(±0.49)	0.97(±0.01)	1.26(±0.01)	5.31(±0.50)	2.38(±0.04)	1.09(±0.02)	1.16(±0.03)	0.44(±0.04)
Willys Lake	11/3/2022	5.43(±1.08)	0.89(±0.01)	1.30(±0.01)	4.84(±0.99)	1.33(±0.02)	0.69(±0.01)	0.81(±0.01)	0.32(±0.05)
Loon Hollow Pond	11/3/2022	9.96(±1.20)	0.91(±0.01)	1.56(±0.01)	9.08(±1.09)	4.03(±0.07)	2.57(±0.03)	3.34(±0.05)	0.57(±0.05)
Little Clear Pond	11/7/2022	3.69(±0.04)	0.87(±0.01)	1.28(±0.01)	3.20(±0.05)	2.17(±0.04)	1.31(±0.02)	1.07(±0.02)	0.70(±0.03)
Black Pond	11/7/2022	4.19(±0.28)	0.89(±0.01)	1.21(±0.01)	3.72(±0.30)	1.29(±0.02)	0.64(±0.01)	0.59(±0.01)	0.37(±0.04)
Indian Lake	11/8/2022	8.27(±0.76)	0.94(±0.01)	1.42(±0.01)	7.79(±0.80)	3.73(±0.04)	2.31(±0.03)	2.34(±0.03)	0.43(±0.06)
Squaw Lake	11/8/2022	2.91(±0.08)	0.85(±0.01)	1.17(±0.01)	2.47(±0.07)	0.88(±0.02)	0.40(±0.01)	0.40(±0.01)	0.39(±0.03)
Queer Lake	11/9/2022	5.14(±0.22)	0.93(±0.01)	1.24(±0.01)	4.78(±0.21)	1.64(±0.03)	0.73(±0.01)	0.73(±0.01)	0.39(±0.03)
Big Hope Pond	11/10/2022	5.41(±0.14)	0.91(±0.01)	1.30(±0.01)	4.93(±0.16)	3.78(±0.06)	2.09(±0.03)	1.83(±0.02)	0.78(±0.04)
G Lake	11/10/2022	3.42(±0.28)	0.96(±0.01)	1.19(±0.01)	3.26(±0.29)	1.01(±0.02)	0.42(±0.01)	0.47(±0.01)	0.32(±0.03)
West Pond Outlet	11/14/2022	8.26(±0.57)	0.97(±0.01)	1.56(±0.01)	8.04(±0.52)	4.43(±0.09)	3.30(±0.06)	3.15(±0.05)	0.43(±0.02)
West Pond	11/14/2022	5.36(±0.40)	0.94(±0.01)	1.40(±0.03)	5.02(±0.36)	2.87(±0.04)	1.84(±0.03)	1.66(±0.03)	0.55(±0.05)
Big Moose Lake	11/14/2022	6.98(±0.72)	0.96(±0.01)	1.31(±0.01)	6.71(±0.76)	2.73(±0.03)	1.38(±0.02)	1.40(±0.04)	0.42(±0.05)
Moss Lake	11/14/2022	5.15(±0.03)	0.94(±0.01)	1.24(±0.01)	4.86(±0.03)	1.89(±0.02)	0.91(±0.01)	0.89(±0.01)	0.41(±0.01)
Bubb Lake	11/14/2022	2.62(±0.13)	0.89(±0.01)	1.20(±0.01)	2.33(±0.09)	0.81(±0.02)	0.42(±0.01)	0.41(±0.01)	0.36(±0.02)
Bubb Lake Outlet	11/14/2022	3.34(±0.06)	0.93(±0.01)	1.31(±0.02)	3.10(±0.04)	1.06(±0.02)	0.69(±0.01)	0.63(±0.01)	0.34(±0.02)
Limekiln Lake	11/15/2022	3.37(±0.43)	0.91(±0.01)	1.17(±0.01)	3.08(±0.35)	1.12(±0.01)	0.46(±0.01)	0.50(±0.01)	0.39(±0.03)
Raquette Lake Reservoir	11/15/2022	10.88(±0.01)	1.01(±0.01)	1.58(±0.01)	11.01(±0.13)	5.13(±0.09)	3.86(±0.07)	4.14(±0.06)	0.34(±0.02)
Sagamore Lake	11/15/2022	10.39(±1.45)	0.98(±0.01)	1.45(±0.01)	10.20(±1.37)	4.57(±0.04)	3.07(±0.02)	2.98(±0.03)	0.36(±0.06)
Arbutus Pond	11/15/2022	4.82(±0.03)	0.91(±0.01)	1.23(±0.01)	4.41(±0.03)	1.80(±0.03)	0.93(±0.01)	0.84(±0.01)	0.46(±0.02)
Clear Pond	11/15/2022	3.65(±0.07)	0.89(±0.01)	1.20(±0.01)	3.26(±0.12)	1.05(±0.02)	0.49(±0.01)	0.46(±0.01)	0.36(±0.01)
Heart Lake	11/15/2022	2.37(±0.09)	0.90(±0.01)	1.16(±0.02)	2.13(±0.10)	0.47(±0.01)	0.23(±0.01)	0.24(±0.01)	0.23(±0.01)
Grass Pond	11/17/2022	7.83(±0.40)	0.99(±0.01)	1.45(±0.01)	7.76(±0.38)	2.80(±0.02)	1.82(±0.01)	1.95(±0.01)	0.29(±0.03)
Middle Branch Lake	11/17/2022	5.58(±0.20)	0.93(±0.01)	1.32(±0.01)	5.19(±0.18)	2.14(±0.02)	1.24(±0.01)	1.12(±0.01)	0.43(±0.02)
Middle Settlement Lake	11/17/2022	3.78(±0.61)	0.96(±0.01)	1.32(±0.01)	3.63(±0.56)	1.67(±0.01)	0.89(±0.01)	0.96(±0.02)	0.45(±0.06)

**Table S4.** Optical properties of whole water samples from ALTM lakes (continued)									
Site Name	Sampling Date	Peak A : Peak T	Peak C : Peak A	Peak C : Peak M	Peak C : Peak T	PARAFAC C1 (R.U.)	PARAFAC C2 (R.U.)	PARAFAC C3 (R.U.)	PARAFAC C4 (R.U.)
South Lake	11/28/2022	4.75(±0.19)	0.93(±0.01)	1.25(±0.01)	4.41(±0.18)	1.57(±0.02)	0.78(±0.01)	0.83(±0.01)	0.38(±0.02)
North Lake	11/28/2022	10.00(±0.18)	0.96(±0.01)	1.48(±0.01)	9.58(±0.19)	3.66(±0.05)	2.57(±0.02)	2.49(±0.02)	0.32(±0.02)
Otter Lake Outlet	11/29/2022	5.14(±0.08)	0.95(±0.01)	1.33(±0.01)	4.86(±0.13)	1.34(±0.02)	0.81(±0.01)	0.85(±0.01)	0.27(±0.02)
Otter Lake	11/29/2022	4.21(±0.09)	0.95(±0.01)	1.26(±0.02)	3.98(±0.11)	1.19(±0.01)	0.63(±0.01)	0.67(±0.01)	0.31(±0.01)
Jockeybush Lake	11/29/2022	5.11(±0.01)	0.94(±0.01)	1.28(±0.01)	4.81(±0.06)	1.58(±0.02)	0.86(±0.01)	0.94(±0.01)	0.34(±0.01)
Avalanche Lake	6/2/2023	8.10(±0.10)	0.89(±0.01)	1.43(±0.02)	7.17(±0.10)	2.93(±0.03)	2.08(±0.01)	1.92(±0.01)	0.41(±0.01)
Lake Colden	6/2/2023	5.53(±0.25)	0.87(±0.01)	1.38(±0.01)	4.81(±0.21)	2.15(±0.02)	1.44(±0.01)	1.39(±0.02)	0.44(±0.01)
Brook Trout Lake	5/22/2023	5.60(±0.39)	0.90(±0.01)	1.30(±0.01)	5.03(±0.36)	1.74(±0.02)	0.99(±0.01)	0.97(±0.01)	0.37(±0.01)
Squash Pond Outlet	5/15/2023	11.41(±0.97)	0.88(±0.01)	1.68(±0.02)	10.05(±0.94)	4.40(±0.09)	3.92(±0.06)	3.78(±0.08)	0.51(±0.01)
Squash Pond	5/15/2023	10.20(±0.67)	0.86(±0.01)	1.62(±0.01)	8.82(±0.60)	5.96(±0.07)	4.94(±0.04)	4.91(±0.05)	0.79(±0.01)
Constable Pond Outlet	5/15/2023	8.65(±0.34)	0.90(±0.01)	1.43(±0.01)	7.77(±0.37)	3.52(±0.05)	2.57(±0.01)	2.29(±0.03)	0.45(±0.01)
Constable Pond	5/15/2023	9.30(±0.51)	0.89(±0.01)	1.46(±0.01)	8.27(±0.40)	4.11(±0.04)	2.96(±0.01)	2.95(±0.02)	0.55(±0.01)
East Copperas Pond	6/27/2023	6.43(±0.29)	0.89(±0.01)	1.41(±0.01)	5.72(±0.24)	4.80(±0.06)	3.36(±0.05)	2.30(±0.06)	0.85(±0.01)
Little Echo Pond	6/29/2023	6.97(±0.24)	0.96(±0.01)	1.56(±0.01)	6.72(±0.24)	4.30(±0.05)	3.61(±0.04)	2.10(±0.04)	0.52(±0.01)
Lake Rondaxe	6/19/2023	4.45(±0.17)	0.92(±0.01)	1.24(±0.02)	4.11(±0.13)	2.10(±0.02)	1.05(±0.01)	0.92(±0.01)	0.55(±0.01)
Cascade Lake	6/16/2023	3.76(±0.22)	0.91(±0.01)	1.18(±0.01)	3.42(±0.18)	1.46(±0.02)	0.70(±0.01)	0.63(±0.01)	0.45(±0.01)
Cascade Lake Outlet	6/16/2023	6.24(±0.43)	0.95(±0.01)	1.40(±0.01)	5.90(±0.36)	3.14(±0.04)	2.36(±0.03)	1.85(±0.03)	0.48(±0.01)
Dart Lake	5/23/2023	8.60(±1.31)	0.92(±0.01)	1.37(±0.01)	7.93(±1.19)	3.27(±0.03)	2.10(±0.01)	1.84(±0.03)	0.44(±0.02)
Woods Lake	6/16/2023	4.44(±0.25)	0.93(±0.01)	1.22(±0.01)	4.12(±0.22)	2.15(±0.05)	1.04(±0.01)	0.97(±0.01)	0.54(±0.01)
Willys Lake	5/23/2023	7.07(±0.47)	0.86(±0.01)	1.46(±0.02)	6.10(±0.41)	1.74(±0.01)	1.17(±0.01)	1.43(±0.01)	0.37(±0.01)
Loon Hollow Pond	5/23/2023	9.64(±0.66)	0.87(±0.01)	1.62(±0.01)	8.34(±0.52)	3.90(±0.02)	3.08(±0.03)	3.42(±0.04)	0.61(±0.01)
Little Clear Pond	6/22/2023	3.77(±0.32)	0.84(±0.01)	1.28(±0.01)	3.17(±0.25)	2.08(±0.01)	1.37(±0.02)	0.94(±0.02)	0.69(±0.03)
Black Pond	6/29/2023	3.75(±0.01)	0.88(±0.01)	1.18(±0.01)	3.29(±0.01)	1.41(±0.01)	0.77(±0.01)	0.58(±0.01)	0.44(±0.01)
Indian Lake	5/22/2023	9.63(±1.26)	0.89(±0.01)	1.48(±0.01)	8.53(±1.03)	3.67(±0.04)	2.81(±0.03)	2.54(±0.03)	0.46(±0.01)
Squaw Lake	5/22/2023	5.65(±0.27)	0.90(±0.01)	1.29(±0.01)	5.07(±0.28)	1.59(±0.01)	0.94(±0.01)	0.78(±0.01)	0.34(±0.01)
Queer Lake	6/6/2023	5.21(±0.07)	0.92(±0.01)	1.24(±0.01)	4.80(±0.01)	1.97(±0.02)	0.96(±0.01)	0.89(±0.01)	0.46(±0.01)
Big Hope Pond	6/23/2023	5.84(±0.15)	0.90(±0.01)	1.29(±0.01)	5.27(±0.17)	3.98(±0.03)	2.34(±0.02)	1.80(±0.02)	0.84(±0.01)
G Lake	6/29/2023	3.47(±0.20)	0.94(±0.01)	1.19(±0.01)	3.25(±0.16)	1.16(±0.01)	0.55(±0.01)	0.53(±0.01)	0.37(±0.01)
West Pond Outlet	6/16/2023	7.84(±0.17)	0.92(±0.02)	1.56(±0.02)	7.18(±0.02)	5.64(±0.04)	4.62(±0.05)	3.66(±0.03)	0.64(±0.01)
West Pond	6/16/2023	6.64(±0.01)	0.88(±0.01)	1.38(±0.01)	5.82(±0.01)	2.92(±0.01)	2.04(±0.01)	1.63(±0.01)	0.50(±0.01)
Big Moose Lake	6/16/2023	7.21(±0.23)	0.90(±0.01)	1.32(±0.01)	6.48(±0.20)	2.88(±0.01)	1.74(±0.01)	1.58(±0.01)	0.47(±0.01)
Moss Lake	5/25/2023	5.55(±0.13)	0.93(±0.01)	1.28(±0.01)	5.18(±0.07)	2.04(±0.02)	1.17(±0.01)	1.01(±0.01)	0.40(±0.01)
Bubb Lake	5/25/2023	3.92(±0.08)	0.89(±0.02)	1.21(±0.02)	3.48(±0.01)	1.04(±0.01)	0.59(±0.01)	0.48(±0.01)	0.33(±0.01)
Bubb Lake Outlet	5/25/2023	4.75(±0.25)	0.92(±0.01)	1.30(±0.01)	4.39(±0.21)	1.18(±0.01)	0.80(±0.01)	0.66(±0.01)	0.27(±0.01)
Limekiln Lake	6/6/2023	3.54(±0.03)	0.94(±0.01)	1.21(±0.01)	3.32(±0.05)	2.11(±0.01)	1.06(±0.01)	0.95(±0.01)	0.64(±0.01)
Raquette Lake Reservoir	5/23/2023	9.73(±0.25)	0.94(±0.01)	1.43(±0.01)	9.12(±0.15)	4.15(±0.02)	2.98(±0.01)	2.54(±0.01)	0.41(±0.01)
Sagamore Lake	6/20/2023	7.98(±0.15)	0.96(±0.01)	1.38(±0.01)	7.68(±0.10)	4.17(±0.01)	2.64(±0.01)	2.19(±0.01)	0.51(±0.01)
Arbutus Pond	6/23/2023	4.81(±0.05)	0.91(±0.01)	1.23(±0.01)	4.36(±0.02)	2.16(±0.01)	1.22(±0.01)	0.95(±0.01)	0.54(±0.01)

**Table S4.** Optical properties of whole water samples from ALTM lakes (continued)									
Site Name	Sampling Date	Peak A : Peak T	Peak C : Peak A	Peak C : Peak M	Peak C : Peak T	PARAFAC C1 (R.U.)	PARAFAC C2 (R.U.)	PARAFAC C3 (R.U.)	PARAFAC C4 (R.U.)
Clear Pond	6/23/2023	3.56(±0.01)	0.86(±0.02)	1.16(±0.03)	3.08(±0.07)	1.20(±0.01)	0.63(±0.01)	0.49(±0.01)	0.44(±0.01)
Heart Lake	6/13/2023	2.64(±0.06)	0.88(±0.02)	1.19(±0.01)	2.31(±0.01)	0.65(±0.01)	0.38(±0.01)	0.34(±0.01)	0.28(±0.01)
Grass Pond	5/16/2023	5.92(±0.51)	0.97(±0.01)	1.33(±0.01)	5.71(±0.42)	1.99(±0.01)	1.17(±0.01)	1.12(±0.01)	0.34(±0.01)
Middle Branch Lake	5/16/2023	6.25(±0.41)	0.93(±0.01)	1.32(±0.02)	5.80(±0.29)	1.93(±0.02)	1.21(±0.01)	0.98(±0.01)	0.35(±0.01)
Middle Settlement Lake	5/16/2023	5.26(±0.05)	0.93(±0.01)	1.30(±0.03)	4.91(±0.12)	1.72(±0.01)	1.00(±0.01)	0.92(±0.01)	0.35(±0.01)
South Lake	6/26/2023	4.45(±0.28)	0.92(±0.01)	1.24(±0.01)	4.11(±0.27)	1.45(±0.01)	0.75(±0.01)	0.71(±0.01)	0.38(±0.01)
North Lake	6/26/2023	7.20(±0.41)	0.91(±0.01)	1.35(±0.01)	6.57(±0.35)	2.67(±0.01)	1.67(±0.01)	1.42(±0.01)	0.43(±0.01)
Otter Lake Outlet	6/5/2023	5.48(±0.02)	0.91(±0.01)	1.27(±0.01)	4.98(±0.05)	1.32(±0.01)	0.75(±0.01)	0.73(±0.01)	0.29(±0.01)
Otter Lake	6/5/2023	5.00(±0.19)	0.90(±0.01)	1.25(±0.02)	4.48(±0.20)	1.41(±0.01)	0.79(±0.01)	0.76(±0.01)	0.34(±0.01)
Jockeybush Lake	6/5/2023	4.20(±0.17)	0.93(±0.01)	1.22(±0.01)	3.90(±0.10)	1.38(±0.01)	0.72(±0.01)	0.69(±0.01)	0.38(±0.01)
Avalanche Lake	9/25/2023	11.14(±0.36)	0.93(±0.01)	1.50(±0.01)	10.33(±0.48)	4.19(±0.01)	2.98(±0.01)	3.09(±0.05)	0.43(±0.02)
Lake Colden	9/25/2023	8.60(±0.10)	0.93(±0.01)	1.45(±0.01)	8.00(±0.16)	3.10(±0.01)	2.12(±0.01)	2.22(±0.01)	0.40(±0.01)
Brook Trout Lake	9/5/2023	4.80(±0.09)	0.88(±0.01)	1.24(±0.01)	4.23(±0.07)	1.64(±0.01)	0.89(±0.01)	0.87(±0.01)	0.43(±0.01)
Squash Pond Outlet	9/18/2023	10.43(±0.01)	0.90(±0.01)	1.65(±0.01)	9.39(±0.04)	4.25(±0.03)	3.65(±0.02)	3.75(±0.02)	0.55(±0.01)
Squash Pond	9/18/2023	8.30(±0.10)	0.87(±0.01)	1.61(±0.01)	7.25(±0.09)	6.42(±0.02)	4.92(±0.03)	5.46(±0.03)	1.06(±0.01)
Constable Pond Outlet	9/18/2023	6.84(±0.39)	0.92(±0.01)	1.39(±0.01)	6.29(±0.38)	3.88(±0.01)	2.52(±0.01)	2.39(±0.01)	0.56(±0.01)
Constable Pond	9/18/2023	7.45(±0.04)	0.90(±0.01)	1.39(±0.01)	6.72(±0.01)	4.00(±0.01)	2.54(±0.01)	2.68(±0.01)	0.63(±0.01)
East Copperas Pond	9/15/2023	7.39(±0.09)	0.90(±0.01)	1.45(±0.01)	6.63(±0.16)	5.19(±0.03)	3.68(±0.01)	2.59(±0.02)	0.80(±0.01)
Little Echo Pond	9/21/2023	8.75(±0.25)	1.00(±0.01)	1.63(±0.01)	8.74(±0.17)	6.06(±0.01)	5.19(±0.01)	3.04(±0.01)	0.48(±0.01)
Lake Rondaxe	9/23/2023	6.05(±0.08)	0.94(±0.01)	1.26(±0.01)	5.71(±0.09)	2.63(±0.01)	1.35(±0.01)	1.21(±0.01)	0.51(±0.01)
Cascade Lake	9/18/2023	4.30(±0.03)	0.91(±0.01)	1.16(±0.01)	3.93(±0.02)	1.60(±0.01)	0.72(±0.01)	0.70(±0.01)	0.48(±0.01)
Cascade Lake Outlet	9/18/2023	6.52(±0.05)	0.93(±0.01)	1.34(±0.01)	6.07(±0.05)	2.51(±0.01)	1.72(±0.01)	1.43(±0.01)	0.42(±0.01)
Dart Lake	9/22/2023	6.31(±0.31)	0.90(±0.01)	1.29(±0.01)	5.69(±0.25)	3.00(±0.01)	1.76(±0.01)	1.56(±0.01)	0.57(±0.01)
Woods Lake	9/18/2023	6.41(±0.38)	0.93(±0.01)	1.31(±0.01)	5.96(±0.31)	3.51(±0.02)	1.99(±0.01)	1.86(±0.01)	0.61(±0.01)
Willys Lake	9/19/2023	5.95(±0.34)	0.86(±0.01)	1.40(±0.02)	5.10(±0.27)	1.59(±0.01)	1.06(±0.01)	1.19(±0.01)	0.39(±0.01)
Loon Hollow Pond	9/19/2023	8.57(±0.17)	0.89(±0.01)	1.59(±0.01)	7.58(±0.16)	4.56(±0.03)	3.52(±0.01)	3.85(±0.01)	0.74(±0.01)
Little Clear Pond	9/12/2023	4.75(±0.19)	0.85(±0.01)	1.28(±0.01)	4.04(±0.16)	2.16(±0.02)	1.46(±0.01)	1.02(±0.01)	0.60(±0.01)
Black Pond	9/21/2023	5.50(±0.08)	0.90(±0.01)	1.24(±0.01)	4.95(±0.02)	1.78(±0.02)	1.03(±0.01)	0.78(±0.01)	0.42(±0.01)
Indian Lake	9/5/2023	9.67(±0.16)	0.92(±0.01)	1.53(±0.01)	8.87(±0.09)	4.73(±0.02)	3.56(±0.02)	3.45(±0.03)	0.54(±0.01)
Squaw Lake	9/5/2023	4.13(±0.15)	0.86(±0.01)	1.20(±0.01)	3.57(±0.09)	1.29(±0.01)	0.71(±0.01)	0.58(±0.01)	0.42(±0.01)
Queer Lake	9/13/2023	4.33(±0.16)	0.90(±0.01)	1.19(±0.02)	3.90(±0.09)	1.58(±0.02)	0.74(±0.01)	0.66(±0.01)	0.46(±0.01)
Big Hope Pond	9/21/2023	6.70(±0.33)	0.89(±0.01)	1.29(±0.01)	5.98(±0.30)	4.06(±0.04)	2.45(±0.01)	1.94(±0.03)	0.79(±0.01)
G Lake	9/12/2023	3.66(±0.09)	0.93(±0.01)	1.17(±0.03)	3.39(±0.06)	1.10(±0.01)	0.51(±0.01)	0.49(±0.01)	0.36(±0.01)
West Pond Outlet	9/4/2023	10.19(±0.36)	0.95(±0.01)	1.63(±0.01)	9.67(±0.37)	8.23(±0.01)	6.73(±0.01)	5.29(±0.01)	0.65(±0.01)
West Pond	9/4/2023	7.40(±0.33)	0.88(±0.01)	1.40(±0.01)	6.55(±0.30)	3.87(±0.01)	2.68(±0.01)	2.16(±0.01)	0.60(±0.01)
Big Moose Lake	9/4/2023	7.73(±0.05)	0.91(±0.01)	1.32(±0.01)	7.07(±0.07)	3.40(±0.01)	2.07(±0.01)	1.80(±0.01)	0.55(±0.01)
Moss Lake	9/26/2023	5.09(±0.06)	0.93(±0.01)	1.22(±0.01)	4.74(±0.11)	1.98(±0.01)	0.99(±0.01)	0.84(±0.01)	0.48(±0.01)
Bubb Lake	9/4/2023	2.81(±0.15)	0.85(±0.01)	1.14(±0.01)	2.38(±0.11)	0.81(±0.01)	0.48(±0.01)	0.40(±0.01)	0.38(±0.01)

**Table S4.** Optical properties of whole water samples from ALTM lakes (continued)									
Site Name	Sampling Date	Peak A : Peak T	Peak C : Peak A	Peak C : Peak M	Peak C : Peak T	PARAFAC C1 (R.U.)	PARAFAC C2 (R.U.)	PARAFAC C3 (R.U.)	PARAFAC C4 (R.U.)
Bubb Lake Outlet	9/4/2023	4.10(±0.18)	0.88(±0.01)	1.21(±0.01)	3.61(±0.19)	0.99(±0.01)	0.64(±0.01)	0.55(±0.01)	0.31(±0.01)
Limekiln Lake	9/4/2023	5.14(±0.17)	0.89(±0.01)	1.21(±0.02)	4.60(±0.11)	1.57(±0.01)	0.83(±0.01)	0.76(±0.01)	0.43(±0.01)
Raquette Lake Reservoir	9/26/2023	9.47(±0.15)	0.96(±0.01)	1.44(±0.01)	9.06(±0.06)	6.85(±0.01)	4.54(±0.01)	3.80(±0.02)	0.62(±0.01)
Sagamore Lake	9/29/2023	10.51(±0.45)	0.93(±0.01)	1.48(±0.01)	9.73(±0.48)	6.85(±0.02)	5.22(±0.02)	4.33(±0.02)	0.68(±0.01)
Arbutus Pond	9/26/2023	7.35(±0.13)	0.92(±0.01)	1.30(±0.01)	6.76(±0.03)	3.82(±0.01)	2.27(±0.01)	1.82(±0.01)	0.66(±0.01)
Clear Pond	9/26/2023	5.24(±0.15)	0.90(±0.01)	1.22(±0.01)	4.72(±0.12)	1.83(±0.01)	1.01(±0.01)	0.80(±0.01)	0.46(±0.01)
Heart Lake	9/5/2023	3.05(±0.10)	0.87(±0.01)	1.19(±0.01)	2.64(±0.11)	0.63(±0.01)	0.38(±0.01)	0.31(±0.01)	0.26(±0.01)
Grass Pond	9/4/2023	8.43(±0.59)	0.95(±0.02)	1.38(±0.01)	8.01(±0.42)	3.35(±0.01)	2.14(±0.01)	2.19(±0.03)	0.41(±0.01)
Middle Branch Lake	9/4/2023	7.13(±0.02)	0.93(±0.01)	1.33(±0.01)	6.62(±0.01)	2.63(±0.01)	1.60(±0.01)	1.27(±0.01)	0.45(±0.01)
Middle Settlement Lake	9/4/2023	6.10(±0.19)	0.94(±0.01)	1.27(±0.01)	5.74(±0.11)	2.18(±0.01)	1.30(±0.01)	1.18(±0.02)	0.42(±0.01)
South Lake	9/6/2023	5.52(±0.02)	0.92(±0.01)	1.25(±0.01)	5.10(±0.08)	1.80(±0.01)	0.94(±0.01)	0.87(±0.01)	0.40(±0.01)
North Lake	9/6/2023	9.16(±0.70)	0.91(±0.01)	1.45(±0.01)	8.31(±0.68)	4.48(±0.01)	3.38(±0.01)	2.90(±0.01)	0.56(±0.01)
Otter Lake Outlet	9/14/2023	5.25(±0.26)	0.95(±0.01)	1.29(±0.02)	5.00(±0.29)	1.46(±0.01)	0.86(±0.01)	0.88(±0.01)	0.32(±0.01)
Otter Lake	9/14/2023	3.75(±0.14)	0.92(±0.02)	1.21(±0.01)	3.46(±0.05)	1.26(±0.01)	0.64(±0.01)	0.64(±0.01)	0.40(±0.01)
Jockeybush Lake	9/12/2023	4.50(±0.22)	0.93(±0.01)	1.23(±0.01)	4.19(±0.18)	1.56(±0.01)	0.84(±0.01)	0.79(±0.01)	0.40(±0.01)

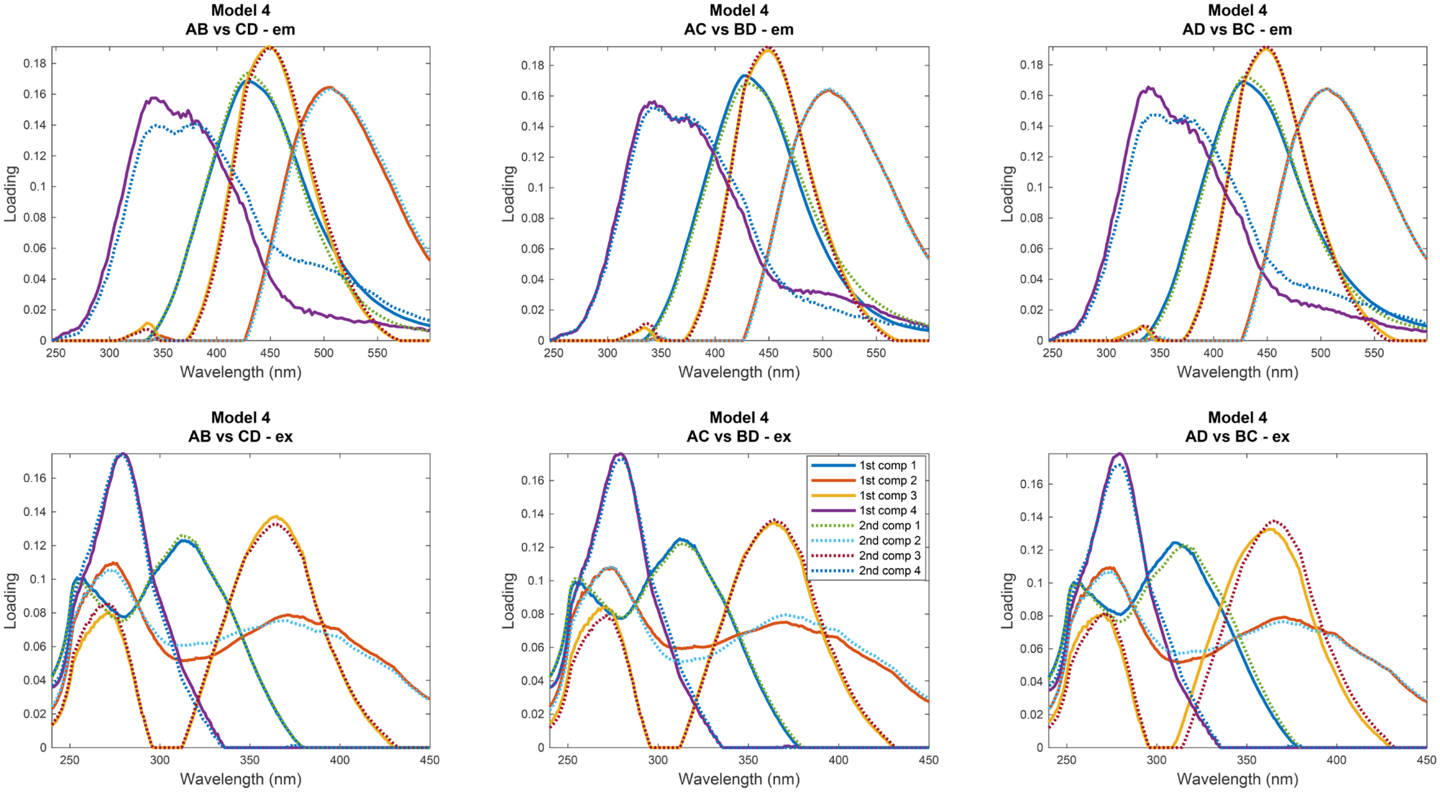

Figure S2. Split-half comparisons of the excitation and emission spectra from the 4-component PARAFAC model.

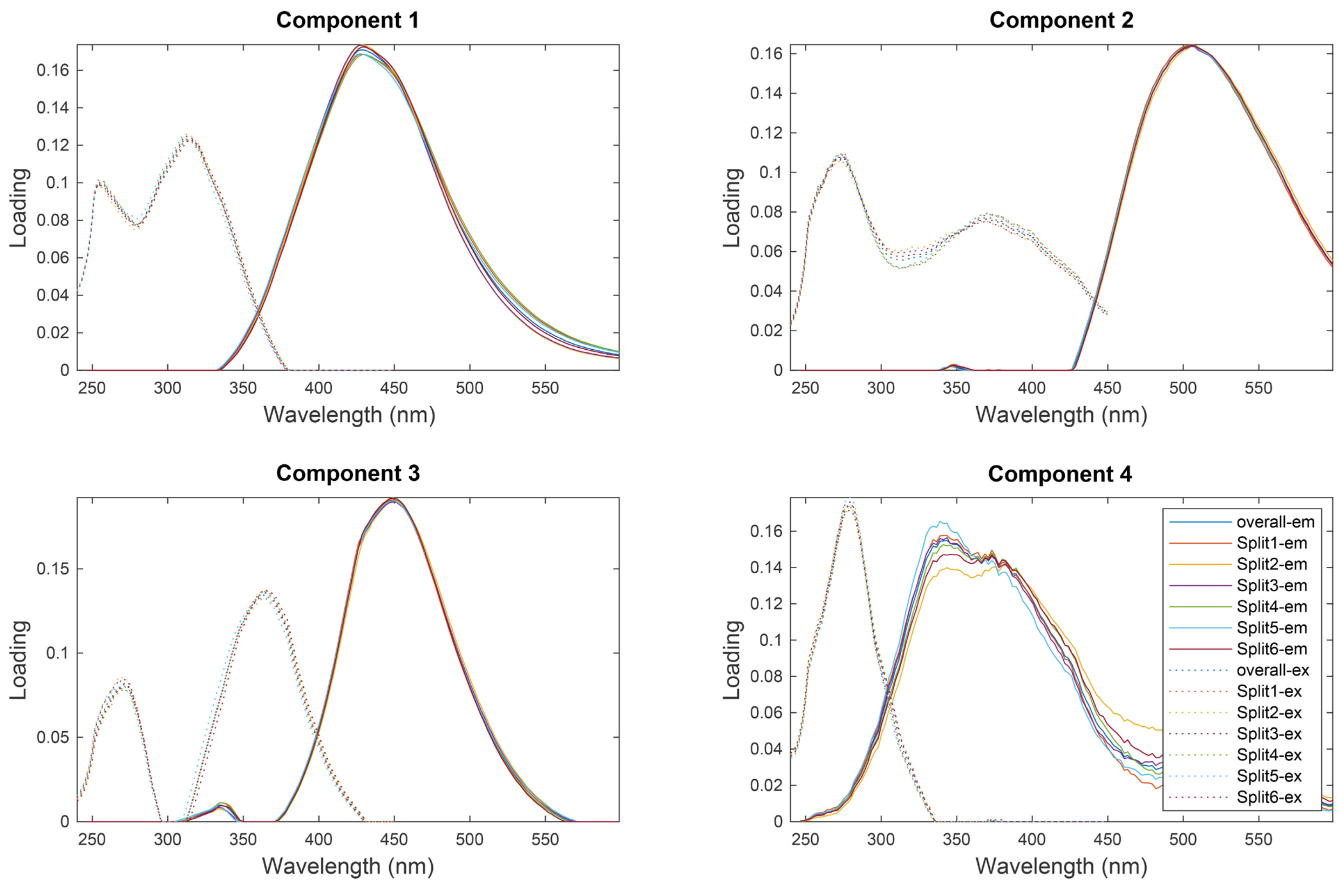

Figure S3. Overlaid spectral loadings of the 4-component PARAFAC model versus the overall model.

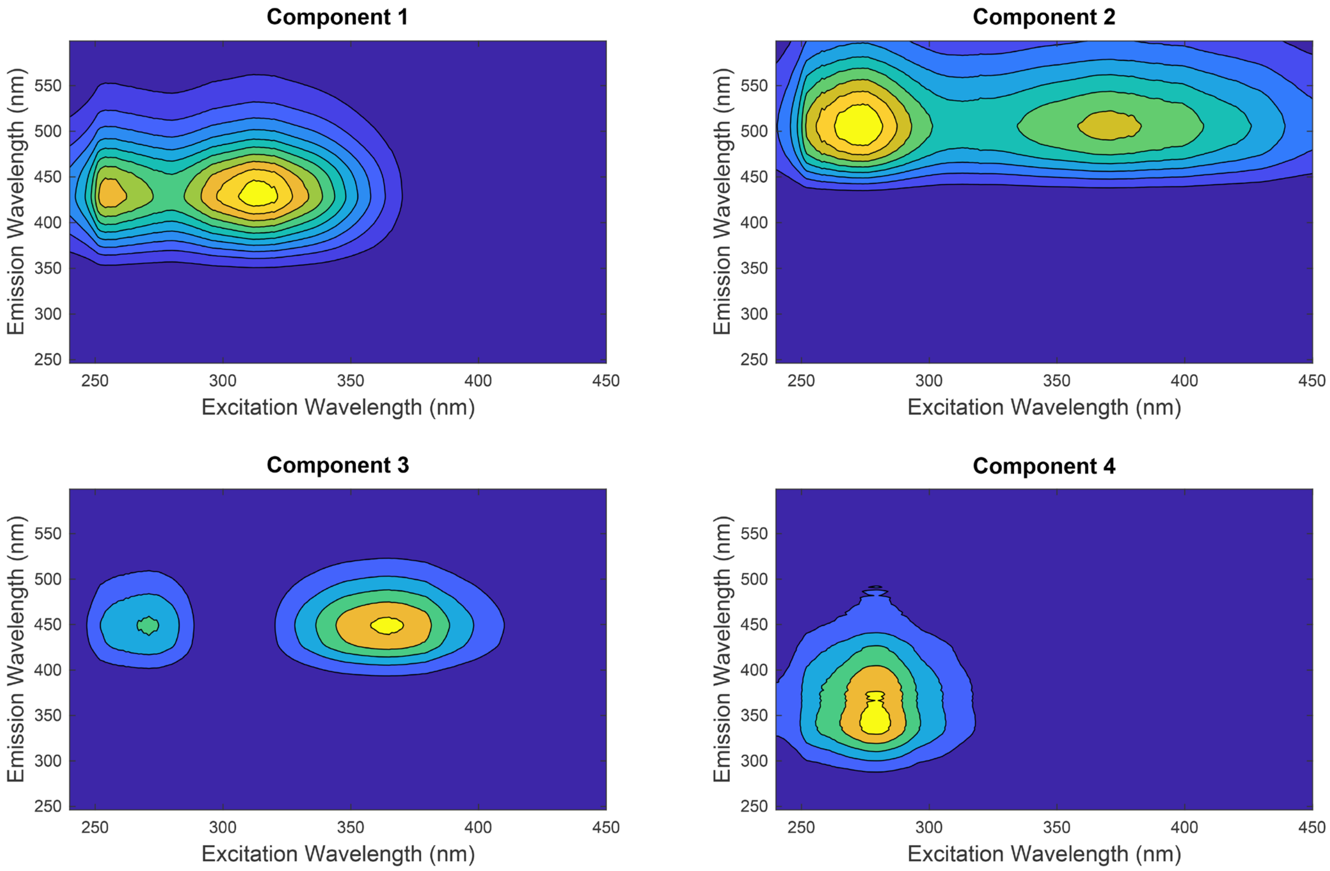

Figure S4. Contour plots of 4 fluorescent components validated for the 4-component PARAFAC model.

6. *p*-Nitroanisole/pyridine actinometry

For each set of photochemistry experiments, 500 μL of sample aliquots were withdrawn from quartz test tubes over one half-life and analyzed for the concentrations of PNA by an Agilent 1260 Infinity II high-performance liquid chromatograph with a variable wavelength detector and a fluorescence detector. For each sample, the disappearance of PNA in 10 μM PNA/5 mM pyr actinometer solutions was monitored to determine the pseudo-first order rate constant for PNA loss, $k_{obs,PNA}$ (s^{-1}) for calculating the rate of light absorption R_a (mol-photons L^{-1} s^{-1}) per unit sample volume as described in previous work:[1, 2, 27-29]

$$R_{loss,\,PNA} = -\frac{d[PNA]}{dt} = k_{obs,\,PNA}[PNA] = \Phi_{PNA}\sum_{\lambda\,=\,290\text{ nm}}^{550\text{ nm}}\frac{W_{\lambda}\varepsilon_{\lambda}[1-10^{-\alpha_{D(\lambda)}z}]}{z\alpha_{\lambda}}[PNA]$$
$$\approx \Phi_{PNA}\sum_{\lambda\,=\,290\text{ nm}}^{550\text{ nm}}I_{\lambda}\frac{\varepsilon_{\lambda}[1-10^{-\alpha_{\lambda}z}]}{\alpha_{\lambda}}[PNA] \qquad \text{(S1)}$$

where $R_{loss,\,PNA}$ (M s^{-1}) is the loss rate of PNA, [PNA] is the concentration of PNA, Φ_{PNA} (1.74×10^{-3} mol mol-photons^{-1}; calculated from Φ_{PNA} = 0.29 [pyr] + 0.00029) is the quantum yield for the loss of PNA at a given pyridine concentration (i.e., [pyr] = 5×10^{-3} M),[2] W_{λ} (10^{-3} mol-photons cm^{-2} s^{-1} nm^{-1}) is the incident light intensity at a given wavelength λ, ε_{λ} (M^{-1} cm^{-1}) is the decadic molar absorption coefficient of PNA at a given wavelength λ,[2] $\alpha_{D(\lambda)}$ (cm^{-1}) is the apparent (or diffuse) attenuation coefficient ($\alpha_{D(\lambda)}\approx\alpha_{\lambda}$ where the distribution function D(λ) is ~1.0 for the quartz test tube[28]), z (1.12 cm) is the optical pathlength for the quartz test tube,[27] α_{λ} (cm^{-1}) is the decadic absorption (or attenuation) coefficient (i.e., the absorbance divided by the optical pathlength of the quartz cuvette), I_{λ} (10^{-3} mol-photons cm^{-3} s^{-1} nm^{-1} or mol-photons L^{-1} s^{-1} nm^{-1}) is the incident light intensity at a given wavelength λ per unit volume, and 290-550 nm is the integration range chosen based on the wavelength dependence of $\Phi_{app,\,{}^1O_2}$.[30, 31]

Equation S1 was re-written given that I_{λ} can be approximated by multiplying the fractional spectral intensity of the xenon arc lamp, ρ_{λ} (nm^{-1}), with the total incident light intensity from 290 to 550 nm per unit volume, I_0 (mol-photons L^{-1} s^{-1}; the wavelength range was selected to account for PNA absorbance past 400 nm[32]):[2, 33, 34]

$$k_{\text{obs, PNA}} = 2.303z\Phi_{\text{PNA}} \sum_{\lambda = 290\text{ nm}}^{550\text{ nm}} \rho_{\lambda} I_0 \varepsilon_{\lambda} = 2.303z\Phi_{\text{PNA}} I_0 \sum_{\lambda = 290\text{ nm}}^{550\text{ nm}} \rho_{\lambda} \varepsilon_{\lambda} \qquad \text{(S2)}$$

Equation S2 was further re-arranged to calculate I_0, assuming that I_0 measured by the PNA/pyr actinometer represented I_0 through a given sample:[2, 33, 34]

$$I_0 = \frac{k_{\text{obs, PNA}}}{2.303z\Phi_{\text{PNA}} \sum_{\lambda = 290\text{ nm}}^{550\text{ nm}} \rho_{\lambda} \varepsilon_{\lambda}} \qquad \text{(S3)}$$

Equation S3 was used to calculate R_a based on the sample-specific α_{λ} (m^{-1}):[33, 34]

$$R_a = \sum_{\lambda = 290\text{ nm}}^{550\text{ nm}} \frac{W_{\lambda}(1 - 10^{-\alpha_{\text{D},\lambda} z})}{z} \approx \sum_{\lambda = 290\text{ nm}}^{550\text{ nm}} \rho_{\lambda} I_0 \,(1 - 10^{-\alpha_{\lambda} z}) \qquad \text{(S4)}$$

7. Measurements of the apparent quantum yields of $^1O_2$

For each set of photochemistry experiments, 500 μL of sample aliquots were withdrawn from quartz test tubes over one half-life and analyzed for the concentrations of FFA by an Agilent 1260 Infinity II high-performance liquid chromatograph with a variable wavelength detector and a fluorescence detector. For each sample, the disappearance of FFA was monitored to determine the pseudo-first order rate constant for FFA loss in the presence of 0.1 mM of methanol (to quench hydroxyl radicals[35]), $k_{\text{obs, FFA}}$ (s^{-1}), with the contributions from the apparent direct photolysis of FFA caused by impurities as described in previous work:[36, 37]

$$R_{\text{loss, FFA}} = -\frac{\text{d[FFA]}}{\text{dt}} = k_{\text{obs, FFA}}[\text{FFA}] = k_{\text{FFA, }^1\text{O}_2}[\text{FFA}][^1\text{O}_2]_{\text{ss}} + k_{\text{direct photolysis, FFA}}[\text{FFA}]SF_{\Sigma\lambda} \quad \text{(S5)}$$

where $R_{\text{loss, FFA}}$ (M s^{-1}) is the loss rate of FFA, [FFA] is the initial concentration of FFA (10 μM), $k_{\text{FFA, }^1\text{O}_2}$ (M^{-1} s^{-1}) is the second-order reaction rate constant of FFA with $^1O_2$, and $[^1O_2]_{ss}$ is the steady-state concentration of $^1O_2$ in the quartz test tube, $k_{\text{direct photolysis, FFA}}$ (s^{-1}) is the apparent direct photolysis rate constant of FFA, and $SF_{\Sigma\lambda}$ is the sample-specific light screening factor.

To account for the potential effect of temperature on the $^1O_2$ reaction kinetics of FFA, a temperature-adjusted $k_{\text{FFA, }^1\text{O}_2}$ of 1.06(±0.07)×10^8 M^{-1} s^{-1} was derived by substituting T = 25 °C (i.e., the solar simulator chamber temperature) into $k_{\text{FFA, }^1\text{O}_2} = (1.00 \pm 0.04) \times 10^8\ M^{-1}\ s^{-1} + [(2.1 \pm 0.3) \times 10^6\ M^{-1}\ s^{-1}\ °C^{-1}] \times (T - 22\ °C)$.[37]

For each sample, the steady-state concentration of $^1O_2$, $[^1O_2]_{ss}$, was calculated as:[33, 36-38]

$$[^1\text{O}_2]_{\text{ss}} = \frac{R_{\text{f, }^1\text{O}_2}}{k_{\text{d}}^{\Delta}} = \frac{(k_{\text{obs, FFA}} - k_{\text{direct photolysis, FFA}}SF_{\Sigma\lambda})(k_{\text{d}}^{\Delta} + k_{\text{FFA, }^1\text{O}_2}[\text{FFA}])}{k_{\text{d}}^{\Delta}k_{\text{FFA, }^1\text{O}_2}} \approx \frac{(k_{\text{obs, FFA}} - k_{\text{direct photolysis, FFA}}SF_{\Sigma\lambda})}{k_{\text{FFA, }^1\text{O}_2}} \quad \text{(S6)}$$

where $R_{\text{f, }^1\text{O}_2}$ (M s^{-1}) is the formation rate of $^1O_2$ and k_{d}^{Δ} (2.78(±0.03)×10^5 s^{-1}; temperature-adjusted for T = 25 °C in the solar simulator chamber[37]) is the pseudo-first order deactivation rate constant of $^1O_2$ by water.

The apparent quantum yield of $^1O_2$, $\Phi_{\text{app, }^1\text{O}_2}$ (mol mol-photons^{-1}), was calculated as:[33, 38]

$$\Phi_{\mathrm{app},\,^1\mathrm{O}_2} = \frac{R_{\mathrm{f},\,^1\mathrm{O}_2}}{R_\mathrm{a}} = \frac{(k_\mathrm{d}^{\Delta} + k_{\mathrm{FFA},\,^1\mathrm{O}_2}[\mathrm{FFA}])[^1\mathrm{O}_2]_\mathrm{ss}}{R_\mathrm{a}} \approx \frac{k_\mathrm{d}^{\Delta}[^1\mathrm{O}_2]_\mathrm{ss}}{R_\mathrm{a}} \tag{S7}$$

8. Calculations of steady-state concentrations of $^1O_2$

To estimate the depth-averaged steady-state concentrations of $^1O_2$ in the euphotic zone of a lake on a given sampling date, Equation S8 was applied with corrections for non-clear-sky conditions:[39-41]

$$[^1O_2]^{\text{euphotic zone}}_{\text{ss, daily average}} = \frac{R^{\text{euphotic zone}}_{\text{f, }^1O_2}}{k^{\Delta}_{\text{d}}} = \frac{\Phi_{\text{app, }^1O_2} R^{\text{euphotic zone}}_{\text{a, daily average}}}{k^{\Delta}_{\text{d}}}$$

$$= \frac{\Phi_{\text{app, }^1O_2}}{k^{\Delta}_{\text{d}}} \times CF \times \sum_{\lambda\,=\,290\text{ nm}}^{550\text{ nm}} \frac{Z_{\lambda,\text{ daily average}}}{z_{\text{euphotic zone}}} \times \left(1 - e^{-K_{\text{d, }\lambda} z_{\text{euphotic zone}}}\right) \times \left(1 - f_{\text{backscatter}}\right) \times f_{\text{abs,CDOM}} \quad \text{(S8)}$$

where $\Phi_{\text{app, }^1O_2}$ (mol mol-photons^{-1}) is the apparent quantum yield of $^1O_2$ measured by FFA,[30, 40] k^{Δ}_{d} (2.74(±0.05)×10^5 s^{-1} averaged over the range of pH and water temperature measured for ALTM samples) is the pseudo-first order deactivation rate constant of $^1O_2$ by water,[37] CF (i.e., 0.50-0.67) is the non-clear-sky correction factor for solar irradiance,[41] $Z_{\lambda,\text{ daily average}}$ is the site-specific daily average solar irradiance at a given wavelength λ (10^{-3} mol-photons cm^{-2} s^{-1} nm^{-1}) modeled using the *Simple Model of the Atmospheric Radiative Transfer of Sunshine (SMARTS) v2.9.9*[42-44] with adjustments made for reflection off the water's surface and increased pathlength within the water column,[27, 39, 45] $z_{\text{euphotic zone}}$ (cm) is the euphotic zone depth estimated based on the relationship previously established for Adirondack lakes (i.e., $z_{\text{euphotic zone}} = 4.6 / [0.15[\text{DOC}]^{1.08}] \times 100$),[46] $K_{\text{d},\lambda}$ is the diffuse attenuation coefficient (cm^{-1}) modeled based on [DOC] (mg C/L) using the relationship proposed by Morris *et al.* ($K_{\text{d},\lambda} = \exp(-0.01347\lambda + 5.36[\text{DOC}]^{0.157})$),[47] $f_{\text{backscatter}}$ (0.03) is the fraction of sunlight backscattered out of the water column,[40] and $f_{\text{abs,CDOM}}$ (0.7) is the fraction of lake water absorbance attributable to colored DOM.[40]

To account for the reflected fraction and the increased pathlength, $Z_{\lambda,\text{ daily average}}$ was calculated as:[39, 45]

$$Z_{\lambda,\text{ daily average}} = W_{\lambda,\text{ daily average, direct}}\left(1 - RF_{\text{direct}}\right)\sec\theta + 1.2 W_{\lambda,\text{ daily average, diffuse}}\left(1 - RF_{\text{diffuse}}\right) \quad \text{(S9)}$$

where $W_{\lambda,\text{ daily average, direct}}$ (10^{-3} mol-photons cm^{-2} s^{-1} nm^{-1}) is the direct beam irradiance at a given wavelength λ, RF_{direct} is the reflected fraction of direct sunlight calculated using the Fresnel's law (= 0.5[sin(SZA −

$\theta) / \sin(\text{SZA} + \theta)]^2 + 0.5[\tan(\text{SZA} - \theta) / \tan(\text{SZA} + \theta)]^2$ with the solar zenith angle (SZA) and θ), θ is the refraction angle calculated using the Snell's law (= $\arcsin(1/1.34 \times \sin \text{SZA})$ with the SZA and the refractive index of water set at 1.335[48]), $W_{\lambda,\text{ daily average, diffuse}}$ (10^{-3} mol-photons cm^{-2} s^{-1} nm^{-1}) is the diffuse (or sky) irradiance at a given wavelength λ, and RF_{diffuse} (0.07) is the average reflected fraction of diffuse sunlight assuming a uniformly bright sky.[45]

To determine $W_{\lambda,\text{ daily average, direct}}$ and $W_{\lambda,\text{ daily average, diffuse}}$ for each site, SMARTS was implemented in batch mode using *Python* (e.g., **Table S5**) to generate outputs for SZA, the clearness index, and site-specific global, direct, and diffuse horizontal irradiance.

Table S5. Example SMARTS input parameters

**Card 1:** Adirondack Long Term Monitoring (ALTM) lakes	**Card 10:** Regional albedo: Water
**Card 2:** Site pressure from Altitude: 0.873 km; Height: 0 km; Latitude: +44.13056	**Card 10b:** Tilted surface & Local albedo: Bypass
**Card 3:** Reference atmosphere: MidLatitude Summer / Winter	**Card 11:** Spectral range: 280 – 800 nm; Solar constant: 1361.1 W/m^2;[49] Solar constant distance correction factor: 1.0
**Card 4:** Precipitable water: 1.715 cm[50]	**Card 12:** Spectral range to be printed: 280 – 800 nm at 1 nm intervals; Spectral results: Global horizontal irradiance; Direct horizontal irradiance; Diffuse horizontal irradiance (W m^{-2} nm^{-1})
**Card 5:** Ozone vertical column: 0.3073 atm-cm (i.e., 307.3 Dobson units);[51] Altitude of reading: Site level	**Card 13:** Circumsolar calculations: Bypass
**Card 6:** Gaseous Absorption and Pollution: Light Pollution	**Card 14:** Extra scanning/smoothing: Bypass
**Card 7:** Carbone dioxide concentration: 415.74 ppmv[52]	**Card 15:** Extra illuminance and photosynthetically active radiation calculations: Bypass
**Card 7a:** Extraterrestrial spectrum: Gueymard 2018[49, 53]	**Card 16:** Extra UV calculations: Bypass
**Card 8:** Aerosol model: Shettle & Fenn Rural[54]	**Card 17:** Solar position and air: Year: 2022; Month: 10; Day: 17; Hour: 12.619 (Local Standard Time); Latitude: +44.13056; Longitude: -73.96983; Time zone: -5
**Card 9:** Atmospheric turbidity: 0.071 specified as Aerosol Optical Depth at 500 nm[50]	

To estimate the depth-averaged steady-state concentrations of $^1O_2$ in the epilimnion of a lake on a given sampling date, Equation S10 was applied with corrections for non-clear-sky conditions:

$$[^1O_2]^{\text{epilimnion}}_{\text{ss, daily average}} = \frac{R^{\text{epilimnion}}_{\text{f, }^1O_2}}{k^{\Delta}_{\text{d}}} = \frac{\Phi_{\text{app, }^1O_2} R^{\text{epilimnion}}_{\text{a, daily average}}}{k^{\Delta}_{\text{d}}}$$

$$= \frac{\Phi_{\text{app, }^1O_2}}{k^{\Delta}_{\text{d}}} \times CF \times \sum_{\lambda = 290\text{ nm}}^{550\text{ nm}} \frac{Z_{\lambda,\text{ daily average}}}{z_{\text{epilimnion}}} \times (1 - e^{-K_{\text{d},\lambda} z_{\text{epilimnion}}}) \times \left(1 - f_{\text{backscatter}}\right) \times f_{\text{abs,CDOM}} \quad \text{(S10)}$$

where $z_{epilimnion}$ (cm) is the epilimnion depth estimated based on lake surface area (A; km^2) using the relationship developed by Hanna ($z_{epilimnion} = [(10^{0.185\log(A)+0.842} - 2.37) / 1.05] \times 100$).[55]

To estimate the near-surface steady-state concentrations of $^1O_2$ in a lake on a given sampling date, Equation S11 was applied with corrections for non-clear-sky conditions:[39-41]

$$[^1O_2]_{ss,\text{ daily average}}^{\text{near-surface}} = \frac{R_{f,\,^1O_2}^{\text{near-surface}}}{k_d^{\Delta}} = \frac{\Phi_{app,\,^1O_2} R_{a,\text{ daily average}}^{\text{near-surface}}}{k_d^{\Delta}}$$

$$= \frac{\Phi_{app,\,^1O_2}}{k_d^{\Delta}} \times CF \times \sum_{\lambda = 290\text{ nm}}^{550\text{ nm}} \frac{Z_{\lambda,\text{ daily average}}}{z_{1\text{ cm}}} \times (1 - e^{-K_{d,\lambda} z_{1\text{ cm}}}) \times (1 - f_{backscatter}) \times f_{abs,CDOM} \quad \text{(S11)}$$

where $z_{1\text{ cm}}$ (1 cm) is the thickness of the near-surface layer.

To estimate the depth-dependent steady-state concentrations of $^1O_2$ in the euphotic zone of a lake on a given sampling date, Equation S12 was applied with corrections for non-clear-sky conditions:[39-41]

$$[^1O_2]_{ss,\text{ daily average}}^{\text{depth}} = \frac{R_{f,\,^1O_2}^{\text{depth}}}{k_d^{\Delta}} = \frac{\Phi_{app,\,^1O_2} R_{a,\text{ daily average}}^{\text{depth}}}{k_d^{\Delta}}$$

$$= \frac{\Phi_{app,\,^1O_2}}{k_d^{\Delta}} \times CF \times \sum_{\lambda = 290\text{ nm}}^{550\text{ nm}} \frac{Z_{\lambda,\text{ daily average}} \times e^{-K_{d,\lambda} z_{depth}}}{\Delta z_{1\text{ cm}}} \times (1 - e^{-K_{d,\lambda} \Delta z_{1\text{ cm}}}) \times (1 - f_{backscatter}) \times f_{abs,CDOM} \quad \text{(S12)}$$

where z_{depth} (cm) is the depth of interest in the euphotic zone and $\Delta z_{1\text{ cm}}$ (1 cm interval) is the pathlength at each depth.

9. Wavelet coherences between the time series of DOC in ALTM lakes and external drivers

Table S6. Summary statistics of wavelet coherence tests

Response Variable – Driver	Timescale Band	Coherence Magnitude	Mean Phase	Band-aggregated *p*-value	Phase Classification[a]
Color – DOC (Figure S5)	Short (3-6 months)	0.520 – 0.589	0.060	<0.0001	Positive (in-phase)
	Intermediate (12-24 months)	0.527 – 0.884	0.013	<0.0001	Positive (in-phase)
	Long (36-72 months)	0.540 – 0.741	0.160	0.006	Positive (in-phase)
DOC – SO_4 + NO_3 Deposition (Figure S6)	Short (3-6 months)	0.067 – 0.226	1.376	0.019	Lagged Negative
	Intermediate (12-24 months)	0.100 – 0.544	1.354	0.012	Lagged Negative
	Long (36-72 months)	0.163 – 0.435	-2.464	0.033	Negative (anti-phase)
DOC – Precipitation (Figure S7)	Short (3-6 months)	0.185 – 0.280	1.906	<0.0001	Lagged Negative
	Intermediate (12-24 months)	0.295 – 0.638	0.860	<0.0001	Lagged Negative
	Long (36-72 months)	0.321 – 0.480	-0.867	0.038	Lagged Positive
DOC – Soil Wetness (Figure S8)	Short (3-6 months)	0.055 – 0.215	0.311	0.028	Positive (in-phase)
	Intermediate (12-24 months)	0.192 – 0.647	-1.305	<0.0001	Lagged Positive
	Long (36-72 months)	0.129 – 0.524	-1.146	0.112	Lagged Positive
DOC – Solar Irradiance (Figure S9)	Short (3-6 months)	0.075 – 0.268	-1.020	0.003	Lagged Positive
	Intermediate (12-24 months)	0.113 – 0.585	3.063	0.017	Negative (anti-phase)
	Long (36-72 months)	0.314 – 0.600	2.806	0.978	Negative (anti-phase)

[a] $-\pi/4 \leq$ phase $\leq \pi/4$ is positive (in-phase); $-3\pi/4 \leq$ phase $< -\pi/4$ is lagged positive; $\pi/4 <$ phase $\leq 3\pi/4$ is lagged negative; and $3\pi/4 <$ phase $\leq \pi$ or $-\pi \leq$ phase $< -3\pi/4$ is negative (anti-phase).[56]

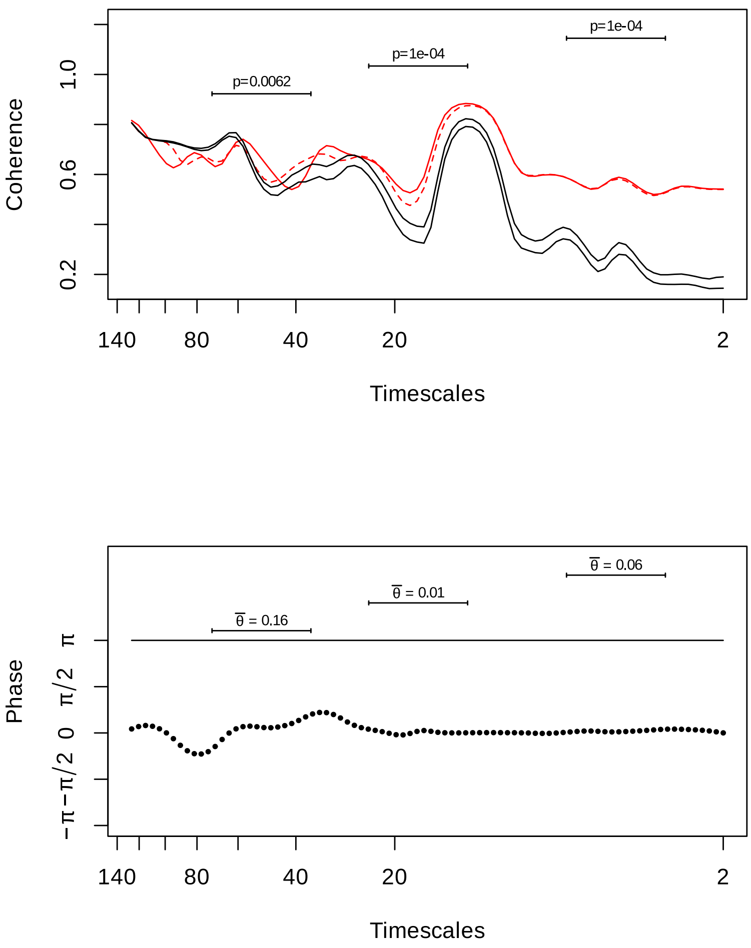

Figure S5. Coherence (upper panel) and phase (lower panel) plots for the time series of DOC and color in ALTM lakes. The solid red line represents the modulus of the observed coherence (i.e., the actual strength of association across timescales), while the dashed red line represents the approximate coherence used to evaluate statistical significance under the fast surrogate method. The black lines represent the 95th and 99th percentiles of surrogate coherences. Coherence is statistically significant when the dashed red line exceeds the black thresholds. Each *p*-value represents the aggregate significance of coherence across each timescale band of interest (i.e., short (3–6 months), intermediate (12–24 months), and long (36–72 months)), and the mean phase is computed over the same band. Timescales are in months.[57]

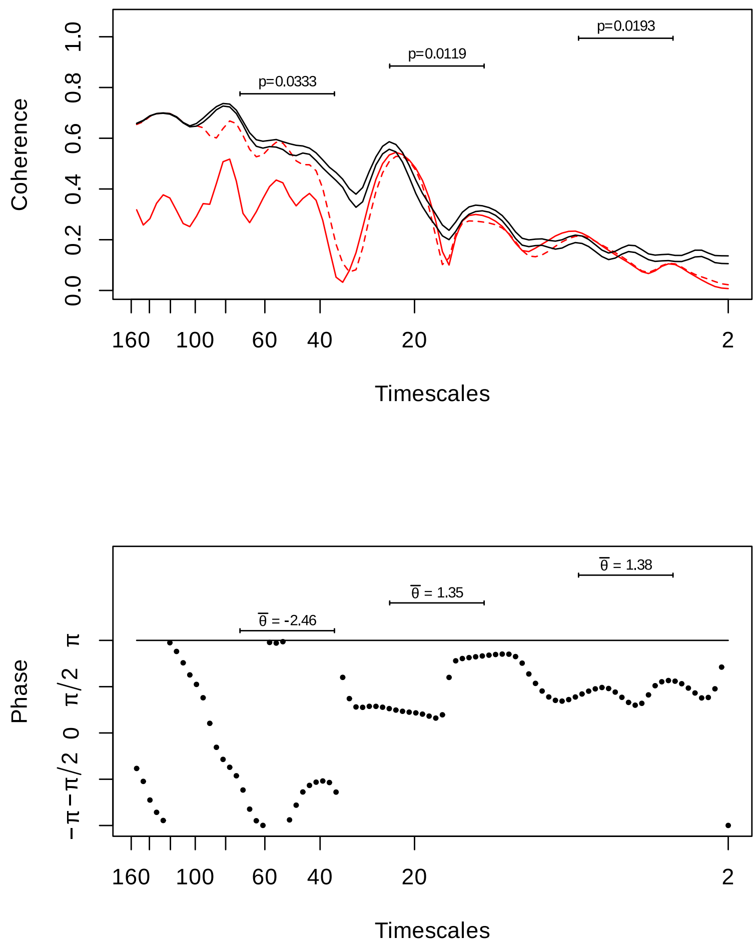

Figure S6. Coherence (upper panel) and phase (lower panel) plots for the time series of DOC in ALTM lakes and the summed sulfate and nitrate wet deposition in the Adirondack region. The solid red line represents the modulus of the observed coherence (i.e., the actual strength of association across timescales), while the dashed red line represents the approximate coherence used to evaluate statistical significance under the fast surrogate method. The black lines represent the 95th and 99th percentiles of surrogate coherences. Coherence is statistically significant when the dashed red line exceeds the black thresholds. Each p-value represents the aggregate significance of coherence across each timescale band of interest (i.e., short (3–6 months), intermediate (12–24 months), and long (36–72 months)), and the mean phase is computed over the same band. Timescales are in months.[57]

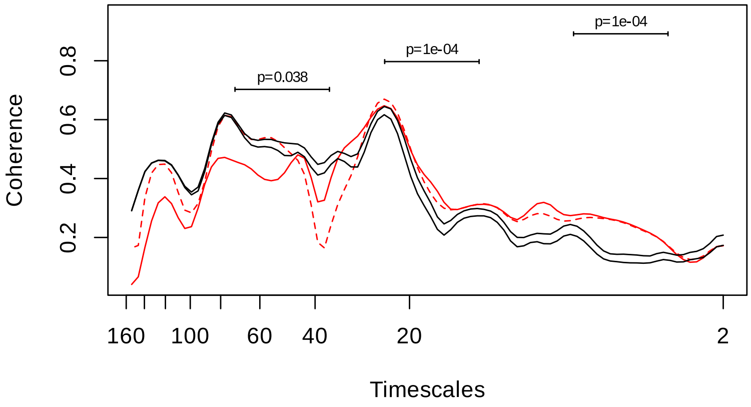

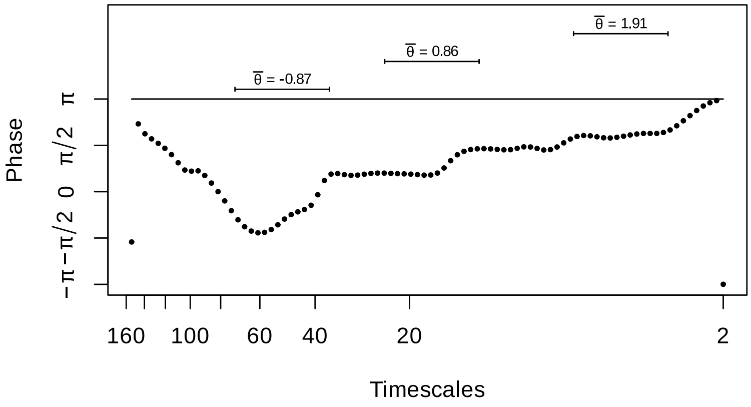

Figure S7. Coherence (upper panel) and phase (lower panel) plots for the time series of DOC in ALTM lakes and precipitation in the Adirondack region. The solid red line represents the modulus of the observed coherence (i.e., the actual strength of association across timescales), while the dashed red line represents the approximate coherence used to evaluate statistical significance under the fast surrogate method. The black lines represent the 95th and 99th percentiles of surrogate coherences. Coherence is statistically significant when the dashed red line exceeds the black thresholds. Each *p*-value represents the aggregate significance of coherence across each timescale band of interest (i.e., short (3–6 months), intermediate (12–24 months), and long (36–72 months)), and the mean phase is computed over the same band. Timescales are in months.[57]

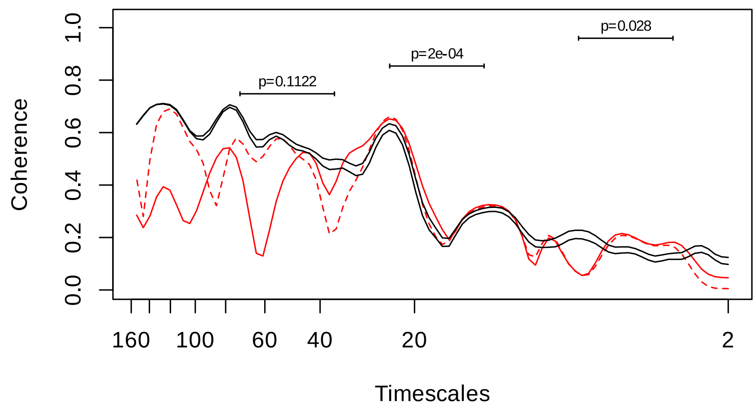

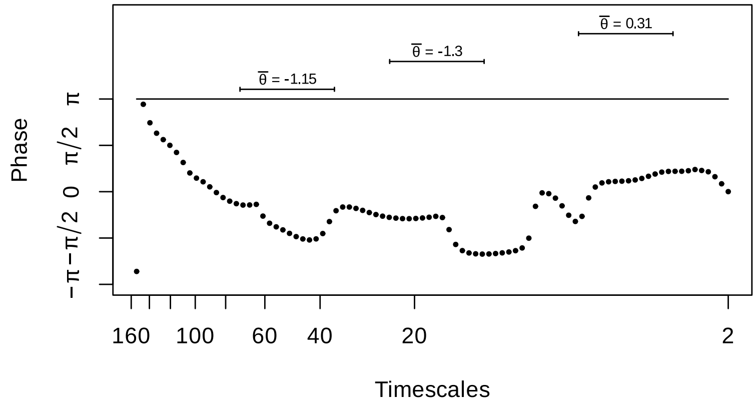

Figure S8. Coherence (upper panel) and phase (lower panel) plots for the time series of DOC in ALTM lakes and root zone soil wetness in the Adirondack region. The solid red line represents the modulus of the observed coherence (i.e., the actual strength of association across timescales), while the dashed red line represents the approximate coherence used to evaluate statistical significance under the fast surrogate method. The black lines represent the 95th and 99th percentiles of surrogate coherences. Coherence is statistically significant when the dashed red line exceeds the black thresholds. Each *p*-value represents the aggregate significance of coherence across each timescale band of interest (i.e., short (3–6 months), intermediate (12–24 months), and long (36–72 months)), and the mean phase is computed over the same band. Timescales are in months.[57]

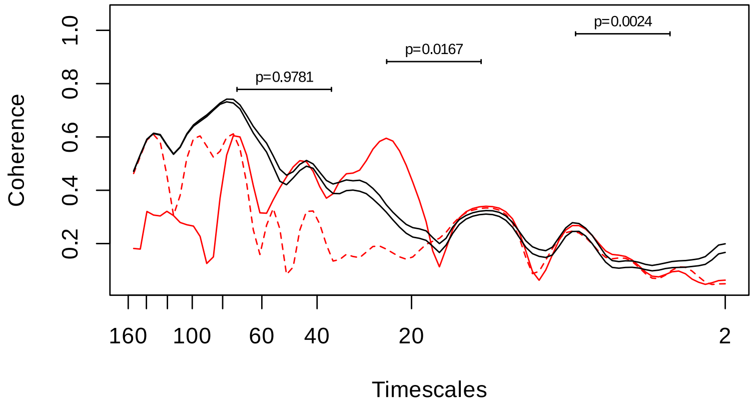

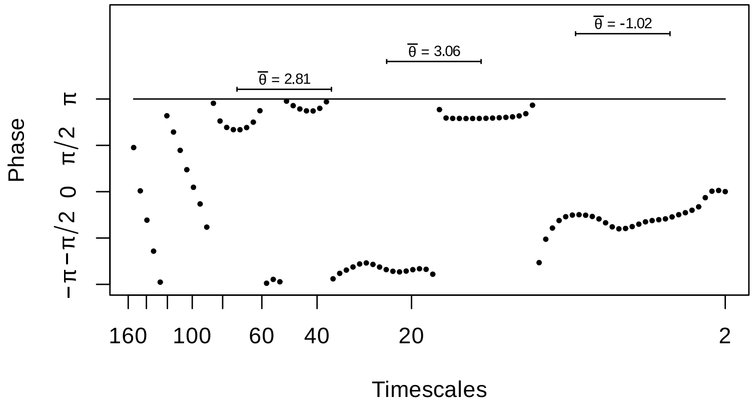

Figure S9. Coherence (upper panel) and phase (lower panel) plots for the time series of DOC in ALTM lakes and solar irradiance in the Adirondack region. The solid red line represents the modulus of the observed coherence (i.e., the actual strength of association across timescales), while the dashed red line represents the approximate coherence used to evaluate statistical significance under the fast surrogate method. The black lines represent the 95th and 99th percentiles of surrogate coherences. Coherence is statistically significant when the dashed red line exceeds the black thresholds. Each *p*-value represents the aggregate significance of coherence across each timescale band of interest (i.e., short (3–6 months), intermediate (12–24 months), and long (36–72 months)), and the mean phase is computed over the same band. Timescales are in months.[57]

**Table S7.** Summary statistics of wavelet linear modeling						
Timescale Band	DOC Synchrony Explained (%)	$SO_4 + NO_3$ Deposition (%)	Precipitation (%)	Soil Wetness (%)	Solar Irradiance (%)	Interactions (%)
Short	35.4	1.7	15.1	5.7	6.7	-6.2
Intermediate	81.1	17.0	17.7	29.8	39.6	-23.0
Long	59.6	19.4	46.2	Not significant	Not significant	-6.0

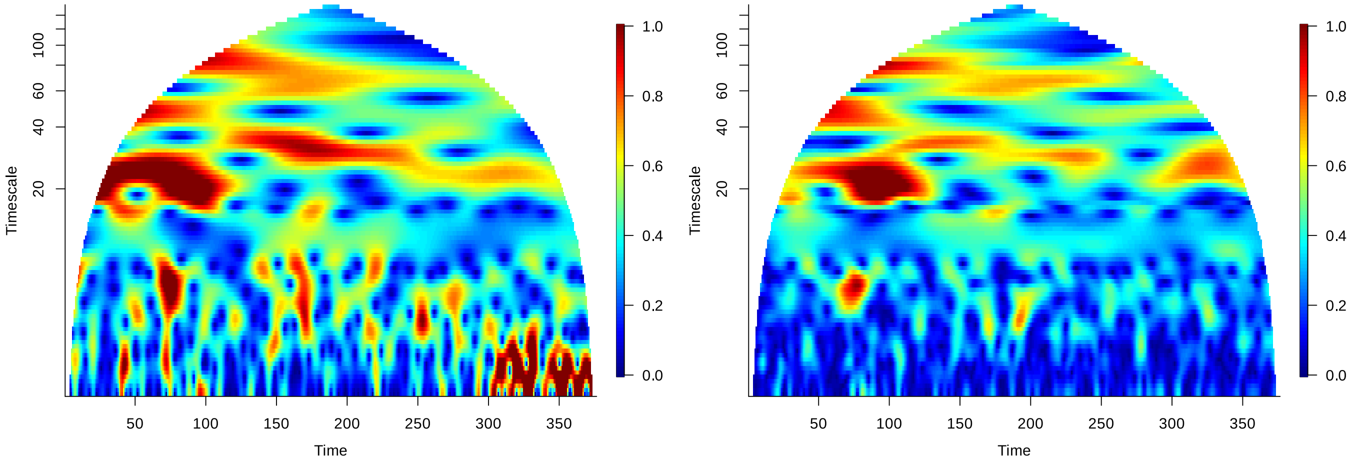

Figure S10. Wavelet mean field plots for observed (left panel; depicting the time and timescale dependence of synchrony) and predicted synchrony (right panel, based on wavelet linear modeling) in DOC across different timescale bands. Coherence magnitudes range from 0 and 1 with values closer to 1 representing stronger synchrony. Timescales are in months, and time is represented as a numeric sequence ranging from 1 (June 1992) to 376 (September 2023).[57]

10. Sen's slopes for the time series of DOC, color, and $SUVA_{254}$ in ALTM lakes

Table S8. Summary of Sen's slopes for water chemistry parameters

Site Name	Site ID	$[SO_4^{2-}]$ (μeq/L-year)	$[NO_3^-]$ (μeq/L-year)	$[Cl^-]$ (μeq/L-year)	$[F^-]$ (μeq/L-year)	$[SO_4^{2-}]+[NO_3^-]$ (μeq/L-year)	ANC (μeq/L-year)	DIC (mg C/L-year)
Avalanche Lake	050707-1	-3.135	-0.461	0.000	0.000	-3.600	0.800	0.009
Lake Colden	050706-1	-2.990	-0.255	0.000	0.000	-3.238	0.664	0.004
Brook Trout Lake	040874-1	-2.823	-0.242	0.000	0.000	-3.177	0.598	0.006
Squash Pond	040754-2	-2.379	0.000	0.000	0.000	-2.535	0.937	0.000
Constable Pond	040777-2	-3.331	-0.369	-0.006	-0.044	-3.828	0.658	0.011
East Copperas Pond	020138-1	-1.561	0.000	0.000	0.000	-1.636	0.489	0.014
Little Echo Pond	020126-1	-1.339	0.000	0.000	0.000	-1.398	0.760	0.000
Lake Rondaxe	040739-1	-2.080	-0.364	0.000	-0.023	-2.426	0.989	0.011
Cascade Lake	040747-1	-2.449	-0.346	0.000	-0.053	-2.957	0.923	0.009
Dart Lake	040750-1	-2.366	-0.452	0.000	-0.025	-2.820	0.965	0.010
Woods Lake	040576-1	-2.776	0.000	0.000	0.000	-2.941	0.467	0.006
Willys Lake	040210-1	-3.517	-0.766	0.000	-0.055	-4.288	0.571	0.000
Loon Hollow Pond	040186-1	-2.776	-0.363	0.000	0.000	-3.311	0.720	0.000
Little Clear Pond	030172-1	-1.388	0.000	0.000	0.000	-1.398	-1.832	-0.021
Black Pond	030256-1	-2.082	0.000	0.000	0.000	-2.123	1.003	0.000
Indian Lake	040852-1	-2.776	-0.228	0.000	0.000	-3.141	0.754	0.004
Squaw Lake	040850-1	-2.862	-0.230	-0.022	0.000	-3.226	0.958	0.007
Queer Lake	060329-1	-2.528	-0.450	-0.047	-0.029	-2.972	0.942	0.008
Big Hope Pond	020059-1	-2.379	0.000	-0.297	0.000	-2.427	1.520	0.016
G Lake	070859-1	-2.602	0.000	0.000	0.000	-3.022	0.957	0.005
West Pond	040753-2	-2.974	-0.074	0.000	-0.048	-3.213	0.431	0.017
Big Moose Lake	040752-1	-2.552	-0.484	0.000	-0.025	-3.089	0.969	0.009
Moss Lake	040746-1	-1.978	-0.346	0.000	-0.022	-2.340	1.193	0.012
Bubb Lake	040748-2	-1.883	0.000	0.000	0.000	-1.896	0.981	0.013
Limekiln Lake	040826-1	-2.280	-0.323	0.000	0.000	-2.600	1.319	0.017
Raquette Lake Reservoir	060315A-1	-2.935	-0.194	-0.067	-0.022	-3.339	0.808	0.021
Sagamore Lake	060313-1	-2.534	-0.269	-0.062	-0.021	-2.843	0.520	0.010
Arbutus Lake	050684-1	-2.366	-0.060	-0.040	-0.018	-2.483	0.385	0.008
Clear Pond	050458-1	-2.082	0.000	0.000	0.000	-2.186	0.400	0.000
Heart Lake	020264-1	-1.787	-0.034	0.000	0.000	-1.886	0.373	0.005
Grass Pond	040706-1	-2.271	0.000	0.000	-0.033	-2.376	0.600	0.006
Middle Branch Lake	040707-1	-1.952	0.000	0.000	0.000	-2.104	0.767	0.001
Middle Settlement Lake	040704-1	-2.258	0.000	0.000	0.000	-2.463	0.874	0.004
South Lake	041004-1	-2.186	-0.684	0.000	0.000	-2.858	1.116	0.013
North Lake	041007-1	-2.231	-0.504	0.000	0.000	-2.841	0.850	0.008
Otter Lake	070729-1	-2.672	0.000	0.000	0.000	-2.723	0.643	0.011
Jockeybush Lake	050259-1	-2.373	-0.209	0.000	0.000	-2.695	0.825	0.007

Table S8. Summary of Sen's slopes for water chemistry parameters (continued)									
Site Name	Site ID	DOC (mg C/L-year)	$[SiO_2]$ (mg C/L-year)	$[Ca^{2+}]$ (μeq/L-year)	$[Mg^{2+}]$ (μeq/L-year)	$[Na^{+}]$ (μeq/L-year)	$[K^{+}]$ (μeq/L-year)	$[NH_4^{+}]$ (μeq/L-year)	Base Cations (μeq/L-year)
Avalanche Lake	050707-1	0.071	-0.046	-1.272	-0.281	-0.065	-0.018	0.000	-1.684
Lake Colden	050706-1	0.067	-0.034	-1.103	-0.239	0.000	-0.026	0.000	-1.405
Brook Trout Lake	040874-1	0.065	-0.060	-0.998	-0.295	0.037	-0.060	0.000	-1.358
Squash Pond	040754-2	0.101	0.000	-0.512	-0.176	0.079	-0.096	0.000	-0.712
Constable Pond	040777-2	0.105	-0.055	-1.347	-0.406	0.000	-0.153	-0.069	-1.907
East Copperas Pond	020138-1	0.101	-0.022	-0.333	-0.099	0.029	-0.045	0.000	-0.442
Little Echo Pond	020126-1	0.000	0.000	-0.299	-0.206	0.213	0.000	0.000	-0.271
Lake Rondaxe	040739-1	0.048	-0.031	-0.550	-0.190	0.174	-0.073	0.000	-0.688
Cascade Lake	040747-1	0.057	0.000	-0.907	-0.315	0.062	-0.085	0.000	-1.230
Dart Lake	040750-1	0.064	-0.030	-0.668	-0.247	0.145	-0.096	0.000	-0.880
Woods Lake	040576-1	0.036	-0.051	-1.566	-0.239	0.000	-0.085	0.000	-1.903
Willys Lake	040210-1	0.065	-0.059	-1.295	-0.313	0.022	-0.109	0.000	-1.705
Loon Hollow Pond	040186-1	0.054	-0.036	-0.645	-0.224	0.109	-0.111	-0.092	-0.854
Little Clear Pond	030172-1	0.000	0.000	-2.246	-1.391	0.033	-0.037	0.000	-3.629
Black Pond	030256-1	0.000	-0.040	-0.451	-0.411	0.000	-0.015	0.000	-0.853
Indian Lake	040852-1	0.088	0.000	-1.019	-0.280	0.046	-0.035	0.000	-1.314
Squaw Lake	040850-1	0.032	0.000	-1.248	-0.366	0.083	-0.085	-0.111	-1.640
Queer Lake	060329-1	0.059	0.010	-0.907	-0.310	0.056	-0.134	-0.010	-1.316
Big Hope Pond	020059-1	0.047	0.038	-0.166	-0.165	0.000	-0.096	-0.139	-0.531
G Lake	070859-1	0.026	-0.035	-0.879	-0.231	0.104	-0.065	-0.049	-1.093
West Pond	040753-2	0.054	-0.042	-1.542	-0.510	-0.136	-0.059	0.000	-2.311
Big Moose Lake	040752-1	0.070	-0.030	-0.624	-0.241	0.145	-0.097	0.000	-0.847
Moss Lake	040746-1	0.038	-0.013	-0.499	-0.164	0.254	-0.060	0.000	-0.473
Bubb Lake	040748-2	0.000	0.000	-0.499	-0.223	0.183	-0.084	0.000	-0.693
Limekiln Lake	040826-1	0.045	0.000	-0.499	-0.222	0.144	-0.053	0.000	-0.560
Raquette Lake Reservoir	060315A-1	0.067	-0.072	-1.198	-0.454	0.000	-0.034	0.000	-1.712
Sagamore Lake	060313-1	0.060	-0.033	-0.950	-0.359	0.060	-0.056	0.000	-1.331
Arbutus Lake	050684-1	0.031	-0.022	-1.089	-0.433	0.000	-0.068	0.000	-1.612
Clear Pond	050458-1	0.027	0.000	-0.998	-0.224	0.041	-0.014	0.000	-1.239
Heart Lake	020264-1	0.005	0.000	-1.227	-0.188	0.130	-0.003	0.000	-1.299
Grass Pond	040706-1	0.026	-0.042	-0.749	-0.285	0.000	-0.057	-0.015	-1.155
Middle Branch Lake	040707-1	0.026	0.019	-0.566	-0.206	0.112	-0.036	0.000	-0.665
Middle Settlement Lake	040704-1	0.065	0.000	-0.455	-0.150	0.114	0.000	0.000	-0.492
South Lake	041004-1	0.057	-0.021	-0.653	-0.240	0.072	-0.066	0.000	-0.889
North Lake	041007-1	0.065	-0.023	-0.689	-0.255	0.064	-0.045	0.000	-0.934
Otter Lake	070729-1	0.056	0.000	-0.895	-0.332	0.041	0.000	0.000	-1.176
Jockeybush Lake	050259-1	0.046	0.000	-0.786	-0.258	0.092	-0.053	-0.055	-1.015
Base Cations = $\Sigma[Ca^{2+}]+[Mg^{2+}]+[Na^{+}]+[K^{+}]$									

Table S8. Summary of Sen's slopes for water chemistry parameters (continued)

Site Name	Site ID	Al_{TD} (µmol/L-year)	Al_{TM} (µmol/L-year)	Al_{OM} (µmol/L-year)	Al_{IM} (µmol/L-year)	pH ($year^{-1}$)	Color (m^{-1}/year)	Color (platinum-cobalt/year)	Specific Conductance (µS/cm-year)	$SUVA_{254}$ (L/mg C•m-year)
Avalanche Lake	050707-1	-0.259	-0.361	0.000	-0.325	0.0189	0.063	1.145	-0.500	-0.262
Lake Colden	050706-1	-0.191	-0.281	0.000	-0.259	0.0195	0.030	0.541	-0.412	-0.144
Brook Trout Lake	040874-1	-0.068	-0.065	0.019	-0.085	0.0273	0.015	0.278	-0.364	0.000
Squash Pond	040754-2	-0.105	-0.219	0.038	-0.232	0.0120	0.087	1.579	-0.500	-0.105
Constable Pond	040777-2	-0.209	-0.219	0.000	-0.229	0.0143	0.069	1.250	-0.500	-0.133
East Copperas Pond	020138-1	0.009	-0.017	0.000	-0.016	0.0057	0.145	2.647	-0.200	-0.100
Little Echo Pond	020126-1	0.000	-0.025	-0.019	-0.012	0.0075	0.220	4.000	-0.286	-0.177
Lake Rondaxe	040739-1	-0.037	-0.025	0.000	-0.026	0.0100	0.030	0.556	-0.187	-0.146
Cascade Lake	040747-1	0.000	0.000	0.000	-0.009	0.0058	0.017	0.312	-0.286	-0.100
Dart Lake	040750-1	-0.063	-0.069	0.000	-0.060	0.0212	0.041	0.750	-0.250	-0.100
Woods Lake	040576-1	0.000	-0.012	0.000	-0.016	0.0054	0.021	0.385	-0.308	0.000
Willys Lake	040210-1	-0.504	-0.469	0.019	-0.499	0.0219	0.026	0.476	-0.632	-0.100
Loon Hollow Pond	040186-1	-0.272	-0.430	0.028	-0.432	0.0144	0.027	0.500	-0.538	0.000
Little Clear Pond	030172-1	0.000	0.000	0.000	-0.037	-0.0084	0.016	0.294	-0.392	0.000
Black Pond	030256-1	0.000	0.000	0.006	-0.019	0.0000	0.017	0.312	-0.183	-0.218
Indian Lake	040852-1	-0.109	-0.166	0.016	-0.176	0.0200	0.075	1.364	-0.417	-0.100
Squaw Lake	040850-1	0.000	-0.009	0.009	-0.028	0.0211	0.016	0.294	-0.333	0.000
Queer Lake	060329-1	-0.046	-0.037	0.008	-0.048	0.0225	0.023	0.417	-0.286	-0.121
Big Hope Pond	020059-1	0.000	-0.020	-0.006	-0.012	0.0144	0.069	1.250	-0.190	-0.150
G Lake	070859-1	-0.062	-0.049	0.012	-0.074	0.0268	0.000	0.000	-0.316	0.000
West Pond	040753-2	-0.135	-0.098	-0.015	-0.065	0.0062	0.047	0.851	-0.440	-0.200
Big Moose Lake	040752-1	-0.096	-0.091	0.000	-0.084	0.0258	0.046	0.833	-0.286	-0.082
Moss Lake	040746-1	0.000	-0.009	0.000	-0.012	0.0053	0.015	0.278	-0.167	-0.100
Bubb Lake	040748-2	0.000	0.000	0.000	-0.015	0.0074	0.000	0.000	-0.167	0.000
Limekiln Lake	040826-1	-0.021	-0.011	0.000	-0.018	0.0156	0.012	0.227	-0.176	0.000
Raquette Lake Reservoir	060315A-1	-0.130	-0.111	-0.016	-0.087	0.0080	0.053	0.957	-0.357	-0.100
Sagamore Lake	060313-1	-0.033	-0.057	-0.014	-0.034	0.0046	0.061	1.111	-0.273	-0.125
Arbutus Lake	050684-1	0.000	0.000	0.000	-0.012	-0.0023	0.021	0.385	-0.267	-0.100
Clear Pond	050458-1	0.039	0.005	0.009	-0.009	0.0000	0.014	0.250	-0.231	-0.105
Heart Lake	020264-1	0.009	0.000	0.008	-0.015	0.0000	0.000	0.000	-0.211	-0.134
Grass Pond	040706-1	0.000	-0.037	0.000	-0.025	0.0078	0.016	0.294	-0.240	-0.050
Middle Branch Lake	040707-1	0.023	0.000	0.000	-0.021	0.0050	0.030	0.556	-0.186	0.000
Middle Settlement Lake	040704-1	-0.111	-0.072	0.000	-0.074	0.0280	0.023	0.417	-0.250	0.000
South Lake	041004-1	-0.091	-0.074	0.009	-0.082	0.0316	0.026	0.476	-0.286	-0.100
North Lake	041007-1	-0.140	-0.185	0.000	-0.152	0.0237	0.055	1.000	-0.333	-0.120
Otter Lake	070729-1	-0.081	-0.062	0.016	-0.074	0.0194	0.023	0.417	-0.300	0.000
Jockeybush Lake	050259-1	-0.065	-0.074	0.008	-0.080	0.0281	0.000	0.000	-0.300	0.000

Al_{TD} = total dissolved Al, Al_{TM} = total monomeric Al, Al_{OM} = organic monomeric Al, Al_{IM} = inorganic monomeric Al.

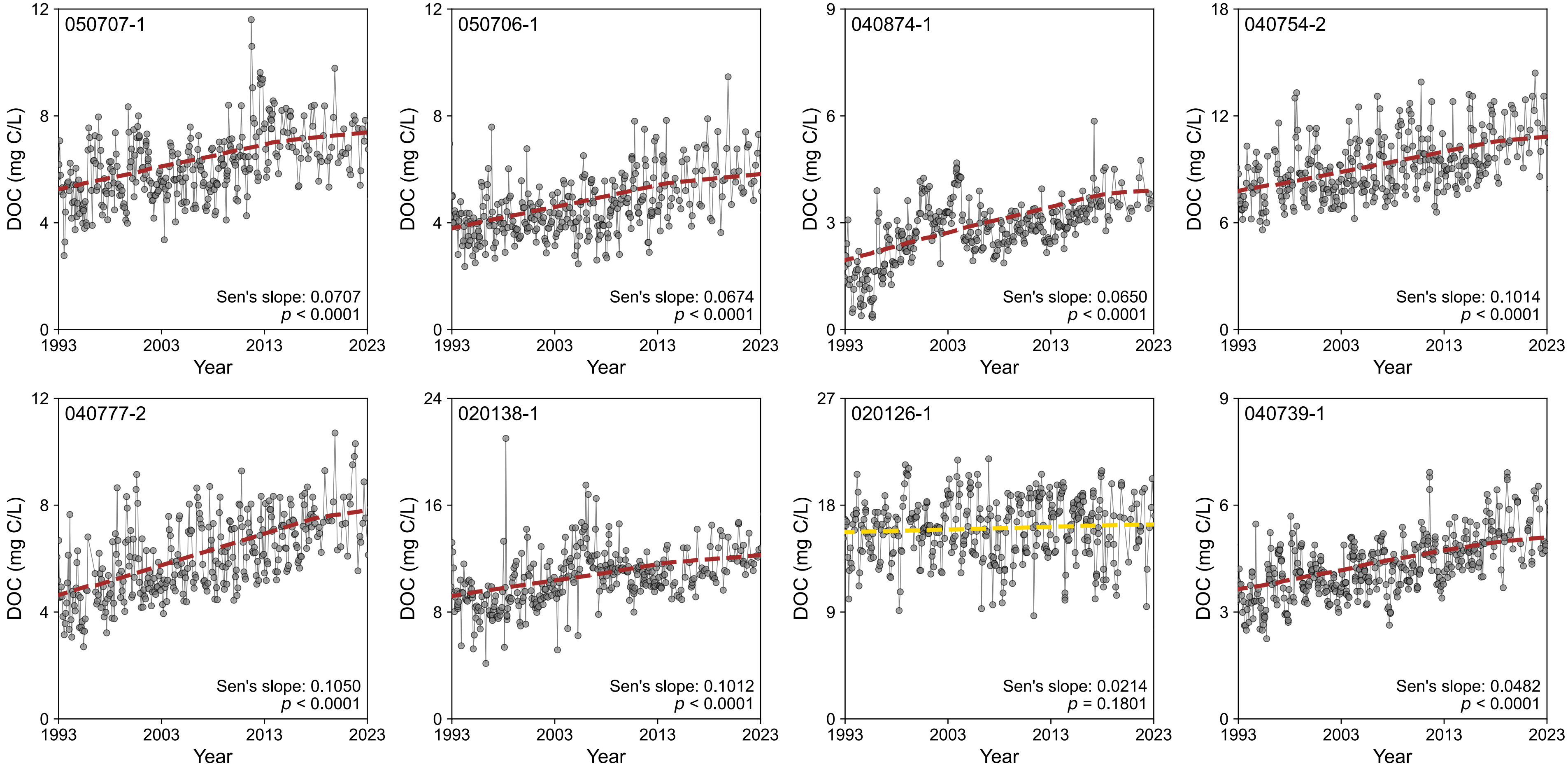
050707-1
Sen's slope: 0.0707
p < 0.0001
050706-1
Sen's slope: 0.0674
p < 0.0001
040874-1
Sen's slope: 0.0650
p < 0.0001
040754-2
Sen's slope: 0.1014
p < 0.0001
040777-2
Sen's slope: 0.1050
p < 0.0001
020138-1
Sen's slope: 0.1012
p < 0.0001
020126-1
Sen's slope: 0.0214
p = 0.1801
040739-1
Sen's slope: 0.0482
p < 0.0001
DOC (mg C/L)
Year
1993
2003
2013
2023

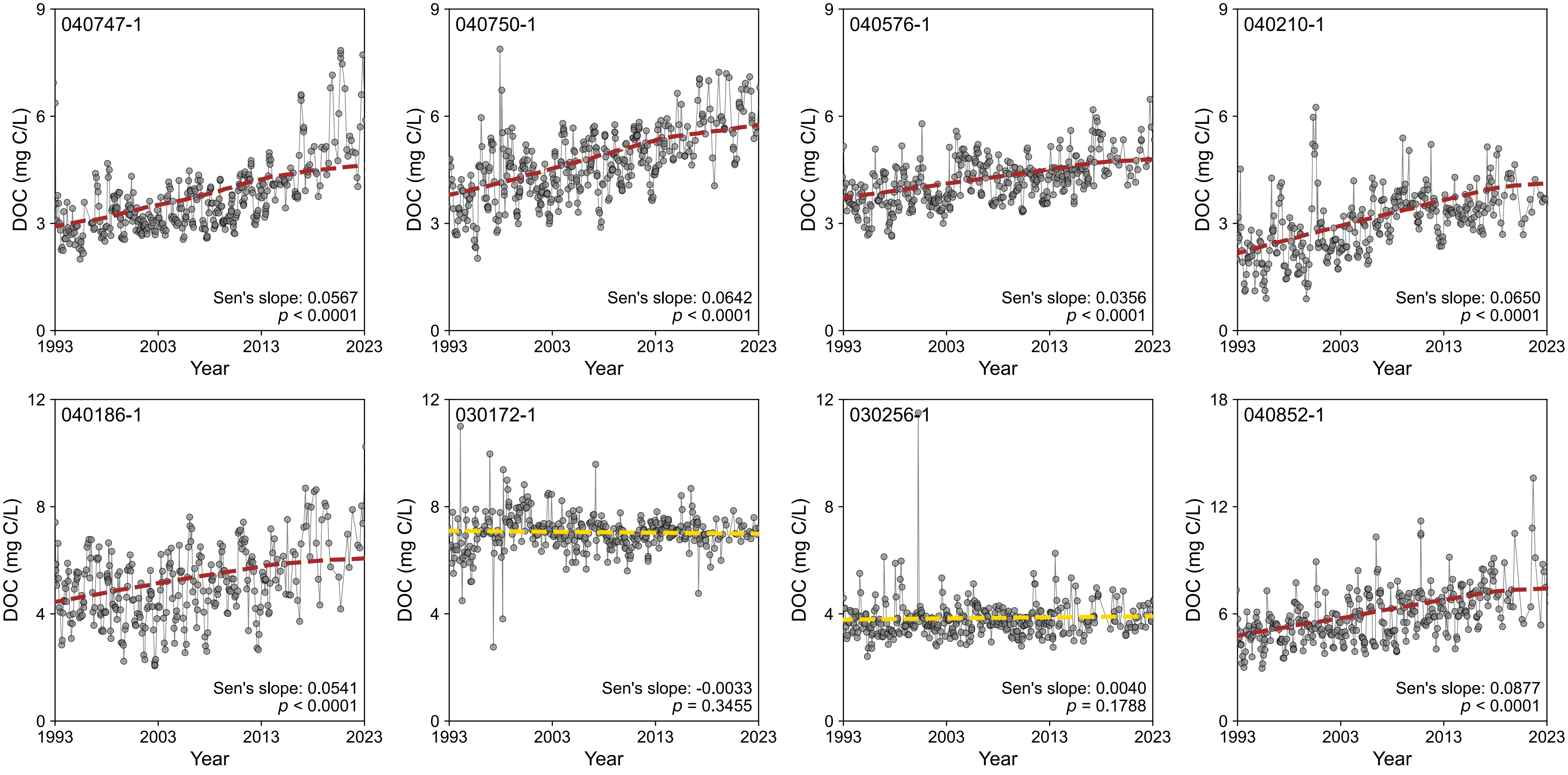

040747-1
Sen's slope: 0.0567
p < 0.0001
040750-1
Sen's slope: 0.0642
p < 0.0001
040576-1
Sen's slope: 0.0356
p < 0.0001
040210-1
Sen's slope: 0.0650
p < 0.0001
040186-1
Sen's slope: 0.0541
p < 0.0001
030172-1
Sen's slope: -0.0033
p = 0.3455
030256-1
Sen's slope: 0.0040
p = 0.1788
040852-1
Sen's slope: 0.0877
p < 0.0001
DOC (mg C/L)
Year
1993
2003
2013
2023

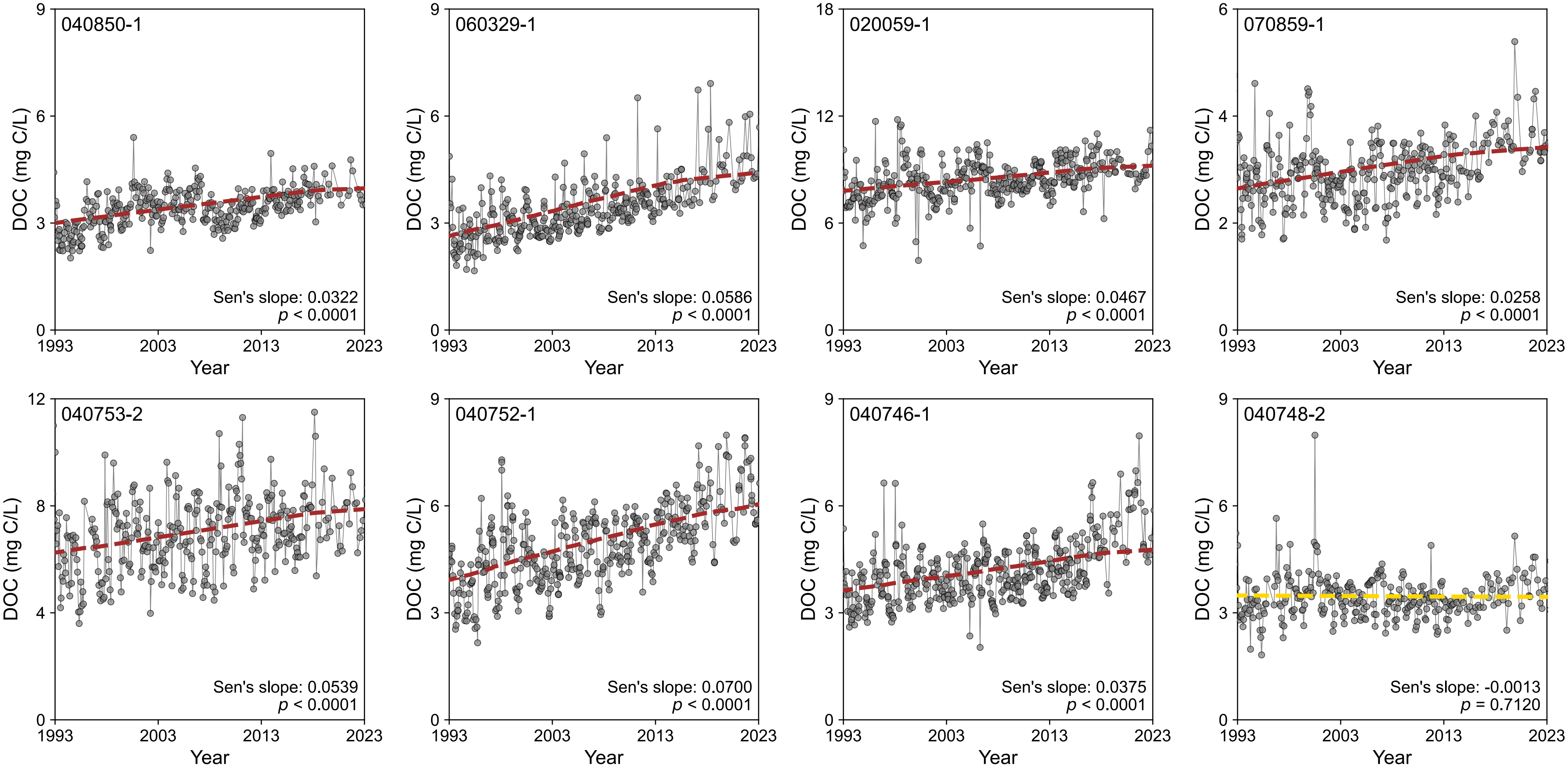
040850-1
Sen's slope: 0.0322
p < 0.0001
060329-1
Sen's slope: 0.0586
p < 0.0001
020059-1
Sen's slope: 0.0467
p < 0.0001
070859-1
Sen's slope: 0.0258
p < 0.0001
040753-2
Sen's slope: 0.0539
p < 0.0001
040752-1
Sen's slope: 0.0700
p < 0.0001
040746-1
Sen's slope: 0.0375
p < 0.0001
040748-2
Sen's slope: -0.0013
p = 0.7120
DOC (mg C/L)
Year

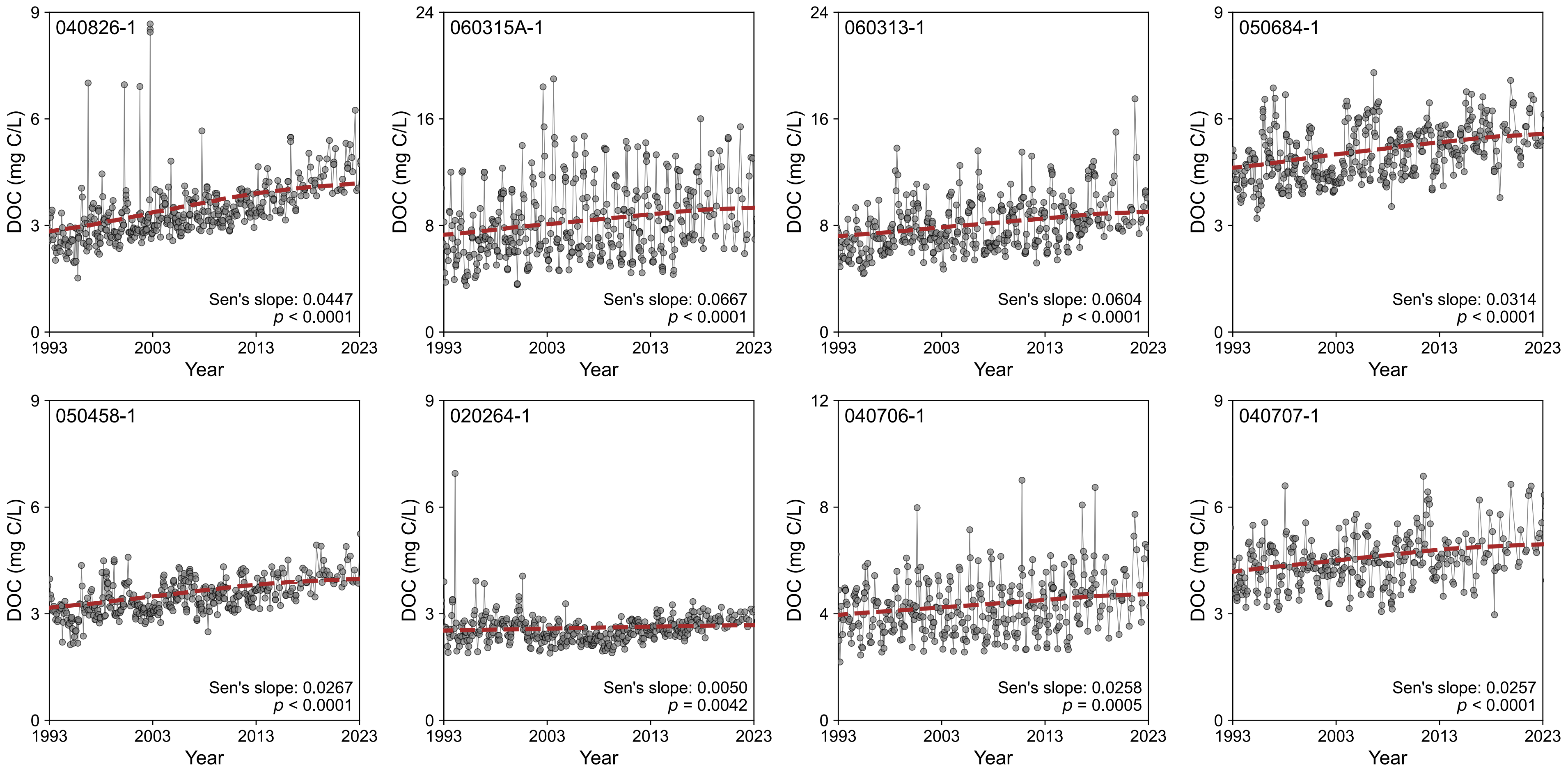
040826-1
Sen's slope: 0.0447
p < 0.0001
060315A-1
Sen's slope: 0.0667
p < 0.0001
060313-1
Sen's slope: 0.0604
p < 0.0001
050684-1
Sen's slope: 0.0314
p < 0.0001
050458-1
Sen's slope: 0.0267
p < 0.0001
020264-1
Sen's slope: 0.0050
p = 0.0042
040706-1
Sen's slope: 0.0258
p = 0.0005
040707-1
Sen's slope: 0.0257
p < 0.0001
DOC (mg C/L)
Year
1993
2003
2013
2023

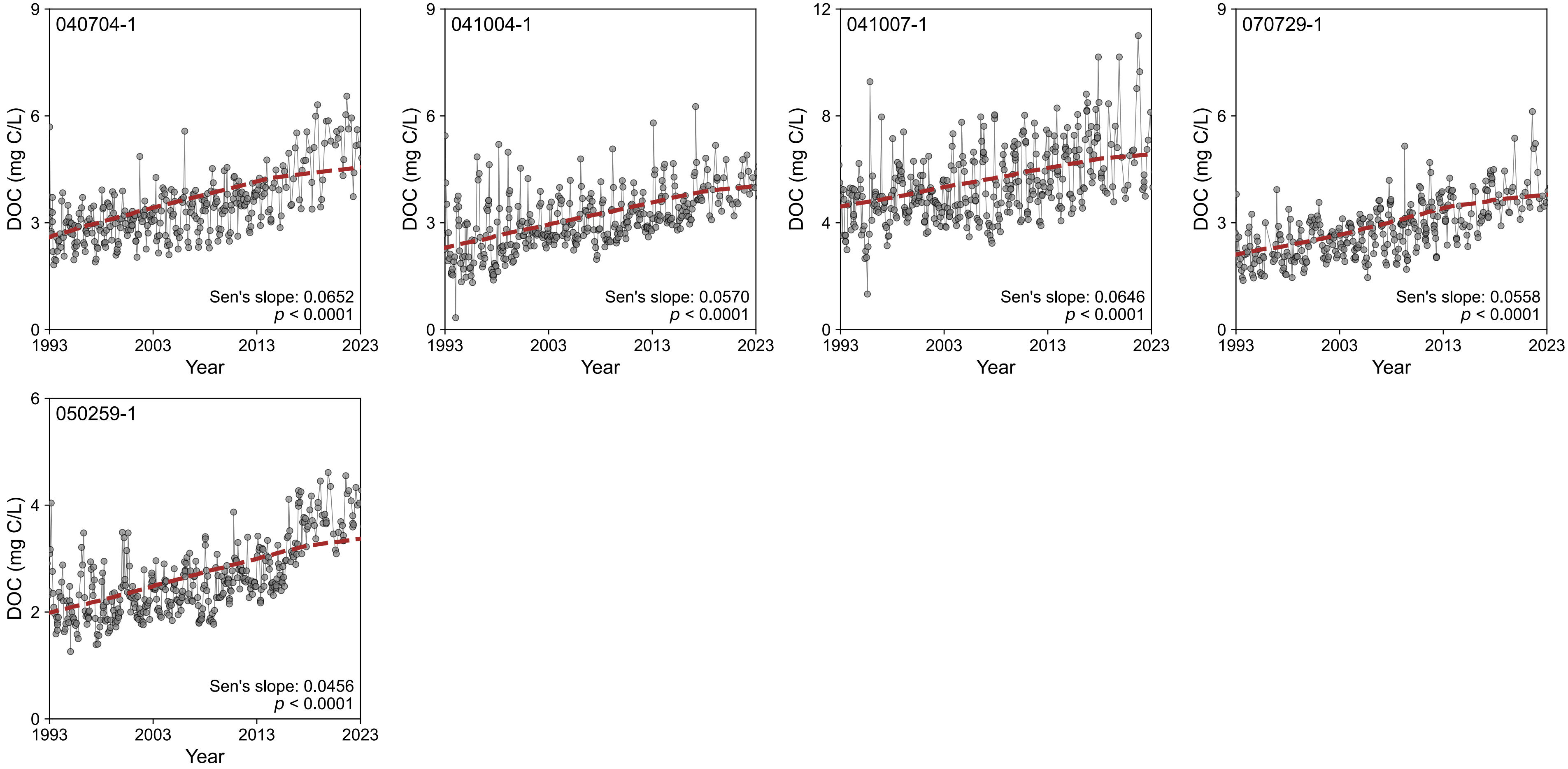

Figure S11. Seasonal Mann-Kendall test of the time series of DOC in ALTM lakes. In each subplot, the dashed line represents the trend curve calculated using Sen's slope. The red dashed line denotes a statistically significant positive slope, the blue dashed line denotes a statistically significant negative slope, and the yellow dashed line denotes a non-significant slope.

11. $\Phi_{app, {}^1O_2}$ for ALTM samples and comparisons with literature data

**Table S9.** $\Phi_{app, {}^1O_2}$ for whole water samples from ALTM lakes						
Site Name	Sampling Date	$\Phi_{app, {}^1O_2}$ ($\times 10^{-2}$ mol mol-photons^{-1})	Sampling Date	$\Phi_{app, {}^1O_2}$ ($\times 10^{-2}$ mol mol-photons^{-1})	Sampling Date	$\Phi_{app, {}^1O_2}$ ($\times 10^{-2}$ mol mol-photons^{-1})
Avalanche Lake	10/17/2022	1.70±0.12	6/2/2023	2.77±0.13	9/25/2023	2.35±0.12
Lake Colden	10/17/2022	2.49±0.16	6/2/2023	3.66±0.14	9/25/2023	3.09±0.13
Brook Trout Lake	10/26/2022	1.63±0.06	5/22/2023	1.90±0.03	9/5/2023	1.75±0.06
Squash Pond Outlet	10/31/2022	2.79±0.04	5/15/2023	3.24±0.09	9/18/2023	3.00±0.07
Squash Pond	10/31/2022	2.80±0.07	5/15/2023	3.17±0.08	9/18/2023	3.03±0.08
Constable Pond Outlet	10/31/2022	2.53±0.08	5/15/2023	3.01±0.12	9/18/2023	2.78±0.06
Constable Pond	10/31/2022	2.53±0.11	5/15/2023	3.05±0.13	9/18/2023	2.85±0.07
East Copperas Pond	11/1/2022	1.56±0.08	6/27/2023	1.94±0.13	9/15/2023	1.89±0.06
Little Echo Pond	11/1/2022	0.58±0.02	6/29/2023	0.66±0.04	9/21/2023	0.64±0.03
Lake Rondaxe	11/2/2022	2.36±0.08	6/19/2023	2.99±0.10	9/23/2023	2.63±0.12
Cascade Lake	11/2/2022	2.12±0.04	6/16/2023	2.55±0.05	9/18/2023	2.20±0.10
Cascade Lake Outlet	11/2/2022	2.14±0.04	6/16/2023	2.54±0.09	9/18/2023	2.26±0.14
Dart Lake	11/2/2022	1.87±0.01	5/23/2023	2.46±0.06	9/22/2023	2.03±0.03
Woods Lake	11/2/2022	2.56±0.06	6/16/2023	3.56±0.11	9/18/2023	3.11±0.18
Willys Lake	11/3/2022	3.40±0.01	5/23/2023	3.61±0.04	9/19/2023	3.49±0.04
Loon Hollow Pond	11/3/2022	2.08±0.06	5/23/2023	2.43±0.04	9/19/2023	2.21±0.04
Little Clear Pond	11/7/2022	0.68±0.05	6/22/2023	0.92±0.05	9/12/2023	0.82±0.04
Black Pond	11/7/2022	1.90±0.04	6/29/2023	2.23±0.09	9/21/2023	2.08±0.06
Indian Lake	11/8/2022	2.79±0.07	5/22/2023	3.31±0.14	9/5/2023	3.06±0.05
Squaw Lake	11/8/2022	3.24±0.07	5/22/2023	3.46±0.09	9/5/2023	3.34±0.04
Queer Lake	11/9/2022	2.51±0.17	6/6/2023	3.44±0.05	9/13/2023	2.92±0.08
Big Hope Pond	11/10/2022	1.76±0.06	6/23/2023	2.53±0.22	9/21/2023	2.04±0.06
G Lake	11/10/2022	2.04±0.01	6/29/2023	2.28±0.03	9/12/2023	2.14±0.06
West Pond Outlet	11/14/2022	1.67±0.06	6/16/2023	1.98±0.06	9/4/2023	1.84±0.04
West Pond	11/14/2022	1.68±0.11	6/16/2023	1.99±0.05	9/4/2023	1.85±0.02
Big Moose Lake	11/14/2022	2.95±0.07	6/16/2023	3.29±0.07	9/4/2023	3.13±0.10
Moss Lake	11/14/2022	2.10±0.12	5/25/2023	2.94±0.15	9/26/2023	2.54±0.05
Bubb Lake	11/14/2022	1.67±0.07	5/25/2023	2.18±0.07	9/4/2023	1.96±0.09
Bubb Lake Outlet	11/14/2022	1.70±0.08	5/25/2023	2.21±0.13	9/4/2023	1.94±0.11
Limekiln Lake	11/15/2022	2.58±0.05	6/6/2023	3.05±0.13	9/4/2023	2.69±0.07
Raquette Lake Reservoir	11/15/2022	1.70±0.04	5/23/2023	1.88±0.02	9/26/2023	1.77±0.03
Sagamore Lake	11/15/2022	1.21±0.06	6/20/2023	1.51±0.04	9/29/2023	1.37±0.04
Arbutus Pond	11/15/2022	1.54±0.03	6/23/2023	1.84±0.05	9/26/2023	1.63±0.06
Clear Pond	11/15/2022	1.78±0.04	6/23/2023	2.24±0.01	9/26/2023	1.88±0.04
Heart Lake	11/15/2022	2.12±0.04	6/13/2023	2.36±0.03	9/5/2023	2.26±0.04
Grass Pond	11/17/2022	1.66±0.06	5/16/2023	2.20±0.12	9/4/2023	1.87±0.06
Middle Branch Lake	11/17/2022	1.55±0.03	5/16/2023	1.97±0.09	9/4/2023	1.76±0.14
Middle Settlement Lake	11/17/2022	2.44±0.09	5/16/2023	3.38±0.12	9/4/2023	3.20±0.08
South Lake	11/28/2022	2.12±0.04	6/26/2023	2.69±0.12	9/6/2023	2.44±0.06
North Lake	11/28/2022	1.87±0.10	6/26/2023	2.44±0.15	9/6/2023	2.15±0.11
Otter Lake Outlet	11/29/2022	2.10±0.17	6/5/2023	2.73±0.09	9/14/2023	2.42±0.04
Otter Lake	11/29/2022	2.13±0.15	6/5/2023	2.81±0.10	9/14/2023	2.46±0.11
Jockeybush Lake	11/29/2022	2.26±0.07	6/5/2023	3.24±0.08	9/12/2023	2.69±0.03

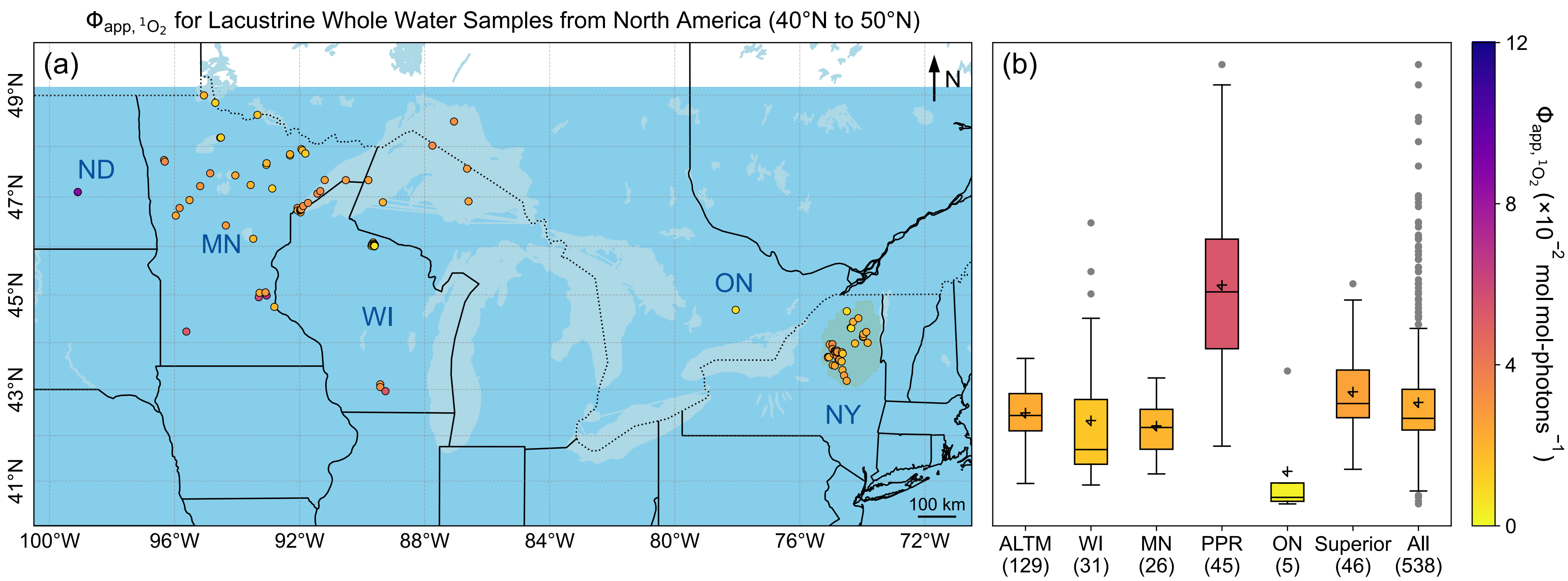

Figure S12. Comparisons of $\Phi_{app,\,{}^1O_2}$ for whole water samples from Adirondack Long-Term Monitoring (ALTM) lakes and literature values for lacustrine whole water samples: **(a)** Map of $\Phi_{app,\,{}^1O_2}$ for ALTM samples and values reported for lacustrine whole water samples from the temperate mid-latitudes (40°N to 50°N) of North America. Marker colors reflect the average value for samples from each site with reference to the color bar. The Adirondack Park is shaded in green. "WI" denotes Wisconsin, "MN" denotes Minnesota, "PPR" refers to the Prairie Pothole Region, "ON" denotes Ontario, "Superior" represents Lake Superior, and "All" aggregates $\Phi_{app,\,{}^1O_2}$ for lacustrine whole water samples from 21 references with relevant data. **(b)** Comparisons of $\Phi_{app,\,{}^1O_2}$ for ALTM samples with those compiled for lacustrine whole water samples from previous studies conducted in North America. In the boxplot, each box spans the 25th to 75th percentiles, with whiskers extending to 1.5 times the interquartile range below the 25th and above the 75th percentiles. The centerline and "+" sign mark the median and mean, respectively. Gray circles represent outliers. Numbers in parentheses represent the number of samples in each group. Box colors correspond to their respective median values referenced against the color bar.

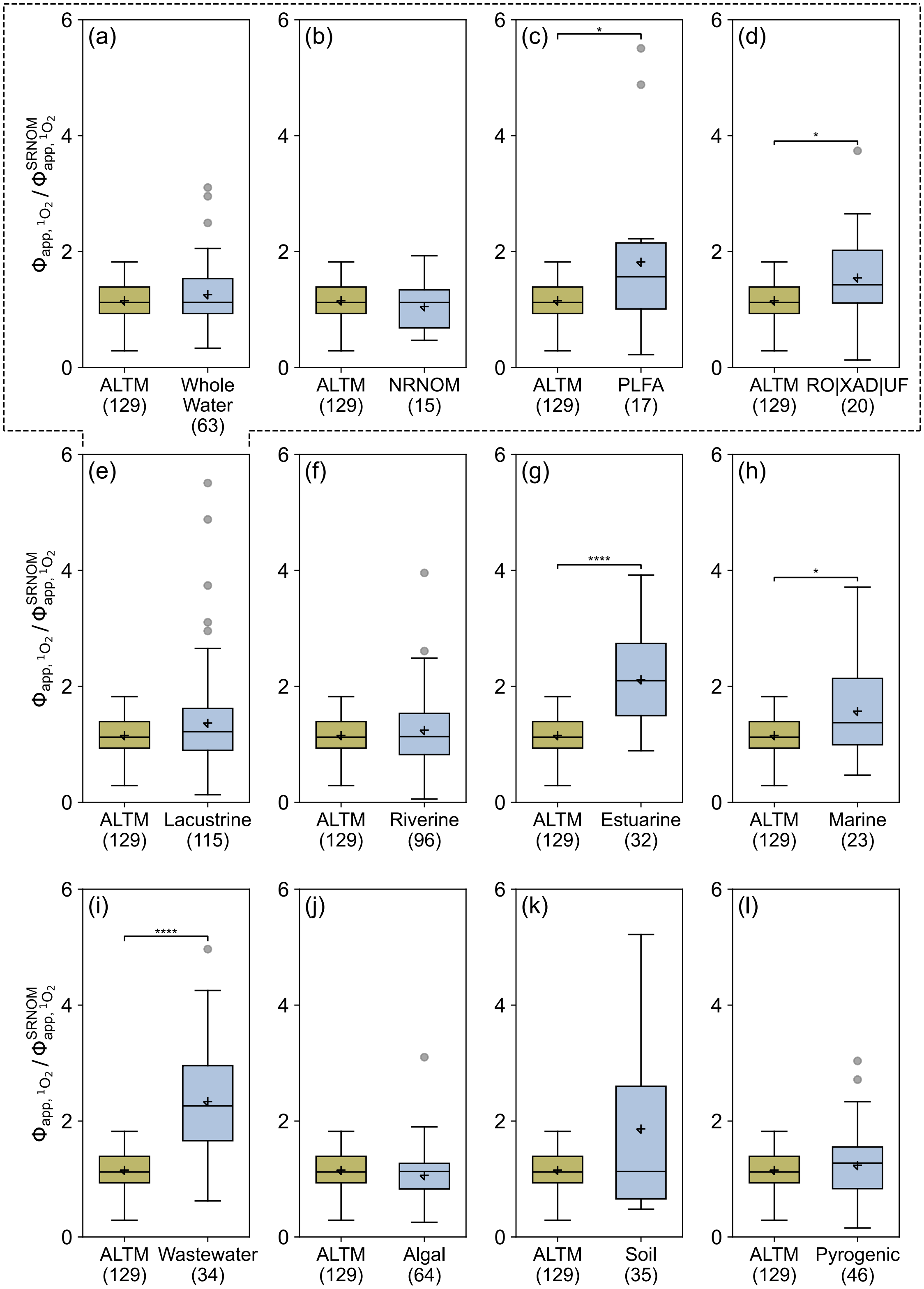

Figure S13. Comparisons of $\Phi_{app,\,^1O_2}/\Phi^{SRNOM}_{app,\,^1O_2}$ for ALTM samples with literature values (Table S18). "NRNOM" refers to Nordic Reservoir Natural Organic Matter, "PLFA" refers to Pony Lake Fulvic Acid, "RO" refers to reverse osmosis, and "UF" refers to ultrafiltration. Each box spans the 25th to 75th percentiles, with whiskers extending to 1.5 times the interquartile range below the 25th and above the 75th percentiles. The centerline and "+" sign mark the median and mean, respectively. Gray circles represent outliers. Numbers in parentheses represent the number of samples in each group. Significant differences are marked by asterisks as "*" (p<0.05), "**" (p<0.01), "***" (p<0.001), or "****" (p<0.0001).

12. Spatiotemporal patterns of optical properties of ALTM samples

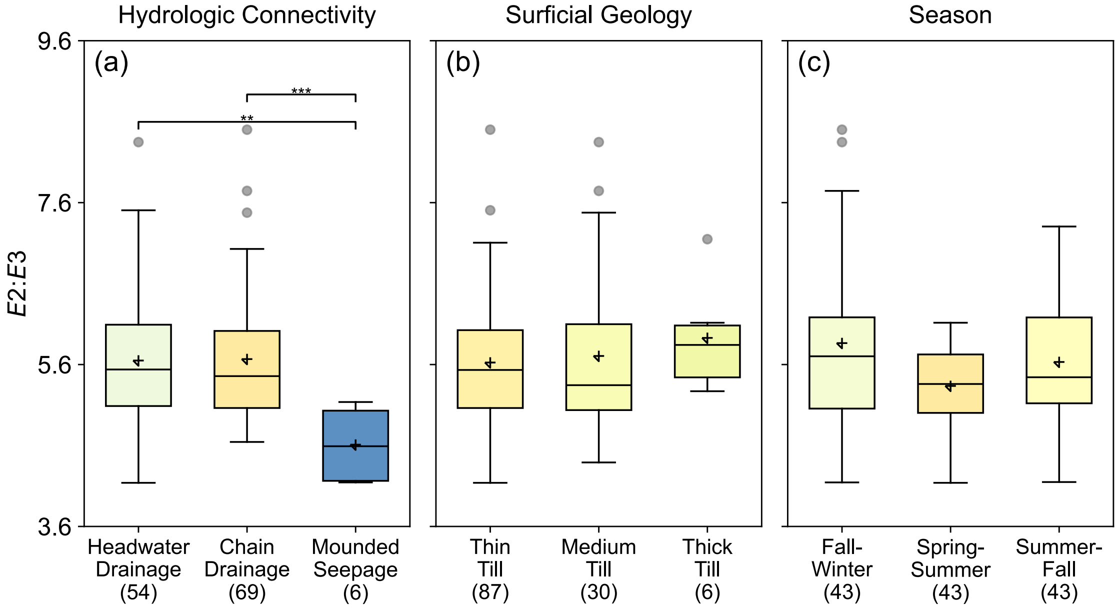

Figure S15. Multiple comparisons of *E2:E3* across ALTM lakes by **(a)** hydrologic connectivity, **(b)** surficial geology, and **(c)** season. Each box spans the 25th to 75th percentiles, with whiskers extending to 1.5 times the interquartile range below the 25th and above the 75th percentiles. The centerline and "+" sign mark the median and mean, respectively. Gray circles represent outliers. Numbers in parentheses represent the number of samples in each group. Box colors correspond to those in Figure 3. For multiple comparisons, a Kruskal-Wallis test was first performed to determine whether statistically significant differences existed among groups. If significant, pairwise Mann-Whitney *U* tests were performed, with significant differences marked by asterisks as "*" (p<0.05), "**" (p<0.01), "***" (p<0.001), or "****" (p<0.0001).

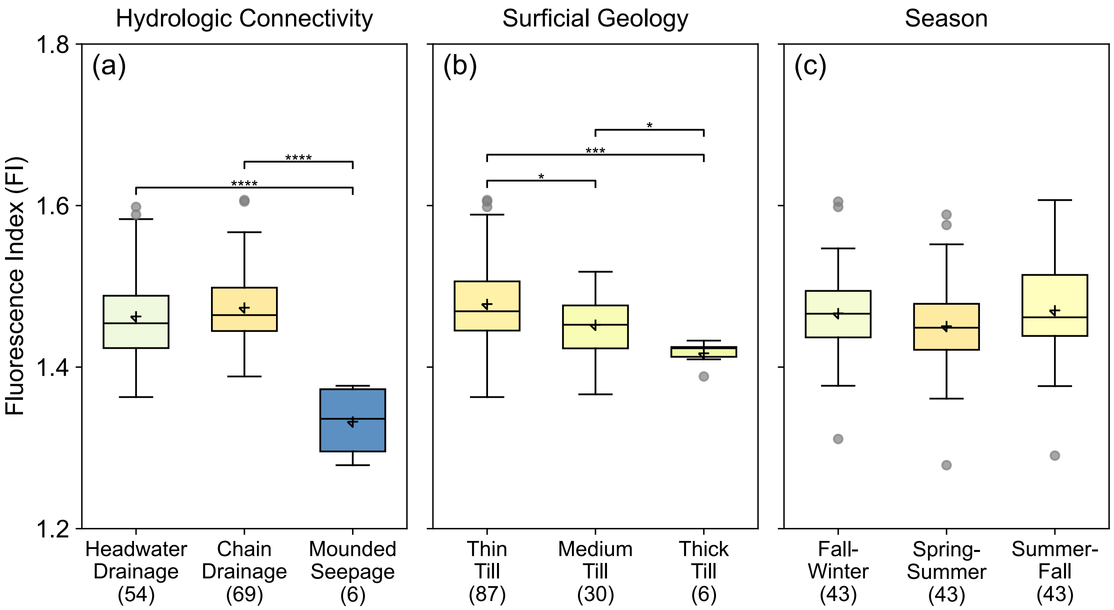

Figure S16. Multiple comparisons of fluorescence index (FI) across ALTM lakes by **(a)** hydrologic connectivity, **(b)** surficial geology, and **(c)** season. Each box spans the 25th to 75th percentiles, with whiskers extending to 1.5 times the interquartile range below the 25th and above the 75th percentiles. The centerline and "+" sign mark the median and mean, respectively. Gray circles represent outliers. Numbers in parentheses represent the number of samples in each group. Box colors correspond to those in Figure 3. For multiple comparisons, a Kruskal-Wallis test was first performed to determine whether statistically significant differences existed among groups. If significant, pairwise Mann-Whitney U tests were performed, with significant differences marked by asterisks as "*" ($p<0.05$), "**" ($p<0.01$), "***" ($p<0.001$), or "****" ($p<0.0001$).

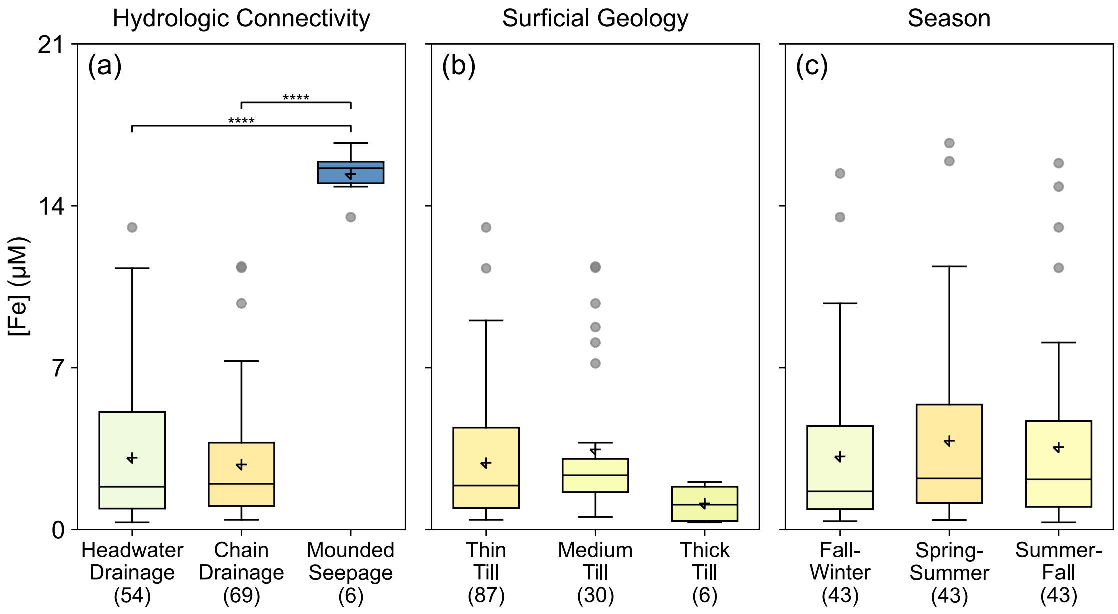

Figure S17. Multiple comparisons of total dissolved iron ([Fe]) across ALTM lakes by **(a)** hydrologic connectivity, **(b)** surficial geology, and **(c)** season. Each box spans the 25th to 75th percentiles, with whiskers extending to 1.5 times the interquartile range below the 25th and above the 75th percentiles. The centerline and "+" sign mark the median and mean, respectively. Gray circles represent outliers. Numbers in parentheses represent the number of samples in each group. Box colors correspond to those in Figure 3. For multiple comparisons, a Kruskal-Wallis test was first performed to determine whether statistically significant differences existed among groups. If significant, pairwise Mann-Whitney *U* tests were performed, with significant differences marked by asterisks as "*" (p<0.05), "**" (p<0.01), "***" (p<0.001), or "****" (p<0.0001).

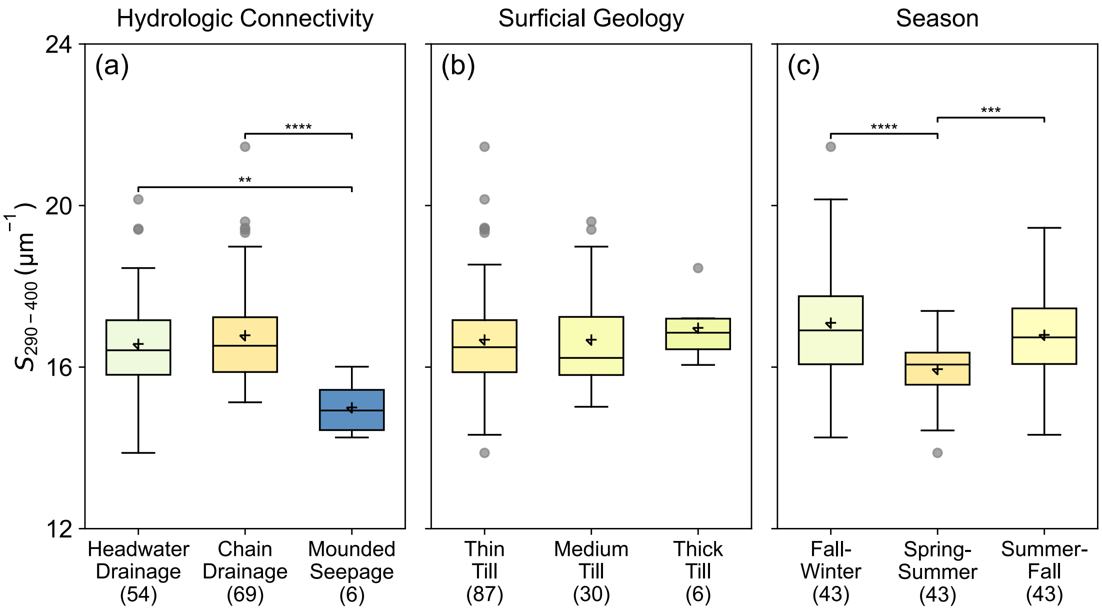

Figure S18. Multiple comparisons of $S_{290\text{-}400}$ across ALTM lakes by **(a)** hydrologic connectivity, **(b)** surficial geology, and **(c)** season. Each box spans the 25th to 75th percentiles, with whiskers extending to 1.5 times the interquartile range below the 25th and above the 75th percentiles. The centerline and "+" sign mark the median and mean, respectively. Gray circles represent outliers. Numbers in parentheses represent the number of samples in each group. Box colors correspond to those in Figure 3. For multiple comparisons, a Kruskal-Wallis test was first performed to determine whether statistically significant differences existed among groups. If significant, pairwise Mann-Whitney *U* tests were performed, with significant differences marked by asterisks as "*" ($p<0.05$), "**" ($p<0.01$), "***" ($p<0.001$), or "****" ($p<0.0001$).

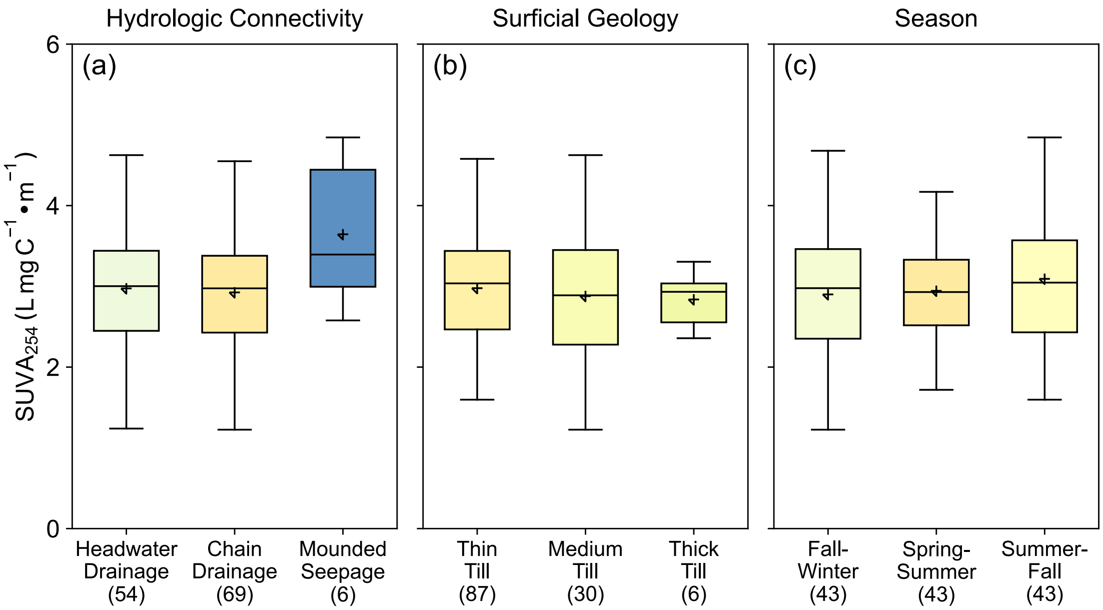

Figure S19. Multiple comparisons of $SUVA_{254}$ across ALTM lakes by **(a)** hydrologic connectivity, **(b)** surficial geology, and **(c)** season. Each box spans the 25th to 75th percentiles, with whiskers extending to 1.5 times the interquartile range below the 25th and above the 75th percentiles. The centerline and "+" sign mark the median and mean, respectively. Gray circles represent outliers. Numbers in parentheses represent the number of samples in each group. Box colors correspond to those in Figure 3. For multiple comparisons, a Kruskal-Wallis test was first performed to determine whether statistically significant differences existed among groups. If significant, pairwise Mann-Whitney *U* tests were performed, with significant differences marked by asterisks as "*" (p<0.05), "**" (p<0.01), "***" (p<0.001), or "****" (p<0.0001).

13. Optical properties as predictors of $\Phi_{app, ^1O_2}$ for ATLM samples

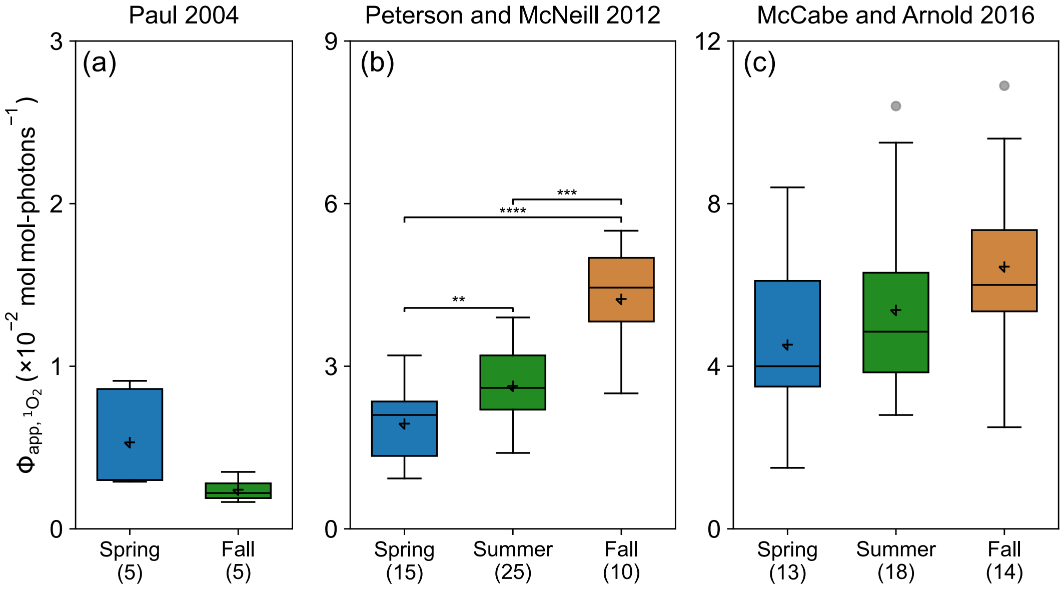

Figure S20. Multiple comparisons of $\Phi_{app, ^1O_2}$ for seasonal samples from **(a)** Scandinavian lakes and streams (Spring 2000 and Fall 1999),[58] **(b)** Lake Superior and its tributaries (Spring: May 2007 and May 2008; Summer: June 2007, August 2008, and June 2009; Fall: September 2007),[19] and **(c)** Prairie Pothole wetlands (Spring: May 2013 and May 2014; Summer: July 2012, July 2013, June 2014, and August 2014; Fall: November 2012, November 2013, and November 2014).[34] Each box spans the 25th to 75th percentiles, with whiskers extending to 1.5 times the interquartile range below the 25th and above the 75th percentiles. The centerline and "+" sign mark the median and mean, respectively. Gray circles represent outliers. Numbers in parentheses represent the number of samples in each group. For multiple comparisons, a Kruskal-Wallis test was first performed to determine whether statistically significant differences existed among groups. If significant, pairwise Mann-Whitney *U* tests were performed, with significant differences marked by asterisks as "*" (p<0.05), "**" (p<0.01), "***" (p<0.001), or "****" (p<0.0001).

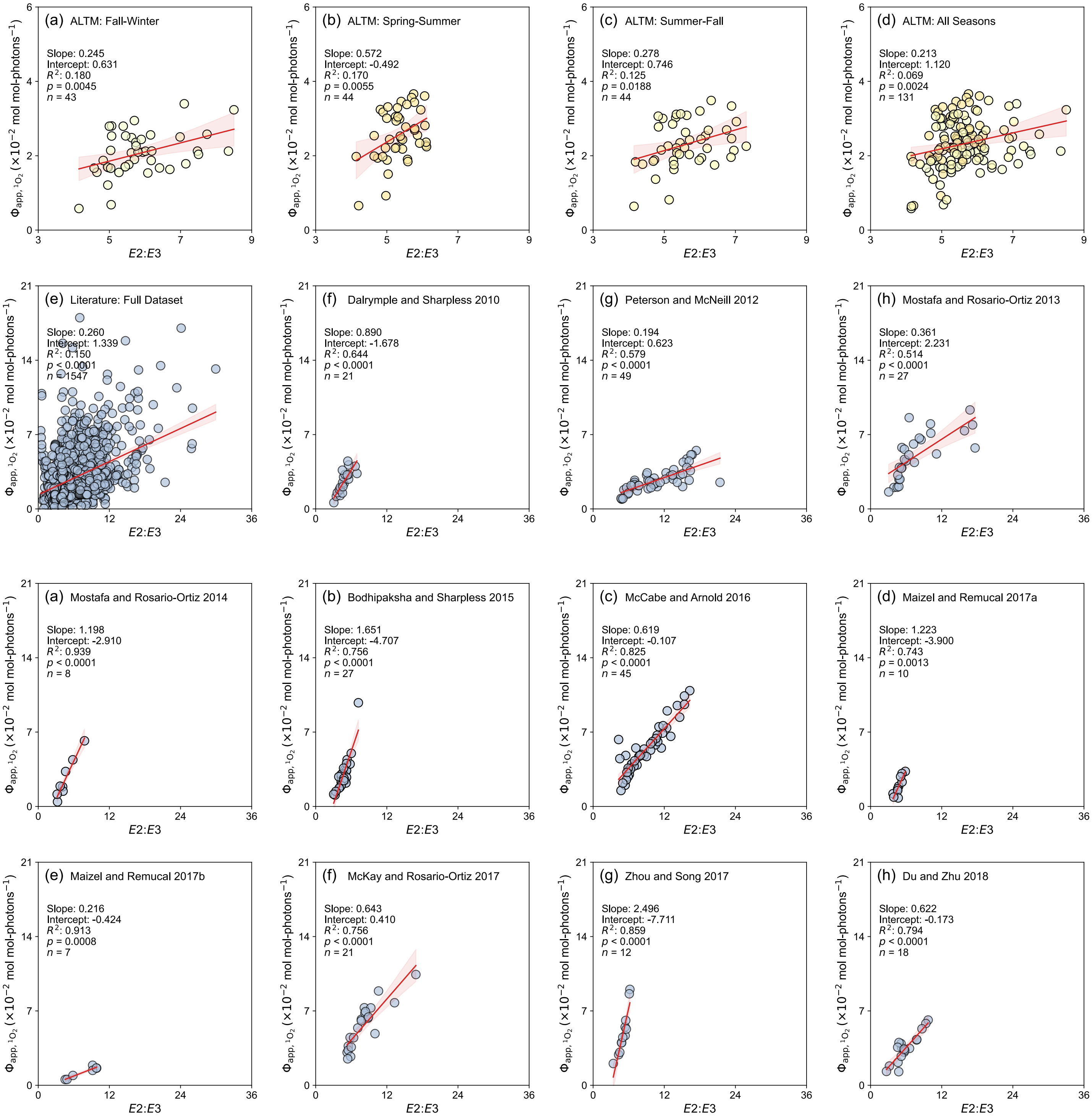
(a) ALTM: Fall-Winter
Slope: 0.245
Intercept: 0.631
R^2: 0.180
p = 0.0045
n = 43
(b) ALTM: Spring-Summer
Slope: 0.572
Intercept: -0.492
R^2: 0.170
p = 0.0055
n = 44
(c) ALTM: Summer-Fall
Slope: 0.278
Intercept: 0.746
R^2: 0.125
p = 0.0188
n = 44
(d) ALTM: All Seasons
Slope: 0.213
Intercept: 1.120
R^2: 0.069
p = 0.0024
n = 131
(e) Literature: Full Dataset
Slope: 0.260
Intercept: 1.339
R^2: 0.150
p < 0.0001
n = 1547
(f) Dalrymple and Sharpless 2010
Slope: 0.890
Intercept: -1.678
R^2: 0.644
p < 0.0001
n = 21
(g) Peterson and McNeill 2012
Slope: 0.194
Intercept: 0.623
R^2: 0.579
p < 0.0001
n = 49
(h) Mostafa and Rosario-Ortiz 2013
Slope: 0.361
Intercept: 2.231
R^2: 0.514
p < 0.0001
n = 27
(a) Mostafa and Rosario-Ortiz 2014
Slope: 1.198
Intercept: -2.910
R^2: 0.939
p < 0.0001
n = 8
(b) Bodhipaksha and Sharpless 2015
Slope: 1.651
Intercept: -4.707
R^2: 0.756
p < 0.0001
n = 27
(c) McCabe and Arnold 2016
Slope: 0.619
Intercept: -0.107
R^2: 0.825
p < 0.0001
n = 45
(d) Maizel and Remucal 2017a
Slope: 1.223
Intercept: -3.900
R^2: 0.743
p = 0.0013
n = 10
(e) Maizel and Remucal 2017b
Slope: 0.216
Intercept: -0.424
R^2: 0.913
p = 0.0008
n = 7
(f) McKay and Rosario-Ortiz 2017
Slope: 0.643
Intercept: 0.410
R^2: 0.756
p < 0.0001
n = 21
(g) Zhou and Song 2017
Slope: 2.496
Intercept: -7.711
R^2: 0.859
p < 0.0001
n = 12
(h) Du and Zhu 2018
Slope: 0.622
Intercept: -0.173
R^2: 0.794
p < 0.0001
n = 18
$\Phi_{app,\,^1O_2}$ ($\times 10^{-2}$ mol mol-photons^{-1})
$E2{:}E3$

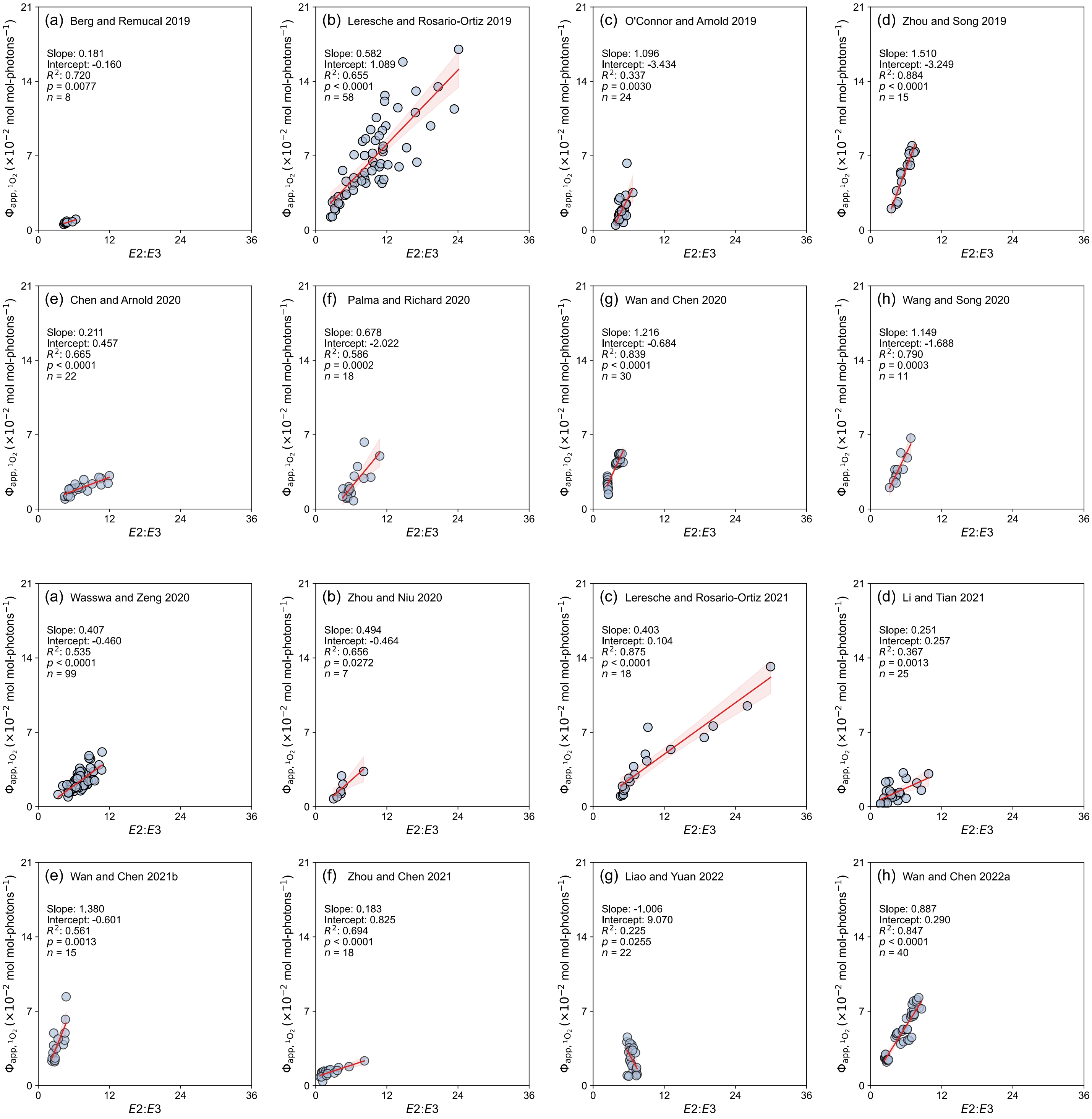

(a) Berg and Remucal 2019
Slope: 0.181
Intercept: -0.160
R^2: 0.720
p = 0.0077
n = 8
(b) Leresche and Rosario-Ortiz 2019
Slope: 0.582
Intercept: 1.089
R^2: 0.655
p < 0.0001
n = 58
(c) O'Connor and Arnold 2019
Slope: 1.096
Intercept: -3.434
R^2: 0.337
p = 0.0030
n = 24
(d) Zhou and Song 2019
Slope: 1.510
Intercept: -3.249
R^2: 0.884
p < 0.0001
n = 15
(e) Chen and Arnold 2020
Slope: 0.211
Intercept: 0.457
R^2: 0.665
p < 0.0001
n = 22
(f) Palma and Richard 2020
Slope: 0.678
Intercept: -2.022
R^2: 0.586
p = 0.0002
n = 18
(g) Wan and Chen 2020
Slope: 1.216
Intercept: -0.684
R^2: 0.839
p < 0.0001
n = 30
(h) Wang and Song 2020
Slope: 1.149
Intercept: -1.688
R^2: 0.790
p = 0.0003
n = 11
(a) Wasswa and Zeng 2020
Slope: 0.407
Intercept: -0.460
R^2: 0.535
p < 0.0001
n = 99
(b) Zhou and Niu 2020
Slope: 0.494
Intercept: -0.464
R^2: 0.656
p = 0.0272
n = 7
(c) Leresche and Rosario-Ortiz 2021
Slope: 0.403
Intercept: 0.104
R^2: 0.875
p < 0.0001
n = 18
(d) Li and Tian 2021
Slope: 0.251
Intercept: 0.257
R^2: 0.367
p = 0.0013
n = 25
(e) Wan and Chen 2021b
Slope: 1.380
Intercept: -0.601
R^2: 0.561
p = 0.0013
n = 15
(f) Zhou and Chen 2021
Slope: 0.183
Intercept: 0.825
R^2: 0.694
p < 0.0001
n = 18
(g) Liao and Yuan 2022
Slope: -1.006
Intercept: 9.070
R^2: 0.225
p = 0.0255
n = 22
(h) Wan and Chen 2022a
Slope: 0.887
Intercept: 0.290
R^2: 0.847
p < 0.0001
n = 40
$\Phi_{app,\,^1O_2}$ ($\times 10^{-2}$ mol mol-photons^{-1})
$E2{:}E3$
0
7
14
21
0
12
24
36

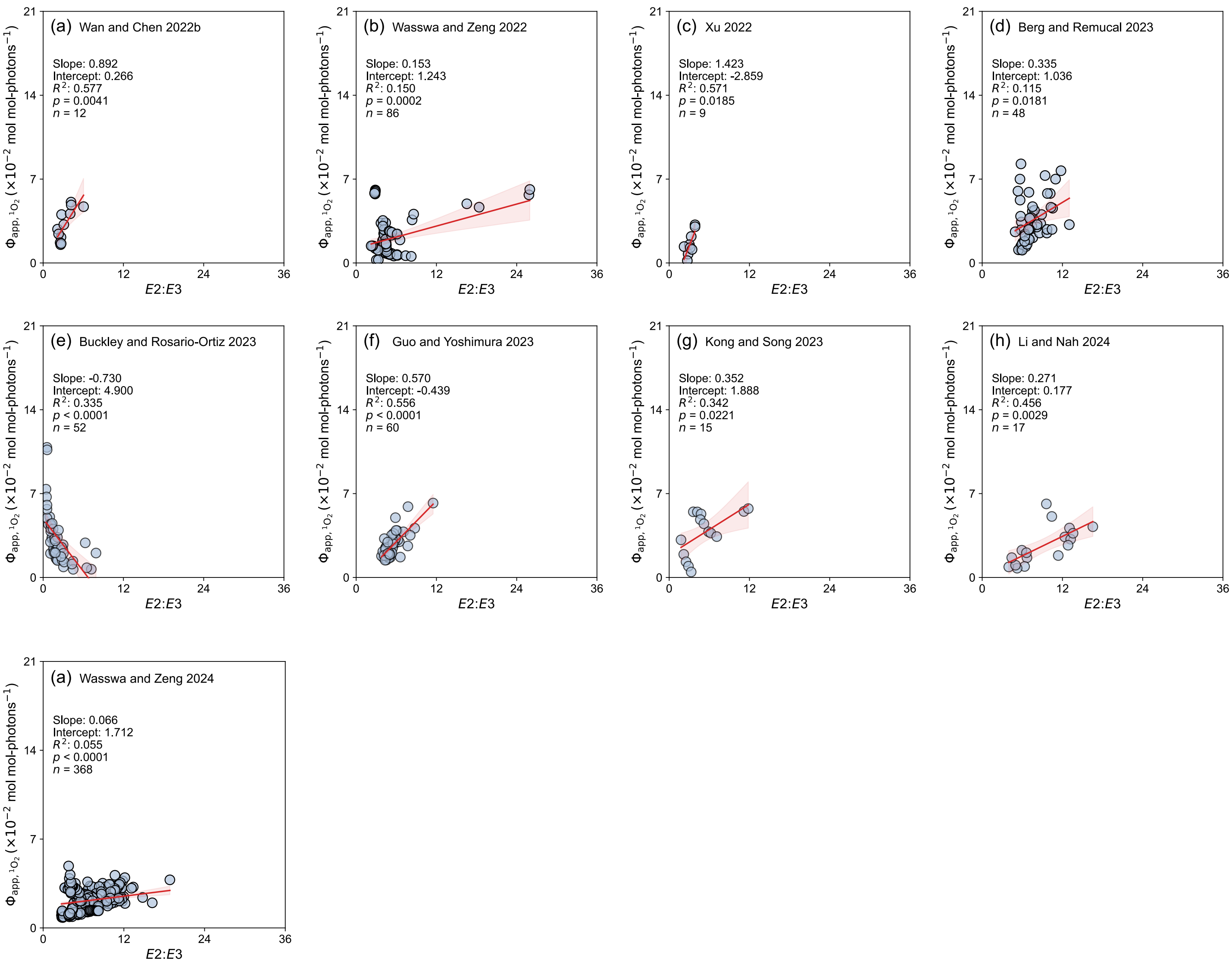

Figure S21. Linear regression between $\Phi_{app,\ ^1O_2}$ and *E2:E3* for ALTM samples and for samples from previous work (Table S10). Only studies with at least seven data points were included in the regression analysis. The red solid line denotes the regression line, and the shaded area represents the 95% confidence interval. Marker colors for ALTM samples correspond to those in Figure 3.

**Table S10.** Summary statistics of linear regression between $\Phi_{app, {}^1O_2}$ and *E2:E3* from the literature									
Reference (See details in Table S18)	Slope	Intercept	R^2	*p*-value	*n*	Minimum $\Phi_{app, {}^1O_2}$ ($\times 10^{-2}$ mol mol-photons^{-1})	Maximum $\Phi_{app, {}^1O_2}$ ($\times 10^{-2}$ mol mol-photons^{-1})	Min *E2:E3*	Max *E2:E3*
Full Dataset	0.26	1.34	0.150	<0.0001	1547	0.19	17.02	0.42	29.94
Dalrymple and Sharpless 2010	0.89	-1.68	0.644	<0.0001	21	0.59	4.50	3.06	6.96
Peterson and McNeill 2012	0.19	0.62	0.579	<0.0001	49	0.93	5.50	4.70	21.40
Mostafa and Rosario-Ortiz 2013	0.36	2.23	0.514	<0.0001	27	1.60	9.32	3.06	17.63
Mostafa and Rosario-Ortiz 2014	1.20	-2.91	0.939	<0.0001	8	0.44	6.18	3.19	7.80
Bodhipaksha and Sharpless 2015	1.65	-4.71	0.756	<0.0001	27	1.07	9.76	3.04	7.20
McCabe and Arnold 2016	0.62	-0.11	0.825	<0.0001	45	1.50	10.90	4.30	16.30
Maizel and Remucal 2017a	1.22	-3.90	0.743	0.0013	10	0.80	3.30	3.79	5.86
Maizel and Remucal 2017b	0.22	-0.42	0.913	0.0008	7	0.54	1.89	4.54	9.85
McKay and Rosario-Ortiz 2017	0.64	0.41	0.756	<0.0001	21	2.49	10.43	5.30	16.90
Zhou and Song 2017	2.50	-7.71	0.859	<0.0001	12	2.05	9.03	3.40	6.20
Du and Zhu 2018	0.62	-0.17	0.794	<0.0001	18	1.28	6.14	2.68	9.71
Berg and Remucal 2019	0.18	-0.16	0.720	0.0077	8	0.54	1.03	4.32	6.33
Leresche and Rosario-Ortiz 2019	0.58	1.09	0.655	<0.0001	58	1.24	17.02	2.52	24.11
O'Connor and Arnold 2019	1.10	-3.43	0.337	0.0030	24	0.47	6.30	3.80	6.70
Zhou and Song 2019	1.51	-3.25	0.884	<0.0001	15	2.02	7.95	3.50	7.50
Chen and Arnold 2020	0.21	0.46	0.665	<0.0001	22	0.95	3.14	4.46	12.00
Palma and Richard 2020	0.68	-2.02	0.586	0.0002	18	0.78	6.30	4.56	10.80
Wan and Chen 2020	1.22	-0.68	0.839	<0.0001	30	1.37	5.22	2.37	5.06
Wang and Song 2020	1.15	-1.69	0.790	0.0003	11	2.02	6.68	3.20	6.80
Wasswa and Zeng 2020	0.41	-0.46	0.535	<0.0001	99	0.92	5.15	3.34	10.77
Zhou and Niu 2020	0.49	-0.46	0.656	0.0272	7	0.74	3.31	3.02	8.09
Leresche and Rosario-Ortiz 2021	0.40	0.10	0.875	<0.0001	18	1.00	13.17	4.62	29.94
Li and Tian 2021	0.25	0.26	0.367	0.0013	25	0.23	3.19	1.69	9.79
Wan and Chen 2021b	1.38	-0.60	0.561	0.0013	15	2.24	8.36	2.30	4.70
Zhou and Chen 2021	0.18	0.83	0.694	<0.0001	18	0.40	2.33	0.76	8.23
Liao and Yuan 2022	-1.01	9.07	0.225	0.0255	22	0.88	4.56	5.69	7.43
Wan and Chen 2022a	0.89	0.29	0.847	<0.0001	40	2.22	8.28	2.37	8.60
Wan and Chen 2022b	0.89	0.27	0.577	0.0041	12	1.53	5.08	2.15	6.04
Wasswa and Zeng 2022	0.15	1.24	0.150	0.0002	86	0.25	6.13	2.24	25.96
Xu 2022	1.42	-2.86	0.571	0.0185	9	0.19	3.18	2.23	3.86
Berg and Remucal 2023	0.34	1.04	0.115	0.0181	48	1.07	8.27	4.93	13.03
Buckley and Rosario-Ortiz 2023	-0.73	4.90	0.335	<0.0001	52	0.68	10.86	0.42	7.90
Guo and Yoshimura 2023	0.57	-0.44	0.556	<0.0001	60	1.44	6.21	3.79	11.50
Kong and Song 2023	0.35	1.89	0.342	0.0221	15	0.46	5.75	1.77	11.83
Li and Nah 2024	0.27	0.18	0.456	0.0029	17	0.78	6.14	3.97	16.54
Wasswa and Zeng 2024	0.07	1.71	0.055	<0.0001	368	0.82	4.88	2.72	18.84

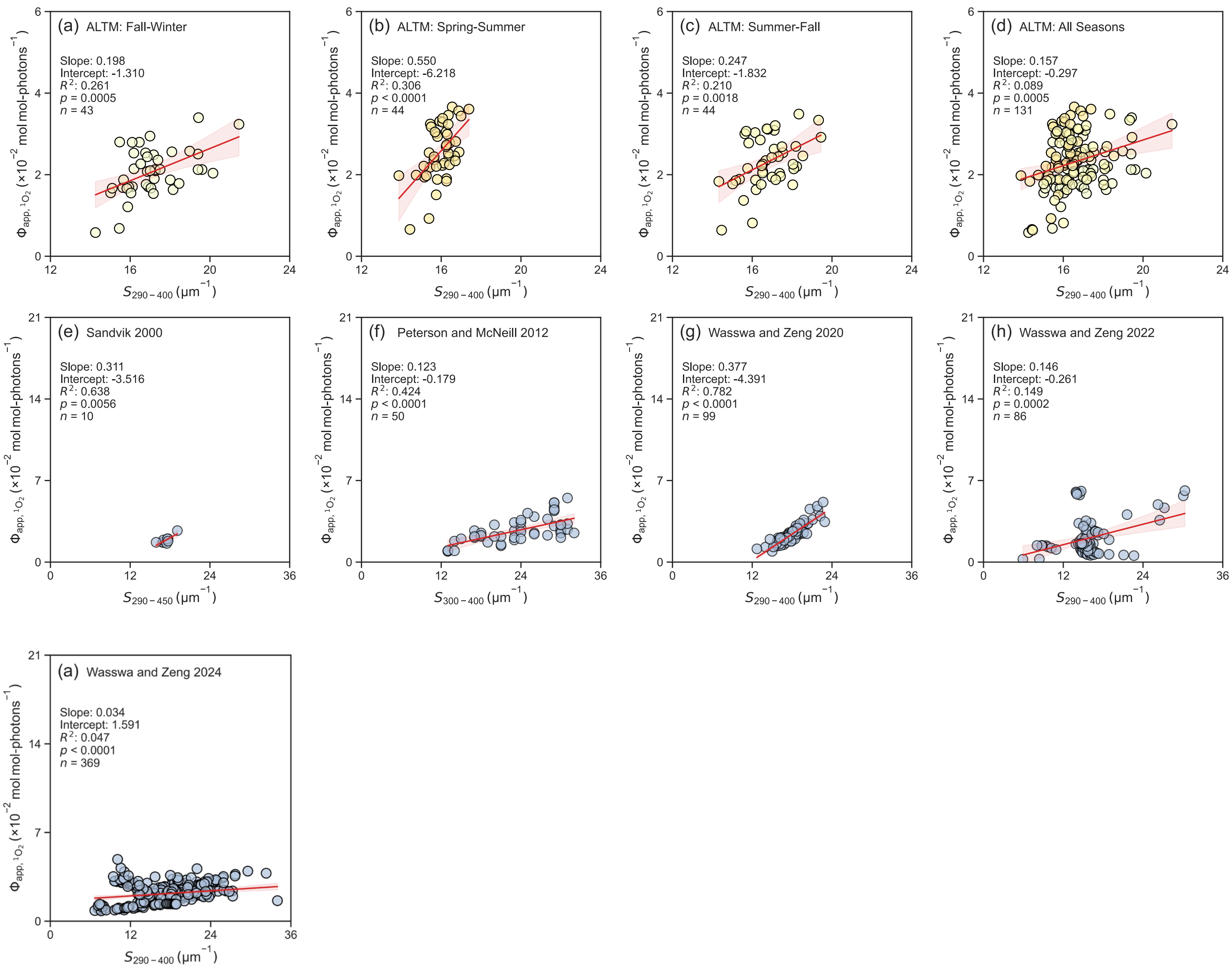
(a) ALTM: Fall-Winter
Slope: 0.198
Intercept: -1.310
R^2: 0.261
p = 0.0005
n = 43
(b) ALTM: Spring-Summer
Slope: 0.550
Intercept: -6.218
R^2: 0.306
p < 0.0001
n = 44
(c) ALTM: Summer-Fall
Slope: 0.247
Intercept: -1.832
R^2: 0.210
p = 0.0018
n = 44
(d) ALTM: All Seasons
Slope: 0.157
Intercept: -0.297
R^2: 0.089
p = 0.0005
n = 131
(e) Sandvik 2000
Slope: 0.311
Intercept: -3.516
R^2: 0.638
p = 0.0056
n = 10
(f) Peterson and McNeill 2012
Slope: 0.123
Intercept: -0.179
R^2: 0.424
p < 0.0001
n = 50
(g) Wasswa and Zeng 2020
Slope: 0.377
Intercept: -4.391
R^2: 0.782
p < 0.0001
n = 99
(h) Wasswa and Zeng 2022
Slope: 0.146
Intercept: -0.261
R^2: 0.149
p = 0.0002
n = 86
(a) Wasswa and Zeng 2024
Slope: 0.034
Intercept: 1.591
R^2: 0.047
p < 0.0001
n = 369
$\Phi_{app, ^1O_2}$ ($\times 10^{-2}$ mol mol-photons^{-1})
$S_{290-400}$ (μm^{-1})
$S_{290-450}$ (μm^{-1})
$S_{300-400}$ (μm^{-1})

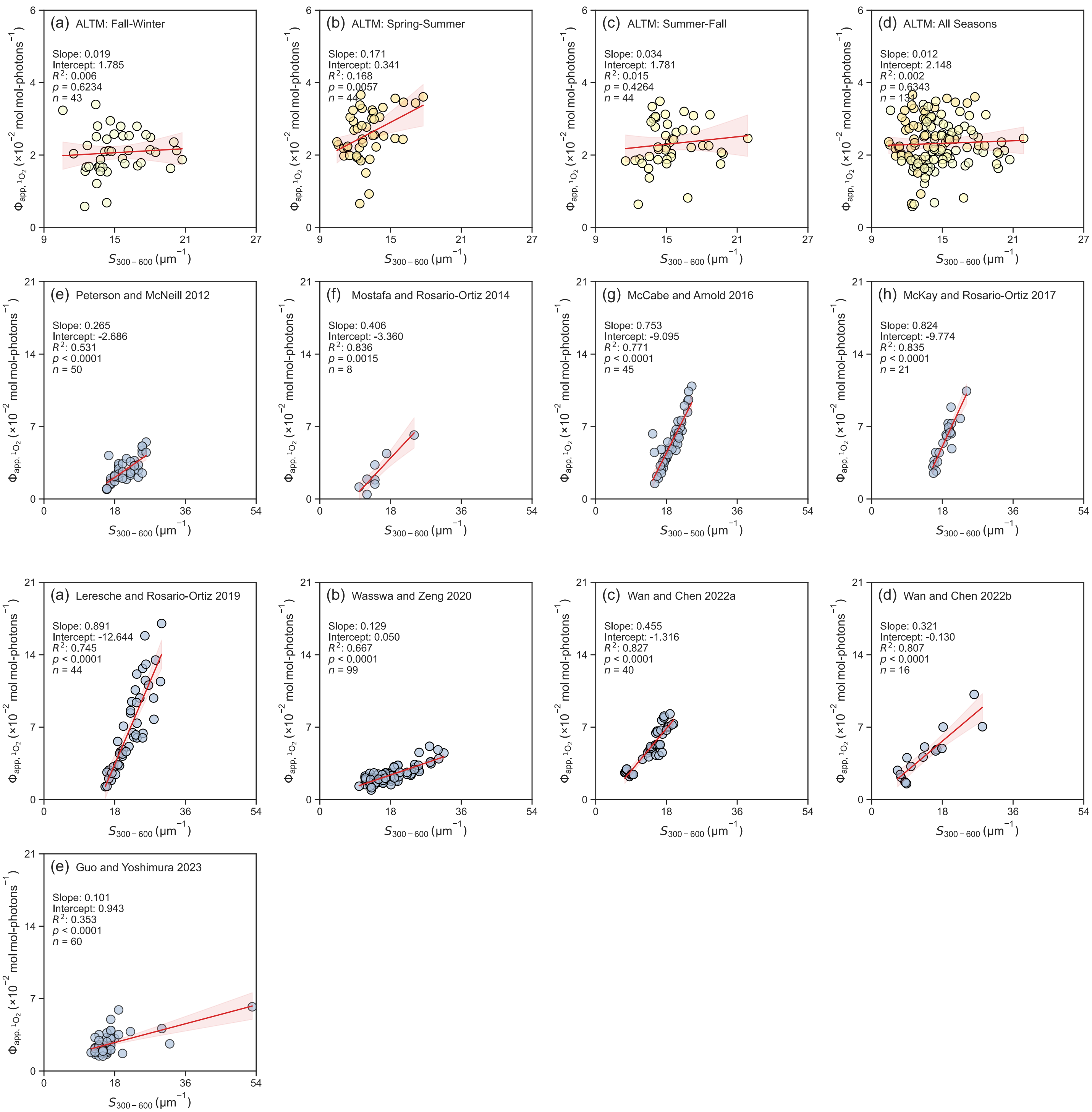

Figure S22. Linear regression between $\Phi_{app, ^1O_2}$ and spectral slope coefficients (e.g., $S_{290-400}$ and $S_{300-600}$) for ALTM samples and for samples from previous work. Only studies with at least seven data points were included in the regression analysis. The red solid line denotes the regression line, and the shaded area represents the 95% confidence interval. Marker colors for ALTM samples correspond to those in Figure 3.

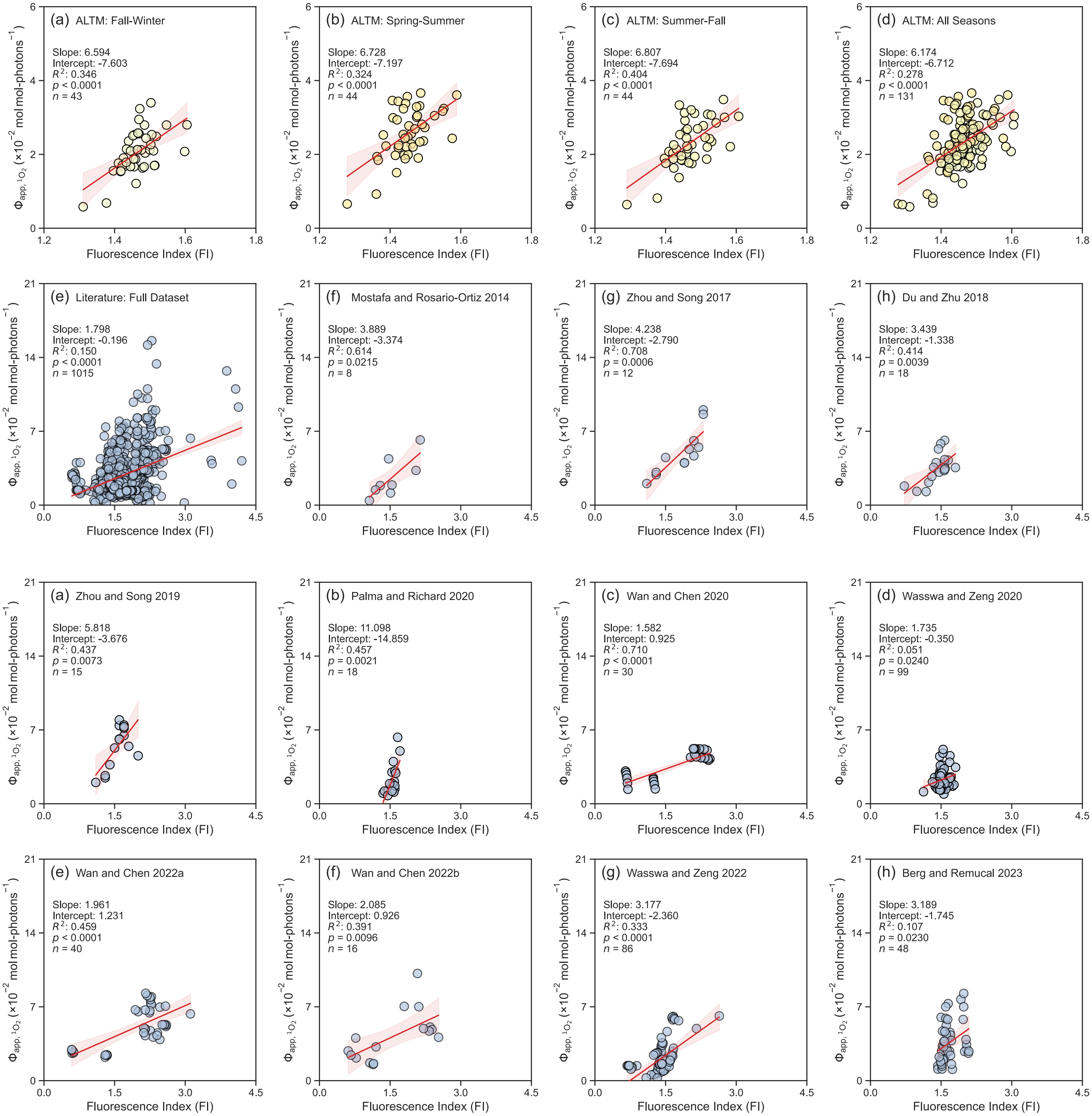

(a) ALTM: Fall-Winter
Slope: 6.594
Intercept: -7.603
R^2: 0.346
p < 0.0001
n = 43
(b) ALTM: Spring-Summer
Slope: 6.728
Intercept: -7.197
R^2: 0.324
p < 0.0001
n = 44
(c) ALTM: Summer-Fall
Slope: 6.807
Intercept: -7.694
R^2: 0.404
p < 0.0001
n = 44
(d) ALTM: All Seasons
Slope: 6.174
Intercept: -6.712
R^2: 0.278
p < 0.0001
n = 131
(e) Literature: Full Dataset
Slope: 1.798
Intercept: -0.196
R^2: 0.150
p < 0.0001
n = 1015
(f) Mostafa and Rosario-Ortiz 2014
Slope: 3.889
Intercept: -3.374
R^2: 0.614
p = 0.0215
n = 8
(g) Zhou and Song 2017
Slope: 4.238
Intercept: -2.790
R^2: 0.708
p = 0.0006
n = 12
(h) Du and Zhu 2018
Slope: 3.439
Intercept: -1.338
R^2: 0.414
p = 0.0039
n = 18
(a) Zhou and Song 2019
Slope: 5.818
Intercept: -3.676
R^2: 0.437
p = 0.0073
n = 15
(b) Palma and Richard 2020
Slope: 11.098
Intercept: -14.859
R^2: 0.457
p = 0.0021
n = 18
(c) Wan and Chen 2020
Slope: 1.582
Intercept: 0.925
R^2: 0.710
p < 0.0001
n = 30
(d) Wasswa and Zeng 2020
Slope: 1.735
Intercept: -0.350
R^2: 0.051
p = 0.0240
n = 99
(e) Wan and Chen 2022a
Slope: 1.961
Intercept: 1.231
R^2: 0.459
p < 0.0001
n = 40
(f) Wan and Chen 2022b
Slope: 2.085
Intercept: 0.926
R^2: 0.391
p = 0.0096
n = 16
(g) Wasswa and Zeng 2022
Slope: 3.177
Intercept: -2.360
R^2: 0.333
p < 0.0001
n = 86
(h) Berg and Remucal 2023
Slope: 3.189
Intercept: -1.745
R^2: 0.107
p = 0.0230
n = 48
$\Phi_{app, {}^1O_2}$ ($\times 10^{-2}$ mol mol-photons^{-1})
Fluorescence Index (FI)

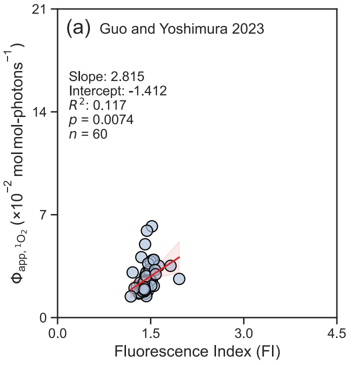

Figure S23. Linear regression between $\Phi_{app,\,^1O_2}$ and fluorescence index (FI) for ALTM samples and for samples from previous work (Table S11). Only studies with at least seven data points were included in the regression analysis. The red solid line denotes the regression line, and the shaded area represents the 95% confidence interval. Marker colors for ALTM samples correspond to those in Figure 3.

**Table S11.** Summary statistics of linear regression between $\Phi_{app,\,^1O_2}$ and fluorescence index (FI) from the literature									
Reference (See details in Table S18)	Slope	Intercept	R^2	p-value	n	Minimum $\Phi_{app,\,^1O_2}$ ($\times 10^{-2}$ mol mol-photons^{-1})	Maximum $\Phi_{app,\,^1O_2}$ ($\times 10^{-2}$ mol mol-photons^{-1})	Min FI	Max FI
Full Dataset	1.80	-0.20	0.150	<0.0001	1015	0.25	10.16	0.59	3.11
Mostafa and Rosario-Ortiz 2014	3.89	-3.37	0.614	0.0215	8	0.44	6.18	1.06	2.14
Zhou and Song 2017	4.24	-2.79	0.708	0.0006	12	2.05	9.03	1.10	2.30
Du and Zhu 2018	3.44	-1.34	0.414	0.0039	18	1.28	6.14	0.72	1.80
Zhou and Song 2019	5.82	-3.68	0.437	0.0073	15	2.02	7.95	1.10	2.00
Palma and Richard 2020	11.10	-14.86	0.457	0.0021	18	0.78	6.30	1.35	1.71
Wan and Chen 2020	1.58	0.93	0.710	<0.0001	30	1.37	5.22	0.65	2.44
Wasswa and Zeng 2020	1.74	-0.35	0.051	0.0240	99	0.92	5.15	1.13	1.81
Wan and Chen 2022a	1.96	1.23	0.459	<0.0001	40	2.22	8.28	0.59	3.11
Wan and Chen 2022b	2.09	0.93	0.391	0.0096	16	1.53	10.16	0.61	2.53
Wasswa and Zeng 2022	3.18	-2.36	0.333	<0.0001	86	0.25	6.13	0.68	2.64
Berg and Remucal 2023	3.19	-1.75	0.107	0.0230	48	1.07	8.27	1.43	2.09
Guo and Yoshimura 2023	2.82	-1.41	0.117	0.0074	60	1.44	6.21	1.18	1.96

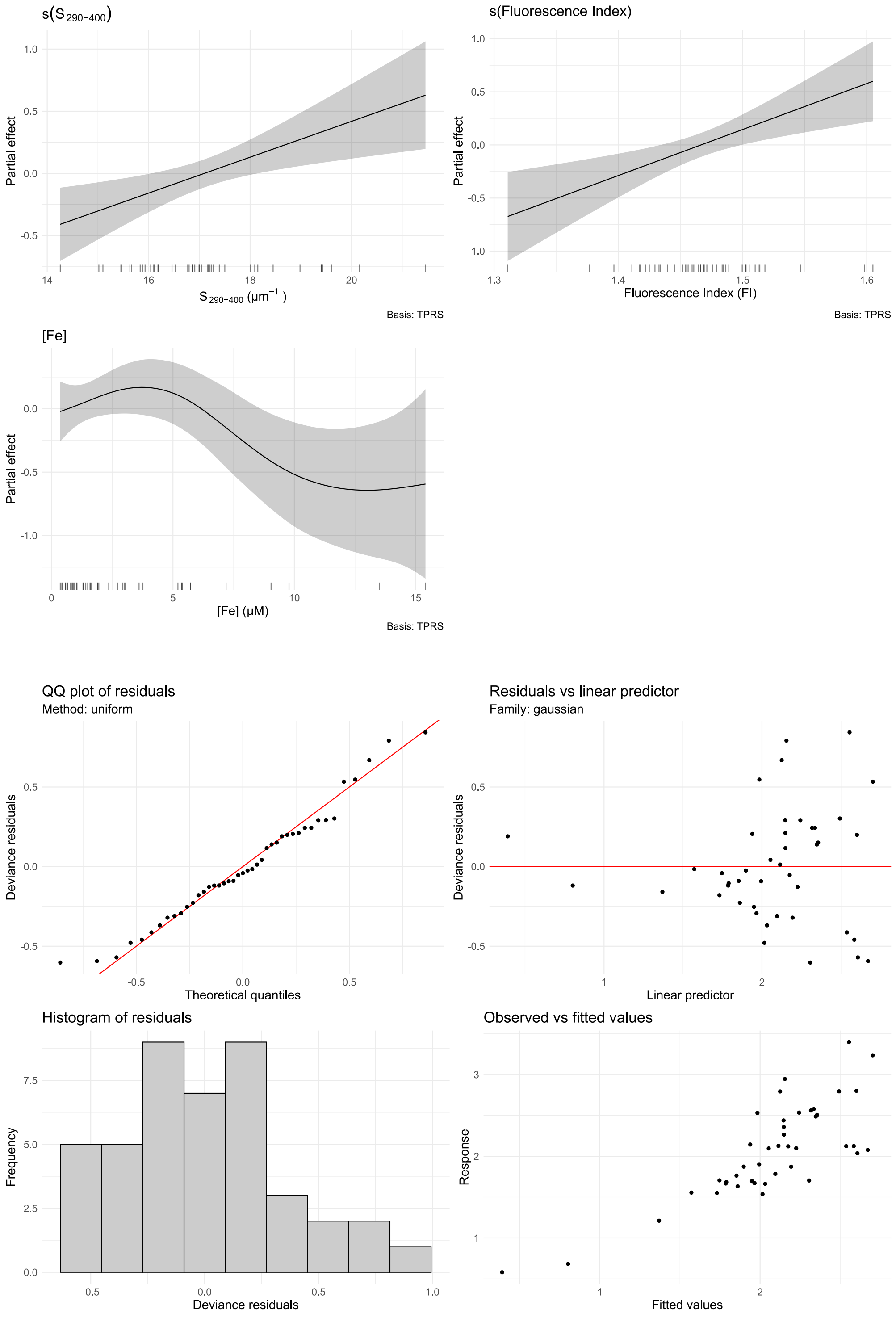

Figure S24. Generalized additive modeling of $\Phi_{app,\,^1O_2}$ for ALTM samples collected during the fall-winter season. Each panel in the partial smooth plots represents a predictor, with the smooth term shown as a solid line, shaded confidence intervals, and rug plots along the *x*-axis indicating the distribution of observed data points. Diagnostic plots evaluate residuals and model fit, including a *Q-Q* plot of residuals, a scale-location plot to assess homoscedasticity, residuals versus fitted values to identify nonlinearity or patterns, and residuals versus leverage to detect influential observations.

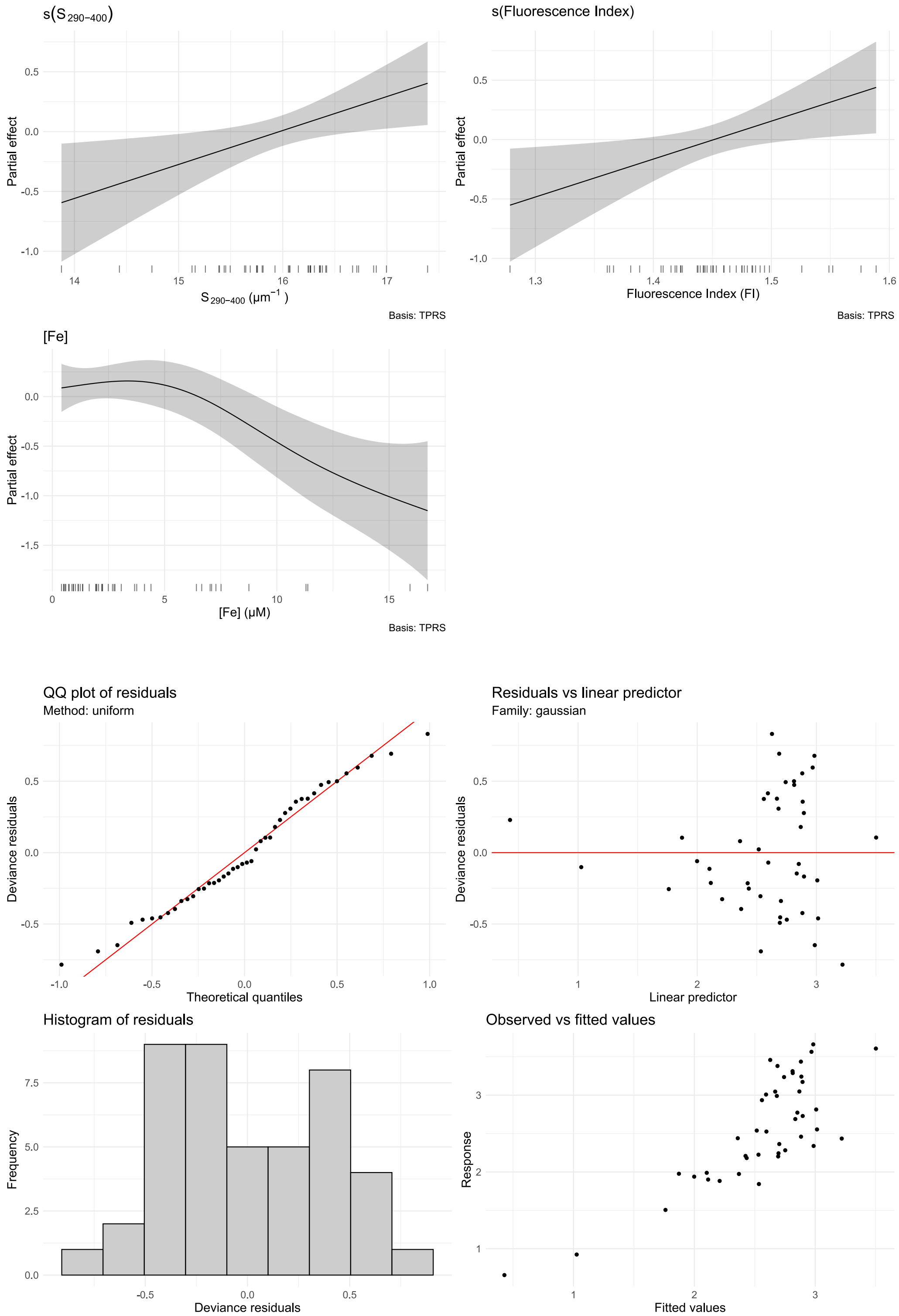

Figure S25. Generalized additive modeling of $\Phi_{app,\ ^1O_2}$ for ALTM samples collected during the spring-summer season. Each panel in the partial smooth plots represents a predictor, with the smooth term shown as a solid line, shaded confidence intervals, and rug plots along the *x*-axis indicating the distribution of observed data points. Diagnostic plots evaluate residuals and model fit, including a *Q-Q* plot of residuals, a scale-location plot to assess homoscedasticity, residuals versus fitted values to identify nonlinearity or patterns, and residuals versus leverage to detect influential observations.

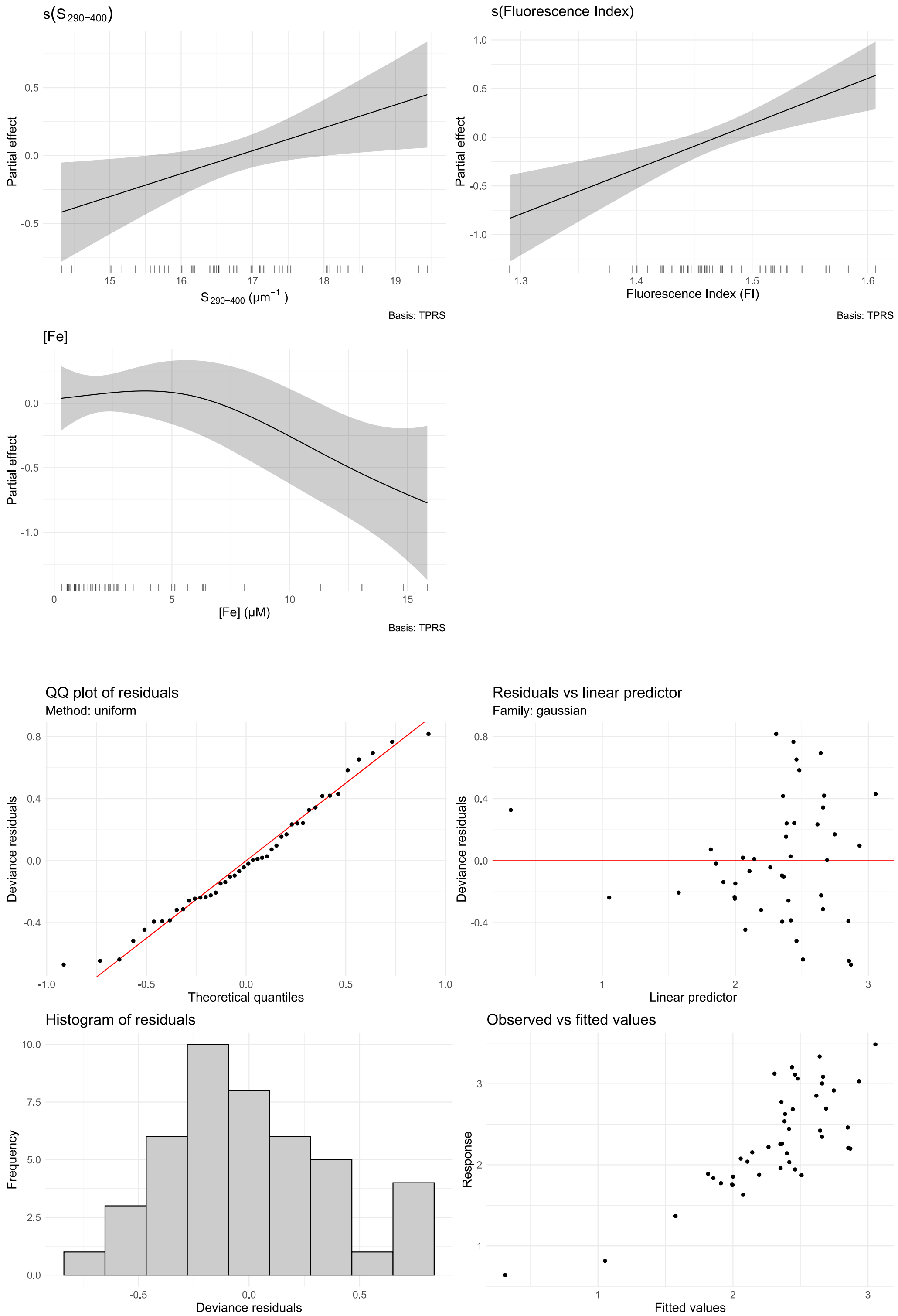

Figure S26. Generalized additive modeling of $\Phi_{app,\ ^1O_2}$ for ALTM samples collected during the summer-fall season. Each panel in the partial smooth plots represents a predictor, with the smooth term shown as a solid line, shaded confidence intervals, and rug plots along the *x*-axis indicating the distribution of observed data points. Diagnostic plots evaluate residuals and model fit, including a *Q-Q* plot of residuals, a scale-location plot to assess homoscedasticity, residuals versus fitted values to identify nonlinearity or patterns, and residuals versus leverage to detect influential observations.

Table S12. Summary statistics of generalized additive modeling

Smooth terms	Effective Degrees of Freedom	*F*-Statistics	*p*-value	R^2 Adjusted	Deviance Explained	Scale Estimate
Fall-Winter Formula: y ~ s($S_{290\text{-}400}$) + s(Fluorescence Index) + s([Fe])						
s($S_{290\text{-}400}$)	1.000	8.708	0.00547	0.579	62.9%	0.14276
s(Fluorescence Index)	1.000	10.734	0.00229			
s([Fe])	2.920	3.131	0.04312			
	Estimate	Standard Error	*t* value	Pr(>$\lvert t \rvert$)		
(Intercept)	2.06768	0.05762	35.88	<2e-16		
Spring-Summer Formula: y ~ s($S_{290\text{-}400}$) + s(Fluorescence Index) + s([Fe])						
s($S_{290\text{-}400}$)	1.000	5.970	0.01918	0.597	63.9%	0.18851
s(Fluorescence Index)	1.000	5.581	0.02324			
s([Fe])	2.453	5.534	0.00302			
	Estimate	Standard Error	*t* value	Pr(>$\lvert t \rvert$)		
(Intercept)	2.56784	0.06545	39.23	<2e-16		
Summer-Fall Formula: y ~ s($S_{290\text{-}400}$) + s(Fluorescence Index) + s([Fe])						
s($S_{290\text{-}400}$)	1.000	5.600	0.02302	0.589	62.8%	0.16141
s(Fluorescence Index)	1.000	14.532	0.00048			
s([Fe])	2.007	3.749	0.03692			
	Estimate	Standard Error	*t* value	Pr(>$\lvert t \rvert$)		
(Intercept)	2.31293	0.06057	38.19	<2e-16		

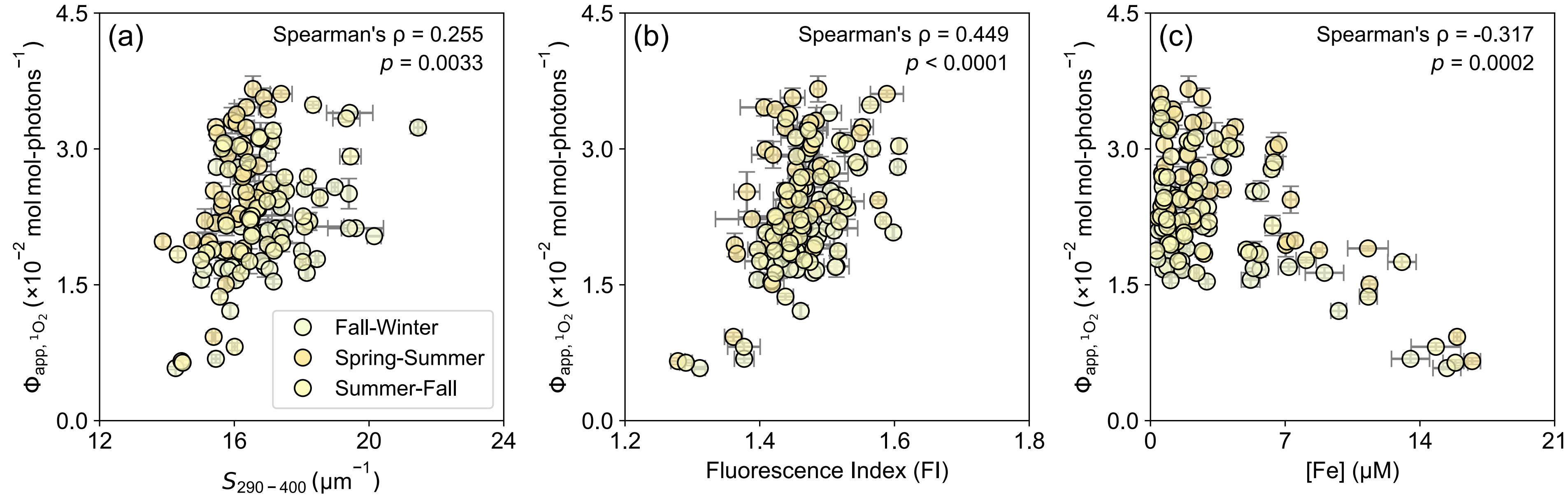

Figure S27. Spearman's correlations between $\Phi_{app, ^1O_2}$ and **(a)** $S_{290\text{-}400}$, **(b)** fluorescence index (FI), or **(c)** total dissolved iron ([Fe]) across ALTM lakes by season. Marker colors correspond to those in Figure 3. Error bars represent the standard deviations from duplicate measurements; where absent, bars fall within symbols.

14. Comparisons of water chemistry and optical properties of ALTM samples by browning status

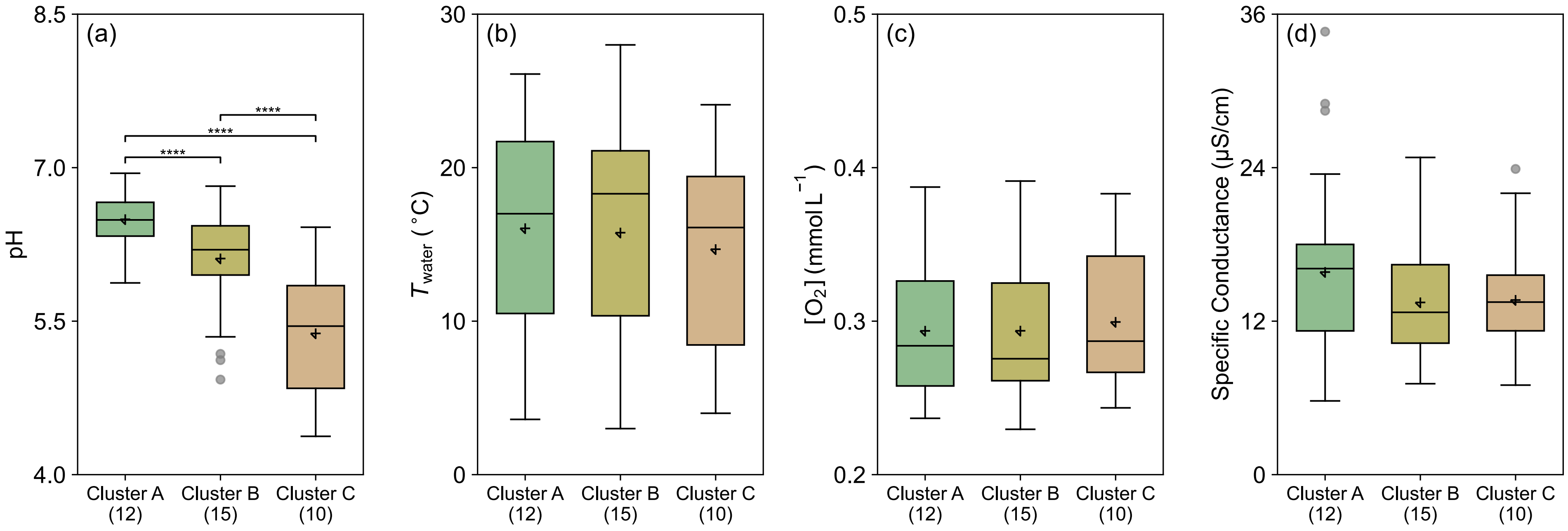

Figure S28. Multiple comparisons of **(a)** pH, **(b)** water temperature, **(c)** dissolved oxygen, and **(d)** specific conductance across ALTM lake clusters. Lakes in clusters A, B, and C were operationally designated as experiencing mild, moderate, and intense browning, respectively. Each box spans the 25th to 75th percentiles, with whiskers extending to 1.5 times the interquartile range below the 25th and above the 75th percentiles. The centerline and "+" sign mark the median and mean, respectively. Gray circles represent outliers. Numbers in parentheses represent the number of lakes in each cluster. For multiple comparisons, a Kruskal-Wallis test was first performed to determine whether statistically significant differences existed among groups. If significant, pairwise Mann-Whitney *U* tests were performed, with significant differences marked by asterisks as "*" (p<0.05), "**" (p<0.01), "***" (p<0.001), or "****" (p<0.0001).

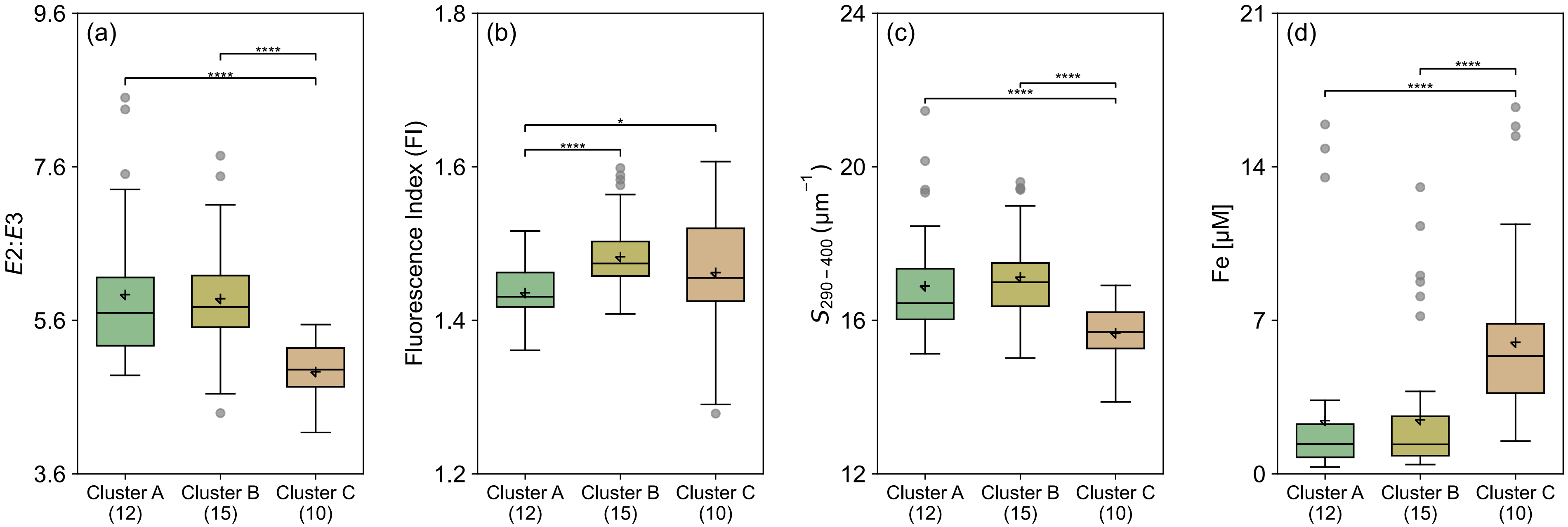

Figure S29. Multiple comparisons of **(a)** *E2:E3*, **(b)** fluorescence index (FI), **(c)** $S_{290\text{-}400}$, and **(d)** total dissolved iron ([Fe]) across ALTM lake clusters. Lakes in clusters A, B, and C were operationally designated as experiencing mild, moderate, and intense browning, respectively. Each box spans the 25th to 75th percentiles, with whiskers extending to 1.5 times the interquartile range below the 25th and above the 75th percentiles. The centerline and "+" sign mark the median and mean, respectively. Gray circles represent outliers. Numbers in parentheses represent the number of lakes in each cluster. For multiple comparisons, a Kruskal-Wallis test was first performed to determine whether statistically significant differences existed among groups. If significant, pairwise Mann-Whitney *U* tests were performed, with significant differences marked by asterisks as "*" (p<0.05), "**" (p<0.01), "***" (p<0.001), or "****" (p<0.0001).

15. Steady-state concentrations and depth profiles of $^1O_2$ in ALTM lakes

Table S13. $[^1O_2]^{euphotic\ zone}_{ss,\ daily\ average}$ in ALTM lakes

Site Name	Sampling Date	$[^1O_2]^{euphotic\ zone}_{ss,\ daily\ average}$ ($\times10^{-15}$ M)	Sampling Date	$[^1O_2]^{euphotic\ zone}_{ss,\ daily\ average}$ ($\times10^{-15}$ M)	Sampling Date	$[^1O_2]^{euphotic\ zone}_{ss,\ daily\ average}$ ($\times10^{-15}$ M)
Avalanche Lake	10/17/2022	1.50±0.28	6/2/2023	3.80±0.46	9/25/2023	3.79±0.67
Lake Colden	10/17/2022	2.01±0.11	6/2/2023	4.50±0.51	9/25/2023	3.63±0.60
Brook Trout Lake	10/26/2022	0.54±0.04	5/22/2023	1.61±0.10	9/5/2023	1.95±0.30
Squash Pond Outlet	10/31/2022	2.89±0.38	5/15/2023	8.70±0.85	9/18/2023	6.49±0.94
Squash Pond	10/31/2022	2.95±0.41	5/15/2023	8.42±0.40	9/18/2023	9.51±1.51
Constable Pond Outlet	10/31/2022	1.75±0.15	5/15/2023	5.31±0.59	9/18/2023	5.59±0.86
Constable Pond	10/31/2022	1.74±0.13	5/15/2023	5.33±0.16	9/18/2023	5.61±0.88
East Copperas Pond	11/1/2022	1.80±0.12	6/27/2023	6.37±0.05	9/15/2023	5.48±0.63
Little Echo Pond	11/1/2022	1.13±0.16	6/29/2023	2.67±0.05	9/21/2023	2.80±0.56
Lake Rondaxe	11/2/2022	0.98±0.15	6/19/2023	4.27±0.18	9/23/2023	3.65±0.27
Cascade Lake	11/2/2022	1.46±0.15	6/16/2023	2.96±0.16	9/18/2023	2.28±0.35
Cascade Lake Outlet	11/2/2022	1.47±0.14	6/16/2023	2.98±0.32	9/18/2023	2.64±0.47
Dart Lake	11/2/2022	0.89±0.11	5/23/2023	4.00±0.38	9/22/2023	2.88±0.40
Woods Lake	11/2/2022	1.26±0.18	6/16/2023	4.60±0.20	9/18/2023	5.31±0.39
Willys Lake	11/3/2022	1.02±0.12	5/23/2023	2.83±0.18	9/19/2023	2.95±0.32
Loon Hollow Pond	11/3/2022	1.33±0.19	5/23/2023	3.69±0.34	9/19/2023	4.19±0.61
Little Clear Pond	11/7/2022	0.37±0.02	6/22/2023	1.72±0.04	9/12/2023	1.24±0.07
Black Pond	11/7/2022	0.54±0.07	6/29/2023	2.40±0.27	9/21/2023	2.11±0.29
Indian Lake	11/8/2022	1.72±0.24	5/22/2023	5.22±0.61	9/5/2023	7.64±1.11
Squaw Lake	11/8/2022	1.03±0.10	5/22/2023	3.23±0.15	9/5/2023	3.28±0.41
Queer Lake	11/9/2022	0.84±0.04	6/6/2023	3.86±0.34	9/13/2023	2.84±0.40
Big Hope Pond	11/10/2022	1.43±0.22	6/23/2023	6.88±0.08	9/21/2023	4.23±0.38
G Lake	11/10/2022	0.53±0.06	6/29/2023	2.00±0.18	9/12/2023	1.80±0.16
West Pond Outlet	11/14/2022	0.94±0.14	6/16/2023	3.66±0.37	9/4/2023	11.32±1.40
West Pond	11/14/2022	0.93±0.05	6/16/2023	3.64±0.37	9/4/2023	3.77±0.51
Big Moose Lake	11/14/2022	1.20±0.11	6/16/2023	5.10±0.26	9/4/2023	5.60±0.84
Moss Lake	11/14/2022	0.77±0.04	5/25/2023	3.54±0.43	9/26/2023	2.61±0.34
Bubb Lake	11/14/2022	0.75±0.06	5/25/2023	3.15±0.33	9/4/2023	1.84±0.13
Bubb Lake Outlet	11/14/2022	0.77±0.05	5/25/2023	3.25±0.42	9/4/2023	1.65±0.10
Limekiln Lake	11/15/2022	0.74±0.10	6/6/2023	4.64±0.54	9/4/2023	3.06±0.28
Raquette Lake Reservoir	11/15/2022	1.72±0.24	5/23/2023	4.04±0.24	9/26/2023	5.24±0.73
Sagamore Lake	11/15/2022	0.92±0.06	6/20/2023	3.49±0.36	9/29/2023	3.98±0.64
Arbutus Pond	11/15/2022	0.59±0.06	6/23/2023	2.69±0.28	9/26/2023	2.56±0.21
Clear Pond	11/15/2022	0.48±0.05	6/23/2023	2.35±0.16	9/26/2023	1.91±0.26
Heart Lake	11/15/2022	0.44±0.06	6/13/2023	1.64±0.14	9/5/2023	1.63±0.15
Grass Pond	11/17/2022	0.78±0.12	5/16/2023	2.63±0.05	9/4/2023	3.41±0.51
Middle Branch Lake	11/17/2022	0.61±0.08	5/16/2023	2.35±0.28	9/4/2023	2.82±0.52
Middle Settlement Lake	11/17/2022	0.89±0.07	5/16/2023	3.32±0.36	9/4/2023	3.78±0.55
South Lake	11/28/2022	0.51±0.07	6/26/2023	2.46±0.08	9/6/2023	2.49±0.31
North Lake	11/28/2022	0.98±0.06	6/26/2023	3.48±0.47	9/6/2023	4.75±0.35
Otter Lake Outlet	11/29/2022	0.87±0.03	6/5/2023	4.20±0.46	9/14/2023	2.09±0.26
Otter Lake	11/29/2022	0.86±0.04	6/5/2023	4.34±0.48	9/14/2023	2.11±0.19
Jockeybush Lake	11/29/2022	0.56±0.05	6/5/2023	2.44±0.12	9/12/2023	2.80±0.34

Table S14. $[^1O_2]^{epilimnion}_{ss,\ daily\ average}$ in ALTM lakes						
Site Name	Sampling Date	$[^1O_2]^{epilimnion}_{ss,\ daily\ average}$ ($\times10^{-15}$ M)	Sampling Date	$[^1O_2]^{epilimnion}_{ss,\ daily\ average}$ ($\times10^{-15}$ M)	Sampling Date	$[^1O_2]^{epilimnion}_{ss,\ daily\ average}$ ($\times10^{-15}$ M)
Avalanche Lake	10/17/2022	3.26±0.61	6/2/2023	11.25±1.41	9/25/2023	5.89±0.89
Lake Colden	10/17/2022	2.93±0.16	6/2/2023	9.36±1.07	9/25/2023	4.38±0.61
Brook Trout Lake	10/26/2022	1.31±0.11	5/22/2023	3.82±0.23	9/5/2023	2.60±0.35
Squash Pond Outlet	10/31/2022	4.95±0.65	5/15/2023	15.57±1.54	9/18/2023	9.42±1.14
Squash Pond	10/31/2022	5.23±0.74	5/15/2023	15.17±0.74	9/18/2023	9.70±1.22
Constable Pond Outlet	10/31/2022	2.24±0.19	5/15/2023	6.97±0.78	9/18/2023	4.25±0.50
Constable Pond	10/31/2022	2.24±0.16	5/15/2023	6.98±0.21	9/18/2023	4.34±0.52
East Copperas Pond	11/1/2022	2.67±0.18	6/27/2023	9.12±0.09	9/15/2023	5.94±0.40
Little Echo Pond	11/1/2022	2.83±0.41	6/29/2023	7.81±0.18	9/21/2023	5.21±0.80
Lake Rondaxe	11/2/2022	1.28±0.19	6/19/2023	4.48±0.18	9/23/2023	2.39±0.12
Cascade Lake	11/2/2022	1.47±0.15	6/16/2023	4.80±0.27	9/18/2023	2.64±0.37
Cascade Lake Outlet	11/2/2022	1.48±0.15	6/16/2023	4.90±0.53	9/18/2023	2.76±0.44
Dart Lake	11/2/2022	1.19±0.14	5/23/2023	4.30±0.41	9/22/2023	2.19±0.24
Woods Lake	11/2/2022	2.04±0.29	6/16/2023	7.76±0.35	9/18/2023	4.42±0.18
Willys Lake	11/3/2022	2.53±0.29	5/23/2023	7.17±0.47	9/19/2023	4.67±0.41
Loon Hollow Pond	11/3/2022	2.75±0.40	5/23/2023	8.72±0.81	9/19/2023	5.31±0.62
Little Clear Pond	11/7/2022	1.29±0.07	6/22/2023	5.36±0.14	9/12/2023	3.21±0.15
Black Pond	11/7/2022	1.24±0.17	6/29/2023	4.47±0.51	9/21/2023	2.64±0.33
Indian Lake	11/8/2022	1.91±0.27	5/22/2023	6.64±0.78	9/5/2023	4.54±0.52
Squaw Lake	11/8/2022	2.01±0.20	5/22/2023	6.44±0.31	9/5/2023	4.53±0.51
Queer Lake	11/9/2022	1.39±0.07	6/6/2023	5.84±0.52	9/13/2023	3.34±0.42
Big Hope Pond	11/10/2022	1.78±0.27	6/23/2023	8.05±0.09	9/21/2023	4.01±0.26
G Lake	11/10/2022	1.29±0.16	6/29/2023	4.40±0.39	9/12/2023	2.83±0.20
West Pond Outlet	11/14/2022	1.57±0.24	6/16/2023	6.11±0.62	9/4/2023	4.09±0.31
West Pond	11/14/2022	1.53±0.08	6/16/2023	5.92±0.60	9/4/2023	4.03±0.45
Big Moose Lake	11/14/2022	0.86±0.08	6/16/2023	3.16±0.16	9/4/2023	2.17±0.28
Moss Lake	11/14/2022	1.16±0.07	5/25/2023	5.24±0.65	9/26/2023	2.69±0.31
Bubb Lake	11/14/2022	1.18±0.09	5/25/2023	4.98±0.52	9/4/2023	3.23±0.17
Bubb Lake Outlet	11/14/2022	1.22±0.09	5/25/2023	5.24±0.69	9/4/2023	3.18±0.14
Limekiln Lake	11/15/2022	0.96±0.13	6/6/2023	3.75±0.43	9/4/2023	2.41±0.17
Raquette Lake Reservoir	11/15/2022	3.99±0.57	5/23/2023	14.31±0.91	9/26/2023	8.19±0.94
Sagamore Lake	11/15/2022	0.60±0.04	6/20/2023	2.50±0.26	9/29/2023	1.28±0.16
Arbutus Pond	11/15/2022	0.82±0.08	6/23/2023	3.31±0.35	9/26/2023	1.74±0.10
Clear Pond	11/15/2022	0.84±0.08	6/23/2023	3.53±0.25	9/26/2023	1.77±0.21
Heart Lake	11/15/2022	1.61±0.22	6/13/2023	5.97±0.53	9/5/2023	4.11±0.34
Grass Pond	11/17/2022	1.86±0.29	5/16/2023	7.70±0.18	9/4/2023	5.20±0.70
Middle Branch Lake	11/17/2022	1.12±0.15	5/16/2023	4.60±0.55	9/4/2023	3.18±0.56
Middle Settlement Lake	11/17/2022	1.80±0.15	5/16/2023	8.01±0.89	9/4/2023	5.69±0.72
South Lake	11/28/2022	0.67±0.09	6/26/2023	3.22±0.10	9/6/2023	2.12±0.26
North Lake	11/28/2022	0.61±0.04	6/26/2023	3.09±0.41	9/6/2023	1.94±0.09
Otter Lake Outlet	11/29/2022	1.38±0.05	6/5/2023	6.39±0.72	9/14/2023	3.89±0.45
Otter Lake	11/29/2022	1.30±0.06	6/5/2023	6.63±0.74	9/14/2023	3.91±0.24
Jockeybush Lake	11/29/2022	1.39±0.12	6/5/2023	7.57±0.41	9/12/2023	4.39±0.49

**Table S15.** $[^1O_2]^{near\text{-}surface}_{ss,\ daily\ average}$ in ALTM lakes						
Site Name	Sampling Date	$[^1O_2]^{near\text{-}surface}_{ss,\ daily\ average}$ ($\times10^{-14}$ M)	Sampling Date	$[^1O_2]^{near\text{-}surface}_{ss,\ daily\ average}$ ($\times10^{-14}$ M)	Sampling Date	$[^1O_2]^{near\text{-}surface}_{ss,\ daily\ average}$ ($\times10^{-14}$ M)
Avalanche Lake	10/17/2022	2.10±0.42	6/2/2023	6.70±0.98	9/25/2023	3.79±0.67
Lake Colden	10/17/2022	2.80±0.20	6/2/2023	8.36±1.11	9/25/2023	3.63±0.60
Brook Trout Lake	10/26/2022	0.76±0.06	5/22/2023	2.66±0.19	9/5/2023	1.95±0.30
Squash Pond Outlet	10/31/2022	2.74±0.40	5/15/2023	11.43±1.37	9/18/2023	6.49±0.94
Squash Pond	10/31/2022	4.18±0.62	5/15/2023	12.10±0.89	9/18/2023	9.51±1.51
Constable Pond Outlet	10/31/2022	2.23±0.23	5/15/2023	8.64±1.18	9/18/2023	5.59±0.86
Constable Pond	10/31/2022	2.42±0.21	5/15/2023	7.78±0.32	9/18/2023	5.61±0.88
East Copperas Pond	11/1/2022	2.57±0.18	6/27/2023	8.07±0.29	9/15/2023	5.48±0.63
Little Echo Pond	11/1/2022	1.68±0.28	6/29/2023	2.96±0.10	9/21/2023	2.80±0.56
Lake Rondaxe	11/2/2022	1.35±0.23	6/19/2023	5.18±0.31	9/23/2023	3.65±0.27
Cascade Lake	11/2/2022	2.02±0.26	6/16/2023	4.20±0.26	9/18/2023	2.28±0.35
Cascade Lake Outlet	11/2/2022	1.81±0.20	6/16/2023	6.41±0.83	9/18/2023	2.64±0.47
Dart Lake	11/2/2022	1.23±0.16	5/23/2023	4.79±0.49	9/22/2023	2.88±0.40
Woods Lake	11/2/2022	1.75±0.31	6/16/2023	6.17±0.34	9/18/2023	5.31±0.39
Willys Lake	11/3/2022	1.43±0.18	5/23/2023	4.02±0.32	9/19/2023	2.95±0.32
Loon Hollow Pond	11/3/2022	1.83±0.31	5/23/2023	5.11±0.53	9/19/2023	4.19±0.61
Little Clear Pond	11/7/2022	0.51±0.04	6/22/2023	2.04±0.07	9/12/2023	1.24±0.07
Black Pond	11/7/2022	0.75±0.11	6/29/2023	2.98±0.38	9/21/2023	2.11±0.29
Indian Lake	11/8/2022	2.37±0.38	5/22/2023	10.35±1.48	9/5/2023	7.64±1.11
Squaw Lake	11/8/2022	1.22±0.13	5/22/2023	4.65±0.32	9/5/2023	3.28±0.41
Queer Lake	11/9/2022	1.16±0.08	6/6/2023	5.04±0.55	9/13/2023	2.84±0.40
Big Hope Pond	11/10/2022	2.00±0.34	6/23/2023	8.84±0.05	9/21/2023	4.23±0.38
G Lake	11/10/2022	0.74±0.10	6/29/2023	2.71±0.25	9/12/2023	1.80±0.16
West Pond Outlet	11/14/2022	2.37±0.41	6/16/2023	11.17±1.44	9/4/2023	11.32±1.40
West Pond	11/14/2022	1.28±0.09	6/16/2023	4.72±0.53	9/4/2023	3.77±0.51
Big Moose Lake	11/14/2022	1.64±0.18	6/16/2023	6.95±0.46	9/4/2023	5.60±0.84
Moss Lake	11/14/2022	1.06±0.08	5/25/2023	4.41±0.62	9/26/2023	2.61±0.34
Bubb Lake	11/14/2022	0.64±0.06	5/25/2023	2.83±0.33	9/4/2023	1.84±0.13
Bubb Lake Outlet	11/14/2022	0.72±0.06	5/25/2023	3.62±0.54	9/4/2023	1.65±0.10
Limekiln Lake	11/15/2022	1.02±0.14	6/6/2023	5.42±0.77	9/4/2023	3.06±0.28
Raquette Lake Reservoir	11/15/2022	2.45±0.39	5/23/2023	8.26±0.71	9/26/2023	5.24±0.73
Sagamore Lake	11/15/2022	1.28±0.10	6/20/2023	7.91±1.03	9/29/2023	3.98±0.64
Arbutus Pond	11/15/2022	0.81±0.09	6/23/2023	4.54±0.57	9/26/2023	2.56±0.21
Clear Pond	11/15/2022	0.66±0.07	6/23/2023	3.04±0.24	9/26/2023	1.91±0.26
Heart Lake	11/15/2022	0.62±0.09	6/13/2023	2.27±0.22	9/5/2023	1.63±0.15
Grass Pond	11/17/2022	1.06±0.18	5/16/2023	3.65±0.16	9/4/2023	3.41±0.51
Middle Branch Lake	11/17/2022	0.83±0.13	5/16/2023	2.84±0.36	9/4/2023	2.82±0.52
Middle Settlement Lake	11/17/2022	1.21±0.12	5/16/2023	4.73±0.63	9/4/2023	3.78±0.55
South Lake	11/28/2022	0.71±0.10	6/26/2023	3.44±0.15	9/6/2023	2.49±0.31
North Lake	11/28/2022	1.34±0.10	6/26/2023	6.43±1.01	9/6/2023	4.75±0.35
Otter Lake Outlet	11/29/2022	0.99±0.06	6/5/2023	3.29±0.43	9/14/2023	2.09±0.26
Otter Lake	11/29/2022	0.63±0.03	6/5/2023	3.57±0.43	9/14/2023	2.11±0.19
Jockeybush Lake	11/29/2022	0.77±0.07	6/5/2023	4.52±0.32	9/12/2023	2.80±0.34

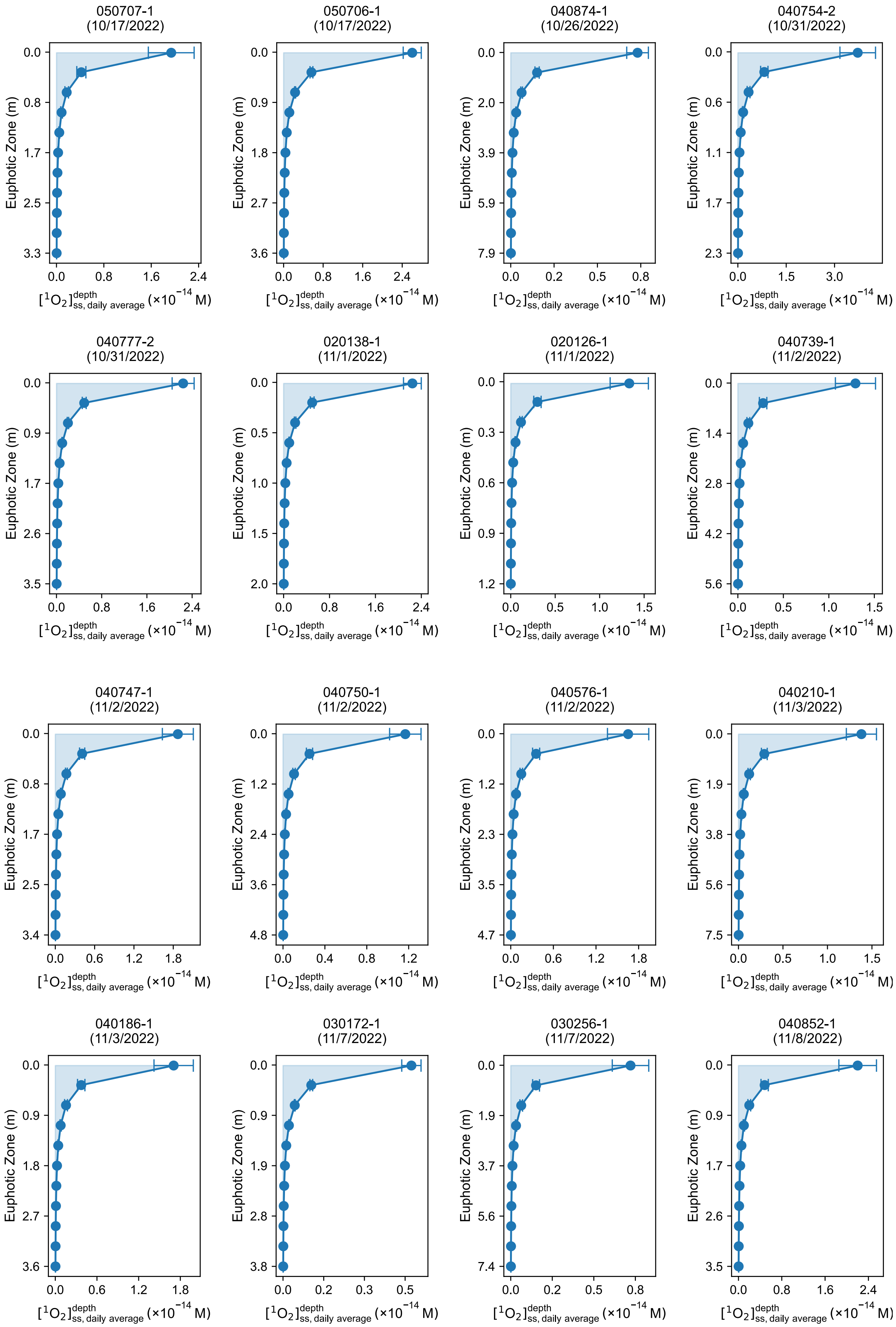
050707-1
(10/17/2022)
050706-1
(10/17/2022)
040874-1
(10/26/2022)
040754-2
(10/31/2022)
040777-2
(10/31/2022)
020138-1
(11/1/2022)
020126-1
(11/1/2022)
040739-1
(11/2/2022)
040747-1
(11/2/2022)
040750-1
(11/2/2022)
040576-1
(11/2/2022)
040210-1
(11/3/2022)
040186-1
(11/3/2022)
030172-1
(11/7/2022)
030256-1
(11/7/2022)
040852-1
(11/8/2022)
Euphotic Zone (m)
$[^1O_2]^{depth}_{ss,\ daily\ average}$ ($\times 10^{-14}$ M)

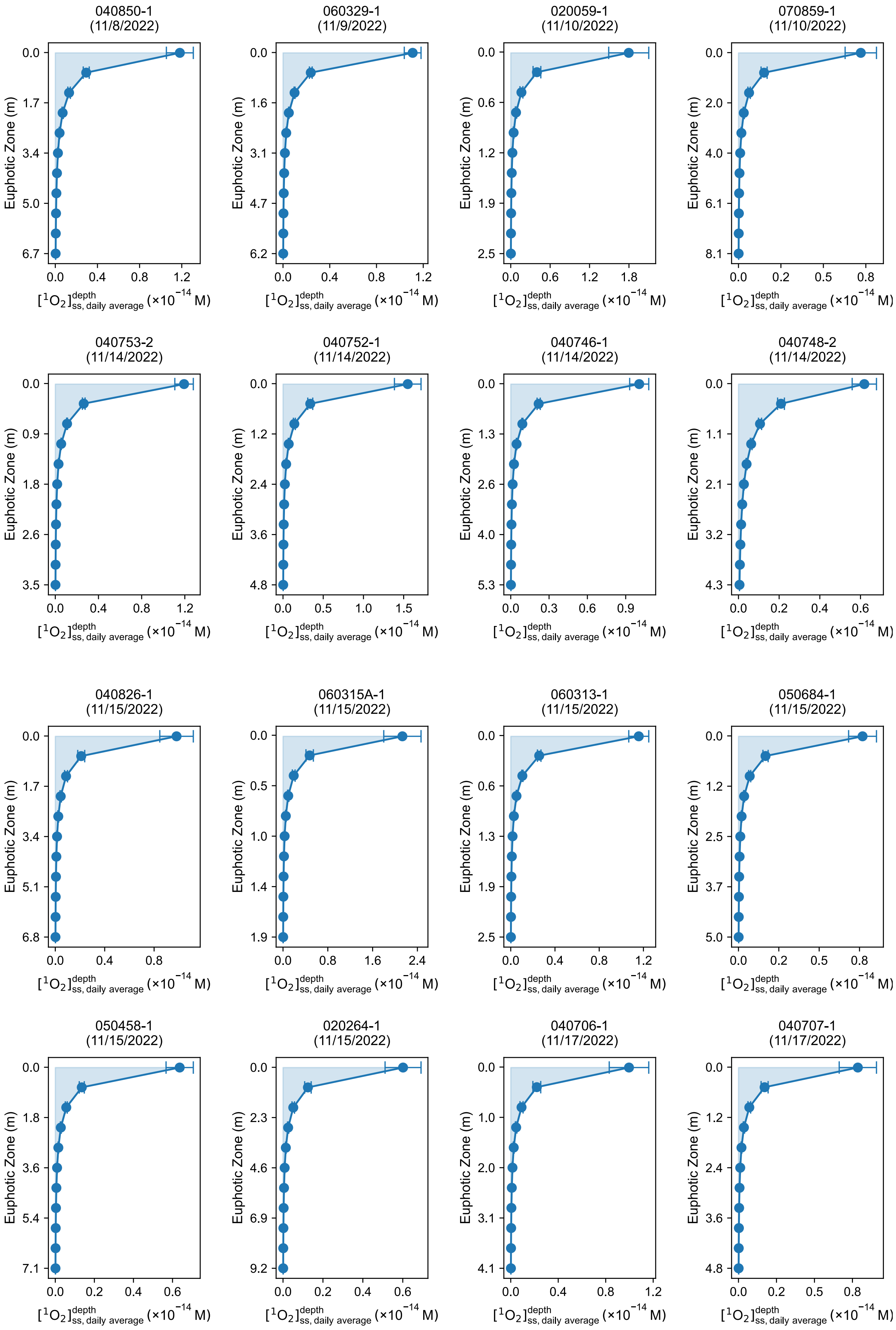

040850-1
(11/8/2022)
Euphotic Zone (m)
0.0 1.7 3.4 5.0 6.7
0.0 0.4 0.8 1.2
$[^1O_2]^{depth}_{ss,\ daily\ average}$ (×10^{-14} M)
060329-1
(11/9/2022)
Euphotic Zone (m)
0.0 1.6 3.1 4.7 6.2
0.0 0.4 0.8 1.2
$[^1O_2]^{depth}_{ss,\ daily\ average}$ (×10^{-14} M)
020059-1
(11/10/2022)
Euphotic Zone (m)
0.0 0.6 1.2 1.9 2.5
0.0 0.6 1.2 1.8
$[^1O_2]^{depth}_{ss,\ daily\ average}$ (×10^{-14} M)
070859-1
(11/10/2022)
Euphotic Zone (m)
0.0 2.0 4.0 6.1 8.1
0.0 0.2 0.5 0.8
$[^1O_2]^{depth}_{ss,\ daily\ average}$ (×10^{-14} M)
040753-2
(11/14/2022)
Euphotic Zone (m)
0.0 0.9 1.8 2.6 3.5
0.0 0.4 0.8 1.2
$[^1O_2]^{depth}_{ss,\ daily\ average}$ (×10^{-14} M)
040752-1
(11/14/2022)
Euphotic Zone (m)
0.0 1.2 2.4 3.6 4.8
0.0 0.5 1.0 1.5
$[^1O_2]^{depth}_{ss,\ daily\ average}$ (×10^{-14} M)
040746-1
(11/14/2022)
Euphotic Zone (m)
0.0 1.3 2.6 4.0 5.3
0.0 0.3 0.6 0.9
$[^1O_2]^{depth}_{ss,\ daily\ average}$ (×10^{-14} M)
040748-2
(11/14/2022)
Euphotic Zone (m)
0.0 1.1 2.1 3.2 4.3
0.0 0.2 0.4 0.6
$[^1O_2]^{depth}_{ss,\ daily\ average}$ (×10^{-14} M)
040826-1
(11/15/2022)
Euphotic Zone (m)
0.0 1.7 3.4 5.1 6.8
0.0 0.4 0.8
$[^1O_2]^{depth}_{ss,\ daily\ average}$ (×10^{-14} M)
060315A-1
(11/15/2022)
Euphotic Zone (m)
0.0 0.5 1.0 1.4 1.9
0.0 0.8 1.6 2.4
$[^1O_2]^{depth}_{ss,\ daily\ average}$ (×10^{-14} M)
060313-1
(11/15/2022)
Euphotic Zone (m)
0.0 0.6 1.3 1.9 2.5
0.0 0.4 0.8 1.2
$[^1O_2]^{depth}_{ss,\ daily\ average}$ (×10^{-14} M)
050684-1
(11/15/2022)
Euphotic Zone (m)
0.0 1.2 2.5 3.7 5.0
0.0 0.2 0.5 0.8
$[^1O_2]^{depth}_{ss,\ daily\ average}$ (×10^{-14} M)
050458-1
(11/15/2022)
Euphotic Zone (m)
0.0 1.8 3.6 5.4 7.1
0.0 0.2 0.4 0.6
$[^1O_2]^{depth}_{ss,\ daily\ average}$ (×10^{-14} M)
020264-1
(11/15/2022)
Euphotic Zone (m)
0.0 2.3 4.6 6.9 9.2
0.0 0.2 0.4 0.6
$[^1O_2]^{depth}_{ss,\ daily\ average}$ (×10^{-14} M)
040706-1
(11/17/2022)
Euphotic Zone (m)
0.0 1.0 2.0 3.1 4.1
0.0 0.4 0.8 1.2
$[^1O_2]^{depth}_{ss,\ daily\ average}$ (×10^{-14} M)
040707-1
(11/17/2022)
Euphotic Zone (m)
0.0 1.2 2.4 3.6 4.8
0.0 0.2 0.5 0.8
$[^1O_2]^{depth}_{ss,\ daily\ average}$ (×10^{-14} M)

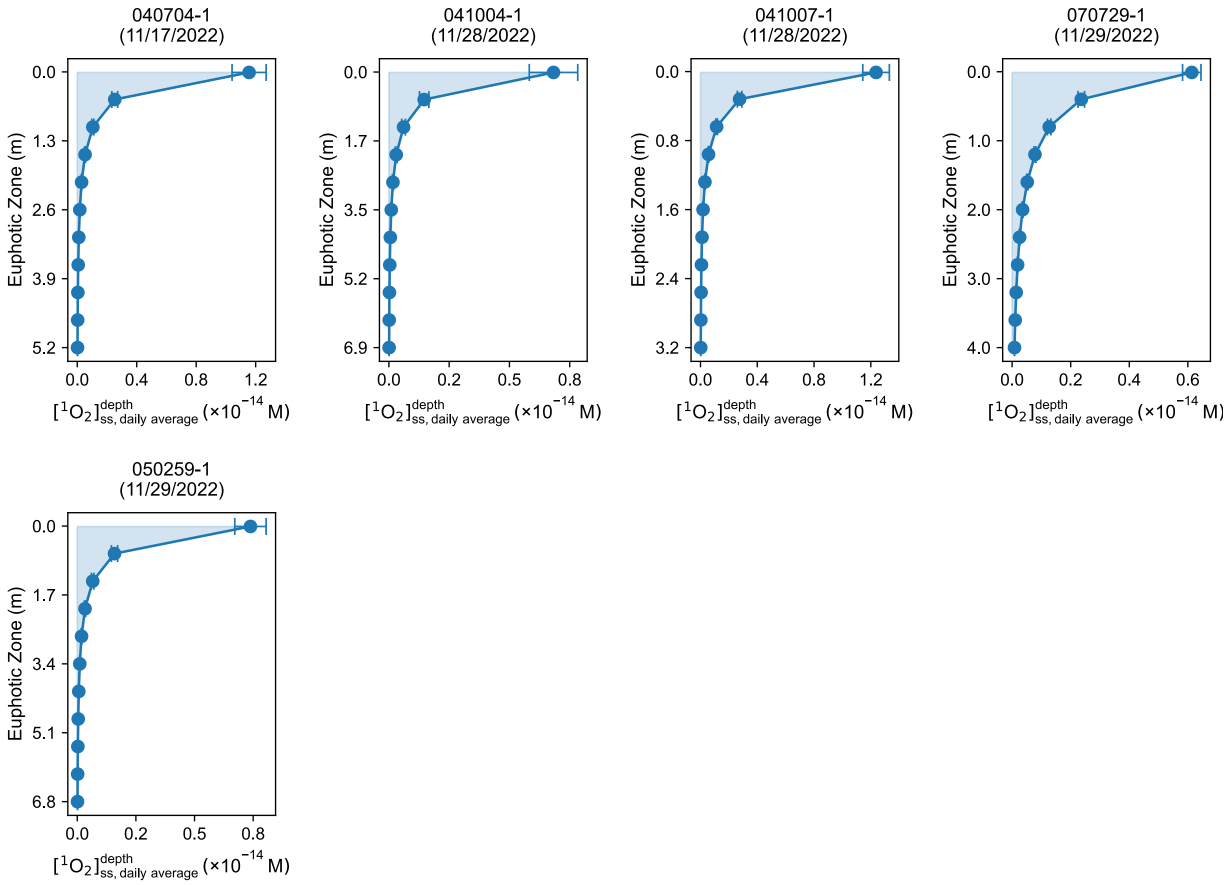

Figure S30. Daily average depth-dependent steady-state concentration profiles of $^1O_2$ in the euphotic zone of ALTM lakes during the fall-winter season. Error bars indicate the standard deviation of duplicate estimates for $[^1O_2]_{ss,\ daily\ average}^{depth}$.

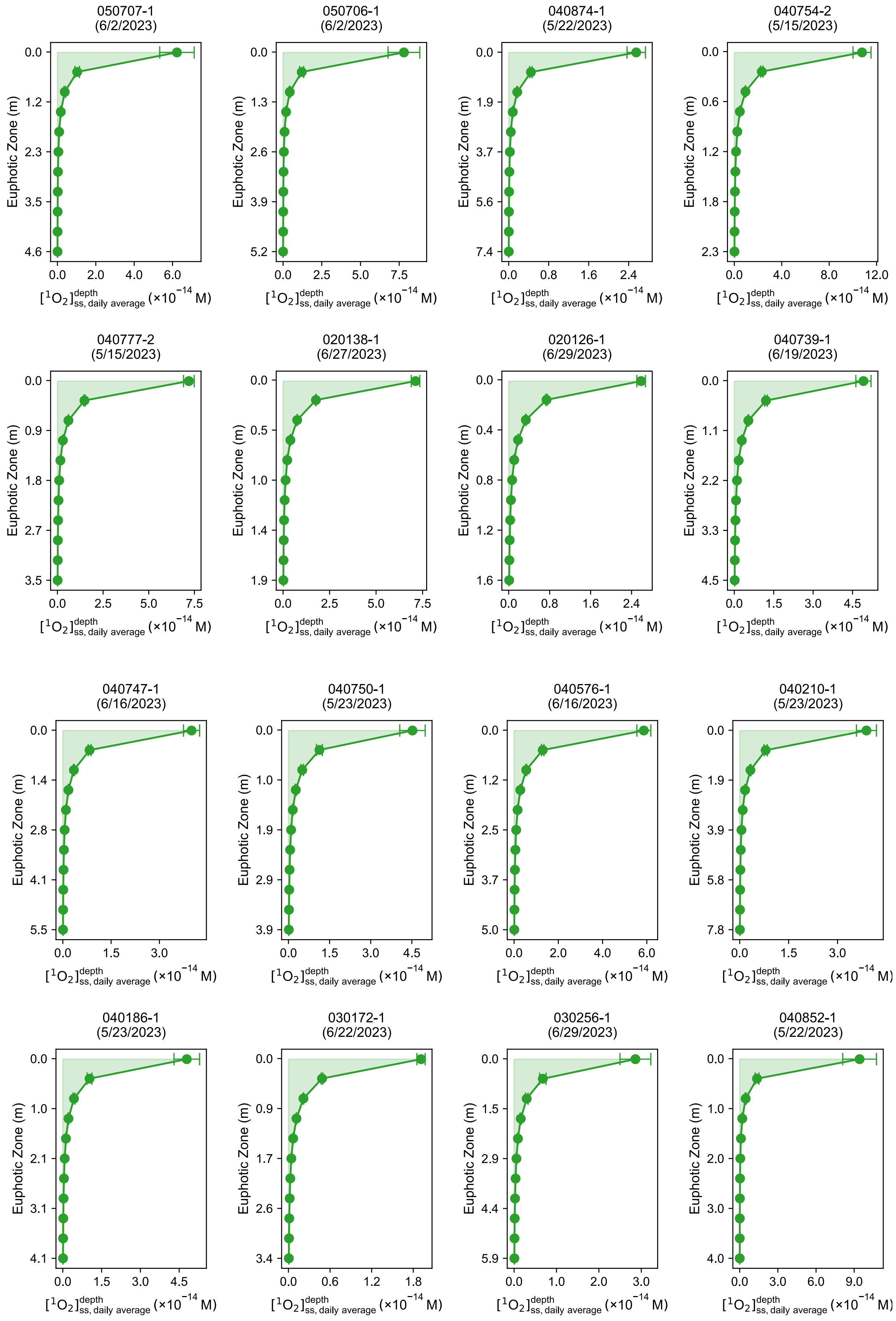
050707-1
(6/2/2023)
050706-1
(6/2/2023)
040874-1
(5/22/2023)
040754-2
(5/15/2023)
040777-2
(5/15/2023)
020138-1
(6/27/2023)
020126-1
(6/29/2023)
040739-1
(6/19/2023)
040747-1
(6/16/2023)
040750-1
(5/23/2023)
040576-1
(6/16/2023)
040210-1
(5/23/2023)
040186-1
(5/23/2023)
030172-1
(6/22/2023)
030256-1
(6/29/2023)
040852-1
(5/22/2023)
Euphotic Zone (m)
$[^1O_2]^{depth}_{ss,\ daily\ average}$ (×10^{-14} M)
0.0
1.2
2.3
3.5
4.6
1.3
2.6
3.9
5.2
1.9
3.7
5.6
7.4
0.6
1.8
0.9
2.7
0.5
1.0
1.4
0.4
0.8
1.6
1.1
2.2
3.3
4.5
2.8
4.1
5.5
2.9
2.5
5.0
5.8
7.8
2.1
3.1
1.7
3.4
4.4
5.9
2.0
3.0
4.0
6.0
7.5
12.0
8.0
1.5
0.6
9.0

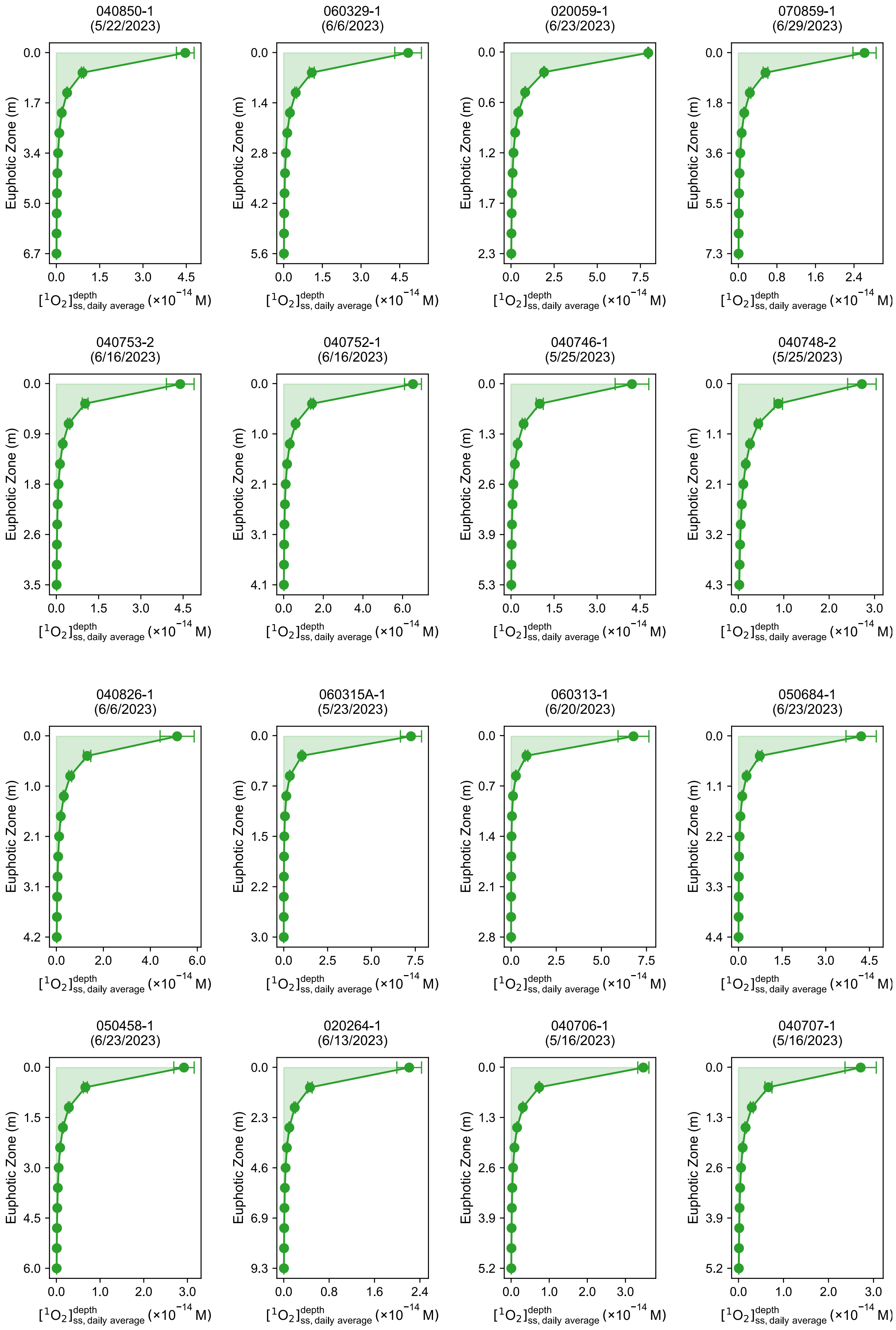
040850-1 (5/22/2023)
060329-1 (6/6/2023)
020059-1 (6/23/2023)
070859-1 (6/29/2023)
040753-2 (6/16/2023)
040752-1 (6/16/2023)
040746-1 (5/25/2023)
040748-2 (5/25/2023)
040826-1 (6/6/2023)
060315A-1 (5/23/2023)
060313-1 (6/20/2023)
050684-1 (6/23/2023)
050458-1 (6/23/2023)
020264-1 (6/13/2023)
040706-1 (5/16/2023)
040707-1 (5/16/2023)
Euphotic Zone (m)
$[^1O_2]^{depth}_{ss, daily average}$ ($\times 10^{-14}$ M)

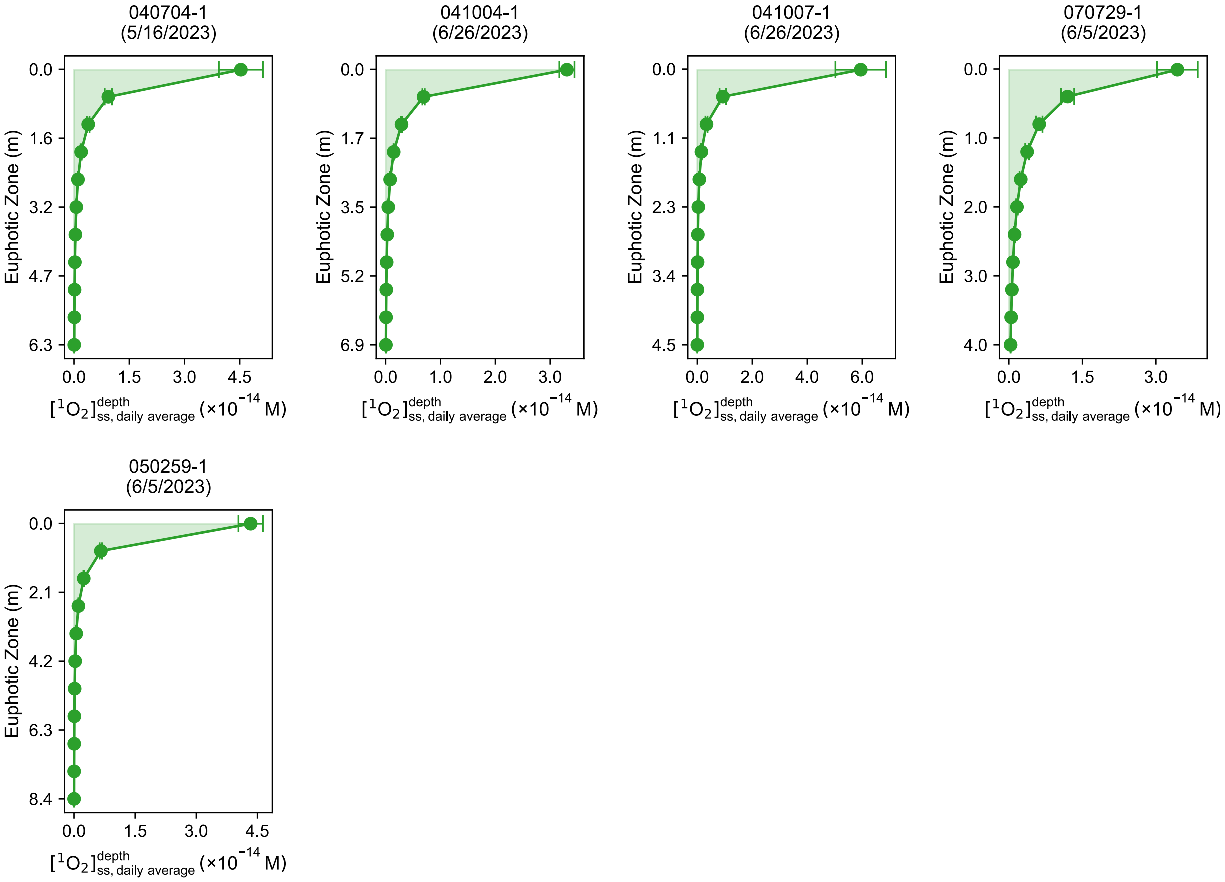

Figure S31. Daily average depth-dependent steady-state concentration profiles of $^1O_2$ in the euphotic zone of ALTM lakes during the spring-summer season. Error bars indicate the standard deviation of duplicate estimates for $[^1O_2]^{depth}_{ss,\ daily\ average}$.

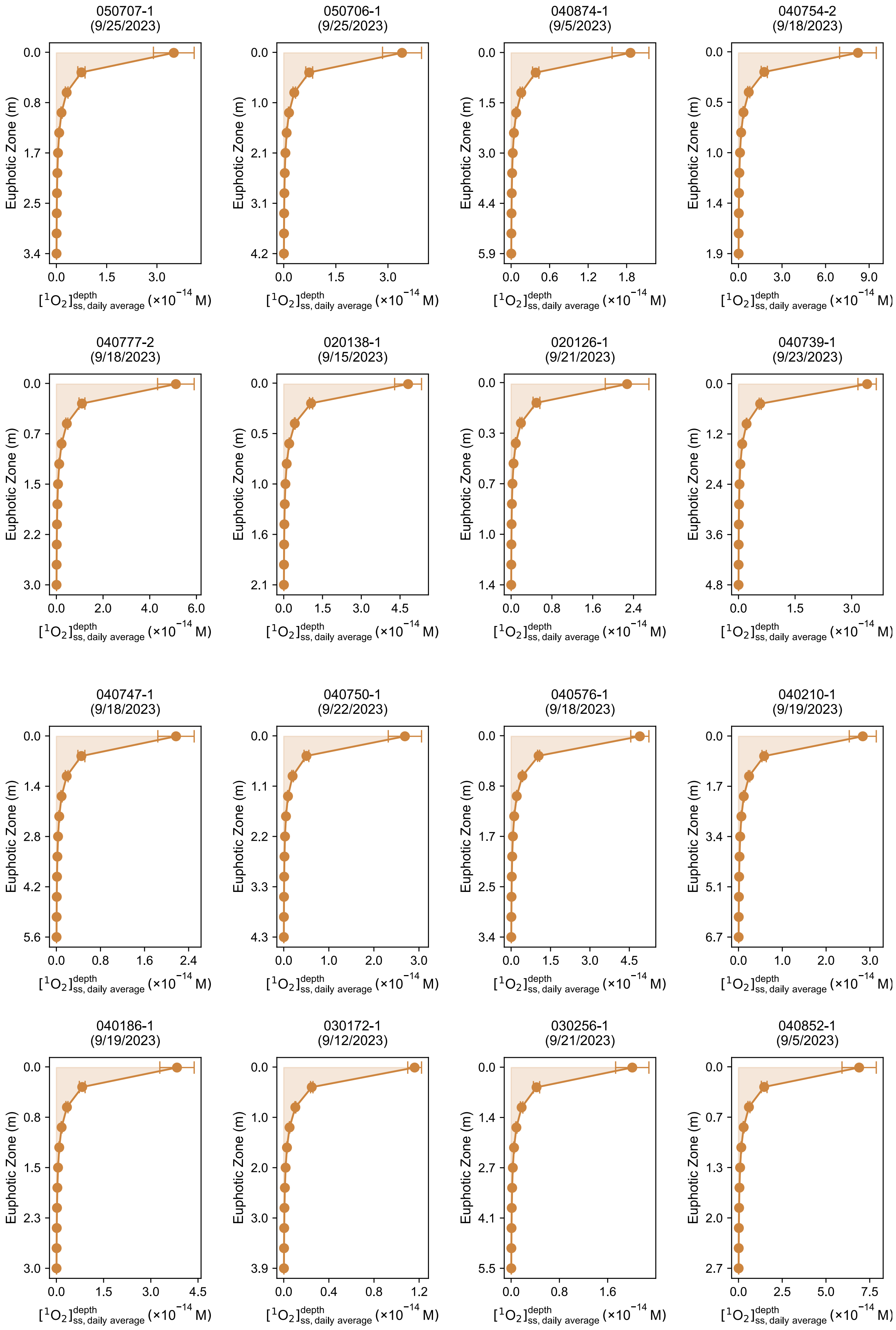
050707-1
(9/25/2023)
050706-1
(9/25/2023)
040874-1
(9/5/2023)
040754-2
(9/18/2023)
040777-2
(9/18/2023)
020138-1
(9/15/2023)
020126-1
(9/21/2023)
040739-1
(9/23/2023)
040747-1
(9/18/2023)
040750-1
(9/22/2023)
040576-1
(9/18/2023)
040210-1
(9/19/2023)
040186-1
(9/19/2023)
030172-1
(9/12/2023)
030256-1
(9/21/2023)
040852-1
(9/5/2023)
Euphotic Zone (m)
$[^1O_2]^{depth}_{ss,\ daily\ average}$ ($\times10^{-14}$ M)

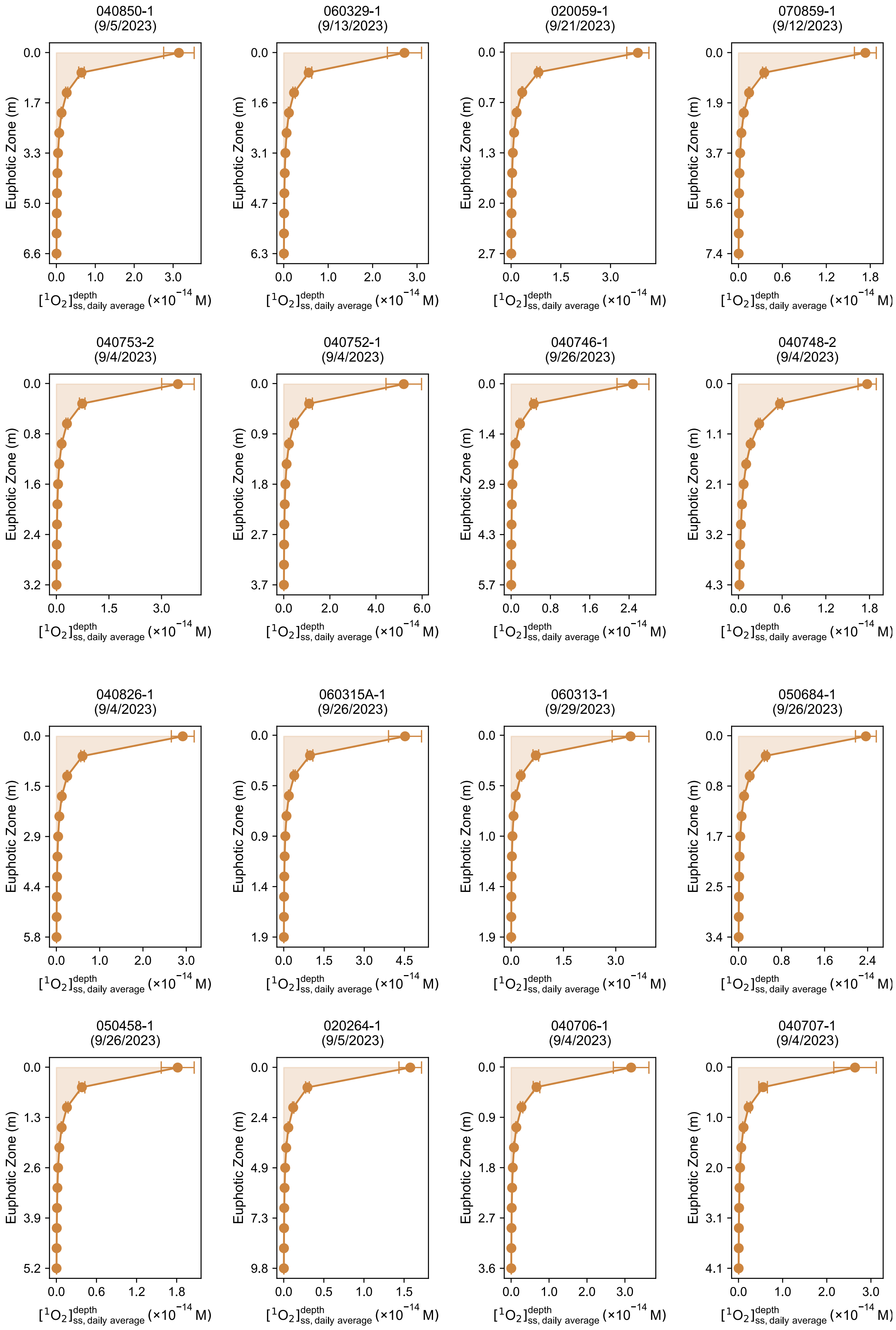

040850-1
(9/5/2023)
0.0 1.7 3.3 5.0 6.6
0.0 1.0 2.0 3.0
060329-1
(9/13/2023)
0.0 1.6 3.1 4.7 6.3
0.0 1.0 2.0 3.0
020059-1
(9/21/2023)
0.0 0.7 1.3 2.0 2.7
0.0 1.5 3.0
070859-1
(9/12/2023)
0.0 1.9 3.7 5.6 7.4
0.0 0.6 1.2 1.8
040753-2
(9/4/2023)
0.0 0.8 1.6 2.4 3.2
0.0 1.5 3.0
040752-1
(9/4/2023)
0.0 0.9 1.8 2.7 3.7
0.0 2.0 4.0 6.0
040746-1
(9/26/2023)
0.0 1.4 2.9 4.3 5.7
0.0 0.8 1.6 2.4
040748-2
(9/4/2023)
0.0 1.1 2.1 3.2 4.3
0.0 0.6 1.2 1.8
040826-1
(9/4/2023)
0.0 1.5 2.9 4.4 5.8
0.0 1.0 2.0 3.0
060315A-1
(9/26/2023)
0.0 0.5 0.9 1.4 1.9
0.0 1.5 3.0 4.5
060313-1
(9/29/2023)
0.0 0.5 1.0 1.4 1.9
0.0 1.5 3.0
050684-1
(9/26/2023)
0.0 0.8 1.7 2.5 3.4
0.0 0.8 1.6 2.4
050458-1
(9/26/2023)
0.0 1.3 2.6 3.9 5.2
0.0 0.6 1.2 1.8
020264-1
(9/5/2023)
0.0 2.4 4.9 7.3 9.8
0.0 0.5 1.0 1.5
040706-1
(9/4/2023)
0.0 0.9 1.8 2.7 3.6
0.0 1.0 2.0 3.0
040707-1
(9/4/2023)
0.0 1.0 2.0 3.1 4.1
0.0 1.0 2.0 3.0
Euphotic Zone (m)
$[^1O_2]^{depth}_{ss,\ daily\ average}$ ($\times 10^{-14}$ M)

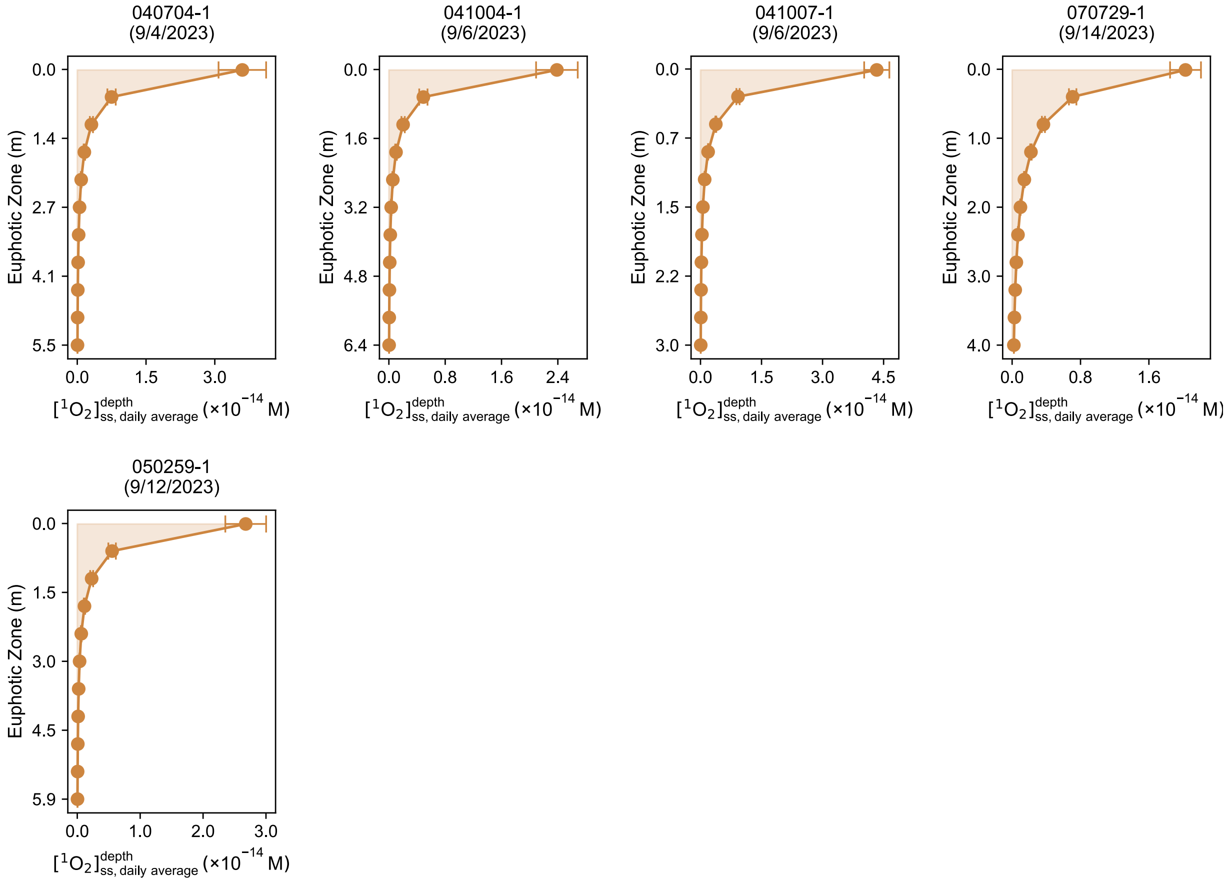

Figure S32. Daily average depth-dependent steady-state concentration profiles of $^1O_2$ in the euphotic zone of ALTM lakes during the summer-fall season. Error bars indicate the standard deviation of duplicate estimates for $[^1O_2]^{depth}_{ss,\ daily\ average}$.

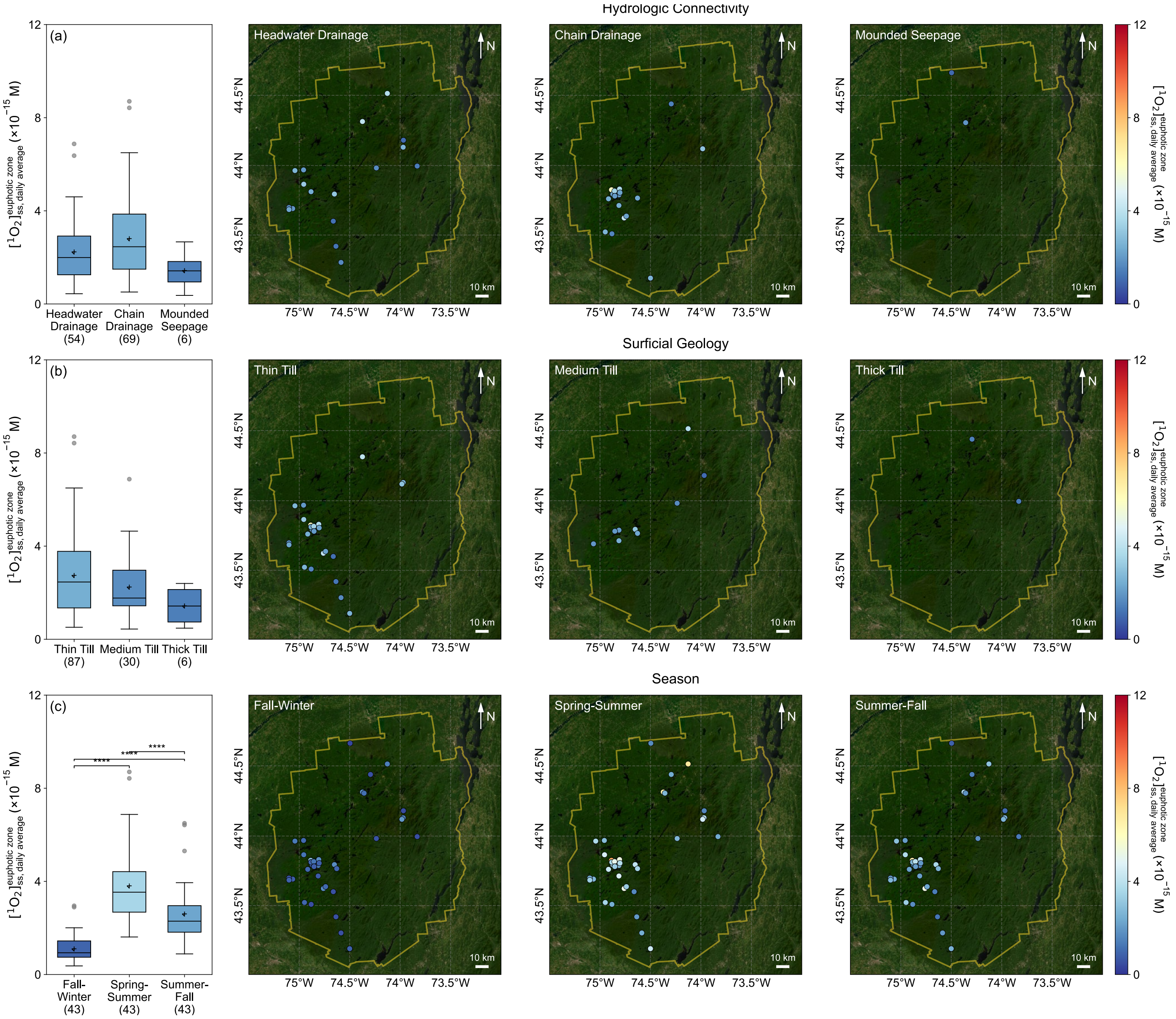

Figure S33. Spatiotemporal patterns of $[^1O_2]_{ss,\ daily\ average}^{euphotic\ zone}$ in ALTM lakes grouped by **(a)** hydrologic connectivity (headwater drainage, chain drainage, or mounded seepage), **(b)** surficial geology (thin till, medium till, or thick till), and **(c)** season (fall-winter, spring-summer, or summer-fall). On panels (a) to (c), each box spans the 25th to 75th percentiles, with whiskers extending to 1.5 times the interquartile range below the 25th and above the 75th percentiles. The centerline and "+" sign mark the median and mean, respectively. Gray circles represent outliers. Numbers in parentheses represent the number of samples in each group. Box colors correspond to their respective median values referenced against the color bar. For multiple comparisons, a Kruskal-Wallis test was first performed to determine whether statistically significant differences existed among groups. If significant, pairwise Mann-Whitney *U* tests were performed, with significant differences marked by asterisks as "*" (p<0.05), "**" (p<0.01), "***" (p<0.001), or "****" (p<0.0001). On each map, the solid yellow line delineates the boundary of Adirondack Park.

16. Comparisons of steady-state concentrations of $^1O_2$ and $\Phi_{app, ^1O_2}$ by lake browning status

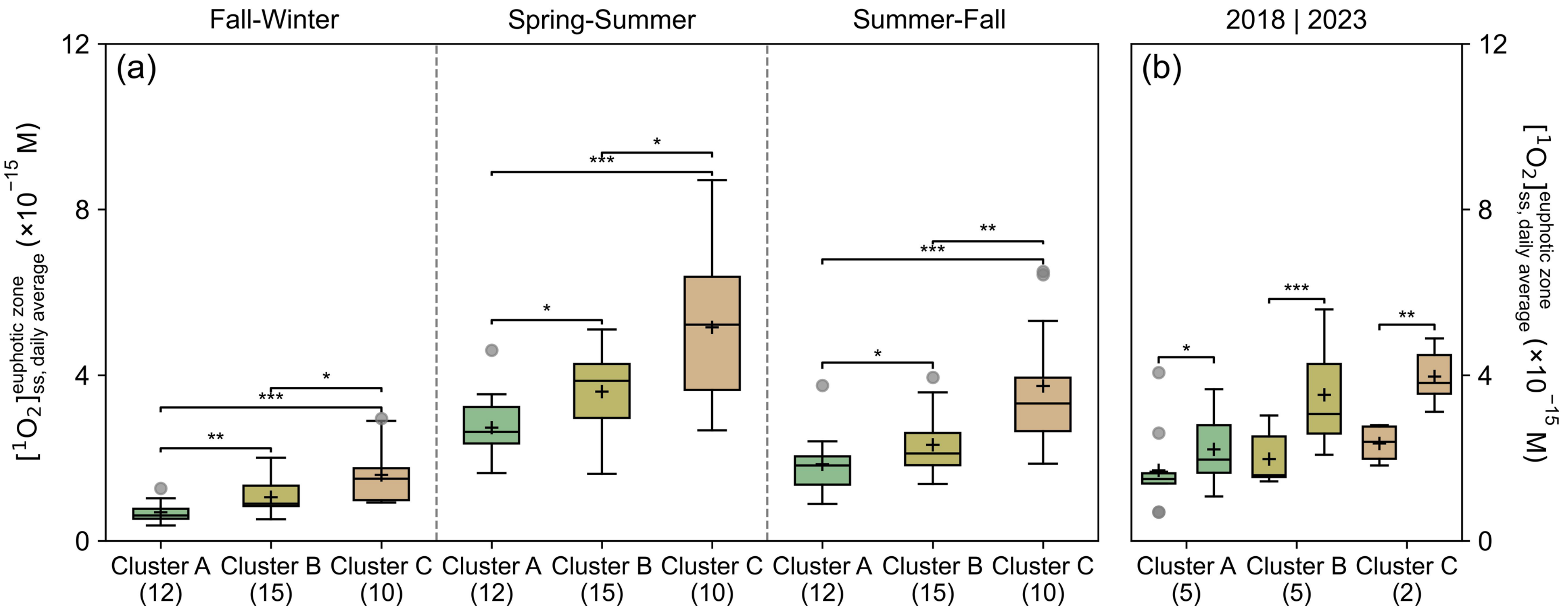

Figure S34. Multiple comparisons of $[^1O_2]_{\text{ss, daily average}}^{\text{euphotic zone}}$ across ALTM lake clusters by **(a)** season and **(b)** sampling year. "2018" represents data from a previous study for 12 ALTM lakes[29] that were also sampled in this work, while "2023" represents data for the same lakes measured in this work. Lakes in clusters A, B, and C were operationally designated as experiencing mild, moderate, and intense browning, respectively. Each box spans the 25th to 75th percentiles, with whiskers extending to 1.5 times the interquartile range below the 25th and above the 75th percentiles. The centerline and "+" sign mark the median and mean, respectively. Gray circles represent outliers. Numbers in parentheses represent the number of lakes in each cluster. For multiple comparisons, a Kruskal-Wallis test was first performed to determine whether statistically significant differences existed among groups. If significant, pairwise Mann-Whitney *U* tests were performed, with significant differences marked by asterisks as "*" (p<0.05), "**" (p<0.01), "***" (p<0.001), or "****" (p<0.0001).

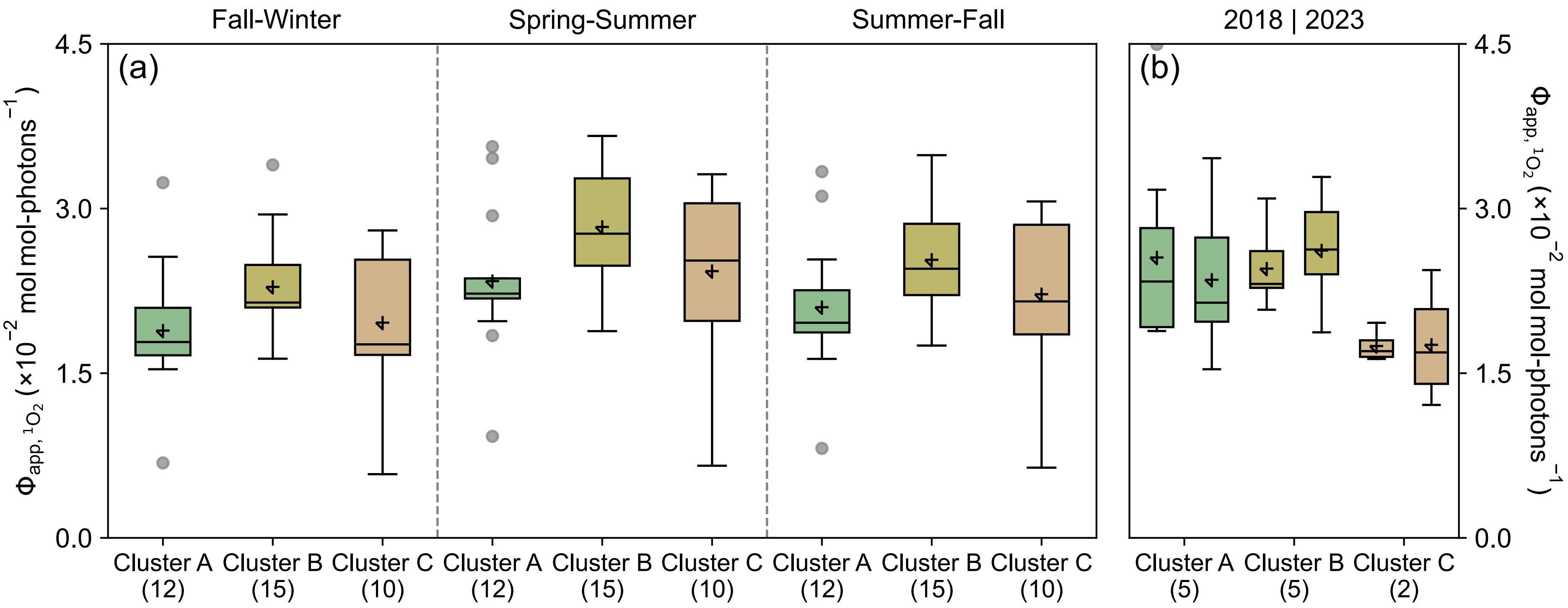

Figure S35. Multiple comparisons of $\Phi_{app, ^1O_2}$ across ALTM lake clusters by **(a)** season and **(b)** sampling year. "2018" represents data from a previous study for 12 ALTM lakes[29] that were also sampled in this work, while "2023" represents data for the same lakes measured in this work. Lakes in clusters A, B, and C were operationally designated as experiencing mild, moderate, and intense browning, respectively. Each box spans the 25th to 75th percentiles, with whiskers extending to 1.5 times the interquartile range below the 25th and above the 75th percentiles. The centerline and "+" sign mark the median and mean, respectively. Gray circles represent outliers. Numbers in parentheses represent the number of lakes in each cluster. For multiple comparisons, a Kruskal-Wallis test was first performed to determine whether statistically significant differences existed among groups. If significant, pairwise Mann-Whitney *U* tests were performed, with significant differences marked by asterisks as "*" ($p<0.05$), "**" ($p<0.01$), "***" ($p<0.001$), or "****" ($p<0.0001$).

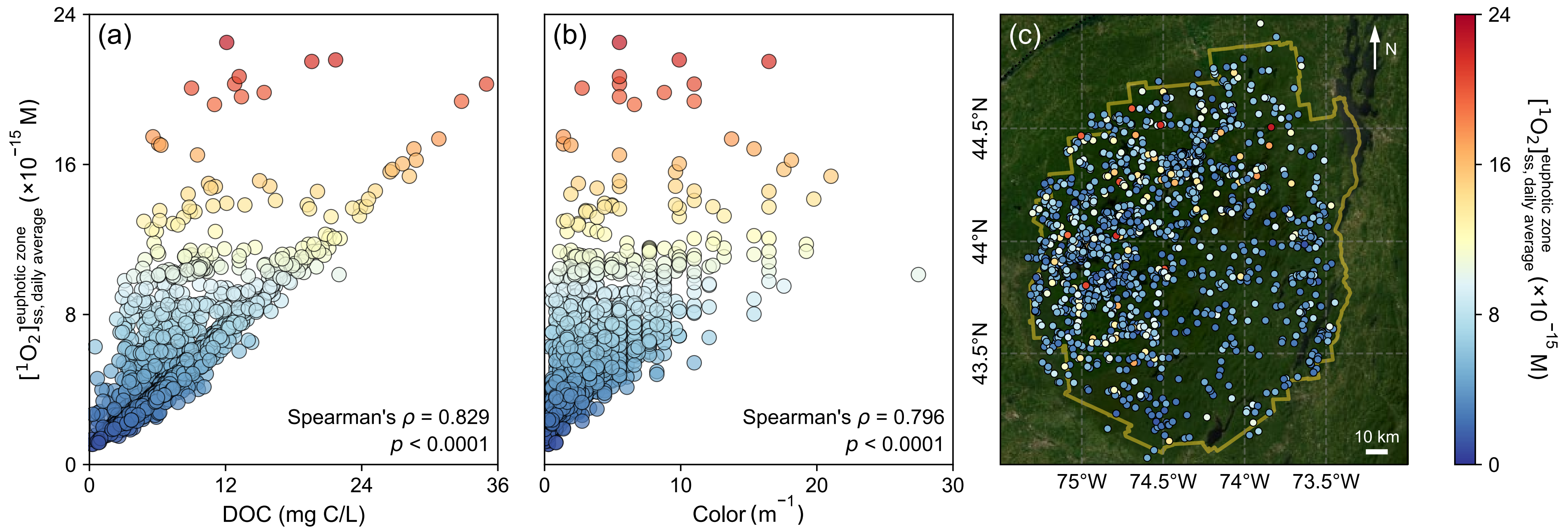

Figure S36. $[^1O_2]^{\text{euphotic zone}}_{\text{ss, daily average}}$ estimated for 1,469 Adirondack lakes surveyed between 1984 and 1987[59, 60]: **(a)** Spearman's correlation between $[^1O_2]^{\text{euphotic zone}}_{\text{ss, daily average}}$ and DOC in Adirondack lakes with historical surface water chemistry data. Contemporary DOC in these lakes was estimated using historical values and the regression slope between historical and contemporary data from ALTM lakes when applicable. **(b)** Spearman's correlation between $[^1O_2]^{\text{euphotic zone}}_{\text{ss, daily average}}$ and color in Adirondack lakes. Contemporary color in these lakes was estimated using historical values and the average Sen's slope for color from ALTM lakes. **(c)** Map of $[^1O_2]^{\text{euphotic zone}}_{\text{ss, daily average}}$ in Adirondack lakes (n=1,469) estimated based on the average $\Phi_{\text{app}, {}^1O_2}$ for ALTM lakes sampled in this work and contemporary site-specific daily average solar irradiance following Equation S8. On panels (a) and (b), marker colors correspond to $[^1O_2]^{\text{euphotic zone}}_{\text{ss, daily average}}$ referenced against the color bar. For panel (c), the solid yellow line delineates the boundary of Adirondack Park.

17. Calculations of contaminant half-lives mediated by $^1O_2$ reactions

To estimate the half-life of a contaminant in the euphotic zone of an ALTM lake due to reaction with $^1O_2$, Equation S13 was used:[40]

$$t_{1/2,\ \mathrm{rxn},\ ^1O_2}^{\mathrm{euphotic\ zone}} = \frac{\ln(2)}{k_{\mathrm{rxn},\ ^1O_2} \times [^1O_2]_{\mathrm{ss,\ daily\ average}}^{\mathrm{euphotic\ zone}}} \tag{S13}$$

where $k_{\mathrm{rxn},\ ^1O_2}$ ($M^{-1}\ s^{-1}$) is the bimolecular reaction rate constant of the compound reacting with $^1O_2$ (**Table S16**).

**Table S16.** Summary of $k_{rxn, {}^1O_2}$ for selected contaminants			
Compound	$k_{rxn, {}^1O_2}$ (M^{-1} s^{-1})[a]	Occurrence in Adirondack Lakes	Category
2-Mercaptobenzothiazole	$3.90(\pm0.80)\times10^7$		Personal Care, Household & Industrial Chemical (Vulcanization Accelerator)
4-Hydroxybenzotriazole	$1.31(\pm0.04)\times10^8$		Personal Care, Household & Industrial Chemical TP (1H-Benzotriazole)
Acetaminophen	3.35×10^5		Pharmaceutical (Analgesic, Antipyretic)
Acetochlor	$5.30(\pm0.10)\times10^5$		Pesticide (Herbicide)
Acyclovir	$4.36(\pm0.32)\times10^6$		Pharmaceutical (Antiviral, DNA polymerase inhibitor)
Alachlor	$3.30(\pm0.10)\times10^5$		Pesticide (Herbicide)
Amikacin	$8.10(\pm0.62)\times10^5$		Pharmaceutical (Antibacterial, Protein biosynthesis inhibitor)
Amoxicillin	1.44×10^4		Pharmaceutical (Antibacterial, Cell wall biosynthesis inhibitor)
Atenolol	$8.47(\pm0.56)\times10^3$		Pharmaceutical (Antiarrhythmic, Antihypertensive, Vasodilator, beta1-Adrenergic receptor antagonist)
Atorvastatin	$3.10(\pm0.20)\times10^8$		Pharmaceutical (Antihyperlipidemic, HMG-CoA reductase inhibitor)
Atrazine	$2.00(\pm0.30)\times10^5$	Median: 21 ng/L[61]	Pesticide (Herbicide)
Azithromycin	$2.42(\pm0.09)\times10^5$		Pharmaceutical (Antibacterial, Protein biosynthesis inhibitor)
Bacitracin A	$6.15(\pm0.42)\times10^7$		Pharmaceutical (Antibacterial, Cell membrane function inhibitor)
Bentazon	$3.10(\pm0.30)\times10^7$		Pesticide (Herbicide)
Benzophenone-1 (2,4-Dihydroxybenzophenone)	9.20×10^6		Personal Care, Household & Industrial Chemical (Sunscreen Agent)
Benzophenone-3 (Oxybenzone)	$1.99(\pm0.08)\times10^5$	Median: 23 ng/L[61]	Personal Care, Household & Industrial Chemical (Sunscreen Agent)
Biochanin A	$1.70(\pm0.20)\times10^7$		Phytoestrogen
Bupropion	$7.70(\pm0.93)\times10^3$		Pharmaceutical (Antidepressant, Noradrenalin and dopamine reuptake inhibitor)
Carbamazepine	$1.88(\pm0.09)\times10^5$		Pharmaceutical (Analgesic, Antiepileptic)
Chlorpyrifos	$1.67(\pm0.28)\times10^6$ [b]		Pesticide (Insecticide, Nematicide)
Cimetidine	$9.20(\pm0.60)\times10^7$		Pharmaceutical (Anti-ulcerative, H2 receptor antagonist)
Ciprofloxacin	1.18×10^7		Pharmaceutical (Antibacterial, Nucleic acid biosynthesis inhibitor)
Clarithromycin	$1.22(\pm0.03)\times10^5$		Pharmaceutical (Antibacterial, Protein biosynthesis inhibitor)
Clofibric Acid	$6.00(\pm1.90)\times10^5$		Pesticide (Herbicide) / Pharmaceutical TP (Clofibrate)
Clopyralid	$7.00(\pm0.90)\times10^5$		Pesticide (Herbicide)
Cyanazine	$1.70(\pm0.20)\times10^5$		Pesticide (Herbicide)
Cyprodinil	$1.38(\pm0.08)\times10^6$		Pesticide (Fungicide)
Danofloxacin	$7.50(\pm0.13)\times10^4$		Pharmaceutical (Antibacterial (veterinary), Nucleic acid biosynthesis inhibitor)
Daptomycin	$1.30(\pm0.20)\times10^7$		Pharmaceutical (Antibacterial, Cell membrane function inhibitor)
Dexamethasone	$1.20(\pm0.09)\times10^6$		Pharmaceutical (Anti-inflammatory, Antipruritic, Glucocorticoid receptor agonist)
Diazinon	$7.29(\pm0.30)\times10^4$		Pesticide (Insecticide)
Dicamba	$7.55(\pm0.61)\times10^5$		Pesticide (Herbicide)
Diclofenac	$1.30(\pm0.20)\times10^7$		Pharmaceutical (Analgesic, Anti-inflammatory, Antipyretic, COX inhibitor)
Dimethomorph	$8.50(\pm0.28)\times10^5$		Pesticide (Fungicide)
Diuron	$2.90(\pm0.60)\times10^5$		Pesticide (Herbicide)
Drometrizole	$8.65(\pm0.40)\times10^7$		Personal Care, Household & Industrial Chemical (UV Stabilizer)
Duloxetine	$4.89(\pm0.45)\times10^4$		Pharmaceutical (Antidepressant, Serotonin-noradrenaline reuptake inhibitor (SNRI))
Equol	$1.70(\pm0.20)\times10^7$		Phytoestrogen
Erythromycin	$1.23(\pm0.04)\times10^5$		Pharmaceutical (Antibacterial, Protein biosynthesis inhibitor)
Estradiol (17β-Estradiol)	1.39×10^7		Pharmaceutical (Menstruation disorder agent, Estrogen receptor agonist)
Fludioxonil	$4.90(\pm0.20)\times10^7$		Pesticide (Fungicide)
Flufenamic Acid	$1.30(\pm0.20)\times10^7$		Pharmaceutical (Anti-inflammatory, COX inhibitor)
Fluoxetine	$1.44(\pm0.14)\times10^8$	Median: 6 ng/L[61]	Pharmaceutical (Antidepressant, Selective serotonin reuptake inhibitor (SSRI))

**Table S16.** Summary of $k_{rxn, {}^1O_2}$ for selected contaminants (continued)			
Compound	$k_{rxn, {}^1O_2}$ (M^{-1} s^{-1})[a]	Occurrence in Adirondack Lakes	Category
Fluroxypyr	$3.59(\pm0.23)\times10^5$		Pesticide (Herbicide)
Fluvastatin	$1.64(\pm0.18)\times10^8$		Pharmaceutical (Antihyperlipidemic, HMG-CoA reductase inhibitor)
Fomesafen	$4.35(\pm0.26)\times10^5$ [b]		Pesticide (Herbicide)
Furaltadone	$3.30(\pm0.20)\times10^6$		Pharmaceutical (Anti-infective (urinary))
Furazolidone	$5.00(\pm2.00)\times10^5$		Pharmaceutical (Antibacterial, Antiprotozoal)
Genistein	$3.10(\pm0.50)\times10^7$		Phytoestrogen
Gentamicin	$2.65(\pm0.26)\times10^5$		Pharmaceutical (Antibacterial, Protein biosynthesis inhibitor)
Ibuprofen	$6.00(\pm0.58)\times10^4$		Pharmaceutical (Analgesic, Anti-inflammatory, Antipyretic, COX inhibitor)
Isoproturon	$1.00(\pm0.10)\times10^6$		Pesticide (Herbicide)
Kanamycin	$6.84(\pm0.51)\times10^5$		Pharmaceutical (Antibacterial, Protein biosynthesis inhibitor)
Lamivudine	$5.20(\pm0.42)\times10^5$		Pharmaceutical (Antiviral, Reverse transcriptase inhibitor)
MCPA ((4-Chloro-2-methylphenoxy)acetic Acid)	$8.52(\pm0.51)\times10^5$		Pesticide (Herbicide)
Meclofenamic Acid	$2.80(\pm0.60)\times10^7$	Median: 105 ng/L[61]	Pharmaceutical (Anti-inflammatory, COX inhibitor)
Mefenamic Acid	$1.60(\pm0.70)\times10^7$		Pharmaceutical (Analgesic, Anti-inflammatory, Antipyretic, COX inhibitor)
Mesotrione	$6.51(\pm1.13)\times10^5$ [b]		Pesticide (Herbicide)
Methylmercury	1.90×10^6	Dittman and Driscoll[62]	Neurotoxin
Metolachlor	$4.40(\pm0.60)\times10^5$	Median: 19 ng/L[61]	Pesticide (Herbicide)
Metoprolol	$6.27(\pm0.25)\times10^3$		Pharmaceutical (Antihypertensive, Vasodilator, beta1-Adrenergic receptor antagonist)
Metoxuron	$1.10(\pm0.10)\times10^6$		Pesticide (Herbicide)
Metronidazole	2.79×10^5		Pharmaceutical (Antibacterial, Antiprotozoal, DNA synthesis inhibitor)
Microcystin-LR	6.30×10^5	Gorney and Smith[63]	Cyanotoxin
Nadolol	$9.18(\pm0.37)\times10^3$		Pharmaceutical (Antiarrhythmic, Antihypertensive, Vasodilator, beta-Adrenergic receptor antagonist)
Niclosamide	$2.25(\pm0.50)\times10^7$		Pesticide (Molluscicide)
Nitrofurantoin	$3.00(\pm2.00)\times10^5$		Pharmaceutical (Antibacterial)
Norfloxacin	4.30×10^5		Pharmaceutical (Antibacterial, Nucleic acid biosynthesis inhibitor)
Octinoxate (Ethylhexyl Methoxycinnamate)	$1.54(\pm0.88)\times10^7$		Personal Care, Household & Industrial Chemical (Sunscreen Agent)
Ornidazole	5.51×10^5		Pharmaceutical (Antibacterial, Antiprotozoal)
Paroxetine	$1.18(\pm0.13)\times10^8$		Pharmaceutical (Antidepressant, Selective serotonin reuptake inhibitor (SSRI))
Penciclovir	$5.56(\pm0.02)\times10^6$		Pharmaceutical (Antiviral, DNA polymerase inhibitor)
Pindolol	6.40×10^7		Pharmaceutical (Antiarrhythmic, Antihypertensive, Vasodilator, beta-Adrenergic receptor antagonist)
Polymyxin B1	$1.02(\pm0.25)\times10^8$		Pharmaceutical (Antibacterial, Cell membrane function inhibitor)
Polymyxin B2	$1.02(\pm0.25)\times10^8$		Pharmaceutical (Antibacterial, Cell membrane function inhibitor)
Polymyxin E1	$1.05(\pm0.18)\times10^8$		Pharmaceutical (Antibacterial, Cell membrane function inhibitor)
Polymyxin E2	$1.47(\pm0.21)\times10^8$		Pharmaceutical (Antibacterial, Cell membrane function inhibitor)
Primidone	$3.18(\pm0.44)\times10^6$		Pharmaceutical (Antiepileptic)
Prometryn	$8.40(\pm1.40)\times10^5$		Pesticide (Herbicide)
Propanil	$7.10(\pm1.80)\times10^4$		Pesticide (Herbicide)
Propiconazole	$3.90(\pm0.30)\times10^6$		Pesticide (Fungicide)
Propranolol	$9.30(\pm0.40)\times10^6$		Pharmaceutical (Antiarrhythmic, Antihypertensive, beta-Adrenergic receptor antagonist)
Prothioconazole	$5.86(\pm0.27)\times10^6$		Pesticide (Fungicide)
Pyrimethanil	$7.61(\pm0.51)\times10^5$		Pesticide (Fungicide)
Ranitidine	$2.10(\pm0.20)\times10^7$		Pharmaceutical (Anti-ulcerative, H2 receptor antagonist)
Roxithromycin	$1.41(\pm0.03)\times10^5$		Pharmaceutical (Antibacterial, Protein biosynthesis inhibitor)

**Table S16.** Summary of $k_{rxn, {}^1O_2}$ for selected contaminants (continued)			
Compound	$k_{rxn, {}^1O_2}$ (M^{-1} s^{-1})[a]	Occurrence in Adirondack Lakes	Category
Streptomycin	$1.58(\pm0.09)\times10^5$		Pharmaceutical (Antibacterial (tuberculostatic), Protein biosynthesis inhibitor)
Sulfachlorpyridazine	$1.34(\pm0.15)\times10^9$		Pharmaceutical (Antibacterial (veterinary))
Sulfadiazine	$2.02(\pm0.14)\times10^8$		Pharmaceutical (Antibacterial, Folic acid biosynthesis inhibitor)
Sulfadimethoxine	$4.83(\pm0.13)\times10^7$		Pharmaceutical (Antibacterial, Folic acid biosynthesis inhibitor)
Sulfamerazine	$1.81(\pm0.18)\times10^8$		Pharmaceutical (Antibacterial, Folic acid biosynthesis inhibitor)
Sulfamethazine	$3.39(\pm0.17)\times10^8$		Pharmaceutical (Antibacterial, Folic acid biosynthesis inhibitor)
Sulfamethizole	$3.87(\pm0.05)\times10^7$		Pharmaceutical (Antibacterial, Folic acid biosynthesis inhibitor)
Sulfamethoxazole	$2.31(\pm0.06)\times10^7$		Pharmaceutical (Antibacterial, Folic acid biosynthesis inhibitor)
Sulfathiazole	$1.84(\pm0.02)\times10^8$		Pharmaceutical (Antibacterial, Folic acid biosynthesis inhibitor)
Sulfisoxazole	$1.09(\pm0.03)\times10^9$		Pharmaceutical (Antibacterial, Folic acid biosynthesis inhibitor)
Terbutaline	$7.10(\pm0.30)\times10^6$		Pharmaceutical (Bronchodilator, beta2-Adrenergic receptor agonist)
Tetrabromobisphenol A	3.90×10^8		Personal Care, Household & Industrial Chemical (Flame retardant)
Thiobencarb	$8.83(\pm0.61)\times10^5$		Pesticide (Herbicide)
Tinidazole	3.16×10^6		Pharmaceutical (Antiprotozoal)
Tobramycin	$1.35(\pm0.23)\times10^5$		Pharmaceutical (Antibacterial, Protein biosynthesis inhibitor)
Tolfenamic Acid	$1.30(\pm0.60)\times10^7$		Pharmaceutical (Anti-inflammatory, COX inhibitor)
Triclosan	$3.00(\pm0.40)\times10^6$	Median: 46 ng/L[61]	Personal Care, Household & Industrial Chemical (Antimicrobial)
Trimethoprim	$3.20(\pm0.20)\times10^6$		Pharmaceutical (Antibacterial, Folic acid biosynthesis inhibitor)
Venlafaxine	$9.03(\pm0.02)\times10^4$		Pharmaceutical (Antidepressant, Serotonin-noradrenaline reuptake inhibitor (SNRI))
Zidovudine	$6.70(\pm0.31)\times10^4$		Pharmaceutical (Antiviral, Reverse transcriptase inhibitor)

[a] See references and experimental details in Table S17. [b] Mean values of multiple measurements.

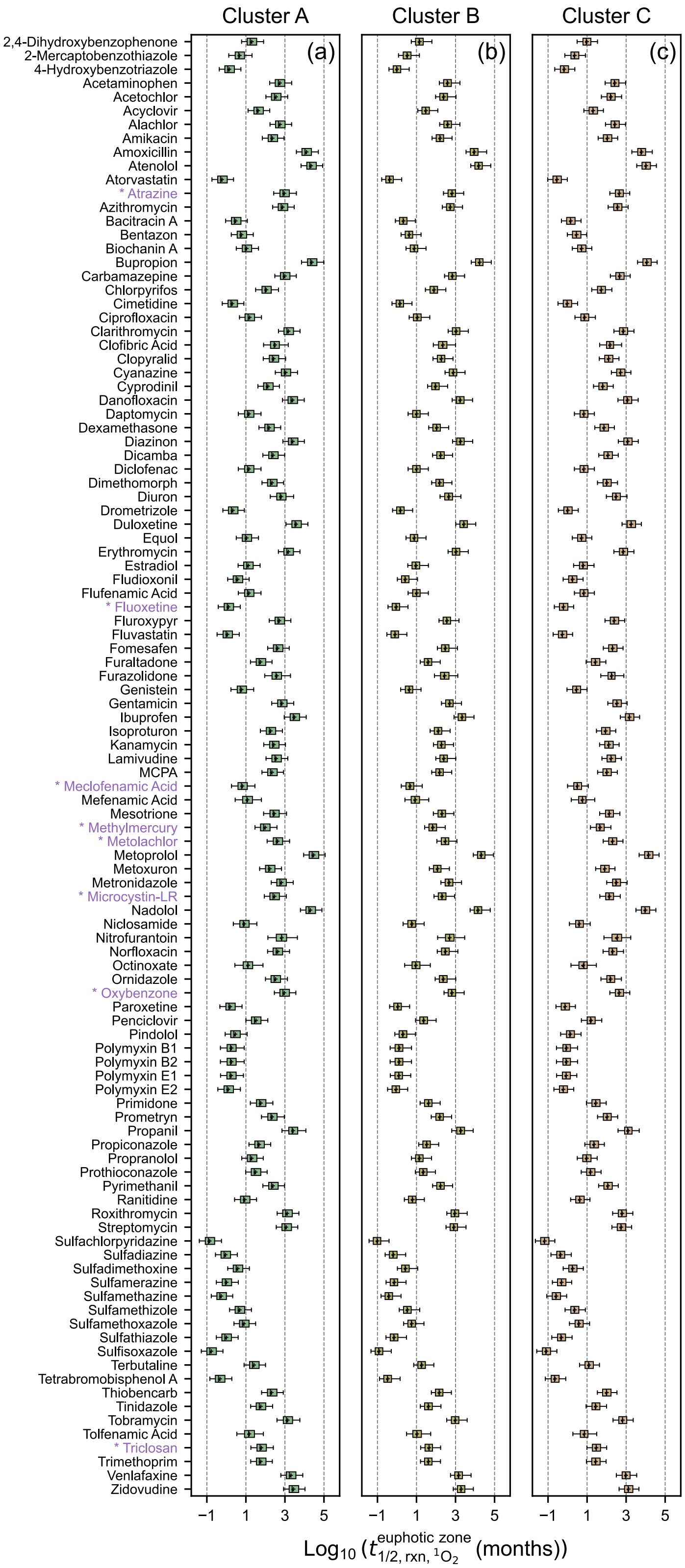

Figure S37. Half-lives of 106 compounds attributable to reaction with $^1O_2$ in the euphotic zone ($t^{\text{euphotic zone}}_{1/2,\ \text{rxn},\ ^1O_2}$; $\log_{10}$-transformed) of ALTM lakes experiencing **(a)** mild (cluster A), **(b)** moderate (cluster B), and **(c)** intense browning (cluster C). Each box spans the 25th to 75th percentiles, with whiskers extending to 1.5 times the interquartile range below the 25th and above the 75th percentiles. The centerline and "+" sign mark the median and mean, respectively. Compounds (n=8) highlighted in purple and marked with an asterisk denote those previously detected in Adirondack lake waters.[61-63]

18. Literature survey of total quenching and bimolecular reaction rate constants with $^1O_2$

Table S17. Summary of $k_{tot,\,^1O_2}$ and $k_{rxn,\,^1O_2}$

Reference	Compound	$k_{tot,\,^1O_2}$ or $k_{rxn,\,^1O_2}$ (M^{-1} s^{-1})	Method	Sensitizer	Reference Compound	Solvent	pH
Latch and McNeill 2003 [64]	Cimetidine	$1.40(\pm0.50)\times10^{8}$	Competition kinetics	Rose Bengal	Furfuryl alcohol ($k_{FFA,\,^1O_2} = 2.0(\pm0.1)\times10^{7}$ M^{-1} s^{-1} in EtOH)	Ethanol (EtOH)	NA
Latch and McNeill 2003	Cimetidine	$3.30(\pm0.30)\times10^{6}$	Competition kinetics	Perinaphthenone	FFA ($k_{FFA,\,^1O_2} = 8.3(\pm0.1)\times10^{7}$ M^{-1} s^{-1} in D_2O)	H_2O	4.2
Latch and McNeill 2003	Cimetidine	$9.20(\pm0.60)\times10^{7}$	Competition kinetics	Rose Bengal	FFA ($k_{FFA,\,^1O_2} = 8.3\times10^{7}$ M^{-1} s^{-1})	H_2O	6.9
Latch and McNeill 2003	Cimetidine	$2.20(\pm0.20)\times10^{8}$	Competition kinetics	Rose Bengal	FFA ($k_{FFA,\,^1O_2} = 8.3\times10^{7}$ M^{-1} s^{-1})	H_2O	8.2
Latch and McNeill 2003	Cimetidine	$2.50(\pm0.20)\times10^{8}$	Competition kinetics	Rose Bengal	FFA ($k_{FFA,\,^1O_2} = 8.3\times10^{7}$ M^{-1} s^{-1})	H_2O	10.2
Latch and McNeill 2003	Ranitidine	$9.00(\pm3.00)\times10^{6}$	Competition kinetics	Rose Bengal	FFA ($k_{FFA,\,^1O_2} = 2.0\times10^{7}$ M^{-1} s^{-1})	EtOH	NA
Latch and McNeill 2003	Ranitidine	$2.10(\pm0.20)\times10^{7}$	Competition kinetics	Rose Bengal	FFA ($k_{FFA,\,^1O_2} = 8.3\times10^{7}$ M^{-1} s^{-1})	H_2O	7.2
Latch and McNeill 2003	Cimetidine	$1.40(\pm0.50)\times10^{8}$	Time-resolved phosphorescence	Rose Bengal	NA	D_2O	7.1
Latch and McNeill 2003	Cimetidine	$1.62(\pm0.02)\times10^{8}$	Time-resolved phosphorescence	Rose Bengal	NA	EtOH	NA
Latch and McNeill 2003	4,5-Dimethyl-1H-imidazole	$1.93(\pm0.05)\times10^{8}$	Time-resolved phosphorescence	Rose Bengal	NA	EtOH	NA
Latch and McNeill 2003	Diethyl Sulfide	$1.62(\pm0.02)\times10^{7}$	Time-resolved phosphorescence	Rose Bengal	NA	EtOH	NA
Latch and McNeill 2003	Cimetidine Sulfoxide	$7.50(\pm0.10)\times10^{7}$	Time-resolved phosphorescence	Rose Bengal	NA	EtOH	NA
Latch and McNeill 2003	Ranitidine	$1.60(\pm0.20)\times10^{7}$	Time-resolved phosphorescence	Eosin Y	NA	D_2O	6.4
Latch and McNeill 2003	Ranitidine	$2.65(\pm0.07)\times10^{7}$	Time-resolved phosphorescence	Rose Bengal	NA	D_2O	7.5
Latch and McNeill 2003	Ranitidine	$6.40(\pm0.40)\times10^{7}$	Time-resolved phosphorescence	Eosin Y	NA	D_2O	9.8
Latch and McNeill 2003	Ranitidine	$4.54(\pm0.04)\times10^{7}$	Time-resolved phosphorescence	Rose Bengal	NA	EtOH	NA
Latch and McNeill 2003	N,N,5-Trimethylfurfurylamine	$1.24(\pm0.04)\times10^{8}$	Time-resolved phosphorescence	Rose Bengal	NA	EtOH	NA
Latch and McNeill 2003	2,5-Dimethylfuran	$3.30(\pm0.20)\times10^{8}$	Time-resolved phosphorescence	Rose Bengal	NA	EtOH	NA
Latch and McNeill 2003	Furfuryl Methyl Sulfide	$4.06(\pm0.08)\times10^{7}$	Time-resolved phosphorescence	Rose Bengal	NA	EtOH	NA
Packer and Arnold 2003 [65]	Naproxen	$1.10(\pm0.10)\times10^{5}$	Time-resolved phosphorescence	Perinaphthenone	NA	EtOH	NA
Boreen and McNeill 2004 [66]	Sulfamethoxazole	$2.00(\pm1.00)\times10^{4}$	Time-resolved phosphorescence	Rose Bengal	NA	Acetone	NA
Boreen and McNeill 2004	Sulfisoxazole	$6.50(\pm0.60)\times10^{7}$	Time-resolved phosphorescence	Rose Bengal	NA	D_2O	9.5
Boreen and McNeill 2004	Sulfisoxazole	$1.00(\pm0.30)\times10^{8}$	Time-resolved phosphorescence	Rose Bengal	NA	D_2O	11.1
Boreen and McNeill 2004	Sulfamethizole	$3.60(\pm0.80)\times10^{6}$	Time-resolved phosphorescence	Rose Bengal	NA	D_2O	8.7
Boreen and McNeill 2004	Sulfathiazole	$5.60(\pm0.70)\times10^{7}$	Time-resolved phosphorescence	Rose Bengal	NA	D_2O	9.5
Boreen and McNeill 2004	Sulfathiazole	$1.00(\pm0.20)\times10^{8}$	Time-resolved phosphorescence	Rose Bengal	NA	D_2O	11.1
Boreen and McNeill 2004	Sulfamoxole	$3.00(\pm0.70)\times10^{8}$	Time-resolved phosphorescence	Rose Bengal	NA	D_2O	9.5
Boreen and McNeill 2004	Sulfisoxazole	$5.50(\pm0.40)\times10^{7}$	Competition kinetics	Rose Bengal	FFA ($k_{FFA,\,^1O_2} = 8.3\times10^{7}$ M^{-1} s^{-1})	H_2O	10.2
Boreen and McNeill 2004	Sulfathiazole	$6.90(\pm0.30)\times10^{7}$	Competition kinetics	Rose Bengal	FFA ($k_{FFA,\,^1O_2} = 8.3\times10^{7}$ M^{-1} s^{-1})	H_2O	10.2
Latch and McNeill 2005 [67]	Triclosan	$3.00(\pm0.40)\times10^{6}$	Competition kinetics	Rose Bengal	FFA ($k_{FFA,\,^1O_2} = 8.3\times10^{7}$ M^{-1} s^{-1})	H_2O	5
Latch and McNeill 2005	Triclosan	$1.07(\pm0.06)\times10^{8}$	Competition kinetics	Rose Bengal	FFA ($k_{FFA,\,^1O_2} = 8.3\times10^{7}$ M^{-1} s^{-1})	H_2O	10
Latch and McNeill 2005	Triclosan	$1.30(\pm0.20)\times10^{8}$	Time-resolved phosphorescence	Rose Bengal	NA	D_2O	9
Latch and McNeill 2005	Triclosan	$2.10(\pm0.20)\times10^{8}$	Time-resolved phosphorescence	Rose Bengal	NA	D_2O	10
Latch and McNeill 2005	Triclosan	$2.10(\pm0.20)\times10^{8}$	Time-resolved phosphorescence	Rose Bengal	NA	D_2O	11
Latch and McNeill 2005	Triclosan	$2.20(\pm0.20)\times10^{8}$	Time-resolved phosphorescence	Rose Bengal	NA	D_2O	12
Edhlund and McNeill 2006 [68]	Furaltadone	$3.30(\pm0.20)\times10^{6}$	Competition kinetics	Rose Bengal	Furfural ($k_{Furfural,\,^1O_2} = 1.11\times10^{6}$ M^{-1} s^{-1} in ACN)	H_2O	7
Edhlund and McNeill 2006	Furazolidone	$5.00(\pm2.00)\times10^{5}$	Competition kinetics	Rose Bengal	Furfural ($k_{Furfural,\,^1O_2} = 1.11\times10^{6}$ M^{-1} s^{-1})	H_2O	7
Edhlund and McNeill 2006	Nitrofurantoin	$3.00(\pm2.00)\times10^{5}$	Competition kinetics	Rose Bengal	Furfural ($k_{Furfural,\,^1O_2} = 1.11\times10^{6}$ M^{-1} s^{-1})	H_2O	7
Edhlund and McNeill 2006	Furaltadone	$2.00(\pm1.00)\times10^{8}$	Time-resolved phosphorescence	Rose Bengal	NA	D_2O	NA
Edhlund and McNeill 2006	Furazolidone	$4.40(\pm0.20)\times10^{7}$	Time-resolved phosphorescence	Rose Bengal	NA	Acetonitrile (ACN)	7
Edhlund and McNeill 2006	Nitrofurantoin	$4.00(\pm0.50)\times10^{7}$	Time-resolved phosphorescence	Rose Bengal	NA	Acetonitrile (ACN)	7
ter Halle and Richard 2006 [69]	Mesotrione	$6.70(\pm0.30)\times10^{5}$	Competition kinetics	Rose Bengal	FFA ($k_{FFA,\,^1O_2} = 1.2\times10^{8}$ M^{-1} s^{-1})	H_2O	6.5
Castillo and García 2007 [70]	Doxycycline	1.40×10^{6}	Competition kinetics	Rose Bengal	Tryptophan ($k_{Tryptophan,\,^1O_2} = 6\times10^{6}$ M^{-1} s^{-1} in MeOH[71])	H_2O:MeOH (70:30)	NA
Castillo and García 2007	Methacycline	2.30×10^{6}	Competition kinetics	Rose Bengal	Tryptophan ($k_{Tryptophan,\,^1O_2} = 6\times10^{6}$ M^{-1} s^{-1})	H_2O:MeOH (70:30)	NA
Castillo and García 2007	Chlorotetracycline	1.50×10^{6}	Competition kinetics	Rose Bengal	Tryptophan ($k_{Tryptophan,\,^1O_2} = 6\times10^{6}$ M^{-1} s^{-1})	H_2O:MeOH (70:30)	NA
Castillo and García 2007	Demeclocycline	1.50×10^{6}	Competition kinetics	Rose Bengal	Tryptophan ($k_{Tryptophan,\,^1O_2} = 6\times10^{6}$ M^{-1} s^{-1})	H_2O:MeOH (70:30)	NA
Castillo and García 2007	Oxytetracycline	1.10×10^{6}	Competition kinetics	Rose Bengal	Tryptophan ($k_{Tryptophan,\,^1O_2} = 6\times10^{6}$ M^{-1} s^{-1})	H_2O:MeOH (70:30)	NA
Castillo and García 2007	Doxycycline	1.40×10^{6}	Time-resolved phosphorescence	Rose Bengal	NA	MeOD	NA
Castillo and García 2007	Methacycline	2.30×10^{6}	Time-resolved phosphorescence	Rose Bengal	NA	MeOD	NA
Castillo and García 2007	Chlorotetracycline	1.60×10^{6}	Time-resolved phosphorescence	Rose Bengal	NA	MeOD	NA
Castillo and García 2007	Demeclocycline	2.50×10^{6}	Time-resolved phosphorescence	Rose Bengal	NA	MeOD	NA
Castillo and García 2007	Oxytetracycline	2.10×10^{6}	Time-resolved phosphorescence	Rose Bengal	NA	MeOD	NA

Table S17. Summary of $k_{tot, ^1O_2}$ and $k_{rxn, ^1O_2}$ (continued)

Reference	Compound	$k_{tot, ^1O_2}$ or $k_{rxn, ^1O_2}$ (M^{-1} s^{-1})	Method	Sensitizer	Reference Compound	Solvent	pH
Barbieri and García 2008 [72]	Bisphenol A	3.00×10^5	Competition kinetics	Rose Bengal	FFA ($k_{FFA, ^1O_2}$ = 6.2×10^7 M^{-1} s^{-1} in H_2O:MeOH (50:50))	H_2O:MeOH (50:50)	NA
Barbieri and García 2008	Bisphenol A	1.01×10^8	Competition kinetics	Rose Bengal	FFA ($k_{FFA, ^1O_2}$ = 1.2×10^8 M^{-1} s^{-1} in H_2O[36, 73])	H_2O	10
Barbieri and García 2008	Bisphenol A	2.00×10^8	Competition kinetics	Rose Bengal	FFA ($k_{FFA, ^1O_2}$ = 1.2×10^8 M^{-1} s^{-1})	H_2O	12
Barbieri and García 2008	4,4'-Isopropylidenebis(2,6-dibromophenol)	9.00×10^5	Competition kinetics	Rose Bengal	FFA ($k_{FFA, ^1O_2}$ = 6.2×10^7 M^{-1} s^{-1})	H_2O:MeOH (50:50)	NA
Barbieri and García 2008	4,4'-Isopropylidenebis(2,6-dibromophenol)	1.50×10^8	Competition kinetics	Rose Bengal	FFA ($k_{FFA, ^1O_2}$ = 1.2×10^8 M^{-1} s^{-1})	H_2O	12
Barbieri and García 2008	4,4'-isopropylidenebis(2,6-dimethylphenol)	1.20×10^6	Competition kinetics	Rose Bengal	FFA ($k_{FFA, ^1O_2}$ = 6.2×10^7 M^{-1} s^{-1})	H_2O:MeOH (50:50)	NA
Barbieri and García 2008	4,4'-isopropylidenebis(2,6-dimethylphenol)	1.40×10^8	Competition kinetics	Rose Bengal	FFA ($k_{FFA, ^1O_2}$ = 1.2×10^8 M^{-1} s^{-1})	H_2O	12
Barbieri and García 2008	Bisphenol A	1.70×10^6	Time-resolved phosphorescence	Rose Bengal	NA	D_2O:MeOD (50:50)	NA
Barbieri and García 2008	Bisphenol A	4.80×10^8	Time-resolved phosphorescence	Rose Bengal	NA	D_2O	10
Barbieri and García 2008	Bisphenol A	7.40×10^8	Time-resolved phosphorescence	Rose Bengal	NA	D_2O	12
Barbieri and García 2008	4,4'-Isopropylidenebis(2,6-dibromophenol)	6.40×10^7	Time-resolved phosphorescence	Rose Bengal	NA	D_2O:MeOD (50:50)	NA
Barbieri and García 2008	4,4'-isopropylidenebis(2,6-dimethylphenol)	3.30×10^7	Time-resolved phosphorescence	Rose Bengal	NA	D_2O:MeOD (50:50)	NA
Boreen and McNeill 2008 [74]	Tyrosine	$9.00(\pm2.00)\times10^6$	Competition kinetics	Rose Bengal	FFA ($k_{FFA, ^1O_2}$ = 8.3×10^7 M^{-1} s^{-1})	H_2O	8.5
Boreen and McNeill 2008	Methionine	$2.20(\pm0.70)\times10^7$	Competition kinetics	Rose Bengal	FFA ($k_{FFA, ^1O_2}$ = 8.3×10^7 M^{-1} s^{-1})	H_2O	8.5
Boreen and McNeill 2008	Histidine	$6.60(\pm0.80)\times10^7$	Competition kinetics	Rose Bengal	FFA ($k_{FFA, ^1O_2}$ = 8.3×10^7 M^{-1} s^{-1})	H_2O	8.5
Boreen and McNeill 2008	Tryptophan	$1.80(\pm0.20)\times10^8$	Competition kinetics	Rose Bengal	FFA ($k_{FFA, ^1O_2}$ = 8.3×10^7 M^{-1} s^{-1})	H_2O	8.5
Boreen and McNeill 2008	Tyrosine	$2.40(\pm0.20)\times10^7$	Competition kinetics	Molybdate+H_2O_2	FFA ($k_{FFA, ^1O_2}$ = 8.3×10^7 M^{-1} s^{-1})	H_2O	10
Boreen and McNeill 2008	Tryptophan	$5.30(\pm0.20)\times10^7$	Competition kinetics	Molybdate+H_2O_2	FFA ($k_{FFA, ^1O_2}$ = 8.3×10^7 M^{-1} s^{-1})	H_2O	10
Boreen and McNeill 2008	Tyrosine	$1.38(\pm0.09)\times10^7$	Time-resolved phosphorescence	Rose Bengal	NA	D_2O	8.9
Boreen and McNeill 2008	Methionine	$1.46(\pm0.02)\times10^7$	Time-resolved phosphorescence	Rose Bengal	NA	D_2O	8.9
Boreen and McNeill 2008	Histidine	$8.90(\pm0.50)\times10^7$	Time-resolved phosphorescence	Rose Bengal	NA	D_2O	8.9
Boreen and McNeill 2008	Tryptophan	$2.30(\pm1.00)\times10^8$	Time-resolved phosphorescence	Rose Bengal	NA	D_2O	8.9
Chen and Qu 2009 [75]	Propranolol	$9.30(\pm0.40)\times10^6$	Competition kinetics	Rose Bengal	FFA ($k_{FFA, ^1O_2}$ = 1.2×10^8 M^{-1} s^{-1})	H_2O	8
Malouki and Richard 2009	2-Mercaptobenzothiazole	$3.90(\pm0.80)\times10^7$	Competition kinetics	Rose Bengal	FFA ($k_{FFA, ^1O_2}$ = 1.2×10^8 M^{-1} s^{-1})	H_2O	6.5
Malouki and Richard 2009	Prometryn	$8.40(\pm1.40)\times10^5$	Competition kinetics	Rose Bengal	FFA ($k_{FFA, ^1O_2}$ = 1.2×10^8 M^{-1} s^{-1})	H_2O	6.5
Malouki and Richard 2009	Mesotrione	$5.30(\pm1.30)\times10^5$	Competition kinetics	Rose Bengal	FFA ($k_{FFA, ^1O_2}$ = 1.2×10^8 M^{-1} s^{-1})	H_2O	6.5
Dell'Arciprete and Gonzalez 2010 [76]	Imidacloprid	$5.50(\pm0.50)\times10^6$	Time-resolved phosphorescence	Rose Bengal	NA	D_2O	NA
Dell'Arciprete and Gonzalez 2010	Thiacloprid	$3.90(\pm1.00)\times10^7$	Time-resolved phosphorescence	Rose Bengal	NA	D_2O	NA
Dell'Arciprete and Gonzalez 2010	Acetamiprid	$1.30(\pm1.00)\times10^6$	Time-resolved phosphorescence	Rose Bengal	NA	D_2O	NA
Razavi and Cooper 2011 [77]	Atorvastatin	$3.10(\pm0.20)\times10^8$	Competition kinetics	Rose Bengal	FFA ($k_{FFA, ^1O_2}$ = 8.3×10^7 M^{-1} s^{-1})	H_2O	5
Remucal and McNeill 2011 [78]	Histidine	$6.00(\pm0.10)\times10^7$	Competition kinetics	Riboflavin	FFA ($k_{FFA, ^1O_2}$ = 8.3×10^7 M^{-1} s^{-1})	H_2O	7.8
Remucal and McNeill 2011	Methionine	$9.00(\pm1.00)\times10^6$	Competition kinetics	Riboflavin	FFA ($k_{FFA, ^1O_2}$ = 8.3×10^7 M^{-1} s^{-1})	H_2O	7.8
Remucal and McNeill 2011	Tryptophan	$2.20(\pm0.40)\times10^7$	Competition kinetics	Riboflavin	FFA ($k_{FFA, ^1O_2}$ = 8.3×10^7 M^{-1} s^{-1})	H_2O	7.8
Remucal and McNeill 2011	Tryptophan	$2.30(\pm0.80)\times10^7$	Competition kinetics	Perinaphthenone	FFA ($k_{FFA, ^1O_2}$ = 8.3×10^7 M^{-1} s^{-1})	H_2O	7.8
Xu and Song 2011 [79]	Amoxicillin	1.44×10^4	Competition kinetics	Rose Bengal	Furfural ($k_{Furfural, ^1O_2}$ = 8.4×10^4 M^{-1} s^{-1} in MeOH[80])	H_2O	7
Vione and Richard 2011 [81]	Ibuprofen	$6.00(\pm0.58)\times10^4$	Competition kinetics	Rose Bengal	FFA ($k_{FFA, ^1O_2}$ = 1.2×10^8 M^{-1} s^{-1})	H_2O	8
Maddigapu and Vione 2011 [82]	2,4-Dichloro-6-nitrophenol	$3.70(\pm1.40)\times10^7$	Kinetic modeling	Rose Bengal	NA	H_2O	8
Felcyn and Latch 2012 [83]	Biochanin A	$1.70(\pm0.20)\times10^7$	Competition kinetics	Molybdate+H_2O_2	FFA ($k_{FFA, ^1O_2}$ = 8.3×10^7 M^{-1} s^{-1})	H_2O	7
Felcyn and Latch 2012	Biochanin A	$6.70(\pm0.70)\times10^7$	Competition kinetics	Molybdate+H_2O_2	FFA ($k_{FFA, ^1O_2}$ = 8.3×10^7 M^{-1} s^{-1})	H_2O	8
Felcyn and Latch 2012	Biochanin A	$8.90(\pm1.50)\times10^7$	Competition kinetics	Molybdate+H_2O_2	FFA ($k_{FFA, ^1O_2}$ = 8.3×10^7 M^{-1} s^{-1})	H_2O	10
Felcyn and Latch 2012	Genistein	$3.10(\pm0.50)\times10^7$	Competition kinetics	Molybdate+H_2O_2	FFA ($k_{FFA, ^1O_2}$ = 8.3×10^7 M^{-1} s^{-1})	H_2O	7
Felcyn and Latch 2012	Genistein	$3.20(\pm0.70)\times10^7$	Competition kinetics	Molybdate+H_2O_2	FFA ($k_{FFA, ^1O_2}$ = 8.3×10^7 M^{-1} s^{-1})	H_2O	8
Felcyn and Latch 2012	Genistein	$1.10(\pm0.10)\times10^8$	Competition kinetics	Molybdate+H_2O_2	FFA ($k_{FFA, ^1O_2}$ = 8.3×10^7 M^{-1} s^{-1})	H_2O	10
Felcyn and Latch 2012	Equol	$1.70(\pm0.20)\times10^7$	Competition kinetics	Molybdate+H_2O_2	FFA ($k_{FFA, ^1O_2}$ = 8.3×10^7 M^{-1} s^{-1})	H_2O	7
Felcyn and Latch 2012	Equol	$4.00(\pm0.30)\times10^7$	Competition kinetics	Molybdate+H_2O_2	FFA ($k_{FFA, ^1O_2}$ = 8.3×10^7 M^{-1} s^{-1})	H_2O	8
Felcyn and Latch 2012	Equol	$3.10(\pm0.80)\times10^8$	Competition kinetics	Molybdate+H_2O_2	FFA ($k_{FFA, ^1O_2}$ = 8.3×10^7 M^{-1} s^{-1})	H_2O	10
Felcyn and Latch 2012	Biochanin A	$1.90(\pm0.20)\times10^8$	Competition kinetics	Perinaphthenone	FFA ($k_{FFA, ^1O_2}$ = 8.3×10^7 M^{-1} s^{-1})	H_2O	7
Felcyn and Latch 2012	Biochanin A	$4.50(\pm0.30)\times10^8$	Competition kinetics	Perinaphthenone	FFA ($k_{FFA, ^1O_2}$ = 8.3×10^7 M^{-1} s^{-1})	H_2O	8
Felcyn and Latch 2012	Biochanin A	$3.80(\pm0.50)\times10^8$	Competition kinetics	Perinaphthenone	FFA ($k_{FFA, ^1O_2}$ = 8.3×10^7 M^{-1} s^{-1})	H_2O	10
Felcyn and Latch 2012	Genistein	$5.90(\pm0.40)\times10^7$	Competition kinetics	Perinaphthenone	FFA ($k_{FFA, ^1O_2}$ = 8.3×10^7 M^{-1} s^{-1})	H_2O	7
Felcyn and Latch 2012	Genistein	$1.56(\pm0.07)\times10^8$	Competition kinetics	Perinaphthenone	FFA ($k_{FFA, ^1O_2}$ = 8.3×10^7 M^{-1} s^{-1})	H_2O	8
Felcyn and Latch 2012	Genistein	$1.09(\pm0.08)\times10^8$	Competition kinetics	Perinaphthenone	FFA ($k_{FFA, ^1O_2}$ = 8.3×10^7 M^{-1} s^{-1})	H_2O	10
Felcyn and Latch 2012	Equol	$2.34(\pm0.08)\times10^8$	Competition kinetics	Perinaphthenone	FFA ($k_{FFA, ^1O_2}$ = 8.3×10^7 M^{-1} s^{-1})	H_2O	7
Felcyn and Latch 2012	Equol	$1.67(\pm0.20)\times10^8$	Competition kinetics	Perinaphthenone	FFA ($k_{FFA, ^1O_2}$ = 8.3×10^7 M^{-1} s^{-1})	H_2O	8

**Table S17.** Summary of $k_{tot,\ ^1O_2}$ and $k_{rxn,\ ^1O_2}$ (continued)							
Reference	Compound	$k_{tot,\ ^1O_2}$ or $k_{rxn,\ ^1O_2}$ ($M^{-1}\ s^{-1}$)	Method	Sensitizer	Reference Compound	Solvent	pH
Felcyn and Latch 2012	Equol	$2.70(\pm0.20)\times10^{8}$	Competition kinetics	Perinaphthenone	FFA ($k_{FFA,\ ^1O_2} = 8.3\times10^{7}\ M^{-1}\ s^{-1}$)	H_2O	10
Kelly and Arnold 2012 [84]	Genistein	$3.57(\pm0.08)\times10^{7}$	Competition kinetics	Rose Bengal	FFA ($k_{FFA,\ ^1O_2} = 1.2\times10^{8}\ M^{-1}\ s^{-1}$)	H_2O	8.5
Kelly and Arnold 2012	Daidzein	$1.84(\pm0.03)\times10^{7}$	Competition kinetics	Rose Bengal	FFA ($k_{FFA,\ ^1O_2} = 1.2\times10^{8}\ M^{-1}\ s^{-1}$)	H_2O	8.5
Santoke and Cooper 2012 [85]	Duloxetine	$4.89(\pm0.45)\times10^{4}$	Competition kinetics	Rose Bengal	Furfural ($k_{Furfural,\ ^1O_2} = 8.4\times10^{4}\ M^{-1}\ s^{-1}$)	H_2O	NA
Santoke and Cooper 2012	Venlafaxine	$9.03(\pm0.04)\times10^{4}$	Competition kinetics	Rose Bengal	Furfural ($k_{Furfural,\ ^1O_2} = 8.4\times10^{4}\ M^{-1}\ s^{-1}$)	H_2O	NA
Santoke and Cooper 2012	Bupropion	$7.70(\pm0.93)\times10^{3}$	Competition kinetics	Rose Bengal	Furfural ($k_{Furfural,\ ^1O_2} = 8.4\times10^{4}\ M^{-1}\ s^{-1}$)	H_2O	NA
Monadjemi and Richard 2012 [86]	Cycloxydim	$1.00(\pm0.20)\times10^{7}$	Kinetic modeling	Perinaphthenone	NA	ACN	NA
Wang and Song 2012 [87]	Atenolol	$8.47(\pm0.56)\times10^{3}$	Competition kinetics	Rose Bengal	Furfural ($k_{Furfural,\ ^1O_2} = 8.4\times10^{4}\ M^{-1}\ s^{-1}$)	H_2O	7
Wang and Song 2012	Metoprolol	$6.27(\pm0.25)\times10^{3}$	Competition kinetics	Rose Bengal	Furfural ($k_{Furfural,\ ^1O_2} = 8.4\times10^{4}\ M^{-1}\ s^{-1}$)	H_2O	7
Wang and Song 2012	Nadolol	$9.18(\pm0.37)\times10^{3}$	Competition kinetics	Rose Bengal	Furfural ($k_{Furfural,\ ^1O_2} = 8.4\times10^{4}\ M^{-1}\ s^{-1}$)	H_2O	7
Luo and Song 2012 [88]	Trimethoprim	$3.20(\pm0.20)\times10^{6}$	Competition kinetics	Rose Bengal	FFA ($k_{FFA,\ ^1O_2} = 8.3\times10^{7}\ M^{-1}\ s^{-1}$)	H_2O	6
Luo and Song 2012	2,4-Diaminopyrimidine	$3.00(\pm0.10)\times10^{6}$	Competition kinetics	Rose Bengal	FFA ($k_{FFA,\ ^1O_2} = 8.3\times10^{7}\ M^{-1}\ s^{-1}$)	H_2O	6
De Laurentiis and Vione 2012 [89]	Carbamazepine	$1.88(\pm0.09)\times10^{5}$	Competition kinetics	Rose Bengal	FFA ($k_{FFA,\ ^1O_2} = 1.2\times10^{8}\ M^{-1}\ s^{-1}$)	H_2O	7
Yang and Song 2013 [90]	Terbutaline	$7.10(\pm0.30)\times10^{6}$	Competition kinetics	Rose Bengal	FFA ($k_{FFA,\ ^1O_2} = 1.2\times10^{8}\ M^{-1}\ s^{-1}$)	H_2O	7
Vione and Minero 2013 [91]	Oxybenzone (Benzophenone-3)	$1.99(\pm0.08)\times10^{5}$	Competition kinetics	Rose Bengal	FFA ($k_{FFA,\ ^1O_2} = 1.2\times10^{8}\ M^{-1}\ s^{-1}$)	H_2O	6.5
Zeng and Arnold 2013 [92]	Atrazine	$2.00(\pm0.30)\times10^{5}$	Quencher experiments	NA	NA	H_2O	8.5
Zeng and Arnold 2013	Cyanazine	$1.70(\pm0.20)\times10^{5}$	Quencher experiments	NA	NA	H_2O	8.5
Zeng and Arnold 2013	Acetochlor	$5.30(\pm0.10)\times10^{5}$	Quencher experiments	NA	NA	H_2O	8.5
Zeng and Arnold 2013	Alachlor	$3.30(\pm0.10)\times10^{5}$	Quencher experiments	NA	NA	H_2O	8.5
Zeng and Arnold 2013	Metolachlor	$4.40(\pm0.60)\times10^{5}$	Quencher experiments	NA	NA	H_2O	8.5
Zeng and Arnold 2013	Mesotrione	$6.90(\pm0.40)\times10^{5}$	Quencher experiments	NA	NA	H_2O	8.5
Zeng and Arnold 2013	Diuron	$2.90(\pm0.60)\times10^{5}$	Quencher experiments	NA	NA	H_2O	8.5
Zeng and Arnold 2013	Isoproturon	$1.00(\pm0.10)\times10^{6}$	Quencher experiments	NA	NA	H_2O	8.5
Zeng and Arnold 2013	Metoxuron	$1.10(\pm0.10)\times10^{6}$	Quencher experiments	NA	NA	H_2O	8.5
Zeng and Arnold 2013	Bentazon	$3.10(\pm0.30)\times10^{7}$	Quencher experiments	NA	NA	H_2O	8.5
Zeng and Arnold 2013	Clopyralid	$7.00(\pm0.90)\times10^{5}$	Quencher experiments	NA	NA	H_2O	8.5
Zeng and Arnold 2013	Chlorpyrifos	$1.90(\pm0.20)\times10^{6}$	Quencher experiments	NA	NA	H_2O	8.5
Zeng and Arnold 2013	Propiconazole	$3.90(\pm0.30)\times10^{6}$	Quencher experiments	NA	NA	H_2O	8.5
Yan and Song 2014 [93]	Microcystin-LR	6.50×10^{5}	Competition kinetics	Rose Bengal	Furfural ($k_{Furfural,\ ^1O_2} = 8.4\times10^{4}\ M^{-1}\ s^{-1}$)	H_2O	7
De Laurentiis and Vione 2014 [94]	Acetaminophen	$3.68(\pm0.73)\times10^{7}$	Kinetic modeling	Rose Bengal	NA	H_2O	7
Avetta and Vione 2014 [95]	Dimethomorph	$8.50(\pm0.28)\times10^{5}$	Kinetic modeling	Rose Bengal	NA	H_2O	7
Zhou and Chen 2015 [96]	Acyclovir	$2.38(\pm0.05)\times10^{6}$	Competition kinetics	Rose Bengal	FFA ($k_{FFA,\ ^1O_2} = 1.2\times10^{8}\ M^{-1}\ s^{-1}$)	H_2O	7
Zhou and Chen 2015	Zidovudine	$6.70(\pm0.31)\times10^{4}$	Competition kinetics	Rose Bengal	FFA ($k_{FFA,\ ^1O_2} = 1.2\times10^{8}\ M^{-1}\ s^{-1}$)	H_2O	7
Zhou and Chen 2015	Lamivudine	$5.20(\pm0.42)\times10^{5}$	Competition kinetics	Rose Bengal	FFA ($k_{FFA,\ ^1O_2} = 1.2\times10^{8}\ M^{-1}\ s^{-1}$)	H_2O	7
Janssen and McNeill 2015 [97]	4-Hydroxybenzotriazole	$1.31(\pm0.04)\times10^{8}$	Competition kinetics	Rose Bengal	FFA ($k_{FFA,\ ^1O_2} = 8.3\times10^{7}\ M^{-1}\ s^{-1}$)	H_2O	7.5
Janssen and McNeill 2015	Indole	$4.34(\pm0.01)\times10^{7}$	Competition kinetics	Rose Bengal	FFA ($k_{FFA,\ ^1O_2} = 8.3\times10^{7}\ M^{-1}\ s^{-1}$)	H_2O	7.5
Chu and McNeill 2015 [98]	Histidine	6.50×10^{7}	Competition kinetics	Rose Bengal	FFA ($k_{FFA,\ ^1O_2} = 1.2\times10^{8}\ M^{-1}\ s^{-1}$)	H_2O	7.7
Chu and McNeill 2015	Histidine	2.00×10^{4}	Competition kinetics	Rose Bengal	FFA ($k_{FFA,\ ^1O_2} = 1.2\times10^{8}\ M^{-1}\ s^{-1}$)	D_2O	2.5
Chu and McNeill 2015	Histamine	8.80×10^{7}	Competition kinetics	Rose Bengal	FFA ($k_{FFA,\ ^1O_2} = 8.3\times10^{7}\ M^{-1}\ s^{-1}$)	H_2O	7.7
Chu and McNeill 2015	Histamine	2.80×10^{4}	Competition kinetics	Rose Bengal	FFA ($k_{FFA,\ ^1O_2} = 8.3\times10^{7}\ M^{-1}\ s^{-1}$)	D_2O	2.5
Vione and Calza 2015 [99]	Ethylhexyl Methoxycinnamate (Octinoxate)	$1.54(\pm0.88)\times10^{7}$	Competition kinetics	Rose Bengal	Acetaminophen ($k_{Acetaminophen,\ ^1O_2} = 3.68(\pm0.73)\times10^{7}\ M^{-1}\ s^{-1}$ in H_2O[94])	H_2O	7
An and An 2016 [100]	Acyclovir	$4.36(\pm0.32)\times10^{6}$	Competition kinetics	Molybdate+H_2O_2	Furfural ($k_{Furfural,\ ^1O_2} = 8.4\times10^{4}\ M^{-1}\ s^{-1}$)	H_2O	7
An and An 2016	Penciclovir	$5.56(\pm0.02)\times10^{6}$	Competition kinetics	Molybdate+H_2O_2	Furfural ($k_{Furfural,\ ^1O_2} = 8.4\times10^{4}\ M^{-1}\ s^{-1}$)	H_2O	7
An and An 2016	Guanine	$3.92(\pm0.02)\times10^{6}$	Competition kinetics	Molybdate+H_2O_2	Furfural ($k_{Furfural,\ ^1O_2} = 8.4\times10^{4}\ M^{-1}\ s^{-1}$)	H_2O	7
An and An 2016	Isocytosine	$8.54(\pm0.13)\times10^{4}$	Competition kinetics	Molybdate+H_2O_2	Furfural ($k_{Furfural,\ ^1O_2} = 8.4\times10^{4}\ M^{-1}\ s^{-1}$)	H_2O	7
An and An 2016	Imidazole	$6.05(\pm0.05)\times10^{6}$	Competition kinetics	Molybdate+H_2O_2	Furfural ($k_{Furfural,\ ^1O_2} = 8.4\times10^{4}\ M^{-1}\ s^{-1}$)	H_2O	7
An and An 2016	Acyclovir	$2.18(\pm0.16)\times10^{6}$	Competition kinetics	Rose Bengal	Furfural ($k_{Furfural,\ ^1O_2} = 8.4\times10^{4}\ M^{-1}\ s^{-1}$)	H_2O	5
An and An 2016	Acyclovir	$3.50(\pm0.05)\times10^{6}$	Competition kinetics	Rose Bengal	Furfural ($k_{Furfural,\ ^1O_2} = 8.4\times10^{4}\ M^{-1}\ s^{-1}$)	H_2O	7
An and An 2016	Acyclovir	$1.60(\pm0.13)\times10^{7}$	Competition kinetics	Rose Bengal	Furfural ($k_{Furfural,\ ^1O_2} = 8.4\times10^{4}\ M^{-1}\ s^{-1}$)	H_2O	9
An and An 2016	Acyclovir	$1.58(\pm0.20)\times10^{7}$	Competition kinetics	Rose Bengal	Furfural ($k_{Furfural,\ ^1O_2} = 8.4\times10^{4}\ M^{-1}\ s^{-1}$)	H_2O	10
An and An 2016	Penciclovir	$2.60(\pm0.05)\times10^{6}$	Competition kinetics	Rose Bengal	Furfural ($k_{Furfural,\ ^1O_2} = 8.4\times10^{4}\ M^{-1}\ s^{-1}$)	H_2O	5
An and An 2016	Penciclovir	$3.95(\pm0.22)\times10^{6}$	Competition kinetics	Rose Bengal	Furfural ($k_{Furfural,\ ^1O_2} = 8.4\times10^{4}\ M^{-1}\ s^{-1}$)	H_2O	7
An and An 2016	Penciclovir	$1.51(\pm0.15)\times10^{7}$	Competition kinetics	Rose Bengal	Furfural ($k_{Furfural,\ ^1O_2} = 8.4\times10^{4}\ M^{-1}\ s^{-1}$)	H_2O	9
An and An 2016	Penciclovir	$2.52(\pm0.30)\times10^{7}$	Competition kinetics	Rose Bengal	Furfural ($k_{Furfural,\ ^1O_2} = 8.4\times10^{4}\ M^{-1}\ s^{-1}$)	H_2O	10

Table S17. Summary of $k_{tot, ^1O_2}$ and $k_{rxn, ^1O_2}$ (continued)

Reference	Compound	$k_{tot, ^1O_2}$ or $k_{rxn, ^1O_2}$ (M^{-1} s^{-1})	Method	Sensitizer	Reference Compound	Solvent	pH
An and An 2016	Acyclovir	2.70(±0.05)×10^6	Competition kinetics	Molybdate+H_2O_2	Furfural ($k_{Furfural, ^1O_2}$ = 8.4×10^4 M^{-1} s^{-1})	H_2O	7
An and An 2016	Acyclovir	8.63(±0.38)×10^6	Competition kinetics	Molybdate+H_2O_2	Furfural ($k_{Furfural, ^1O_2}$ = 8.4×10^4 M^{-1} s^{-1})	H_2O	9
An and An 2016	Acyclovir	1.13(±0.10)×10^7	Competition kinetics	Molybdate+H_2O_2	Furfural ($k_{Furfural, ^1O_2}$ = 8.4×10^4 M^{-1} s^{-1})	H_2O	10
An and An 2016	Penciclovir	5.50(±0.10)×10^5	Competition kinetics	Molybdate+H_2O_2	Furfural ($k_{Furfural, ^1O_2}$ = 8.4×10^4 M^{-1} s^{-1})	H_2O	7
An and An 2016	Penciclovir	1.76(±0.22)×10^7	Competition kinetics	Molybdate+H_2O_2	Furfural ($k_{Furfural, ^1O_2}$ = 8.4×10^4 M^{-1} s^{-1})	H_2O	9
An and An 2016	Penciclovir	1.83(±0.26)×10^7	Competition kinetics	Molybdate+H_2O_2	Furfural ($k_{Furfural, ^1O_2}$ = 8.4×10^4 M^{-1} s^{-1})	H_2O	10
Ren and Pan 2016 [101]	Ethinyl Estradiol (17α-Ethynylestradiol)	9.71×10^7	Competition kinetics	Rose Bengal	FFA ($k_{FFA, ^1O_2}$ = 1.2×10^8 M^{-1} s^{-1})	H_2O	8
Chu and McNeill 2016 [102]	Cysteine	2.30(±0.10)×10^8	Kinetic modeling	Perinaphthenone	NA	D_2O	10
Chu and McNeill 2016	Cysteine	1.50(±0.10)×10^8	Time-resolved phosphorescence	Perinaphthenone	NA	D_2O	10.2
Lundeen and McNeill 2016 [103]	Bacitracin A	6.15(±0.42)×10^7	Kinetic modeling	Lumichrome	NA	H_2O	7.9
Lundeen and McNeill 2016	Bacitracin A	5.17(±0.16)×10^7	Kinetic modeling	Rose Bengal	NA	H_2O	8.0
Avetta and Vione 2016 [104]	Diclofenac	1.30(±0.20)×10^7	Kinetic modeling	Rose Bengal	NA	H_2O	6.5
Avetta and Vione 2016	Clofibric Acid	6.00(±1.90)×10^5	Kinetic modeling	Rose Bengal	NA	H_2O	6.5
Li and Song 2016 [105]	Streptomycin	1.58(±0.09)×10^5	Competition kinetics	Rose Bengal	Furfural ($k_{Furfural, ^1O_2}$ = 8.4×10^4 M^{-1} s^{-1})	H_2O	7
Li and Song 2016	Kanamycin	6.84(±0.51)×10^5	Competition kinetics	Rose Bengal	Furfural ($k_{Furfural, ^1O_2}$ = 8.4×10^4 M^{-1} s^{-1})	H_2O	7
Li and Song 2016	Gentamicin	2.62(±0.26)×10^5	Competition kinetics	Rose Bengal	Furfural ($k_{Furfural, ^1O_2}$ = 8.4×10^4 M^{-1} s^{-1})	H_2O	7
Li and Song 2016	Tobramycin	1.35(±0.23)×10^5	Competition kinetics	Rose Bengal	Furfural ($k_{Furfural, ^1O_2}$ = 8.4×10^4 M^{-1} s^{-1})	H_2O	7
Li and Song 2016	Amikacin	8.10(±0.62)×10^5	Competition kinetics	Rose Bengal	Furfural ($k_{Furfural, ^1O_2}$ = 8.4×10^4 M^{-1} s^{-1})	H_2O	7
Santoke and Cooper 2017 [106]	Danofloxacin	7.50(±0.13)×10^4	Competition kinetics	Rose Bengal	Furfural ($k_{Furfural, ^1O_2}$ = 8.4×10^4 M^{-1} s^{-1})	H_2O	NA
Santoke and Cooper 2017	Fluvastatin	1.64(±0.18)×10^8	Competition kinetics	Rose Bengal	FFA ($k_{FFA, ^1O_2}$ = 1.2×10^8 M^{-1} s^{-1})	H_2O	NA
Santoke and Cooper 2017	Paroxetine	1.18(±0.13)×10^8	Competition kinetics	Rose Bengal	FFA ($k_{FFA, ^1O_2}$ = 1.2×10^8 M^{-1} s^{-1})	H_2O	NA
Davis and Janssen 2017 [107]	Mefenamic Acid	1.60(±0.70)×10^7	Kinetic solvent isotope effect	Perinaphthenone	NA	H_2O/D_2O	7.5
Davis and Janssen 2017	Tolfenamic Acid	1.30(±0.60)×10^7	Kinetic solvent isotope effect	Perinaphthenone	NA	H_2O/D_2O	7.5
Davis and Janssen 2017	Meclofenamic Acid	2.80(±0.60)×10^7	Kinetic solvent isotope effect	Perinaphthenone	NA	H_2O/D_2O	7.5
Davis and Janssen 2017	Flufenamic Acid	1.30(±0.20)×10^7	Kinetic solvent isotope effect	Perinaphthenone	NA	H_2O/D_2O	7.5
McConville and Remucal 2017 [108]	Niclosamide	2.25(±0.50)×10^7	Quencher experiments	NA	NA	H_2O	8
Li and Song 2017 [109]	Acetaminophen	3.35×10^5	Competition kinetics	Rose Bengal	Furfural ($k_{Furfural, ^1O_2}$ = 8.4×10^4 M^{-1} s^{-1})	H_2O	7
Li and Song 2017	Acetaminophen	2.26×10^6	Competition kinetics	Rose Bengal	Furfural ($k_{Furfural, ^1O_2}$ = 8.4×10^4 M^{-1} s^{-1})	H_2O	10
Carena and Vione 2017 [110]	Propanil	7.10(±1.80)×10^4	Kinetic modeling	Rose Bengal	NA	H_2O	6.7
Wang and Chen 2019 [111]	2,4-Dihydroxybenzophenone	9.20×10^6	Competition kinetics	Rose Bengal	FFA ($k_{FFA, ^1O_2}$ = 1.2×10^8 M^{-1} s^{-1})	H_2O	8
Chen and Chen 2019 [112]	2-(2-Hydroxy-5-methylphenyl)benzotriazole	8.65(±0.40)×10^7	Competition kinetics	Rose Bengal	FFA ($k_{FFA, ^1O_2}$ = 1.2×10^8 M^{-1} s^{-1})	H_2O	8
Apell and McNeill 2019 [113]	Fludioxonil	4.90(±0.20)×10^7	Competition kinetics	Perinaphthenone	FFA ($k_{FFA, ^1O_2}$ = 1.0×10^8 M^{-1} s^{-1})	H_2O	7
Apell and McNeill 2019	Pyrrole	4.95(±0.20)×10^8	Competition kinetics	Perinaphthenone	FFA ($k_{FFA, ^1O_2}$ = 1.0×10^8 M^{-1} s^{-1})	H_2O	7
Apell and McNeill 2019	3-Phenylpyrrole	7.90(±0.33)×10^8	Competition kinetics	Perinaphthenone	FFA ($k_{FFA, ^1O_2}$ = 1.0×10^8 M^{-1} s^{-1})	H_2O	7
Apell and McNeill 2019	3-Cyanopyrrole	4.38(±0.18)×10^7	Competition kinetics	Perinaphthenone	FFA ($k_{FFA, ^1O_2}$ = 1.0×10^8 M^{-1} s^{-1})	H_2O	7
Ge and Halsall 2019 [114]	Sulfamethoxazole	2.31(±0.06)×10^7	Competition kinetics	Rose Bengal	FFA ($k_{FFA, ^1O_2}$ = 1.2×10^8 M^{-1} s^{-1})	H_2O	6.5
Ge and Halsall 2019	Sulfamethoxazole	2.91(±0.10)×10^7	Competition kinetics	Rose Bengal	FFA ($k_{FFA, ^1O_2}$ = 1.2×10^8 M^{-1} s^{-1})	H_2O	2
Ge and Halsall 2019	Sulfamethoxazole	2.52(±0.28)×10^7	Competition kinetics	Rose Bengal	FFA ($k_{FFA, ^1O_2}$ = 1.2×10^8 M^{-1} s^{-1})	H_2O	5
Ge and Halsall 2019	Sulfamethoxazole	1.90(±0.03)×10^8	Competition kinetics	Rose Bengal	FFA ($k_{FFA, ^1O_2}$ = 1.2×10^8 M^{-1} s^{-1})	H_2O	8
Ge and Halsall 2019	Sulfisoxazole	1.09(±0.03)×10^9	Competition kinetics	Rose Bengal	FFA ($k_{FFA, ^1O_2}$ = 1.2×10^8 M^{-1} s^{-1})	H_2O	6.5
Ge and Halsall 2019	Sulfisoxazole	1.70(±0.04)×10^9	Competition kinetics	Rose Bengal	FFA ($k_{FFA, ^1O_2}$ = 1.2×10^8 M^{-1} s^{-1})	H_2O	2
Ge and Halsall 2019	Sulfisoxazole	1.30(±0.08)×10^9	Competition kinetics	Rose Bengal	FFA ($k_{FFA, ^1O_2}$ = 1.2×10^8 M^{-1} s^{-1})	H_2O	5
Ge and Halsall 2019	Sulfisoxazole	7.19(±0.20)×10^8	Competition kinetics	Rose Bengal	FFA ($k_{FFA, ^1O_2}$ = 1.2×10^8 M^{-1} s^{-1})	H_2O	8
Ge and Halsall 2019	Sulfamethizole	3.83(±0.05)×10^7	Competition kinetics	Rose Bengal	FFA ($k_{FFA, ^1O_2}$ = 1.2×10^8 M^{-1} s^{-1})	H_2O	6.5
Ge and Halsall 2019	Sulfamethizole	3.96(±0.51)×10^7	Competition kinetics	Rose Bengal	FFA ($k_{FFA, ^1O_2}$ = 1.2×10^8 M^{-1} s^{-1})	H_2O	2
Ge and Halsall 2019	Sulfamethizole	4.60(±0.08)×10^7	Competition kinetics	Rose Bengal	FFA ($k_{FFA, ^1O_2}$ = 1.2×10^8 M^{-1} s^{-1})	H_2O	5
Ge and Halsall 2019	Sulfamethizole	1.62(±0.04)×10^8	Competition kinetics	Rose Bengal	FFA ($k_{FFA, ^1O_2}$ = 1.2×10^8 M^{-1} s^{-1})	H_2O	8
Ge and Halsall 2019	Sulfathiazole	1.84(±0.02)×10^8	Competition kinetics	Rose Bengal	FFA ($k_{FFA, ^1O_2}$ = 1.2×10^8 M^{-1} s^{-1})	H_2O	6.5
Ge and Halsall 2019	Sulfathiazole	7.57(±0.21)×10^7	Competition kinetics	Rose Bengal	FFA ($k_{FFA, ^1O_2}$ = 1.2×10^8 M^{-1} s^{-1})	H_2O	2
Ge and Halsall 2019	Sulfathiazole	1.87(±0.03)×10^8	Competition kinetics	Rose Bengal	FFA ($k_{FFA, ^1O_2}$ = 1.2×10^8 M^{-1} s^{-1})	H_2O	5
Ge and Halsall 2019	Sulfathiazole	2.31(±0.04)×10^8	Competition kinetics	Rose Bengal	FFA ($k_{FFA, ^1O_2}$ = 1.2×10^8 M^{-1} s^{-1})	H_2O	8
Ge and Halsall 2019	Sulfamethazine	3.39(±0.17)×10^8	Competition kinetics	Rose Bengal	FFA ($k_{FFA, ^1O_2}$ = 1.2×10^8 M^{-1} s^{-1})	H_2O	6.5
Ge and Halsall 2019	Sulfamethazine	3.47(±0.07)×10^7	Competition kinetics	Rose Bengal	FFA ($k_{FFA, ^1O_2}$ = 1.2×10^8 M^{-1} s^{-1})	H_2O	2
Ge and Halsall 2019	Sulfamethazine	1.53(±0.01)×10^8	Competition kinetics	Rose Bengal	FFA ($k_{FFA, ^1O_2}$ = 1.2×10^8 M^{-1} s^{-1})	H_2O	5

Table S17. Summary of $k_{tot, {}^1O_2}$ and $k_{rxn, {}^1O_2}$ (continued)

Reference	Compound	$k_{tot, {}^1O_2}$ or $k_{rxn, {}^1O_2}$ (M^{-1} s^{-1})	Method	Sensitizer	Reference Compound	Solvent	pH
Ge and Halsall 2019	Sulfamethazine	$1.72(\pm0.08)\times10^9$	Competition kinetics	Rose Bengal	FFA ($k_{FFA, {}^1O_2} = 1.2\times10^8\ M^{-1}\ s^{-1}$)	H_2O	8
Ge and Halsall 2019	Sulfamerazine	$1.81(\pm0.18)\times10^8$	Competition kinetics	Rose Bengal	FFA ($k_{FFA, {}^1O_2} = 1.2\times10^8\ M^{-1}\ s^{-1}$)	H_2O	6.5
Ge and Halsall 2019	Sulfamerazine	$8.25(\pm0.64)\times10^7$	Competition kinetics	Rose Bengal	FFA ($k_{FFA, {}^1O_2} = 1.2\times10^8\ M^{-1}\ s^{-1}$)	H_2O	2
Ge and Halsall 2019	Sulfamerazine	$1.24(\pm0.03)\times10^8$	Competition kinetics	Rose Bengal	FFA ($k_{FFA, {}^1O_2} = 1.2\times10^8\ M^{-1}\ s^{-1}$)	H_2O	5
Ge and Halsall 2019	Sulfamerazine	$5.39(\pm0.28)\times10^8$	Competition kinetics	Rose Bengal	FFA ($k_{FFA, {}^1O_2} = 1.2\times10^8\ M^{-1}\ s^{-1}$)	H_2O	8
Ge and Halsall 2019	Sulfadiazine	$2.02(\pm0.01)\times10^8$	Competition kinetics	Rose Bengal	FFA ($k_{FFA, {}^1O_2} = 1.2\times10^8\ M^{-1}\ s^{-1}$)	H_2O	6.5
Ge and Halsall 2019	Sulfadiazine	$1.16(\pm0.03)\times10^8$	Competition kinetics	Rose Bengal	FFA ($k_{FFA, {}^1O_2} = 1.2\times10^8\ M^{-1}\ s^{-1}$)	H_2O	2
Ge and Halsall 2019	Sulfadiazine	$1.75(\pm0.07)\times10^8$	Competition kinetics	Rose Bengal	FFA ($k_{FFA, {}^1O_2} = 1.2\times10^8\ M^{-1}\ s^{-1}$)	H_2O	5
Ge and Halsall 2019	Sulfadiazine	$4.62(\pm0.02)\times10^8$	Competition kinetics	Rose Bengal	FFA ($k_{FFA, {}^1O_2} = 1.2\times10^8\ M^{-1}\ s^{-1}$)	H_2O	8
Ge and Halsall 2019	Sulfachlorpyridazine	$1.34(\pm0.15)\times10^9$	Competition kinetics	Rose Bengal	FFA ($k_{FFA, {}^1O_2} = 1.2\times10^8\ M^{-1}\ s^{-1}$)	H_2O	6.5
Ge and Halsall 2019	Sulfachlorpyridazine	$8.27(\pm0.15)\times10^8$	Competition kinetics	Rose Bengal	FFA ($k_{FFA, {}^1O_2} = 1.2\times10^8\ M^{-1}\ s^{-1}$)	H_2O	2
Ge and Halsall 2019	Sulfachlorpyridazine	$3.74(\pm0.04)\times10^9$	Competition kinetics	Rose Bengal	FFA ($k_{FFA, {}^1O_2} = 1.2\times10^8\ M^{-1}\ s^{-1}$)	H_2O	5
Ge and Halsall 2019	Sulfachlorpyridazine	$9.24(\pm0.59)\times10^8$	Competition kinetics	Rose Bengal	FFA ($k_{FFA, {}^1O_2} = 1.2\times10^8\ M^{-1}\ s^{-1}$)	H_2O	8
Ge and Halsall 2019	Sulfadimethoxine	$4.83(\pm0.13)\times10^7$	Competition kinetics	Rose Bengal	FFA ($k_{FFA, {}^1O_2} = 1.2\times10^8\ M^{-1}\ s^{-1}$)	H_2O	6.5
Ge and Halsall 2019	Sulfadimethoxine	$3.51(\pm0.23)\times10^7$	Competition kinetics	Rose Bengal	FFA ($k_{FFA, {}^1O_2} = 1.2\times10^8\ M^{-1}\ s^{-1}$)	H_2O	2
Ge and Halsall 2019	Sulfadimethoxine	$4.08(\pm0.05)\times10^7$	Competition kinetics	Rose Bengal	FFA ($k_{FFA, {}^1O_2} = 1.2\times10^8\ M^{-1}\ s^{-1}$)	H_2O	5
Ge and Halsall 2019	Sulfadimethoxine	$1.55(\pm0.06)\times10^8$	Competition kinetics	Rose Bengal	FFA ($k_{FFA, {}^1O_2} = 1.2\times10^8\ M^{-1}\ s^{-1}$)	H_2O	8
Jaramillo and O'Shea 2020 [115]	Domoic Acid	5.10×10^5	Competition kinetics	Rose Bengal	FFA ($k_{FFA, {}^1O_2} = 1.0\times10^8\ M^{-1}\ s^{-1}$)	H_2O	8
Pozdnyakov and Chen 2020 [116]	Imipramine	$1.10(\pm0.10)\times10^8$	Time-resolved phosphorescence	Meso-tetra-(N-methyl-4-pyridyl) porphyrin	NA	D_2O	NA
Pozdnyakov and Parkhats 2020 [117]	Dicamba	$5.30(\pm2.90)\times10^8$	Time-resolved phosphorescence	Meso-tetra-(N-methyl-4-pyridyl) porphyrin	NA	D_2O	NA
Sunday and Sakugawa 2020 [118]	Diazinon	$7.29(\pm0.30)\times10^4$	Competition kinetics	Rose Bengal	FFA ($k_{FFA, {}^1O_2} = 1.09\times10^8\ M^{-1}\ s^{-1}$)	H_2O	NA
Sunday and Sakugawa 2020	Methylmercury	1.90×10^6	Competition kinetics	Rose Bengal	FFA ($k_{FFA, {}^1O_2} = 1.09\times10^8\ M^{-1}\ s^{-1}$)	H_2O	NA
Sunday and Sakugawa 2020	Tetrabromobisphenol A (TBBPA)	3.90×10^8	Competition kinetics	Rose Bengal	FFA ($k_{FFA, {}^1O_2} = 1.09\times10^8\ M^{-1}\ s^{-1}$)	H_2O	NA
Davis and Janssen 2020 [119]	Daptomycin	$1.30(\pm0.20)\times10^7$	Kinetic solvent isotope effect	Perinaphthenone	NA	H_2O/D_2O	7.5
Davis and Janssen 2020	Polymyxin B1	$1.02(\pm0.25)\times10^8$	Kinetic solvent isotope effect	Perinaphthenone	NA	H_2O/D_2O	7.5
Davis and Janssen 2020	Polymyxin B2	$1.02(\pm0.25)\times10^8$	Kinetic solvent isotope effect	Perinaphthenone	NA	H_2O/D_2O	7.5
Davis and Janssen 2020	Polymyxin E1	$1.05(\pm0.18)\times10^8$	Kinetic solvent isotope effect	Perinaphthenone	NA	H_2O/D_2O	7.5
Davis and Janssen 2020	Polymyxin E2	$1.47(\pm0.21)\times10^8$	Kinetic solvent isotope effect	Perinaphthenone	NA	H_2O/D_2O	7.5
Natumi and Janssen 2021 [120]	Tyrosine	$1.32(\pm0.05)\times10^8$	Kinetic solvent isotope effect	Perinaphthenone	NA	H_2O/D_2O	11.6
Natumi and Janssen 2021	Anabaenopeptin B	$9.65(\pm0.59)\times10^7$	Kinetic solvent isotope effect	Perinaphthenone	NA	H_2O/D_2O	11.6
Natumi and Janssen 2021	Anabaenopeptin F	$9.10(\pm0.59)\times10^7$	Kinetic solvent isotope effect	Perinaphthenone	NA	H_2O/D_2O	11.6
Natumi and Janssen 2021	Lyngbyaureidamide B	$1.03(\pm0.38)\times10^8$	Kinetic solvent isotope effect	Perinaphthenone	NA	H_2O/D_2O	11.6
Natumi and Janssen 2021	Microcystin-HtyR	$9.64(\pm1.56)\times10^7$	Kinetic solvent isotope effect	Perinaphthenone	NA	H_2O/D_2O	11.6
Natumi and Janssen 2021	Microcystin-YR	$1.02(\pm0.27)\times10^8$	Kinetic solvent isotope effect	Perinaphthenone	NA	H_2O/D_2O	11.6
Natumi and Janssen 2021	Anabaenopeptin C	$1.45(\pm0.17)\times10^8$	Kinetic solvent isotope effect	Perinaphthenone	NA	H_2O/D_2O	11.6
Natumi and Janssen 2021	Anabaenopeptin A	$1.64(\pm0.20)\times10^8$	Kinetic solvent isotope effect	Perinaphthenone	NA	H_2O/D_2O	11.6
Natumi and Janssen 2021	Oscillamide Y	$2.09(\pm0.15)\times10^8$	Kinetic solvent isotope effect	Perinaphthenone	NA	H_2O/D_2O	11.6
Natumi and Janssen 2021	Anabaenopeptin 871	$3.83(\pm0.84)\times10^8$	Kinetic solvent isotope effect	Perinaphthenone	NA	H_2O/D_2O	11.6
Cai and Bollmann 2021 [121]	Medetomidine	$1.80(\pm0.02)\times10^8$	Competition kinetics	Zinc Porphyrin	FFA ($k_{FFA, {}^1O_2} = 1\times10^8\ M^{-1}\ s^{-1}$)	H_2O	9.2
Cai and Bollmann	Medetomidine	$1.80(\pm0.02)\times10^8$	Competition kinetics	Zinc Porphyrin	FFA ($k_{FFA, {}^1O_2} = 1\times10^8\ M^{-1}\ s^{-1}$)	H_2O	10.5
Jia and Song 2021 [122]	Erythromycin	$1.23(\pm0.04)\times10^5$	Competition kinetics	Rose Bengal	Furfural ($k_{Furfural, {}^1O_2} = 8.4\times10^4\ M^{-1}\ s^{-1}$)	H_2O	7
Jia and Song	Roxithromycin	$1.41(\pm0.03)\times10^5$	Competition kinetics	Rose Bengal	Furfural ($k_{Furfural, {}^1O_2} = 8.4\times10^4\ M^{-1}\ s^{-1}$)	H_2O	7
Jia and Song	Clarithromycin	$1.22(\pm0.04)\times10^5$	Competition kinetics	Rose Bengal	Furfural ($k_{Furfural, {}^1O_2} = 8.4\times10^4\ M^{-1}\ s^{-1}$)	H_2O	7
Jia and Song	Azithromycin	$2.42(\pm0.09)\times10^5$	Competition kinetics	Rose Bengal	Furfural ($k_{Furfural, {}^1O_2} = 8.4\times10^4\ M^{-1}\ s^{-1}$)	H_2O	7
Lian and Yan 2021 [123]	Estradiol (17β-Estradiol)	1.39×10^7	Competition kinetics	Rose Bengal	Furfural ($k_{Furfural, {}^1O_2} = 8.4\times10^4\ M^{-1}\ s^{-1}$)	H_2O	7
Lian and Yan 2021	Estradiol (17β-Estradiol)	1.67×10^7	Competition kinetics	Rose Bengal	Furfural ($k_{Furfural, {}^1O_2} = 8.4\times10^4\ M^{-1}\ s^{-1}$)	H_2O	8
Lian and Yan 2021	Estradiol (17β-Estradiol)	1.07×10^8	Competition kinetics	Rose Bengal	Furfural ($k_{Furfural, {}^1O_2} = 8.4\times10^4\ M^{-1}\ s^{-1}$)	H_2O	10
Gornik and Vione 2021 [124]	Paroxetine	$1.26(\pm0.20)\times10^8$	Kinetic modeling	Rose Bengal	NA	H_2O	7
Wan and Chen 2022 [125]	Cimetidine	$2.70(\pm0.20)\times10^8$	Competition kinetics	Rose Bengal	FFA ($k_{FFA, {}^1O_2} = 1\times10^8\ M^{-1}\ s^{-1}$)	H_2O	8
Norris and Rosario-Ortiz 2024 [126]	Saxitoxin	1.27×10^7	Competition kinetics	Rose Bengal	FFA ($k_{FFA, {}^1O_2} = 1\times10^8\ M^{-1}\ s^{-1}$)	H_2O	8
Wang and Zhang 2024 [127]	Pindolol	6.40×10^7	Competition kinetics	Rose Bengal	FFA ($k_{FFA, {}^1O_2} = 1.4\times10^8\ M^{-1}\ s^{-1}$)	H_2O	8
Lee and Sáez 2025 [128]	Dexamethasone	$1.20(\pm0.09)\times10^6$	Kinetic solvent isotope effect	Perinaphthenone	NA	H_2O/D_2O	7.5
Lee and Sáez 2025	Fluoxetine	$1.44(\pm0.14)\times10^8$	Kinetic solvent isotope effect	Perinaphthenone	NA	H_2O/D_2O	7.5
Lee and Sáez 2025	Primidone	$3.18(\pm0.44)\times10^6$	Kinetic solvent isotope effect	Perinaphthenone	NA	H_2O/D_2O	7.5

Table S17. Summary of $k_{tot, ^1O_2}$ and $k_{rxn, ^1O_2}$ (continued)

Reference	Compound	$k_{tot, ^1O_2}$ or $k_{rxn, ^1O_2}$ (M^{-1} s^{-1})	Method	Sensitizer	Reference Compound	Solvent	pH
Zong and Zhang 2025 [129]	Metronidazole	2.79×10^5	Competition kinetics	Rose Bengal	FFA ($k_{FFA, ^1O_2} = 1.2\times10^8$ M^{-1} s^{-1})	H_2O	7.0
Zong and Zhang 2025	Ornidazole	5.51×10^5	Competition kinetics	Rose Bengal	FFA ($k_{FFA, ^1O_2} = 1.2\times10^8$ M^{-1} s^{-1})	H_2O	7.0
Zong and Zhang 2025	Norfloxacin	4.30×10^5	Competition kinetics	Rose Bengal	FFA ($k_{FFA, ^1O_2} = 1.2\times10^8$ M^{-1} s^{-1})	H_2O	7.0
Zong and Zhang 2025	Tinidazole	3.16×10^6	Competition kinetics	Rose Bengal	FFA ($k_{FFA, ^1O_2} = 1.2\times10^8$ M^{-1} s^{-1})	H_2O	7.0
Zong and Zhang 2025	Ciprofloxacin	1.18×10^7	Competition kinetics	Rose Bengal	FFA ($k_{FFA, ^1O_2} = 1.2\times10^8$ M^{-1} s^{-1})	H_2O	7.0
Zong and Zhang 2025	Metronidazole	2.25×10^5	Competition kinetics	Rose Bengal	FFA ($k_{FFA, ^1O_2} = 1.2\times10^8$ M^{-1} s^{-1})	H_2O (estuarine water)	7.6
Zong and Zhang 2025	Ornidazole	5.34×10^5	Competition kinetics	Rose Bengal	FFA ($k_{FFA, ^1O_2} = 1.2\times10^8$ M^{-1} s^{-1})	H_2O (estuarine water)	7.6
Zong and Zhang 2025	Norfloxacin	1.50×10^5	Competition kinetics	Rose Bengal	FFA ($k_{FFA, ^1O_2} = 1.2\times10^8$ M^{-1} s^{-1})	H_2O (estuarine water)	7.6
Zong and Zhang 2025	Tinidazole	5.80×10^6	Competition kinetics	Rose Bengal	FFA ($k_{FFA, ^1O_2} = 1.2\times10^8$ M^{-1} s^{-1})	H_2O (estuarine water)	7.6
Zong and Zhang 2025	Ciprofloxacin	5.04×10^6	Competition kinetics	Rose Bengal	FFA ($k_{FFA, ^1O_2} = 1.2\times10^8$ M^{-1} s^{-1})	H_2O (estuarine water)	7.6
Zong and Zhang 2025	Metronidazole	4.91×10^5	Competition kinetics	Rose Bengal	FFA ($k_{FFA, ^1O_2} = 1.2\times10^8$ M^{-1} s^{-1})	H_2O (seawater)	8.1
Zong and Zhang 2025	Ornidazole	3.38×10^5	Competition kinetics	Rose Bengal	FFA ($k_{FFA, ^1O_2} = 1.2\times10^8$ M^{-1} s^{-1})	H_2O (seawater)	8.1
Zong and Zhang 2025	Norfloxacin	2.75×10^5	Competition kinetics	Rose Bengal	FFA ($k_{FFA, ^1O_2} = 1.2\times10^8$ M^{-1} s^{-1})	H_2O (seawater)	8.1
Zong and Zhang 2025	Tinidazole	3.42×10^6	Competition kinetics	Rose Bengal	FFA ($k_{FFA, ^1O_2} = 1.2\times10^8$ M^{-1} s^{-1})	H_2O (seawater)	8.1
Zong and Zhang 2025	Ciprofloxacin	3.35×10^6	Competition kinetics	Rose Bengal	FFA ($k_{FFA, ^1O_2} = 1.2\times10^8$ M^{-1} s^{-1})	H_2O (seawater)	8.1
Arciva and Anastasio 2025 [130]	2,5-Dimethylfuran (2,5-DMF)	$5.90(\pm0.50)\times10^8$	Competition kinetics	Rose Bengal	FFA ($k_{FFA, ^1O_2} = 9.60(\pm0.04)\times10^7$ M^{-1} s^{-1} in H_2O)	H_2O	5
Arciva and Anastasio 2025	2,3-Dimethylfuran (2,3-DMF)	$4.60(\pm0.40)\times10^8$	Competition kinetics	Rose Bengal	FFA ($k_{FFA, ^1O_2} = 9.60\times10^7$ M^{-1} s^{-1})	H_2O	5
Arciva and Anastasio 2025	2-Methylfuran (2-MF)	$3.20(\pm0.80)\times10^8$	Competition kinetics	Rose Bengal	FFA ($k_{FFA, ^1O_2} = 9.60\times10^7$ M^{-1} s^{-1})	H_2O	5
Arciva and Anastasio 2025	2-Methyl-furanhydrazide	$1.50(\pm0.04)\times10^8$	Competition kinetics	Rose Bengal	FFA ($k_{FFA, ^1O_2} = 9.60\times10^7$ M^{-1} s^{-1})	H_2O	5
Arciva and Anastasio 2025	2,5-Bis(hydroxymethyl)furan (BHMF)	$1.04(\pm0.02)\times10^8$	Competition kinetics	Rose Bengal	FFA ($k_{FFA, ^1O_2} = 9.60\times10^7$ M^{-1} s^{-1})	H_2O	5
Arciva and Anastasio 2025	Furan	$8.30(\pm0.50)\times10^7$	Competition kinetics	Rose Bengal	FFA ($k_{FFA, ^1O_2} = 9.60\times10^7$ M^{-1} s^{-1})	H_2O	5
Arciva and Anastasio 2025	Furoic Acid	$4.20(\pm0.20)\times10^7$	Competition kinetics	Rose Bengal	FFA ($k_{FFA, ^1O_2} = 9.60\times10^7$ M^{-1} s^{-1})	H_2O	5.1
Arciva and Anastasio 2025	Furfurylamine (FFAm)	$3.90(\pm1.30)\times10^7$	Competition kinetics	Rose Bengal	FFA ($k_{FFA, ^1O_2} = 9.60\times10^7$ M^{-1} s^{-1})	H_2O	5
Arciva and Anastasio 2025	2-Methylfuran-3,4-dicarboxylic Acid (MFDCA)	$7.60(\pm1.10)\times10^6$	Competition kinetics	Rose Bengal	FFA ($k_{FFA, ^1O_2} = 9.60\times10^7$ M^{-1} s^{-1})	H_2O	2.5
Arciva and Anastasio 2025	Furoic Acid	$3.80(\pm0.30)\times10^6$	Competition kinetics	Rose Bengal	FFA ($k_{FFA, ^1O_2} = 9.60\times10^7$ M^{-1} s^{-1})	H_2O	2
Arciva and Anastasio 2025	5-Nitro-2-furaldehyde	$1.50(\pm0.30)\times10^6$	Competition kinetics	Rose Bengal	FFA ($k_{FFA, ^1O_2} = 9.60\times10^7$ M^{-1} s^{-1})	H_2O	5
Arciva and Anastasio 2025	5-Hydroxymethyl-2-furaldehyde (HMF)	$1.20(\pm0.10)\times10^6$	Competition kinetics	Rose Bengal	FFA ($k_{FFA, ^1O_2} = 9.60\times10^7$ M^{-1} s^{-1})	H_2O	5
Arciva and Anastasio 2025	Furfural	$6.30(\pm0.40)\times10^5$	Competition kinetics	Rose Bengal	FFA ($k_{FFA, ^1O_2} = 9.60\times10^7$ M^{-1} s^{-1})	H_2O	5
Arciva and Anastasio 2025	5-Iodo-2-furaldehyde	$4.00(\pm0.30)\times10^5$	Competition kinetics	Rose Bengal	FFA ($k_{FFA, ^1O_2} = 9.60\times10^7$ M^{-1} s^{-1})	H_2O	5
Arciva and Anastasio 2025	5-Chloro-2-furaldehyde	$3.60(\pm0.50)\times10^5$	Competition kinetics	Rose Bengal	FFA ($k_{FFA, ^1O_2} = 9.60\times10^7$ M^{-1} s^{-1})	H_2O	5
Arciva and Anastasio 2025	5-Bromo-2-furaldehyde	$3.40(\pm0.80)\times10^5$	Competition kinetics	Rose Bengal	FFA ($k_{FFA, ^1O_2} = 9.60\times10^7$ M^{-1} s^{-1})	H_2O	5
Arciva and Anastasio 2025	5-Hydroxymethyl-2-furanone	$0.30(\pm2.10)\times10^4$	Competition kinetics	Rose Bengal	FFA ($k_{FFA, ^1O_2} = 9.60\times10^7$ M^{-1} s^{-1})	H_2O	5
de Brito Anton and Apell 2025 [131]	Chlorpyrifos	$1.47(\pm0.12)\times10^6$	Kinetic solvent isotope effect	Zinc Porphyrin	NA	H_2O/D_2O	7
de Brito Anton and Apell 2025	Cyprodinil	$1.38(\pm0.08)\times10^6$	Kinetic solvent isotope effect	Zinc Porphyrin	NA	H_2O/D_2O	7
de Brito Anton and Apell 2025	Dicamba	$7.55(\pm0.61)\times10^5$	Kinetic solvent isotope effect	Zinc Porphyrin	NA	H_2O/D_2O	7
de Brito Anton and Apell 2025	Fluroxypyr	$3.59(\pm0.23)\times10^5$	Kinetic solvent isotope effect	Zinc Porphyrin	NA	H_2O/D_2O	7
de Brito Anton and Apell 2025	Fomesafen	$4.53(\pm0.31)\times10^5$	Kinetic solvent isotope effect	Zinc Porphyrin	NA	H_2O/D_2O	7
de Brito Anton and Apell 2025	(4-Chloro-2-methylphenoxy)acetic Acid (MCPA)	$8.52(\pm0.51)\times10^5$	Kinetic solvent isotope effect	Zinc Porphyrin	NA	H_2O/D_2O	7
de Brito Anton and Apell 2025	Mesotrione	$7.54(\pm0.38)\times10^5$	Kinetic solvent isotope effect	Zinc Porphyrin	NA	H_2O/D_2O	7
de Brito Anton and Apell 2025	Prothioconazole	$5.86(\pm0.27)\times10^6$	Kinetic solvent isotope effect	Zinc Porphyrin	NA	H_2O/D_2O	5
de Brito Anton and Apell 2025	Prothioconazole	$9.22(\pm0.40)\times10^7$	Kinetic solvent isotope effect	Zinc Porphyrin	NA	H_2O/D_2O	9
de Brito Anton and Apell 2025	Pyrimethanil	$7.61(\pm0.51)\times10^5$	Kinetic solvent isotope effect	Zinc Porphyrin	NA	H_2O/D_2O	7
de Brito Anton and Apell 2025	Thiobencarb	$8.83(\pm0.61)\times10^5$	Kinetic solvent isotope effect	Zinc Porphyrin	NA	H_2O/D_2O	7
de Brito Anton and Apell 2025	Chlorpyrifos	$1.87(\pm0.13)\times10^6$	Competition kinetics	Perinaphthenone	FFA ($k_{FFA, ^1O_2} = 1\times10^8$ M^{-1} s^{-1})	H_2O	7
de Brito Anton and Apell 2025	Fomesafen	$4.16(\pm0.32)\times10^5$	Competition kinetics	Perinaphthenone	FFA ($k_{FFA, ^1O_2} = 1\times10^8$ M^{-1} s^{-1})	H_2O	7
de Brito Anton and Apell 2025	Prothioconazole	$1.25(\pm0.06)\times10^8$	Competition kinetics	Perinaphthenone	FFA ($k_{FFA, ^1O_2} = 1\times10^8$ M^{-1} s^{-1})	H_2O	9

$k_{tot, ^1O_2}$= total quenching rate constant, which is the sum of both physical quenching and chemical reaction of the compound with $^1O_2$; $k_{rxn, ^1O_2}$ = bimolecular reaction rate constant with $^1O_2$.

19. Literature survey of $\Phi_{app,\ ^1O_2}$ and optical data

**Table S18.** Summary of $\Phi_{app,\ ^1O_2}$ and optical data									
Reference	Sample ID	Sample Classification	*E2:E3*	$SUVA_{254}$ (L mg C^{-1}•m^{-1})	$S_{290–400}$ / $S_{300–400}$ / $S_{290–450}$ / $S_{295–400}$ (μm^{-1})	$S_{300–600}$ / $S_{300–500}$ / $S_{300–700}$ / $S_{275–500}$ (μm^{-1})	FI	Wavelength Range (nm)	$\Phi_{app,\ ^1O_2}$ ($\times 10^{-2}$ mol mol-photons^{-1})
Zepp 1977 [132]	Okefenokee Swamp, Georgia	Whole Water (Wetland)	NA	NA	NA	NA	NA	366	5.60
Zepp 1977	Aucilla River, Lamont, Florida	Whole Water (River)	NA	NA	NA	NA	NA	366	2.60
Zepp 1977	Econfina River near Perry, Florida	Whole Water (River)	NA	NA	NA	NA	NA	366	3.20
Zepp 1977	Fenholloway River, Foley, Florida	Whole Water (River)	NA	NA	NA	NA	NA	366	1.40
Zepp 1977	Wakulla River near Wakulla Springs, Florida	Whole Water (River)	NA	NA	NA	NA	NA	366	6.00
Zepp 1977	St Marks River, St Marks, FL	Whole Water (River)	NA	NA	NA	NA	NA	366	3.00
Zepp 1977	Gulf of Mexico, Shell Point, FL	Whole Water (Estuary)	NA	NA	NA	NA	NA	366	6.00
Zepp 1977	Gulf of Mexico, Live Oak Island, FL	Whole Water (Estuary)	NA	NA	NA	NA	NA	366	5.20
Zepp 1977	Puddle in peanut field, Sylvester, GA	Whole Water (Puddle)	NA	NA	NA	NA	NA	366	6.50
Zepp 1977	Mississippi River, Baton Rouge, Louisiana	Whole Water (River)	NA	NA	NA	NA	NA	366	9.30
Zepp 1977	Hickory Hills Pond, Athens, GA	Whole Water (Pond)	NA	NA	NA	NA	NA	366	1.90
Baxter and Carey 1982 [133]	Wylde Lake	Whole Water (Lake)	NA	NA	NA	NA	NA	365	1.00
Haag and Hoigné 1984 [134]	Fluka Humic Acid 366 nm	XAD Fraction (Soil)	NA	NA	NA	NA	NA	366	0.49
Haag and Hoigné 1984	Fluka Humic Acid 405 nm	XAD Fraction (Soil)	NA	NA	NA	NA	NA	405	0.50
Haag and Hoigné 1984	Fluka Humic Acid 436 nm	XAD Fraction (Soil)	NA	NA	NA	NA	NA	436	0.48
Haag and Hoigné 1984	Fluka Humic Acid 546 nm	XAD Fraction (Soil)	NA	NA	NA	NA	NA	546	0.50
Haag and Hoigné 1984	Black Lake Humic Acid 366 nm	XAD Fraction (Lake)	NA	NA	NA	NA	NA	366	1.00
Haag and Hoigné 1984	Black Lake Humic Acid 405 nm	XAD Fraction (Lake)	NA	NA	NA	NA	NA	405	1.30
Haag and Hoigné 1984	Black Lake Humic Acid 436 nm	XAD Fraction (Lake)	NA	NA	NA	NA	NA	436	1.20
Haag and Hoigné 1984	Black Lake Humic Acid 546 nm	XAD Fraction (Lake)	NA	NA	NA	NA	NA	546	0.51
Haag and Hoigné 1984	Lake Baldegg DOM Concentrate 366 nm	RO Isolate (Lake)	NA	NA	NA	NA	NA	366	2.60
Haag and Hoigné 1984	Lake Baldegg DOM Concentrate 405 nm	RO Isolate (Lake)	NA	NA	NA	NA	NA	405	2.30
Haag and Hoigné 1984	Lake Baldegg DOM Concentrate 436 nm	RO Isolate (Lake)	NA	NA	NA	NA	NA	436	2.00
Haag and Hoigné 1984	Lake Baldegg DOM Concentrate 546 nm	RO Isolate (Lake)	NA	NA	NA	NA	NA	546	1.10
Zepp 1985 [135]	Aucilla River, Lamont, Florida 313 nm	Whole Water (River)	NA	NA	NA	NA	NA	313	2.80
Zepp 1985	Aucilla River, Lamont, Florida 366 nm	Whole Water (River)	NA	NA	NA	NA	NA	366	1.10
Zepp 1985	Aucilla River, Lamont, Florida 405 nm	Whole Water (River)	NA	NA	NA	NA	NA	405	0.60
Zepp 1985	Ogeechee River near Savannah, GA 366 nm	Whole Water (River)	NA	NA	NA	NA	NA	366	1.60
Zepp 1985	Suwannee River, Suwannee Springs, FL 313 nm	Whole Water (River)	NA	NA	NA	NA	NA	313	2.40
Zepp 1985	Suwannee River, Suwannee Springs, FL 366 nm	Whole Water (River)	NA	NA	NA	NA	NA	366	1.70
Zepp 1985	Wylde Lake humus (Ontario) 313 nm	XAD Fraction (Lake)	NA	NA	NA	NA	NA	313	1.60
Zepp 1985	Wylde Lake humus (Ontario) 366 nm	XAD Fraction (Lake)	NA	NA	NA	NA	NA	366	0.60
Zepp 1985	Wylde Lake humus (Ontario) 405 nm	XAD Fraction (Lake)	NA	NA	NA	NA	NA	405	0.30
Zepp 1985	Fluka A.G. humic acid 313 nm	XAD Fraction (Soil)	NA	NA	NA	NA	NA	313	1.10
Zepp 1985	Fluka A.G. humic acid 366 nm	XAD Fraction (Soil)	NA	NA	NA	NA	NA	366	0.40
Zepp 1985	Fluka A.G. humic acid 405 nm	XAD Fraction (Soil)	NA	NA	NA	NA	NA	405	0.40
Zepp 1985	Aldrich humic acid 313 nm	XAD Fraction (Soil)	NA	NA	NA	NA	NA	313	1.30
Zepp 1985	Aldrich humic acid 366 nm	XAD Fraction (Soil)	NA	NA	NA	NA	NA	366	0.40
Zepp 1985	Contech fulvic acid 313 nm	XAD Fraction (Soil)	NA	NA	NA	NA	NA	313	0.40
Zepp 1985	Contech fulvic acid 366 nm	XAD Fraction (Soil)	NA	NA	NA	NA	NA	366	0.50
Frimmel 1987 [136]	BAN 13 (FA) Bansee lake (Germany)	XAD Fraction (Lake)	NA	NA	NA	NA	NA	366	3.00
Frimmel 1987	BAN 13 (FA)	XAD Fraction (Lake)	NA	NA	NA	NA	NA	366	2.10
Frimmel 1987	BAN 13 (FA)	XAD Fraction (Lake)	NA	NA	NA	NA	NA	366	1.50
Frimmel 1987	BAN 13 (HA)	XAD Fraction (Lake)	NA	NA	NA	NA	NA	366	2.00
Frimmel 1987	BM 4 (FA) Brunnensee bog lake (Germany)	XAD Fraction (Lake)	NA	NA	NA	NA	NA	366	1.60
Frimmel 1987	BM 4 (FA/1)	XAD Fraction (Lake)	NA	NA	NA	NA	NA	366	1.60
Frimmel 1987	BM 7 (FA)	XAD Fraction (Lake)	NA	NA	NA	NA	NA	366	1.00
Frimmel 1987	ZIL 1 (FA) Zillhamer See lake (Germany)	XAD Fraction (Lake)	NA	NA	NA	NA	NA	366	2.60

**Table S18.** Summary of $\Phi_{app,\ ^1O_2}$ and optical data (continued)									
Reference	Sample ID	Sample Classification	*E2:E3*	SUVA$_{254}$ (L mg C^{-1}•m^{-1})	$S_{290–400}$ / $S_{300–400}$ / $S_{290–450}$ / $S_{295–400}$ (μm^{-1})	$S_{300–600}$ / $S_{300–500}$ / $S_{300–700}$ / $S_{275–500}$ (μm^{-1})	FI	Wavelength Range (nm)	$\Phi_{app,\ ^1O_2}$ (×10^{-2} mol mol-photons^{-1})
Frimmel 1987	ZIL 1 (FA)	XAD Fraction (Lake)	NA	NA	NA	NA	NA	366	1.70
Frimmel 1987	Suwannee River fulvic acid (SRFA)	IHSS Isolate (SRFA)	NA	NA	NA	NA	NA	366	1.80
Frimmel 1987	Suwannee River fulvic acid (SRFA)	IHSS Isolate (SRFA)	NA	NA	NA	NA	NA	366	1.00
Frimmel 1987	ERD (FA) Soil fulvic acid material (Cultivated Rendzina soil, München-Grosshadern)	XAD Fraction (Soil)	NA	NA	NA	NA	NA	366	3.00
Faust and Allen 1992 [137]	UVA89-1 (Rain sample collected over September 1988, Shenandoah Peak, VA, USA)	Whole Water (Rainwater)	NA	NA	NA	NA	NA	335	4.80
Faust and Allen 1992	SUN-11 (Rain sample collected over August 1988, Whiteface Mountain, NY, USA)	Whole Water (Rainwater)	NA	NA	NA	NA	NA	335	13.00
Faust and Allen 1992	SUN89-1-6 (Rain sample collected over August 1989, Whiteface Mountain, NY, [illegible]	Whole Water (Rainwater)	NA	NA	NA	NA	NA	335	20.00
Shao and Cooper 1994 [138]	Sharpes Bay water sample, Ontario, Canada 280 nm	Whole Water (Lake)	NA	NA	NA	NA	NA	280	3.35
Shao and Cooper 1994	Sharpes Bay water sample, Ontario, Canada 300 nm	Whole Water (Lake)	NA	NA	NA	NA	NA	300	0.59
Shao and Cooper 1994	Sharpes Bay water sample, Ontario, Canada 340 nm	Whole Water (Lake)	NA	NA	NA	NA	NA	340	0.14
Shao and Cooper 1994	Sharpes Bay water sample, Ontario, Canada 360 nm	Whole Water (Lake)	NA	NA	NA	NA	NA	360	0.08
Shao and Cooper 1994	Sharpes Bay water sample, Ontario, Canada 400 nm	Whole Water (Lake)	NA	NA	NA	NA	NA	400	0.24
Aguer and Richard 1997 [139]	Ranker FA	XAD Fraction (Soil)	NA	NA	NA	NA	NA	365	0.55
Aguer and Richard 1997	Ranker HA	XAD Fraction (Soil)	NA	NA	NA	NA	NA	365	0.36
Aguer and Richard 1997	Commercial Aldrich HA	XAD Fraction (Soil)	NA	NA	NA	NA	NA	365	0.20
Aguer and Richard 1997	Rendzina HA	XAD Fraction (Soil)	NA	NA	NA	NA	NA	365	0.11
Aguer and Richard 1997	Podzol A 1 or Bb HA	XAD Fraction (Soil)	NA	NA	NA	NA	NA	365	0.04
Sandvik 2000 [140]	Mississippi 1995 T2C > 3kDa	UF Fraction (River)	NA	NA	16.90	NA	NA	280-380	1.70
Sandvik 2000	Mississippi 1995 T2D> 3kDa	UF Fraction (River)	NA	NA	17.20	NA	NA	280-380	1.70
Sandvik 2000	Mississippi 1995 T2E > 3kDa	UF Fraction (River)	NA	NA	16.90	NA	NA	280-380	1.70
Sandvik 2000	Atchafalaya 1995 T1A > 3kDa	UF Fraction (River)	NA	NA	19.10	NA	NA	280-380	2.70
Sandvik 2000	Atchafalaya 1995 T1B > 3kDa	UF Fraction (River)	NA	NA	17.00	NA	NA	280-380	1.90
Sandvik 2000	Atchafalaya 1995 T1D > 3kDa	UF Fraction (River)	NA	NA	17.70	NA	NA	280-380	2.00
Sandvik 2000	Atchafalaya 1995 T1E > 3kDa	UF Fraction (River)	NA	NA	17.50	NA	NA	280-380	1.60
Sandvik 2000	Gulf of Mexico BGOM > 1kDa	UF Fraction (Estuary)	NA	NA	NA	NA	NA	280-380	3.90
Sandvik 2000	Mississippi River BMISS > 1kDa	UF Fraction (River)	NA	NA	NA	NA	NA	280-380	6.10
Sandvik 2000	Apalachicola River APP > 3kDa	UF Fraction (River)	NA	NA	NA	NA	NA	280-380	1.40
Sandvik 2000	Aldrich Humic Acids ALD	XAD Fraction (Soil)	NA	NA	NA	NA	NA	280-380	1.00
Sandvik 2000	Mississippi 1996 MD > 3kDa	UF Fraction (River)	NA	NA	17.60	NA	NA	280-380	2.00
Sandvik 2000	Mississippi 1996 MC > 3kDa	UF Fraction (River)	NA	NA	17.70	NA	NA	280-380	1.80
Sandvik 2000	Mississippi 1996 MA > 3kDa	UF Fraction (River)	NA	NA	15.90	NA	NA	280-380	1.70
Sandvik 2000	Blackwater River BWR	UF Fraction (River)	NA	NA	NA	NA	NA	280-380	3.80
Bilski 2003 [141]	Alburg Dune State Park (Pond) Alburg (VT)	Lyophilized (Pond)	NA	NA	NA	NA	NA	365	0.50
Bilski 2003	Mud Creek WMA (Pond) Alburg (VT)	Lyophilized (Pond)	NA	NA	NA	NA	NA	365	1.30
Bilski 2003	Poultney River (Wetland) West Haven (VT)	Lyophilized (Wetland)	NA	NA	NA	NA	NA	365	1.10
Bilski 2003	Otter Creek (Wetland) Ferrisburg (VT)	Lyophilized (Wetland)	NA	NA	NA	NA	NA	365	2.40
Paul 2004 [58]	Val-f_Valkea-Kotinen NOM (fall 1999)	RO Isolate (Lake)	NA	NA	NA	NA	NA	480	0.17
Paul 2004	Bir-f_Birkenes NOM (fall 1999)	RO Isolate (Stream)	NA	NA	NA	NA	NA	480	0.19
Paul 2004	Lu-'NOM'_Luther Marsh 'NOM' (Ontario)	UF Fraction (Wetland)	NA	NA	NA	NA	NA	480	0.21
Paul 2004	SKJ-f_Skjervatjern NOM (fall 1999)	RO Isolate (Lake)	NA	NA	NA	NA	NA	480	0.22
Paul 2004	Suwannee River natural organic matter (SRNOM)	IHSS Isolate (SRNOM)	NA	NA	NA	NA	NA	480	0.23
Paul 2004	SVA-f_Svartberget NOM (fall 1999)	RO Isolate (Stream)	NA	NA	NA	NA	NA	480	0.35
Paul 2004	NOR-NOM_Nordic reference NOM	IHSS Isolate (NLNOM)	NA	NA	NA	NA	NA	480	0.30
Paul 2004	SKJ-s_Skjervatjern NOM (spring 2000)	RO Isolate (Lake)	NA	NA	NA	NA	NA	480	0.29
Paul 2004	Val-s_Valkea-Kotinen NOM (spring 2000)	RO Isolate (Lake)	NA	NA	NA	NA	NA	480	0.30
Paul 2004	SVA-s_Svartberget NOM (spring 2000)	RO Isolate (Stream)	NA	NA	NA	NA	NA	480	0.30
Paul 2004	SVA-s_Svartberget NOM (spring 2000)	RO Isolate (Stream)	NA	NA	NA	NA	NA	430	0.60
Paul 2004	SVA-s_Svartberget NOM (spring 2000)	RO Isolate (Stream)	NA	NA	NA	NA	NA	450	0.52
Paul 2004	SVA-s_Svartberget NOM (spring 2000)	RO Isolate (Stream)	NA	NA	NA	NA	NA	500	0.11
Paul 2004	SVA-s_Svartberget NOM (spring 2000)	RO Isolate (Stream)	NA	NA	NA	NA	NA	550	0.04
Paul 2004	Suwannee River humic acid (SRHA)	IHSS Isolate (SRHA)	NA	NA	NA	NA	NA	480	0.31

Table S18. Summary of $\Phi_{app, {}^1O_2}$ and optical data (continued)

Reference	Sample ID	Sample Classification	*E2:E3*	SUVA$_{254}$ (L mg C^{-1}•m^{-1})	$S_{290–400}$ / $S_{300–400}$ / $S_{290–450}$ / $S_{295–400}$ (μm^{-1})	$S_{300–600}$ / $S_{300–500}$ / $S_{300–700}$ / $S_{275–500}$ (μm^{-1})	FI	Wavelength Range (nm)	$\Phi_{app, {}^1O_2}$ ($\times 10^{-2}$ mol mol-photons^{-1})
Paul 2004	Sa-'NOM'_Sanctuary Pond 'NOM' (Ontario)	UF Fraction (Wetland)	NA	NA	NA	NA	NA	480	0.38
Paul 2004	Heo-NOM_Hellrudmyra NOM	RO Isolate (Lake)	NA	NA	NA	NA	NA	480	0.43
Paul 2004	Fuku-NOM_Fuchskuhle NOM (Germany)	RO Isolate (Lake)	NA	NA	NA	NA	NA	480	0.48
Paul 2004	Suwannee River fulvic acid (SRFA)	IHSS Isolate (SRFA)	NA	NA	NA	NA	NA	480	0.57
Paul 2004	Hiet-f_Hietajärvi NOM (fall 1999)	RO Isolate (Lake)	NA	NA	NA	NA	NA	480	0.28
Paul 2004	Hiet-f_Hietajärvi NOM (fall 1999)	RO Isolate (Lake)	NA	NA	NA	NA	NA	430	0.61
Paul 2004	Hiet-f_Hietajärvi NOM (fall 1999)	RO Isolate (Lake)	NA	NA	NA	NA	NA	450	0.46
Paul 2004	Hiet-f_Hietajärvi NOM (fall 1999)	RO Isolate (Lake)	NA	NA	NA	NA	NA	500	0.15
Paul 2004	Hiet-f_Hietajärvi NOM (fall 1999)	RO Isolate (Lake)	NA	NA	NA	NA	NA	550	0.03
Paul 2004	Hiet-s_Hietajärvi NOM (spring 2000)	RO Isolate (Lake)	NA	NA	NA	NA	NA	480	0.86
Paul 2004	Bir-s_Birkenes NOM (spring 2000)	RO Isolate (Stream)	NA	NA	NA	NA	NA	480	0.91
Paul 2004	Bev-'NOM'_Beverly Swamp 'NOM' (Ontario)	UF Fraction (Wetland)	NA	NA	NA	NA	NA	480	0.61
Paul 2004	HS1500 Synthetic NOM	Synthetic FA (Weyl GmbH)	NA	NA	NA	NA	NA	480	0.06
Paul 2004	Peat(R)-HA_Pahokee peat humic acid reference (PPHA)	IHSS Isolate (PPHA)	NA	NA	NA	NA	NA	480	0.81
Paul 2004	Peat(S)-HA_Pahokee peat humic acid standard (PPHA)	IHSS Isolate (PPHA)	NA	NA	NA	NA	NA	480	0.88
Paul 2004	Peat(S)-HA_Pahokee peat humic acid standard (PPHA)	IHSS Isolate (PPHA)	NA	NA	NA	NA	NA	430	1.40
Paul 2004	Peat(S)-HA_Pahokee peat humic acid standard (PPHA)	IHSS Isolate (PPHA)	NA	NA	NA	NA	NA	450	1.20
Paul 2004	Peat(S)-HA_Pahokee peat humic acid standard (PPHA)	IHSS Isolate (PPHA)	NA	NA	NA	NA	NA	500	0.61
Paul 2004	Peat(S)-HA_Pahokee peat humic acid standard (PPHA)	IHSS Isolate (PPHA)	NA	NA	NA	NA	NA	550	0.40
Paul 2004	Lau-FA_Laurentian fulvic acid (FA)	XAD Fraction (Soil)	NA	NA	NA	NA	NA	480	0.26
Paul 2004	Leon-HA_Leonardit humic acid (LHA)	IHSS Isolate (LHA)	NA	NA	NA	NA	NA	480	0.66
Paul 2004	Sum-HA_Summit Hill Soil humic acid reference	IHSS Isolate (SHSHA)	NA	NA	NA	NA	NA	480	0.67
Paul 2004	Soil-HAII_Elliott soil humic acid standard (ESHA)	IHSS Isolate (ESHA)	NA	NA	NA	NA	NA	480	0.75
Paul 2004	Lau-HA_Laurentian humic acid (HA)	XAD Fraction (Soil)	NA	NA	NA	NA	NA	480	1.08
Paul 2004	Soil-FAII_Elliott soil fulvic acid standard II (ESFA)	IHSS Isolate (ESFA)	NA	NA	NA	NA	NA	480	2.69
Paul 2004	Soil-FAII_Elliott soil fulvic acid standard II (ESFA)	IHSS Isolate (ESFA)	NA	NA	NA	NA	NA	355	3.70
Paul 2004	Soil-FAII_Elliott soil fulvic acid standard II (ESFA)	IHSS Isolate (ESFA)	NA	NA	NA	NA	NA	450	5.51
Paul 2004	Soil-FAII_Elliott soil fulvic acid standard II (ESFA)	IHSS Isolate (ESFA)	NA	NA	NA	NA	NA	500	1.64
Paul 2004	Soil-FAII_Elliott soil fulvic acid standard II (ESFA)	IHSS Isolate (ESFA)	NA	NA	NA	NA	NA	550	0.92
Halladja and Richard 2007 [142]	Elliott soil fulvic acid (ESFA) 320 nm	IHSS Isolate (ESFA)	NA	NA	NA	NA	NA	320	0.14
Halladja and Richard 2007	Elliott soil fulvic acid (ESFA) 340 nm	IHSS Isolate (ESFA)	NA	NA	NA	NA	NA	340	0.14
Halladja and Richard 2007	Elliott soil fulvic acid (ESFA) 375 nm	IHSS Isolate (ESFA)	NA	NA	NA	NA	NA	375	0.18
Halladja and Richard 2007	Elliott soil fulvic acid (ESFA) 400 nm	IHSS Isolate (ESFA)	NA	NA	NA	NA	NA	400	0.24
Halladja and Richard 2007	Elliott soil fulvic acid (ESFA) 425 nm	IHSS Isolate (ESFA)	NA	NA	NA	NA	NA	425	0.24
Halladja and Richard 2007	Elliott soil fulvic acid (ESFA) 450 nm	IHSS Isolate (ESFA)	NA	NA	NA	NA	NA	450	0.20
Halladja and Richard 2007	Elliott soil fulvic acid (ESFA) 500 nm	IHSS Isolate (ESFA)	NA	NA	NA	NA	NA	500	0.05
Cory and McNeill 2009 [143]	Suwannee River fulvic acid (SRFA)	IHSS Isolate (SRFA)	NA	3.20	NA	NA	1.24	350	0.47
Cory and McNeill 2009	Pony Lake fulvic acid (PLFA)	IHSS Isolate (PLFA)	NA	1.70	NA	NA	1.51	350	0.69
Dalrymple and Sharpless 2010 [38]	Pony Lake tangential flow ultrafiltration (TFUF)	UF Fraction (PLFA)	3.06	NA	NA	NA	NA	365	0.59
Dalrymple and Sharpless 2010	Nordic aquatic humic acid (NHA)	IHSS Isolate (NHA)	3.48	NA	NA	NA	NA	365	1.18
Dalrymple and Sharpless 2010	Lake Toolik tangential flow ultrafiltration (TFUF)	UF Fraction (Lake)	4.12	NA	NA	NA	NA	365	1.26
Dalrymple and Sharpless 2010	Suwannee River natural organic matter (SDOM)	IHSS Isolate (SRNOM)	3.64	NA	NA	NA	NA	365	1.53
Dalrymple and Sharpless 2010	Suwannee River natural organic matter (SDOM)	IHSS Isolate (SRNOM)	4.16	NA	NA	NA	NA	365	1.81
Dalrymple and Sharpless 2010	Suwannee River natural organic matter (SDOM)	IHSS Isolate (SRNOM)	3.99	NA	NA	NA	NA	365	1.82
Dalrymple and Sharpless 2010	Nordic aquatic fulvic acid (NFA)	IHSS Isolate (NFA)	4.15	NA	NA	NA	NA	365	2.03
Dalrymple and Sharpless 2010	Suwannee River natural organic matter (SDOM)	IHSS Isolate (SRNOM)	4.78	NA	NA	NA	NA	365	2.38
Dalrymple and Sharpless 2010	Nordic aquatic natural organic matter (NDOM)	IHSS Isolate (NLNOM)	4.54	NA	NA	NA	NA	365	2.48
Dalrymple and Sharpless 2010	Lake Anna XAD (LAX) HOPA (Virginia)	XAD Fraction (Lake)	5.55	NA	NA	NA	NA	365	2.81
Dalrymple and Sharpless 2010	Pony Lake XAD HOPA	XAD Fraction (PLFA)	4.70	NA	NA	NA	NA	365	3.01
Dalrymple and Sharpless 2010	Rappahannock River XAD (RR5X, sampled 3/07) HOPA Waterview site	XAD Fraction (River)	5.52	NA	NA	NA	NA	365	3.24
Dalrymple and Sharpless 2010	Rappahannock River XAD (RR3X, sampled 3/07) HOPA Hicks Landing site	XAD Fraction (River)	4.95	NA	NA	NA	NA	365	3.25

Table S18. Summary of $\Phi_{app, {}^1O_2}$ and optical data (continued)

Reference	Sample ID	Sample Classification	*E2:E3*	$SUVA_{254}$ (L mg C^{-1}•m^{-1})	$S_{290–400}$ / $S_{300–400}$ / $S_{290–450}$ / $S_{295–400}$ (μm^{-1})	$S_{300–600}$ / $S_{300–500}$ / $S_{300–700}$ / $S_{275–500}$ (μm^{-1})	FI	Wavelength Range (nm)	$\Phi_{app, {}^1O_2}$ (×10^{-2} mol mol-photons^{-1})
Dalrymple and Sharpless 2010	Rappahannock River UF (RR5UFR) Waterview site	UF Fraction (River)	6.96	NA	NA	NA	NA	365	3.34
Dalrymple and Sharpless 2010	Lake Anna UF (LAUFR)	UF Fraction (Lake)	6.66	NA	NA	NA	NA	365	4.02
Dalrymple and Sharpless 2010	Lake Toolik XAD HOPA	XAD Fraction (Lake)	5.55	NA	NA	NA	NA	365	4.14
Dalrymple and Sharpless 2010	Rappahannock River XAD (RR3X, sampled 6/07) HOPA Hicks Landing site	XAD Fraction (River)	4.50	NA	NA	NA	NA	365	3.65
Dalrymple and Sharpless 2010	Rappahannock River XAD (RR5X, sampled 6/07) HOPA Waterview site	XAD Fraction (River)	5.42	NA	NA	NA	NA	365	4.50
Dalrymple and Sharpless 2010	Mt. Pleasant (Virginia)	Whole Water (Wetland)	4.51	NA	NA	NA	NA	365	1.50
Dalrymple and Sharpless 2010	Tappahannock (Virginia)	Whole Water (Wetland)	4.59	NA	NA	NA	NA	365	2.10
Dalrymple and Sharpless 2010	Waterview XAD isolate	XAD Fraction (River)	5.32	NA	NA	NA	NA	365	3.20
Carlos 2012 [144]	Aldrich humic acid (AHA)	XAD Fraction (Soil)	NA	NA	NA	NA	NA	400	0.10
Carlos 2012	Leonardite humic acid (LHA)	IHSS Isolate (LHA)	NA	NA	NA	NA	NA	400	0.40
Carlos 2012	Fe3O4/AHA	XAD Fraction (Soil, Magnetite)	NA	NA	NA	NA	NA	400	0.07
Carlos 2012	Fe3O4/LHA	IHSS Isolate (LHA, Magnetite)	NA	NA	NA	NA	NA	400	0.07
Peterson and McNeill 2012 [19]	SLR_2 Jun 07	Whole Water (River)	5.18	NA	14.00	17.00	1.22	365	1.80
Peterson and McNeill 2012	SLR_5 Sep 07	Whole Water (River)	7.01	NA	17.00	19.00	1.31	365	2.70
Peterson and McNeill 2012	SLR_15 May 08	Whole Water (River)	4.80	NA	13.00	16.00	1.17	365	0.93
Peterson and McNeill 2012	SLB_26 Aug 08	Whole Water (River)	5.86	NA	21.00	17.00	1.25	365	1.40
Peterson and McNeill 2012	HBR_2 Jun 07	Whole Water (Lake)	5.65	NA	15.00	17.00	1.27	365	2.00
Peterson and McNeill 2012	HBR_5 Sep 07	Whole Water (Lake)	7.00	NA	18.00	19.00	1.38	365	2.50
Peterson and McNeill 2012	HBR_15 May 08	Whole Water (Lake)	4.86	NA	13.00	16.00	1.18	365	0.93
Peterson and McNeill 2012	HBR_26 Aug 08	Whole Water (Lake)	5.97	NA	21.00	17.00	1.32	365	1.50
Peterson and McNeill 2012	A_30 May 07	Whole Water (Lake)	6.57	NA	17.00	18.00	1.34	365	2.10
Peterson and McNeill 2012	A_5 Sep 07	Whole Water (Lake)	13.60	NA	28.00	23.00	1.36	365	3.70
Peterson and McNeill 2012	A_13 May 08	Whole Water (Lake)	4.70	NA	13.00	16.00	1.29	365	0.99
Peterson and McNeill 2012	A_26 Aug 08	Whole Water (Lake)	9.01	NA	20.00	19.00	1.34	365	3.20
Peterson and McNeill 2012	AB1_2 Jun 07	Whole Water (Lake)	6.76	NA	18.00	18.00	1.33	365	2.20
Peterson and McNeill 2012	AB1_5 Sep 07	Whole Water (Lake)	15.80	NA	29.00	25.00	1.37	365	4.70
Peterson and McNeill 2012	AB1_13 May 08	Whole Water (Lake)	5.03	NA	14.00	16.00	1.30	365	0.97
Peterson and McNeill 2012	AB1_26 Aug 08	Whole Water (Lake)	9.25	NA	23.00	19.00	1.32	365	2.70
Peterson and McNeill 2012	AB2_2 Jun 07	Whole Water (Lake)	6.97	NA	18.00	18.00	1.33	365	2.30
Peterson and McNeill 2012	AB2_5 Sep 07	Whole Water (Lake)	17.40	NA	31.00	26.00	1.37	365	5.50
Peterson and McNeill 2012	AB2_13 May 08	Whole Water (Lake)	7.46	NA	19.00	18.00	1.32	365	1.70
Peterson and McNeill 2012	AB2_26 Aug 08	Whole Water (Lake)	9.12	NA	23.00	19.00	1.33	365	3.40
Peterson and McNeill 2012	B_30 May 07	Whole Water (Lake)	7.61	NA	21.00	19.00	1.35	365	2.20
Peterson and McNeill 2012	B_5 Sep 07	Whole Water (Lake)	15.20	NA	29.00	25.00	1.34	365	4.40
Peterson and McNeill 2012	B_13 May 08	Whole Water (Lake)	9.28	NA	23.00	21.00	1.30	365	1.90
Peterson and McNeill 2012	B_26 Aug 08	Whole Water (Lake)	10.30	NA	24.00	19.00	1.34	365	2.90
Peterson and McNeill 2012	C_30 May 07	Whole Water (Lake)	7.74	NA	20.00	19.00	1.32	365	2.20
Peterson and McNeill 2012	C_5 Sep 07	Whole Water (Lake)	16.70	NA	29.00	25.00	1.36	365	5.10
Peterson and McNeill 2012	C_13 May 08	Whole Water (Lake)	10.80	NA	23.00	19.00	1.30	365	2.10
Peterson and McNeill 2012	C_26 Aug 08	Whole Water (Lake)	12.30	NA	24.00	20.00	1.37	365	3.40
Peterson and McNeill 2012	D_1 Jun 07	Whole Water (Lake)	9.82	NA	26.00	22.00	1.32	365	2.50
Peterson and McNeill 2012	D_5 Sep 07	Whole Water (Lake)	16.50	NA	29.00	25.00	1.39	365	5.10
Peterson and McNeill 2012	D_13 May 08	Whole Water (Lake)	21.40	NA	30.00	22.00	1.29	365	2.50
Peterson and McNeill 2012	D_26 Aug 08	Whole Water (Lake)	10.50	NA	26.00	19.00	1.35	365	2.40
Peterson and McNeill 2012	F_1 Jun 07	Whole Water (Lake)	10.20	NA	28.00	22.00	1.33	365	2.30
Peterson and McNeill 2012	F_5 Sep 07	Whole Water (Lake)	16.20	NA	29.00	26.00	1.38	365	4.50
Peterson and McNeill 2012	F_13 May 08	Whole Water (Lake)	16.20	NA	30.00	22.00	1.31	365	2.70
Peterson and McNeill 2012	F_26 Aug 08	Whole Water (Lake)	15.80	NA	26.00	21.00	1.34	365	3.90
Peterson and McNeill 2012	G_1 Jun 07	Whole Water (Lake)	13.00	NA	29.00	23.00	1.33	365	3.10
Peterson and McNeill 2012	G_26 Aug 08	Whole Water (Lake)	16.40	NA	24.00	22.00	1.36	365	3.60
Peterson and McNeill 2012	I_1 Jun 07	Whole Water (Lake)	12.20	NA	30.00	23.00	1.30	365	2.80

Table S18. Summary of $\Phi_{app,\,{}^1O_2}$ and optical data (continued)

Reference	Sample ID	Sample Classification	*E2:E3*	$SUVA_{254}$ (L mg C^{-1}•m^{-1})	$S_{290–400}$ / $S_{300–400}$ / $S_{290–450}$ / $S_{295–400}$ (μm^{-1})	$S_{300–600}$ / $S_{300–500}$ / $S_{300–700}$ / $S_{275–500}$ (μm^{-1})	FI	Wavelength Range (nm)	$\Phi_{app,\,{}^1O_2}$ ($\times 10^{-2}$ mol mol-photons^{-1})
Peterson and McNeill 2012	10/WML_31 May 07	Whole Water (Lake)	11.30	NA	30.00	22.00	1.31	365	2.50
Peterson and McNeill 2012	10/WML_14 May 08	Whole Water (Lake)	14.70	NA	30.00	23.00	1.29	365	3.20
Peterson and McNeill 2012	10/WML_20 Jun 09	Whole Water (Lake)	15.10	NA	30.00	24.00	1.33	365	2.10
Peterson and McNeill 2012	CDM_30 May 07	Whole Water (Lake)	6.40	NA	17.00	18.00	1.34	365	2.20
Peterson and McNeill 2012	CDM_5 Sep 07	Whole Water (Lake)	0.02	NA	25.00	16.50	4.20	365	4.20
Peterson and McNeill 2012	EM_13 Jun 09	Whole Water (Lake)	13.50	NA	31.00	24.00	1.36	365	2.80
Peterson and McNeill 2012	SM_14 Jun 09	Whole Water (Lake)	14.20	NA	32.00	25.00	1.35	365	2.50
Peterson and McNeill 2012	CM_15 Jun 09	Whole Water (Lake)	15.10	NA	31.00	24.00	1.33	365	3.30
Peterson and McNeill 2012	NM_16 Jun 09	Whole Water (Lake)	13.40	NA	30.00	23.00	1.32	365	3.20
Peterson and McNeill 2012	Baptism R. (offshore)_16 Jun 09	Whole Water (Lake)	9.50	NA	24.00	20.00	1.31	365	2.60
Peterson and McNeill 2012	Ontonagon R. (offshore)_19 Jun 09	Whole Water (Lake)	9.75	NA	24.00	20.00	1.35	365	2.00
Sharpless 2012 [145]	Suwannee River natural organic matter (SRNOM)	IHSS Isolate (SRNOM)	NA	NA	NA	NA	NA	370	1.40
Sharpless 2012	SRNOM NaBH4 reduced	IHSS Isolate (SRNOM, NaBH4)	NA	NA	NA	NA	NA	370	1.40
Sharpless 2012	Suwannee River fulvic acid (SRFA)	IHSS Isolate (SRFA)	NA	NA	NA	NA	NA	370	1.80
Sharpless 2012	SRFA NaBH4 reduced	IHSS Isolate (SRFA, NaBH4)	NA	NA	NA	NA	NA	370	1.80
Sharpless 2012	Nordic Lake natural organic matter (NLNOM)	IHSS Isolate (NLNOM)	NA	NA	NA	NA	NA	370	2.70
Sharpless 2012	NLNOM reduced	IHSS Isolate (NLNOM, NaBH4)	NA	NA	NA	NA	NA	370	3.20
De Laurentiis and Vione 2013 [146]	II-10B_2 (Inexpressible Island lake)	Whole Water (Lake)	9.24	0.55	9.90	NA	NA	295-600	0.42
De Laurentiis and Vione 2013	EP-14 (Edmonson Point)	Whole Water (Lake)	9.93	1.07	9.60	NA	NA	295-600	6.60
De Laurentiis and Vione 2013	EP-15A (Edmonson Point)	Whole Water (Lake)	2.88	0.80	5.70	NA	NA	295-600	0.46
De Laurentiis and Vione 2013	TF-20_1 (Tarn Flat)	Whole Water (Lake)	2.23	1.32	4.10	NA	NA	295-600	0.76
De Laurentiis and Vione 2013	TF-20_2 (Tarn Flat)	Whole Water (Lake)	4.28	0.84	8.40	NA	NA	295-600	7.02
De Laurentiis and Vione 2013	GW lake (Gondwana Lake)	Whole Water (Lake)	3.74	0.75	7.90	NA	NA	295-600	0.71
Glover and Rosario-Ortiz 2013 [147]	SRHA_Cl_0 mM	IHSS Isolate (SRHA)	NA	NA	NA	NA	NA	290-400	0.29
Glover and Rosario-Ortiz 2013	SRHA_Cl_0.08 mM	IHSS Isolate (SRHA, Chloride)	NA	NA	NA	NA	NA	290-400	0.38
Glover and Rosario-Ortiz 2013	SRHA_Cl_0.31 mM	IHSS Isolate (SRHA, Chloride)	NA	NA	NA	NA	NA	290-400	0.33
Glover and Rosario-Ortiz 2013	SRHA_Cl_3.08 mM	IHSS Isolate (SRHA, Chloride)	NA	NA	NA	NA	NA	290-400	0.38
Glover and Rosario-Ortiz 2013	SRHA_Cl_4.62 mM	IHSS Isolate (SRHA, Chloride)	NA	NA	NA	NA	NA	290-400	0.51
Glover and Rosario-Ortiz 2013	SRHA_Cl_50 mM	IHSS Isolate (SRHA, Chloride)	NA	NA	NA	NA	NA	290-400	2.23
Glover and Rosario-Ortiz 2013	SRHA_Cl_100 mM	IHSS Isolate (SRHA, Chloride)	NA	NA	NA	NA	NA	290-400	2.67
Glover and Rosario-Ortiz 2013	SRHA_Cl_500 mM	IHSS Isolate (SRHA, Chloride)	NA	NA	NA	NA	NA	290-400	2.89
Glover and Rosario-Ortiz 2013	SRHA_Br_0 mM	IHSS Isolate (SRHA)	NA	NA	NA	NA	NA	290-400	0.29
Glover and Rosario-Ortiz 2013	SRHA_Br_0.0093 mM	IHSS Isolate (SRHA, Bromide)	NA	NA	NA	NA	NA	290-400	0.30
Glover and Rosario-Ortiz 2013	SRHA_Br_0.0698 mM	IHSS Isolate (SRHA, Bromide)	NA	NA	NA	NA	NA	290-400	0.49
Glover and Rosario-Ortiz 2013	SRHA_Br_0.093 mM	IHSS Isolate (SRHA, Bromide)	NA	NA	NA	NA	NA	290-400	1.14
Glover and Rosario-Ortiz 2013	SRHA_Br_0.1861 mM	IHSS Isolate (SRHA, Bromide)	NA	NA	NA	NA	NA	290-400	2.46
Glover and Rosario-Ortiz 2013	SRHA_Br_0.08 mM	IHSS Isolate (SRHA, Bromide)	NA	NA	NA	NA	NA	290-400	2.60
Glover and Rosario-Ortiz 2013	SRHA_Br_0.4 mM	IHSS Isolate (SRHA, Bromide)	NA	NA	NA	NA	NA	290-400	2.89
Glover and Rosario-Ortiz 2013	SRHA_Br_0.8 mM	IHSS Isolate (SRHA, Bromide)	NA	NA	NA	NA	NA	290-400	2.45
Glover and Rosario-Ortiz 2013	SRFA_Cl_0 mM	IHSS Isolate (SRFA)	NA	NA	NA	NA	NA	290-400	0.37
Glover and Rosario-Ortiz 2013	SRFA_Cl_0.08 mM	IHSS Isolate (SRFA, Chloride)	NA	NA	NA	NA	NA	290-400	0.48
Glover and Rosario-Ortiz 2013	SRFA_Cl_0.31 mM	IHSS Isolate (SRFA, Chloride)	NA	NA	NA	NA	NA	290-400	0.49
Glover and Rosario-Ortiz 2013	SRFA_Cl_3.08 mM	IHSS Isolate (SRFA, Chloride)	NA	NA	NA	NA	NA	290-400	0.53
Glover and Rosario-Ortiz 2013	SRFA_Cl_4.62 mM	IHSS Isolate (SRFA, Chloride)	NA	NA	NA	NA	NA	290-400	0.59
Glover and Rosario-Ortiz 2013	SRFA_Cl_50 mM	IHSS Isolate (SRFA, Chloride)	NA	NA	NA	NA	NA	290-400	3.03
Glover and Rosario-Ortiz 2013	SRFA_Cl_100 mM	IHSS Isolate (SRFA, Chloride)	NA	NA	NA	NA	NA	290-400	3.26
Glover and Rosario-Ortiz 2013	SRFA_Cl_500 mM	IHSS Isolate (SRFA, Chloride)	NA	NA	NA	NA	NA	290-400	3.16
Glover and Rosario-Ortiz 2013	SRFA_Br_0 mM	IHSS Isolate (SRFA)	NA	NA	NA	NA	NA	290-400	0.49
Glover and Rosario-Ortiz 2013	SRFA_Br_0.0093 mM	IHSS Isolate (SRFA, Bromide)	NA	NA	NA	NA	NA	290-400	0.69
Glover and Rosario-Ortiz 2013	SRFA_Br_0.0698 mM	IHSS Isolate (SRFA, Bromide)	NA	NA	NA	NA	NA	290-400	1.13
Glover and Rosario-Ortiz 2013	SRFA_Br_0.093 mM	IHSS Isolate (SRFA, Bromide)	NA	NA	NA	NA	NA	290-400	1.19

Table S18. Summary of $\Phi_{app, {}^1O_2}$ and optical data (continued)									
Reference	Sample ID	Sample Classification	*E2:E3*	SUVA$_{254}$ (L mg C^{-1}•m^{-1})	$S_{290–400}$ / $S_{300–400}$ / $S_{290–450}$ / $S_{295–400}$ (μm^{-1})	$S_{300–600}$ / $S_{300–500}$ / $S_{300–700}$ / $S_{275–500}$ (μm^{-1})	FI	Wavelength Range (nm)	$\Phi_{app, {}^1O_2}$ (×10^{-2} mol mol-photons^{-1})
Glover and Rosario-Ortiz 2013	SRFA_Br_0.1861 mM	IHSS Isolate (SRFA, Bromide)	NA	NA	NA	NA	NA	290-400	3.48
Glover and Rosario-Ortiz 2013	SRFA_Br_0.08 mM	IHSS Isolate (SRFA, Bromide)	NA	NA	NA	NA	NA	290-400	3.26
Glover and Rosario-Ortiz 2013	SRFA_Br_0.4 mM	IHSS Isolate (SRFA, Bromide)	NA	NA	NA	NA	NA	290-400	3.16
Glover and Rosario-Ortiz 2013	SRFA_Br_0.8 mM	IHSS Isolate (SRFA, Bromide)	NA	NA	NA	NA	NA	290-400	3.19
Glover and Rosario-Ortiz 2013	SRNOM_Cl_0 mM	IHSS Isolate (SRNOM)	NA	NA	NA	NA	NA	290-400	0.21
Glover and Rosario-Ortiz 2013	SRNOM_Cl_0.08 mM	IHSS Isolate (SRNOM, Chloride)	NA	NA	NA	NA	NA	290-400	0.28
Glover and Rosario-Ortiz 2013	SRNOM_Cl_0.31 mM	IHSS Isolate (SRNOM, Chloride)	NA	NA	NA	NA	NA	290-400	0.41
Glover and Rosario-Ortiz 2013	SRNOM_Cl_3.08 mM	IHSS Isolate (SRNOM, Chloride)	NA	NA	NA	NA	NA	290-400	0.51
Glover and Rosario-Ortiz 2013	SRNOM_Cl_4.62 mM	IHSS Isolate (SRNOM, Chloride)	NA	NA	NA	NA	NA	290-400	0.96
Glover and Rosario-Ortiz 2013	SRNOM_Cl_50 mM	IHSS Isolate (SRNOM, Chloride)	NA	NA	NA	NA	NA	290-400	1.24
Glover and Rosario-Ortiz 2013	SRNOM_Cl_100 mM	IHSS Isolate (SRNOM, Chloride)	NA	NA	NA	NA	NA	290-400	2.37
Glover and Rosario-Ortiz 2013	SRNOM_Cl_500 mM	IHSS Isolate (SRNOM, Chloride)	NA	NA	NA	NA	NA	290-400	2.37
Glover and Rosario-Ortiz 2013	SRNOM_Br_0 mM	IHSS Isolate (SRNOM)	NA	NA	NA	NA	NA	290-400	0.21
Glover and Rosario-Ortiz 2013	SRNOM_Br_0.0093 mM	IHSS Isolate (SRNOM, Bromide)	NA	NA	NA	NA	NA	290-400	0.57
Glover and Rosario-Ortiz 2013	SRNOM_Br_0.0698 mM	IHSS Isolate (SRNOM, Bromide)	NA	NA	NA	NA	NA	290-400	0.77
Glover and Rosario-Ortiz 2013	SRNOM_Br_0.093 mM	IHSS Isolate (SRNOM, Bromide)	NA	NA	NA	NA	NA	290-400	0.88
Glover and Rosario-Ortiz 2013	SRNOM_Br_0.1861 mM	IHSS Isolate (SRNOM, Bromide)	NA	NA	NA	NA	NA	290-400	1.00
Glover and Rosario-Ortiz 2013	SRNOM_Br_0.08 mM	IHSS Isolate (SRNOM, Bromide)	NA	NA	NA	NA	NA	290-400	0.82
Glover and Rosario-Ortiz 2013	SRNOM_Br_0.4 mM	IHSS Isolate (SRNOM, Bromide)	NA	NA	NA	NA	NA	290-400	1.24
Glover and Rosario-Ortiz 2013	SRNOM_Br_0.8 mM	IHSS Isolate (SRNOM, Bromide)	NA	NA	NA	NA	NA	290-400	2.00
Glover and Rosario-Ortiz 2013	PLFA_Cl_0 mM	IHSS Isolate (PLFA)	NA	NA	NA	NA	NA	290-400	1.14
Glover and Rosario-Ortiz 2013	PLFA_Cl_0.08 mM	IHSS Isolate (PLFA, Chloride)	NA	NA	NA	NA	NA	290-400	1.73
Glover and Rosario-Ortiz 2013	PLFA_Cl_0.31 mM	IHSS Isolate (PLFA, Chloride)	NA	NA	NA	NA	NA	290-400	4.93
Glover and Rosario-Ortiz 2013	PLFA_Cl_3.08 mM	IHSS Isolate (PLFA, Chloride)	NA	NA	NA	NA	NA	290-400	6.45
Glover and Rosario-Ortiz 2013	PLFA_Cl_4.62 mM	IHSS Isolate (PLFA, Chloride)	NA	NA	NA	NA	NA	290-400	6.86
Glover and Rosario-Ortiz 2013	PLFA_Cl_50 mM	IHSS Isolate (PLFA, Chloride)	NA	NA	NA	NA	NA	290-400	5.25
Glover and Rosario-Ortiz 2013	PLFA_Cl_100 mM	IHSS Isolate (PLFA, Chloride)	NA	NA	NA	NA	NA	290-400	6.13
Glover and Rosario-Ortiz 2013	PLFA_Cl_500 mM	IHSS Isolate (PLFA, Chloride)	NA	NA	NA	NA	NA	290-400	6.27
Glover and Rosario-Ortiz 2013	PLFA_Br_0 mM	IHSS Isolate (PLFA)	NA	NA	NA	NA	NA	290-400	1.01
Glover and Rosario-Ortiz 2013	PLFA_Br_0.0093 mM	IHSS Isolate (PLFA, Bromide)	NA	NA	NA	NA	NA	290-400	1.06
Glover and Rosario-Ortiz 2013	PLFA_Br_0.0698 mM	IHSS Isolate (PLFA, Bromide)	NA	NA	NA	NA	NA	290-400	1.09
Glover and Rosario-Ortiz 2013	PLFA_Br_0.093 mM	IHSS Isolate (PLFA, Bromide)	NA	NA	NA	NA	NA	290-400	1.14
Glover and Rosario-Ortiz 2013	PLFA_Br_0.1861 mM	IHSS Isolate (PLFA, Bromide)	NA	NA	NA	NA	NA	290-400	1.20
Glover and Rosario-Ortiz 2013	PLFA_Br_0.08 mM	IHSS Isolate (PLFA, Bromide)	NA	NA	NA	NA	NA	290-400	6.10
Glover and Rosario-Ortiz 2013	PLFA_Br_0.4 mM	IHSS Isolate (PLFA, Bromide)	NA	NA	NA	NA	NA	290-400	6.27
Glover and Rosario-Ortiz 2013	PLFA_Br_0.8 mM	IHSS Isolate (PLFA, Bromide)	NA	NA	NA	NA	NA	290-400	5.42
Mostafa and Rosario-Ortiz 2013 [35]	Suwannee River natural organic matter (SRNOM)	IHSS Isolate (SRNOM)	3.94	NA	NA	NA	NA	290-400	2.02
Mostafa and Rosario-Ortiz 2013	Pony Lake fulvic acid (PLFA)	IHSS Isolate (PLFA)	4.50	NA	NA	NA	NA	290-400	2.04
Mostafa and Rosario-Ortiz 2013	Suwannee River fulvic acid (SRFA)	IHSS Isolate (SRFA)	4.77	NA	NA	NA	NA	290-400	2.11
Mostafa and Rosario-Ortiz 2013	Suwannee River humic acid (SRHA)	IHSS Isolate (SRHA)	3.06	NA	NA	NA	NA	290-400	1.60
Mostafa and Rosario-Ortiz 2013	Louisville (LWW)-UV disinfection (eff)	Whole Water (WWTP Effluent)	4.79	NA	NA	16.70	NA	290-400	2.77
Mostafa and Rosario-Ortiz 2013	LWW-eff < 10k Da	UF Fraction (WWTP Effluent)	4.96	NA	NA	18.50	NA	290-400	2.79
Mostafa and Rosario-Ortiz 2013	LWW-eff < 1k Da	UF Fraction (WWTP Effluent)	5.27	NA	NA	16.80	NA	290-400	3.88
Mostafa and Rosario-Ortiz 2013	LWW + 0.14 mM Cl2	UF Fraction (WWTP Effluent, Cl2)	6.02	NA	NA	NA	NA	290-400	4.53
Mostafa and Rosario-Ortiz 2013	LWW + 0.28 mM Cl2	UF Fraction (WWTP Effluent, Cl2)	7.25	NA	NA	NA	NA	290-400	6.11
Mostafa and Rosario-Ortiz 2013	LWW + 0.42 mM Cl2	UF Fraction (WWTP Effluent, Cl2)	8.52	NA	NA	NA	NA	290-400	6.52
Mostafa and Rosario-Ortiz 2013	LWW + 0.56 mM Cl2	UF Fraction (WWTP Effluent, Cl2)	10.11	NA	NA	NA	NA	290-400	8.01
Mostafa and Rosario-Ortiz 2013	Boulder (BWW)-secondary clarification (2C)	Whole Water (WWTP Effluent)	4.59	NA	NA	16.70	NA	290-400	3.18
Mostafa and Rosario-Ortiz 2013	BWW-2C < 10 kDa	UF Fraction (WWTP Effluent)	4.86	NA	NA	19.60	NA	290-400	3.91
Mostafa and Rosario-Ortiz 2013	BWW-2C < 1 kDa	UF Fraction (WWTP Effluent)	5.28	NA	NA	18.70	NA	290-400	5.78
Mostafa and Rosario-Ortiz 2013	BWW-final chlorination (eff)	Whole Water (WWTP Effluent)	5.71	NA	NA	19.20	NA	290-400	4.66

**Table S18.** Summary of $\Phi_{app,\ ^1O_2}$ and optical data (continued)									
Reference	Sample ID	Sample Classification	*E2:E3*	$SUVA_{254}$ (L mg C^{-1}•m^{-1})	$S_{290-400}$ / $S_{300-400}$ / $S_{290-450}$ / $S_{295-400}$ (μm^{-1})	$S_{300-600}$ / $S_{300-500}$ / $S_{300-700}$ / $S_{275-500}$ (μm^{-1})	FI	Wavelength Range (nm)	$\Phi_{app,\ ^1O_2}$ ($\times 10^{-2}$ mol mol-photons^{-1})
Mostafa and Rosario-Ortiz 2013	BWW-eff < 10 kDa	UF Fraction (WWTP Effluent)	5.70	NA	NA	22.10	NA	290-400	5.62
Mostafa and Rosario-Ortiz 2013	BWW-eff < 1k Da	UF Fraction (WWTP Effluent)	6.51	NA	NA	16.70	NA	290-400	8.59
Mostafa and Rosario-Ortiz 2013	BWW-2C + 0.07 mM Cl2	Whole Water (WWTP Effluent, Cl2)	5.19	NA	NA	NA	NA	290-400	3.82
Mostafa and Rosario-Ortiz 2013	BWW-2C + 0.14 mM Cl2	Whole Water (WWTP Effluent, Cl2)	6.46	NA	NA	NA	NA	290-400	5.09
Mostafa and Rosario-Ortiz 2013	BWW-2C + 0.21 mM Cl2	Whole Water (WWTP Effluent, Cl2)	8.22	NA	NA	NA	NA	290-400	6.67
Mostafa and Rosario-Ortiz 2013	BWW-2C + 0.28 mM Cl2	Whole Water (WWTP Effluent, Cl2)	10.16	NA	NA	NA	NA	290-400	7.13
Mostafa and Rosario-Ortiz 2013	BWW-2C + 0.42 mM Cl2	Whole Water (WWTP Effluent, Cl2)	15.84	NA	NA	NA	NA	290-400	7.36
Mostafa and Rosario-Ortiz 2013	BWW-2C + 0.56 mM Cl2	Whole Water (WWTP Effluent, Cl2)	17.63	NA	NA	NA	NA	290-400	5.74
Mostafa and Rosario-Ortiz 2013	BWW-2C + 0.044 mM O3	Whole Water (WWTP Effluent, O3)	7.39	NA	NA	NA	NA	290-400	4.38
Mostafa and Rosario-Ortiz 2013	BWW-2C + 0.088 mM O3	Whole Water (WWTP Effluent, O3)	11.12	NA	NA	NA	NA	290-400	5.18
Mostafa and Rosario-Ortiz 2013	BWW-2C + 0.132 mM O3	Whole Water (WWTP Effluent, O3)	17.21	NA	NA	NA	NA	290-400	7.91
Mostafa and Rosario-Ortiz 2013	BWW-2C + 0.176 mM O3	Whole Water (WWTP Effluent, O3)	16.77	NA	NA	NA	NA	290-400	9.32
Cawley and Rosario-Ortiz 2014 [148]	Suwannee River natural organic matter (SRNOM)	IHSS Isolate (SRNOM)	4.56	3.92	NA	NA	1.29	290-400	2.87
Cawley and Rosario-Ortiz 2014	Suwannee River HPOA	XAD Fraction (SRNOM)	5.99	2.28	NA	NA	1.39	290-400	2.74
Cawley and Rosario-Ortiz 2014	Suwannee River TPIA	XAD Fraction (SRNOM)	4.20	5.55	NA	NA	1.28	290-400	6.54
Janssen and McNeill 2014 [149]	Suwannee River fulvic acid (SRFA)	IHSS Isolate (SRFA)	NA	NA	NA	NA	NA	250-500	5.40
Janssen and McNeill 2014	Waskish peat humic acid (WPHA)	IHSS Isolate (WPHA)	NA	NA	NA	NA	NA	250-500	1.85
Janssen and McNeill 2014	Nordic Lake natural organic matter (NLNOM)	IHSS Isolate (NLNOM)	NA	NA	NA	NA	NA	250-500	5.00
Janssen and McNeill 2014	Aldrich humic acid	XAD Fraction (Soil)	NA	NA	NA	NA	NA	250-500	4.40
Mostafa and Rosario-Ortiz 2014 [150]	Suwannee River natural organic matter (SRNOM)	IHSS Isolate (SRNOM)	4.11	0.99	NA	14.00	1.29	290-400	1.85
Mostafa and Rosario-Ortiz 2014	SRNOM > 10kDa	UF Fraction (SRNOM)	3.25	2.17	NA	12.00	1.06	290-400	0.44
Mostafa and Rosario-Ortiz 2014	SRNOM 1-10 kDa	UF Fraction (SRNOM)	4.18	1.44	NA	14.00	1.19	290-400	1.44
Mostafa and Rosario-Ortiz 2014	SRNOM < 1kDa	UF Fraction (SRNOM)	5.83	0.56	NA	17.00	1.47	290-400	4.39
Mostafa and Rosario-Ortiz 2014	BWW	Whole Water (WWTP Effluent)	4.62	2.43	NA	14.00	2.05	290-400	3.29
Mostafa and Rosario-Ortiz 2014	BWW >10 kDa	UF Fraction (WWTP Effluent)	3.19	2.59	NA	10.00	1.51	290-400	1.15
Mostafa and Rosario-Ortiz 2014	BWW 1-10 kDa	UF Fraction (WWTP Effluent)	3.70	2.01	NA	12.00	1.54	290-400	1.91
Mostafa and Rosario-Ortiz 2014	BWW <1 kDa	UF Fraction (WWTP Effluent)	7.80	2.48	NA	24.00	2.14	290-400	6.18
Nkhili and Richard 2014 [151]	L-WEOM20	WEOM (Soil)	9.50	NA	NA	NA	NA	290-450	2.71
Nkhili and Richard 2014	L-WEOM60	WEOM (Soil)	7.18	NA	NA	NA	NA	290-450	4.17
Nkhili and Richard 2014	P-WEOM20	WEOM (Soil)	4.38	NA	NA	NA	NA	290-450	1.00
Nkhili and Richard 2014	P-WEOM60	WEOM (Soil)	4.06	NA	NA	NA	NA	290-450	1.21
Nkhili and Richard 2014	E-WEOM20	WEOM (Soil)	6.55	NA	NA	NA	NA	290-450	1.42
Nkhili and Richard 2014	E-WEOM60	WEOM (Soil)	7.20	NA	NA	NA	NA	290-450	1.00
Nkhili and Richard 2014	E-FA	IHSS Isolate (ESFA)	4.06	NA	NA	NA	NA	290-450	5.42
Nkhili and Richard 2014	E-HA	IHSS Isolate (ESHA)	2.30	NA	NA	NA	NA	290-450	2.08
Sharpless 2014 [33]	Suwannee River Fulvic Acid (SRFA) 0 h	IHSS Isolate (SRFA)	4.13	NA	NA	NA	NA	300-500	1.80
Sharpless 2014	Suwannee River Fulvic Acid (SRFA) 11 h	IHSS Isolate (SRFA)	4.68	NA	NA	NA	NA	300-500	1.92
Sharpless 2014	Suwannee River Fulvic Acid (SRFA) 35 h	IHSS Isolate (SRFA)	5.52	NA	NA	NA	NA	300-500	1.96
Sharpless 2014	Suwannee River Fulvic Acid (SRFA) 59 h	IHSS Isolate (SRFA)	6.38	NA	NA	NA	NA	300-500	1.99
Sharpless 2014	Nordic Aquatic Fulvic Acid (NAFA) 0 h	IHSS Isolate (NFA)	3.80	NA	NA	NA	NA	300-500	1.42
Sharpless 2014	Nordic Aquatic Fulvic Acid (NAFA) 11 h	IHSS Isolate (NFA)	4.44	NA	NA	NA	NA	300-500	1.58
Sharpless 2014	Nordic Aquatic Fulvic Acid (NAFA) 35 h	IHSS Isolate (NFA)	5.25	NA	NA	NA	NA	300-500	1.67
Sharpless 2014	Nordic Aquatic Fulvic Acid (NAFA) 59 h	IHSS Isolate (NFA)	5.97	NA	NA	NA	NA	300-500	1.74
Sharpless 2014	Elliot Soil Humic Acid (ESHA) 0 h	IHSS Isolate (ESHA)	2.29	NA	NA	NA	NA	300-500	2.00
Sharpless 2014	Elliot Soil Humic Acid (ESHA) 11 h	IHSS Isolate (ESHA)	2.47	NA	NA	NA	NA	300-500	2.23
Sharpless 2014	Elliot Soil Humic Acid (ESHA) 35 h	IHSS Isolate (ESHA)	2.58	NA	NA	NA	NA	300-500	2.37
Sharpless 2014	Elliot Soil Humic Acid (ESHA) 59 h	IHSS Isolate (ESHA)	2.64	NA	NA	NA	NA	300-500	2.68
Zhang and Song 2014 [152]	Suwannee River humic acid (SRHA)	IHSS Isolate (SRHA)	NA	NA	NA	NA	NA	290-400	1.38
Zhang and Song 2014	Suwannee River fulvic acid (SRFA)	IHSS Isolate (SRFA)	NA	NA	NA	NA	NA	290-400	1.85
Zhang and Song 2014	Pony Lake fulvic acid (PLFA)	IHSS Isolate (PLFA)	NA	NA	NA	NA	NA	290-400	1.34
Zhang and Song 2014	Effluent	Whole Water (WWTP Effluent)	NA	2.07	NA	NA	NA	290-400	2.66

Table S18. Summary of $\Phi_{app,\ ^1O_2}$ and optical data (continued)

Reference	Sample ID	Sample Classification	*E2:E3*	$SUVA_{254}$ (L mg C^{-1}•m^{-1})	$S_{290–400}$ / $S_{300–400}$ / $S_{290–450}$ / $S_{295–400}$ (μm^{-1})	$S_{300–600}$ / $S_{300–500}$ / $S_{300–700}$ / $S_{275–500}$ (μm^{-1})	FI	Wavelength Range (nm)	$\Phi_{app,\ ^1O_2}$ ($\times 10^{-2}$ mol mol-photons^{-1})
Zhang and Song 2014	HPO	XAD Fraction (WWTP Effluent)	NA	NA	NA	NA	NA	290-400	3.24
Zhang and Song 2014	TPI	XAD Fraction (WWTP Effluent)	NA	NA	NA	NA	NA	290-400	2.45
Zhang and Song 2014	HPI	XAD Fraction (WWTP Effluent)	NA	NA	NA	NA	NA	290-400	8.16
Bodhipaksha and Sharpless 2015 [153]	Hockanum whole water upstream	Whole Water (River)	4.10	5.50	NA	NA	1.50	365	1.80
Bodhipaksha and Sharpless 2015	Hockanum whole water downstream	Whole Water (WW-Impacted River)	4.40	5.10	NA	NA	1.40	365	2.20
Bodhipaksha and Sharpless 2015	Hockanum OM isolate upstream	PPL Extract (River)	4.40	4.40	NA	NA	1.70	365	3.05
Bodhipaksha and Sharpless 2015	Hockanum OM isolate downstream	PPL Extract (WW-Impacted River)	4.90	2.70	NA	NA	2.30	365	3.46
Bodhipaksha and Sharpless 2015	Hockanum 2012 DOM isolate upstream	PPL Extract (River)	5.30	2.60	NA	NA	1.20	365	4.40
Bodhipaksha and Sharpless 2015	Hockanum 2012 EfOM isolate	PPL Extract (WWTP Effluent)	7.20	2.00	NA	NA	1.90	365	9.76
Bodhipaksha and Sharpless 2015	East Fork Little (EFL) Miami whole water upstream	Whole Water (River)	5.20	2.90	NA	NA	1.30	365	3.36
Bodhipaksha and Sharpless 2015	East Fork Little (EFL) Miami whole water downstream	Whole Water (WW-Impacted River)	5.20	2.80	NA	NA	1.50	365	3.40
Bodhipaksha and Sharpless 2015	East Fork Little (EFL) Miami OM isolate upstream	PPL Extract (River)	5.80	3.40	NA	NA	1.80	365	3.99
Bodhipaksha and Sharpless 2015	East Fork Little (EFL) Miami OM isolate downstream	PPL Extract (WW-Impacted River)	5.20	2.00	NA	NA	2.10	365	4.04
Bodhipaksha and Sharpless 2015	East Fork Little (EFL) Miami 2012 DOM isolate upstream	PPL Extract (River)	5.00	3.40	NA	NA	1.10	365	2.47
Bodhipaksha and Sharpless 2015	East Fork Little (EFL) Miami 2012 EfOM isolate	PPL Extract (WWTP Effluent)	4.60	2.50	NA	NA	1.40	365	3.08
Bodhipaksha and Sharpless 2015	Pomperaug whole water upstream	Whole Water (River)	4.30	3.60	NA	NA	1.20	365	2.30
Bodhipaksha and Sharpless 2015	Pomperaug whole water downstream	Whole Water (WW-Impacted River)	4.30	3.90	NA	NA	1.20	365	2.81
Bodhipaksha and Sharpless 2015	Pomperaug OM isolate upstream	PPL Extract (River)	5.20	3.50	NA	NA	1.30	365	2.74
Bodhipaksha and Sharpless 2015	Pomperaug OM isolate downstream	PPL Extract (WW-Impacted River)	5.10	3.40	NA	NA	1.40	365	2.21
Bodhipaksha and Sharpless 2015	Pomperaug 2013 DOM isolate upstream	PPL Extract (River)	4.30	1.60	NA	NA	1.80	365	3.00
Bodhipaksha and Sharpless 2015	Pomperaug 2013 EfOM isolate	PPL Extract (WWTP Effluent)	6.00	0.80	NA	NA	2.20	365	5.00
Bodhipaksha and Sharpless 2015	Pomperaug 2012 DOM isolate upstream	PPL Extract (River)	4.70	1.30	NA	NA	1.90	365	3.60
Bodhipaksha and Sharpless 2015	Pomperaug 2011 DOM isolate upstream	PPL Extract (River)	3.90	1.80	NA	NA	1.50	365	2.80
Bodhipaksha and Sharpless 2015	Pony Lake fulvic acid (PLFA)	IHSS Isolate (PLFA)	4.76	NA	NA	NA	NA	365	2.65
Bodhipaksha and Sharpless 2015	Suwannee River natural organic matter (SRNOM)	IHSS Isolate (SRNOM)	4.30	NA	NA	NA	NA	365	1.97
Bodhipaksha and Sharpless 2015	Suwannee River fulvic acid (SRFA)	IHSS Isolate (SRFA)	4.74	NA	NA	NA	NA	365	2.43
Bodhipaksha and Sharpless 2015	Suwannee River humic acid (SRHA)	IHSS Isolate (SRHA)	3.96	NA	NA	NA	NA	365	1.72
Bodhipaksha and Sharpless 2015	Nordic aquatic fulvic acid (NFA)	IHSS Isolate (NFA)	3.27	NA	NA	NA	NA	365	1.07
Bodhipaksha and Sharpless 2015	Nordic aquatic humic acid (NHA)	IHSS Isolate (NHA)	3.40	NA	NA	NA	NA	365	1.41
Bodhipaksha and Sharpless 2015	Wakish Peat humic acid (WPHA)	IHSS Isolate (WPHA)	3.04	NA	NA	NA	NA	365	1.15
Chu and McNeill 2015 [98]	Suwannee River natural organic matter (SRNOM)	IHSS Isolate (SRNOM)	NA	NA	NA	NA	NA	365	1.00
Chu and McNeill 2015	Suwannee River natural organic matter (SRNOM)	IHSS Isolate (SRNOM)	NA	NA	NA	NA	NA	365	0.97
Chu and McNeill 2015	Suwannee River natural organic matter (SRNOM)	IHSS Isolate (SRNOM)	NA	NA	NA	NA	NA	365	0.88
Chu and McNeill 2015	Suwannee River natural organic matter (SRNOM)	IHSS Isolate (SRNOM)	NA	NA	NA	NA	NA	365	0.77
Marchisio and Vione 2015 [154]	Avigliana Grande 313 nm	Whole Water (Lake)	11.10	NA	21.80	NA	NA	313	1.09
Marchisio and Vione 2015	Avigliana Grande 365 nm	Whole Water (Lake)	11.10	NA	21.80	NA	NA	365	1.07
Marchisio and Vione 2015	Avigliana Grande 420 nm	Whole Water (Lake)	11.10	NA	21.80	NA	NA	420	0.83
Marchisio and Vione 2015	Candia 313 nm	Whole Water (Lake)	9.20	NA	19.70	NA	NA	313	0.81
Marchisio and Vione 2015	Candia 365 nm	Whole Water (Lake)	9.20	NA	19.70	NA	NA	365	0.69
Marchisio and Vione 2015	Candia 420 nm	Whole Water (Lake)	9.20	NA	19.70	NA	NA	420	0.62
Marchisio and Vione 2015	Viverone 313 nm	Whole Water (Lake)	11.30	NA	20.70	NA	NA	313	0.93
Marchisio and Vione 2015	Viverone 365 nm	Whole Water (Lake)	11.30	NA	20.70	NA	NA	365	0.98
Marchisio and Vione 2015	Viverone 420 nm	Whole Water (Lake)	11.30	NA	20.70	NA	NA	420	0.46
Marchisio and Vione 2015	Balma 313 nm	Whole Water (Lake)	5.50	NA	15.10	NA	NA	313	1.68
Marchisio and Vione 2015	Balma 365 nm	Whole Water (Lake)	5.50	NA	15.10	NA	NA	365	0.56
Marchisio and Vione 2015	Balma 420 nm	Whole Water (Lake)	5.50	NA	15.10	NA	NA	420	0.40
Marchisio and Vione 2015	Sottano della Sella 313 nm	Whole Water (Lake)	4.60	NA	13.70	NA	NA	313	1.16
Marchisio and Vione 2015	Sottano della Sella 365 nm	Whole Water (Lake)	4.60	NA	13.70	NA	NA	365	0.69
Fu and Zhu 2016 [155]	DBC_Bamboo	DBC (pyDOM)	5.47	NA	NA	NA	NA	290-400	4.07
Fu and Zhu 2016	DBC_Bamboo_NaBH4 reduced	DBC (pyDOM, NaBH4)	NA	NA	NA	NA	NA	290-400	3.18
Fu and Zhu 2016	SRHA	IHSS Isolate (SRHA)	NA	NA	NA	NA	NA	290-400	1.57

Table S18. Summary of $\Phi_{app, {}^1O_2}$ and optical data (continued)

Reference	Sample ID	Sample Classification	*E2:E3*	SUVA$_{254}$ (L mg C^{-1}•m^{-1})	$S_{290–400}$ / $S_{300–400}$ / $S_{290–450}$ / $S_{295–400}$ (μm^{-1})	$S_{300–600}$ / $S_{300–500}$ / $S_{300–700}$ / $S_{275–500}$ (μm^{-1})	FI	Wavelength Range (nm)	$\Phi_{app, {}^1O_2}$ (×10^{-2} mol mol-photons^{-1})
McCabe and Arnold 2016 [34]	P1-July-2012_Semi-permanent	Whole Water (Wetland)	14.20	1.80	NA	23.46	NA	275-400	9.50
McCabe and Arnold 2016	P1-Nov-2012_Semi-permanent	Whole Water (Wetland)	16.30	1.80	NA	24.45	NA	275-400	10.90
McCabe and Arnold 2016	P1-May-2013_Semi-permanent	Whole Water (Wetland)	14.60	2.00	NA	23.39	NA	275-400	8.40
McCabe and Arnold 2016	P1-July-2013_Semi-permanent	Whole Water (Wetland)	15.40	2.00	NA	23.64	NA	275-400	10.40
McCabe and Arnold 2016	P1-Nov-2013_Semi-permanent	Whole Water (Wetland)	15.40	1.80	NA	23.63	NA	275-400	9.60
McCabe and Arnold 2016	P1-May-2014_Semi-permanent	Whole Water (Wetland)	12.40	1.70	NA	21.89	NA	275-400	7.40
McCabe and Arnold 2016	P1-Aug-2014_Semi-permanent	Whole Water (Wetland)	11.50	2.10	NA	20.37	NA	275-400	5.50
McCabe and Arnold 2016	P1-Nov-2014_Semi-permanent	Whole Water (Wetland)	13.10	1.90	NA	21.97	NA	275-400	6.60
McCabe and Arnold 2016	P7-July-2012_Semi-permanent	Whole Water (Wetland)	10.60	1.70	NA	20.10	NA	275-400	6.30
McCabe and Arnold 2016	P7-Nov-2012_Semi-permanent	Whole Water (Wetland)	12.50	1.60	NA	22.57	NA	275-400	9.00
McCabe and Arnold 2016	P7-May-2013_Semi-permanent	Whole Water (Wetland)	11.70	1.80	NA	21.50	NA	275-400	6.90
McCabe and Arnold 2016	P7-July-2013_Semi-permanent	Whole Water (Wetland)	11.10	1.80	NA	20.94	NA	275-400	7.50
McCabe and Arnold 2016	P7-Nov-2013_Semi-permanent	Whole Water (Wetland)	10.50	1.90	NA	20.01	NA	275-400	5.80
McCabe and Arnold 2016	P7-May-2014_Semi-permanent	Whole Water (Wetland)	10.90	2.00	NA	20.93	NA	275-400	6.10
McCabe and Arnold 2016	P7-Aug-2014_Semi-permanent	Whole Water (Wetland)	10.80	2.00	NA	20.47	NA	275-400	6.70
McCabe and Arnold 2016	P7-Nov-2014_Semi-permanent	Whole Water (Wetland)	11.80	1.90	NA	21.77	NA	275-400	7.60
McCabe and Arnold 2016	P8-July-2012_Semi-permanent	Whole Water (Wetland)	8.60	2.20	NA	18.89	NA	275-400	4.80
McCabe and Arnold 2016	P8-Nov-2012_Semi-permanent	Whole Water (Wetland)	10.60	2.10	NA	21.08	NA	275-400	6.40
McCabe and Arnold 2016	P8-May-2013_Semi-permanent	Whole Water (Wetland)	7.70	2.90	NA	18.70	NA	275-400	4.70
McCabe and Arnold 2016	P8-July-2013 _Semi-permanent	Whole Water (Wetland)	8.60	2.70	NA	19.65	NA	275-400	5.40
McCabe and Arnold 2016	P8-Nov-2013_Semi-permanent	Whole Water (Wetland)	9.70	2.20	NA	20.85	NA	275-400	5.30
McCabe and Arnold 2016	P8-May-2014_Semi-permanent	Whole Water (Wetland)	7.40	2.50	NA	17.99	NA	275-400	3.90
McCabe and Arnold 2016	P8-Aug-2014_Semi-permanent	Whole Water (Wetland)	8.10	2.70	NA	18.68	NA	275-400	4.90
McCabe and Arnold 2016	P8-Nov-2014_Semi-permanent	Whole Water (Wetland)	10.00	2.40	NA	21.06	NA	275-400	6.10
McCabe and Arnold 2016	T9-July-2012_Temporary	Whole Water (Wetland)	9.00	2.40	NA	20.41	NA	275-400	4.70
McCabe and Arnold 2016	T9-May-2013_Temporary	Whole Water (Wetland)	6.10	3.40	NA	17.17	NA	275-400	3.50
McCabe and Arnold 2016	T9-July-2013_Temporary	Whole Water (Wetland)	6.60	3.40	NA	18.10	NA	275-400	4.00
McCabe and Arnold 2016	T9-Nov-2013_Temporary	Whole Water (Wetland)	7.20	2.70	NA	18.34	NA	275-400	5.50
McCabe and Arnold 2016	T9-May-2014_Temporary	Whole Water (Wetland)	6.50	3.20	NA	17.45	NA	275-400	4.00
McCabe and Arnold 2016	T9-Aug-2014_Temporary	Whole Water (Wetland)	6.90	3.40	NA	17.71	NA	275-400	3.80
McCabe and Arnold 2016	T9-Nov-2014_Temporary	Whole Water (Wetland)	9.70	2.60	NA	21.21	NA	275-400	5.90
McCabe and Arnold 2016	R1-May-2013_Temporary	Whole Water (Wetland)	5.40	3.40	NA	15.94	NA	275-400	2.00
McCabe and Arnold 2016	R1-July-2013_Temporary	Whole Water (Wetland)	6.10	3.50	NA	17.22	NA	275-400	3.00
McCabe and Arnold 2016	R1-Nov-2013_Temporary	Whole Water (Wetland)	7.90	2.90	NA	19.38	NA	275-400	4.80
McCabe and Arnold 2016	R1-May-2014_Temporary	Whole Water (Wetland)	6.30	3.70	NA	17.43	NA	275-400	3.50
McCabe and Arnold 2016	R1-Aug-2014_Temporary	Whole Water (Wetland)	5.90	3.80	NA	16.69	NA	275-400	2.80
McCabe and Arnold 2016	R1-Nov-2014_Temporary	Whole Water (Wetland)	7.00	3.40	NA	18.41	NA	275-400	4.30
McCabe and Arnold 2016	R2-May-2013_Temporary	Whole Water (Wetland)	4.70	4.40	NA	15.01	NA	275-400	1.50
McCabe and Arnold 2016	R2-July-2013_Temporary	Whole Water (Wetland)	5.90	3.30	NA	17.08	NA	275-400	3.60
McCabe and Arnold 2016	R2-Nov-2013_Temporary	Whole Water (Wetland)	5.60	3.80	NA	16.71	NA	275-400	2.50
McCabe and Arnold 2016	R2-May-2014_Temporary	Whole Water (Wetland)	5.00	5.10	NA	15.56	NA	275-400	2.20
McCabe and Arnold 2016	R2-Aug-2014_Temporary	Whole Water (Wetland)	5.70	3.80	NA	16.43	NA	275-400	3.20
McCabe and Arnold 2016	D1-Mar-2013_Temporary	Whole Water (Wetland)	5.50	2.40	NA	16.70	NA	275-400	4.80
McCabe and Arnold 2016	D1-Jun-2014_1_Temporary	Whole Water (Wetland)	4.30	6.00	NA	14.50	NA	275-400	6.30
McCabe and Arnold 2016	D1-Jun-2014_2_Temporary	Whole Water (Wetland)	4.50	4.70	NA	14.92	NA	275-400	4.50
McKay and Rosario-Ortiz 2016 [156]	Boulder Creek (BC)	Whole Water (WW-Impacted	5.56	NA	NA	NA	NA	290-400	3.65
McKay and Rosario-Ortiz 2016	BC base modification	Whole Water (WW-Impacted Stream, [illegible]	5.61	NA	NA	NA	NA	290-400	3.82
McKay and Rosario-Ortiz 2016	Boulder Wastewater (BWW)	Whole Water (WWTP Effluent)	5.25	NA	NA	NA	NA	290-400	2.77
McKay and Rosario-Ortiz 2016	BWW base modification	Whole Water (WWTP Effluent, Base)	5.79	NA	NA	NA	NA	290-400	3.58
McKay and Rosario-Ortiz 2016	Orange County Water District (OCWD)	Whole Water (WWTP Effluent)	5.01	NA	NA	NA	NA	290-400	2.61
McKay and Rosario-Ortiz 2016	OCWD base modification	Whole Water (WWTP Effluent, Base)	5.14	NA	NA	NA	NA	290-400	3.71

Table S18. Summary of $\Phi_{app, ^1O_2}$ and optical data (continued)

Reference	Sample ID	Sample Classification	*E2:E3*	$SUVA_{254}$ (L mg C^{-1}•m^{-1})	$S_{290–400}$ / $S_{300–400}$ / $S_{290–450}$ / $S_{295–400}$ (μm^{-1})	$S_{300–600}$ / $S_{300–500}$ / $S_{300–700}$ / $S_{275–500}$ (μm^{-1})	FI	Wavelength Range (nm)	$\Phi_{app, ^1O_2}$ ($\times 10^{-2}$ mol mol-photons^{-1})
McKay and Rosario-Ortiz 2016	Longmont Wastewater (LM)	Whole Water (WWTP Effluent)	4.59	NA	NA	NA	NA	290-400	2.36
McKay and Rosario-Ortiz 2016	LM Longmont 30 mg alum/L	Whole Water (WWTP Effluent, Alum)	4.88	NA	NA	NA	NA	290-400	2.78
McKay and Rosario-Ortiz 2016	LM Longmont 60 mg alum/L	Whole Water (WWTP Effluent, Alum)	5.21	NA	NA	NA	NA	290-400	3.00
McKay and Rosario-Ortiz 2016	LM Longmont 90 mg alum/L	Whole Water (WWTP Effluent, Alum)	5.37	NA	NA	NA	NA	290-400	3.52
McKay and Rosario-Ortiz 2016	LM Longmont 120 mg alum/L	Whole Water (WWTP Effluent, Alum)	5.51	NA	NA	NA	NA	290-400	3.63
McKay and Rosario-Ortiz 2016	SRFA < 5 kDa	UF Fraction (SRFA)	5.46	NA	NA	NA	NA	290-400	2.98
McKay and Rosario-Ortiz 2016	SRFA < 5 kDa reduction	UF Fraction (SRFA, NaBH4)	7.37	NA	NA	NA	NA	290-400	2.56
McKay and Rosario-Ortiz 2016	Suwannee River fulvic acid (SRFA)	IHSS Isolate (SRFA)	4.73	NA	NA	NA	NA	290-400	1.81
McKay and Rosario-Ortiz 2016	SRFA reduction	IHSS Isolate (SRFA, NaBH4)	6.36	NA	NA	NA	NA	290-400	1.64
McKay and Rosario-Ortiz 2016	SRFA > 5 kDa	UF Fraction (SRFA)	4.57	NA	NA	NA	NA	290-400	1.54
McKay and Rosario-Ortiz 2016	SRFA > 5 kDa reduction	UF Fraction (SRFA, NaBH4)	6.33	NA	NA	NA	NA	290-400	1.52
Bodhipaksha and MacKay 2017 [157]	Hockanum River Upstream	Whole Water (River)	4.30	3.50	NA	NA	NA	290-450	4.90
Bodhipaksha and MacKay 2017	Vernon Wastewater Treatment Plant Effluent	Whole Water (WWTP Effluent)	7.00	2.00	NA	NA	NA	290-450	18.00
Bodhipaksha and MacKay 2017	Hockanum River Downstream WWTP	Whole Water (WW-Impacted River)	4.60	3.30	NA	NA	NA	290-450	6.30
Maizel and Remucal 2017a [158]	Pony Lake fulvic acid (PLFA)	IHSS Isolate (PLFA)	4.74	3.32	NA	NA	NA	365	1.90
Maizel and Remucal 2017a	PLFA < 3 kDa	UF Fraction (PLFA)	5.86	2.66	NA	NA	NA	365	3.30
Maizel and Remucal 2017a	PLFA 3-5 kDa	UF Fraction (PLFA)	5.31	2.37	NA	NA	NA	365	2.30
Maizel and Remucal 2017a	PLFA 5-10 kDa	UF Fraction (PLFA)	4.58	3.38	NA	NA	NA	365	1.67
Maizel and Remucal 2017a	PLFA > 10 kDa	UF Fraction (PLFA)	3.79	5.12	NA	NA	NA	365	1.20
Maizel and Remucal 2017a	Suwannee River fulvic acid (SRFA)	IHSS Isolate (SRFA)	4.58	4.83	NA	NA	NA	365	1.50
Maizel and Remucal 2017a	SRFA < 3 kDa	UF Fraction (SRFA)	5.30	3.80	NA	NA	NA	365	3.10
Maizel and Remucal 2017a	SRFA 3-5 kDa	UF Fraction (SRFA)	5.06	3.68	NA	NA	NA	365	2.80
Maizel and Remucal 2017a	SRFA 5-10 kDa	UF Fraction (SRFA)	4.66	4.96	NA	NA	NA	365	0.80
Maizel and Remucal 2017a	SRFA >10 kDa	UF Fraction (SRFA)	3.91	5.93	NA	NA	NA	365	0.88
Maizel and Remucal 2017b [159]	Crystal Bog (CB)	Whole Water (Lake)	4.54	3.20	NA	NA	NA	365	0.54
Maizel and Remucal 2017b	Trout Bog (TB)	Whole Water (Lake)	4.85	3.81	NA	NA	NA	365	0.55
Maizel and Remucal 2017b	Allequash L. (AL)	Whole Water (Lake)	5.85	2.72	NA	NA	NA	365	0.93
Maizel and Remucal 2017b	Big Muskellunge L. (BM)	Whole Water (Lake)	9.85	0.90	NA	NA	NA	365	1.62
Maizel and Remucal 2017b	Crystal L. (CR)	Whole Water (Lake)	9.17	0.59	NA	NA	NA	365	1.89
Maizel and Remucal 2017b	Sparkling L. (SP)	Whole Water (Lake)	9.17	1.20	NA	NA	NA	365	1.40
Maizel and Remucal 2017b	Trout L. (TR)	Whole Water (Lake)	9.80	1.25	NA	NA	NA	365	1.63
Maizel and Remucal 2017c [160]	Big Muskellunge Lake ambient	Whole Water (Lake)	10.51	0.96	NA	19.60	NA	365	1.32
Maizel and Remucal 2017c	Sparkling Lake ambient	Whole Water (Lake)	9.63	1.38	NA	19.80	NA	365	1.27
Maizel and Remucal 2017c	Allequash Lake ambient	Whole Water (Lake)	5.97	3.06	NA	17.00	NA	365	1.01
Maizel and Remucal 2017c	St. Louis River ambient	Whole Water (River)	4.68	4.43	NA	15.20	NA	365	1.22
Maizel and Remucal 2017c	Toivola Swamp ambient	Whole Water (Wetland)	5.07	3.97	NA	15.60	NA	365	1.37
Maizel and Remucal 2017c	Trout Bog ambient	Whole Water (Lake)	4.86	3.72	NA	15.20	NA	365	0.74
Maizel and Remucal 2017c	WLSSD Wastewater Eff ambient	Whole Water (WWTP Effluent)	5.98	2.78	NA	17.10	NA	365	1.44
Maizel and Remucal 2017c	MMSD Wastewater Eff ambient	Whole Water (WWTP Effluent)	5.36	2.46	NA	16.40	NA	365	1.42
Maizel and Remucal 2017c	Big Muskellunge Lake standardized	Whole Water (Lake)	NA	NA	NA	NA	NA	365	1.42
Maizel and Remucal 2017c	Sparkling Lake standardized	Whole Water (Lake)	NA	NA	NA	NA	NA	365	1.30
Maizel and Remucal 2017c	Allequash Lake standardized	Whole Water (Lake)	NA	NA	NA	NA	NA	365	0.98
Maizel and Remucal 2017c	St. Louis River standardized	Whole Water (River)	NA	NA	NA	NA	NA	365	0.87
Maizel and Remucal 2017c	Toivola Swamp standardized	Whole Water (Wetland)	NA	NA	NA	NA	NA	365	0.82
Maizel and Remucal 2017c	Trout Bog standardized	Whole Water (Lake)	NA	NA	NA	NA	NA	365	0.63
Maizel and Remucal 2017c	WLSSD Wastewater Eff standardized	Whole Water (WWTP Effluent)	NA	NA	NA	NA	NA	365	1.63
Maizel and Remucal 2017c	MMSD Wastewater Eff standardized	Whole Water (WWTP Effluent)	NA	NA	NA	NA	NA	365	1.58
Maizel and Remucal 2017c	Big Muskellunge Lake SPE	PPL Extract (Lake)	10.43	1.24	NA	21.10	NA	365	1.42
Maizel and Remucal 2017c	Sparkling Lake SPE	PPL Extract (Lake)	9.93	1.50	NA	21.10	NA	365	1.78
Maizel and Remucal 2017c	Allequash Lake SPE	PPL Extract (Lake)	5.65	3.38	NA	16.50	NA	365	1.13
Maizel and Remucal 2017c	St. Louis River SPE	PPL Extract (Lake)	5.04	4.15	NA	15.80	NA	365	1.25

Table S18. Summary of $\Phi_{app, {}^1O_2}$ and optical data (continued)

Reference	Sample ID	Sample Classification	*E2:E3*	$SUVA_{254}$ (L mg C^{-1}•m^{-1})	$S_{290–400}$ / $S_{300–400}$ / $S_{290–450}$ / $S_{295–400}$ (μm^{-1})	$S_{300–600}$ / $S_{300–500}$ / $S_{300–700}$ / $S_{275–500}$ (μm^{-1})	FI	Wavelength Range (nm)	$\Phi_{app, {}^1O_2}$ ($\times 10^{-2}$ mol mol-photons^{-1})
Maizel and Remucal 2017c	Toivola Swamp SPE	PPL Extract (Lake)	5.40	3.75	NA	16.30	NA	365	1.01
Maizel and Remucal 2017c	Trout Bog SPE	PPL Extract (Lake)	4.84	3.79	NA	15.30	NA	365	0.82
Maizel and Remucal 2017c	WLSSD Wastewater Eff SPE	PPL Extract (WWTP Effluent)	6.34	2.83	NA	17.90	NA	365	1.87
Maizel and Remucal 2017c	MMSD Wastewater Eff SPE	PPL Extract (WWTP Effluent)	4.73	2.60	NA	14.90	NA	365	1.68
McKay and Rosario-Ortiz 2017 [161]	FB21_Sep Everglades estuary	Whole Water (Estuary)	10.60	1.02	NA	20.22	NA	290-400	8.88
McKay and Rosario-Ortiz 2017	FB21_Oct Everglades estuary	Whole Water (Estuary)	16.90	1.01	NA	24.16	NA	290-400	10.43
McKay and Rosario-Ortiz 2017	FB21_Nov Everglades estuary	Whole Water (Estuary)	13.30	0.92	NA	22.56	NA	290-400	7.77
McKay and Rosario-Ortiz 2017	SRS2_Sep Everglades freshwater marsh	Whole Water (Estuary)	7.10	2.89	NA	18.16	NA	290-400	5.39
McKay and Rosario-Ortiz 2017	SRS2_Oct Everglades freshwater marsh	Whole Water (Estuary)	7.70	2.25	NA	18.97	NA	290-400	6.21
McKay and Rosario-Ortiz 2017	SRS2_Nov Everglades freshwater marsh	Whole Water (Estuary)	8.20	2.27	NA	19.47	NA	290-400	7.30
McKay and Rosario-Ortiz 2017	SRS4_Sep Everglades mangrove estuarine	Whole Water (Estuary)	5.50	3.52	NA	15.91	NA	290-400	3.69
McKay and Rosario-Ortiz 2017	SRS4_Oct Everglades mangrove estuarine	Whole Water (Estuary)	5.80	2.86	NA	16.31	NA	290-400	4.50
McKay and Rosario-Ortiz 2017	SRS4_Nov Everglades mangrove estuarine	Whole Water (Estuary)	6.40	3.15	NA	17.19	NA	290-400	4.47
McKay and Rosario-Ortiz 2017	SRS6_Sep Everglades mangrove estuarine	Whole Water (Estuary)	5.30	3.69	NA	15.59	NA	290-400	3.09
McKay and Rosario-Ortiz 2017	SRS6_Oct Everglades mangrove estuarine	Whole Water (Estuary)	5.50	3.51	NA	15.91	NA	290-400	3.31
McKay and Rosario-Ortiz 2017	SRS6_Nov Everglades mangrove estuarine	Whole Water (Estuary)	6.00	3.49	NA	16.78	NA	290-400	3.59
McKay and Rosario-Ortiz 2017	TS2_Sep Everglades freshwater marsh	Whole Water (Estuary)	8.30	2.16	NA	19.72	NA	290-400	6.80
McKay and Rosario-Ortiz 2017	TS2_Oct Everglades freshwater marsh	Whole Water (Estuary)	8.80	1.87	NA	20.28	NA	290-400	6.31
McKay and Rosario-Ortiz 2017	TS2_Nov Everglades freshwater marsh	Whole Water (Estuary)	9.30	1.78	NA	20.31	NA	290-400	7.29
McKay and Rosario-Ortiz 2017	TS3_Sep Everglades freshwater marsh	Whole Water (Estuary)	7.70	2.36	NA	19.13	NA	290-400	6.07
McKay and Rosario-Ortiz 2017	TS3_Oct Everglades freshwater marsh	Whole Water (Estuary)	8.40	2.03	NA	19.56	NA	290-400	6.94
McKay and Rosario-Ortiz 2017	TS3_Nov Everglades freshwater marsh	Whole Water (Estuary)	8.90	2.10	NA	19.97	NA	290-400	6.42
McKay and Rosario-Ortiz 2017	TS7_Sep Everglades mangrove estuarine	Whole Water (Estuary)	5.90	3.25	NA	16.41	NA	290-400	2.70
McKay and Rosario-Ortiz 2017	TS7_Oct Everglades mangrove estuarine	Whole Water (Estuary)	5.40	3.32	NA	15.75	NA	290-400	2.49
McKay and Rosario-Ortiz 2017	TS7_Nov Everglades mangrove estuarine	Whole Water (Estuary)	10.00	2.13	NA	20.41	NA	290-400	4.86
McKay and Rosario-Ortiz 2017	Suwannee River natural organic matter (SRNOM)	IHSS Isolate (SRNOM)	NA	NA	NA	NA	NA	290-400	2.66
McKay and Rosario-Ortiz 2017	TS7_Sep Everglades mangrove estuarine	PPL Extract (Estuary)	NA	NA	NA	NA	NA	290-400	3.31
McKay and Rosario-Ortiz 2017	SRS6_Nov Everglades mangrove estuarine	PPL Extract (Estuary)	NA	NA	NA	NA	NA	290-400	4.07
McKay and Rosario-Ortiz 2017	SRS6_Oct Everglades mangrove estuarine	PPL Extract (Estuary)	NA	NA	NA	NA	NA	290-400	4.92
McKay and Rosario-Ortiz 2017	SRS4_Nov Everglades mangrove estuarine	PPL Extract (Estuary)	NA	NA	NA	NA	NA	290-400	5.54
McKay and Rosario-Ortiz 2017	SRS2_Nov Everglades freshwater marsh	PPL Extract (Estuary)	NA	NA	NA	NA	NA	290-400	7.75
McKay and Rosario-Ortiz 2017	TS3_Oct Everglades freshwater marsh	PPL Extract (Estuary)	NA	NA	NA	NA	NA	290-400	7.63
McKay and Rosario-Ortiz 2017	TS2_Oct Everglades freshwater marsh	PPL Extract (Estuary)	NA	NA	NA	NA	NA	290-400	8.16
McKay and Rosario-Ortiz 2017	FB21_Oct Everglades estuary	PPL Extract (Estuary)	NA	NA	NA	NA	NA	290-400	8.11
Pozdnyakov 2017 [162]	CS (Henan Changsheng Industrial Corporation) pH 3	XAD Fraction (Soil)	NA	NA	NA	NA	NA	532	0.44
Pozdnyakov 2017	AL (Aladdin Industrial Corporation) pH 3	XAD Fraction (Soil)	NA	NA	NA	NA	NA	532	0.06
Pozdnyakov 2017	NA (Nordic Acid, IHSS reference, 1R105F) pH 3	IHSS Isolate (NFA)	NA	NA	NA	NA	NA	532	0.19
Pozdnyakov 2017	WP (Waskish Peat, IHSS reference, 1R107F) pH 3	IHSS Isolate (WPFA)	NA	NA	NA	NA	NA	532	0.10
Pozdnyakov 2017	CS (Henan Changsheng Industrial Corporation) pH 10	XAD Fraction (Soil)	NA	NA	NA	NA	NA	532	1.70
Pozdnyakov 2017	AL (Aladdin Industrial Corporation) pH 10	XAD Fraction (Soil)	NA	NA	NA	NA	NA	532	0.29
Pozdnyakov 2017	NA (Nordic Acid, IHSS reference, 1R105F) pH 10	IHSS Isolate (NFA)	NA	NA	NA	NA	NA	532	0.58
Pozdnyakov 2017	WP (Waskish Peat, IHSS reference, 1R107F) pH 10	IHSS Isolate (WPFA)	NA	NA	NA	NA	NA	532	0.36
Silva and Rosario-Ortiz 2017 [163]	SRNOM (1R101N)	IHSS Isolate (SRNOM)	3.57	4.22	NA	12.50	NA	290-400	0.93
Silva and Rosario-Ortiz 2017	CVT230	WEOM (Biowaste)	2.45	6.26	NA	8.50	NA	290-400	0.88
Silva and Rosario-Ortiz 2017	CVDF110	WEOM (Biowaste)	2.47	5.49	NA	8.40	NA	290-400	0.80
Tenorio and Strathmann 2017 [164]	SRNOM + Fe + EDTA	IHSS Isolate (SRNOM)	NA	NA	NA	NA	NA	310-400	1.42
Tenorio and Strathmann 2017	SRNOM + Fe + EDTA	IHSS Isolate (SRNOM)	NA	NA	NA	NA	NA	310-400	1.73
Tenorio and Strathmann 2017	SRNOM + Fe + EDTA	IHSS Isolate (SRNOM)	NA	NA	NA	NA	NA	310-400	2.15
Tenorio and Strathmann 2017	SRNOM + Fe + EDTA	IHSS Isolate (SRNOM)	NA	NA	NA	NA	NA	310-400	2.53
Tenorio and Strathmann 2017	Extracellular organic matter during growth phase IV (data from Fig. 5)	EOM (C. reinhardtii)	NA	NA	NA	NA	NA	310-400	1.25
Tenorio and Strathmann 2017	Extracellular organic matter during growth phase III (end) (data from Fig. 5)	EOM (C. reinhardtii)	NA	NA	NA	NA	NA	310-400	1.31

Table S18. Summary of $\Phi_{app, {}^1O_2}$ and optical data (continued)									
Reference	Sample ID	Sample Classification	$E2:E3$	$SUVA_{254}$ (L mg C^{-1}•m^{-1})	$S_{290–400}$ / $S_{300–400}$ / $S_{290–450}$ / $S_{295–400}$ (μm^{-1})	$S_{300–600}$ / $S_{300–500}$ / $S_{300–700}$ / $S_{275–500}$ (μm^{-1})	FI	Wavelength Range (nm)	$\Phi_{app, {}^1O_2}$ ($\times 10^{-2}$ mol mol-photons^{-1})
Tenorio and Strathmann 2017	Extracellular organic matter during growth phase III (min) (data from Fig. 5)	EOM (C. reinhardtii)	NA	NA	NA	NA	NA	310-400	1.46
Tenorio and Strathmann 2017	Extracellular organic matter during growth phase II (end) (data from Fig. 5)	EOM (C. reinhardtii)	NA	NA	NA	NA	NA	310-400	1.59
Tenorio and Strathmann 2017	Extracellular organic matter during growth phase IV (data from Fig. 5)	EOM (C. reinhardtii)	NA	NA	NA	NA	NA	310-400	1.86
Tenorio and Strathmann 2017	Extracellular organic matter during growth phase IV (data from Fig. 5)	EOM (C. reinhardtii)	NA	NA	NA	NA	NA	310-400	2.73
Zhou and Song 2017 [165]	Effluent1	Whole Water (WWTP Effluent)	5.40	1.90	NA	NA	2.20	290-400	5.49
Zhou and Song 2017	HPO1	XAD Fraction (WWTP Effluent)	4.80	2.40	NA	NA	1.90	290-400	4.02
Zhou and Song 2017	TPI1	XAD Fraction (WWTP Effluent)	5.40	1.50	NA	NA	2.10	290-400	4.67
Zhou and Song 2017	HPI1	XAD Fraction (WWTP Effluent)	6.20	0.90	NA	NA	2.30	290-400	9.03
Zhou and Song 2017	Effluent2	Whole Water (WWTP Effluent)	5.50	1.90	NA	NA	2.10	290-400	6.11
Zhou and Song 2017	HPO2	XAD Fraction (WWTP Effluent)	4.80	2.30	NA	NA	1.90	290-400	4.00
Zhou and Song 2017	TPI2	XAD Fraction (WWTP Effluent)	5.60	1.40	NA	NA	2.00	290-400	5.29
Zhou and Song 2017	HPI2	XAD Fraction (WWTP Effluent)	6.10	0.90	NA	NA	2.30	290-400	8.61
Zhou and Song 2017	Suwannee River natural organic matter (SRNOM)	IHSS Isolate (SRNOM)	4.30	4.10	NA	NA	1.30	290-400	2.88
Zhou and Song 2017	Suwannee River humic acid (SRHA)	IHSS Isolate (SRHA)	3.40	4.60	NA	NA	1.10	290-400	2.05
Zhou and Song 2017	Suwannee River fulvic acid (SRFA)	IHSS Isolate (SRFA)	4.50	4.20	NA	NA	1.30	290-400	3.10
Zhou and Song 2017	Pony Lake fulvic acid (PLFA)	IHSS Isolate (PLFA)	5.00	2.20	NA	NA	1.50	290-400	4.51
Du and Zhu 2018 [166]	DBC_Corn	DBC (pyDOM)	6.57	2.16	NA	NA	1.59	290-400	3.47
Du and Zhu 2018	DBC_Purslane	DBC (pyDOM)	5.24	1.44	NA	NA	1.80	290-400	3.56
Du and Zhu 2018	DBC_Soybean	DBC (pyDOM)	9.71	3.41	NA	NA	1.58	290-400	6.14
Du and Zhu 2018	DBC_Rice	DBC (pyDOM)	5.14	3.41	NA	NA	1.57	290-400	3.92
Du and Zhu 2018	DBC_Sorghum	DBC (pyDOM)	9.32	2.63	NA	NA	1.49	290-400	5.81
Du and Zhu 2018	DBC_Bamboo	DBC (pyDOM)	8.67	5.63	NA	NA	1.44	290-400	5.32
Du and Zhu 2018	DBC_Wheat	DBC (pyDOM)	7.76	2.21	NA	NA	1.65	290-400	4.27
Du and Zhu 2018	DBC_Millet	DBC (pyDOM)	7.80	2.42	NA	NA	1.58	290-400	4.31
Du and Zhu 2018	DBC_Peanut	DBC (pyDOM)	4.79	5.25	NA	NA	1.39	290-400	4.03
Du and Zhu 2018	Suwannee River fulvic acid (SRFA)	IHSS Isolate (SRFA)	4.63	7.86	NA	NA	1.24	290-400	2.15
Du and Zhu 2018	Nordic aquatic natural organic matter (Nor NOM)	IHSS Isolate (NLNOM)	4.57	4.50	NA	NA	1.34	290-400	3.58
Du and Zhu 2018	Pony Lake fulvic acid (PLFA)	IHSS Isolate (PLFA)	5.66	2.83	NA	NA	1.46	290-400	3.12
Du and Zhu 2018	Upper Mississippi River NOM (UM NOM)	IHSS Isolate (UMRNOM)	5.77	4.00	NA	NA	1.57	290-400	3.28
Du and Zhu 2018	Pahokee peat humic acid standard (PP HA)	IHSS Isolate (PPHA)	3.18	7.31	NA	NA	0.72	290-400	1.81
Du and Zhu 2018	Suwannee River natural organic matter (SRNOM)	IHSS Isolate (SRNOM)	5.17	4.25	NA	NA	1.30	290-400	2.73
Du and Zhu 2018	Leonardit humic acid (Leo HA)	IHSS Isolate (LHA)	2.68	8.99	NA	NA	0.98	290-400	1.31
Du and Zhu 2018	Pahokee peat fulvic acid standard (PP FA)	IHSS Isolate (PPFA)	5.66	7.30	NA	NA	1.58	290-400	3.40
Du and Zhu 2018	Nordic aquatic fulvic acid reference (Nor FA)	IHSS Isolate (NFA)	4.75	5.77	NA	NA	1.18	290-400	1.28
Hong 2018 [167]	Oct2016sample (October 2016, Wuhan, China)	Whole Water (Rainwater)	7.45	NA	NA	NA	NA	290-400	1.92
Hong 2018	Nov2016sample (November 2016, Wuhan, China)	Whole Water (Rainwater)	4.39	NA	NA	NA	NA	290-400	0.94
Hong 2018	Dec2016sample (December 2016, Wuhan, China)	Whole Water (Rainwater)	6.28	NA	NA	NA	NA	290-400	1.66
Hong 2018	SRFA	IHSS Isolate (SRFA)	NA	NA	NA	NA	NA	290-400	1.71
Zhou and Chen 2018 [168]	Fresh water (L1) Yellow River main stream (L1)	Whole Water (River)	NA	NA	NA	NA	NA	280-400	7.67
Zhou and Chen 2018	Estuarine water (L2) Bohai Sea (L3)	Whole Water (Estuary)	NA	NA	NA	NA	NA	280-400	6.70
Zhou and Chen 2018	Seawater (L3) Guangli River estuary (L2)	Whole Water (Seawater)	NA	NA	NA	NA	NA	280-400	1.94
Zhou and Chen 2018	SRFA	IHSS Isolate (SRFA)	NA	NA	NA	NA	NA	280-400	2.83
Zhou and Chen 2018	SRHA	IHSS Isolate (SRHA)	NA	NA	NA	NA	NA	280-400	1.83
Zhou and Chen 2018	PLFA	IHSS Isolate (PLFA)	NA	NA	NA	NA	NA	280-400	4.96
Berg and Remucal 2019 [169]	Sand Creek_A	Whole Water (River)	4.32	5.20	NA	NA	NA	365	0.54
Berg and Remucal 2019	Meadowlands_B	Whole Water (River)	4.43	4.52	NA	NA	NA	365	0.69
Berg and Remucal 2019	River Inn_C	Whole Water (River)	4.67	4.33	NA	NA	NA	365	0.63
Berg and Remucal 2019	East Detroit_D	Whole Water (River)	4.74	4.26	NA	NA	NA	365	0.70
Berg and Remucal 2019	Munger Landing_E	Whole Water (River)	4.80	4.17	NA	NA	NA	365	0.85
Berg and Remucal 2019	WLSSD_F	Whole Water (WWTP Effluent)	6.33	2.40	NA	NA	NA	365	1.03
Berg and Remucal 2019	Blatnik Bridge_G	Whole Water (WW-Impacted River)	4.88	4.01	NA	NA	NA	365	0.73

Table S18. Summary of $\Phi_{app, {}^1O_2}$ and optical data (continued)

Reference	Sample ID	Sample Classification	*E2:E3*	$SUVA_{254}$ (L mg C^{-1}•m^{-1})	$S_{290–400}$ / $S_{300–400}$ / $S_{290–450}$ / $S_{295–400}$ (μm^{-1})	$S_{300–600}$ / $S_{300–500}$ / $S_{300–700}$ / $S_{275–500}$ (μm^{-1})	FI	Wavelength Range (nm)	$\Phi_{app, {}^1O_2}$ ($\times 10^{-2}$ mol mol-photons^{-1})
Berg and Remucal 2019	Wisconsin Point_H	Whole Water (Lake)	5.78	2.57	NA	NA	NA	365	0.79
Chen and Chen 2019 [112]	DOM-S1 (from polyculture of fish-shellfish ponds)	RO/ED Isolate (Seawater)	NA	NA	NA	NA	NA	280-500	1.24
Chen and Chen 2019	DOM-S2 (from tidal creek linking several mariculture ponds)	RO/ED Isolate (Seawater)	NA	NA	NA	NA	NA	280-500	0.69
Chen and Chen 2019	DOM-S3 (from offshore area)	RO/ED Isolate (Seawater)	NA	NA	NA	NA	NA	280-500	0.82
Chen and Chen 2019	SRNOM	IHSS Isolate (SRNOM)	NA	NA	NA	NA	NA	280-500	0.76
Leresche and Rosario-Ortiz 2019 [170]	Pony Lake fulvic acid (PLFA) pH 3 ozone 0 mmol O3 mmol C	IHSS Isolate (PLFA)	3.15	2.28	NA	16.56	NA	290-400	2.84
Leresche and Rosario-Ortiz 2019	PLFA pH 3 ozone 0.025 mmol O3 mmol C	IHSS Isolate (PLFA, O3)	4.55	2.02	NA	18.78	NA	290-400	5.62
Leresche and Rosario-Ortiz 2019	PLFA pH 3 ozone 0.055 mmol O3 mmol C	IHSS Isolate (PLFA, O3)	6.47	1.71	NA	20.22	NA	290-400	7.09
Leresche and Rosario-Ortiz 2019	PLFA pH 3 ozone 0.1 mmol O3 mmol C	IHSS Isolate (PLFA, O3)	7.90	1.48	NA	22.03	NA	290-400	8.36
Leresche and Rosario-Ortiz 2019	PLFA pH 3 ozone 0.15 mmol O3 mmol C	IHSS Isolate (PLFA, O3)	8.42	1.41	NA	22.03	NA	290-400	8.60
Leresche and Rosario-Ortiz 2019	PLFA pH 3 ozone 0.2 mmol O3 mmol C	IHSS Isolate (PLFA, O3)	9.29	1.30	NA	22.35	NA	290-400	9.46
Leresche and Rosario-Ortiz 2019	PLFA pH 3 ozone 0.25 mmol O3 mmol C	IHSS Isolate (PLFA, O3)	10.22	1.23	NA	23.24	NA	290-400	10.59
Leresche and Rosario-Ortiz 2019	PLFA pH 3 ozone 0.35 mmol O3 mmol C	IHSS Isolate (PLFA, O3)	11.67	1.16	NA	25.11	NA	290-400	12.68
Leresche and Rosario-Ortiz 2019	PLFA pH 3 ozone 0.5 mmol O3 mmol C	IHSS Isolate (PLFA, O3)	11.83	1.14	NA	24.39	NA	290-400	9.81
Leresche and Rosario-Ortiz 2019	PLFA pH 3 ozone 0.75 mmol O3 mmol C	IHSS Isolate (PLFA, O3)	13.86	1.05	NA	25.80	NA	290-400	11.52
Leresche and Rosario-Ortiz 2019	PLFA pH 3 ozone 1 mmol O3 mmol C	IHSS Isolate (PLFA, O3)	16.92	1.01	NA	25.94	NA	290-400	13.08
Leresche and Rosario-Ortiz 2019	PLFA pH 7 ozone 0 mmol O3 mmol C	IHSS Isolate (PLFA)	2.81	2.47	NA	16.04	NA	290-400	2.63
Leresche and Rosario-Ortiz 2019	PLFA pH 7 ozone 0.025 mmol O3 mmol C	IHSS Isolate (PLFA, O3)	3.82	2.15	NA	17.65	NA	290-400	3.15
Leresche and Rosario-Ortiz 2019	PLFA pH 7 ozone 0.055 mmol O3 mmol C	IHSS Isolate (PLFA, O3)	5.13	1.85	NA	19.44	NA	290-400	4.60
Leresche and Rosario-Ortiz 2019	PLFA pH 7 ozone 0.1 mmol O3 mmol C	IHSS Isolate (PLFA, O3)	7.99	1.47	NA	21.63	NA	290-400	5.12
Leresche and Rosario-Ortiz 2019	PLFA pH 7 ozone 0.15 mmol O3 mmol C	IHSS Isolate (PLFA, O3)	9.64	1.33	NA	22.60	NA	290-400	6.45
Leresche and Rosario-Ortiz 2019	PLFA pH 7 ozone 0.2 mmol O3 mmol C	IHSS Isolate (PLFA, O3)	11.25	1.21	NA	23.55	NA	290-400	9.38
Leresche and Rosario-Ortiz 2019	PLFA pH 7 ozone 0.25 mmol O3 mmol C	IHSS Isolate (PLFA, O3)	11.63	1.17	NA	23.61	NA	290-400	12.13
Leresche and Rosario-Ortiz 2019	PLFA pH 7 ozone 0.35 mmol O3 mmol C	IHSS Isolate (PLFA, O3)	14.71	1.06	NA	25.68	NA	290-400	15.83
Leresche and Rosario-Ortiz 2019	PLFA pH 7 ozone 0.5 mmol O3 mmol C	IHSS Isolate (PLFA, O3)	16.81	1.00	NA	26.60	NA	290-400	11.06
Leresche and Rosario-Ortiz 2019	PLFA pH 7 ozone 0.75 mmol O3 mmol C	IHSS Isolate (PLFA, O3)	20.65	0.93	NA	28.39	NA	290-400	13.49
Leresche and Rosario-Ortiz 2019	PLFA pH 7 ozone 1 mmol O3 mmol C	IHSS Isolate (PLFA, O3)	24.11	0.91	NA	29.94	NA	290-400	17.02
Leresche and Rosario-Ortiz 2019	SRFA pH 3 ozone 0 mmol O3 mmol C	IHSS Isolate (SRFA)	3.33	3.95	NA	17.11	NA	290-400	1.85
Leresche and Rosario-Ortiz 2019	SRFA pH 3 ozone 0.025 mmol O3 mmol C	IHSS Isolate (SRFA, O3)	4.06	3.56	NA	18.23	NA	290-400	2.43
Leresche and Rosario-Ortiz 2019	SRFA pH 3 ozone 0.055 mmol O3 mmol C	IHSS Isolate (SRFA, O3)	4.93	3.14	NA	19.24	NA	290-400	3.27
Leresche and Rosario-Ortiz 2019	SRFA pH 3 ozone 0.1 mmol O3 mmol C	IHSS Isolate (SRFA, O3)	6.16	2.74	NA	19.99	NA	290-400	4.20
Leresche and Rosario-Ortiz 2019	SRFA pH 3 ozone 0.15 mmol O3 mmol C	IHSS Isolate (SRFA, O3)	8.28	2.17	NA	20.13	NA	290-400	4.80
Leresche and Rosario-Ortiz 2019	SRFA pH 3 ozone 0.2 mmol O3 mmol C	IHSS Isolate (SRFA, O3)	8.46	2.19	NA	19.24	NA	290-400	4.43
Leresche and Rosario-Ortiz 2019	SRFA pH 3 ozone 0.25 mmol O3 mmol C	IHSS Isolate (SRFA, O3)	9.96	1.96	NA	22.86	NA	290-400	6.05
Leresche and Rosario-Ortiz 2019	SRFA pH 3 ozone 0.35 mmol O3 mmol C	IHSS Isolate (SRFA, O3)	10.71	1.83	NA	23.67	NA	290-400	5.96
Leresche and Rosario-Ortiz 2019	SRFA pH 3 ozone 0.5 mmol O3 mmol C	IHSS Isolate (SRFA, O3)	11.36	1.71	NA	23.67	NA	290-400	7.38
Leresche and Rosario-Ortiz 2019	SRFA pH 3 ozone 0.75 mmol O3 mmol C	IHSS Isolate (SRFA, O3)	15.33	1.45	NA	27.99	NA	290-400	7.76
Leresche and Rosario-Ortiz 2019	SRFA pH 3 ozone 1 mmol O3 mmol C	IHSS Isolate (SRFA, O3)	17.08	1.40	NA	25.19	NA	290-400	6.40
Leresche and Rosario-Ortiz 2019	SRFA pH 7 ozone 0 mmol O3 mmol C	IHSS Isolate (SRFA)	2.52	4.16	NA	15.58	NA	290-400	1.24
Leresche and Rosario-Ortiz 2019	SRFA pH 7 ozone 0.025 mmol O3 mmol C	IHSS Isolate (SRFA, O3)	2.81	3.87	NA	16.04	NA	290-400	1.27
Leresche and Rosario-Ortiz 2019	SRFA pH 7 ozone 0.055 mmol O3 mmol C	IHSS Isolate (SRFA, O3)	3.17	3.51	NA	16.50	NA	290-400	2.03
Leresche and Rosario-Ortiz 2019	SRFA pH 7 ozone 0.1 mmol O3 mmol C	IHSS Isolate (SRFA, O3)	3.84	3.08	NA	17.28	NA	290-400	2.55
Leresche and Rosario-Ortiz 2019	SRFA pH 7 ozone 0.15 mmol O3 mmol C	IHSS Isolate (SRFA, O3)	5.20	2.54	NA	18.81	NA	290-400	3.39
Leresche and Rosario-Ortiz 2019	SRFA pH 7 ozone 0.2 mmol O3 mmol C	IHSS Isolate (SRFA, O3)	6.58	2.15	NA	19.99	NA	290-400	4.40
Leresche and Rosario-Ortiz 2019	SRFA pH 7 ozone 0.25 mmol O3 mmol C	IHSS Isolate (SRFA, O3)	7.75	1.92	NA	20.91	NA	290-400	4.63
Leresche and Rosario-Ortiz 2019	SRFA pH 7 ozone 0.35 mmol O3 mmol C	IHSS Isolate (SRFA, O3)	10.98	1.65	NA	23.38	NA	290-400	6.25
Leresche and Rosario-Ortiz 2019	SRFA pH 7 ozone 0.5 mmol O3 mmol C	IHSS Isolate (SRFA, O3)	14.06	1.47	NA	25.11	NA	290-400	5.96
Leresche and Rosario-Ortiz 2019	SRFA pH 7 ozone 0.75 mmol O3 mmol C	IHSS Isolate (SRFA, O3)	19.40	1.30	NA	27.90	NA	290-400	9.81
Leresche and Rosario-Ortiz 2019	SRFA pH 7 ozone 1 mmol O3 mmol C	IHSS Isolate (SRFA, O3)	23.37	1.24	NA	29.65	NA	290-400	11.40
Leresche and Rosario-Ortiz 2019	San Juan River with tBuOH	Whole Water (River, tBuOH)	6.35	NA	NA	NA	NA	290-400	3.76
Leresche and Rosario-Ortiz 2019	San Juan River 0.05 mmol O3 mmolC w/ tBuOH	Whole Water (River, tBuOH)	8.27	NA	NA	NA	NA	290-400	5.41

Table S18. Summary of $\Phi_{app,\,^1O_2}$ and optical data (continued)

Reference	Sample ID	Sample Classification	*E2:E3*	$SUVA_{254}$ (L mg C^{-1}•m^{-1})	$S_{290–400}$ / $S_{300–400}$ / $S_{290–450}$ / $S_{295–400}$ (μm^{-1})	$S_{300–600}$ / $S_{300–500}$ / $S_{300–700}$ / $S_{275–500}$ (μm^{-1})	FI	Wavelength Range (nm)	$\Phi_{app,\,^1O_2}$ (×10^{-2} mol mol-photons^{-1})
Leresche and Rosario-Ortiz 2019	San Juan River 0.1 mmol O3 mmolC w/ tBuOH	Whole Water (River, tBuOH)	9.48	NA	NA	NA	NA	290-400	5.59
Leresche and Rosario-Ortiz 2019	San Juan River 0.2 mmol O3 mmolC w/ tBuOH	Whole Water (River, tBuOH)	10.70	NA	NA	NA	NA	290-400	4.74
Leresche and Rosario-Ortiz 2019	San Juan River 0.5 mmol O3 mmolC w/ tBuOH	Whole Water (River, tBuOH)	11.18	NA	NA	NA	NA	290-400	4.41
Leresche and Rosario-Ortiz 2019	San Juan River 0.75 mmol O3 mmolC w/ tBuOH	Whole Water (River, tBuOH)	12.17	NA	NA	NA	NA	290-400	6.15
Leresche and Rosario-Ortiz 2019	San Juan River 1.0 mmol O3 mmolC w/ tBuOH	Whole Water (River, tBuOH)	11.48	NA	NA	NA	NA	290-400	4.78
Leresche and Rosario-Ortiz 2019	San Juan River without tBuOH	Whole Water (River)	6.43	NA	NA	NA	NA	290-400	4.92
Leresche and Rosario-Ortiz 2019	San Juan River 0.05 mmol O3 mmolC without tBuOH	Whole Water (River, O3)	8.25	NA	NA	NA	NA	290-400	7.02
Leresche and Rosario-Ortiz 2019	San Juan River 0.1 mmol O3 mmolC without tBuOH	Whole Water (River, O3)	9.60	NA	NA	NA	NA	290-400	7.24
Leresche and Rosario-Ortiz 2019	San Juan River 0.2 mmol O3 mmolC without tBuOH	Whole Water (River, O3)	10.07	NA	NA	NA	NA	290-400	8.45
Leresche and Rosario-Ortiz 2019	San Juan River 0.5 mmol O3 mmolC without tBuOH	Whole Water (River, O3)	10.70	NA	NA	NA	NA	290-400	8.88
Leresche and Rosario-Ortiz 2019	San Juan River 0.75 mmol O3 mmolC without tBuOH	Whole Water (River, O3)	11.31	NA	NA	NA	NA	290-400	7.63
Leresche and Rosario-Ortiz 2019	San Juan River 1 mmol O3 mmolC without tBuOH	Whole Water (River, O3)	11.39	NA	NA	NA	NA	290-400	7.90
O'Connor and Arnold 2019 [32]	5_Santa Cruz River	Whole Water (WW-Impacted River)	5.50	2.30	NA	NA	2.11	275-600	3.29
O'Connor and Arnold 2019	10_Metro	Whole Water (WWTP Effluent)	4.90	2.48	NA	NA	2.14	275-600	1.95
O'Connor and Arnold 2019	12_Metro	Whole Water (WWTP Effluent)	5.30	3.00	NA	NA	2.09	275-600	2.00
O'Connor and Arnold 2019	17_Santa Ana River	Whole Water (WW-Impacted River)	6.70	2.74	NA	NA	1.82	275-600	3.53
O'Connor and Arnold 2019	18_Blue Lake	Whole Water (WWTP Effluent)	4.80	3.04	NA	NA	2.05	275-600	1.88
O'Connor and Arnold 2019	19_St. Croix Valley	Whole Water (WWTP Effluent)	4.50	2.48	NA	NA	2.25	275-600	1.69
O'Connor and Arnold 2019	20_Empire	Whole Water (WWTP Effluent)	4.30	2.74	NA	NA	2.29	275-600	1.40
O'Connor and Arnold 2019	26_Cannon Falls	Whole Water (WWTP Effluent)	4.30	1.78	NA	NA	2.51	275-600	2.83
O'Connor and Arnold 2019	31_Big Lake	Whole Water (WWTP Effluent)	5.30	2.17	NA	NA	2.20	275-600	1.54
O'Connor and Arnold 2019	37_Cannon Falls	Whole Water (WWTP Effluent)	4.30	1.95	NA	NA	2.21	275-600	0.86
O'Connor and Arnold 2019	40_Zumbrota	Whole Water (WWTP Effluent)	5.70	1.22	NA	NA	2.20	275-600	6.30
O'Connor and Arnold 2019	41_Kasson	Whole Water (WWTP Effluent)	4.50	1.74	NA	NA	2.22	275-600	1.47
O'Connor and Arnold 2019	42_Owatonna	Whole Water (WWTP Effluent)	5.60	2.17	NA	NA	2.12	275-600	2.49
O'Connor and Arnold 2019	47_Wanamingo	Whole Water (WWTP Effluent)	4.80	2.78	NA	NA	1.99	275-600	1.47
O'Connor and Arnold 2019	50_St. Croix	Whole Water (WWTP Effluent)	4.10	2.69	NA	NA	2.11	275-600	0.80
O'Connor and Arnold 2019	51_Becker (combined stream)	Whole Water (WWTP Effluent)	4.30	2.56	NA	NA	2.12	275-600	0.90
O'Connor and Arnold 2019	54_Northfield	Whole Water (WWTP Effluent)	3.80	1.78	NA	NA	2.11	275-600	0.47
O'Connor and Arnold 2019	60_Zumbrota	Whole Water (WWTP Effluent)	4.50	1.39	NA	NA	2.11	275-600	3.04
O'Connor and Arnold 2019	73_Becker (residential waste stream)	Whole Water (WWTP Effluent)	5.00	2.30	NA	NA	1.94	275-600	1.27
O'Connor and Arnold 2019	74_St. Cloud	Whole Water (WWTP Effluent)	5.10	2.26	NA	NA	2.08	275-600	0.73
O'Connor and Arnold 2019	82_Faribault	Whole Water (WWTP Effluent)	5.50	1.74	NA	NA	2.06	275-600	2.44
O'Connor and Arnold 2019	83_Seneca	Whole Water (WWTP Effluent)	4.40	2.43	NA	NA	2.21	275-600	1.00
O'Connor and Arnold 2019	91_Rochester (non-conventional aeration)	Whole Water (WWTP Effluent)	5.60	2.30	NA	NA	2.13	275-600	1.37
O'Connor and Arnold 2019	92_Kasson	Whole Water (WWTP Effluent)	4.80	1.82	NA	NA	2.19	275-600	1.79
Wan and Chen 2019 [171]	EfOM Wastewater effluent	C18 Extract (WWTP Effluent)	3.90	NA	NA	NA	NA	300-600	3.29
Wan and Chen 2019	Henan Changsheng Industrial Fulvic acid (FA)	XAD Fraction (Soil)	2.30	NA	NA	NA	NA	300-600	2.23
Wan and Chen 2019	Aldrich humic acid (HA)	XAD Fraction (Soil)	2.70	NA	NA	NA	NA	300-600	2.15
Wang and Chen 2019 [111]	CS-DOM-1#	RO/ED Isolate (Seawater)	6.50	2.07	NA	NA	NA	290-400	3.36
Wang and Chen 2019	CS-DOM-2#	RO/ED Isolate (Seawater)	5.05	2.22	NA	NA	NA	290-400	2.75
Wang and Chen 2019	SRFA	IHSS Isolate (SRFA)	4.39	9.24	NA	NA	NA	290-400	2.20
Wang and Chen 2019	SRNOM	IHSS Isolate (SRNOM)	4.45	8.93	NA	NA	NA	290-400	2.79
Wenk 2019 [172]	Wetland influent PPL Isolate	PPL Extract (WW-Impacted	4.95	3.04	NA	13.87	NA	300-700	2.66
Wenk 2019	Wetland influent	Whole Water (WW-Impacted	4.61	2.57	NA	14.92	NA	300-700	2.27
Wenk 2019	Bulrush cell effluent PPL Isolate	PPL Extract (WW-Impacted	4.03	3.14	NA	13.50	NA	300-700	1.86
Wenk 2019	Bulrush cell effluent	Whole Water (WW-Impacted	3.77	2.73	NA	14.40	NA	300-700	1.95
Wenk 2019	Cattail cell effluent PPL Isolate	PPL Extract (WW-Impacted	4.67	3.48	NA	13.47	NA	300-700	1.80
Wenk 2019	Cattail cell effluent	Whole Water (WW-Impacted	4.26	2.71	NA	14.27	NA	300-700	2.11
Wenk 2019	Open water cell effluent PPL Isolate	PPL Extract (WW-Impacted	5.65	3.04	NA	14.33	NA	300-700	2.52
Wenk 2019	Open water cell effluent	Whole Water (WW-Impacted	5.35	2.33	NA	15.23	NA	300-700	3.20

Table S18. Summary of $\Phi_{app,\ ^1O_2}$ and optical data (continued)

Reference	Sample ID	Sample Classification	*E2:E3*	$SUVA_{254}$ (L mg C^{-1}•m^{-1})	$S_{290–400}$ / $S_{300–400}$ / $S_{290–450}$ / $S_{295–400}$ (μm^{-1})	$S_{300–600}$ / $S_{300–500}$ / $S_{300–700}$ / $S_{275–500}$ (μm^{-1})	FI	Wavelength Range (nm)	$\Phi_{app,\ ^1O_2}$ ($\times 10^{-2}$ mol mol-photons^{-1})
Wenk 2019	Suwannee River fulvic acid (SRFA)	IHSS Isolate (SRFA)	4.96	4.09	NA	16.07	NA	300-700	1.44
Wenk 2019	Pony Lake fulvic acid (PLFA)	IHSS Isolate (PLFA)	5.64	2.37	NA	15.23	NA	300-700	1.92
Zhou and Song 2019 [173]	WWOM 1 Wastewater-impacted lake Dianshan Lake, China (Shanghai)	PPL Extract (WW-Impacted Lake)	7.50	2.00	NA	NA	1.60	290-400	7.42
Zhou and Song 2019	WWOM 2 Wastewater-impacted river Huangpu River, China	PPL Extract (WW-Impacted River)	7.00	1.70	NA	NA	1.60	290-400	7.95
Zhou and Song 2019	WWOM 3 Wastewater-impacted river Suzhou River, China (Shangahi)	PPL Extract (WW-Impacted River)	6.60	1.90	NA	NA	1.70	290-400	7.46
Zhou and Song 2019	WWOM 4 Wastewater-impacted river Suzhou River, China (Shangahi)	PPL Extract (WW-Impacted River)	6.60	1.80	NA	NA	1.70	290-400	6.47
Zhou and Song 2019	WWOM 5 Wastewater-impacted lake Jiangwan Lake, China (Shangahi)	PPL Extract (WW-Impacted Lake)	6.20	2.00	NA	NA	1.60	290-400	6.14
Zhou and Song 2019	WWOM 6 Wastewater-impacted lake Fudan Lake, China (Shangahi)	PPL Extract (WW-Impacted Lake)	6.70	1.60	NA	NA	1.60	290-400	6.11
Zhou and Song 2019	WWOM 7 Wastewater-impacted lake Fudan Lake, China (Shangahi)	PPL Extract (WW-Impacted Lake)	6.50	1.50	NA	NA	1.70	290-400	7.18
Zhou and Song 2019	WWOM 8 Wastewater-impacted river Wusongkou Port, China	PPL Extract (WW-Impacted River)	7.30	1.50	NA	NA	1.70	290-400	7.30
Zhou and Song 2019	EfOM 1 Wastewater Effluent Jiangsu, China	PPL Extract (WWTP Effluent)	5.20	1.80	NA	NA	2.00	290-400	4.55
Zhou and Song 2019	EfOM 2 Wastewater Effluent Shanghai, China	PPL Extract (WWTP Effluent)	5.10	1.90	NA	NA	1.80	290-400	5.45
Zhou and Song 2019	Suwannee River natural organic matter (SRNOM)	IHSS Isolate (SRNOM)	4.40	4.20	NA	NA	1.30	290-400	2.46
Zhou and Song 2019	Suwannee River humic acid (SRHA)	IHSS Isolate (SRHA)	3.50	5.80	NA	NA	1.10	290-400	2.02
Zhou and Song 2019	Suwannee River fulvic acid (SRFA)	IHSS Isolate (SRFA)	4.60	4.60	NA	NA	1.30	290-400	2.66
Zhou and Song 2019	Pony Lake fulvic acid (PLFA)	IHSS Isolate (PLFA)	5.10	2.70	NA	NA	1.50	290-400	5.29
Zhou and Song 2019	Nordic Lake natural organic matter (NLNOM)	IHSS Isolate (NLNOM)	4.40	4.40	NA	NA	1.40	290-400	3.70
Chen and Arnold 2020 [174]	Lake Bemidji	Whole Water (Lake)	12.00	3.66	NA	NA	NA	275-600	3.14
Chen and Arnold 2020	Blandin Reservoir	Whole Water (Lake)	6.29	3.18	NA	NA	NA	275-600	1.98
Chen and Arnold 2020	Burntside Lake	Whole Water (Lake)	8.31	1.99	NA	NA	NA	275-600	1.70
Chen and Arnold 2020	Gull Lake	Whole Water (Lake)	NA	NA	NA	NA	NA	275-600	2.73
Chen and Arnold 2020	Island Lake	Whole Water (Lake)	9.11	1.97	NA	NA	NA	275-600	2.37
Chen and Arnold 2020	Lake Itasca	Whole Water (Lake)	10.56	1.34	NA	NA	NA	275-600	2.91
Chen and Arnold 2020	Mille Lacs Lake	Whole Water (Lake)	5.32	1.61	NA	NA	NA	275-600	1.91
Chen and Arnold 2020	Rainy Lake	Whole Water (Lake)	5.82	3.18	NA	NA	NA	275-600	1.59
Chen and Arnold 2020	Shagawa Lake	Whole Water (Lake)	6.74	2.87	NA	NA	NA	275-600	1.87
Chen and Arnold 2020	South Sturgeon Lake	Whole Water (Lake)	4.53	4.85	NA	NA	NA	275-600	0.95
Chen and Arnold 2020	Sturgeon Lake	Whole Water (Lake)	7.30	2.26	NA	NA	NA	275-600	1.99
Chen and Arnold 2020	Lake Vermilion (Big Bay)	Whole Water (Lake)	6.77	3.05	NA	NA	NA	275-600	2.06
Chen and Arnold 2020	Lake Vermilion (Pike Bay)	Whole Water (Lake)	4.46	5.05	NA	NA	NA	275-600	1.20
Chen and Arnold 2020	White Iron Lake - South	Whole Water (Lake)	5.01	4.63	NA	NA	NA	275-600	1.18
Chen and Arnold 2020	Lake Winnibigosh	Whole Water (Lake)	10.62	1.79	NA	NA	NA	275-600	2.28
Chen and Arnold 2020	Upper Red Lake #1	Whole Water (Lake)	NA	NA	NA	NA	NA	275-600	3.18
Chen and Arnold 2020	Upper Red Lake #2	Whole Water (Lake)	5.04	4.20	NA	NA	NA	275-600	1.25
Chen and Arnold 2020	Lake of the Woods (Muskeg Bay)	Whole Water (Lake)	5.65	3.26	NA	NA	NA	275-600	1.94
Chen and Arnold 2020	Lake of the Woods (Fourmile Bay)	Whole Water (Lake)	5.40	3.85	NA	NA	NA	275-600	1.15
Chen and Arnold 2020	Crystal Lake	Whole Water (Lake)	11.78	1.61	NA	NA	NA	275-600	2.42
Chen and Arnold 2020	Detroit Lake	Whole Water (Lake)	10.24	2.10	NA	NA	NA	275-600	2.99
Chen and Arnold 2020	Mississippi River	Whole Water (River)	6.21	3.32	NA	NA	NA	275-600	2.35
Chen and Arnold 2020	Vadnais Lake	Whole Water (Lake)	7.69	2.36	NA	NA	NA	275-600	2.78
Chen and Arnold 2020	Lake Saint Croix	Whole Water (Lake)	5.18	4.26	NA	NA	NA	275-600	1.87
Palma and Richard 2020 [175]	Allier March	Whole Water (River)	5.22	2.60	NA	NA	1.35	300-450	1.00
Palma and Richard 2020	Allier July	Whole Water (River)	5.24	2.82	NA	NA	1.46	300-450	1.30
Palma and Richard 2020	Allier October	Whole Water (River)	5.38	2.36	NA	NA	1.38	300-450	1.20
Palma and Richard 2020	B4 March	Whole Water (River)	6.01	0.78	NA	NA	1.54	300-450	1.50
Palma and Richard 2020	B4 July	Whole Water (River)	5.57	0.30	NA	NA	1.59	300-450	2.10
Palma and Richard 2020	B4 October	Whole Water (River)	5.12	1.15	NA	NA	1.60	300-450	1.40
Palma and Richard 2020	B7 March	Whole Water (River)	5.43	1.10	NA	NA	1.58	300-450	1.10
Palma and Richard 2020	B7 July	Whole Water (River)	4.60	0.49	NA	NA	1.55	300-450	1.90
Palma and Richard 2020	B7 October	Whole Water (River)	5.52	1.26	NA	NA	1.60	300-450	1.70
Palma and Richard 2020	Vendage March	Whole Water (River)	6.38	1.78	NA	NA	1.45	300-450	0.78

**Table S18.** Summary of $\Phi_{app,\ ^1O_2}$ and optical data (continued)									
Reference	Sample ID	Sample Classification	*E2:E3*	SUVA$_{254}$ (L mg C^{-1}•m^{-1})	$S_{290–400}$ / $S_{300–400}$ / $S_{290–450}$ / $S_{295–400}$ (μm^{-1})	$S_{300–600}$ / $S_{300–500}$ / $S_{300–700}$ / $S_{275–500}$ (μm^{-1})	FI	Wavelength Range (nm)	$\Phi_{app,\ ^1O_2}$ (×10^{-2} mol mol-photons^{-1})
Palma and Richard 2020	Vendage July	Whole Water (River)	5.75	0.44	NA	NA	1.49	300-450	1.90
Palma and Richard 2020	Vendage October	Whole Water (River)	4.56	1.55	NA	NA	1.54	300-450	1.20
Palma and Richard 2020	PZ1 March	Whole Water (Groundwater)	9.30	1.08	NA	NA	1.54	300-450	3.00
Palma and Richard 2020	PZ1 July	Whole Water (Groundwater)	6.50	0.55	NA	NA	1.60	300-450	3.10
Palma and Richard 2020	PZ1 October	Whole Water (Groundwater)	7.06	1.25	NA	NA	1.57	300-450	4.00
Palma and Richard 2020	PZ5 March	Whole Water (Groundwater)	10.80	0.30	NA	NA	1.71	300-450	5.00
Palma and Richard 2020	PZ5 July	Whole Water (Groundwater)	8.12	0.28	NA	NA	1.62	300-450	2.90
Palma and Richard 2020	PZ5 October	Whole Water (Groundwater)	8.19	0.53	NA	NA	1.66	300-450	6.30
Partanen and McNeill 2020 [30]	Suwannee River natural organic matter (SRNOM)	IHSS Isolate (SRNOM)	NA	NA	NA	NA	NA	365	1.80
Partanen and McNeill 2020	Suwannee River fulvic acid (SRFA)	IHSS Isolate (SRFA)	NA	NA	NA	NA	NA	365	1.40
Partanen and McNeill 2020	Suwannee River humic acid (SRHA)	IHSS Isolate (SRHA)	NA	NA	NA	NA	NA	365	0.60
Partanen and McNeill 2020	Nordic Lake natural organic matter (NLNOM)	IHSS Isolate (NLNOM)	NA	NA	NA	NA	NA	365	1.30
Partanen and McNeill 2020	Mississippi River natural organic matter (MRNOM)	IHSS Isolate (UMRNOM)	NA	NA	NA	NA	NA	365	2.00
Partanen and McNeill 2020	Pony Lake fulvic acid (PLFA)	IHSS Isolate (PLFA)	NA	NA	NA	NA	NA	365	2.40
Partanen and McNeill 2020	Pacific Ocean HPOA	XAD Fraction (Seawater)	NA	NA	NA	NA	NA	365	2.60
Partanen and McNeill 2020	Lake Fryxell FA (Antarctic)	XAD Fraction (Lake)	NA	NA	NA	NA	NA	365	2.80
Partanen and McNeill 2020	Everglades HPOA	XAD Fraction (Estuary)	NA	NA	NA	NA	NA	365	1.60
Partanen and McNeill 2020	Williams Lake HPOA (Minnesota)	XAD Fraction (Lake)	NA	NA	NA	NA	NA	365	2.20
Partanen and McNeill 2020	Everglades TPIA	XAD Fraction (Estuary)	NA	NA	NA	NA	NA	365	2.80
Partanen and McNeill 2020	Williams Lake TPIA	XAD Fraction (Lake)	NA	NA	NA	NA	NA	365	2.10
Partanen and McNeill 2020	Everglades HPON	XAD Fraction (Estuary)	NA	NA	NA	NA	NA	365	3.80
Partanen and McNeill 2020	Williams Lake HPON	XAD Fraction (Lake)	NA	NA	NA	NA	NA	365	3.00
Partanen and McNeill 2020	Great Dismal Swamp (Florida)	Whole Water (Wetland)	NA	NA	NA	NA	NA	365	1.00
Partanen and McNeill 2020	Lake Bradford (Florida)	Whole Water (Lake)	NA	NA	NA	NA	NA	365	1.40
Partanen and McNeill 2020	Suwannee River	Whole Water (River)	NA	NA	NA	NA	NA	365	1.60
Partanen and McNeill 2020	Étang de la Gruère (Switzerland)	Whole Water (Lake)	NA	NA	NA	NA	NA	365	0.80
Partanen and McNeill 2020	SRNOM 300 nm	IHSS Isolate (SRNOM)	NA	NA	NA	NA	NA	300	2.50
Partanen and McNeill 2020	SRNOM 330 nm	IHSS Isolate (SRNOM)	NA	NA	NA	NA	NA	330	2.10
Partanen and McNeill 2020	SRNOM 400 nm	IHSS Isolate (SRNOM)	NA	NA	NA	NA	NA	400	1.30
Partanen and McNeill 2020	SRNOM 430 nm	IHSS Isolate (SRNOM)	NA	NA	NA	NA	NA	430	0.90
Partanen and McNeill 2020	SRNOM 470 nm	IHSS Isolate (SRNOM)	NA	NA	NA	NA	NA	470	0.50
Partanen and McNeill 2020	SRNOM 500 nm	IHSS Isolate (SRNOM)	NA	NA	NA	NA	NA	500	0.30
Partanen and McNeill 2020	SRNOM 540 nm	IHSS Isolate (SRNOM)	NA	NA	NA	NA	NA	540	0.20
Partanen and McNeill 2020	SRNOM 560 nm	IHSS Isolate (SRNOM)	NA	NA	NA	NA	NA	560	0.10
Partanen and McNeill 2020	PLFA 300 nm	IHSS Isolate (PLFA)	NA	NA	NA	NA	NA	300	4.00
Partanen and McNeill 2020	PLFA 400 nm	IHSS Isolate (PLFA)	NA	NA	NA	NA	NA	400	1.60
Partanen and McNeill 2020	PLFA 430 nm	IHSS Isolate (PLFA)	NA	NA	NA	NA	NA	430	1.10
Partanen and McNeill 2020	PLFA 470 nm	IHSS Isolate (PLFA)	NA	NA	NA	NA	NA	470	0.40
Partanen and McNeill 2020	Suwannee River 320 nm	Whole Water (River)	NA	NA	NA	NA	NA	320	1.80
Partanen and McNeill 2020	Suwannee River 400 nm	Whole Water (River)	NA	NA	NA	NA	NA	400	1.50
Partanen and McNeill 2020	Suwannee River 430 nm	Whole Water (River)	NA	NA	NA	NA	NA	430	1.10
Partanen and McNeill 2020	Étang de la Gruère 300 nm	Whole Water (Lake)	NA	NA	NA	NA	NA	300	1.10
Partanen and McNeill 2020	Étang de la Gruère 320 nm	Whole Water (Lake)	NA	NA	NA	NA	NA	320	1.20
Partanen and McNeill 2020	Étang de la Gruère 340 nm	Whole Water (Lake)	NA	NA	NA	NA	NA	340	0.80
Partanen and McNeill 2020	Étang de la Gruère 380 nm	Whole Water (Lake)	NA	NA	NA	NA	NA	380	0.60
Wan and Chen 2020 [176]	EfOM1	C18 Extract (WWTP Effluent)	4.26	1.36	NA	NA	2.44	300-600	4.26
Wan and Chen 2020	EfOM1 + UV 15 min	C18 Extract (WWTP Effluent, UV)	4.27	1.14	NA	NA	2.36	300-600	4.33
Wan and Chen 2020	EfOM1 + UV 30 min	C18 Extract (WWTP Effluent, UV)	4.31	1.04	NA	NA	2.35	300-600	4.39
Wan and Chen 2020	EfOM1 + UV 60 min	C18 Extract (WWTP Effluent, UV)	4.46	0.94	NA	NA	2.32	300-600	4.40
Wan and Chen 2020	EfOM1+UV 120 min	C18 Extract (WWTP Effluent, UV)	4.60	0.81	NA	NA	2.29	300-600	4.41
Wan and Chen 2020	EfOM1+UV 240 min	C18 Extract (WWTP Effluent, UV)	5.06	0.65	NA	NA	2.03	300-600	4.40

**Table S18.** Summary of $\Phi_{app, {}^1O_2}$ and optical data (continued)									
Reference	Sample ID	Sample Classification	*E2:E3*	SUVA$_{254}$ (L mg C^{-1}•m^{-1})	$S_{290–400}$ / $S_{300–400}$ / $S_{290–450}$ / $S_{295–400}$ (μm^{-1})	$S_{300–600}$ / $S_{300–500}$ / $S_{300–700}$ / $S_{275–500}$ (μm^{-1})	FI	Wavelength Range (nm)	$\Phi_{app, {}^1O_2}$ (×10^{-2} mol mol-photons^{-1})
Wan and Chen 2020	EfOM2	C18 Extract (WWTP Effluent)	3.80	1.19	NA	NA	2.41	300-600	4.12
Wan and Chen 2020	EfOM2 + UV 15 min	C18 Extract (WWTP Effluent, UV)	3.83	1.08	NA	NA	2.40	300-600	4.27
Wan and Chen 2020	EfOM2 + UV 30 min	C18 Extract (WWTP Effluent, UV)	3.84	0.97	NA	NA	2.33	300-600	4.33
Wan and Chen 2020	EfOM2 + UV 60 min	C18 Extract (WWTP Effluent, UV)	3.91	0.92	NA	NA	2.25	300-600	4.36
Wan and Chen 2020	EfOM2 + UV 120 min	C18 Extract (WWTP Effluent, UV)	4.15	0.67	NA	NA	2.15	300-600	4.45
Wan and Chen 2020	EfOM2 + UV 240 min	C18 Extract (WWTP Effluent, UV)	4.56	0.59	NA	NA	2.12	300-600	4.67
Wan and Chen 2020	EfOM3	C18 Extract (WWTP Effluent)	4.32	1.08	NA	NA	2.38	300-600	5.08
Wan and Chen 2020	EfOM3 + UV 15 min	C18 Extract (WWTP Effluent, UV)	4.33	0.91	NA	NA	2.32	300-600	5.18
Wan and Chen 2020	EfOM3 + UV 30 min	C18 Extract (WWTP Effluent, UV)	4.34	0.84	NA	NA	2.22	300-600	5.20
Wan and Chen 2020	EfOM3 + UV 60 min	C18 Extract (WWTP Effluent, UV)	4.41	0.72	NA	NA	2.14	300-600	5.20
Wan and Chen 2020	EfOM3 + UV 120 min	C18 Extract (WWTP Effluent, UV)	4.62	0.63	NA	NA	2.11	300-600	5.21
Wan and Chen 2020	EfOM3 + UV 240 min	C18 Extract (WWTP Effluent, UV)	4.88	0.63	NA	NA	2.09	300-600	5.22
Wan and Chen 2020	Henan Changsheng Industrial Fulvic acid (FA)	XAD Fraction (Soil)	2.39	11.86	NA	NA	0.65	300-600	3.10
Wan and Chen 2020	FA + UV 15 min	XAD Fraction (Soil, UV)	2.45	11.34	NA	NA	0.66	300-600	2.95
Wan and Chen 2020	FA + UV 30 min	XAD Fraction (Soil, UV)	2.47	10.78	NA	NA	0.67	300-600	2.73
Wan and Chen 2020	FA + UV 60 min	XAD Fraction (Soil, UV)	2.50	10.55	NA	NA	0.68	300-600	2.43
Wan and Chen 2020	FA + UV 120 min	XAD Fraction (Soil, UV)	2.54	10.41	NA	NA	0.69	300-600	1.96
Wan and Chen 2020	FA + UV 240 min	XAD Fraction (Soil, UV)	2.58	10.37	NA	NA	0.70	300-600	1.37
Wan and Chen 2020	Aldrich humic acid (HA)	XAD Fraction (Soil)	2.37	5.53	NA	NA	1.24	300-600	2.47
Wan and Chen 2020	HA + UV 15 min	XAD Fraction (Soil, UV)	2.40	5.43	NA	NA	1.24	300-600	2.34
Wan and Chen 2020	HA + UV 30 min	XAD Fraction (Soil, UV)	2.46	5.36	NA	NA	1.24	300-600	2.30
Wan and Chen 2020	HA + UV 60 min	XAD Fraction (Soil, UV)	2.50	5.19	NA	NA	1.25	300-600	2.10
Wan and Chen 2020	HA + UV 120 min	XAD Fraction (Soil, UV)	2.52	5.01	NA	NA	1.26	300-600	1.82
Wang and Song 2020 [177]	Dissolved black carbon_sorghum straw	DBC (pyDOM)	4.10	NA	NA	NA	NA	290-400	3.71
Wang and Song 2020	Dissolved black carbon_peanut straw	DBC (pyDOM)	6.80	NA	NA	NA	NA	290-400	6.68
Wang and Song 2020	Dissolved black carbon_maize straw	DBC (pyDOM)	5.50	NA	NA	NA	NA	290-400	3.75
Wang and Song 2020	Dissolved black carbon_soybean straw	DBC (pyDOM)	6.20	NA	NA	NA	NA	290-400	4.82
Wang and Song 2020	Dissolved black carbon_bamboo	DBC (pyDOM)	4.30	NA	NA	NA	NA	290-400	3.21
Wang and Song 2020	Dissolved black carbon_rice straw	DBC (pyDOM)	4.30	NA	NA	NA	NA	290-400	3.08
Wang and Song 2020	Nordic Lake natural organic matter (NLNOM)	IHSS Isolate (NLNOM)	4.50	NA	NA	NA	NA	290-400	3.71
Wang and Song 2020	Pony Lake fulvic acid (PLFA)	IHSS Isolate (PLFA)	5.10	NA	NA	NA	NA	290-400	5.29
Wang and Song 2020	Suwannee River fulvic acid (SRFA)	IHSS Isolate (SRFA)	3.90	NA	NA	NA	NA	290-400	2.66
Wang and Song 2020	Suwannee River humic acid (SRHA)	IHSS Isolate (SRHA)	3.20	NA	NA	NA	NA	290-400	2.02
Wang and Song 2020	Suwannee River natural organic matter (SRNOM)	IHSS Isolate (SRNOM)	4.30	NA	NA	NA	NA	290-400	2.46
Wasswa and Zeng 2020 [29]	Arbutus Lake	Whole Water (Lake)	7.04	2.49	18.38	17.98	1.44	290-550	2.65
Wasswa and Zeng 2020	Big Moose Lake	Whole Water (Lake)	6.50	2.96	18.34	19.03	1.54	290-550	2.62
Wasswa and Zeng 2020	Black Pond	Whole Water (Lake)	6.36	2.84	17.03	14.98	1.46	290-550	1.89
Wasswa and Zeng 2020	Dart Lake	Whole Water (Lake)	6.80	2.89	18.46	16.72	1.50	290-550	2.58
Wasswa and Zeng 2020	G Lake	Whole Water (Lake)	7.44	2.09	18.74	17.11	1.59	290-550	2.34
Wasswa and Zeng 2020	Honnedaga Lake	Whole Water (Lake)	5.53	2.93	15.90	11.92	1.69	290-550	1.90
Wasswa and Zeng 2020	Limekiln Lake	Whole Water (Lake)	8.21	2.05	17.40	11.49	1.57	290-550	2.12
Wasswa and Zeng 2020	Little Hope Pond	Whole Water (Lake)	5.03	3.81	15.66	13.83	1.40	290-550	1.53
Wasswa and Zeng 2020	Moss Lake	Whole Water (Lake)	6.45	2.68	17.39	14.04	1.50	290-550	1.88
Wasswa and Zeng 2020	North Lake	Whole Water (Lake)	5.43	3.62	16.56	14.88	1.51	290-550	1.96
Wasswa and Zeng 2020	Lake Rondaxe	Whole Water (Lake)	8.30	1.78	15.90	11.68	1.58	290-550	2.08
Wasswa and Zeng 2020	Sagamore Lake	Whole Water (Lake)	5.19	3.95	16.26	14.79	1.46	290-550	1.63
Wasswa and Zeng 2020	South Lake	Whole Water (Lake)	7.38	2.40	18.46	15.73	1.52	290-550	2.30
Wasswa and Zeng 2020	Squaw Lake	Whole Water (Lake)	7.44	2.13	19.67	25.15	1.47	290-550	2.88
Wasswa and Zeng 2020	Willis Lake	Whole Water (Lake)	6.00	2.83	17.10	15.98	1.43	290-550	2.17
Wasswa and Zeng 2020	Wolf Lake	Whole Water (Lake)	9.32	1.87	20.37	19.87	1.52	290-550	3.31
Wasswa and Zeng 2020	Arbutus Lake - pH 6.5 Standardized	Whole Water (Lake)	8.20	2.95	19.31	18.90	1.54	290-550	3.17

**Table S18.** Summary of $\Phi_{app, ^1O_2}$ and optical data (continued)									
Reference	Sample ID	Sample Classification	*E2:E3*	SUVA$_{254}$ (L mg C^{-1}•m^{-1})	$S_{290–400}$ / $S_{300–400}$ / $S_{290–450}$ / $S_{295–400}$ (μm^{-1})	$S_{300–600}$ / $S_{300–500}$ / $S_{300–700}$ / $S_{275–500}$ (μm^{-1})	FI	Wavelength Range (nm)	$\Phi_{app, ^1O_2}$ (×10^{-2} mol mol-photons^{-1})
Wasswa and Zeng 2020	Big Moose Lake - pH 6.5 Standardized	Whole Water (Lake)	6.59	2.87	18.83	24.20	1.48	290-550	3.09
Wasswa and Zeng 2020	Black Pond - pH 6.5 Standardized	Whole Water (Lake)	6.36	2.84	17.03	14.98	1.46	290-550	1.89
Wasswa and Zeng 2020	Dart Lake - pH 6.5 Standardized	Whole Water (Lake)	6.63	2.85	18.43	24.27	1.47	290-550	2.84
Wasswa and Zeng 2020	G Lake - pH 6.5 Standardized	Whole Water (Lake)	7.44	2.09	18.74	17.11	1.59	290-550	2.34
Wasswa and Zeng 2020	Honnedaga Lake - pH 6.5 Standardized	Whole Water (Lake)	5.53	2.93	15.90	11.92	1.58	290-550	1.90
Wasswa and Zeng 2020	Limekiln Lake - pH 6.5 Standardized	Whole Water (Lake)	7.55	2.04	18.92	14.02	1.47	290-550	2.32
Wasswa and Zeng 2020	Little Hope Pond - pH 6.5 Standardized	Whole Water (Lake)	5.22	3.94	16.24	15.93	1.38	290-550	1.64
Wasswa and Zeng 2020	Moss Lake - pH 6.5 Standardized	Whole Water (Lake)	6.56	2.80	17.55	15.69	1.54	290-550	2.02
Wasswa and Zeng 2020	North Lake - pH 6.5 Standardized	Whole Water (Lake)	5.17	3.75	15.76	15.16	1.52	290-550	1.75
Wasswa and Zeng 2020	Lake Rondaxe - pH 6.5 Standardized	Whole Water (Lake)	6.54	2.01	17.40	11.52	1.63	290-550	2.27
Wasswa and Zeng 2020	Sagamore Lake - pH 6.5 Standardized	Whole Water (Lake)	5.17	3.91	16.19	14.72	1.44	290-550	1.66
Wasswa and Zeng 2020	South Lake - pH 6.5 Standardized	Whole Water (Lake)	7.38	2.40	18.46	15.73	1.52	290-550	2.30
Wasswa and Zeng 2020	Squaw Lake - pH 6.5 Standardized	Whole Water (Lake)	8.83	2.07	21.88	31.57	1.47	290-550	4.50
Wasswa and Zeng 2020	Willis Lake - pH 6.5 Standardized	Whole Water (Lake)	6.15	2.71	17.53	17.84	1.36	290-550	2.49
Wasswa and Zeng 2020	Wolf Lake - pH 6.5 Standardized	Whole Water (Lake)	9.32	1.87	20.37	19.87	1.54	290-550	3.31
Wasswa and Zeng 2020	Arbutus Lake - pH 4.5	Whole Water (Lake)	7.66	2.51	18.59	18.90	1.53	290-550	3.12
Wasswa and Zeng 2020	Big Moose Lake - pH 4.5	Whole Water (Lake)	6.54	3.68	16.56	11.59	1.53	290-550	1.81
Wasswa and Zeng 2020	Black Pond - pH 4.5	Whole Water (Lake)	6.83	2.81	17.59	15.76	1.46	290-550	2.18
Wasswa and Zeng 2020	Dart Lake - pH 4.5	Whole Water (Lake)	7.32	2.84	19.76	24.16	1.56	290-550	3.39
Wasswa and Zeng 2020	G Lake - pH 4.5	Whole Water (Lake)	7.49	1.98	17.55	12.71	1.64	290-550	2.53
Wasswa and Zeng 2020	Honnedaga Lake - pH 4.5	Whole Water (Lake)	6.19	2.92	17.29	17.18	1.70	290-550	2.35
Wasswa and Zeng 2020	Limekiln Lake - pH 4.5	Whole Water (Lake)	7.83	2.15	17.86	14.80	1.59	290-550	2.10
Wasswa and Zeng 2020	Little Hope Pond - pH 4.5	Whole Water (Lake)	5.28	3.66	16.47	18.14	1.45	290-550	1.78
Wasswa and Zeng 2020	Moss Lake - pH 4.5	Whole Water (Lake)	6.00	3.26	15.60	11.57	1.51	290-550	1.44
Wasswa and Zeng 2020	North Lake - pH 4.5	Whole Water (Lake)	5.84	4.69	16.56	12.58	1.48	290-550	1.67
Wasswa and Zeng 2020	Lake Rondaxe - pH 4.5	Whole Water (Lake)	8.57	1.46	20.67	25.25	1.69	290-550	4.55
Wasswa and Zeng 2020	Sagamore Lake - pH 4.5	Whole Water (Lake)	5.66	3.80	16.83	15.76	1.51	290-550	2.01
Wasswa and Zeng 2020	South Lake - pH 4.5	Whole Water (Lake)	8.56	2.21	21.78	30.06	1.54	290-550	4.80
Wasswa and Zeng 2020	Squaw Lake - pH 4.5	Whole Water (Lake)	8.79	2.16	21.08	28.20	1.54	290-550	3.65
Wasswa and Zeng 2020	Willis Lake - pH 4.5	Whole Water (Lake)	6.50	2.83	18.18	23.19	1.51	290-550	2.34
Wasswa and Zeng 2020	Wolf Lake - pH 4.5	Whole Water (Lake)	10.77	1.80	22.67	27.91	1.54	290-550	5.15
Wasswa and Zeng 2020	Arbutus Lake - pH 8.5	Whole Water (Lake)	6.76	2.70	16.50	13.10	1.51	290-550	2.15
Wasswa and Zeng 2020	Big Moose Lake - pH 8.5	Whole Water (Lake)	6.55	3.09	17.42	15.97	1.48	290-550	2.28
Wasswa and Zeng 2020	Black Pond - pH 8.5	Whole Water (Lake)	6.96	2.93	17.61	16.80	1.47	290-550	2.09
Wasswa and Zeng 2020	Dart Lake - pH 8.5	Whole Water (Lake)	6.87	2.99	17.80	16.01	1.47	290-550	2.15
Wasswa and Zeng 2020	G Lake - pH 8.5	Whole Water (Lake)	7.90	2.05	17.99	16.17	1.55	290-550	2.42
Wasswa and Zeng 2020	Honnedaga Lake - pH 8.5	Whole Water (Lake)	6.58	2.90	17.57	15.83	1.57	290-550	2.38
Wasswa and Zeng 2020	Limekiln Lake - pH 8.5	Whole Water (Lake)	8.94	1.94	19.66	14.86	1.52	290-550	2.46
Wasswa and Zeng 2020	Little Hope Pond - pH 8.5	Whole Water (Lake)	5.11	3.79	15.59	13.79	1.43	290-550	1.39
Wasswa and Zeng 2020	Moss Lake - pH 8.5	Whole Water (Lake)	6.90	2.73	17.94	16.03	1.47	290-550	2.06
Wasswa and Zeng 2020	North Lake - pH 8.5	Whole Water (Lake)	5.64	3.57	16.91	17.25	1.47	290-550	1.90
Wasswa and Zeng 2020	Lake Rondaxe - pH 8.5	Whole Water (Lake)	10.26	1.80	22.24	30.25	1.68	290-550	3.94
Wasswa and Zeng 2020	Sagamore Lake - pH 8.5	Whole Water (Lake)	5.26	3.88	15.85	13.76	1.45	290-550	1.47
Wasswa and Zeng 2020	South Lake - pH 8.5	Whole Water (Lake)	7.01	2.35	17.98	13.88	1.52	290-550	2.06
Wasswa and Zeng 2020	Squaw Lake - pH 8.5	Whole Water (Lake)	7.53	2.24	17.95	13.76	1.56	290-550	2.57
Wasswa and Zeng 2020	Willis Lake - pH 8.5	Whole Water (Lake)	5.99	2.83	16.67	14.68	1.45	290-550	1.77
Wasswa and Zeng 2020	Wolf Lake - pH 8.5	Whole Water (Lake)	7.76	2.06	16.68	11.69	1.57	290-550	1.95
Wasswa and Zeng 2020	Arbutus Lake - pH 4.5 + Al	Whole Water (Lake)	7.47	2.45	18.14	18.16	1.57	290-550	2.29
Wasswa and Zeng 2020	Big Moose Lake - pH 4.5 + Al	Whole Water (Lake)	6.75	2.96	18.11	16.76	1.71	290-550	2.03
Wasswa and Zeng 2020	Black Pond - pH 4.5 + Al	Whole Water (Lake)	7.14	3.02	16.74	13.69	1.56	290-550	1.49
Wasswa and Zeng 2020	Dart Lake - pH 4.5 + Al	Whole Water (Lake)	7.23	2.70	18.91	23.19	1.68	290-550	2.47

Table S18. Summary of $\Phi_{app, {}^1O_2}$ and optical data (continued)

Reference	Sample ID	Sample Classification	*E2:E3*	$SUVA_{254}$ (L mg C^{-1}•m^{-1})	$S_{290–400}$ / $S_{300–400}$ / $S_{290–450}$ / $S_{295–400}$ (μm^{-1})	$S_{300–600}$ / $S_{300–500}$ / $S_{300–700}$ / $S_{275–500}$ (μm^{-1})	FI	Wavelength Range (nm)	$\Phi_{app, {}^1O_2}$ (×10^{-2} mol mol-photons^{-1})
Wasswa and Zeng 2020	G Lake - pH 4.5 + Al	Whole Water (Lake)	7.40	1.93	17.08	12.68	1.74	290-550	1.66
Wasswa and Zeng 2020	Honnedaga Lake - pH 4.5 + Al	Whole Water (Lake)	6.91	2.94	17.91	19.27	1.77	290-550	1.80
Wasswa and Zeng 2020	Limekiln Lake - pH 4.5 + Al	Whole Water (Lake)	9.31	2.05	19.73	16.70	1.71	290-550	2.55
Wasswa and Zeng 2020	Little Hope Pond - pH 4.5 + Al	Whole Water (Lake)	5.03	3.57	15.00	13.08	1.56	290-550	0.92
Wasswa and Zeng 2020	Moss Lake - pH 4.5 + Al	Whole Water (Lake)	7.50	2.82	18.13	18.22	1.62	290-550	1.99
Wasswa and Zeng 2020	North Lake - pH 4.5 + Al	Whole Water (Lake)	5.65	3.38	16.56	14.70	1.67	290-550	1.71
Wasswa and Zeng 2020	Lake Rondaxe - pH 4.5 + Al	Whole Water (Lake)	10.70	1.83	22.91	28.29	1.81	290-550	3.46
Wasswa and Zeng 2020	Sagamore Lake - pH 4.5 + Al	Whole Water (Lake)	5.32	3.74	15.89	13.79	1.60	290-550	1.39
Wasswa and Zeng 2020	South Lake - pH 4.5 + Al	Whole Water (Lake)	7.66	2.38	18.43	16.90	1.64	290-550	1.98
Wasswa and Zeng 2020	Squaw Lake - pH 4.5 + Al	Whole Water (Lake)	8.40	2.35	18.60	15.89	1.68	290-550	2.40
Wasswa and Zeng 2020	Willis Lake - pH 4.5 + Al	Whole Water (Lake)	6.55	2.75	17.84	19.85	1.56	290-550	1.78
Wasswa and Zeng 2020	Wolf Lake - ppH 4.5 + Al	Whole Water (Lake)	9.52	1.86	20.22	22.18	1.69	290-550	2.44
Wasswa and Zeng 2020	Arbutus Lake - pH 8.5 + Fe	Whole Water (Lake)	6.49	3.01	16.33	11.59	1.49	290-550	2.07
Wasswa and Zeng 2020	Big Moose Lake - pH 8.5 + Fe	Whole Water (Lake)	6.64	3.27	18.67	22.17	1.49	290-550	2.63
Wasswa and Zeng 2020	Black Pond - pH 8.5 + Fe	Whole Water (Lake)	6.56	3.03	18.22	25.30	1.47	290-550	2.72
Wasswa and Zeng 2020	Dart Lake - pH 8.5 + Fe	Whole Water (Lake)	6.99	3.14	19.76	28.10	1.48	290-550	3.62
Wasswa and Zeng 2020	G Lake - pH 8.5 + Fe	Whole Water (Lake)	7.06	2.25	19.18	25.10	1.57	290-550	3.31
Wasswa and Zeng 2020	Honnedaga Lake - pH 8.5 + Fe	Whole Water (Lake)	5.95	3.57	17.59	20.12	1.54	290-550	2.21
Wasswa and Zeng 2020	Limekiln Lake - pH 8.5 + Fe	Whole Water (Lake)	7.10	2.36	18.80	24.06	1.51	290-550	2.91
Wasswa and Zeng 2020	Little Hope Pond - pH 8.5 + Fe	Whole Water (Lake)	5.19	4.10	16.32	18.10	1.42	290-550	1.43
Wasswa and Zeng 2020	Moss Lake - pH 8.5 + Fe	Whole Water (Lake)	6.60	3.03	18.10	20.32	1.49	290-550	2.63
Wasswa and Zeng 2020	North Lake - pH 8.5 + Fe	Whole Water (Lake)	5.38	3.69	17.27	18.99	1.42	290-550	2.09
Wasswa and Zeng 2020	Lake Rondaxe - pH 8.5 + Fe	Whole Water (Lake)	5.66	2.29	16.18	12.93	1.58	290-550	1.93
Wasswa and Zeng 2020	Sagamore Lake - pH 8.5 + Fe	Whole Water (Lake)	5.06	4.03	16.20	16.10	1.46	290-550	1.58
Wasswa and Zeng 2020	South Lake - pH 8.5 + Fe	Whole Water (Lake)	5.53	2.87	15.69	14.22	1.49	290-550	1.63
Wasswa and Zeng 2020	Squaw Lake - pH 8.5 + Fe	Whole Water (Lake)	5.11	2.71	13.99	9.99	1.49	290-550	1.29
Wasswa and Zeng 2020	Willis Lake - pH 8.5 + Fe	Whole Water (Lake)	5.07	3.27	15.18	12.83	1.47	290-550	1.30
Wasswa and Zeng 2020	Wolf Lake - pH 8.5 + Fe	Whole Water (Lake)	6.35	2.40	16.78	16.14	1.46	290-550	1.86
Wasswa and Zeng 2020	Suwannee River natural organic matter (SRNOM)	IHSS Isolate (SRNOM)	4.13	4.11	14.66	14.94	1.36	290-550	1.96
Wasswa and Zeng 2020	Suwannee River fulvic acid (SRFA)	IHSS Isolate (SRFA)	4.83	3.64	16.21	16.64	1.32	290-550	2.07
Wasswa and Zeng 2020	Suwannee River humic acid (SRHA)	IHSS Isolate (SRHA)	3.34	5.09	12.70	13.15	1.13	290-550	1.14
Zhao and Chen 2020 [178]	SRFA	IHSS Isolate (SRFA)	NA	NA	NA	NA	NA	290-500	1.61
Zhou and Niu 2020 [179]	SRFA	IHSS Isolate (SRFA)	4.28	NA	NA	NA	NA	290-400	1.23
Zhou and Niu 2020	SRHA	IHSS Isolate (SRHA)	3.02	NA	NA	NA	NA	290-400	0.74
Zhou and Niu 2020	SRNOM	IHSS Isolate (SRNOM)	4.21	NA	NA	NA	NA	290-400	1.44
Zhou and Niu 2020	PLFA	IHSS Isolate (PLFA)	4.37	NA	NA	NA	NA	290-400	2.90
Zhou and Niu 2020	NAFA	IHSS Isolate (NFA)	3.61	NA	NA	NA	NA	290-400	0.92
Zhou and Niu 2020	MRNOM	IHSS Isolate (UMRNOM)	4.60	NA	NA	NA	NA	290-400	2.11
Zhou and Niu 2020	SDOM	RO/ED Isolate (Seawater)	8.09	NA	NA	NA	NA	290-400	3.31
Wan and Chen 2020	HA + UV 240 min	XAD Fraction (Soil, UV)	2.56	4.97	NA	NA	1.28	300-600	1.41
Guo and Chen 2021 [180]	SDOM-1	RO/ED Isolate (Seawater)	NA	2.32	NA	NA	1.71	290-400	0.92
Guo and Chen 2021	SDOM-2	RO/ED Isolate (Seawater)	NA	1.81	NA	NA	1.63	290-400	0.75
Guo and Chen 2021	NRNOM	IHSS Isolate (NRNOM)	NA	7.77	NA	NA	NA	290-400	0.55
Leresche and Rosario-Ortiz 2021 [181]	SRFA (1S101F) pH 7 ozone 0 mmol O3 mmol C	IHSS Isolate (SRFA)	4.62	4.19	NA	NA	NA	340-410	1.00
Leresche and Rosario-Ortiz 2021	SRFA pH 7 ozone 0.025 mmol O3 mmol C	IHSS Isolate (SRFA, O3)	4.94	3.87	NA	NA	NA	340-410	1.05
Leresche and Rosario-Ortiz 2021	SRFA pH 7 ozone 0.055 mmol O3 mmol C	IHSS Isolate (SRFA, O3)	5.13	3.60	NA	NA	NA	340-410	1.15
Leresche and Rosario-Ortiz 2021	SRFA pH 7 ozone 0.1 mmol O3 mmol C	IHSS Isolate (SRFA, O3)	5.19	3.13	NA	NA	NA	340-410	1.53
Leresche and Rosario-Ortiz 2021	SRFA pH 7 ozone 0.15 mmol O3 mmol C	IHSS Isolate (SRFA, O3)	5.38	2.65	NA	NA	NA	340-410	1.69
Leresche and Rosario-Ortiz 2021	SRFA pH 7 ozone 0.2 mmol O3 mmol C	IHSS Isolate (SRFA, O3)	6.20	1.95	NA	NA	NA	340-410	2.35
Leresche and Rosario-Ortiz 2021	SRFA pH 7 ozone 0.25 mmol O3 mmol C	IHSS Isolate (SRFA, O3)	7.03	1.67	NA	NA	NA	340-410	3.02
Leresche and Rosario-Ortiz 2021	SRFA pH 7 ozone 0.35 mmol O3 mmol C	IHSS Isolate (SRFA, O3)	8.80	1.03	NA	NA	NA	340-410	4.94

**Table S18.** Summary of $\Phi_{app, {}^1O_2}$ and optical data (continued)									
Reference	Sample ID	Sample Classification	*E2:E3*	$SUVA_{254}$ (L mg C^{-1}•m^{-1})	$S_{290–400}$ / $S_{300–400}$ / $S_{290–450}$ / $S_{295–400}$ (µm^{-1})	$S_{300–600}$ / $S_{300–500}$ / $S_{300–700}$ / $S_{275–500}$ (µm^{-1})	FI	Wavelength Range (nm)	$\Phi_{app, {}^1O_2}$ (×10^{-2} mol mol-photons^{-1})
Leresche and Rosario-Ortiz 2021	SRFA pH 7 ozone 0.5 mmol O3 mmol C	IHSS Isolate (SRFA, O3)	9.24	0.73	NA	NA	NA	340-410	7.47
Leresche and Rosario-Ortiz 2021	PLFA (1R109F) pH 7 ozone 0 mmol O3 mmol C	IHSS Isolate (PLFA)	4.94	2.56	NA	NA	NA	340-410	1.92
Leresche and Rosario-Ortiz 2021	PLFA pH 7 ozone 0.025 mmol O3 mmol C	IHSS Isolate (PLFA, O3)	6.01	2.20	NA	NA	NA	340-410	2.67
Leresche and Rosario-Ortiz 2021	PLFA pH 7 ozone 0.055 mmol O3 mmol C	IHSS Isolate (PLFA, O3)	6.84	1.96	NA	NA	NA	340-410	3.77
Leresche and Rosario-Ortiz 2021	PLFA pH 7 ozone 0.1 mmol O3 mmol C	IHSS Isolate (PLFA, O3)	9.05	1.57	NA	NA	NA	340-410	4.31
Leresche and Rosario-Ortiz 2021	PLFA pH 7 ozone 0.15 mmol O3 mmol C	IHSS Isolate (PLFA, O3)	13.10	1.19	NA	NA	NA	340-410	5.38
Leresche and Rosario-Ortiz 2021	PLFA pH 7 ozone 0.2 mmol O3 mmol C	IHSS Isolate (PLFA, O3)	18.73	0.90	NA	NA	NA	340-410	6.51
Leresche and Rosario-Ortiz 2021	PLFA pH 7 ozone 0.25 mmol O3 mmol C	IHSS Isolate (PLFA, O3)	20.25	0.78	NA	NA	NA	340-410	7.59
Leresche and Rosario-Ortiz 2021	PLFA pH 7 ozone 0.35 mmol O3 mmol C	IHSS Isolate (PLFA, O3)	26.01	0.64	NA	NA	NA	340-410	9.48
Leresche and Rosario-Ortiz 2021	PLFA pH 7 ozone 0.5 mmol O3 mmol C	IHSS Isolate (PLFA, O3)	29.94	0.50	NA	NA	NA	340-410	13.17
Li and Tian 2021 [182]	DBC-W200	DBC (pyDOM)	3.12	3.07	NA	NA	NA	Natural sunlight	2.37
Li and Tian 2021	DBC-W300	DBC (pyDOM)	6.01	2.21	NA	NA	NA	Natural sunlight	2.66
Li and Tian 2021	DBC-W400	DBC (pyDOM)	5.51	1.27	NA	NA	NA	Natural sunlight	3.19
Li and Tian 2021	DBC-W500	DBC (pyDOM)	5.00	0.31	NA	NA	NA	Natural sunlight	1.19
Li and Tian 2021	DBC-W600	DBC (pyDOM)	4.60	0.27	NA	NA	NA	Natural sunlight	0.56
Li and Tian 2021	DBC-R200	DBC (pyDOM)	3.29	2.66	NA	NA	NA	Natural sunlight	0.87
Li and Tian 2021	DBC-R300	DBC (pyDOM)	3.89	1.91	NA	NA	NA	Natural sunlight	1.25
Li and Tian 2021	DBC-R400	DBC (pyDOM)	2.66	2.05	NA	NA	NA	Natural sunlight	1.45
Li and Tian 2021	DBC-R500	DBC (pyDOM)	2.25	0.64	NA	NA	NA	Natural sunlight	0.80
Li and Tian 2021	DBC-R600	DBC (pyDOM)	2.20	0.21	NA	NA	NA	Natural sunlight	0.29
Li and Tian 2021	DBC-B200	DBC (pyDOM)	3.71	2.06	NA	NA	NA	Natural sunlight	1.17
Li and Tian 2021	DBC-B300	DBC (pyDOM)	8.57	1.68	NA	NA	NA	Natural sunlight	1.55
Li and Tian 2021	DBC-B400	DBC (pyDOM)	2.56	0.89	NA	NA	NA	Natural sunlight	2.31
Li and Tian 2021	DBC-B500	DBC (pyDOM)	4.23	0.20	NA	NA	NA	Natural sunlight	1.00
Li and Tian 2021	DBC-B600	DBC (pyDOM)	3.63	0.09	NA	NA	NA	Natural sunlight	0.85
Li and Tian 2021	DBC-C200	DBC (pyDOM)	3.47	3.02	NA	NA	NA	Natural sunlight	1.12
Li and Tian 2021	DBC-C300	DBC (pyDOM)	7.80	2.39	NA	NA	NA	Natural sunlight	2.23
Li and Tian 2021	DBC-C400	DBC (pyDOM)	9.79	2.01	NA	NA	NA	Natural sunlight	3.09
Li and Tian 2021	DBC-C500	DBC (pyDOM)	2.46	0.23	NA	NA	NA	Natural sunlight	0.37
Li and Tian 2021	DBC-C600	DBC (pyDOM)	1.72	0.07	NA	NA	NA	Natural sunlight	0.23
Li and Tian 2021	DBC-P200	DBC (pyDOM)	5.99	2.43	NA	NA	NA	Natural sunlight	0.79
Li and Tian 2021	DBC-P300	DBC (pyDOM)	4.89	1.50	NA	NA	NA	Natural sunlight	1.34
Li and Tian 2021	DBC-P400	DBC (pyDOM)	3.25	0.99	NA	NA	NA	Natural sunlight	1.47
Li and Tian 2021	DBC-P500	DBC (pyDOM)	2.86	0.56	NA	NA	NA	Natural sunlight	0.34
Li and Tian 2021	DBC-P600	DBC (pyDOM)	1.69	0.31	NA	NA	NA	Natural sunlight	0.29
Lv and Zhang 2021 [183]	SRFA (2S101F)	IHSS Isolate (SRFA)	NA	3.67	NA	NA	NA	Unknown	1.80
Lv and Zhang 2021	SRFA Fe/C 3.75	IHSS Isolate (SRFA, Ferrihydrite)	NA	1.76	NA	NA	NA	Unknown	2.30
Lv and Zhang 2021	SRFA Fe/C 11.25	IHSS Isolate (SRFA, Ferrihydrite)	NA	0.58	NA	NA	NA	Unknown	3.28
Lv and Zhang 2021	NAFA (1R105F)	IHSS Isolate (NFA)	NA	3.93	NA	NA	NA	Unknown	1.53
Lv and Zhang 2021	NAFA Fe/C 3.75	IHSS Isolate (NFA, Ferrihydrite)	NA	2.21	NA	NA	NA	Unknown	1.72
Lv and Zhang 2021	NAFA Fe/C 11.25	IHSS Isolate (NFA, Ferrihydrite)	NA	0.66	NA	NA	NA	Unknown	2.33
Lv and Zhang 2021	MNOM (1R110N)	IHSS Isolate (UMRNOM)	NA	2.76	NA	NA	NA	Unknown	2.28
Lv and Zhang 2021	MNOM Fe/C 3.75	IHSS Isolate (MRNOM, Ferrihydrite)	NA	1.13	NA	NA	NA	Unknown	3.29
Lv and Zhang 2021	MNOM Fe/C 11.25	IHSS Isolate (MRNOM, Ferrihydrite)	NA	0.53	NA	NA	NA	Unknown	6.16
Lv and Zhang 2021	SRNOM (2R101N)	IHSS Isolate (SRNOM)	NA	2.98	NA	NA	NA	Unknown	1.81
Lv and Zhang 2021	SRNOM Fe/C 3.75	IHSS Isolate (SRNOM, Ferrihydrite)	NA	1.20	NA	NA	NA	Unknown	3.32
Lv and Zhang 2021	SRNOM Fe/C 11.25	IHSS Isolate (SRNOM, Ferrihydrite)	NA	0.50	NA	NA	NA	Unknown	3.64
Wan and Chen 2021a [184]	rice	DBC (pyDOM)	3.73	4.70	NA	NA	NA	300-400	3.75
Wan and Chen 2021a	corn	DBC (pyDOM)	4.25	4.18	NA	NA	NA	300-400	3.96
Wan and Chen 2021a	wheat	DBC (pyDOM)	5.29	3.30	NA	NA	NA	300-400	4.25
Wan and Chen 2021b [185]	EfOM	C18 Extract (WWTP Effluent)	4.24	2.21	NA	NA	NA	300-400	3.85

**Table S18.** Summary of $\Phi_{app, {}^1O_2}$ and optical data (continued)									
Reference	Sample ID	Sample Classification	*E2:E3*	$SUVA_{254}$ (L mg C^{-1}•m^{-1})	$S_{290–400}$ / $S_{300–400}$ / $S_{290–450}$ / $S_{295–400}$ (μm^{-1})	$S_{300–600}$ / $S_{300–500}$ / $S_{300–700}$ / $S_{275–500}$ (μm^{-1})	FI	Wavelength Range (nm)	$\Phi_{app, {}^1O_2}$ (×10^{-2} mol mol-photons^{-1})
Wan and Chen 2021b	EfOM + 1.0 mg/L Cl2	C18 Extract (WWTP Effluent, Cl2)	4.47	2.14	NA	NA	NA	300-400	4.28
Wan and Chen 2021b	EfOM + 2.0 mg/L Cl2	C18 Extract (WWTP Effluent, Cl2)	4.51	2.05	NA	NA	NA	300-400	4.96
Wan and Chen 2021b	EfOM + 5.0 mg/L Cl2	C18 Extract (WWTP Effluent, Cl2)	4.58	1.82	NA	NA	NA	300-400	6.23
Wan and Chen 2021b	EfOM + 10.0 mg/L Cl2	C18 Extract (WWTP Effluent, Cl2)	4.70	1.57	NA	NA	NA	300-400	8.36
Wan and Chen 2021b	Henan Changsheng Industrial Fulvic acid (FA)	XAD Fraction (Soil)	2.30	9.23	NA	NA	NA	300-400	2.31
Wan and Chen 2021b	FA + 1.0 mg/L Cl2	XAD Fraction (Soil, Cl2)	2.35	9.21	NA	NA	NA	300-400	2.58
Wan and Chen 2021b	FA + 2.0 mg/L Cl2	XAD Fraction (Soil, Cl2)	2.48	9.18	NA	NA	NA	300-400	3.08
Wan and Chen 2021b	FA + 5.0 mg/L Cl2	XAD Fraction (Soil, Cl2)	2.58	7.77	NA	NA	NA	300-400	3.78
Wan and Chen 2021b	FA + 10.0 mg/L Cl2	XAD Fraction (Soil, Cl2)	2.61	8.35	NA	NA	NA	300-400	4.94
Wan and Chen 2021b	Aldrich humic acid (HA)	XAD Fraction (Soil)	2.77	6.75	NA	NA	NA	300-400	2.24
Wan and Chen 2021b	HA + 1.0 mg/L Cl2	XAD Fraction (Soil, Cl2)	2.81	6.68	NA	NA	NA	300-400	2.38
Wan and Chen 2021b	HA + 2.0 mg/L Cl2	XAD Fraction (Soil, Cl2)	2.84	6.47	NA	NA	NA	300-400	2.60
Wan and Chen 2021b	HA + 5.0 mg/L Cl2	XAD Fraction (Soil, Cl2)	3.02	6.25	NA	NA	NA	300-400	3.49
Wan and Chen 2021b	HA + 10.0 mg/L Cl2	XAD Fraction (Soil, Cl2)	3.35	6.15	NA	NA	NA	300-400	4.38
Wu and Chu 2021 [31]	SRNOM (1R101N)	IHSS Isolate (SRNOM)	NA	NA	NA	NA	NA	375	0.84
Wu and Chu 2021	SRNOM (1R101N)	IHSS Isolate (SRNOM)	NA	NA	NA	NA	NA	387	0.55
Wu and Chu 2021	SRNOM (1R101N)	IHSS Isolate (SRNOM)	NA	NA	NA	NA	NA	425	0.55
Wu and Chu 2021	SRNOM (1R101N)	IHSS Isolate (SRNOM)	NA	NA	NA	NA	NA	461	0.35
Wu and Chu 2021	SRNOM (1R101N)	IHSS Isolate (SRNOM)	NA	NA	NA	NA	NA	490	0.16
Zeng and Wang 2021 [186]	Irrigation	Whole Water (Paddy)	4.25	4.51	NA	NA	4.07	290-400	11.01
Zeng and Wang 2021	Seedling1	Whole Water (Paddy)	3.84	4.01	NA	NA	3.88	290-400	12.72
Zeng and Wang 2021	Seedling2	Whole Water (Paddy)	4.71	7.30	NA	NA	4.54	290-400	7.65
Zeng and Wang 2021	Seedling3	Whole Water (Paddy)	4.56	6.45	NA	NA	4.13	290-400	9.29
Zeng and Wang 2021	Seedling4	Whole Water (Paddy)	4.13	4.52	NA	NA	2.29	290-400	15.59
Zeng and Wang 2021	Tillering1	Whole Water (Paddy)	5.29	7.22	NA	NA	2.35	290-400	3.95
Zeng and Wang 2021	Tillering2	Whole Water (Paddy)	5.97	9.07	NA	NA	2.32	290-400	5.00
Zeng and Wang 2021	Tillering3	Whole Water (Paddy)	5.23	12.65	NA	NA	2.34	290-400	3.14
Zeng and Wang 2021	Tillering4	Whole Water (Paddy)	3.33	5.50	NA	NA	3.99	290-400	2.00
Zeng and Wang 2021	Jointing1	Whole Water (Paddy)	6.96	4.10	NA	NA	2.37	290-400	8.99
Zeng and Wang 2021	Jointing2	Whole Water (Paddy)	7.49	5.08	NA	NA	2.39	290-400	13.39
Zeng and Wang 2021	Heading1	Whole Water (Paddy)	3.13	6.37	NA	NA	3.55	290-400	4.25
Zeng and Wang 2021	Heading2	Whole Water (Paddy)	3.08	5.74	NA	NA	3.57	290-400	3.92
Zhou and Chen 2021 [187]	SRFA initial	IHSS Isolate (SRFA)	0.99	5.12	NA	NA	NA	290-400	1.25
Zhou and Chen 2021	SRFA UV/Cl2 0 μM	IHSS Isolate (SRFA, UV/Cl2)	0.76	4.91	NA	NA	NA	290-400	0.81
Zhou and Chen 2021	SRFA UV/Cl2 25 μM	IHSS Isolate (SRFA, UV/Cl2)	1.09	4.25	NA	NA	NA	290-400	1.15
Zhou and Chen 2021	SRFA UV/Cl2 50 μM	IHSS Isolate (SRFA, UV/Cl2)	1.08	4.02	NA	NA	NA	290-400	1.24
Zhou and Chen 2021	SRFA UV/Cl2 75 μM	IHSS Isolate (SRFA, UV/Cl2)	1.36	3.42	NA	NA	NA	290-400	1.36
Zhou and Chen 2021	SRFA UV/Cl2 100 μM	IHSS Isolate (SRFA, UV/Cl2)	1.68	2.93	NA	NA	NA	290-400	1.32
Zhou and Chen 2021	SRFA UV/Cl2 125 μM	IHSS Isolate (SRFA, UV/Cl2)	2.17	2.66	NA	NA	NA	290-400	1.44
Zhou and Chen 2021	SRFA UV/Cl2 150 μM	IHSS Isolate (SRFA, UV/Cl2)	2.47	2.5	NA	NA	NA	290-400	1.50
Zhou and Chen 2021	SRFA UV/Cl2 200 μM	IHSS Isolate (SRFA, UV/Cl2)	3.87	1.42	NA	NA	NA	290-400	1.70
Zhou and Chen 2021	SRNOM initial	IHSS Isolate (SRNOM)	1.34	3.46	NA	NA	NA	290-400	1.00
Zhou and Chen 2021	SRNOM UV/Cl2 0 μM	IHSS Isolate (SRNOM, UV/Cl2)	1.18	3.26	NA	NA	NA	290-400	0.40
Zhou and Chen 2021	SRNOM UV/Cl2 25 μM	IHSS Isolate (SRNOM, UV/Cl2)	0.98	3.17	NA	NA	NA	290-400	0.82
Zhou and Chen 2021	SRNOM UV/Cl2 50 μM	IHSS Isolate (SRNOM, UV/Cl2)	1.82	2.57	NA	NA	NA	290-400	0.96
Zhou and Chen 2021	SRNOM UV/Cl2 75 μM	IHSS Isolate (SRNOM, UV/Cl2)	1.99	2.22	NA	NA	NA	290-400	1.13
Zhou and Chen 2021	SRNOM UV/Cl2 100 μM	IHSS Isolate (SRNOM, UV/Cl2)	3.14	1.66	NA	NA	NA	290-400	1.19
Zhou and Chen 2021	SRNOM UV/Cl2 125 μM	IHSS Isolate (SRNOM, UV/Cl2)	3.64	1.37	NA	NA	NA	290-400	1.42
Zhou and Chen 2021	SRNOM UV/Cl2 150 μM	IHSS Isolate (SRNOM, UV/Cl2)	5.62	0.99	NA	NA	NA	290-400	1.78
Zhou and Chen 2021	SRNOM UV/Cl2 200 μM	IHSS Isolate (SRNOM, UV/Cl2)	8.23	0.72	NA	NA	NA	290-400	2.33
Zhou and Yuan 2021 [188]	M-EPS (Shewanella oneidensis MR-1)	EPS	NA	2.81	NA	NA	0.69	280-700	4.01

Table S18. Summary of $\Phi_{app, ^1O_2}$ and optical data (continued)

Reference	Sample ID	Sample Classification	*E2:E3*	$SUVA_{254}$ (L mg C^{-1}•m^{-1})	$S_{290–400}$ / $S_{300–400}$ / $S_{290–450}$ / $S_{295–400}$ (μm^{-1})	$S_{300–600}$ / $S_{300–500}$ / $S_{300–700}$ / $S_{275–500}$ (μm^{-1})	FI	Wavelength Range (nm)	$\Phi_{app, ^1O_2}$ ($\times 10^{-2}$ mol mol-photons^{-1})
Zhou and Yuan 2021	E-EPS (Escherichia coli)	EPS	NA	1.32	NA	NA	1.52	280-700	4.08
Zhou and Yuan 2021	S-EPS (Mixed culture sewage sludge flocs)	EPS	NA	4.43	NA	NA	1.31	280-700	7.4
Hu and Zhang 2022 [189]	DOMACS	WEOM (Bio-Stabilization Sludge)	4.09	1.49	NA	NA	NA	300-700	0.52
Hu and Zhang 2022	DOMADS	WEOM (Bio-Stabilization Sludge)	3.72	0.92	NA	NA	NA	300-700	0.52
Liao and Yuan 2022 [190]	Wet Season S1	Whole Water (WW-Impacted River)	6.17	1.77	NA	NA	1.34	290-400	2.39
Liao and Yuan 2022	Wet Season S2	Whole Water (WW-Impacted River)	5.69	1.25	NA	NA	1.73	290-400	4.11
Liao and Yuan 2022	Wet Season S3	Whole Water (WW-Impacted River)	6.53	2.06	NA	NA	1.56	290-400	3.85
Liao and Yuan 2022	Wet Season S4	Whole Water (WW-Impacted River)	5.77	1.24	NA	NA	1.59	290-400	4.56
Liao and Yuan 2022	Wet Season S5	Whole Water (WW-Impacted River)	6.20	2.15	NA	NA	1.73	290-400	4.00
Liao and Yuan 2022	Wet Season S6	Whole Water (WW-Impacted River)	6.09	2.08	NA	NA	1.67	290-400	3.56
Liao and Yuan 2022	Wet Season S7	Whole Water (WW-Impacted River)	6.22	2.09	NA	NA	1.52	290-400	3.31
Liao and Yuan 2022	Wet Season S8	Whole Water (WW-Impacted River)	6.38	1.52	NA	NA	1.38	290-400	2.14
Liao and Yuan 2022	Wet Season S9	Whole Water (WW-Impacted River)	6.80	1.97	NA	NA	1.66	290-400	3.53
Liao and Yuan 2022	Wet Season S10	Whole Water (WW-Impacted River)	6.75	2.53	NA	NA	1.61	290-400	1.38
Liao and Yuan 2022	Wet Season S11	Whole Water (WW-Impacted River)	5.75	2.38	NA	NA	1.57	290-400	0.95
Liao and Yuan 2022	Dry Season S1	Whole Water (WW-Impacted River)	6.00	2.60	NA	NA	1.49	290-400	3.15
Liao and Yuan 2022	Dry Season S2	Whole Water (WW-Impacted River)	6.30	2.46	NA	NA	1.58	290-400	2.71
Liao and Yuan 2022	Dry Season S3	Whole Water (WW-Impacted River)	7.14	2.01	NA	NA	1.70	290-400	3.09
Liao and Yuan 2022	Dry Season S4	Whole Water (WW-Impacted River)	6.56	2.59	NA	NA	1.62	290-400	1.89
Liao and Yuan 2022	Dry Season S5	Whole Water (WW-Impacted River)	7.29	2.33	NA	NA	1.59	290-400	1.17
Liao and Yuan 2022	Dry Season S6	Whole Water (WW-Impacted River)	7.25	2.17	NA	NA	1.54	290-400	1.49
Liao and Yuan 2022	Dry Season S7	Whole Water (WW-Impacted River)	7.43	2.47	NA	NA	1.68	290-400	1.05
Liao and Yuan 2022	Dry Season S8	Whole Water (WW-Impacted River)	7.29	2.39	NA	NA	1.82	290-400	0.94
Liao and Yuan 2022	Dry Season S9	Whole Water (WW-Impacted River)	6.67	2.18	NA	NA	1.56	290-400	3.32
Liao and Yuan 2022	Dry Season S10	Whole Water (WW-Impacted River)	7.33	1.96	NA	NA	1.48	290-400	1.70
Liao and Yuan 2022	Dry Season S11	Whole Water (WW-Impacted River)	6.00	1.41	NA	NA	1.74	290-400	0.88
Liu and Zhu 2022 [191]	DBC	DBC (pyDOM)	NA	NA	NA	NA	NA	300-400	5.90
Liu and Zhu 2022	LDOC	WEOM (Biochar)	NA	NA	NA	NA	NA	300-400	1.70
Ma and Zhang 2022 [192]	I1_<1 kDa	UF Fraction (M. aeruginosa)	24.00	1.11	NA	NA	1.97	290-400	25.21
Ma and Zhang 2022	I2_1–5 kDa	UF Fraction (M. aeruginosa)	10.00	1.38	NA	NA	1.92	290-400	14.28
Ma and Zhang 2022	I3_5–10 kDa	UF Fraction (M. aeruginosa)	15.50	1.43	NA	NA	1.88	290-400	9.92
Ma and Zhang 2022	I4_10–30 kDa	UF Fraction (M. aeruginosa)	16.00	1.47	NA	NA	1.90	290-400	15.93
Ma and Zhang 2022	I5_30–100 kDa	UF Fraction (M. aeruginosa)	13.00	1.80	NA	NA	1.83	290-400	18.64
Ma and Zhang 2022	I6_>100 kDa	UF Fraction (M. aeruginosa)	5.75	2.12	NA	NA	1.75	290-400	9.10
Ma and Zhang 2022	I0_bulk	UF Fraction (M. aeruginosa)	8.20	1.89	NA	NA	1.86	290-400	17.50
Ma and Zhang 2022	SRFA	IHSS Isolate (SRFA)	4.34	11.19	NA	NA	1.27	290-400	2.15
Song and Jiang 2022 [193]	Flood	Whole Water (Lake)	5.19	11.83	NA	NA	1.21	290-600	1.01
Song and Jiang 2022	Drought	Whole Water (Lake)	4.97	6.63	NA	NA	2.30	290-600	6.66
Wan and Chen 2022a [194]	EfOM-1	C18 Extract (WWTP Effluent)	6.03	1.70	NA	17.00	3.11	290-400	6.33
Wan and Chen 2022a	EfOM-1 + KMnO4 0.5 mg L^{-1}	C18 Extract (WWTP Effluent, KMnO4)	6.88	1.61	NA	18.30	2.46	290-400	6.97
Wan and Chen 2022a	EfOM-1 + KMnO4 1.0 mg L^{-1}	C18 Extract (WWTP Effluent, KMnO4)	7.27	1.51	NA	18.60	2.58	290-400	7.06
Wan and Chen 2022a	EfOM-1 + KMnO4 2.0 mg L^{-1}	C18 Extract (WWTP Effluent, KMnO4)	8.19	1.49	NA	19.80	2.27	290-400	7.32
Wan and Chen 2022a	EfOM-1 + KMnO4 4.0 mg L^{-1}	C18 Extract (WWTP Effluent, KMnO4)	8.60	1.38	NA	19.40	2.28	290-400	7.22
Wan and Chen 2022a	EfOM-2	C18 Extract (WWTP Effluent)	4.13	1.44	NA	13.20	2.41	290-400	4.52
Wan and Chen 2022a	EfOM-2 + KMnO4 0.5 mg L^{-1}	C18 Extract (WWTP Effluent, KMnO4)	4.32	1.33	NA	13.60	2.26	290-400	4.73
Wan and Chen 2022a	EfOM-2 + KMnO4 1.0 mg L^{-1}	C18 Extract (WWTP Effluent, KMnO4)	4.41	1.28	NA	14.40	2.15	290-400	4.88
Wan and Chen 2022a	EfOM-2 + KMnO4 2.0 mg L^{-1}	C18 Extract (WWTP Effluent, KMnO4)	4.47	1.20	NA	13.90	2.15	290-400	4.91
Wan and Chen 2022a	EfOM-2 + KMnO4 4.0 mg L^{-1}	C18 Extract (WWTP Effluent, KMnO4)	4.65	1.14	NA	14.20	2.12	290-400	5.00
Wan and Chen 2022a	Henan Changsheng Industrial Fulvic acid (FA)	XAD Fraction (Soil)	2.37	8.41	NA	7.40	0.60	290-400	2.59
Wan and Chen 2022a	FA + KMnO4 0.5 mg L^{-1}	XAD Fraction (Soil, KMnO4)	2.41	8.19	NA	7.50	0.63	290-400	2.64
Wan and Chen 2022a	FA + KMnO4 1.0 mg L^{-1}	XAD Fraction (Soil, KMnO4)	2.45	8.23	NA	7.70	0.63	290-400	2.73

Table S18. Summary of $\Phi_{app,\ ^1O_2}$ and optical data (continued)									
Reference	Sample ID	Sample Classification	*E2:E3*	SUVA$_{254}$ (L mg C^{-1}•m^{-1})	$S_{290–400}$ / $S_{300–400}$ / $S_{290–450}$ / $S_{295–400}$ (μm^{-1})	$S_{300–600}$ / $S_{300–500}$ / $S_{300–700}$ / $S_{275–500}$ (μm^{-1})	FI	Wavelength Range (nm)	$\Phi_{app,\ ^1O_2}$ (×10^{-2} mol mol-photons^{-1})
Wan and Chen 2022a	FA + KMnO4 2.0 mg L^{-1}	XAD Fraction (Soil, KMnO4)	2.51	7.87	NA	7.80	0.59	290-400	2.90
Wan and Chen 2022a	FA + KMnO4 4.0 mg L^{-1}	XAD Fraction (Soil, KMnO4)	2.54	7.31	NA	7.90	0.59	290-400	2.95
Wan and Chen 2022a	Aldrich humic acid (HA)	XAD Fraction (Soil)	2.70	5.79	NA	8.60	1.30	290-400	2.22
Wan and Chen 2022a	HA + KMnO4 0.5 mg L^{-1}	XAD Fraction (Soil, KMnO4)	2.72	5.50	NA	9.00	1.34	290-400	2.39
Wan and Chen 2022a	HA + KMnO4 1.0 mg L^{-1}	XAD Fraction (Soil, KMnO4)	2.81	5.24	NA	9.10	1.34	290-400	2.44
Wan and Chen 2022a	HA + KMnO4 2.0 mg L^{-1}	XAD Fraction (Soil, KMnO4)	2.93	5.19	NA	9.40	1.33	290-400	2.44
Wan and Chen 2022a	HA + KMnO4 4.0 mg L^{-1}	XAD Fraction (Soil, KMnO4)	3.03	4.83	NA	9.60	1.30	290-400	2.42
Wan and Chen 2022a	NOM-1	Whole Water (Lake)	6.79	0.84	NA	15.30	2.24	290-400	6.43
Wan and Chen 2022a	NOM-1 + KMnO4 0.5 mg L^{-1}	Whole Water (Lake, KMnO4)	6.93	0.84	NA	15.60	2.25	290-400	6.62
Wan and Chen 2022a	NOM-1 + KMnO4 1.0 mg L^{-1}	Whole Water (Lake, KMnO4)	7.24	0.81	NA	15.70	2.00	290-400	6.54
Wan and Chen 2022a	NOM-1 + KMnO4 2.0 mg L^{-1}	Whole Water (Lake, KMnO4)	7.28	0.75	NA	16.60	2.15	290-400	6.70
Wan and Chen 2022a	NOM-1 + KMnO4 4.0 mg L^{-1}	Whole Water (Lake, KMnO4)	7.39	0.66	NA	18.00	1.94	290-400	6.71
Wan and Chen 2022a	NOM-2	Whole Water (Lake)	5.36	0.80	NA	14.00	2.59	290-400	5.13
Wan and Chen 2022a	NOM-2 + KMnO4 0.5 mg L^{-1}	Whole Water (Lake, KMnO4)	5.50	0.78	NA	15.20	2.60	290-400	5.28
Wan and Chen 2022a	NOM-2 + KMnO4 1.0 mg L^{-1}	Whole Water (Lake, KMnO4)	5.51	0.72	NA	15.60	2.57	290-400	5.30
Wan and Chen 2022a	NOM-2 + KMnO4 2.0 mg L^{-1}	Whole Water (Lake, KMnO4)	6.17	0.68	NA	16.10	2.55	290-400	5.30
Wan and Chen 2022a	NOM-2 + KMnO4 4.0 mg L^{-1}	Whole Water (Lake, KMnO4)	6.21	0.60	NA	16.40	2.51	290-400	5.31
Wan and Chen 2022a	NOM-3	Whole Water (Lake)	5.08	1.00	NA	11.90	2.46	290-400	3.90
Wan and Chen 2022a	NOM-3 + KMnO4 0.5 mg L^{-1}	Whole Water (Lake, KMnO4)	5.58	1.00	NA	13.50	2.32	290-400	4.13
Wan and Chen 2022a	NOM-3 + KMnO4 1.0 mg L^{-1}	Whole Water (Lake, KMnO4)	6.28	0.98	NA	15.30	2.40	290-400	4.30
Wan and Chen 2022a	NOM-3 + KMnO4 2.0 mg L^{-1}	Whole Water (Lake, KMnO4)	6.51	0.93	NA	16.10	2.15	290-400	4.27
Wan and Chen 2022a	NOM-3 + KMnO4 4.0 mg L^{-1}	Whole Water (Lake, KMnO4)	6.94	0.79	NA	16.90	2.12	290-400	4.54
Wan and Chen 2022a	NOM-4	Whole Water (Lake)	7.02	0.67	NA	16.80	2.26	290-400	7.67
Wan and Chen 2022a	NOM-4 + KMnO4 0.5 mg L^{-1}	Whole Water (Lake, KMnO4)	7.26	0.66	NA	17.30	2.28	290-400	7.96
Wan and Chen 2022a	NOM-4 + KMnO4 1.0 mg L^{-1}	Whole Water (Lake, KMnO4)	7.73	0.59	NA	17.40	2.25	290-400	7.89
Wan and Chen 2022a	NOM-4 + KMnO4 2.0 mg L^{-1}	Whole Water (Lake, KMnO4)	7.79	0.54	NA	17.40	2.19	290-400	8.07
Wan and Chen 2022a	NOM-4 + KMnO4 4.0 mg L^{-1}	Whole Water (Lake, KMnO4)	8.13	0.48	NA	18.90	2.16	290-400	8.28
Wan and Chen 2022b [125]	Henan Changsheng Industrial Fulvic acid (FA)	XAD Fraction (Soil)	2.15	7.71	NA	6.60	0.61	290-400	2.82
Wan and Chen 2022b	FA + NaClO	XAD Fraction (Soil, Cl2)	2.75	6.47	NA	9.00	0.77	290-400	4.04
Wan and Chen 2022b	FA + UV254	XAD Fraction (Soil, UV)	2.66	5.72	NA	7.50	0.78	290-400	2.15
Wan and Chen 2022b	FA + O3	XAD Fraction (Soil, O3)	2.31	7.37	NA	7.30	0.67	290-400	2.42
Wan and Chen 2022b	Aldrich humic acid (HA)	XAD Fraction (Soil)	2.54	5.17	NA	8.50	1.07	290-400	1.69
Wan and Chen 2022b	HA + NaClO	XAD Fraction (Soil, Cl2)	3.09	4.71	NA	10.00	1.20	290-400	3.20
Wan and Chen 2022b	HA + UV254	XAD Fraction (Soil, UV)	2.61	4.70	NA	8.90	1.14	290-400	1.53
Wan and Chen 2022b	HA + O3	XAD Fraction (Soil, O3)	2.69	3.49	NA	8.70	1.15	290-400	1.62
Wan and Chen 2022b	EfOM	PPL Extract (WWTP Effluent)	4.04	2.21	NA	13.10	2.53	290-400	4.11
Wan and Chen 2022b	EfOM + NaClO	PPL Extract (WWTP Effluent, Cl2)	4.17	1.81	NA	13.50	2.35	290-400	5.08
Wan and Chen 2022b	EfOM + UV254	PPL Extract (WWTP Effluent, UV)	4.20	1.29	NA	16.60	2.39	290-400	4.84
Wan and Chen 2022b	EfOM + O3	PPL Extract (WWTP Effluent, O3)	6.04	1.51	NA	16.30	2.33	290-400	4.70
Wan and Chen 2022b	WW	Whole Water (WWTP Effluent)	NA	NA	NA	17.90	2.21	290-400	4.94
Wan and Chen 2022b	WW + NaClO	Whole Water (WWTP Effluent, Cl2)	NA	NA	NA	26.10	2.08	290-400	10.16
Wan and Chen 2022b	WW + UV254	Whole Water (WWTP Effluent, UV)	NA	NA	NA	18.20	2.11	290-400	7.01
Wan and Chen 2022b	WW + O3	Whole Water (WWTP Effluent, O3)	NA	NA	NA	28.20	1.80	290-400	7.04
Wan and Chen 2022c [195]	Scindapsus aureus 265 nm	C18+PPL Extract (Root Exudate)	3.97	0.86	NA	NA	1.99	280-410	6.63
Wan and Chen 2022c	Scindapsus aureus 275 nm	C18+PPL Extract (Root Exudate)	3.97	0.86	NA	NA	1.99	280-410	10.52
Wan and Chen 2022c	Scindapsus aureus 285 nm	C18+PPL Extract (Root Exudate)	3.97	0.86	NA	NA	1.99	280-410	8.25
Wan and Chen 2022c	Scindapsus aureus 295 nm	C18+PPL Extract (Root Exudate)	3.97	0.86	NA	NA	1.99	280-410	7.28
Wan and Chen 2022c	Scindapsus aureus 305 nm	C18+PPL Extract (Root Exudate)	3.97	0.86	NA	NA	1.99	280-410	3.58
Wan and Chen 2022c	Scindapsus aureus 335 nm	C18+PPL Extract (Root Exudate)	3.97	0.86	NA	NA	1.99	280-410	2.90
Wan and Chen 2022c	Scindapsus aureus 365 nm	C18+PPL Extract (Root Exudate)	3.97	0.86	NA	NA	1.99	280-410	0.59
Wan and Chen 2022c	Scindapsus aureus 390 nm	C18+PPL Extract (Root Exudate)	3.97	0.86	NA	NA	1.99	280-410	0.57

Table S18. Summary of $\Phi_{app, {}^1O_2}$ and optical data (continued)

Reference	Sample ID	Sample Classification	*E2:E3*	$SUVA_{254}$ (L mg C^{-1}•m^{-1})	$S_{290–400}$ / $S_{300–400}$ / $S_{290–450}$ / $S_{295–400}$ (μm^{-1})	$S_{300–600}$ / $S_{300–500}$ / $S_{300–700}$ / $S_{275–500}$ (μm^{-1})	FI	Wavelength Range (nm)	$\Phi_{app, {}^1O_2}$ (×10^{-2} mol mol-photons^{-1})
Wan and Chen 2022c	Scindapsus aureus 400 nm	C18+PPL Extract (Root Exudate)	3.97	0.86	NA	NA	1.99	280-410	0.33
Wan and Chen 2022c	Pistia stratiotes 265 nm	C18+PPL Extract (Root Exudate)	5.79	1.35	NA	NA	2.20	280-410	10.10
Wan and Chen 2022c	Pistia stratiotes 275 nm	C18+PPL Extract (Root Exudate)	5.79	1.35	NA	NA	2.20	280-410	15.18
Wan and Chen 2022c	Pistia stratiotes 285 nm	C18+PPL Extract (Root Exudate)	5.79	1.35	NA	NA	2.20	280-410	11.01
Wan and Chen 2022c	Pistia stratiotes 295 nm	C18+PPL Extract (Root Exudate)	5.79	1.35	NA	NA	2.20	280-410	8.29
Wan and Chen 2022c	Pistia stratiotes 305 nm	C18+PPL Extract (Root Exudate)	5.79	1.35	NA	NA	2.20	280-410	4.08
Wan and Chen 2022c	Pistia stratiotes 335 nm	C18+PPL Extract (Root Exudate)	5.79	1.35	NA	NA	2.20	280-410	3.58
Wan and Chen 2022c	Pistia stratiotes 365 nm	C18+PPL Extract (Root Exudate)	5.79	1.35	NA	NA	2.20	280-410	1.31
Wan and Chen 2022c	Pistia stratiotes 390 nm	C18+PPL Extract (Root Exudate)	5.79	1.35	NA	NA	2.20	280-410	1.10
Wan and Chen 2022c	Pistia stratiotes 400 nm	C18+PPL Extract (Root Exudate)	5.79	1.35	NA	NA	2.20	280-410	0.79
Wan and Chen 2022c	Eichhornia crassipes 265 nm	C18+PPL Extract (Root Exudate)	4.94	1.67	NA	NA	1.75	280-410	6.47
Wan and Chen 2022c	Eichhornia crassipes 275 nm	C18+PPL Extract (Root Exudate)	4.94	1.67	NA	NA	1.75	280-410	7.74
Wan and Chen 2022c	Eichhornia crassipes 285 nm	C18+PPL Extract (Root Exudate)	4.94	1.67	NA	NA	1.75	280-410	5.90
Wan and Chen 2022c	Eichhornia crassipes 295 nm	C18+PPL Extract (Root Exudate)	4.94	1.67	NA	NA	1.75	280-410	3.97
Wan and Chen 2022c	Eichhornia crassipes 305 nm	C18+PPL Extract (Root Exudate)	4.94	1.67	NA	NA	1.75	280-410	2.99
Wan and Chen 2022c	Eichhornia crassipes 335 nm	C18+PPL Extract (Root Exudate)	4.94	1.67	NA	NA	1.75	280-410	2.33
Wan and Chen 2022c	Eichhornia crassipes 365 nm	C18+PPL Extract (Root Exudate)	4.94	1.67	NA	NA	1.75	280-410	1.45
Wan and Chen 2022c	Eichhornia crassipes 390 nm	C18+PPL Extract (Root Exudate)	4.94	1.67	NA	NA	1.75	280-410	1.26
Wan and Chen 2022c	Eichhornia crassipes 400 nm	C18+PPL Extract (Root Exudate)	4.94	1.67	NA	NA	1.75	280-410	0.93
Wan and Chen 2022c	Nymphaea tetragona 265 nm	C18+PPL Extract (Root Exudate)	3.45	9.13	NA	NA	1.96	280-410	4.83
Wan and Chen 2022c	Nymphaea tetragona 275 nm	C18+PPL Extract (Root Exudate)	3.45	9.13	NA	NA	1.96	280-410	5.57
Wan and Chen 2022c	Nymphaea tetragona 285 nm	C18+PPL Extract (Root Exudate)	3.45	9.13	NA	NA	1.96	280-410	4.33
Wan and Chen 2022c	Nymphaea tetragona 295 nm	C18+PPL Extract (Root Exudate)	3.45	9.13	NA	NA	1.96	280-410	3.62
Wan and Chen 2022c	Nymphaea tetragona 305 nm	C18+PPL Extract (Root Exudate)	3.45	9.13	NA	NA	1.96	280-410	1.42
Wan and Chen 2022c	Nymphaea tetragona 335 nm	C18+PPL Extract (Root Exudate)	3.45	9.13	NA	NA	1.96	280-410	1.26
Wan and Chen 2022c	Nymphaea tetragona 365 nm	C18+PPL Extract (Root Exudate)	3.45	9.13	NA	NA	1.96	280-410	0.90
Wan and Chen 2022c	Nymphaea tetragona 390 nm	C18+PPL Extract (Root Exudate)	3.45	9.13	NA	NA	1.96	280-410	0.72
Wan and Chen 2022c	Nymphaea tetragona 400 nm	C18+PPL Extract (Root Exudate)	3.45	9.13	NA	NA	1.96	280-410	0.45
Wasswa and Zeng 2022 [196]	W16L Soil Oa Photo 0 h	WEOM (Soil)	4.16	4.68	13.90	NA	1.38	290-550	1.55
Wasswa and Zeng 2022	W16L Soil Oa Photo 1 h	WEOM (Soil, photo)	4.21	4.54	14.60	NA	1.36	290-550	1.10
Wasswa and Zeng 2022	W16L Soil Oa Photo 2 h	WEOM (Soil, photo)	4.29	4.37	14.78	NA	1.34	290-550	1.03
Wasswa and Zeng 2022	W16L Soil Oa Photo 4 h	WEOM (Soil, photo)	4.38	4.23	14.81	NA	1.34	290-550	0.91
Wasswa and Zeng 2022	W16L Soil Oa Photo 8 h	WEOM (Soil, photo)	4.44	3.89	14.94	NA	1.32	290-550	0.81
Wasswa and Zeng 2022	W16L Soil Oa Photo 16 h	WEOM (Soil, photo)	4.75	3.83	15.36	NA	1.31	290-550	0.75
Wasswa and Zeng 2022	W16L Soil Oa Photo 32 h	WEOM (Soil, photo)	5.04	3.74	15.59	NA	1.31	290-550	0.69
Wasswa and Zeng 2022	W16L Soil Oa Photo 64 h	WEOM (Soil, photo)	5.49	3.63	15.63	NA	1.29	290-550	0.64
Wasswa and Zeng 2022	W16L Soil Oa Photo 96 h	WEOM (Soil, photo)	5.64	3.24	16.10	NA	1.26	290-550	0.58
Wasswa and Zeng 2022	W24R Soil Oa Photo 0 h	WEOM (Soil)	4.41	4.47	14.27	NA	1.41	290-550	1.59
Wasswa and Zeng 2022	W24R Soil Oa Photo 1 h	WEOM (Soil, photo)	4.48	4.33	14.98	NA	1.40	290-550	1.23
Wasswa and Zeng 2022	W24R Soil Oa Photo 2 h	WEOM (Soil, photo)	4.51	4.18	15.28	NA	1.40	290-550	1.14
Wasswa and Zeng 2022	W24R Soil Oa Photo 4 h	WEOM (Soil, photo)	4.58	4.07	15.63	NA	1.38	290-550	1.01
Wasswa and Zeng 2022	W24R Soil Oa Photo 8 h	WEOM (Soil, photo)	4.60	4.04	15.79	NA	1.37	290-550	0.91
Wasswa and Zeng 2022	W24R Soil Oa Photo 16 h	WEOM (Soil, photo)	5.23	3.82	15.97	NA	1.35	290-550	0.81
Wasswa and Zeng 2022	W24R Soil Oa Photo 32 h	WEOM (Soil, photo)	5.41	3.66	16.08	NA	1.33	290-550	0.76
Wasswa and Zeng 2022	W24R Soil Oa Photo 64 h	WEOM (Soil, photo)	6.10	3.29	16.25	NA	1.31	290-550	0.70
Wasswa and Zeng 2022	W24R Soil Oa Photo 96 h	WEOM (Soil, photo)	6.18	3.01	16.60	NA	1.30	290-550	0.64
Wasswa and Zeng 2022	Leaf Photo 0 h	WEOM (Leaf)	4.25	3.43	15.45	NA	1.53	290-550	1.42
Wasswa and Zeng 2022	Leaf Photo 1 h	WEOM (Leaf, photo)	4.44	3.15	15.74	NA	1.50	290-550	1.08
Wasswa and Zeng 2022	Leaf Photo 2 h	WEOM (Leaf, photo)	4.54	3.07	16.57	NA	1.47	290-550	0.93
Wasswa and Zeng 2022	Leaf Photo 4 h	WEOM (Leaf, photo)	4.63	3.02	16.77	NA	1.43	290-550	0.87
Wasswa and Zeng 2022	Leaf Photo 8 h	WEOM (Leaf, photo)	4.80	2.98	17.12	NA	1.38	290-550	0.77

**Table S18.** Summary of $\Phi_{app,\ ^1O_2}$ and optical data (continued)									
Reference	Sample ID	Sample Classification	*E2:E3*	$SUVA_{254}$ (L mg C^{-1}•m^{-1})	$S_{290–400}$ / $S_{300–400}$ / $S_{290–450}$ / $S_{295–400}$ (μm^{-1})	$S_{300–600}$ / $S_{300–500}$ / $S_{300–700}$ / $S_{275–500}$ (μm^{-1})	FI	Wavelength Range (nm)	$\Phi_{app,\ ^1O_2}$ (×10^{-2} mol mol-photons^{-1})
Wasswa and Zeng 2022	Leaf Photo 16 h	WEOM (Leaf, photo)	5.06	2.76	17.35	NA	1.31	290-550	0.72
Wasswa and Zeng 2022	Leaf Photo 32 h	WEOM (Leaf, photo)	6.09	2.66	18.89	NA	1.28	290-550	0.66
Wasswa and Zeng 2022	Leaf Photo 64 h	WEOM (Leaf, photo)	7.39	2.40	21.08	NA	1.25	290-550	0.61
Wasswa and Zeng 2022	Leaf Photo 96 h	WEOM (Leaf, photo)	8.24	1.97	22.63	NA	1.19	290-550	0.55
Wasswa and Zeng 2022	W16L Soil Oa Bio 0 d	WEOM (Soil)	4.14	4.78	15.43	NA	1.38	290-550	1.56
Wasswa and Zeng 2022	W16L Soil Oa Bio 8 d	WEOM (Soil, bio)	4.11	4.84	15.40	NA	1.40	290-550	2.46
Wasswa and Zeng 2022	W16L Soil Oa Bio 16 d	WEOM (Soil, bio)	4.06	4.96	15.22	NA	1.41	290-550	3.16
Wasswa and Zeng 2022	W16L Soil Oa Bio 32 d	WEOM (Soil, bio)	3.71	5.11	14.34	NA	1.41	290-550	3.33
Wasswa and Zeng 2022	W16L Soil Oa Photo-Bio 0 d	WEOM (Soil, photo)	4.36	4.60	15.75	NA	1.33	290-550	1.03
Wasswa and Zeng 2022	W16L Soil Oa Photo-Bio 8 d	WEOM (Soil, photo-bio)	4.27	4.79	15.29	NA	1.35	290-550	1.34
Wasswa and Zeng 2022	W16L Soil Oa Photo-Bio 16 d	WEOM (Soil, photo-bio)	4.16	4.88	15.22	NA	1.36	290-550	1.60
Wasswa and Zeng 2022	W16L Soil Oa Photo-Bio 32 d	WEOM (Soil, photo-bio)	3.93	5.25	14.53	NA	1.38	290-550	1.85
Wasswa and Zeng 2022	W24R Soil Oa Bio 0 d	WEOM (Soil)	4.49	4.71	16.51	NA	1.41	290-550	1.60
Wasswa and Zeng 2022	W24R Soil Oa Bio 8 d	WEOM (Soil, bio)	4.48	4.75	16.43	NA	1.41	290-550	2.75
Wasswa and Zeng 2022	W24R Soil Oa Bio 16 d	WEOM (Soil, bio)	4.41	4.81	16.17	NA	1.43	290-550	3.35
Wasswa and Zeng 2022	W24R Soil Oa Bio 32 d	WEOM (Soil, bio)	4.00	5.14	15.42	NA	1.45	290-550	3.55
Wasswa and Zeng 2022	W24R Soil Oa Photo-Bio 0 d	WEOM (Soil, photo)	4.56	4.80	16.15	NA	1.40	290-550	1.14
Wasswa and Zeng 2022	W24R Soil Oa Photo-Bio 8 d	WEOM (Soil, photo-bio)	4.51	4.83	16.07	NA	1.40	290-550	1.49
Wasswa and Zeng 2022	W24R Soil Oa Photo-Bio 16 d	WEOM (Soil, photo-bio)	4.48	4.97	15.96	NA	1.41	290-550	1.67
Wasswa and Zeng 2022	W24R Soil Oa Photo-Bio 32 d	WEOM (Soil, photo-bio)	4.08	5.28	14.78	NA	1.43	290-550	2.02
Wasswa and Zeng 2022	Leaf Bio 0 d	WEOM (Leaf)	4.23	3.57	15.81	NA	1.53	290-550	1.42
Wasswa and Zeng 2022	Leaf Bio 8 d	WEOM (Leaf, bio)	4.19	3.75	15.61	NA	1.61	290-550	2.35
Wasswa and Zeng 2022	Leaf Bio 16 d	WEOM (Leaf, bio)	4.15	4.02	15.47	NA	1.65	290-550	2.78
Wasswa and Zeng 2022	Leaf Bio 32 d	WEOM (Leaf, bio)	3.99	4.58	15.04	NA	1.67	290-550	3.08
Wasswa and Zeng 2022	Leaf Photo-Bio 0 d	WEOM (Leaf, photo)	4.57	3.39	15.71	NA	1.46	290-550	0.94
Wasswa and Zeng 2022	Leaf Photo-Bio 8 d	WEOM (Leaf, photo-bio)	4.49	3.74	15.64	NA	1.57	290-550	1.34
Wasswa and Zeng 2022	Leaf Photo-Bio 16 d	WEOM (Leaf, photo-bio)	4.44	4.02	15.53	NA	1.58	290-550	1.77
Wasswa and Zeng 2022	Leaf Photo-Bio 32 d	WEOM (Leaf, photo-bio)	4.41	4.39	15.30	NA	1.61	290-550	1.97
Wasswa and Zeng 2022	W16L Soil Oa Low	WEOM (Soil)	4.15	4.74	14.65	NA	1.37	290-550	1.56
Wasswa and Zeng 2022	W16L Soil Bs Low	WEOM (Soil)	2.85	6.39	14.44	NA	1.64	290-550	5.94
Wasswa and Zeng 2022	W16L Soil Oa Mid	WEOM (Soil)	4.13	4.69	14.87	NA	1.37	290-550	1.57
Wasswa and Zeng 2022	W16L Soil Bs Mid	WEOM (Soil)	2.83	6.35	14.47	NA	1.65	290-550	5.76
Wasswa and Zeng 2022	W16L Soil Oa High	WEOM (Soil)	4.19	4.82	15.04	NA	1.38	290-550	1.56
Wasswa and Zeng 2022	W16L Soil Bs High	WEOM (Soil)	2.86	6.37	14.69	NA	1.67	290-550	6.09
Wasswa and Zeng 2022	W24R Soil Oa Low	WEOM (Soil)	4.46	4.59	14.55	NA	1.41	290-550	1.59
Wasswa and Zeng 2022	W24R Soil Bs Low	WEOM (Soil)	2.88	6.45	13.91	NA	1.64	290-550	6.02
Wasswa and Zeng 2022	W24R Soil Oa Mid	WEOM (Soil)	4.45	4.52	14.78	NA	1.42	290-550	1.59
Wasswa and Zeng 2022	W24R Soil Bs Mid	WEOM (Soil)	2.82	6.15	13.95	NA	1.68	290-550	5.93
Wasswa and Zeng 2022	W24R Soil Oa High	WEOM (Soil)	4.46	4.43	14.81	NA	1.42	290-550	1.60
Wasswa and Zeng 2022	W24R Soil Bs High	WEOM (Soil)	2.75	6.11	14.06	NA	1.70	290-550	5.82
Wasswa and Zeng 2022	Stream ST16	Whole Water (Stream)	5.28	3.41	17.20	NA	1.65	290-550	2.32
Wasswa and Zeng 2022	Stream ST24	Whole Water (Stream)	6.00	3.90	16.30	NA	1.63	290-550	2.38
Wasswa and Zeng 2022	Honnedaga Lake	Whole Water (Lake)	6.55	1.22	18.90	NA	1.59	290-550	1.92
Wasswa and Zeng 2022	Suwannee River Fulvic Acid	IHSS Isolate (SRFA)	NA	NA	NA	NA	NA	290-550	2.51
Wasswa and Zeng 2022	Suwannee River Humic Acid	IHSS Isolate (SRHA)	NA	NA	NA	NA	NA	290-550	1.47
Wasswa and Zeng 2022	Suwannee River NOM	IHSS Isolate (SRNOM)	NA	NA	NA	NA	NA	290-550	2.39
Wasswa and Zeng 2022	Pahokee Peat Fulvic Acid	IHSS Isolate (PPFA)	NA	NA	NA	NA	NA	290-550	2.57
Wasswa and Zeng 2022	Pahokee Peat Humic Acid	IHSS Isolate (PPHA)	NA	NA	NA	NA	NA	290-550	1.57
Wasswa and Zeng 2022	Nordic Reservoir NOM	IHSS Isolate (NRNOM)	NA	NA	NA	NA	NA	290-550	2.78
Wasswa and Zeng 2022	Upper Mississippi River NOM	IHSS Isolate (UMRNOM)	NA	NA	NA	NA	NA	290-550	2.75
Wasswa and Zeng 2022	Glucose Bio 0 d Batch 1	WEOM (Glucose)	3.02	0.28	5.93	NA	1.32	290-550	0.25

Table S18. Summary of $\Phi_{app, {}^1O_2}$ and optical data (continued)

Reference	Sample ID	Sample Classification	*E2:E3*	$SUVA_{254}$ (L mg C^{-1}•m^{-1})	$S_{290–400}$ / $S_{300–400}$ / $S_{290–450}$ / $S_{295–400}$ (μm^{-1})	$S_{300–600}$ / $S_{300–500}$ / $S_{300–700}$ / $S_{275–500}$ (μm^{-1})	FI	Wavelength Range (nm)	$\Phi_{app, {}^1O_2}$ ($\times 10^{-2}$ mol mol-photons^{-1})
Wasswa and Zeng 2022	Glucose Bio 8 d Batch 1	WEOM (Glucose, bio)	8.36	2.67	26.51	NA	1.43	290-550	3.60
Wasswa and Zeng 2022	Glucose Bio 16 d Batch 1	WEOM (Glucose, bio)	18.39	2.98	27.21	NA	1.53	290-550	4.65
Wasswa and Zeng 2022	Glucose Bio 32 d Batch 1	WEOM (Glucose, bio)	25.83	3.26	30.06	NA	1.78	290-550	5.69
Wasswa and Zeng 2022	ESHA Photo 0 h	IHSS Isolate (ESHA)	2.62	2.66	9.45	NA	0.89	290-550	1.41
Wasswa and Zeng 2022	ESHA Photo 4 h	IHSS Isolate (ESHA, photo)	2.68	2.61	9.66	NA	0.79	290-550	1.31
Wasswa and Zeng 2022	ESHA Photo 16 h	IHSS Isolate (ESHA, photo)	2.89	2.44	10.21	NA	0.79	290-550	1.20
Wasswa and Zeng 2022	ESHA Photo 64 h	IHSS Isolate (ESHA, photo)	3.26	2.35	10.93	NA	0.76	290-550	1.08
Wasswa and Zeng 2022	SRFA Photo 0 h	IHSS Isolate (SRFA)	4.91	8.32	16.79	NA	1.33	290-550	2.66
Wasswa and Zeng 2022	SRFA Photo 4 h	IHSS Isolate (SRFA, photo)	5.06	7.11	16.86	NA	1.31	290-550	2.63
Wasswa and Zeng 2022	SRFA Photo 16 h	IHSS Isolate (SRFA, photo)	5.34	5.88	17.23	NA	1.28	290-550	2.56
Wasswa and Zeng 2022	SRFA Photo 64 h	IHSS Isolate (SRFA, photo)	6.13	4.88	18.08	NA	1.25	290-550	2.42
Wasswa and Zeng 2022	Glucose Bio 0 d Batch 2	WEOM (Glucose)	3.31	0.06	8.40	NA	1.09	290-550	0.26
Wasswa and Zeng 2022	Glucose Bio 8 d Batch 2	WEOM (Glucose, bio)	8.59	2.36	21.60	NA	1.41	290-550	4.08
Wasswa and Zeng 2022	Glucose Bio 16 d Batch 2	WEOM (Glucose, bio)	16.54	3.22	26.25	NA	2.16	290-550	4.94
Wasswa and Zeng 2022	Glucose Bio 32 d Batch 2	WEOM (Glucose, bio)	25.96	4.27	30.30	NA	2.64	290-550	6.13
Wasswa and Zeng 2022	ESHA Bio 0 d	IHSS Isolate (ESHA)	2.46	8.13	8.86	NA	0.68	290-550	1.42
Wasswa and Zeng 2022	ESHA Bio 8 d	IHSS Isolate (ESHA, bio)	2.45	8.00	8.78	NA	0.70	290-550	1.42
Wasswa and Zeng 2022	ESHA Bio 16 d	IHSS Isolate (ESHA, bio)	2.44	8.09	8.76	NA	0.72	290-550	1.42
Wasswa and Zeng 2022	ESHA Bio 32 d	IHSS Isolate (ESHA, bio)	2.24	8.26	8.05	NA	0.73	290-550	1.43
Xu 2022 [197]	ADOM_Bulk	WEOM (Algae Lake)	3.30	1.33	NA	NA	NA	290-600	2.21
Xu 2022	ADOM_HMW	WEOM+UF Fraction (Algae Lake)	3.03	1.31	NA	NA	NA	290-600	1.52
Xu 2022	ADOM_LMW	WEOM+UF Fraction (Algae Lake)	3.86	0.98	NA	NA	NA	290-600	3.18
Xu 2022	MDOM_Bulk	WEOM (Microphyte Lake)	2.83	5.16	NA	NA	NA	290-600	0.74
Xu 2022	MDOM_HMW	WEOM+UF Fraction (Microphyte Lake)	2.72	5.54	NA	NA	NA	290-600	0.19
Xu 2022	MDOM_LMW	WEOM+UF Fraction (Microphyte Lake)	2.23	1.38	NA	NA	NA	290-600	1.37
Xu 2022	SRHA_Bulk	IHSS Isolate (SRHA)	3.14	4.85	NA	NA	NA	290-600	1.28
Xu 2022	SRHA_HMW	UF Fraction (SRHA)	3.42	6.38	NA	NA	NA	290-600	1.13
Xu 2022	SRHA_LMW	UF Fraction (SRHA)	3.86	2.28	NA	NA	NA	290-600	3.02
Zeng and Wang 2022 [198]	CK_1d	Whole Water (Paddy)	4.13	NA	NA	NA	NA	290-400	1.43
Zeng and Wang 2022	Biochar_1d	Whole Water (Paddy)	4.85	NA	NA	NA	NA	290-400	2.31
Zeng and Wang 2022	Straw_1d	Whole Water (Paddy)	2.30	NA	NA	NA	NA	290-400	1.48
Zeng and Wang 2022	Lime_1d	Whole Water (Paddy)	3.47	NA	NA	NA	NA	290-400	1.80
Zeng and Wang 2022	CK_10d	Whole Water (Paddy)	NA	NA	NA	NA	NA	290-400	1.57
Zeng and Wang 2022	Biochar_10d	Whole Water (Paddy)	NA	NA	NA	NA	NA	290-400	0.93
Zeng and Wang 2022	Straw_10d	Whole Water (Paddy)	NA	NA	NA	NA	NA	290-400	1.83
Zeng and Wang 2022	Lime_10d	Whole Water (Paddy)	NA	NA	NA	NA	NA	290-400	2.36
Zeng and Wang 2022	CK_20d	Whole Water (Paddy)	NA	NA	NA	NA	NA	290-400	1.58
Zeng and Wang 2022	Biochar_20d	Whole Water (Paddy)	NA	NA	NA	NA	NA	290-400	2.47
Zeng and Wang 2022	Straw_20d	Whole Water (Paddy)	NA	NA	NA	NA	NA	290-400	2.86
Zeng and Wang 2022	Lime_20d	Whole Water (Paddy)	NA	NA	NA	NA	NA	290-400	5.72
Zeng and Wang 2022	CK_30d	Whole Water (Paddy)	2.68	NA	NA	NA	NA	290-400	1.43
Zeng and Wang 2022	Biochar_30d	Whole Water (Paddy)	2.72	NA	NA	NA	NA	290-400	1.03
Zeng and Wang 2022	Straw_30d	Whole Water (Paddy)	3.71	NA	NA	NA	NA	290-400	1.46
Zeng and Wang 2022	Lime_30d	Whole Water (Paddy)	3.13	NA	NA	NA	NA	290-400	3.30
Zeng and Wang 2022	CK_45d	Whole Water (Paddy)	NA	NA	NA	NA	NA	290-400	1.53
Zeng and Wang 2022	Biochar_45d	Whole Water (Paddy)	NA	NA	NA	NA	NA	290-400	2.20
Zeng and Wang 2022	Straw_45d	Whole Water (Paddy)	NA	NA	NA	NA	NA	290-400	3.47
Zeng and Wang 2022	Lime_45d	Whole Water (Paddy)	NA	NA	NA	NA	NA	290-400	7.28
Zeng and Wang 2022	CK_60d	Whole Water (Paddy)	3.08	NA	NA	NA	NA	290-400	1.31
Zeng and Wang 2022	Biochar_60d	Whole Water (Paddy)	3.07	NA	NA	NA	NA	290-400	2.03
Zeng and Wang 2022	Straw_60d	Whole Water (Paddy)	6.19	NA	NA	NA	NA	290-400	1.73

**Table S18.** Summary of $\Phi_{app, {}^1O_2}$ and optical data (continued)									
Reference	Sample ID	Sample Classification	*E2:E3*	SUVA$_{254}$ (L mg C^{-1}•m^{-1})	$S_{290–400}$ / $S_{300–400}$ / $S_{290–450}$ / $S_{295–400}$ (μm^{-1})	$S_{300–600}$ / $S_{300–500}$ / $S_{300–700}$ / $S_{275–500}$ (μm^{-1})	FI	Wavelength Range (nm)	$\Phi_{app, {}^1O_2}$ (×10^{-2} mol mol-photons^{-1})
Zeng and Wang 2022	Lime_60d	Whole Water (Paddy)	4.65	NA	NA	NA	NA	290-400	2.50
Allen and McKay 2023 [199]	SRFA pH 4	IHSS Isolate (SRFA)	NA	NA	NA	NA	NA	350-400	0.96
Allen and McKay 2023	SRFA pH 5	IHSS Isolate (SRFA)	NA	NA	NA	NA	NA	350-400	0.88
Allen and McKay 2023	SRFA pH 6	IHSS Isolate (SRFA)	NA	NA	NA	NA	NA	350-400	0.83
Allen and McKay 2023	SRFA pH 7	IHSS Isolate (SRFA)	NA	NA	NA	NA	NA	350-400	0.81
Allen and McKay 2023	SRFA pH 8	IHSS Isolate (SRFA)	NA	NA	NA	NA	NA	350-400	0.79
Allen and McKay 2023	SRFA pH 9	IHSS Isolate (SRFA)	NA	NA	NA	NA	NA	350-400	0.61
Allen and McKay 2023	SRNOM pH 4	IHSS Isolate (SRNOM)	NA	NA	NA	NA	NA	350-400	1.06
Allen and McKay 2023	SRNOM pH 5	IHSS Isolate (SRNOM)	NA	NA	NA	NA	NA	350-400	0.99
Allen and McKay 2023	SRNOM pH 6	IHSS Isolate (SRNOM)	NA	NA	NA	NA	NA	350-400	0.99
Allen and McKay 2023	SRNOM pH 7	IHSS Isolate (SRNOM)	NA	NA	NA	NA	NA	350-400	0.95
Allen and McKay 2023	SRNOM pH 8	IHSS Isolate (SRNOM)	NA	NA	NA	NA	NA	350-400	0.78
Allen and McKay 2023	SRNOM pH 9	IHSS Isolate (SRNOM)	NA	NA	NA	NA	NA	350-400	0.74
Allen and McKay 2023	MRNOM pH 4	IHSS Isolate (UMRNOM)	NA	NA	NA	NA	NA	350-400	1.42
Allen and McKay 2023	MRNOM pH 5	IHSS Isolate (UMRNOM)	NA	NA	NA	NA	NA	350-400	1.29
Allen and McKay 2023	MRNOM pH 6	IHSS Isolate (UMRNOM)	NA	NA	NA	NA	NA	350-400	1.15
Allen and McKay 2023	MRNOM pH 7	IHSS Isolate (UMRNOM)	NA	NA	NA	NA	NA	350-400	1.24
Allen and McKay 2023	MRNOM pH 8	IHSS Isolate (UMRNOM)	NA	NA	NA	NA	NA	350-400	1.26
Allen and McKay 2023	MRNOM pH 9	IHSS Isolate (UMRNOM)	NA	NA	NA	NA	NA	350-400	1.11
Allen and McKay 2023	PPFA pH 4	IHSS Isolate (PPFA)	NA	NA	NA	NA	NA	350-400	3.30
Allen and McKay 2023	PPFA pH 5	IHSS Isolate (PPFA)	NA	NA	NA	NA	NA	350-400	2.92
Allen and McKay 2023	PPFA pH 6	IHSS Isolate (PPFA)	NA	NA	NA	NA	NA	350-400	3.17
Allen and McKay 2023	PPFA pH 7	IHSS Isolate (PPFA)	NA	NA	NA	NA	NA	350-400	3.01
Allen and McKay 2023	PPFA pH 8	IHSS Isolate (PPFA)	NA	NA	NA	NA	NA	350-400	2.74
Allen and McKay 2023	PPFA pH 9	IHSS Isolate (PPFA)	NA	NA	NA	NA	NA	350-400	2.95
Allen and McKay 2023	PPHA pH 4	IHSS Isolate (PPHA)	NA	NA	NA	NA	NA	350-400	0.70
Allen and McKay 2023	PPHA pH 5	IHSS Isolate (PPHA)	NA	NA	NA	NA	NA	350-400	0.90
Allen and McKay 2023	PPHA pH 6	IHSS Isolate (PPHA)	NA	NA	NA	NA	NA	350-400	0.81
Allen and McKay 2023	PPHA pH 7	IHSS Isolate (PPHA)	NA	NA	NA	NA	NA	350-400	0.91
Allen and McKay 2023	PPHA pH 8	IHSS Isolate (PPHA)	NA	NA	NA	NA	NA	350-400	0.89
Allen and McKay 2023	PPHA pH 9	IHSS Isolate (PPHA)	NA	NA	NA	NA	NA	350-400	0.86
Allen and McKay 2023	ESHA pH 4	IHSS Isolate (ESHA)	NA	NA	NA	NA	NA	350-400	0.94
Allen and McKay 2023	ESHA pH 5	IHSS Isolate (ESHA)	NA	NA	NA	NA	NA	350-400	1.41
Allen and McKay 2023	ESHA pH 6	IHSS Isolate (ESHA)	NA	NA	NA	NA	NA	350-400	1.62
Allen and McKay 2023	ESHA pH 7	IHSS Isolate (ESHA)	NA	NA	NA	NA	NA	350-400	1.58
Allen and McKay 2023	ESHA pH 8	IHSS Isolate (ESHA)	NA	NA	NA	NA	NA	350-400	1.56
Allen and McKay 2023	ESHA pH 9	IHSS Isolate (ESHA)	NA	NA	NA	NA	NA	350-400	1.70
Berg and Remucal 2023 [200]	Crystal Bog	Whole Water (Lake)	5.78	2.16	NA	NA	1.43	315-415	1.40
Berg and Remucal 2023	Trout Bog	Whole Water (Lake)	5.79	2.89	NA	NA	1.43	315-415	1.10
Berg and Remucal 2023	Allequash Lake	Whole Water (Lake)	7.56	1.92	NA	NA	1.50	315-415	2.10
Berg and Remucal 2023	Big Muskellunge Lake	Whole Water (Lake)	8.75	0.47	NA	NA	1.53	315-415	3.30
Berg and Remucal 2023	Crystal Lake	Whole Water (Lake)	8.58	0.25	NA	NA	1.54	315-415	2.50
Berg and Remucal 2023	Sparkling Lake	Whole Water (Lake)	9.67	1.08	NA	NA	1.60	315-415	2.50
Berg and Remucal 2023	Trout Lake	Whole Water (Lake)	9.70	0.81	NA	NA	1.51	315-415	2.80
Berg and Remucal 2023	Memories	Whole Water (River)	9.40	2.50	NA	NA	1.65	315-415	7.30
Berg and Remucal 2023	Olsen Ditch	Whole Water (Agricultural Ditch)	10.53	3.90	NA	NA	1.69	315-415	4.57
Berg and Remucal 2023	Wammer Ditch	Whole Water (Agricultural Ditch)	8.88	2.31	NA	NA	1.64	315-415	4.04
Berg and Remucal 2023	Seven-Mile Creek	Whole Water (River)	7.50	3.01	NA	NA	1.70	315-415	4.32
Berg and Remucal 2023	WRRF Pre-Cl	Whole Water (WWTP Effluent)	8.16	1.62	NA	NA	2.04	315-415	3.00
Berg and Remucal 2023	WRRF Reuse	Whole Water (WWTP Effluent, Cl2)	11.78	0.96	NA	NA	1.92	315-415	7.70
Berg and Remucal 2023	WRRF Post-Cl	Whole Water (WWTP Effluent, Cl2)	10.17	1.29	NA	NA	1.97	315-415	5.80

**Table S18.** Summary of $\Phi_{app,\ ^1O_2}$ and optical data (continued)									
Reference	Sample ID	Sample Classification	*E2:E3*	$SUVA_{254}$ (L mg C^{-1}•m^{-1})	$S_{290–400}$ / $S_{300–400}$ / $S_{290–450}$ / $S_{295–400}$ (μm^{-1})	$S_{300–600}$ / $S_{300–500}$ / $S_{300–700}$ / $S_{275–500}$ (μm^{-1})	FI	Wavelength Range (nm)	$\Phi_{app,\ ^1O_2}$ ($\times 10^{-2}$ mol mol-photons^{-1})
Berg and Remucal 2023	Kiwannis	Whole Water (WW-Impacted River)	7.81	2.51	NA	NA	1.61	315-415	4.40
Berg and Remucal 2023	North Yahara	Whole Water (River)	7.40	2.89	NA	NA	1.60	315-415	3.08
Berg and Remucal 2023	Lake Mendota	Whole Water (Lake)	10.44	1.57	NA	NA	1.68	315-415	2.80
Berg and Remucal 2023	Lake Wingra	Whole Water (Lake)	8.22	2.03	NA	NA	1.61	315-415	3.20
Berg and Remucal 2023	Lake Kegonsa	Whole Water (Lake)	5.67	1.22	NA	NA	1.61	315-415	5.25
Berg and Remucal 2023	South Yahara	Whole Water (River)	7.09	1.34	NA	NA	1.64	315-415	2.80
Berg and Remucal 2023	Badfish Upstream	Whole Water (River)	7.31	3.40	NA	NA	1.72	315-415	3.80
Berg and Remucal 2023	Nine Springs PreUV	Whole Water (WWTP Effluent)	5.86	2.04	NA	NA	2.04	315-415	3.42
Berg and Remucal 2023	Nine Springs PostUV	Whole Water (WWTP Effluent, UV)	5.74	1.66	NA	NA	2.03	315-415	3.90
Berg and Remucal 2023	Badfish Downstream	Whole Water (WW-Impacted River)	5.79	2.28	NA	NA	2.00	315-415	2.76
Berg and Remucal 2023	Confluence	Whole Water (WW-Impacted River)	6.40	2.34	NA	NA	1.70	315-415	2.40
Berg and Remucal 2023	Sand Creek	Whole Water (River)	5.35	3.31	NA	NA	1.50	315-415	6.00
Berg and Remucal 2023	Meadowlands	Whole Water (River)	5.43	3.18	NA	NA	1.52	315-415	1.10
Berg and Remucal 2023	River Inn	Whole Water (River)	6.54	3.18	NA	NA	1.52	315-415	1.37
Berg and Remucal 2023	Munger Landing	Whole Water (River)	6.05	2.88	NA	NA	1.50	315-415	1.70
Berg and Remucal 2023	East Detroit	Whole Water (River)	5.94	2.97	NA	NA	1.51	315-415	1.55
Berg and Remucal 2023	WLSSD Pre-Cl; Mill Shutdown	Whole Water (WWTP Effluent)	5.98	2.18	NA	NA	1.74	315-415	1.07
Berg and Remucal 2023	WLSSD Post-Cl; Mill Shutdown	Whole Water (WWTP Effluent, Cl2)	6.72	2.06	NA	NA	1.72	315-415	1.56
Berg and Remucal 2023	WLSSD Pre-Cl	Whole Water (WWTP Effluent)	6.93	1.86	NA	NA	1.56	315-415	2.04
Berg and Remucal 2023	WLSSD Post-Cl	Whole Water (WWTP Effluent, Cl2)	7.06	2.34	NA	NA	1.54	315-415	1.96
Berg and Remucal 2023	Blatnik Bridge	Whole Water (River)	6.34	2.12	NA	NA	1.49	315-415	1.80
Berg and Remucal 2023	Wisconsin Point	Whole Water (Lake)	6.38	0.20	NA	NA	1.46	315-415	2.25
Berg and Remucal 2023	River Front Park	Whole Water (River)	7.53	0.82	NA	NA	1.53	315-415	4.70
Berg and Remucal 2023	East River Parkway	Whole Water (River)	13.03	4.72	NA	NA	1.55	315-415	3.20
Berg and Remucal 2023	Minnesota River	Whole Water (River)	6.99	2.24	NA	NA	1.60	315-415	5.90
Berg and Remucal 2023	Metro WWTP Pre-Cl	Whole Water (WWTP Effluent)	5.80	1.51	NA	NA	1.98	315-415	8.27
Berg and Remucal 2023	Metro WWTP Post-Cl	Whole Water (WWTP Effluent, Cl2)	5.65	1.46	NA	NA	1.98	315-415	7.00
Berg and Remucal 2023	Metro Downstream	Whole Water (WW-Impacted River)	7.15	2.33	NA	NA	1.56	315-415	3.70
Berg and Remucal 2023	Eagles Point Pre-UV	Whole Water (WWTP Effluent)	4.93	1.42	NA	NA	2.08	315-415	2.60
Berg and Remucal 2023	Eagles Point Post-UV	Whole Water (WWTP Effluent, UV)	6.93	2.12	NA	NA	2.09	315-415	2.79
Berg and Remucal 2023	Eagles Point Downstream	Whole Water (WW-Impacted River)	7.10	2.14	NA	NA	1.57	315-415	3.74
Berg and Remucal 2023	Lake of the Isles	Whole Water (Lake)	9.75	0.97	NA	NA	1.64	315-415	5.80
Berg and Remucal 2023	Vadnais Lake	Whole Water (Lake)	10.28	1.64	NA	NA	1.58	315-415	4.65
Berg and Remucal 2023	Lake Phalen	Whole Water (Lake)	10.97	1.30	NA	NA	1.60	315-415	7.00
Buckley and Rosario-Ortiz 2023 [201]	PLFA 0 mmol O3 / mmol C Fraction 1	IHSS Isolate (PLFA, SEC)	3.03	NA	NA	NA	NA	290-400	0.88
Buckley and Rosario-Ortiz 2023	PLFA 0 mmol O3 / mmol C Fraction 2	IHSS Isolate (PLFA, SEC)	1.90	NA	NA	NA	NA	290-400	1.61
Buckley and Rosario-Ortiz 2023	PLFA 0 mmol O3 / mmol C Fraction 3	IHSS Isolate (PLFA, SEC)	1.09	NA	NA	NA	NA	290-400	2.01
Buckley and Rosario-Ortiz 2023	PLFA 0 mmol O3 / mmol C Reconstituted	IHSS Isolate (PLFA)	2.04	NA	NA	NA	NA	290-400	1.50
Buckley and Rosario-Ortiz 2023	PLFA 0.05 mmol O3 / mmol C Fraction 1	IHSS Isolate (PLFA, SEC, O3)	2.98	NA	NA	NA	NA	290-400	2.73
Buckley and Rosario-Ortiz 2023	PLFA 0.05 mmol O3 / mmol C Fraction 2	IHSS Isolate (PLFA, SEC, O3)	1.68	NA	NA	NA	NA	290-400	2.18
Buckley and Rosario-Ortiz 2023	PLFA 0.05 mmol O3 / mmol C Fraction 3	IHSS Isolate (PLFA, SEC, O3)	0.98	NA	NA	NA	NA	290-400	5.06
Buckley and Rosario-Ortiz 2023	PLFA 0.05 mmol O3 / mmol C Reconstituted	IHSS Isolate (PLFA, O3)	1.87	NA	NA	NA	NA	290-400	2.87
Buckley and Rosario-Ortiz 2023	PLFA 0.05 t-BuOH mmol O3 / mmol C Fraction 1	IHSS Isolate (PLFA, SEC, O3, tBuOH)	2.15	NA	NA	NA	NA	290-400	1.42
Buckley and Rosario-Ortiz 2023	PLFA 0.05 t-BuOH mmol O3 / mmol C Fraction 2	IHSS Isolate (PLFA, SEC, O3, tBuOH)	1.57	NA	NA	NA	NA	290-400	2.28
Buckley and Rosario-Ortiz 2023	PLFA 0.05 t-BuOH mmol O3 / mmol C Fraction 3	IHSS Isolate (PLFA, SEC, O3, tBuOH)	0.80	NA	NA	NA	NA	290-400	4.42
Buckley and Rosario-Ortiz 2023	PLFA 0.05 t-BuOH mmol O3 / mmol C Reconstituted	IHSS Isolate (PLFA, O3, tBuOH)	1.56	NA	NA	NA	NA	290-400	2.50
Buckley and Rosario-Ortiz 2023	PLFA 0.1 mmol O3 / mmol C Fraction 1	IHSS Isolate (PLFA, SEC, O3)	1.98	NA	NA	NA	NA	290-400	2.36
Buckley and Rosario-Ortiz 2023	PLFA 0.1 mmol O3 / mmol C Fraction 2	IHSS Isolate (PLFA, SEC, O3)	1.30	NA	NA	NA	NA	290-400	3.24
Buckley and Rosario-Ortiz 2023	PLFA 0.1 mmol O3 / mmol C Fraction 3	IHSS Isolate (PLFA, SEC, O3)	0.57	NA	NA	NA	NA	290-400	4.95
Buckley and Rosario-Ortiz 2023	PLFA 0.1 mmol O3 / mmol C Reconstituted	IHSS Isolate (PLFA, O3)	1.31	NA	NA	NA	NA	290-400	3.35
Buckley and Rosario-Ortiz 2023	PLFA 0.1 t-BuOH mmol O3 / mmol C Fraction 1	IHSS Isolate (PLFA, SEC, O3, tBuOH)	1.72	NA	NA	NA	NA	290-400	2.89

Table S18. Summary of $\Phi_{app,\ ^1O_2}$ and optical data (continued)

Reference	Sample ID	Sample Classification	*E2:E3*	$SUVA_{254}$ (L mg C^{-1}•m^{-1})	$S_{290–400}$ / $S_{300–400}$ / $S_{290–450}$ / $S_{295–400}$ (µm^{-1})	$S_{300–600}$ / $S_{300–500}$ / $S_{300–700}$ / $S_{275–500}$ (µm^{-1})	FI	Wavelength Range (nm)	$\Phi_{app,\ ^1O_2}$ (×10^{-2} mol mol-photons^{-1})
Buckley and Rosario-Ortiz 2023	PLFA 0.1 t-BuOH mmol O3 / mmol C Fraction 2	IHSS Isolate (PLFA, SEC, O3, tBuOH)	1.19	NA	NA	NA	NA	290-400	4.36
Buckley and Rosario-Ortiz 2023	PLFA 0.1 t-BuOH mmol O3 / mmol C Fraction 3	IHSS Isolate (PLFA, SEC, O3, tBuOH)	0.57	NA	NA	NA	NA	290-400	5.70
Buckley and Rosario-Ortiz 2023	PLFA 0.1 t-BuOH mmol O3 / mmol C Reconstituted	IHSS Isolate (PLFA, O3, tBuOH)	1.18	NA	NA	NA	NA	290-400	4.31
Buckley and Rosario-Ortiz 2023	PLFA 0.2 mmol O3 / mmol C Fraction 1	IHSS Isolate (PLFA, SEC, O3)	2.23	NA	NA	NA	NA	290-400	2.60
Buckley and Rosario-Ortiz 2023	PLFA 0.2 mmol O3 / mmol C Fraction 2	IHSS Isolate (PLFA, SEC, O3)	1.08	NA	NA	NA	NA	290-400	2.96
Buckley and Rosario-Ortiz 2023	PLFA 0.2 mmol O3 / mmol C Fraction 3	IHSS Isolate (PLFA, SEC, O3)	0.42	NA	NA	NA	NA	290-400	7.37
Buckley and Rosario-Ortiz 2023	PLFA 0.2 mmol O3 / mmol C Reconstituted	IHSS Isolate (PLFA, O3)	1.13	NA	NA	NA	NA	290-400	3.84
Buckley and Rosario-Ortiz 2023	PLFA 0.2 t-BuOH mmol O3 / mmol C Fraction 1	IHSS Isolate (PLFA, SEC, O3, tBuOH)	1.80	NA	NA	NA	NA	290-400	3.22
Buckley and Rosario-Ortiz 2023	PLFA 0.2 t-BuOH mmol O3 / mmol C Fraction 2	IHSS Isolate (PLFA, SEC, O3, tBuOH)	1.14	NA	NA	NA	NA	290-400	4.07
Buckley and Rosario-Ortiz 2023	PLFA 0.2 t-BuOH mmol O3 / mmol C Fraction 3	IHSS Isolate (PLFA, SEC, O3, tBuOH)	0.50	NA	NA	NA	NA	290-400	6.72
Buckley and Rosario-Ortiz 2023	PLFA 0.2 t-BuOH mmol O3 / mmol C Reconstituted	IHSS Isolate (PLFA, O3, tBuOH)	1.14	NA	NA	NA	NA	290-400	4.46
Buckley and Rosario-Ortiz 2023	SRFA 0 mmol O3 / mmol C Fraction 1	IHSS Isolate (SRFA, SEC)	7.20	NA	NA	NA	NA	290-400	0.69
Buckley and Rosario-Ortiz 2023	SRFA 0 mmol O3 / mmol C Fraction 2	IHSS Isolate (SRFA, SEC)	2.96	NA	NA	NA	NA	290-400	2.30
Buckley and Rosario-Ortiz 2023	SRFA 0 mmol O3 / mmol C Fraction 3	IHSS Isolate (SRFA, SEC)	1.71	NA	NA	NA	NA	290-400	3.23
Buckley and Rosario-Ortiz 2023	SRFA 0 mmol O3 / mmol C Reconstituted	IHSS Isolate (SRFA)	4.45	NA	NA	NA	NA	290-400	1.36
Buckley and Rosario-Ortiz 2023	SRFA 0.05 mmol O3 / mmol C Fraction 1	IHSS Isolate (SRFA, SEC, O3)	4.49	NA	NA	NA	NA	290-400	0.68
Buckley and Rosario-Ortiz 2023	SRFA 0.05 mmol O3 / mmol C Fraction 2	IHSS Isolate (SRFA, SEC, O3)	2.84	NA	NA	NA	NA	290-400	1.45
Buckley and Rosario-Ortiz 2023	SRFA 0.05 mmol O3 / mmol C Fraction 3	IHSS Isolate (SRFA, SEC, O3)	1.48	NA	NA	NA	NA	290-400	4.04
Buckley and Rosario-Ortiz 2023	SRFA 0.05 mmol O3 / mmol C Reconstituted	IHSS Isolate (SRFA, O3)	3.14	NA	NA	NA	NA	290-400	1.29
Buckley and Rosario-Ortiz 2023	SRFA 0.05 t-BuOH mmol O3 / mmol C Fraction 1	IHSS Isolate (SRFA, SEC, O3, tBuOH)	4.31	NA	NA	NA	NA	290-400	1.12
Buckley and Rosario-Ortiz 2023	SRFA 0.05 t-BuOH mmol O3 / mmol C Fraction 2	IHSS Isolate (SRFA, SEC, O3, tBuOH)	2.77	NA	NA	NA	NA	290-400	2.52
Buckley and Rosario-Ortiz 2023	SRFA 0.05 t-BuOH mmol O3 / mmol C Fraction 3	IHSS Isolate (SRFA, SEC, O3, tBuOH)	1.35	NA	NA	NA	NA	290-400	4.51
Buckley and Rosario-Ortiz 2023	SRFA 0.05 t-BuOH mmol O3 / mmol C Reconstituted	IHSS Isolate (SRFA, O3, tBuOH)	3.02	NA	NA	NA	NA	290-400	2.01
Buckley and Rosario-Ortiz 2023	SRFA 0.1 mmol O3 / mmol C Fraction 1	IHSS Isolate (SRFA, SEC, O3)	6.52	NA	NA	NA	NA	290-400	0.81
Buckley and Rosario-Ortiz 2023	SRFA 0.1 mmol O3 / mmol C Fraction 2	IHSS Isolate (SRFA, SEC, O3)	1.62	NA	NA	NA	NA	290-400	2.08
Buckley and Rosario-Ortiz 2023	SRFA 0.1 mmol O3 / mmol C Fraction 3	IHSS Isolate (SRFA, SEC, O3)	0.57	NA	NA	NA	NA	290-400	6.04
Buckley and Rosario-Ortiz 2023	SRFA 0.1 mmol O3 / mmol C Reconstituted	IHSS Isolate (SRFA, O3)	2.67	NA	NA	NA	NA	290-400	1.74
Buckley and Rosario-Ortiz 2023	SRFA 0.2 mmol O3 / mmol C Fraction 1	IHSS Isolate (SRFA, SEC, O3)	7.90	NA	NA	NA	NA	290-400	2.04
Buckley and Rosario-Ortiz 2023	SRFA 0.2 mmol O3 / mmol C Fraction 2	IHSS Isolate (SRFA, SEC, O3)	1.81	NA	NA	NA	NA	290-400	2.06
Buckley and Rosario-Ortiz 2023	SRFA 0.2 mmol O3 / mmol C Fraction 3	IHSS Isolate (SRFA, SEC, O3)	0.56	NA	NA	NA	NA	290-400	10.86
Buckley and Rosario-Ortiz 2023	SRFA 0.2 mmol O3 / mmol C Reconstituted	IHSS Isolate (SRFA, O3)	2.13	NA	NA	NA	NA	290-400	3.35
Buckley and Rosario-Ortiz 2023	SRFA 0.2 t-BuOH mmol O3 / mmol C Fraction 1	IHSS Isolate (SRFA, SEC, O3, tBuOH)	6.29	NA	NA	NA	NA	290-400	2.89
Buckley and Rosario-Ortiz 2023	SRFA 0.2 t-BuOH mmol O3 / mmol C Fraction 2	IHSS Isolate (SRFA, SEC, O3, tBuOH)	1.82	NA	NA	NA	NA	290-400	3.06
Buckley and Rosario-Ortiz 2023	SRFA 0.2 t-BuOH mmol O3 / mmol C Fraction 3	IHSS Isolate (SRFA, SEC, O3, tBuOH)	0.59	NA	NA	NA	NA	290-400	10.65
Buckley and Rosario-Ortiz 2023	SRFA 0.2 t-BuOH mmol O3 / mmol C Reconstituted	IHSS Isolate (SRFA, O3, tBuOH)	2.29	NA	NA	NA	NA	290-400	3.95
Guo and Yoshimura 2023 [202]	1_Iwanai	Whole Water (Reservoir)	5.79	3.25	NA	16.00	1.41	280-400	2.84
Guo and Yoshimura 2023	2_Kanayama	Whole Water (Reservoir)	6.45	3.31	NA	17.00	1.44	280-400	2.98
Guo and Yoshimura 2023	3_Katsurazawa	Whole Water (Reservoir)	5.17	3.85	NA	15.00	1.40	280-400	2.00
Guo and Yoshimura 2023	4_Tokachi	Whole Water (Reservoir)	4.68	3.95	NA	15.00	1.38	280-400	1.89
Guo and Yoshimura 2023	5_Tokisato	Whole Water (Reservoir)	5.53	3.15	NA	16.00	1.49	280-400	3.23
Guo and Yoshimura 2023	6_Chūbetsu	Whole Water (Reservoir)	4.71	2.57	NA	14.00	1.40	280-400	2.11
Guo and Yoshimura 2023	7_Shijūshida	Whole Water (Reservoir)	4.38	3.36	NA	14.00	1.52	280-400	2.45
Guo and Yoshimura 2023	8_Sagae	Whole Water (Reservoir)	4.65	3.12	NA	14.00	1.45	280-400	2.30
Guo and Yoshimura 2023	9_Naruko	Whole Water (Reservoir)	5.05	3.86	NA	15.00	1.28	280-400	2.10
Guo and Yoshimura 2023	10_Kamafusa	Whole Water (Reservoir)	4.51	3.71	NA	14.00	1.40	280-400	1.99
Guo and Yoshimura 2023	11_Shichikashuku	Whole Water (Reservoir)	4.93	3.97	NA	14.00	1.45	280-400	1.77
Guo and Yoshimura 2023	12_Gassan	Whole Water (Reservoir)	5.23	2.60	NA	16.00	1.38	280-400	2.25
Guo and Yoshimura 2023	13_Surikamigawa	Whole Water (Reservoir)	5.11	3.65	NA	15.00	1.35	280-400	1.67
Guo and Yoshimura 2023	14_Futase	Whole Water (Reservoir)	4.65	2.17	NA	15.00	1.55	280-400	2.15
Guo and Yoshimura 2023	15_Ikari	Whole Water (Reservoir)	5.42	2.09	NA	17.00	1.40	280-400	2.65
Guo and Yoshimura 2023	16_Kawamata	Whole Water (Reservoir)	6.24	2.26	NA	18.00	1.46	280-400	3.14

Table S18. Summary of $\Phi_{app, {}^1O_2}$ and optical data (continued)

Reference	Sample ID	Sample Classification	*E2:E3*	$SUVA_{254}$ (L mg C^{-1}•m^{-1})	$S_{290–400}$ / $S_{300–400}$ / $S_{290–450}$ / $S_{295–400}$ (μm^{-1})	$S_{300–600}$ / $S_{300–500}$ / $S_{300–700}$ / $S_{275–500}$ (μm^{-1})	FI	Wavelength Range (nm)	$\Phi_{app, {}^1O_2}$ ($\times 10^{-2}$ mol mol-photons^{-1})
Guo and Yoshimura 2023	17_Miyagase	Whole Water (Reservoir)	6.13	1.79	NA	17.00	1.53	280-400	3.91
Guo and Yoshimura 2023	18_Sonohara	Whole Water (Reservoir)	4.11	4.31	NA	13.00	1.42	280-400	1.63
Guo and Yoshimura 2023	19_Fujiwara	Whole Water (Reservoir)	4.52	3.66	NA	14.00	1.43	280-400	1.46
Guo and Yoshimura 2023	20_Naramata	Whole Water (Reservoir)	4.91	3.94	NA	16.00	1.31	280-400	1.63
Guo and Yoshimura 2023	21_Yagisawa	Whole Water (Reservoir)	5.25	3.42	NA	17.00	1.38	280-400	2.07
Guo and Yoshimura 2023	22_Shimokubo	Whole Water (Reservoir)	11.50	2.09	NA	53.00	1.52	280-400	6.21
Guo and Yoshimura 2023	23_Ōishi	Whole Water (Reservoir)	4.64	2.83	NA	16.00	1.39	280-400	1.78
Guo and Yoshimura 2023	24_Ōkawa	Whole Water (Reservoir)	5.27	2.71	NA	17.00	1.43	280-400	3.07
Guo and Yoshimura 2023	25_Ōmachi	Whole Water (Reservoir)	7.06	1.33	NA	22.00	1.50	280-400	3.81
Guo and Yoshimura 2023	26_Yawagi	Whole Water (Reservoir)	4.90	2.75	NA	15.00	1.36	280-400	2.23
Guo and Yoshimura 2023	27_Yokoyama	Whole Water (Reservoir)	4.33	1.10	NA	13.00	1.40	280-400	2.25
Guo and Yoshimura 2023	28_Nagashima	Whole Water (Reservoir)	8.75	1.38	NA	30.00	1.35	280-400	4.11
Guo and Yoshimura 2023	29_Misogawa	Whole Water (Reservoir)	5.76	0.60	NA	17.00	1.21	280-400	3.07
Guo and Yoshimura 2023	30_Tokuyama	Whole Water (Reservoir)	5.90	1.42	NA	17.00	1.48	280-400	3.26
Guo and Yoshimura 2023	31_Takayama	Whole Water (Reservoir)	5.10	2.87	NA	16.00	1.33	280-400	2.38
Guo and Yoshimura 2023	32_Shorenji	Whole Water (Reservoir)	5.14	3.16	NA	16.00	1.38	280-400	1.93
Guo and Yoshimura 2023	33_Hinachi	Whole Water (Reservoir)	5.16	2.75	NA	16.00	1.39	280-400	2.11
Guo and Yoshimura 2023	34_Hitokura	Whole Water (Reservoir)	5.43	2.65	NA	17.00	1.48	280-400	3.11
Guo and Yoshimura 2023	35_Tomata	Whole Water (Reservoir)	4.48	2.81	NA	14.00	1.40	280-400	1.79
Guo and Yoshimura 2023	36_Sugesawa	Whole Water (Reservoir)	4.39	2.43	NA	14.00	1.38	280-400	2.26
Guo and Yoshimura 2023	37_Haji	Whole Water (Reservoir)	4.46	3.35	NA	14.00	1.42	280-400	2.53
Guo and Yoshimura 2023	38_Hattabara	Whole Water (Reservoir)	5.59	2.93	NA	16.00	1.46	280-400	3.68
Guo and Yoshimura 2023	39_Ikeda	Whole Water (Reservoir)	4.45	1.40	NA	15.00	1.49	280-400	2.52
Guo and Yoshimura 2023	40_Sameura	Whole Water (Reservoir)	6.60	2.70	NA	20.00	1.32	280-400	1.70
Guo and Yoshimura 2023	41_Shingu	Whole Water (Reservoir)	5.45	2.30	NA	17.00	1.41	280-400	2.45
Guo and Yoshimura 2023	42_Tomisato	Whole Water (Reservoir)	5.35	1.95	NA	17.00	1.45	280-400	2.04
Guo and Yoshimura 2023	43_Ōdo	Whole Water (Reservoir)	7.75	1.16	NA	19.00	1.44	280-400	5.91
Guo and Yoshimura 2023	44_Nagayasuguchi	Whole Water (Reservoir)	3.79	2.48	NA	12.00	1.37	280-400	1.79
Guo and Yoshimura 2023	45_Tsuruda	Whole Water (Reservoir)	3.98	2.29	NA	13.00	1.51	280-400	2.17
Guo and Yoshimura 2023	46_Shimouke	Whole Water (Reservoir)	4.40	2.57	NA	14.00	1.39	280-400	2.38
Guo and Yoshimura 2023	47_Yabakei	Whole Water (Reservoir)	4.94	3.34	NA	15.00	1.40	280-400	2.05
Guo and Yoshimura 2023	48_Ryūmon	Whole Water (Reservoir)	4.19	2.29	NA	13.00	1.41	280-400	1.84
Guo and Yoshimura 2023	49_Haneji	Whole Water (Reservoir)	5.49	2.04	NA	16.00	1.43	280-400	2.82
Guo and Yoshimura 2023	50_Arakawa	Whole Water (Reservoir)	4.87	2.29	NA	15.00	1.38	280-400	1.94
Guo and Yoshimura 2023	L1_Shinobazuike	Whole Water (Lake)	7.72	0.95	NA	32.00	1.96	280-400	2.63
Guo and Yoshimura 2023	L2_Senzokuike	Whole Water (Lake)	8.08	1.31	NA	19.00	1.63	280-400	3.52
Guo and Yoshimura 2023	L3_Suwako	Whole Water (Lake)	5.29	1.82	NA	14.00	1.82	280-400	3.52
Guo and Yoshimura 2023	L4_Inbanuma	Whole Water (Lake)	5.97	2.21	NA	17.00	1.55	280-400	3.93
Guo and Yoshimura 2023	L5_Teganuma	Whole Water (Lake)	4.21	2.22	NA	13.00	1.57	280-400	3.24
Guo and Yoshimura 2023	L6_Ushikunuma	Whole Water (Lake)	5.87	1.81	NA	17.00	1.41	280-400	4.99
Guo and Yoshimura 2023	L7_Kasumigaura	Whole Water (Lake)	4.72	2.05	NA	14.00	1.58	280-400	2.95
Guo and Yoshimura 2023	SRNOM	IHSS Isolate (SRNOM)	4.73	4.63	NA	15.00	1.24	280-400	2.00
Guo and Yoshimura 2023	SRFA	IHSS Isolate (SRFA)	4.33	5.82	NA	15.00	1.18	280-400	1.44
Guo and Yoshimura 2023	UMRNOM	IHSS Isolate (UMRNOM)	5.02	4.61	NA	15.00	1.40	280-400	1.92
Kong and Song 2023 [203]	IOM 0H Taihu Lake (China)	PPL Extract (Algae Lake)	1.77	1.88	NA	NA	NA	290-400	3.14
Kong and Song 2023	IOM 1H	PPL Extract (Algae Lake, Photo)	2.18	1.88	NA	NA	NA	290-400	1.93
Kong and Song 2023	IOM 10H	PPL Extract (Algae Lake, Photo)	2.52	0.88	NA	NA	NA	290-400	1.31
Kong and Song 2023	IOM 50H	PPL Extract (Algae Lake, Photo)	2.88	0.50	NA	NA	NA	290-400	0.94
Kong and Song 2023	IOM 100H	PPL Extract (Algae Lake, Photo)	3.29	0.36	NA	NA	NA	290-400	0.46
Kong and Song 2023	EOM 0H Taihu Lake (China)	PPL Extract (Algae Lake)	3.61	1.26	NA	NA	NA	290-400	5.48
Kong and Song 2023	EOM 1H	PPL Extract (Algae Lake, Photo)	4.21	1.05	NA	NA	NA	290-400	5.47

Table S18. Summary of $\Phi_{app,\ ^1O_2}$ and optical data (continued)

Reference	Sample ID	Sample Classification	*E2:E3*	$SUVA_{254}$ (L mg C^{-1}•m^{-1})	$S_{290–400}$ / $S_{300–400}$ / $S_{290–450}$ / $S_{295–400}$ (μm^{-1})	$S_{300–600}$ / $S_{300–500}$ / $S_{300–700}$ / $S_{275–500}$ (μm^{-1})	FI	Wavelength Range (nm)	$\Phi_{app,\ ^1O_2}$ ($\times10^{-2}$ mol mol-photons^{-1})
Kong and Song 2023	EOM 10H	PPL Extract (Algae Lake, Photo)	4.76	0.76	NA	NA	NA	290-400	5.30
Kong and Song 2023	EOM 50H	PPL Extract (Algae Lake, Photo)	11.17	0.57	NA	NA	NA	290-400	5.51
Kong and Song 2023	EOM 100H	PPL Extract (Algae Lake, Photo)	11.83	0.50	NA	NA	NA	290-400	5.75
Kong and Song 2023	SRDOM 0H	IHSS Isolate (SRNOM)	4.66	3.24	NA	NA	NA	290-400	4.83
Kong and Song 2023	SRDOM 1H	IHSS Isolate (SRNOM, Photo)	5.19	3.01	NA	NA	NA	290-400	4.48
Kong and Song 2023	SRDOM 10H	IHSS Isolate (SRNOM, Photo)	5.89	2.63	NA	NA	NA	290-400	3.81
Kong and Song 2023	SRDOM 50H	IHSS Isolate (SRNOM, Photo)	6.22	1.24	NA	NA	NA	290-400	3.71
Kong and Song 2023	SRDOM 100H	IHSS Isolate (SRNOM, Photo)	7.11	0.56	NA	NA	NA	290-400	3.41
Wan and Chen 2023 [204]	EfOM	C18 Extract (WWTP Effluent, O3)	4.14	0.82	NA	NA	NA	300-600	2.01
Wan and Chen 2023	EfOM + 0.29 g O3/g TOC	C18 Extract (WWTP Effluent, O3)	4.86	0.70	NA	NA	NA	300-600	1.87
Wan and Chen 2023	EfOM + 0.53 g O3/g TOC	C18 Extract (WWTP Effluent, O3)	5.08	0.58	NA	NA	NA	300-600	1.63
Wan and Chen 2023	EfOM + 1.05 g O3/g TOC	C18 Extract (WWTP Effluent, O3)	4.41	0.47	NA	NA	NA	300-600	1.85
Wan and Chen 2023	EfOM+ 1.92 g O3/g TOC	C18 Extract (WWTP Effluent, O3)	5.51	0.43	NA	NA	NA	300-600	1.31
Wan and Chen 2023	Aldrich humic acid (HA)	XAD Fraction (Soil)	2.31	5.04	NA	NA	NA	300-600	1.13
Wan and Chen 2023	HA + 0.29 g O3/g TOC	XAD Fraction (Soil, O3)	2.36	4.79	NA	NA	NA	300-600	0.73
Wan and Chen 2023	HA + 0.53 g O3/g TOC	XAD Fraction (Soil, O3)	2.39	4.50	NA	NA	NA	300-600	0.59
Wan and Chen 2023	HA + 1.05 g O3/g TOC	XAD Fraction (Soil, O3)	2.44	4.15	NA	NA	NA	300-600	0.52
Wan and Chen 2023	HA + 1.92 g O3/g TOC	XAD Fraction (Soil, O3)	2.58	3.14	NA	NA	NA	300-600	0.45
Wan and Chen 2023	Henan Changsheng Industrial Fulvic acid (FA)	XAD Fraction (Soil)	2.33	11.96	NA	NA	NA	300-600	5.05
Wan and Chen 2023	FA + 0.29 g O3/g TOC	XAD Fraction (Soil, O3)	2.45	11.92	NA	NA	NA	300-600	3.52
Wan and Chen 2023	FA + 0.53 g O3/g TOC	XAD Fraction (Soil, O3)	2.51	11.56	NA	NA	NA	300-600	2.83
Wan and Chen 2023	FA + 1.05 g O3/g TOC	XAD Fraction (Soil, O3)	2.66	10.66	NA	NA	NA	300-600	1.92
Wan and Chen 2023	FA + 1.92 g O3/g TOC	XAD Fraction (Soil, O3)	2.79	9.99	NA	NA	NA	300-600	1.37
Wang and Chu 2023 [205]	Pine Needle BC 500 C 365 nm	DBC (pyDOM)	4.45	NA	NA	NA	NA	365	2.17
Wang and Chu 2023	Shell BC 365 nm	DBC (pyDOM)	4.28	NA	NA	NA	NA	365	2.54
Wang and Chu 2023	Straw BC 365 nm	DBC (pyDOM)	10.19	NA	NA	NA	NA	365	3.73
Wang and Chu 2023	Wood BC 365 nm	DBC (pyDOM)	8.03	NA	NA	NA	NA	365	1.65
Wang and Chu 2023	Wood BC 375 nm	DBC (pyDOM)	8.03	NA	NA	NA	NA	375	3.63
Wang and Chu 2023	Wood BC 387 nm	DBC (pyDOM)	8.03	NA	NA	NA	NA	387	2.14
Wang and Chu 2023	Wood BC 425 nm	DBC (pyDOM)	8.03	NA	NA	NA	NA	425	1.31
Wang and Chu 2023	Wood BC 461 nm	DBC (pyDOM)	8.03	NA	NA	NA	NA	461	0.76
Wang and Chu 2023	Wood BC 490 nm	DBC (pyDOM)	8.03	NA	NA	NA	NA	490	0.33
Wang and Chu 2023	Wood BC 300 C	DBC (pyDOM)	4.47	NA	NA	NA	NA	365	0.95
Wang and Chu 2023	Wood BC 500 C	DBC (pyDOM)	8.03	NA	NA	NA	NA	365	1.48
Wang and Chu 2023	Wood BC 700 C	DBC (pyDOM)	3.81	NA	NA	NA	NA	365	0.40
Wang and Chu 2023	Wood BC 900 C	DBC (pyDOM)	0.95	NA	NA	NA	NA	365	0.05
Wang and Chu 2023	Wood BC 0.22 µm	DBC (pyDOM)	NA	NA	NA	NA	NA	365	7.38
Wang and Chu 2023	Wood BC 10 µm	DBC (pyDOM)	NA	NA	NA	NA	NA	365	1.06
Wang and Chu 2023	Wood BC 80 µm	DBC (pyDOM)	NA	NA	NA	NA	NA	365	1.05
Du and Yu 2024 [206]	YQH_Whole water	Whole Water (River)	NA	NA	NA	NA	NA	290-400	10.80
Du and Yu 2024	YQH_100 mL loading	PPL Extract (River)	NA	NA	NA	NA	NA	290-400	2.40
Du and Yu 2024	YQH_2 L loading	PPL Extract (River)	NA	NA	NA	NA	NA	290-400	1.80
Du and Yu 2024	CH_Whole water	Whole Water (River)	NA	NA	NA	NA	NA	290-400	69.80
Du and Yu 2024	CH_100 mL loading	PPL Extract (River)	NA	NA	NA	NA	NA	290-400	7.20
Du and Yu 2024	CH_2 L loading	PPL Extract (River)	NA	NA	NA	NA	NA	290-400	4.90
Du and Yu 2024	YDH_Whole water	Whole Water (River)	NA	NA	NA	NA	NA	290-400	3.60
Du and Yu 2024	YDH_100 mL loading	PPL Extract (River)	NA	NA	NA	NA	NA	290-400	0.60
Du and Yu 2024	YDH_2 L loading	PPL Extract (River)	NA	NA	NA	NA	NA	290-400	0.40
Du and Yu 2024	QH_Whole water	Whole Water (WW-Impacted River)	NA	NA	NA	NA	NA	290-400	6.50
Du and Yu 2024	QH_100 mL loading	PPL Extract (WW-Impacted River)	NA	NA	NA	NA	NA	290-400	2.00
Du and Yu 2024	QH_2 L loading	PPL Extract (WW-Impacted River)	NA	NA	NA	NA	NA	290-400	1.30

**Table S18.** Summary of $\Phi_{app, {}^1O_2}$ and optical data (continued)									
Reference	Sample ID	Sample Classification	*E2:E3*	$SUVA_{254}$ (L mg C^{-1}•m^{-1})	$S_{290–400}$ / $S_{300–400}$ / $S_{290–450}$ / $S_{295–400}$ (μm^{-1})	$S_{300–600}$ / $S_{300–500}$ / $S_{300–700}$ / $S_{275–500}$ (μm^{-1})	FI	Wavelength Range (nm)	$\Phi_{app, {}^1O_2}$ ($\times 10^{-2}$ mol mol-photons^{-1})
Du and Yu 2024	YMY_Whole water	Whole Water (WW-Impacted River)	NA	NA	NA	NA	NA	290-400	29.30
Du and Yu 2024	YMY_100 mL loading	PPL Extract (WW-Impacted River)	NA	NA	NA	NA	NA	290-400	2.40
Du and Yu 2024	YMY_2 L loading	PPL Extract (WW-Impacted River)	NA	NA	NA	NA	NA	290-400	1.50
Du and Yu 2024	HGZ_Whole water	Whole Water (WW-Impacted River)	NA	NA	NA	NA	NA	290-400	14.50
Du and Yu 2024	HGZ_100 mL loading	PPL Extract (WW-Impacted River)	NA	NA	NA	NA	NA	290-400	2.00
Du and Yu 2024	HGZ_2 L loading	PPL Extract (WW-Impacted River)	NA	NA	NA	NA	NA	290-400	1.60
Du and Yu 2024	HT_Whole water	Whole Water (WW-Impacted River)	NA	NA	NA	NA	NA	290-400	73.30
Du and Yu 2024	HT_100 mL loading	PPL Extract (WW-Impacted River)	NA	NA	NA	NA	NA	290-400	11.50
Du and Yu 2024	HT_2 L loading	PPL Extract (WW-Impacted River)	NA	NA	NA	NA	NA	290-400	2.80
Du and Yu 2024	SRHA	IHSS Isolate (SRHA)	NA	NA	NA	NA	NA	290-400	1.40
Du and Yu 2024	SRFA	IHSS Isolate (SRFA)	NA	NA	NA	NA	NA	290-400	2.20
Guo and Chen 2024 [207]	Henan Changsheng Industrial Fulvic acid (FA) 365 nm	XAD Fraction (Soil)	2.50	NA	NA	NA	NA	365	375.50
Guo and Chen 2024	FA 310 nm	XAD Fraction (Soil)	NA	NA	NA	NA	NA	310	1.60
Guo and Chen 2024	FA 280 nm	XAD Fraction (Soil)	NA	NA	NA	NA	NA	280	10.60
Guo and Chen 2024	FA 260 nm	XAD Fraction (Soil)	NA	NA	NA	NA	NA	260	7.30
Guo and Chen 2024	Aldrich humic acid (HA) 365 nm	XAD Fraction (Soil)	2.30	NA	NA	NA	NA	365	134.50
Guo and Chen 2024	HA 310 nm	XAD Fraction (Soil)	NA	NA	NA	NA	NA	310	0.50
Guo and Chen 2024	HA 280 nm	XAD Fraction (Soil)	NA	NA	NA	NA	NA	280	3.90
Guo and Chen 2024	HA 260 nm	XAD Fraction (Soil)	NA	NA	NA	NA	NA	260	2.30
Guo and Chen 2024	EfOM 365 nm	PPL Extract (WWTP Effluent)	4.20	NA	NA	NA	NA	365	785.20
Guo and Chen 2024	EfOM 310 nm	PPL Extract (WWTP Effluent)	NA	NA	NA	NA	NA	310	0.90
Guo and Chen 2024	EfOM 280 nm	PPL Extract (WWTP Effluent)	NA	NA	NA	NA	NA	280	13.80
Guo and Chen 2024	EfOM 260 nm	PPL Extract (WWTP Effluent)	NA	NA	NA	NA	NA	260	10.20
He and Zhang 2024 [208]	DOMRe	Whole Water (Reservoir)	7.25	2.03	NA	NA	NA	300-600	0.29
He and Zhang 2024	DOMRi	Whole Water (River)	5.76	2.59	NA	NA	NA	300-600	0.14
Li and Nah 2024 [209]	Sep._1	Whole Water (Seawater)	10.41	0.41	NA	NA	1.56	290-700	5.09
Li and Nah 2024	Sep._2	Whole Water (Seawater)	5.94	0.21	NA	NA	1.68	290-700	2.27
Li and Nah 2024	Sep._3	Whole Water (Seawater)	6.70	0.28	NA	NA	1.74	290-700	1.65
Li and Nah 2024	Sep._4	Whole Water (Seawater)	6.34	0.44	NA	NA	1.59	290-700	0.92
Li and Nah 2024	Sep._5	Whole Water (Seawater)	5.23	0.26	NA	NA	1.45	290-700	0.78
Li and Nah 2024	Sep._6	Whole Water (Seawater)	3.97	0.28	NA	NA	1.41	290-700	0.88
Li and Nah 2024	Sep._7	Whole Water (Seawater)	4.99	0.40	NA	NA	1.56	290-700	1.03
Li and Nah 2024	Sep._8	Whole Water (Seawater)	6.62	0.19	NA	NA	1.64	290-700	2.07
Li and Nah 2024	Nov._1	Whole Water (Seawater)	9.59	0.16	NA	NA	1.62	290-700	6.14
Li and Nah 2024	Nov._2	Whole Water (Seawater)	16.54	0.29	NA	NA	1.45	290-700	4.24
Li and Nah 2024	Nov._3	Whole Water (Seawater)	13.05	0.34	NA	NA	1.79	290-700	4.13
Li and Nah 2024	Nov._4	Whole Water (Seawater)	11.37	0.53	NA	NA	1.39	290-700	1.83
Li and Nah 2024	Nov._5	Whole Water (Seawater)	12.36	0.23	NA	NA	1.57	290-700	3.38
Li and Nah 2024	Nov._6	Whole Water (Seawater)	13.23	0.41	NA	NA	1.60	290-700	3.19
Li and Nah 2024	Nov._7	Whole Water (Seawater)	13.59	0.53	NA	NA	1.37	290-700	3.69
Li and Nah 2024	Nov._8	Whole Water (Seawater)	12.84	0.14	NA	NA	1.70	290-700	2.69
Li and Nah 2024	SRNOM	IHSS Isolate (SRNOM)	4.42	3.36	NA	NA	NA	290-700	1.66
Madhiyan and Moor 2024 [210]	SRNOM 300 nm phosphorescence	IHSS Isolate (SRNOM)	NA	NA	NA	NA	NA	300	2.70
Madhiyan and Moor 2024	Grizzly Creek 300 nm	DBC (pyDOM)	NA	NA	NA	NA	NA	330	5.70
Madhiyan and Moor 2024	Oak250 pyDOM 300 nm	DBC (pyDOM)	NA	NA	NA	NA	NA	365	6.30
Madhiyan and Moor 2024	Pine150 pyDOM 300 nm	DBC (pyDOM)	NA	NA	NA	NA	NA	390	4.50
Madhiyan and Moor 2024	Pine250 pyDOM 300 nm	DBC (pyDOM)	NA	NA	NA	NA	NA	420	8.20
Madhiyan and Moor 2024	Pine350 pyDOM 300 nm	DBC (pyDOM)	NA	NA	NA	NA	NA	430	1.70
Madhiyan and Moor 2024	Grizzly Creek 330 nm	DBC (pyDOM)	NA	NA	NA	NA	NA	500	3.50
Madhiyan and Moor 2024	Oak250 pyDOM 330 nm	DBC (pyDOM)	NA	NA	NA	NA	NA	300	4.30
Madhiyan and Moor 2024	Pine250 pyDOM 330 nm	DBC (pyDOM)	NA	NA	NA	NA	NA	365	3.70

**Table S18.** Summary of $\Phi_{app,\ ^1O_2}$ and optical data (continued)									
Reference	Sample ID	Sample Classification	*E2:E3*	$SUVA_{254}$ (L mg C^{-1}•m^{-1})	$S_{290-400}$ / $S_{300-400}$ / $S_{290-450}$ / $S_{295-400}$ (μm^{-1})	$S_{300-600}$ / $S_{300-500}$ / $S_{300-700}$ / $S_{275-500}$ (μm^{-1})	FI	Wavelength Range (nm)	$\Phi_{app,\ ^1O_2}$ (×10^{-2} mol mol-photons^{-1})
Madhiyan and Moor 2024	SRNOM 365 nm	IHSS Isolate (SRNOM)	NA	NA	NA	NA	NA	420	2.40
Madhiyan and Moor 2024	Grizzly Creek 365 nm	DBC (pyDOM)	NA	NA	NA	NA	NA	430	2.10
Madhiyan and Moor 2024	Oak250 pyDOM 365 nm	DBC (pyDOM)	NA	NA	NA	NA	NA	450	2.40
Madhiyan and Moor 2024	Pine150 pyDOM 365 nm	DBC (pyDOM)	NA	NA	NA	NA	NA	500	2.10
Madhiyan and Moor 2024	Pine250 pyDOM 365 nm	DBC (pyDOM)	NA	NA	NA	NA	NA	300	2.70
Madhiyan and Moor 2024	Pine350 pyDOM 365 nm	DBC (pyDOM)	NA	NA	NA	NA	NA	330	0.60
Madhiyan and Moor 2024	Grizzly Creek 390 nm	DBC (pyDOM)	NA	NA	NA	NA	NA	390	2.00
Madhiyan and Moor 2024	Oak250 pyDOM 390 nm	DBC (pyDOM)	NA	NA	NA	NA	NA	420	1.50
Madhiyan and Moor 2024	Pine250 pyDOM 390 nm	DBC (pyDOM)	NA	NA	NA	NA	NA	450	2.10
Madhiyan and Moor 2024	Grizzly Creek 420 nm	DBC (pyDOM)	NA	NA	NA	NA	NA	330	1.80
Madhiyan and Moor 2024	Pine250 pyDOM 420 nm	DBC (pyDOM)	NA	NA	NA	NA	NA	420	0.80
Madhiyan and Moor 2024	SRNOM 430 nm	IHSS Isolate (SRNOM)	NA	NA	NA	NA	NA	450	1.30
Madhiyan and Moor 2024	Grizzly Creek 450 nm	DBC (pyDOM)	NA	NA	NA	NA	NA	430	0.90
Madhiyan and Moor 2024	Oak250 pyDOM 450 nm	DBC (pyDOM)	NA	NA	NA	NA	NA	450	0.70
Madhiyan and Moor 2024	Pine150 pyDOM 450 nm	DBC (pyDOM)	NA	NA	NA	NA	NA	500	0.20
Madhiyan and Moor 2024	Pine250 pyDOM 450 nm	DBC (pyDOM)	NA	NA	NA	NA	NA	300	0.50
Madhiyan and Moor 2024	Pine350 pyDOM 450 nm	DBC (pyDOM)	NA	NA	NA	NA	NA	330	0.20
Madhiyan and Moor 2024	SRNOM 500 nm	IHSS Isolate (SRNOM)	NA	NA	NA	NA	NA	365	0.60
Madhiyan and Moor 2024	Grizzly Creek 500 nm	DBC (pyDOM)	NA	NA	NA	NA	NA	390	0.50
Madhiyan and Moor 2024	Pine250 pyDOM 500 nm	DBC (pyDOM)	NA	NA	NA	NA	NA	450	0.50
Madhiyan and Moor 2024	SRNOM polychromatic	IHSS Isolate (SRNOM)	NA	NA	NA	NA	NA	300-430	1.81
Madhiyan and Moor 2024	Grizzly Creek polychromatic	DBC (pyDOM)	NA	NA	NA	NA	NA	300-450	2.10
Madhiyan and Moor 2024	Oak250 pyDOM polychromatic	DBC (pyDOM)	NA	NA	NA	NA	NA	300-450	2.31
Madhiyan and Moor 2024	Pine150 pyDOM polychromatic	DBC (pyDOM)	NA	NA	NA	NA	NA	300-450	1.80
Madhiyan and Moor 2024	Pine250 pyDOM polychromatic	DBC (pyDOM)	NA	NA	NA	NA	NA	300-450	2.31
Madhiyan and Moor 2024	Pine350 pyDOM polychromatic	DBC (pyDOM)	NA	NA	NA	NA	NA	300-450	0.51
Madhiyan and Moor 2024	1,2-benzenedicarboxylic acid 300 nm	Model Compound	NA	NA	NA	NA	NA	300	21.80
Madhiyan and Moor 2024	1,2,4-benzenetricarboxylic acid 300 nm	Model Compound	NA	NA	NA	NA	NA	300	8.20
Madhiyan and Moor 2024	1,2,3-benzetricarboxylic acid 300 nm	Model Compound	NA	NA	NA	NA	NA	300	13.90
Madhiyan and Moor 2024	SRNOM 365 nm FFA	IHSS Isolate (SRNOM)	NA	NA	NA	NA	NA	300-430	1.10
Madhiyan and Moor 2024	Grizzly Creek 365 nm FFA	DBC (pyDOM)	NA	NA	NA	NA	NA	300-450	1.40
Wasswa and Zeng 2024 [211]	Lake 2 06/08/2019	Whole Water (Lake)	5.97	2.89	17.51	NA	1.58	290-550	1.97
Wasswa and Zeng 2024	Lake 2 08/31/2019	Whole Water (Lake)	7.88	3.01	19.64	NA	1.59	290-550	2.09
Wasswa and Zeng 2024	Lake 6 06/18/2019	Whole Water (Lake)	5.69	3.12	16.69	NA	1.50	290-550	1.85
Wasswa and Zeng 2024	Lake 6 08/28/2019	Whole Water (Lake)	6.98	3.21	18.28	NA	1.63	290-550	3.31
Wasswa and Zeng 2024	Lake 8 08/05/2019	Whole Water (Lake)	7.26	2.39	18.37	NA	1.55	290-550	1.87
Wasswa and Zeng 2024	Lake 12 06/08/2019	Whole Water (Lake)	6.93	2.92	18.80	NA	1.53	290-550	1.93
Wasswa and Zeng 2024	Lake 12 09/01/2019	Whole Water (Lake)	7.83	2.80	18.60	NA	1.64	290-550	2.33
Wasswa and Zeng 2024	Lake 13 07/08/2018	Whole Water (Lake)	4.91	3.19	14.49	NA	1.48	290-550	3.01
Wasswa and Zeng 2024	Lake 13 07/22/2018	Whole Water (Lake)	6.92	2.92	19.05	NA	1.46	290-550	2.29
Wasswa and Zeng 2024	Lake 13 08/06/2018	Whole Water (Lake)	9.47	2.37	22.08	NA	1.62	290-550	2.65
Wasswa and Zeng 2024	Lake 13 08/21/2018	Whole Water (Lake)	6.78	2.83	18.34	NA	1.52	290-550	2.94
Wasswa and Zeng 2024	Lake 13 09/02/2018	Whole Water (Lake)	10.90	1.97	24.87	NA	1.74	290-550	2.73
Wasswa and Zeng 2024	Lake 13 06/10/2019	Whole Water (Lake)	5.41	3.04	16.66	NA	1.43	290-550	1.68
Wasswa and Zeng 2024	Lake 13 08/31/2019	Whole Water (Lake)	5.95	2.92	17.38	NA	1.51	290-550	2.06
Wasswa and Zeng 2024	Lake 17 07/01/2018	Whole Water (Lake)	8.03	1.78	19.24	NA	1.70	290-550	2.03
Wasswa and Zeng 2024	Lake 17 07/15/2018	Whole Water (Lake)	7.06	2.45	17.09	NA	1.69	290-550	1.85
Wasswa and Zeng 2024	Lake 17 07/28/2018	Whole Water (Lake)	6.86	2.49	16.57	NA	1.63	290-550	2.06
Wasswa and Zeng 2024	Lake 17 08/12/2018	Whole Water (Lake)	9.18	2.32	22.48	NA	1.62	290-550	2.29
Wasswa and Zeng 2024	Lake 17 08/26/2018	Whole Water (Lake)	10.92	1.26	25.12	NA	1.70	290-550	2.37
Wasswa and Zeng 2024	Lake 17 07/15/2019	Whole Water (Lake)	8.33	2.38	19.84	NA	1.65	290-550	2.06

**Table S18.** Summary of $\Phi_{app, {}^1O_2}$ and optical data (continued)									
Reference	Sample ID	Sample Classification	*E2:E3*	SUVA$_{254}$ (L mg C^{-1}•m^{-1})	$S_{290–400}$ / $S_{300–400}$ / $S_{290–450}$ / $S_{295–400}$ (μm^{-1})	$S_{300–600}$ / $S_{300–500}$ / $S_{300–700}$ / $S_{275–500}$ (μm^{-1})	FI	Wavelength Range (nm)	$\Phi_{app, {}^1O_2}$ (×10^{-2} mol mol-photons^{-1})
Wasswa and Zeng 2024	Lake 17 08/12/2019	Whole Water (Lake)	7.13	2.00	17.43	NA	1.63	290-550	2.07
Wasswa and Zeng 2024	Lake 18 08/05/2018	Whole Water (Lake)	10.05	1.71	21.14	NA	1.69	290-550	2.11
Wasswa and Zeng 2024	Lake 18 08/18/2018	Whole Water (Lake)	9.10	1.77	20.95	NA	1.65	290-550	2.10
Wasswa and Zeng 2024	Lake 18 09/09/2018	Whole Water (Lake)	7.05	1.99	17.18	NA	1.76	290-550	1.94
Wasswa and Zeng 2024	Lake 18 09/30/2018	Whole Water (Lake)	6.52	2.07	18.24	NA	1.76	290-550	2.46
Wasswa and Zeng 2024	Lake 18 06/15/2019	Whole Water (Lake)	7.55	1.89	20.05	NA	1.78	290-550	2.11
Wasswa and Zeng 2024	Lake 18 08/12/2019	Whole Water (Lake)	8.49	1.83	18.66	NA	1.72	290-550	1.94
Wasswa and Zeng 2024	Lake 21 07/02/2019	Whole Water (Lake)	8.56	1.84	19.61	NA	1.62	290-550	2.01
Wasswa and Zeng 2024	Lake 21 08/13/2019	Whole Water (Lake)	9.11	1.84	20.18	NA	1.69	290-550	2.06
Wasswa and Zeng 2024	Lake 22 06/23/2019	Whole Water (Lake)	10.14	2.40	21.17	NA	1.56	290-550	2.04
Wasswa and Zeng 2024	Lake 22 06/23/2019	Whole Water (Lake)	8.15	2.34	19.75	NA	1.74	290-550	2.01
Wasswa and Zeng 2024	Lake 22 09/16/2019	Whole Water (Lake)	10.00	2.22	20.52	NA	1.67	290-550	2.11
Wasswa and Zeng 2024	Lake 22 09/16/2019	Whole Water (Lake)	9.51	2.26	19.70	NA	1.55	290-550	2.19
Wasswa and Zeng 2024	Lake 23 06/12/2019	Whole Water (Lake)	6.05	2.49	16.81	NA	1.54	290-550	1.93
Wasswa and Zeng 2024	Lake 23 09/17/2019	Whole Water (Lake)	7.56	2.70	18.69	NA	1.60	290-550	2.70
Wasswa and Zeng 2024	Lake 25 07/08/2018	Whole Water (Lake)	8.92	2.61	20.03	NA	1.72	290-550	2.30
Wasswa and Zeng 2024	Lake 25 07/29/2018	Whole Water (Lake)	9.91	2.39	22.51	NA	1.76	290-550	2.40
Wasswa and Zeng 2024	Lake 25 06/09/2019	Whole Water (Lake)	10.05	2.31	22.24	NA	1.73	290-550	2.35
Wasswa and Zeng 2024	Lake 29 06/02/2019	Whole Water (Lake)	7.62	1.61	19.59	NA	1.56	290-550	2.01
Wasswa and Zeng 2024	Lake 29 08/12/2019	Whole Water (Lake)	7.82	1.76	18.18	NA	1.59	290-550	1.85
Wasswa and Zeng 2024	Lake 31 06/15/2019	Whole Water (Lake)	5.79	3.18	17.11	NA	1.52	290-550	2.74
Wasswa and Zeng 2024	Lake 31 08/27/2019	Whole Water (Lake)	6.77	3.21	18.45	NA	1.53	290-550	2.13
Wasswa and Zeng 2024	Lake 33 08/18/2019	Whole Water (Lake)	8.45	2.14	19.37	NA	1.70	290-550	2.25
Wasswa and Zeng 2024	Lake 33 09/01/2019	Whole Water (Lake)	7.46	2.37	18.18	NA	1.75	290-550	2.90
Wasswa and Zeng 2024	Lake 34 06/04/2019	Whole Water (Lake)	5.73	2.97	17.44	NA	1.52	290-550	1.78
Wasswa and Zeng 2024	Lake 34 06/04/2019	Whole Water (Lake)	5.34	2.97	16.11	NA	1.45	290-550	1.61
Wasswa and Zeng 2024	Lake 37 06/23/2019	Whole Water (Lake)	9.44	1.84	23.17	NA	1.62	290-550	2.52
Wasswa and Zeng 2024	Lake 37 08/19/2019	Whole Water (Lake)	8.45	2.03	18.56	NA	1.85	290-550	2.03
Wasswa and Zeng 2024	Lake 38 06/17/2019	Whole Water (Lake)	7.37	1.86	19.23	NA	1.65	290-550	1.93
Wasswa and Zeng 2024	Lake 39 07/20/2019	Whole Water (Lake)	9.60	1.82	21.74	NA	1.64	290-550	2.50
Wasswa and Zeng 2024	Lake 40 06/27/2018	Whole Water (Lake)	6.74	2.63	19.09	NA	1.47	290-550	1.96
Wasswa and Zeng 2024	Lake 40 07/14/2018	Whole Water (Lake)	11.83	1.75	27.66	NA	1.67	290-550	3.70
Wasswa and Zeng 2024	Lake 40 08/01/2018	Whole Water (Lake)	8.87	1.76	20.80	NA	1.66	290-550	2.18
Wasswa and Zeng 2024	Lake 40 08/12/2018	Whole Water (Lake)	11.16	1.68	23.77	NA	1.70	290-550	3.16
Wasswa and Zeng 2024	Lake 40 08/29/2018	Whole Water (Lake)	6.68	2.82	18.58	NA	1.50	290-550	3.04
Wasswa and Zeng 2024	Lake 40 06/19/2019	Whole Water (Lake)	10.45	1.73	26.30	NA	1.55	290-550	2.39
Wasswa and Zeng 2024	Lake 41 07/07/2018	Whole Water (Lake)	10.31	2.00	21.91	NA	1.65	290-550	3.10
Wasswa and Zeng 2024	Lake 41 07/17/2018	Whole Water (Lake)	11.99	1.98	25.95	NA	1.63	290-550	2.46
Wasswa and Zeng 2024	Lake 41 07/29/2018	Whole Water (Lake)	11.48	1.94	24.03	NA	1.70	290-550	3.07
Wasswa and Zeng 2024	Lake 41 08/19/2018	Whole Water (Lake)	11.69	1.90	24.61	NA	1.66	290-550	3.14
Wasswa and Zeng 2024	Lake 45 08/18/2019	Whole Water (Lake)	7.76	2.27	19.88	NA	1.78	290-550	2.12
Wasswa and Zeng 2024	Lake 49 07/08/2019	Whole Water (Lake)	9.47	2.01	21.09	NA	1.67	290-550	2.25
Wasswa and Zeng 2024	Lake 50 06/10/2019	Whole Water (Lake)	7.58	2.59	19.97	NA	1.56	290-550	2.05
Wasswa and Zeng 2024	Lake 52 06/30/2019	Whole Water (Lake)	8.92	1.50	23.68	NA	1.59	290-550	2.96
Wasswa and Zeng 2024	Lake 53 06/09/2019	Whole Water (Lake)	7.02	1.90	17.43	NA	1.44	290-550	1.77
Wasswa and Zeng 2024	Lake 57 06/29/2019	Whole Water (Lake)	8.19	2.03	21.56	NA	1.60	290-550	2.15
Wasswa and Zeng 2024	Lake 57 09/08/2019	Whole Water (Lake)	9.61	2.10	21.26	NA	1.74	290-550	2.15
Wasswa and Zeng 2024	Lake 58 09/10/2019	Whole Water (Lake)	4.78	3.85	15.66	NA	1.49	290-550	3.51
Wasswa and Zeng 2024	Lake 61 07/09/2018	Whole Water (Lake)	8.66	1.93	19.69	NA	1.67	290-550	2.01
Wasswa and Zeng 2024	Lake 61 07/19/2018	Whole Water (Lake)	5.73	2.07	13.55	NA	1.73	290-550	2.26
Wasswa and Zeng 2024	Lake 61 08/19/2018	Whole Water (Lake)	8.27	1.88	19.68	NA	1.62	290-550	2.00

**Table S18.** Summary of $\Phi_{app, ^1O_2}$ and optical data (continued)									
Reference	Sample ID	Sample Classification	*E2:E3*	SUVA$_{254}$ (L mg C^{-1}•m^{-1})	$S_{290–400}$ / $S_{300–400}$ / $S_{290–450}$ / $S_{295–400}$ (μm^{-1})	$S_{300–600}$ / $S_{300–500}$ / $S_{300–700}$ / $S_{275–500}$ (μm^{-1})	FI	Wavelength Range (nm)	$\Phi_{app, ^1O_2}$ (×10^{-2} mol mol-photons^{-1})
Wasswa and Zeng 2024	Lake 61 09/02/2018	Whole Water (Lake)	11.12	1.81	25.78	NA	1.65	290-550	3.40
Wasswa and Zeng 2024	Lake 61 09/22/2018	Whole Water (Lake)	7.89	2.36	19.21	NA	1.69	290-550	1.94
Wasswa and Zeng 2024	Lake 61 07/21/2019	Whole Water (Lake)	10.31	1.95	22.96	NA	1.63	290-550	2.48
Wasswa and Zeng 2024	Lake 61 07/23/2019	Whole Water (Lake)	10.32	1.94	22.28	NA	1.62	290-550	2.89
Wasswa and Zeng 2024	Lake 66 08/16/2019	Whole Water (Lake)	9.13	2.76	20.08	NA	1.70	290-550	3.00
Wasswa and Zeng 2024	Lake 68 09/17/2019	Whole Water (Lake)	9.28	2.70	21.14	NA	1.53	290-550	2.35
Wasswa and Zeng 2024	Lake 69 06/09/2019	Whole Water (Lake)	6.09	2.66	17.89	NA	1.46	290-550	1.80
Wasswa and Zeng 2024	Lake 69 08/19/2019	Whole Water (Lake)	6.13	3.15	17.36	NA	1.49	290-550	1.85
Wasswa and Zeng 2024	Lake 72 07/12/2019	Whole Water (Lake)	5.47	3.75	16.73	NA	1.50	290-550	1.96
Wasswa and Zeng 2024	Lake 73 07/20/2019	Whole Water (Lake)	8.05	2.69	19.61	NA	1.58	290-550	2.00
Wasswa and Zeng 2024	Lake 73 08/24/2019	Whole Water (Lake)	8.11	2.61	19.06	NA	1.69	290-550	2.21
Wasswa and Zeng 2024	Lake 74 06/08/2019	Whole Water (Lake)	7.07	2.70	18.99	NA	1.54	290-550	1.94
Wasswa and Zeng 2024	Lake 74 08/18/2019	Whole Water (Lake)	8.73	2.51	19.98	NA	1.66	290-550	1.92
Wasswa and Zeng 2024	Lake 75 06/02/2019	Whole Water (Lake)	8.47	1.74	20.62	NA	1.49	290-550	2.14
Wasswa and Zeng 2024	Lake 77 06/16/2019	Whole Water (Lake)	6.62	3.27	18.47	NA	1.59	290-550	2.14
Wasswa and Zeng 2024	Lake 77 06/16/2019	Whole Water (Lake)	5.54	3.37	15.92	NA	1.57	290-550	2.18
Wasswa and Zeng 2024	Lake 77 09/22/2019	Whole Water (Lake)	7.97	3.10	19.71	NA	1.59	290-550	2.92
Wasswa and Zeng 2024	Lake 77 09/22/2019	Whole Water (Lake)	8.34	3.09	20.67	NA	1.61	290-550	2.21
Wasswa and Zeng 2024	Lake 78 06/02/2019	Whole Water (Lake)	6.04	2.13	16.57	NA	1.51	290-550	2.67
Wasswa and Zeng 2024	Lake 88 06/24/2019	Whole Water (Lake)	7.57	2.28	20.63	NA	1.48	290-550	2.23
Wasswa and Zeng 2024	Lake 88 08/21/2019	Whole Water (Lake)	8.38	2.09	19.75	NA	1.51	290-550	1.82
Wasswa and Zeng 2024	Lake 89 07/10/2019	Whole Water (Lake)	8.24	2.47	19.67	NA	1.57	290-550	1.98
Wasswa and Zeng 2024	Lake 90 07/01/2018	Whole Water (Lake)	7.33	2.34	18.07	NA	1.67	290-550	1.86
Wasswa and Zeng 2024	Lake 90 08/12/2018	Whole Water (Lake)	6.51	3.22	18.41	NA	1.53	290-550	2.58
Wasswa and Zeng 2024	Lake 90 09/11/2018	Whole Water (Lake)	6.04	3.44	17.57	NA	1.54	290-550	1.98
Wasswa and Zeng 2024	Lake 90 06/08/2019	Whole Water (Lake)	5.88	3.04	17.18	NA	1.46	290-550	1.71
Wasswa and Zeng 2024	Lake 90 08/17/2019	Whole Water (Lake)	7.19	3.05	18.42	NA	1.54	290-550	1.88
Wasswa and Zeng 2024	Lake 92 08/06/2018	Whole Water (Lake)	8.85	1.64	25.88	NA	1.95	290-550	3.52
Wasswa and Zeng 2024	Lake 92 08/19/2018	Whole Water (Lake)	13.39	1.52	24.86	NA	1.95	290-550	3.22
Wasswa and Zeng 2024	Lake 92 09/03/2018	Whole Water (Lake)	8.57	1.93	19.96	NA	1.68	290-550	2.09
Wasswa and Zeng 2024	Lake 92 06/04/2019	Whole Water (Lake)	12.14	1.44	29.55	NA	1.68	290-550	3.96
Wasswa and Zeng 2024	Lake 92 06/17/2019	Whole Water (Lake)	11.22	1.56	24.74	NA	1.70	290-550	3.28
Wasswa and Zeng 2024	Lake 92 08/11/2019	Whole Water (Lake)	11.47	1.58	21.17	NA	1.76	290-550	2.30
Wasswa and Zeng 2024	Lake 92 09/01/2019	Whole Water (Lake)	8.85	1.51	17.51	NA	1.72	290-550	1.90
Wasswa and Zeng 2024	Lake 96 08/05/2019	Whole Water (Lake)	7.95	2.02	18.14	NA	1.73	290-550	2.03
Wasswa and Zeng 2024	Lake 99 08/10/2019	Whole Water (Lake)	5.98	3.43	17.39	NA	1.49	290-550	1.94
Wasswa and Zeng 2024	Lake 100 06/23/2019	Whole Water (Lake)	5.87	2.83	17.69	NA	1.47	290-550	1.81
Wasswa and Zeng 2024	Lake 100 08/18/2019	Whole Water (Lake)	6.80	2.62	17.92	NA	1.58	290-550	1.81
Wasswa and Zeng 2024	Lake 102 06/16/2019	Whole Water (Lake)	7.49	2.45	18.60	NA	1.71	290-550	1.90
Wasswa and Zeng 2024	Lake 102 09/22/2019	Whole Water (Lake)	7.03	2.95	17.40	NA	1.79	290-550	2.30
Wasswa and Zeng 2024	Lake 103 06/09/2019	Whole Water (Lake)	5.01	3.29	15.07	NA	1.51	290-550	1.58
Wasswa and Zeng 2024	Lake 107 08/15/2019	Whole Water (Lake)	8.08	2.22	19.73	NA	1.59	290-550	2.02
Wasswa and Zeng 2024	Lake 108 08/17/2019	Whole Water (Lake)	9.16	2.07	20.42	NA	1.67	290-550	2.15
Wasswa and Zeng 2024	Lake 109 07/08/2019	Whole Water (Lake)	6.70	3.69	18.92	NA	1.57	290-550	2.56
Wasswa and Zeng 2024	Lake 109 08/18/2019	Whole Water (Lake)	7.67	3.55	19.38	NA	1.63	290-550	3.19
Wasswa and Zeng 2024	Lake 115 07/08/2019	Whole Water (Lake)	8.98	2.33	22.42	NA	1.70	290-550	2.08
Wasswa and Zeng 2024	Lake 115 07/08/2019	Whole Water (Lake)	8.91	2.35	20.68	NA	1.61	290-550	2.31
Wasswa and Zeng 2024	Lake 115 09/09/2019	Whole Water (Lake)	7.82	2.41	17.84	NA	1.66	290-550	1.96
Wasswa and Zeng 2024	Lake 115 09/09/2019	Whole Water (Lake)	7.70	2.41	17.70	NA	1.65	290-550	2.03
Wasswa and Zeng 2024	Lake 117 08/22/2019	Whole Water (Lake)	7.44	2.39	18.85	NA	1.55	290-550	1.94
Wasswa and Zeng 2024	Lake 117 09/05/2019	Whole Water (Lake)	7.70	2.25	19.35	NA	1.58	290-550	1.95

Table S18. Summary of $\Phi_{app,\ ^1O_2}$ and optical data (continued)

Reference	Sample ID	Sample Classification	*E2:E3*	SUVA$_{254}$ (L mg C^{-1}•m^{-1})	$S_{290–400}$ / $S_{300–400}$ / $S_{290–450}$ / $S_{295–400}$ (μm^{-1})	$S_{300–600}$ / $S_{300–500}$ / $S_{300–700}$ / $S_{275–500}$ (μm^{-1})	FI	Wavelength Range (nm)	$\Phi_{app,\ ^1O_2}$ (×10^{-2} mol mol-photons^{-1})
Wasswa and Zeng 2024	Lake 120 06/10/2019	Whole Water (Lake)	7.48	2.40	19.48	NA	1.55	290-550	2.01
Wasswa and Zeng 2024	Lake 120 08/19/2019	Whole Water (Lake)	8.83	2.37	19.88	NA	1.59	290-550	2.14
Wasswa and Zeng 2024	Lake 126 07/08/2018	Whole Water (Lake)	9.25	1.80	21.58	NA	1.57	290-550	1.96
Wasswa and Zeng 2024	Lake 126 07/21/2018	Whole Water (Lake)	7.28	2.24	21.80	NA	1.70	290-550	2.12
Wasswa and Zeng 2024	Lake 126 08/17/2019	Whole Water (Lake)	7.88	2.03	18.84	NA	1.65	290-550	2.06
Wasswa and Zeng 2024	Lake 130 08/25/2019	Whole Water (Lake)	8.53	2.16	19.22	NA	1.63	290-550	2.04
Wasswa and Zeng 2024	Lake 130 09/22/2019	Whole Water (Lake)	8.87	2.21	20.36	NA	1.57	290-550	2.12
Wasswa and Zeng 2024	Lake 132 06/10/2019	Whole Water (Lake)	6.01	2.17	16.97	NA	1.63	290-550	1.71
Wasswa and Zeng 2024	Lake 132 09/03/2019	Whole Water (Lake)	7.40	2.09	17.49	NA	1.75	290-550	2.44
Wasswa and Zeng 2024	Lake 133 06/04/2019	Whole Water (Lake)	8.77	2.49	18.35	NA	1.60	290-550	1.89
Wasswa and Zeng 2024	Lake 133 08/19/2019	Whole Water (Lake)	11.42	2.41	20.75	NA	1.69	290-550	2.17
Wasswa and Zeng 2024	Lake 135 05/27/2019	Whole Water (Lake)	6.23	2.70	18.04	NA	1.46	290-550	1.89
Wasswa and Zeng 2024	Lake 135 08/31/2019	Whole Water (Lake)	8.44	2.52	20.43	NA	1.55	290-550	2.13
Wasswa and Zeng 2024	Lake 136 06/08/2019	Whole Water (Lake)	7.43	2.42	18.16	NA	1.54	290-550	1.92
Wasswa and Zeng 2024	Lake 136 09/20/2019	Whole Water (Lake)	9.23	2.34	19.79	NA	1.60	290-550	2.44
Wasswa and Zeng 2024	Lake 137 08/18/2019	Whole Water (Lake)	13.13	1.75	23.11	NA	1.61	290-550	3.13
Wasswa and Zeng 2024	Lake 139 08/06/2018	Whole Water (Lake)	5.08	1.44	13.04	NA	1.85	290-550	1.47
Wasswa and Zeng 2024	Lake 139 09/19/2018	Whole Water (Lake)	6.29	1.03	14.56	NA	1.42	290-550	2.38
Wasswa and Zeng 2024	Lake 145 06/06/2019	Whole Water (Lake)	5.83	3.05	17.43	NA	1.49	290-550	1.76
Wasswa and Zeng 2024	Lake 145 08/15/2019	Whole Water (Lake)	6.59	3.02	18.19	NA	1.46	290-550	1.84
Wasswa and Zeng 2024	Lake 149 06/10/2019	Whole Water (Lake)	7.34	2.37	19.13	NA	1.52	290-550	1.95
Wasswa and Zeng 2024	Lake 149 09/21/2019	Whole Water (Lake)	11.67	1.91	23.94	NA	1.59	290-550	2.42
Wasswa and Zeng 2024	Lake 153 08/25/2019	Whole Water (Lake)	8.04	2.14	18.61	NA	1.53	290-550	1.72
Wasswa and Zeng 2024	Lake 153 09/08/2019	Whole Water (Lake)	7.20	2.12	16.97	NA	1.67	290-550	1.92
Wasswa and Zeng 2024	Lake 164 06/23/2019	Whole Water (Lake)	8.03	2.47	19.57	NA	1.75	290-550	2.00
Wasswa and Zeng 2024	Lake 164 08/27/2019	Whole Water (Lake)	5.96	3.00	18.49	NA	1.62	290-550	2.03
Wasswa and Zeng 2024	Lake 166 07/14/2019	Whole Water (Lake)	9.95	2.11	21.06	NA	1.68	290-550	2.28
Wasswa and Zeng 2024	Lake 166 09/29/2019	Whole Water (Lake)	10.61	2.14	22.59	NA	1.67	290-550	2.01
Wasswa and Zeng 2024	Lake 169 07/22/2019	Whole Water (Lake)	8.90	2.35	21.06	NA	1.55	290-550	2.38
Wasswa and Zeng 2024	Lake 169 08/18/2019	Whole Water (Lake)	9.58	2.23	21.19	NA	1.57	290-550	2.35
Wasswa and Zeng 2024	Lake 176 07/15/2018	Whole Water (Lake)	6.45	2.66	18.23	NA	1.48	290-550	1.85
Wasswa and Zeng 2024	Lake 176 06/15/2019	Whole Water (Lake)	5.47	3.04	16.86	NA	1.44	290-550	1.72
Wasswa and Zeng 2024	Lake 176 08/19/2019	Whole Water (Lake)	6.02	2.99	17.55	NA	1.49	290-550	1.80
Wasswa and Zeng 2024	Lake 177 08/19/2019	Whole Water (Lake)	6.64	2.68	18.41	NA	1.56	290-550	1.89
Wasswa and Zeng 2024	Lake 178 08/06/2019	Whole Water (Lake)	9.28	2.38	19.87	NA	1.56	290-550	2.01
Wasswa and Zeng 2024	Lake 182 07/17/2018	Whole Water (Lake)	11.77	1.76	23.93	NA	1.74	290-550	2.10
Wasswa and Zeng 2024	Lake 182 07/31/2018	Whole Water (Lake)	9.82	2.16	23.61	NA	1.74	290-550	2.46
Wasswa and Zeng 2024	Lake 182 08/14/2018	Whole Water (Lake)	10.05	1.90	23.66	NA	1.58	290-550	3.38
Wasswa and Zeng 2024	Lake 182 09/11/2018	Whole Water (Lake)	11.48	1.80	27.63	NA	1.65	290-550	3.57
Wasswa and Zeng 2024	Lake 182 06/18/2019	Whole Water (Lake)	9.32	1.72	20.30	NA	1.72	290-550	3.25
Wasswa and Zeng 2024	Lake 182 06/18/2019	Whole Water (Lake)	8.50	1.77	18.79	NA	1.71	290-550	2.40
Wasswa and Zeng 2024	Lake 182 07/01/2019	Whole Water (Lake)	7.79	2.21	21.05	NA	1.64	290-550	2.17
Wasswa and Zeng 2024	Lake 182 07/08/2019	Whole Water (Lake)	9.76	2.02	20.58	NA	1.63	290-550	1.89
Wasswa and Zeng 2024	Lake 182 08/12/2019	Whole Water (Lake)	12.17	1.74	22.94	NA	1.84	290-550	1.93
Wasswa and Zeng 2024	Lake 182 08/12/2019	Whole Water (Lake)	10.17	1.74	20.27	NA	1.73	290-550	2.14
Wasswa and Zeng 2024	Lake 182 08/27/2019	Whole Water (Lake)	7.89	1.90	18.15	NA	1.92	290-550	2.05
Wasswa and Zeng 2024	Lake 183 08/25/2019	Whole Water (Lake)	7.40	2.24	18.61	NA	1.58	290-550	1.91
Wasswa and Zeng 2024	Lake 183 09/10/2019	Whole Water (Lake)	7.84	2.43	19.25	NA	1.59	290-550	1.97
Wasswa and Zeng 2024	Lake 190 06/17/2019	Whole Water (Lake)	7.24	3.22	18.99	NA	1.90	290-550	3.26
Wasswa and Zeng 2024	Lake 190 08/19/2019	Whole Water (Lake)	8.33	3.20	19.16	NA	1.64	290-550	3.26
Wasswa and Zeng 2024	Lake 192 09/25/2019	Whole Water (Lake)	5.95	3.13	17.36	NA	1.53	290-550	1.89

**Table S18.** Summary of $\Phi_{app,\ ^1O_2}$ and optical data (continued)									
Reference	Sample ID	Sample Classification	*E2:E3*	SUVA$_{254}$ (L mg C^{-1}•m^{-1})	$S_{290-400}$ / $S_{300-400}$ / $S_{290-450}$ / $S_{295-400}$ (μm^{-1})	$S_{300-600}$ / $S_{300-500}$ / $S_{300-700}$ / $S_{275-500}$ (μm^{-1})	FI	Wavelength Range (nm)	$\Phi_{app,\ ^1O_2}$ (×10^{-2} mol mol-photons^{-1})
Wasswa and Zeng 2024	Lake 194 06/26/2018	Whole Water (Lake)	6.86	2.81	18.41	NA	1.52	290-550	1.97
Wasswa and Zeng 2024	Lake 194 07/09/2018	Whole Water (Lake)	6.61	2.92	18.94	NA	1.46	290-550	1.89
Wasswa and Zeng 2024	Lake 194 07/23/2018	Whole Water (Lake)	8.62	2.19	20.54	NA	1.65	290-550	2.25
Wasswa and Zeng 2024	Lake 194 08/07/2018	Whole Water (Lake)	8.23	2.27	20.80	NA	1.59	290-550	2.27
Wasswa and Zeng 2024	Lake 194 08/20/2018	Whole Water (Lake)	7.81	2.42	19.80	NA	1.65	290-550	2.09
Wasswa and Zeng 2024	Lake 194 09/09/2018	Whole Water (Lake)	8.10	2.40	20.70	NA	1.61	290-550	2.18
Wasswa and Zeng 2024	Lake 194 09/22/2019	Whole Water (Lake)	9.23	2.58	21.45	NA	1.61	290-550	2.47
Wasswa and Zeng 2024	Lake 195 06/03/2019	Whole Water (Lake)	7.34	3.44	14.73	NA	2.39	290-550	1.58
Wasswa and Zeng 2024	Lake 199 06/19/2018	Whole Water (Lake)	6.61	1.93	15.66	NA	1.72	290-550	2.39
Wasswa and Zeng 2024	Lake 199 07/03/2018	Whole Water (Lake)	22.68	0.88	33.97	NA	1.93	290-550	1.62
Wasswa and Zeng 2024	Lake 199 07/18/2018	Whole Water (Lake)	10.67	1.27	21.94	NA	1.63	290-550	4.15
Wasswa and Zeng 2024	Lake 199 07/31/2018	Whole Water (Lake)	6.47	1.40	16.21	NA	1.66	290-550	2.64
Wasswa and Zeng 2024	Lake 199 08/14/2018	Whole Water (Lake)	8.07	1.36	18.69	NA	1.65	290-550	1.49
Wasswa and Zeng 2024	Lake 199 08/28/2018	Whole Water (Lake)	10.12	1.81	23.71	NA	1.62	290-550	1.92
Wasswa and Zeng 2024	Lake 199 09/11/2018	Whole Water (Lake)	10.90	1.26	25.91	NA	1.64	290-550	3.04
Wasswa and Zeng 2024	Lake 199 06/23/2019	Whole Water (Lake)	11.33	0.88	21.06	NA	1.37	290-550	3.43
Wasswa and Zeng 2024	Lake 199 07/08/2019	Whole Water (Lake)	9.58	1.40	21.13	NA	1.50	290-550	1.73
Wasswa and Zeng 2024	Lake 199 07/30/2019	Whole Water (Lake)	7.83	1.47	17.14	NA	1.56	290-550	2.71
Wasswa and Zeng 2024	Lake 199 08/18/2019	Whole Water (Lake)	11.71	1.25	22.02	NA	1.44	290-550	2.29
Wasswa and Zeng 2024	Lake 199 09/15/2019	Whole Water (Lake)	18.84	1.22	32.30	NA	1.62	290-550	3.79
Wasswa and Zeng 2024	Lake 203 06/16/2019	Whole Water (Lake)	6.42	2.66	17.23	NA	1.67	290-550	1.73
Wasswa and Zeng 2024	Lake 203 09/22/2019	Whole Water (Lake)	8.40	2.63	19.48	NA	1.62	290-550	1.98
Wasswa and Zeng 2024	Lake 205 06/08/2019	Whole Water (Lake)	6.08	2.83	17.27	NA	1.50	290-550	1.97
Wasswa and Zeng 2024	Lake 205 08/06/2019	Whole Water (Lake)	7.00	2.86	17.99	NA	1.56	290-550	1.99
Wasswa and Zeng 2024	Lake 209 08/18/2019	Whole Water (Lake)	6.01	2.29	17.71	NA	1.63	290-550	1.80
Wasswa and Zeng 2024	Lake 210 06/02/2019	Whole Water (Lake)	5.57	2.89	16.49	NA	1.53	290-550	1.56
Wasswa and Zeng 2024	Lake 210 08/18/2019	Whole Water (Lake)	5.53	3.17	16.08	NA	1.55	290-550	1.50
Wasswa and Zeng 2024	Lake 212 06/16/2018	Whole Water (Lake)	11.32	1.31	23.37	NA	1.65	290-550	2.12
Wasswa and Zeng 2024	Lake 212 07/14/2018	Whole Water (Lake)	8.86	2.73	19.93	NA	1.58	290-550	2.34
Wasswa and Zeng 2024	Lake 212 07/28/2018	Whole Water (Lake)	8.68	2.73	19.99	NA	1.61	290-550	2.47
Wasswa and Zeng 2024	Lake 212 08/12/2018	Whole Water (Lake)	9.20	2.69	20.84	NA	1.62	290-550	2.14
Wasswa and Zeng 2024	Lake 212 06/17/2019	Whole Water (Lake)	6.35	2.99	16.77	NA	1.60	290-550	2.46
Wasswa and Zeng 2024	Lake 213 08/18/2019	Whole Water (Lake)	7.50	2.29	18.28	NA	1.80	290-550	2.30
Wasswa and Zeng 2024	Lake 215 06/09/2019	Whole Water (Lake)	5.52	2.83	16.76	NA	1.43	290-550	2.67
Wasswa and Zeng 2024	Lake 215 09/15/2019	Whole Water (Lake)	5.84	2.96	16.83	NA	1.47	290-550	2.36
Wasswa and Zeng 2024	Lake 223 08/19/2019	Whole Water (Lake)	8.45	3.02	19.52	NA	1.60	290-550	2.48
Wasswa and Zeng 2024	Lake 225 07/28/2019	Whole Water (Lake)	6.55	2.93	17.66	NA	1.52	290-550	2.03
Wasswa and Zeng 2024	Lake 229 06/29/2019	Whole Water (Lake)	7.08	2.24	18.66	NA	1.58	290-550	1.93
Wasswa and Zeng 2024	Lake 230 06/08/2019	Whole Water (Lake)	6.84	2.78	19.58	NA	1.61	290-550	2.26
Wasswa and Zeng 2024	Lake 231 06/04/2019	Whole Water (Lake)	7.59	2.86	18.80	NA	1.58	290-550	1.93
Wasswa and Zeng 2024	Lake 234 06/20/2018	Whole Water (Lake)	10.47	1.88	22.50	NA	1.70	290-550	2.91
Wasswa and Zeng 2024	Lake 234 07/15/2018	Whole Water (Lake)	10.62	1.81	23.45	NA	1.85	290-550	3.11
Wasswa and Zeng 2024	Lake 234 06/09/2019	Whole Water (Lake)	6.35	2.09	17.99	NA	1.89	290-550	1.84
Wasswa and Zeng 2024	Lake 234 08/12/2019	Whole Water (Lake)	7.71	1.97	18.19	NA	1.70	290-550	1.93
Wasswa and Zeng 2024	Lake 235 07/14/2019	Whole Water (Lake)	6.21	3.18	17.64	NA	1.48	290-550	1.76
Wasswa and Zeng 2024	Lake 236 07/14/2019	Whole Water (Lake)	8.62	2.11	22.57	NA	1.53	290-550	2.86
Wasswa and Zeng 2024	Lake 236 09/15/2019	Whole Water (Lake)	7.22	2.13	17.65	NA	1.62	290-550	2.08
Wasswa and Zeng 2024	Lake 238 08/12/2019	Whole Water (Lake)	7.10	3.21	18.66	NA	1.54	290-550	2.53
Wasswa and Zeng 2024	Lake 239 06/09/2019	Whole Water (Lake)	7.15	2.44	19.23	NA	1.68	290-550	2.19
Wasswa and Zeng 2024	Lake 239 08/18/2019	Whole Water (Lake)	8.16	2.65	18.77	NA	1.76	290-550	2.28
Wasswa and Zeng 2024	Lake 245 09/22/2019	Whole Water (Lake)	8.16	2.12	19.18	NA	1.63	290-550	2.09

**Table S18.** Summary of $\Phi_{app,\,^1O_2}$ and optical data (continued)									
Reference	Sample ID	Sample Classification	*E2:E3*	SUVA$_{254}$ (L mg C^{-1}•m^{-1})	$S_{290–400}$ / $S_{300–400}$ / $S_{290–450}$ / $S_{295–400}$ (μm^{-1})	$S_{300–600}$ / $S_{300–500}$ / $S_{300–700}$ / $S_{275–500}$ (μm^{-1})	FI	Wavelength Range (nm)	$\Phi_{app,\,^1O_2}$ (×10^{-2} mol mol-photons^{-1})
Wasswa and Zeng 2024	Lake 246 08/05/2019	Whole Water (Lake)	5.94	3.43	16.82	NA	1.64	290-550	2.03
Wasswa and Zeng 2024	Lake 247 06/08/2019	Whole Water (Lake)	6.62	2.55	18.20	NA	1.54	290-550	1.95
Wasswa and Zeng 2024	Lake 247 09/20/2019	Whole Water (Lake)	8.19	2.55	20.39	NA	1.58	290-550	2.27
Wasswa and Zeng 2024	Lake 248 08/18/2019	Whole Water (Lake)	5.86	3.30	16.35	NA	1.59	290-550	1.91
Wasswa and Zeng 2024	Lake 249 07/08/2019	Whole Water (Lake)	7.40	2.22	18.82	NA	1.76	290-550	1.91
Wasswa and Zeng 2024	Lake 249 09/01/2019	Whole Water (Lake)	7.00	2.46	17.91	NA	1.71	290-550	1.95
Wasswa and Zeng 2024	Lake 250 07/08/2019	Whole Water (Lake)	8.01	2.06	21.51	NA	1.61	290-550	2.35
Wasswa and Zeng 2024	Lake 250 08/18/2019	Whole Water (Lake)	7.77	2.13	18.05	NA	1.72	290-550	1.85
Wasswa and Zeng 2024	Lake 251 08/27/2019	Whole Water (Lake)	7.31	3.28	18.66	NA	1.73	290-550	2.42
Wasswa and Zeng 2024	Lake 251 09/17/2019	Whole Water (Lake)	6.66	3.37	16.62	NA	1.74	290-550	2.48
Wasswa and Zeng 2024	Lake 253 07/02/2019	Whole Water (Lake)	14.79	1.72	27.24	NA	1.64	290-550	2.40
Wasswa and Zeng 2024	Lake 253 08/25/2019	Whole Water (Lake)	9.85	1.75	19.06	NA	1.67	290-550	3.44
Wasswa and Zeng 2024	Lake 253 09/15/2019	Whole Water (Lake)	16.22	1.64	27.28	NA	1.70	290-550	1.98
Wasswa and Zeng 2024	Lake 256 07/05/2018	Whole Water (Lake)	10.68	1.82	23.04	NA	1.72	290-550	2.97
Wasswa and Zeng 2024	Lake 256 07/16/2018	Whole Water (Lake)	10.03	1.77	20.70	NA	1.64	290-550	2.85
Wasswa and Zeng 2024	Lake 256 07/31/2018	Whole Water (Lake)	10.59	1.82	21.86	NA	1.68	290-550	2.22
Wasswa and Zeng 2024	Lake 256 08/15/2018	Whole Water (Lake)	8.68	1.83	20.56	NA	1.71	290-550	2.67
Wasswa and Zeng 2024	Lake 256 08/29/2018	Whole Water (Lake)	6.66	2.51	18.38	NA	1.46	290-550	2.58
Wasswa and Zeng 2024	Lake 256 09/14/2018	Whole Water (Lake)	9.97	1.86	23.25	NA	1.74	290-550	2.35
Wasswa and Zeng 2024	Lake 256 09/28/2018	Whole Water (Lake)	11.22	1.77	26.77	NA	1.70	290-550	2.34
Wasswa and Zeng 2024	Lake 256 06/18/2019	Whole Water (Lake)	8.13	1.83	20.04	NA	1.68	290-550	1.60
Wasswa and Zeng 2024	Lake 256 06/18/2019	Whole Water (Lake)	6.35	1.91	16.55	NA	1.66	290-550	2.21
Wasswa and Zeng 2024	Lake 256 08/13/2019	Whole Water (Lake)	9.46	1.82	20.64	NA	1.69	290-550	2.10
Wasswa and Zeng 2024	Lake 257 08/24/2019	Whole Water (Lake)	6.05	3.83	17.43	NA	1.58	290-550	2.24
Wasswa and Zeng 2024	Lake 258 06/16/2019	Whole Water (Lake)	6.79	2.63	17.65	NA	1.58	290-550	2.39
Wasswa and Zeng 2024	Lake 258 08/11/2019	Whole Water (Lake)	5.81	2.81	15.56	NA	1.59	290-550	2.19
Wasswa and Zeng 2024	Lake 259 09/01/2019	Whole Water (Lake)	6.60	3.46	17.97	NA	1.60	290-550	3.63
Wasswa and Zeng 2024	Lake 260 06/01/2019	Whole Water (Lake)	5.58	2.87	17.26	NA	1.53	290-550	1.72
Wasswa and Zeng 2024	Lake 260 08/12/2019	Whole Water (Lake)	6.81	2.99	18.29	NA	1.59	290-550	2.10
Wasswa and Zeng 2024	Lake 261 06/17/2019	Whole Water (Lake)	6.50	2.79	18.18	NA	1.59	290-550	2.31
Wasswa and Zeng 2024	Lake 261 08/12/2019	Whole Water (Lake)	7.32	2.72	18.08	NA	1.61	290-550	2.14
Wasswa and Zeng 2024	Lake 262 06/17/2019	Whole Water (Lake)	5.31	3.55	16.57	NA	1.40	290-550	1.81
Wasswa and Zeng 2024	Lake 262 09/21/2019	Whole Water (Lake)	6.17	3.39	17.97	NA	1.45	290-550	1.92
Wasswa and Zeng 2024	Lake 261 09/04/2021_Lysate A	Bloom Lysate	2.93	0.68	7.46	NA	2.24	290-550	0.92
Wasswa and Zeng 2024	Lake 238 09/07/2021_Lysate B	Bloom Lysate	2.88	0.65	7.37	NA	2.24	290-550	0.93
Wasswa and Zeng 2024	Lake 147 08/30/2021_Lysate C	Bloom Lysate	2.72	0.67	7.16	NA	2.14	290-550	1.00
Wasswa and Zeng 2024	Lake 138 09/07/2021_Lysate D	Bloom Lysate	2.98	0.57	8.15	NA	2.20	290-550	0.99
Wasswa and Zeng 2024	Lake 33 09/04/2021_Lysate E	Bloom Lysate	2.76	0.65	6.65	NA	2.49	290-550	0.84
Wasswa and Zeng 2024	Lake 37 08/20/2021_Lysate F	Bloom Lysate	2.91	0.50	7.61	NA	2.11	290-550	0.82
Wasswa and Zeng 2024	Lake 38 08/29/2021_Lysate G	Bloom Lysate	2.91	0.73	7.98	NA	2.12	290-550	1.19
Wasswa and Zeng 2024	Lake 40 09/16/2021_Lysate H	Bloom Lysate	2.75	0.61	7.16	NA	2.12	290-550	1.23
Wasswa and Zeng 2024	Lake 78 08/22/2021_ Lysate I	Bloom Lysate	2.85	0.54	7.75	NA	2.23	290-550	1.14
Wasswa and Zeng 2024	Lake 82 08/17/2021_Lysate J	Bloom Lysate	2.80	0.46	7.61	NA	2.37	290-550	1.30
Wasswa and Zeng 2024	Lake 93 08/29/2021_Lysate K	Bloom Lysate	3.01	0.46	8.34	NA	2.15	290-550	0.88
Wasswa and Zeng 2024	Lake 221 09/06/2021_Lysate L	Bloom Lysate	2.84	0.68	7.38	NA	2.14	290-550	1.35
Wasswa and Zeng 2024	Lake 256 10/12/2021_Otisco	Whole Water (Lake)	8.20	1.61	18.81	NA	1.75	290-550	1.72
Wasswa and Zeng 2024	Lysate A 25% + Otisco 75%	Whole Water (Lake, Bloom Lysate)	6.58	1.26	15.53	NA	1.86	290-550	1.25
Wasswa and Zeng 2024	Lysate A 50% + Otisco 50%	Whole Water (Lake, Bloom Lysate)	5.16	1.06	12.53	NA	2.00	290-550	1.03
Wasswa and Zeng 2024	Lysate A 75% + Otisco 25%	Whole Water (Lake, Bloom Lysate)	3.89	0.86	9.62	NA	2.20	290-550	0.88
Wasswa and Zeng 2024	Lysate B 25% + Otisco 75%	Whole Water (Lake, Bloom Lysate)	6.95	1.22	16.46	NA	1.78	290-550	1.29
Wasswa and Zeng 2024	Lysate B 50% + Otisco 50%	Whole Water (Lake, Bloom Lysate)	5.65	0.99	13.92	NA	1.82	290-550	1.07

Table S18. Summary of $\Phi_{app,\ ^1O_2}$ and optical data (continued)

Reference	Sample ID	Sample Classification	*E2:E3*	SUVA$_{254}$ (L mg C^{-1}•m^{-1})	$S_{290–400}$ / $S_{300–400}$ / $S_{290–450}$ / $S_{295–400}$ (μm^{-1})	$S_{300–600}$ / $S_{300–500}$ / $S_{300–700}$ / $S_{275–500}$ (μm^{-1})	FI	Wavelength Range (nm)	$\Phi_{app,\ ^1O_2}$ (×10^{-2} mol mol-photons^{-1})
Wasswa and Zeng 2024	Lysate B 75% + Otisco 25%	Whole Water (Lake, Bloom Lysate)	4.31	0.75	11.06	NA	1.89	290-550	0.89
Wasswa and Zeng 2024	Lysate C 25% + Otisco 75%	Whole Water (Lake, Bloom Lysate)	6.66	1.26	15.82	NA	1.81	290-550	1.31
Wasswa and Zeng 2024	Lysate C 50% + Otisco 50%	Whole Water (Lake, Bloom Lysate)	5.29	1.07	13.02	NA	1.90	290-550	1.11
Wasswa and Zeng 2024	Lysate C 75% + Otisco 25%	Whole Water (Lake, Bloom Lysate)	4.05	0.88	10.28	NA	2.02	290-550	0.97
Wasswa and Zeng 2024	Lysate D 25% + Otisco 75%	Whole Water (Lake, Bloom Lysate)	6.65	1.26	15.81	NA	1.79	290-550	1.37
Wasswa and Zeng 2024	Lysate D 50% + Otisco 50%	Whole Water (Lake, Bloom Lysate)	5.27	1.05	12.99	NA	1.86	290-550	1.18
Wasswa and Zeng 2024	Lysate D 75% + Otisco 25%	Whole Water (Lake, Bloom Lysate)	4.01	0.85	10.23	NA	1.97	290-550	1.05
Wasswa and Zeng 2024	Lysate E 25% + Otisco 75%	Whole Water (Lake, Bloom Lysate)	6.51	1.27	15.64	NA	1.78	290-550	1.31
Wasswa and Zeng 2024	Lysate E 50% + Otisco 50%	Whole Water (Lake, Bloom Lysate)	5.07	1.07	12.76	NA	1.83	290-550	1.11
Wasswa and Zeng 2024	Lysate E 75% + Otisco 25%	Whole Water (Lake, Bloom Lysate)	3.82	0.87	9.98	NA	1.91	290-550	0.97
Wasswa and Zeng 2024	Lysate F 25% + Otisco 75%	Whole Water (Lake, Bloom Lysate)	6.85	1.24	16.31	NA	1.78	290-550	1.37
Wasswa and Zeng 2024	Lysate F 50% + Otisco 50%	Whole Water (Lake, Bloom Lysate)	5.53	1.02	13.77	NA	1.83	290-550	1.18
Wasswa and Zeng 2024	Lysate F 75% + Otisco 25%	Whole Water (Lake, Bloom Lysate)	4.24	0.80	11.10	NA	1.92	290-550	1.04
Wasswa and Zeng 2024	SRNOM	IHSS Isolate (SRNOM)	4.69	3.97	15.47	NA	1.37	290-550	2.57
Wasswa and Zeng 2024	Lysate A 25% + SRNOM 75%	IHSS Isolate (SRNOM, Bloom	4.53	3.15	14.66	NA	1.57	290-550	1.90
Wasswa and Zeng 2024	Lysate A 50% + SRNOM 50%	IHSS Isolate (SRNOM, Bloom	4.27	2.31	13.44	NA	1.79	290-550	1.46
Wasswa and Zeng 2024	Lysate A 75% + SRNOM 25%	IHSS Isolate (SRNOM, Bloom	3.81	1.49	11.34	NA	1.71	290-550	1.05
Wasswa and Zeng 2024	Lysate B 25% + SRNOM 75%	IHSS Isolate (SRNOM, Bloom	4.58	3.08	14.94	NA	1.41	290-550	1.93
Wasswa and Zeng 2024	Lysate B 50% + SRNOM 50%	IHSS Isolate (SRNOM, Bloom	4.39	2.23	14.08	NA	1.47	290-550	1.44
Wasswa and Zeng 2024	Lysate B 75% + SRNOM 25%	IHSS Isolate (SRNOM, Bloom	4.02	1.38	12.41	NA	1.60	290-550	1.02
Wasswa and Zeng 2024	Lysate C 25% + SRNOM 75%	IHSS Isolate (SRNOM, Bloom	4.54	3.16	14.76	NA	1.45	290-550	1.90
Wasswa and Zeng 2024	Lysate C 50% + SRNOM 50%	IHSS Isolate (SRNOM, Bloom	4.31	2.32	13.67	NA	1.57	290-550	1.55
Wasswa and Zeng 2024	Lysate C 75% + SRNOM 25%	IHSS Isolate (SRNOM, Bloom	3.90	1.50	11.77	NA	1.77	290-550	1.15
Wasswa and Zeng 2024	Lysate D 25% + SRNOM 75%	IHSS Isolate (SRNOM, Bloom	4.54	3.12	14.75	NA	1.42	290-550	1.96
Wasswa and Zeng 2024	Lysate D 50% + SRNOM 50%	IHSS Isolate (SRNOM, Bloom	4.31	2.32	13.65	NA	1.50	290-550	1.57
Wasswa and Zeng 2024	Lysate D 75% + SRNOM 25%	IHSS Isolate (SRNOM, Bloom	3.88	1.49	11.74	NA	1.68	290-550	1.18
Wasswa and Zeng 2024	Lysate E 25% + SRNOM 75%	IHSS Isolate (SRNOM, Bloom	4.51	3.14	14.70	NA	1.41	290-550	1.90
Wasswa and Zeng 2024	Lysate E 50% + SRNOM 50%	IHSS Isolate (SRNOM, Bloom	4.24	2.31	13.53	NA	1.48	290-550	1.44
Wasswa and Zeng 2024	Lysate E 75% + SRNOM 25%	IHSS Isolate (SRNOM, Bloom	3.77	1.48	11.54	NA	1.62	290-550	1.07
Wasswa and Zeng 2024	Lysate F 25% + SRNOM 75%	IHSS Isolate (SRNOM, Bloom	4.57	3.13	14.91	NA	1.41	290-550	1.98
Wasswa and Zeng 2024	Lysate F 50% + SRNOM 50%	IHSS Isolate (SRNOM, Bloom	4.37	2.29	14.01	NA	1.47	290-550	1.53
Wasswa and Zeng 2024	Lysate F 75% + SRNOM 25%	IHSS Isolate (SRNOM, Bloom	3.99	1.42	12.36	NA	1.61	290-550	1.15
Wasswa and Zeng 2024	Lake 261 09/04/2021_Supernatant A_1	Bloom Supernatant	5.10	1.88	13.65	NA	1.98	290-550	2.09
Wasswa and Zeng 2024	Lake 261 09/04/2021_Supernatant A_2	Bloom Supernatant	4.90	1.92	13.30	NA	2.01	290-550	2.45
Wasswa and Zeng 2024	Lake 261 09/04/2021_Supernatant A_3	Bloom Supernatant	4.62	1.98	12.79	NA	2.04	290-550	2.68
Wasswa and Zeng 2024	Lake 261 09/04/2021_Supernatant A_4	Bloom Supernatant	4.07	2.10	11.68	NA	2.12	290-550	2.89
Wasswa and Zeng 2024	Lake 261 09/04/2021_Supernatant A_5	Bloom Supernatant	3.62	2.18	10.66	NA	2.14	290-550	3.05
Wasswa and Zeng 2024	Lake 261 09/04/2021_Supernatant A_6	Bloom Supernatant	3.34	2.21	9.96	NA	2.19	290-550	3.13
Wasswa and Zeng 2024	Lake 261 09/04/2021_Supernatant A_7	Bloom Supernatant	3.25	2.22	9.74	NA	2.20	290-550	3.16
Wasswa and Zeng 2024	Lake 261 09/04/2021_Supernatant A_8	Bloom Supernatant	3.23	2.21	9.68	NA	2.20	290-550	3.17
Wasswa and Zeng 2024	Lake 261 09/04/2021_Supernatant A_9	Bloom Supernatant	3.20	2.22	9.61	NA	2.24	290-550	3.17
Wasswa and Zeng 2024	Lake 238 09/07/2021_Supernatant B_1	Bloom Supernatant	5.75	1.93	14.68	NA	1.94	290-550	2.13
Wasswa and Zeng 2024	Lake 238 09/07/2021_Supernatant B_2	Bloom Supernatant	5.63	1.95	14.49	NA	2.00	290-550	2.35
Wasswa and Zeng 2024	Lake 238 09/07/2021_Supernatant B_3	Bloom Supernatant	5.26	2.01	13.90	NA	2.02	290-550	2.55
Wasswa and Zeng 2024	Lake 238 09/07/2021_Supernatant B_4	Bloom Supernatant	4.71	2.10	12.94	NA	2.10	290-550	2.73
Wasswa and Zeng 2024	Lake 238 09/07/2021_Supernatant B_5	Bloom Supernatant	4.26	2.16	12.06	NA	2.12	290-550	2.88
Wasswa and Zeng 2024	Lake 238 09/07/2021_Supernatant B_6	Bloom Supernatant	3.95	2.18	11.40	NA	2.18	290-550	3.02
Wasswa and Zeng 2024	Lake 238 09/07/2021_Supernatant B_7	Bloom Supernatant	3.76	2.18	10.99	NA	2.18	290-550	3.05
Wasswa and Zeng 2024	Lake 238 09/07/2021_Supernatant B_8	Bloom Supernatant	3.74	2.19	10.93	NA	2.19	290-550	3.07
Wasswa and Zeng 2024	Lake 238 09/07/2021_Supernatant B_9	Bloom Supernatant	3.68	2.19	10.80	NA	2.22	290-550	3.07
Wasswa and Zeng 2024	Lake 147 08/30/2021_Supernatant C_1	Bloom Supernatant	5.35	1.93	13.11	NA	1.94	290-550	2.15

Table S18. Summary of $\Phi_{app, {}^1O_2}$ and optical data (continued)									
Reference	Sample ID	Sample Classification	*E2:E3*	$SUVA_{254}$ (L mg C^{-1}•m^{-1})	$S_{290–400}$ / $S_{300–400}$ / $S_{290–450}$ / $S_{295–400}$ (μm^{-1})	$S_{300–600}$ / $S_{300–500}$ / $S_{300–700}$ / $S_{275–500}$ (μm^{-1})	FI	Wavelength Range (nm)	$\Phi_{app, {}^1O_2}$ (×10^{-2} mol mol-$photons^{-1}$)
Wasswa and Zeng 2024	Lake 147 08/30/2021_Supernatant C_2	Bloom Supernatant	5.18	1.96	12.82	NA	1.97	290-550	2.62
Wasswa and Zeng 2024	Lake 147 08/30/2021_Supernatant C_3	Bloom Supernatant	4.89	2.03	12.33	NA	1.99	290-550	2.87
Wasswa and Zeng 2024	Lake 147 08/30/2021_Supernatant C_4	Bloom Supernatant	4.50	2.10	11.61	NA	2.07	290-550	3.09
Wasswa and Zeng 2024	Lake 147 08/30/2021_Supernatant C_5	Bloom Supernatant	4.21	2.16	11.02	NA	2.09	290-550	3.19
Wasswa and Zeng 2024	Lake 147 08/30/2021_Supernatant C_6	Bloom Supernatant	4.03	2.19	10.65	NA	2.15	290-550	3.26
Wasswa and Zeng 2024	Lake 147 08/30/2021_Supernatant C_7	Bloom Supernatant	3.95	2.20	10.47	NA	2.15	290-550	3.29
Wasswa and Zeng 2024	Lake 147 08/30/2021_Supernatant C_8	Bloom Supernatant	3.94	2.21	10.44	NA	2.16	290-550	3.31
Wasswa and Zeng 2024	Lake 147 08/30/2021_Supernatant C_9	Bloom Supernatant	3.92	2.21	10.41	NA	2.19	290-550	3.31
Wasswa and Zeng 2024	Lake 138 09/07/2021_Supernatant D_1	Bloom Supernatant	5.55	1.90	13.35	NA	1.87	290-550	2.11
Wasswa and Zeng 2024	Lake 138 09/07/2021_Supernatant D_2	Bloom Supernatant	5.37	1.93	13.05	NA	1.90	290-550	2.76
Wasswa and Zeng 2024	Lake 138 09/07/2021_Supernatant D_3	Bloom Supernatant	5.04	1.98	12.51	NA	1.92	290-550	3.02
Wasswa and Zeng 2024	Lake 138 09/07/2021_Supernatant D_4	Bloom Supernatant	4.69	2.04	11.87	NA	2.00	290-550	3.17
Wasswa and Zeng 2024	Lake 138 09/07/2021_Supernatant D_5	Bloom Supernatant	4.41	2.07	11.34	NA	2.02	290-550	3.27
Wasswa and Zeng 2024	Lake 138 09/07/2021_Supernatant D_6	Bloom Supernatant	4.31	2.08	11.13	NA	2.04	290-550	3.32
Wasswa and Zeng 2024	Lake 138 09/07/2021_Supernatant D_7	Bloom Supernatant	4.25	2.09	11.01	NA	2.06	290-550	3.35
Wasswa and Zeng 2024	Lake 138 09/07/2021_Supernatant D_8	Bloom Supernatant	4.23	2.09	10.98	NA	2.08	290-550	3.37
Wasswa and Zeng 2024	Lake 138 09/07/2021_Supernatant D_9	Bloom Supernatant	4.22	2.09	10.96	NA	2.12	290-550	3.38
Wasswa and Zeng 2024	Lake 33 09/04/2021_Supernatant E	Bloom Supernatant	3.96	2.24	13.12	NA	1.95	290-550	3.16
Wasswa and Zeng 2024	Lake 37 08/20/2021_Supernatant F	Bloom Supernatant	4.12	2.08	11.57	NA	1.88	290-550	2.74
Wasswa and Zeng 2024	Lake 38 08/29/2021_Supernatant G	Bloom Supernatant	3.93	2.24	9.41	NA	1.92	290-550	3.52
Wasswa and Zeng 2024	Lake 40 09/16/2021_Supernatant H	Bloom Supernatant	3.89	2.23	11.07	NA	1.88	290-550	3.90
Wasswa and Zeng 2024	Lake 78 08/22/2021_Supernatant I	Bloom Supernatant	4.16	2.34	11.67	NA	1.92	290-550	3.60
Wasswa and Zeng 2024	Lake 82 08/17/2021_Supernatant J	Bloom Supernatant	4.01	2.27	10.56	NA	1.94	290-550	4.19
Wasswa and Zeng 2024	Lake 93 08/29/2021_Supernatant K	Bloom Supernatant	4.06	2.14	13.32	NA	1.90	290-550	2.84
Wasswa and Zeng 2024	Lake 221 09/06/2021_Supernatant L	Bloom Supernatant	3.78	2.45	10.06	NA	1.94	290-550	4.88
Wasswa and Zeng 2024	Lake 256 10/12/2021_Otisco_1-1	Whole Water (Lake)	7.01	1.78	17.45	NA	1.96	290-550	1.39
Wasswa and Zeng 2024	Lake 256 10/12/2021_Otisco_1-2	Whole Water (Lake)	7.10	1.77	17.56	NA	1.95	290-550	1.38
Wasswa and Zeng 2024	Lake 256 10/12/2021_Otisco_1-3	Whole Water (Lake)	7.30	1.74	17.80	NA	1.88	290-550	1.38
Wasswa and Zeng 2024	Lake 256 10/12/2021_Otisco_1-4	Whole Water (Lake)	7.50	1.72	18.03	NA	1.88	290-550	1.38
Wasswa and Zeng 2024	Lake 256 10/12/2021_Otisco_1-5	Whole Water (Lake)	7.75	1.69	18.32	NA	1.86	290-550	1.37
Wasswa and Zeng 2024	Lake 256 10/12/2021_Otisco_1-6	Whole Water (Lake)	7.91	1.67	18.50	NA	1.86	290-550	1.36
Wasswa and Zeng 2024	Lake 256 10/12/2021_Otisco_1-7	Whole Water (Lake)	8.01	1.65	18.61	NA	1.84	290-550	1.36
Wasswa and Zeng 2024	Lake 256 10/12/2021_Otisco_1-8	Whole Water (Lake)	8.11	1.64	18.72	NA	1.77	290-550	1.35
Wasswa and Zeng 2024	Lake 256 10/12/2021_Otisco_1-9	Whole Water (Lake)	8.19	1.63	18.80	NA	1.75	290-550	1.35
Wasswa and Zeng 2024	Lake 256 10/12/2021_Otisco_2-1	Whole Water (Lake)	6.93	1.75	17.35	NA	1.96	290-550	1.39
Wasswa and Zeng 2024	Lake 256 10/12/2021_Otisco_2-2	Whole Water (Lake)	7.02	1.74	17.45	NA	1.95	290-550	1.39
Wasswa and Zeng 2024	Lake 256 10/12/2021_Otisco_2-3	Whole Water (Lake)	7.21	1.71	17.70	NA	1.88	290-550	1.38
Wasswa and Zeng 2024	Lake 256 10/12/2021_Otisco_2-4	Whole Water (Lake)	7.43	1.69	17.95	NA	1.88	290-550	1.38
Wasswa and Zeng 2024	Lake 256 10/12/2021_Otisco_2-5	Whole Water (Lake)	7.65	1.66	18.20	NA	1.86	290-550	1.37
Wasswa and Zeng 2024	Lake 256 10/12/2021_Otisco_2-6	Whole Water (Lake)	7.85	1.64	18.43	NA	1.86	290-550	1.37
Wasswa and Zeng 2024	Lake 256 10/12/2021_Otisco_2-7	Whole Water (Lake)	8.01	1.61	18.61	NA	1.84	290-550	1.36
Wasswa and Zeng 2024	Lake 256 10/12/2021_Otisco_2-8	Whole Water (Lake)	8.15	1.60	18.75	NA	1.77	290-550	1.36
Wasswa and Zeng 2024	Lake 256 10/12/2021_Otisco_2-9	Whole Water (Lake)	8.22	1.59	18.83	NA	1.75	290-550	1.35
Xie and Wang 2024 [212]	CM200	DBC (pyDOM)	NA	0.31	NA	NA	NA	320-400	0.32
Xie and Wang 2024	CM230	DBC (pyDOM)	NA	0.16	NA	NA	NA	320-400	0.59
Xie and Wang 2024	CM260	DBC (pyDOM)	NA	0.20	NA	NA	NA	320-400	0.86
Xie and Wang 2024	SM200	DBC (pyDOM)	NA	0.36	NA	NA	NA	320-400	0.02
Xie and Wang 2024	SM230	DBC (pyDOM)	NA	0.71	NA	NA	NA	320-400	0.05
Xie and Wang 2024	SM260	DBC (pyDOM)	NA	0.99	NA	NA	NA	320-400	0.06
Xie and Wang 2024 [213]	DBC200	DBC (pyDOM)	2.74	1.47	NA	NA	2.98	320-400	0.21
Xie and Wang 2024	DBC300	DBC (pyDOM)	4.01	2.53	NA	NA	2.25	320-400	0.21

**Table S18.** Summary of $\Phi_{app,\ ^1O_2}$ and optical data (continued)									
Reference	Sample ID	Sample Classification	*E2:E3*	$SUVA_{254}$ (L mg C^{-1}•m^{-1})	$S_{290–400}$ / $S_{300–400}$ / $S_{290–450}$ / $S_{295–400}$ (μm^{-1})	$S_{300–600}$ / $S_{300–500}$ / $S_{300–700}$ / $S_{275–500}$ (μm^{-1})	FI	Wavelength Range (nm)	$\Phi_{app,\ ^1O_2}$ ($\times 10^{-2}$ mol mol-photons^{-1})
Xie and Wang 2024	DBC400	DBC (pyDOM)	4.33	4.47	NA	NA	2.01	320-400	0.61
Xie and Wang 2024	DBC500	DBC (pyDOM)	7.07	3.55	NA	NA	1.50	320-400	1.77
Wan and Bi 2025 [214]	EOM	C18+PPL Extract (M. aeruginosa)	3.51	1.73	NA	NA	NA	365	0.88
Wan and Bi 2025	EOM + Cl1 (FAC/TOC=1.0)	C18+PPL Extract (M. aeruginosa, Cl2)	8.31	1.15	NA	NA	NA	365	2.45
Wan and Bi 2025	EOM + Cl2 (FAC/TOC=2.0)	C18+PPL Extract (M. aeruginosa, Cl2)	13.34	0.98	NA	NA	NA	365	1.41
Wan and Bi 2025	IOM	Lyophilized (M. aeruginosa)	3.32	2.21	NA	NA	NA	365	0.20
Wan and Bi 2025	IOM + Cl1 (FAC/TOC=1.0)	Lyophilized (M. aeruginosa, Cl2)	5.86	1.44	NA	NA	NA	365	0.79
Wan and Bi 2025	IOM + Cl2 (FAC/TOC=2.0)	Lyophilized (M. aeruginosa, Cl2)	7.49	1.04	NA	NA	NA	365	1.47
Wan and Bi 2025	NOM	IHSS Isolate (SRNOM)	4.70	4.58	NA	NA	NA	365	0.79
Wan and Bi 2025	NOM + Cl1 (FAC/TOC=1.0)	IHSS Isolate (SRNOM, Cl2)	6.68	3.35	NA	NA	NA	365	1.58
Wan and Bi 2025	NOM + Cl2 (FAC/TOC=2.0)	IHSS Isolate (SRNOM, Cl2)	9.37	2.65	NA	NA	NA	365	2.89
Zhang and Zhou 2025 [215]	PS MPs-DOM	WEOM (Microplastic)	3.94	NA	NA	NA	1.48	300-400	0.16
Zhang and Zhou 2025	PVC MPs-DOM	WEOM (Microplastic)	2.29	NA	NA	NA	1.91	300-400	0.35
Luo and Zhang 2025 [216]	sDBC_Before sorption on ferrihydrite	DBC (pyDOM)	NA	NA	NA	NA	NA	320-400	1.14
Luo and Zhang 2025	sDBC_After sorption on ferrihydrite	DBC (pyDOM, Ferrihydrite)	NA	NA	NA	NA	NA	320-400	0.98
Luo and Zhang 2025	rDBC_Before sorption on ferrihydrite	DBC (pyDOM)	NA	NA	NA	NA	NA	320-400	1.35
Luo and Zhang 2025	rDBC_After sorption on ferrihydrite	DBC (pyDOM, Ferrihydrite)	NA	NA	NA	NA	NA	320-400	1.07
Luo and Zhang 2025	mDBC_Before sorption on ferrihydrite	DBC (pyDOM)	NA	NA	NA	NA	NA	320-400	0.57
Luo and Zhang 2025	mDBC_After sorption on ferrihydrite	DBC (pyDOM, Ferrihydrite)	NA	NA	NA	NA	NA	320-400	0.54

References

1. Dulin, D.; Mill, T., Development and evaluation of sunlight actinometers. *Environmental Science & Technology* **1982,** *16*, (11), 815-820.
2. Laszakovits, J. R.; Berg, S. M.; Anderson, B. G.; O'Brien, J. E.; Wammer, K. H.; Sharpless, C. M., *p*-Nitroanisole/pyridine and *p*-nitroacetophenone/pyridine actinometers revisited: Quantum yield in comparison to ferrioxalate. *Environmental Science & Technology Letters* **2017,** *4*, (1), 11-14.
3. Deyette, N. A.; Lawrence, G. B.; Wall, G. R. *Anions - Ion Chromatography - SOP*; U.S. Geological Survey, New York Water Science Center, Soil and Low-Ionic-Strength Water Quality Laboratory: Troy, NY, 2022.
4. Ingleston, H. H.; Lawrence, G. B.; Deyette, N. A.; Wall, G. R. *Organic Carbon Analysis (TOC) (DOC) - SOP*; U.S. Geological Survey, New York Water Science Center, Soil and Low-Ionic-Strength Water Quality Laboratory: Troy, NY, 2022.
5. Ingleston, H. H.; Lawrence, G. B.; Deyette, N. A.; Wall, G. R. *Dissolved Inorganic Carbon Analysis (DIC) - SOP*; U.S. Geological Survey, New York Water Science Center, Soil and Low-Ionic-Strength Water Quality Laboratory: Troy, NY, 2022.
6. Ingleston, H. H.; Lawrence, G. B.; Deyette, N. A.; Wall, G. R. *pH, ANC, Cond - Electronic Measurements and Titration - SOP*; U.S. Geological Survey, New York Water Science Center, Soil and Low-Ionic-Strength Water Quality Laboratory: Troy, NY, 2022.
7. Lincoln, T. A.; Lawrence, G. B.; Deyette, N. A.; Wall, G. R. *Inductively Coupled Plasma – Optical Emission Spectrometry - SOP*; U.S. Geological Survey, New York Water Science Center, Soil and Low-Ionic-Strength Water Quality Laboratory: Troy, NY, 2022.
8. Lincoln, T. A.; Lawrence, G. B.; Deyette, N. A.; Wall, G. R. *Speciated Aluminum - SOP*; U.S. Geological Survey, New York Water Science Center, Soil and Low-Ionic-Strength Water Quality Laboratory: Troy, NY, 2022.
9. Cory, R. M.; Miller, M. P.; McKnight, D. M.; Guerard, J. J.; Miller, P. L., Effect of instrument-specific response on the analysis of fulvic acid fluorescence spectra. *Limnology and Oceanography: Methods* **2010,** *8*, (2), 67-78.
10. Ohno, T., Fluorescence inner-filtering correction for determining the humification index of dissolved organic matter. *Environmental Science & Technology* **2002,** *36*, (4), 742-746.
11. Kothawala, D. N.; Murphy, K. R.; Stedmon, C. A.; Weyhenmeyer, G. A.; Tranvik, L. J., Inner filter correction of dissolved organic matter fluorescence. *Limnology and Oceanography: Methods* **2013,** *11*, (12), 616-630.
12. Lawaetz, A. J.; Stedmon, C. A., Fluorescence intensity calibration using the Raman scatter peak of water. *Applied Spectroscopy* **2009,** *63*, (8), 936-940.
13. Murphy, K. R.; Stedmon, C. A.; Graeber, D.; Bro, R., Fluorescence spectroscopy and multi-way techniques. PARAFAC. *Analytical Methods* **2013,** *5*, (23), 6557-6566.
14. Cuthbert, I. D.; del Giorgio, P., Toward a standard method of measuring color in freshwater. *Limnology and Oceanography* **1992,** *37*, (6), 1319-1326.
15. Weishaar, J. L.; Aiken, G. R.; Bergamaschi, B. A.; Fram, M. S.; Fujii, R.; Mopper, K., Evaluation of specific ultraviolet absorbance as an indicator of the chemical composition and reactivity of dissolved organic carbon. *Environmental Science & Technology* **2003,** *37*, (20), 4702-4708.
16. De Haan, H.; De Boer, T., Applicability of light absorbance and fluorescence as measures of concentration and molecular size of dissolved organic carbon in humic Lake Tjeukemeer. *Water Research* **1987,** *21*, (6), 731-734.
17. Moran, M. A.; Sheldon, W. M., Jr.; Zepp, R. G., Carbon loss and optical property changes during long-term photochemical and biological degradation of estuarine dissolved organic matter. *Limnology and Oceanography* **2000,** *45*, (6), 1254-1264.
18. Twardowski, M. S.; Boss, E.; Sullivan, J. M.; Donaghay, P. L., Modeling the spectral shape of absorption by chromophoric dissolved organic matter. *Marine Chemistry* **2004,** *89*, (1), 69-88.

19. Peterson, B. M.; McNally, A. M.; Cory, R. M.; Thoemke, J. D.; Cotner, J. B.; McNeill, K., Spatial and temporal distribution of singlet oxygen in Lake Superior. *Environmental Science & Technology* **2012,** *46*, (13), 7222-7229.
20. Helms, J. R.; Stubbins, A.; Ritchie, J. D.; Minor, E. C.; Kieber, D. J.; Mopper, K., Absorption spectral slopes and slope ratios as indicators of molecular weight, source, and photobleaching of chromophoric dissolved organic matter. *Limnology and Oceanography* **2008,** *53*, (3), 955-969.
21. McKnight, D. M.; Boyer, E. W.; Westerhoff, P. K.; Doran, P. T.; Kulbe, T.; Andersen, D. T., Spectrofluorometric characterization of dissolved organic matter for indication of precursor organic material and aromaticity. *Limnology and Oceanography* **2001,** *46*, (1), 38-48.
22. Zsolnay, A.; Baigar, E.; Jimenez, M.; Steinweg, B.; Saccomandi, F., Differentiating with fluorescence spectroscopy the sources of dissolved organic matter in soils subjected to drying. *Chemosphere* **1999,** *38*, (1), 45-50.
23. Wilson, H. F.; Xenopoulos, M. A., Effects of agricultural land use on the composition of fluvial dissolved organic matter. *Nature Geoscience* **2008,** *2*, 37-41.
24. Murphy, K. R.; Stedmon, C. A.; Wenig, P.; Bro, R., OpenFluor- an online spectral library of auto-fluorescence by organic compounds in the environment. *Analytical Methods* **2014,** *6*, (3), 658-661.
25. Lambert, T.; Bouillon, S.; Darchambeau, F.; Massicotte, P.; Borges, A. V., Shift in the chemical composition of dissolved organic matter in the Congo River network. *Biogeosciences* **2016,** *13*, (18), 5405-5420.
26. Fellman, J. B.; Hood, E.; Spencer, R. G. M., Fluorescence spectroscopy opens new windows into dissolved organic matter dynamics in freshwater ecosystems: A review. *Limnology and Oceanography* **2010,** *55*, (6), 2452-2462.
27. Leifer, A., *The Kinetics of Environmental Aquatic Photochemistry: Theory and Practice*. American Chemical Society: Washington, D.C., 1988.
28. Schwarzenbach, R. P.; Gschwend, P. M.; Imboden, D. M., *Environmental Organic Chemistry*. 3rd ed.; John Wiley & Sons, Inc.: Hoboken, NJ, 2016.
29. Wasswa, J.; Driscoll, C. T.; Zeng, T., Photochemical characterization of surface waters from lakes in the Adirondack Region of New York. *Environmental Science & Technology* **2020,** *54*, (17), 10654-10667.
30. Partanen, S. B.; Erickson, P. R.; Latch, D. E.; Moor, K. J.; McNeill, K., Dissolved organic matter singlet oxygen quantum yields: Evaluation using time-resolved singlet oxygen phosphorescence. *Environmental Science & Technology* **2020,** *54*, (6), 3316-3324.
31. Wu, B.; Liu, T.; Wang, Y.; Zhao, G.; Chen, B.; Chu, C., High sample throughput LED reactor for facile characterization of the quantum yield spectrum of photochemically produced reactive intermediates. *Environmental Science & Technology* **2021,** *55*, (23), 16204-16214.
32. O'Connor, M.; Helal, S. R.; Latch, D. E.; Arnold, W. A., Quantifying photo-production of triplet excited states and singlet oxygen from effluent organic matter. *Water Research* **2019,** *156*, 23-33.
33. Sharpless, C. M.; Aeschbacher, M.; Page, S. E.; Wenk, J.; Sander, M.; McNeill, K., Photooxidation-induced changes in optical, electrochemical, and photochemical properties of humic substances. *Environmental Science & Technology* **2014,** *48*, (5), 2688-2696.
34. McCabe, A. J.; Arnold, W. A., Seasonal and spatial variabilities in the water chemistry of prairie pothole wetlands influence the photoproduction of reactive intermediates. *Chemosphere* **2016,** *155*, 640-647.
35. Mostafa, S.; Rosario-Ortiz, F. L., Singlet oxygen formation from wastewater organic matter. *Environmental Science & Technology* **2013,** *47*, (15), 8179-8186.
36. Haag, W. R.; Hoigné, J.; Gassman, E.; Braun, A. M., Singlet oxygen in surface waters - Part I: Furfuryl alcohol as a trapping agent. *Chemosphere* **1984,** *13*, (5-6), 631-640.
37. Appiani, E.; Ossola, R.; Latch, D. E.; Erickson, P. R.; McNeill, K., Aqueous singlet oxygen reaction kinetics of furfuryl alcohol: Effect of temperature, pH, and salt content. *Environmental Science: Processes & Impacts* **2017,** *19*, (4), 507-516.
38. Dalrymple, R. M.; Carfagno, A. K.; Sharpless, C. M., Correlations between dissolved organic matter optical properties and quantum yields of singlet oxygen and hydrogen peroxide. *Environmental Science & Technology* **2010,** *44*, (15), 5824-5829.

39. Apell, J. N.; McNeill, K., Updated and validated solar irradiance reference spectra for estimating environmental photodegradation rates. *Environmental Science: Processes & Impacts* **2019,** *21*, (3), 427-437.
40. Partanen, S. B.; Apell, J. N.; Lin, J.; McNeill, K., Factors affecting the mixed-layer concentrations of singlet oxygen in sunlit lakes. *Environmental Science: Processes & Impacts* **2021,** *23*, (8), 1130-1145.
41. Partanen, S. B.; McNeill, K., Global corrections to reference irradiance spectra for non-clear-sky conditions. *Environmental Science & Technology* **2023,** *57*, (6), 2682-2690.
42. Gueymard, C. A., *SMARTS2, A Simple Model of the Atmospheric Radiative Transfer of Sunshine: Algorithms and Performance Assessment*. Florida Solar Energy Center/University of Central Florida: Cocoa, FL, 1995.
43. Gueymard, C. A., Parameterized transmittance model for direct beam and circumsolar spectral irradiance. *Solar Energy* **2001,** *71*, (5), 325-346.
44. Gueymard, C. A., The SMARTS spectral irradiance model after 25 years: New developments and validation of reference spectra. *Solar Energy* **2019,** *187*, 233-253.
45. Zepp, R. G.; Cline, D. M., Rates of direct photolysis in aquatic environment. *Environmental Science & Technology* **1977,** *11*, (4), 359-366.
46. Bukaveckas, P. A.; Robbins-Forbes, M., Role of dissolved organic carbon in the attenuation of photosynthetically active and ultraviolet radiation in Adirondack lakes. *Freshwater Biology* **2000,** *43*, (3), 339-354.
47. Morris, D. P.; Zagarese, H.; Williamson, C. E.; Balseiro, E. G.; Hargreaves, B. R.; Modenutti, B.; Moeller, R.; Queimalinos, C., The attenuation of solar UV radiation in lakes and the role of dissolved organic carbon. *Limnology and Oceanography* **1995,** *40*, (8), 1381-1391.
48. Parrish, C. *Index of Refraction of Seawater and Freshwater as a Function of Wavelength and Temperature*; Oregon State University: Corvallis, OR, 2020. https://research.engr.oregonstate.edu/parrish/index-refraction-seawater-and-freshwater-function-wavelength-and-temperature (accessed June 20, 2025).
49. Gueymard, C. A., A reevaluation of the solar constant based on a 42-year total solar irradiance time series and a reconciliation of spaceborne observations. *Solar Energy* **2018,** *168*, 2-9.
50. National Aeronautics and Space Administration (NASA), Goddard Space Flight Center. *Aerosol Robotic Network (AERONET) Site Information Database*; National Aeronautics and Space Administration: Washington, D.C., 2024. https://aeronet.gsfc.nasa.gov/new_web/site_info_v3.html (accessed June 20, 2025).
51. National Aeronautics and Space Administration (NASA), Goddard Earth Sciences Data and Information Services Center (GES DISC). *OMI/Aura TOMS-Like Ozone and Radiative Cloud Fraction L3 1 day 0.25 degree x 0.25 degree V3 (OMTO3e)*; National Aeronautics and Space Administration: Washington, D.C., 2024. https://disc.gsfc.nasa.gov/datasets/OMTO3e_003/summary (accessed June 20, 2025).
52. National Oceanic and Atmospheric Administration (NOAA), Earth System Research Laboratories, Global Monitoring Laboratory. *Trends in Atmospheric Carbon Dioxide*; National Oceanic and Atmospheric Administration: Washington, D.C., 2024. https://gml.noaa.gov/ccgg/trends/data.html (accessed June 20, 2025).
53. Gueymard, C. A., Revised composite extraterrestrial spectrum based on recent solar irradiance observations. *Solar Energy* **2018,** *169*, 434-440.
54. Gueymard, C. A., Prediction and validation of cloudless shortwave solar spectra incident on horizontal, tilted, or tracking surfaces. *Solar Energy* **2008,** *82*, (3), 260-271.
55. Hanna, M., Evaluation of models predicting mixing depth. *Canadian Journal of Fisheries and Aquatic Sciences* **1990,** *47*, (5), 940-947.
56. Walter, J. A.; Fleck, R.; Kastens, J. H.; Pace, M. L.; Wilkinson, G. M., Temporal coherence between lake and landscape primary productivity. *Ecosystems* **2021,** *24*, (3), 502-515.
57. Sheppard, L. W.; Walter, J. A.; Anderson, T. L.; Zhao, L.; Reuman, D. *Wavelet approaches to synchrony (wsyn) package vignette*; 2019.
58. Paul, A.; Hackbarth, S.; Vogt, R. D.; Roder, B.; Burnison, B. K.; Steinberg, C. E. W., Photogeneration of singlet oxygen by humic substances: Comparison of humic substances of aquatic and terrestrial origin. *Photochemical & Photobiological Sciences* **2004,** *3*, (3), 273-280.

59. Adirondack Lakes Survey Corporation; New York State Department of Environmental Conservation. *The Adirondack Long-Term Monitoring Lakes: A Compendium of Site Descriptions, Recent Chemistry and Selected Research Information*; 19-30; The New York State Energy Research and Development Authority: Albany, NY, 2019. https://www.nyserda.ny.gov/-/media/Project/Nyserda/Files/Publications/Research/Environmental/19-30-ALTM-Compendium-Report.pdf (accessed June 20, 2025); pp 1-335.
60. Kretzer, W.; Roy, K.; Dukett, J.; Houck, N.; Philip, S. *Adirondack Lakes Survey 1984-1987 Complete Data Set*; Figshare, 2023. https://doi.org/10.6084/m9.figshare.22312732.v1 (accessed June 20, 2025).
61. Wang, S.; Matt, M.; Murphy, B. L.; Perkins, M.; Matthews, D. A.; Moran, S. D.; Zeng, T., Organic micropollutants in New York lakes: A statewide citizen science occurrence study. *Environmental Science & Technology* **2020,** *54*, (21), 13759-13770.
62. Dittman, J. A.; Driscoll, C. T., Factors influencing changes in mercury concentrations in lake water and yellow perch (Perca flavescens) in Adirondack lakes. *Biogeochemistry* **2009,** *93*, (3), 179-196.
63. Gorney, R. M.; June, S. G.; Stainbrook, K. M.; Smith, A. J., Detections of cyanobacteria harmful algal blooms (cyanoHABs) in New York State, United States (2012–2020). *Lake and Reservoir Management* **2023,** *39*, (1), 21-36.
64. Latch, D. E.; Stender, B. L.; Packer, J. L.; Arnold, W. A.; McNeill, K., Photochemical fate of pharmaceuticals in the environment: Cimetidine and ranitidine. *Environmental Science & Technology* **2003,** *37*, (15), 3342-3350.
65. Packer, J. L.; Werner, J. J.; Latch, D. E.; McNeill, K.; Arnold, W. A., Photochemical fate of pharmaceuticals in the environment: Naproxen, diclofenac, clofibric acid, and ibuprofen. *Aquatic Sciences* **2003,** *65*, (4), 342-351.
66. Boreen, A. L.; Arnold, W. A.; McNeill, K., Photochemical fate of sulfa drugs in the aquatic environment: Sulfa drugs containing five-membered heterocyclic groups. *Environmental Science & Technology* **2004,** *38*, (14), 3933-3940.
67. Latch, D. E.; Packer, J. L.; Stender, B. L.; VanOverbeke, J.; Arnold, W. A.; McNeill, K., Aqueous photochemistry of triclosan: Formation of 2,4-dichlorophenol, 2,8-dichlorodibenzo-*p*-dioxin, and oligomerization products. *Environmental Toxicology and Chemistry* **2005,** *24*, (3), 517-525.
68. Edhlund, B. L.; Arnold, W. A.; McNeill, K., Aquatic photochemistry of nitrofuran antibiotics. *Environmental Science & Technology* **2006,** *40*, (17), 5422-5427.
69. ter Halle, A.; Richard, C., Simulated solar light irradiation of mesotrione in natural waters. *Environmental Science & Technology* **2006,** *40*, (12), 3842-3847.
70. Castillo, C.; Criado, S.; Díaz, M.; García, N. A., Riboflavin as a sensitiser in the photodegradation of tetracyclines. Kinetics, mechanism and microbiological implications. *Dyes and Pigments* **2007,** *72*, (2), 178-184.
71. Smith, G. J., Photo-oxidation of tryptophan sensitized by methylene blue. *Journal of the Chemical Society, Faraday Transactions 2: Molecular and Chemical Physics* **1978,** *74*, (0), 1350-1354.
72. Barbieri, Y.; Massad, W. A.; Díaz, D. J.; Sanz, J.; Amat-Guerri, F.; García, N. A., Photodegradation of bisphenol A and related compounds under natural-like conditions in the presence of riboflavin: Kinetics, mechanism and photoproducts. *Chemosphere* **2008,** *73*, (4), 564-571.
73. Wilkinson, F.; Helman, W. P.; Ross, A. B., Rate constants for the decay and reactions of the lowest electronically excited singlet state of molecular oxygen in solution. An expanded and revised compilation. *Journal of Physical and Chemical Reference Data* **1995,** *24*, (2), 663-677.
74. Boreen, A. L.; Edhlund, B. L.; Cotner, J. B.; McNeill, K., Indirect photodegradation of dissolved free amino acids: The contribution of singlet oxygen and the differential reactivity of DOM from various sources. *Environmental Science & Technology* **2008,** *42*, (15), 5492-5498.
75. Chen, Y.; Hu, C.; Hu, X.; Qu, J., Indirect photodegradation of amine drugs in aqueous solution under simulated sunlight. *Environmental Science & Technology* **2009,** *43*, (8), 2760-2765.
76. Dell'Arciprete, M. L.; Santos-Juanes, L.; Arques, A.; Vercher, R. F.; Amat, A. M.; Furlong, J. P.; Mártire, D. O.; Gonzalez, M. C., Reactivity of neonicotinoid pesticides with singlet oxygen. *Catalysis Today* **2010,** *151*, (1–2), 137-142.

77. Razavi, B.; Ben Abdelmelek, S.; Song, W.; O'Shea, K. E.; Cooper, W. J., Photochemical fate of atorvastatin (lipitor) in simulated natural waters. *Water Research* **2011,** *45*, (2), 625-631.
78. Remucal, C. K.; McNeill, K., Photosensitized amino acid degradation in the presence of riboflavin and its derivatives. *Environmental Science & Technology* **2011,** *45*, (12), 5230-5237.
79. Xu, H.; Cooper, W. J.; Jung, J.; Song, W., Photosensitized degradation of amoxicillin in natural organic matter isolate solutions. *Water Research* **2011,** *45*, (2), 632-638.
80. Gollnick, K.; Griesbeck, A., Singlet oxygen photooxygenation of furans: Isolation and reactions of (4+2)-cycloaddition products (unsaturated sec.-ozonides). *Tetrahedron* **1985,** *41*, (11), 2057-2068.
81. Vione, D.; Maddigapu, P. R.; De Laurentiis, E.; Minella, M.; Pazzi, M.; Maurino, V.; Minero, C.; Kouras, S.; Richard, C., Modelling the photochemical fate of ibuprofen in surface waters. *Water Research* **2011,** *45*, (20), 6725-6736.
82. Maddigapu, P. R.; Minella, M.; Vione, D.; Maurino, V.; Minero, C., Modeling phototransformation reactions in surface water bodies: 2,4-Dichloro-6-nitrophenol as a case study. *Environmental Science & Technology* **2011,** *45*, (1), 209-214.
83. Felcyn, J. R.; Davis, J. C. C.; Tran, L. H.; Berude, J. C.; Latch, D. E., Aquatic photochemistry of isoflavone phytoestrogens: Degradation kinetics and pathways. *Environmental Science & Technology* **2012,** *46*, (12), 6698-6704.
84. Kelly, M. M.; Arnold, W. A., Direct and indirect photolysis of the phytoestrogens genistein and daidzein. *Environmental Science & Technology* **2012,** *46*, (10), 5396-5403.
85. Santoke, H.; Song, W.; Cooper, W. J.; Peake, B. M., Advanced oxidation treatment and photochemical fate of selected antidepressant pharmaceuticals in solutions of Suwannee River humic acid. *Journal of Hazardous Materials* **2012,** *217-218*, 382-390.
86. Monadjemi, S.; Halle, A. t.; Richard, C., Reactivity of cycloxydim toward singlet oxygen in solution and on wax film. *Chemosphere* **2012,** *89*, (3), 269-273.
87. Wang, L.; Xu, H.; Cooper, W. J.; Song, W., Photochemical fate of beta-blockers in NOM enriched waters. *Science of The Total Environment* **2012,** *426*, 289-295.
88. Luo, X.; Zheng, Z.; Greaves, J.; Cooper, W. J.; Song, W., Trimethoprim: Kinetic and mechanistic considerations in photochemical environmental fate and AOP treatment. *Water Research* **2012,** *46*, (4), 1327-1336.
89. De Laurentiis, E.; Chiron, S.; Kouras-Hadef, S.; Richard, C.; Minella, M.; Maurino, V.; Minero, C.; Vione, D., Photochemical fate of carbamazepine in surface freshwaters: Laboratory measures and modeling. *Environmental Science & Technology* **2012,** *46*, (15), 8164-8173.
90. Yang, W.; Ben Abdelmelek, S.; Zheng, Z.; An, T.; Zhang, D.; Song, W., Photochemical transformation of terbutaline (pharmaceutical) in simulated natural waters: Degradation kinetics and mechanisms. *Water Research* **2013,** *47*, (17), 6558-6565.
91. Vione, D.; Caringella, R.; De Laurentiis, E.; Pazzi, M.; Minero, C., Phototransformation of the sunlight filter benzophenone-3 (2-hydroxy-4-methoxybenzophenone) under conditions relevant to surface waters. *Science of The Total Environment* **2013,** *463–464*, 243-251.
92. Zeng, T.; Arnold, W. A., Pesticide photolysis in prairie potholes: Probing photosensitized processes. *Environmental Science & Technology* **2013,** *47*, (13), 6735-6745.
93. Yan, S.; Zhang, D.; Song, W., Mechanistic considerations of photosensitized transformation of microcystin-LR (cyanobacterial toxin) in aqueous environments. *Environmental Pollution* **2014,** *193*, 111-118.
94. De Laurentiis, E.; Prasse, C.; Ternes, T. A.; Minella, M.; Maurino, V.; Minero, C.; Sarakha, M.; Brigante, M.; Vione, D., Assessing the photochemical transformation pathways of acetaminophen relevant to surface waters: Transformation kinetics, intermediates, and modelling. *Water Research* **2014,** *53*, 235-248.
95. Avetta, P.; Marchetti, G.; Minella, M.; Pazzi, M.; De Laurentiis, E.; Maurino, V.; Minero, C.; Vione, D., Phototransformation pathways of the fungicide dimethomorph ((E,Z) 4-[3-(4-chlorophenyl)-3-(3,4-dimethoxyphenyl)-1-oxo-2-propenyl]morpholine), relevant to sunlit surface waters. *Science of The Total Environment* **2014,** *500-501*, 351-360.

96. Zhou, C.; Chen, J.; Xie, Q.; Wei, X.; Zhang, Y.-n.; Fu, Z., Photolysis of three antiviral drugs acyclovir, zidovudine and lamivudine in surface freshwater and seawater. *Chemosphere* **2015,** *138*, 792-797.
97. Janssen, E. M. L.; Marron, E.; McNeill, K., Aquatic photochemical kinetics of benzotriazole and structurally related compounds. *Environmental Science: Processes & Impacts* **2015,** *17*, (5), 939-946.
98. Chu, C.; Lundeen, R. A.; Remucal, C. K.; Sander, M.; McNeill, K., Enhanced indirect photochemical transformation of histidine and histamine through association with chromophoric dissolved organic matter. *Environmental Science & Technology* **2015,** *49*, (9), 5511-5519.
99. Vione, D.; Calza, P.; Galli, F.; Fabbri, D.; Santoro, V.; Medana, C., The role of direct photolysis and indirect photochemistry in the environmental fate of ethylhexyl methoxy cinnamate (EHMC) in surface waters. *Science of The Total Environment* **2015,** *537*, 58-68.
100. An, J.; Li, G.; An, T.; Nie, X., Indirect photochemical transformations of acyclovir and penciclovir in aquatic environments increase ecological risk. *Environmental Toxicology and Chemistry* **2016,** *35*, (3), 584-592.
101. Ren, D.; Huang, B.; Bi, T.; Xiong, D.; Pan, X., Effects of pH and dissolved oxygen on the photodegradation of 17α-ethynylestradiol in dissolved humic acid solution. *Environmental Science: Processes & Impacts* **2016,** *18*, (1), 78-86.
102. Chu, C.; Erickson, P. R.; Lundeen, R. A.; Stamatelatos, D.; Alaimo, P. J.; Latch, D. E.; McNeill, K., Photochemical and nonphotochemical transformations of cysteine with dissolved organic matter. *Environmental Science & Technology* **2016,** *50*, (12), 6363-6373.
103. Lundeen, R. A.; Chu, C.; Sander, M.; McNeill, K., Photooxidation of the antimicrobial, nonribosomal peptide bacitracin A by singlet oxygen under environmentally relevant conditions. *Environmental Science & Technology* **2016,** *50*, (16), 8586-8595.
104. Avetta, P.; Fabbri, D.; Minella, M.; Brigante, M.; Maurino, V.; Minero, C.; Pazzi, M.; Vione, D., Assessing the phototransformation of diclofenac, clofibric acid and naproxen in surface waters: Model predictions and comparison with field data. *Water Research* **2016,** *105*, 383-394.
105. Li, R.; Zhao, C.; Yao, B.; Li, D.; Yan, S.; O'Shea, K. E.; Song, W., Photochemical transformation of aminoglycoside antibiotics in simulated natural waters. *Environmental Science & Technology* **2016,** *50*, (6), 2921-2930.
106. Santoke, H.; Cooper, W. J., Environmental photochemical fate of selected pharmaceutical compounds in natural and reconstituted Suwannee River water: Role of reactive species in indirect photolysis. *Science of The Total Environment* **2017,** *580*, 626-631.
107. Davis, C. A.; Erickson, P. R.; McNeill, K.; Janssen, E. M. L., Environmental photochemistry of fenamate NSAIDs and their radical intermediates. *Environmental Science: Processes & Impacts* **2017,** *19*, (5), 656-665.
108. McConville, M. B.; Mezyk, S. P.; Remucal, C. K., Indirect photodegradation of the lampricides TFM and niclosamide. *Environmental Science: Processes & Impacts* **2017,** *19*, (8), 1028-1039.
109. Li, Y.; Pan, Y.; Lian, L.; Yan, S.; Song, W.; Yang, X., Photosensitized degradation of acetaminophen in natural organic matter solutions: The role of triplet states and oxygen. *Water Research* **2017,** *109*, 266-273.
110. Carena, L.; Minella, M.; Barsotti, F.; Brigante, M.; Milan, M.; Ferrero, A.; Berto, S.; Minero, C.; Vione, D., Phototransformation of the herbicide propanil in paddy field water. *Environmental Science & Technology* **2017,** *51*, (5), 2695-2704.
111. Wang, J.; Chen, J.; Qiao, X.; Zhang, Y.-n.; Uddin, M.; Guo, Z., Disparate effects of DOM extracted from coastal seawaters and freshwaters on photodegradation of 2,4-Dihydroxybenzophenone. *Water Research* **2019,** *151*, 280-287.
112. Chen, X.; Wang, J.; Chen, J.; Zhou, C.; Cui, F.; Sun, G., Photodegradation of 2-(2-hydroxy-5-methylphenyl)benzotriazole (UV-P) in coastal seawaters: Important role of DOM. *Journal of Environmental Sciences* **2019,** *85*, 129-137.
113. Apell, J. N.; Pflug, N. C.; McNeill, K., Photodegradation of fludioxonil and other pyrroles: The importance of indirect photodegradation for understanding environmental fate and photoproduct formation. *Environmental Science & Technology* **2019,** *53*, (19), 11240-11250.

114. Ge, L.; Zhang, P.; Halsall, C.; Li, Y.; Chen, C.-E.; Li, J.; Sun, H.; Yao, Z., The importance of reactive oxygen species on the aqueous phototransformation of sulfonamide antibiotics: Kinetics, pathways, and comparisons with direct photolysis. *Water Research* **2019,** *149*, 243-250.
115. Jaramillo, M.; Joens, J. A.; O'Shea, K. E., Fundamental studies of the singlet oxygen reactions with the potent marine toxin domoic acid. *Environmental Science & Technology* **2020,** *54*, (10), 6073-6081.
116. Pozdnyakov, I. P.; Tyutereva, Y. E.; Parkhats, M. V.; Grivin, V. P.; Fang, Y.; Liu, L.; Wan, D.; Luo, F.; Chen, Y., Mechanistic investigation of humic substances assisted photodegradation of imipramine under simulated sunlight. *Science of The Total Environment* **2020,** *738*, Article Number: 140298.
117. Pozdnyakov, I. P.; Parkhats, M. V., Direct measurements of the reactivity of singlet oxygen to some persistent herbicides in aqueous solutions. *Chemosphere* **2020,** *247*, Article Number: 125872.
118. Sunday, M. O.; Takeda, K.; Sakugawa, H., Singlet oxygen photogeneration in coastal seawater: Prospect of large-scale modeling in seawater surface and its environmental significance. *Environmental Science & Technology* **2020,** *54*, (10), 6125-6133.
119. Davis, C. A.; Janssen, E. M. L., Environmental fate processes of antimicrobial peptides daptomycin, bacitracins, and polymyxins. *Environment International* **2020,** *134*, Article Number: 105271.
120. Natumi, R.; Dieziger, C.; Janssen, E. M. L., Cyanobacterial toxins and cyanopeptide transformation kinetics by singlet oxygen and pH-dependence in sunlit surface waters. *Environmental Science & Technology* **2021,** *55*, (22), 15196-15205.
121. Cai, Y.; Apell, J. N.; Pflug, N. C.; McNeill, K.; Bollmann, U. E., Photochemical fate of medetomidine in coastal and marine environments. *Water Research* **2021,** *191*, Article Number: 116791.
122. Jia, X.; Lian, L.; Yan, S.; Song, Y.; Nie, J.; Zhu, X.; Song, W., Comprehensive understanding of the phototransformation process of macrolide antibiotics in simulated natural waters. *ACS ES&T Water* **2021,** *1*, (4), 938-948.
123. Lian, L.; Miao, C.; Hao, Z.; Liu, Q.; Liu, Y.; Song, W.; Yan, S., Reevaluation of the contributions of reactive intermediates to the photochemical transformation of 17β-estradiol in sewage effluent. *Water Research* **2021,** *189*, Article Number: 116633.
124. Gornik, T.; Carena, L.; Kosjek, T.; Vione, D., Phototransformation study of the antidepressant paroxetine in surface waters. *Science of The Total Environment* **2021,** *774*, Article Number: 145380.
125. Wan, D.; Wang, J.; Chen, T.; Xiang, W.; Selvinsimpson, S.; Chen, Y., Effect of disinfection on the photoreactivity of effluent organic matter and photodegradation of organic contaminants. *Water Research* **2022,** *219*, Article Number: 118552.
126. Norris, K. E.; Kurtz, T.; Wang, S.; Zeng, T.; Leresche, F.; Rosario-Ortiz, F. L., Photochemical degradation of saxitoxins in surface waters. *ACS ES&T Water* **2024,** *4*, (2), 346-354.
127. Wang, J.; Guo, Z.; Guo, Y.; Zhang, Y.; Yu, P.; Ye, Z.; Qian, Y.; Yoshimura, C.; Wang, T.; Zhang, L., Photochemical fate of β-blocker pindolol in riverine and its downstream coastal waters. *Science of The Total Environment* **2024,** *927*, Article Number: 172236.
128. Lee, D.; Alyami, I.; Zimila, H.; Arnold, R. G.; Quanrud, D. M.; Sáez, A. E., Photolytic transformation of trace organic compounds: Roles of direct photolysis and indirect photolysis by singlet oxygen. *Water Research* **2025,** *283*, Article Number: 123799.
129. Zong, C.; Cui, J.; Qu, J.; Cheng, F.; Zhang, Y.-N., Insights into photodegradation of antibiotics in aquatic environment considering effects of dissolved organic matter and halide ions. *Environmental Earth Sciences* **2025,** *84*, (10), Article Number: 254.
130. Arciva, S.; Zhou, Y.; Jiang, W.; Ross, A.; Zhang, Q.; Anastasio, C., Aqueous oxidation of biomass-burning furans by singlet molecular oxygen ($^1O_2$*). *Environmental Science & Technology* **2025,** *59*, (21), 10357-10367.
131. de Brito Anton, L.; Silverman, A. I.; Apell, J. N., Comparing photodegradation model systems: Measuring bimolecular rate constants between photochemically produced reactive intermediates and organic contaminants. *Environmental Science: Processes & Impacts* **2025**, DOI: 10.1039/D5EM00199D.
132. Zepp, R. G.; Wolfe, N. L.; Baughman, G. L.; Hollis, R. C., Singlet oxygen in natural waters. *Nature* **1977,** *267*, (5610), 421-423.

133. Baxter, R. M.; Carey, J. H., Reactions of singlet oxygen in humic waters. *Freshwater Biology* **1982,** *12*, (3), 285-292.
134. Haag, W. R.; Hoigné, J.; Gassman, E.; Braun, A. M., Singlet oxygen in surface waters - Part II: Quantum yields of its production by some natural humic materials as a function of wavelength. *Chemosphere* **1984,** *13*, (5-6), 641-650.
135. Zepp, R. G.; Schlotzhauer, P. F.; Sink, R. M., Photosensitized transformations involving electronic energy transfer in natural waters: Role of humic substances. *Environmental Science & Technology* **1985,** *19*, (1), 74-81.
136. Frimmel, F. H.; Bauer, H.; Putzien, J.; Murasecco, P.; Braun, A. M., Laser flash photolysis of dissolved aquatic humic material and the sensitized production of singlet oxygen. *Environmental Science & Technology* **1987,** *21*, (6), 541-545.
137. Faust, B. C.; Allen, J. M., Aqueous-phase photochemical sources of peroxyl radicals and singlet molecular oxygen in clouds and fog. *Journal of Geophysical Research: Atmospheres* **1992,** *97*, (D12), 12913-12926.
138. Shao, C.; Cooper, W. J.; Lean, D. R. S., Singlet oxygen formation in lake waters from mid-latitudes. In *Aquatic and Surface Photochemistry*, Helz, G. R.; Zepp, R. G.; Crosby, D. G., Eds. CRC Press: Boca Raton, FL, 1994; pp 215-222.
139. Richard, C.; Vialaton, D.; Aguer, J.-P.; Andreux, F., Transformation of monuron photosensitized by soil extracted humic substances: energy or hydrogen transfer mechanism? *Journal of Photochemistry and Photobiology A: Chemistry* **1997,** *111*, (1), 265-271.
140. Sandvik, S. L. H.; Bilski, P.; Pakulski, J. D.; Chignell, C. F.; Coffin, R. B., Photogeneration of singlet oxygen and free radicals in dissolved organic matter isolated from the Mississippi and Atchafalaya River plumes. *Marine Chemistry* **2000,** *69*, (1), 139-152.
141. Bilski, P.; Burkhart, J. G.; Chignell, C. F., Photochemical characterization of water samples from Minnesota and Vermont sites with malformed frogs: Potential influence of photosensitization by singlet molecular oxygen ($^1O_2$) and free radicals on aquatic toxicity. *Aquatic Toxicology* **2003,** *65*, (3), 229-241.
142. Halladja, S.; ter Halle, A.; Aguer, J.-P.; Boulkamh, A.; Richard, C., Inhibition of humic substances mediated photooxygenation of furfuryl alcohol by 2,4,6-trimethylphenol. Evidence for reactivity of the phenol with humic triplet excited states. *Environmental Science & Technology* **2007,** *41*, (17), 6066-6073.
143. Cory, R. M.; Cotner, J. B.; McNeill, K., Quantifying interactions between singlet oxygen and aquatic fulvic acids. *Environmental Science & Technology* **2008,** *43*, (3), 718-723.
144. Carlos, L.; Cipollone, M.; Soria, D. B.; Sergio Moreno, M.; Ogilby, P. R.; García Einschlag, F. S.; Mártire, D. O., The effect of humic acid binding to magnetite nanoparticles on the photogeneration of reactive oxygen species. *Separation and Purification Technology* **2012,** *91*, 23-29.
145. Sharpless, C. M., Lifetimes of triplet dissolved natural organic matter (DOM) and the effect of $NaBH_4$ reduction on singlet oxygen quantum yields: Implications for DOM photophysics. *Environmental Science & Technology* **2012,** *46*, (8), 4466-4473.
146. De Laurentiis, E.; Buoso, S.; Maurino, V.; Minero, C.; Vione, D., Optical and photochemical characterization of chromophoric dissolved organic matter from lakes in Terra Nova Bay, Antarctica. Evidence of considerable photoreactivity in an extreme environment. *Environmental Science & Technology* **2013,** *47*, (24), 14089-14098.
147. Glover, C. M.; Rosario-Ortiz, F. L., Impact of halides on the photoproduction of reactive intermediates from organic matter. *Environmental Science & Technology* **2013,** *47*, (24), 13949-13956.
148. Cawley, K. M.; Korak, J. A.; Rosario-Ortiz, F. L., Quantum yields for the formation of reactive intermediates from dissolved organic matter samples from the Suwannee River. *Environmental Engineering Science* **2014,** *32*, (1), 31-37.
149. Janssen, E. M. L.; Erickson, P. R.; McNeill, K., Dual roles of dissolved organic matter as sensitizer and quencher in the photooxidation of tryptophan. *Environmental Science & Technology* **2014,** *48*, (9), 4916-4924.
150. Mostafa, S.; Korak, J. A.; Shimabuku, K.; Glover, C. M.; Rosario-Ortiz, F. L., Relation between optical properties and formation of reactive intermediates from different size fractions of organic matter. In *Advances in the Physicochemical Characterization of Dissolved Organic Matter: Impact on Natural and Engineered Systems*, Rosario-Ortiz, F. L., Ed. American Chemical Society: 2014; Vol. 1160, pp 159-179.

151. Nkhili, E.; Boguta, P.; Bejger, R.; Guyot, G.; Sokołowska, Z.; Richard, C., Photosensitizing properties of water-extractable organic matter from soils. *Chemosphere* **2014,** *95*, 317-323.
152. Zhang, D.; Yan, S.; Song, W., Photochemically induced formation of reactive oxygen species (ROS) from effluent organic matter. *Environmental Science & Technology* **2014,** *48*, (21), 12645-12653.
153. Bodhipaksha, L. C.; Sharpless, C. M.; Chin, Y.-P.; Sander, M.; Langston, W. K.; MacKay, A. A., Triplet photochemistry of effluent and natural organic matter in whole water and isolates from effluent-receiving rivers. *Environmental Science & Technology* **2015,** *49*, (6), 3453-3463.
154. Marchisio, A.; Minella, M.; Maurino, V.; Minero, C.; Vione, D., Photogeneration of reactive transient species upon irradiation of natural water samples: Formation quantum yields in different spectral intervals, and implications for the photochemistry of surface waters. *Water Research* **2015,** *73*, 145-156.
155. Fu, H.; Liu, H.; Mao, J.; Chu, W.; Li, Q.; Alvarez, P. J. J.; Qu, X.; Zhu, D., Photochemistry of dissolved black carbon released from biochar reactive oxygen species generation and phototransformation. *Environmental Science & Technology* **2016,** *50*, (3), 1218-1226.
156. McKay, G.; Couch, K. D.; Mezyk, S. P.; Rosario-Ortiz, F. L., Investigation of the coupled effects of molecular weight and charge-transfer interactions on the optical and photochemical properties of dissolved organic matter. *Environmental Science & Technology* **2016,** *50*, (15), 8093-8102.
157. Bodhipaksha, L. C.; Sharpless, C. M.; Chin, Y.-P.; MacKay, A. A., Role of effluent organic matter in the photochemical degradation of compounds of wastewater origin. *Water Research* **2017,** *110*, (Supplement C), 170-179.
158. Maizel, A. C.; Remucal, C. K., Molecular composition and photochemical reactivity of size-fractionated dissolved organic matter. *Environmental Science & Technology* **2017,** *51*, (4), 2113-2123.
159. Maizel, A. C.; Li, J.; Remucal, C. K., Relationships between dissolved organic matter composition and photochemistry in lakes of diverse trophic status. *Environmental Science & Technology* **2017,** *51*, (17), 9624-9632.
160. Maizel, A. C.; Remucal, C. K., The effect of probe choice and solution conditions on the apparent photoreactivity of dissolved organic matter. *Environmental Science: Processes & Impacts* **2017,** *19*, (8), 1040-1050.
161. McKay, G.; Huang, W.; Romera-Castillo, C.; Crouch, J. E.; Rosario-Ortiz, F. L.; Jaffé, R., Predicting reactive intermediate quantum yields from dissolved organic matter photolysis using optical properties and antioxidant capacity. *Environmental Science & Technology* **2017,** *51*, (10), 5404-5413.
162. Pozdnyakov, I. P.; Salomatova, V. A.; Parkhats, M. V.; Dzhagarov, B. M.; Bazhin, N. M., Efficiency of singlet oxygen generation by fulvic acids and its influence on UV photodegradation of herbicide Amitrole in aqueous solutions. *Mendeleev Communications* **2017,** *27*, (4), 399-401.
163. Silva, M. P.; Lastre-Acosta, A. M.; Mostafa, S.; McKay, G.; Linden, K. G.; Rosario-Ortiz, F. L.; Teixeira, A. C. S. C., Photochemical generation of reactive intermediates from urban-waste bio-organic substances under UV and solar irradiation. *Environmental Science and Pollution Research* **2017,** *24*, (22), 18470-18478.
164. Tenorio, R.; Fedders, A. C.; Strathmann, T. J.; Guest, J. S., Impact of growth phases on photochemically produced reactive species in the extracellular matrix of algal cultivation systems. *Environmental Science: Water Research & Technology* **2017,** *3*, (6), 1095-1108.
165. Zhou, H.; Lian, L.; Yan, S.; Song, W., Insights into the photo-induced formation of reactive intermediates from effluent organic matter: The role of chemical constituents. *Water Research* **2017,** *112*, 120-128.
166. Du, Z.; He, Y.; Fan, J.; Fu, H.; Zheng, S.; Xu, Z.; Qu, X.; Kong, A.; Zhu, D., Predicting apparent singlet oxygen quantum yields of dissolved black carbon and humic substances using spectroscopic indices. *Chemosphere* **2018,** *194*, 405-413.
167. Hong, J.; Liu, J.; Wang, L.; Kong, S.; Tong, C.; Qin, J.; Chen, L.; Sui, Y.; Li, B., Characterization of reactive photoinduced species in rainwater. *Environmental Science and Pollution Research* **2018,** *25*, (36), 36368-36380.
168. Zhou, C.; Chen, J.; Xie, H.; Zhang, Y.-N.; Li, Y.; Wang, Y.; Xie, Q.; Zhang, S., Modeling photodegradation kinetics of organic micropollutants in water bodies: A case of the Yellow River estuary. *Journal of Hazardous Materials* **2018,** *349*, 60-67.

169. Berg, S. M.; Whiting, Q. T.; Herrli, J. A.; Winkels, R.; Wammer, K. H.; Remucal, C. K., The role of dissolved organic matter composition in determining photochemical reactivity at the molecular level. *Environmental Science & Technology* **2019,** *53*, (20), 11725-11734.
170. Leresche, F.; McKay, G.; Kurtz, T.; von Gunten, U.; Canonica, S.; Rosario-Ortiz, F. L., Effects of ozone on the photochemical and photophysical properties of dissolved organic matter. *Environmental Science & Technology* **2019,** *53*, (10), 5622-5632.
171. Wan, D.; Sharma, V. K.; Liu, L.; Zuo, Y.; Chen, Y., Mechanistic insight into the effect of metal ions on photogeneration of reactive species from dissolved organic matter. *Environmental Science & Technology* **2019,** *53*, (10), 5778-5786.
172. Wenk, J.; Nguyen, M. T.; Nelson, K. L., Natural photosensitizers in constructed unit process wetlands: Photochemical characterization and inactivation of pathogen indicator organisms. *Environmental Science & Technology* **2019,** *53*, (13), 7724-7735.
173. Zhou, H.; Yan, S.; Lian, L.; Song, W., Triplet-state photochemistry of dissolved organic matter: Triplet-state energy distribution and surface electric charge conditions. *Environmental Science & Technology* **2019,** *53*, (5), 2482-2490.
174. Chen, Y.; Hozalski, R. M.; Olmanson, L. G.; Page, B. P.; Finlay, J. C.; Brezonik, P. L.; Arnold, W. A., Prediction of photochemically produced reactive intermediates in surface waters via satellite remote sensing. *Environmental Science & Technology* **2020,** *54*, (11), 6671-6681.
175. Palma, D.; Sleiman, M.; Voldoire, O.; Beauger, A.; Parlanti, E.; Richard, C., Study of the dissolved organic matter (DOM) of the Auzon cut-off meander (Allier River, France) by spectral and photoreactivity approaches. *Environmental Science and Pollution Research* **2020,** *27*, (21), 26385-26394.
176. Wan, D.; Kong, Y.; Selvinsimpson, S.; Luo, F.; Chen, Y., Effect of UV_{254} disinfection on the photoformation of reactive species from effluent organic matter of wastewater treatment plant. *Water Research* **2020,** *185*, Article Number: 116301.
177. Wang, H.; Zhou, H.; Ma, J.; Nie, J.; Yan, S.; Song, W., Triplet photochemistry of dissolved black carbon and its effects on the photochemical formation of reactive oxygen species. *Environmental Science & Technology* **2020,** *54*, (8), 4903-4911.
178. Zhao, J.; Zhou, Y.; Li, C.; Xie, Q.; Chen, J.; Chen, G.; Peijnenburg, W. J. G. M.; Zhang, Y.-n.; Qu, J., Development of a quantitative structure-activity relationship model for mechanistic interpretation and quantum yield prediction of singlet oxygen generation from dissolved organic matter. *Science of The Total Environment* **2020,** *712*, Article Number: 136450.
179. Zhou, C.; Xie, Q.; Wang, J.; Chen, X.; Niu, J.; Chen, J., Effects of dissolved organic matter derived from freshwater and seawater on photodegradation of three antiviral drugs. *Environmental Pollution* **2020,** *258*, 113700.
180. Guo, Z.; Wang, J.; Chen, X.; Cui, F.; Wang, T.; Zhou, C.; Song, G.; Zhang, S.; Chen, J., Photochemistry of dissolved organic matter extracted from coastal seawater: Excited triplet-states and contents of phenolic moieties. *Water Research* **2021,** *188*, Article Number: 116568.
181. Leresche, F.; Torres-Ruiz, J. A.; Kurtz, T.; von Gunten, U.; Rosario-Ortiz, F. L., Optical properties and photochemical production of hydroxyl radical and singlet oxygen after ozonation of dissolved organic matter. *Environmental Science: Water Research & Technology* **2021,** *7*, (2), 346-356.
182. Tu, Y.; Liu, H.; Li, Y.; Zhang, Z.; Lei, Y.; Zhao, Q.; Tian, S., Radical chemistry of dissolved black carbon under sunlight irradiation: Quantum yield prediction and effects on sulfadiazine photodegradation. *Environmental Science and Pollution Research* **2022,** *29*, (15), 21517-21527.
183. Wang, Z.; Lv, J.; Zhang, S.; Christie, P.; Zhang, S., Interfacial molecular fractionation on ferrihydrite reduces the photochemical reactivity of dissolved organic matter. *Environmental Science & Technology* **2021,** *55*, (3), 1769-1778.
184. Wan, D.; Wang, J.; Dionysiou, D. D.; Kong, Y.; Yao, W.; Selvinsimpson, S.; Chen, Y., Photogeneration of reactive species from biochar-derived dissolved black carbon for the degradation of amine and phenolic pollutants. *Environmental Science & Technology* **2021,** *55*, (13), 8866-8876.

185. Wan, D.; Wang, H.; Sharma, V. K.; Selvinsimpson, S.; Dai, H.; Luo, F.; Wang, C.; Chen, Y., Mechanistic investigation of enhanced photoreactivity of dissolved organic matter after chlorination. *Environmental Science & Technology* **2021,** *55*, (13), 8937-8946.
186. Zeng, Y.; Fang, G.; Fu, Q.; Dionysiou, D. D.; Wang, X.; Gao, J.; Zhou, D.; Wang, Y., Photochemical characterization of paddy water during rice cultivation: Formation of reactive intermediates for As(III) oxidation. *Water Research* **2021,** *206*, Article Number: 117721.
187. Zhou, Y.; Cheng, F.; He, D.; Zhang, Y.-n.; Qu, J.; Yang, X.; Chen, J.; Peijnenburg, W. J. G. M., Effect of UV/chlorine treatment on photophysical and photochemical properties of dissolved organic matter. *Water Research* **2021,** *192*, Article Number: 116857.
188. Zhou, S.; Liao, Z.; Zhang, B.; Hou, R.; Wang, Y.; Zhou, S.; Zhang, Y.; Ren, Z. J.; Yuan, Y., Photochemical behavior of microbial extracellular polymeric substances in the aquatic environment. *Environmental Science & Technology* **2021,** *55*, (22), 15090-15099.
189. Hu, A.; Li, L.; Huang, Y.; Fu, Q.-L.; Wang, D.; Zhang, W., Photochemical transformation mechanisms of dissolved organic matters (DOM) derived from different bio-stabilization sludge. *Environment International* **2022,** *169*, Article Number: 107534.
190. Liao, Z.; Wang, Y.; Xie, K.; Xie, N.; Cai, X.; Zhou, L.; Yuan, Y., Photochemistry of dissolved organic matter in water from the Pearl river (China): Seasonal patterns and predictive modelling. *Water Research* **2022,** *208*, Article Number: 117875.
191. Liu, Y.; Wang, M.; Yin, S.; Xie, L.; Qu, X.; Fu, H.; Shi, Q.; Zhou, F.; Xu, F.; Tao, S.; Zhu, D., Comparing photoactivities of dissolved organic matter released from rice straw-pyrolyzed biochar and composted rice straw. *Environmental Science & Technology* **2022,** *56*, (4), 2803-2815.
192. Zhang, T.; Ma, H.; Hong, Z.; Fu, G.; Zheng, Y.; Li, Z.; Cui, F., Photo-reactivity and photo-transformation of algal dissolved organic matter unraveled by optical spectroscopy and high-resolution mass spectrometry analysis. *Environmental Science & Technology* **2022,** *56*, (18), 13439-13448.
193. Song, N.; Wu, D.; Xu, H.; Jiang, H., Integrated evaluation of the reactive oxygen species (ROS) production characteristics in one large lake under alternating flood and drought conditions. *Water Research* **2022,** *225*, Article Number: 119136.
194. Wan, D.; Kong, Y.; Wang, X.; Selvinsimpson, S.; Sharma, V. K.; Zuo, Y.; Chen, Y., Effect of permanganate oxidation on the photoreactivity of dissolved organic matter for photodegradation of typical pharmaceuticals. *Science of The Total Environment* **2022,** *813*, Article Number: 152647.
195. Wan, D.; Yang, J.; Wang, X.; Xiang, W.; Selvinsimpson, S.; Chen, Y., Wavelength-dependent photoreactivity of root exudates from aquatic plants under UV-LED irradiation. *ACS ES&T Water* **2022,** *2*, (12), 2613-2622.
196. Wasswa, J.; Driscoll, C. T.; Zeng, T., Contrasting impacts of photochemical and microbial processing on the photoreactivity of dissolved organic matter in an Adirondack Lake watershed. *Environmental Science & Technology* **2022,** *56*, (3), 1688-1701.
197. Xu, H.; Li, X.; Guo, M.; Li, F.; Yang, K.; Liu, X., Dissolved organic matters with low molecular weight fractions exhibit high photochemical potential for reactive oxygen formation. *Chemosphere* **2022,** *305*, Article Number: 135542.
198. Zeng, Y.; Fang, G.; Fu, Q.; Peng, F.; Wang, X.; Dionysiou, D. D.; Guo, J.; Gao, J.; Zhou, D.; Wang, Y., Mechanistic study of the effects of agricultural amendments on photochemical processes in paddy water during rice growth. *Environmental Science & Technology* **2022,** *56*, (7), 4221-4230.
199. Allen, A.; Cheng, K.; McKay, G., Evaluating the pH-dependence of DOM absorbance, fluorescence, and photochemical production of singlet oxygen. *Environmental Science: Processes & Impacts* **2023,** *25*, (12), 1974-1985.
200. Berg, S. M.; Wammer, K. H.; Remucal, C. K., Dissolved organic matter photoreactivity is determined by its optical properties, redox activity, and molecular composition. *Environmental Science & Technology* **2023,** *57*, (16), 6703-6711.

201. Buckley, S.; Leresche, F.; Hanson, B.; Rosario-Ortiz, F. L., Decoupling optical response and photochemical formation of singlet oxygen in size isolated fractions of ozonated dissolved organic matter. *Environmental Science & Technology* **2023,** *57*, (14), 5603-5610.
202. Guo, Z.; Wang, T.; Chen, G.; Wang, J.; Fujii, M.; Yoshimura, C., Apparent quantum yield for photo-production of singlet oxygen in reservoirs and its relation to the water matrix. *Water Research* **2023,** *244*, Article Number: 120456.
203. Kong, S.; Liu, X.; Jiang, H.; Hong, W.; Zhang, J.; Song, W.; Yan, S., Photobleaching-induced changes in the optical and photochemical properties of algal organic matter. *Water Research* **2023,** *243*, Article Number: 120395.
204. Chen, X.; Deng, L.; Chen, Y.; Wan, D., Impact of ozonation on the optical properties and photo-reactivity of dissolved organic matter. *Journal of Environmental Chemical Engineering* **2023,** *11*, (1), Article Number: 109251.
205. Wang, Y.; Wu, B.; Zheng, X.; Chen, B.; Chu, C., Assessing the quantum yield spectrum of photochemically produced reactive intermediates from black carbon of various sources and properties. *Water Research* **2023,** *229*, Article Number: 119450.
206. Du, R.; Wen, J.; Huang, J.; Zhang, Q.; Shi, X.; Wang, B.; Deng, S.; Yu, G., Dissolved organic matter isolates obtained by solid phase extraction exhibit higher absorption and lower photo-reactivity: Effect of components. *Water Research* **2024**, Article Number: 121604.
207. Guo, Z.-C.; Zhang, L.; Chen, Y.; Huang, C.; Liao, Z.-M., Effect of UV-LED wavelength on reactive species photogeneration from dissolved organic matter. *Water* **2024,** *16*, (5), Article Number: 635.
208. He, H.; Sun, N.; Li, L.; Zhou, H.; Hu, A.; Yang, X.; Ai, J.; Jiao, R.; Yang, X.; Wang, D.; Zhang, W., Photochemical transformation of dissolved organic matter in surface water augmented the formation of disinfection byproducts. *Environmental Science & Technology* **2024,** *58*, (7), 3399-3411.
209. Li, Y.; Zhang, K.; Apell, J.; Ruan, Y.; Huang, X.; Nah, T., Photoproduction of reactive intermediates from dissolved organic matter in coastal seawater around an urban metropolis in South China: Characterization and predictive modeling. *Science of The Total Environment* **2024,** *921*, Article Number: 170998.
210. Madhiyan, M.; Moor, K. J., Singlet oxygen quantum yields of pyrogenic dissolved organic matter from lab-prepared and wildfire chars. *Environmental Science & Technology* **2024,** *58*, (2), 1265-1273.
211. Wasswa, J.; Perkins, M.; Matthews, D. A.; Zeng, T., Characterizing the impact of cyanobacterial blooms on the photoreactivity of surface waters from New York lakes: A combined statewide survey and laboratory investigation. *Environmental Science & Technology* **2024,** *58*, (18), 8020-8031.
212. Xie, H.; Li, Q.; Wang, M.; Feng, Y.; Wang, B., Unraveling the photochemical behavior of dissolved organic matter derived from hydrothermal carbonization process water: Insights from molecular transformation and photoactive species. *Journal of Hazardous Materials* **2024,** *469*, Article Number: 133946.
213. Li, L.; Cheng, W.; Xie, X.; Zhao, R.; Wang, Y.; Wang, Z., Photo-reactivity of dissolved black carbon unveiled by combination of optical spectroscopy and FT-ICR MS analysis: Effects of pyrolysis temperature. *Water Research* **2024,** *251*, Article Number: 121138.
214. Wan, D.; Yu, C.; Zhao, Y.; Song, G.; Mi, W.; Zhu, Y.; Liu, C.; Bi, Y., Photodegradation mechanism of organic contaminants mediated by chlorinated algal organic matter. *Water Research* **2025,** *281*, Article Number: 123674.
215. Zhang, J.; Hou, X.; Zhang, K.; Xiao, Q.; Gardea-Torresdey, J. L.; Zhou, X.; Yan, B., Photochemistry of microplastics-derived dissolved organic matter: Reactive species generation and organic pollutant degradation. *Water Research* **2025,** *269*, Article Number: 122802.
216. Luo, H.; Yi, Z.; Wang, Z.; Wang, W.; Yang, Y.; Qin, D.; Qin, F.; Zhou, Y.; Huang, C.; Chen, L.; Zeng, G.; Zhang, C., Molecular insights into dissolved black carbons during adsorptive fractionation: Implications for photochemical reactivity and decontamination. *Chemical Engineering Journal* **2025,** *510*, Article Number: 161657.
